# Identification of over 200-fold more hairpin ribozymes than previously known in diverse circular RNAs

**DOI:** 10.1093/nar/gkab454

**Published:** 2021-06-07

**Authors:** Christina E Weinberg, V Janett Olzog, Iris Eckert, Zasha Weinberg

**Affiliations:** Institute for Biochemistry, Leipzig University, Brüderstraße 34, 04103 Leipzig, Germany; Institute for Biochemistry, Leipzig University, Brüderstraße 34, 04103 Leipzig, Germany; Bioinformatics Group, Department of Computer Science and Interdisciplinary Centre for Bioinformatics, Leipzig University, Härtelstraße 16-18, 04107 Leipzig, Germany; Bioinformatics Group, Department of Computer Science and Interdisciplinary Centre for Bioinformatics, Leipzig University, Härtelstraße 16-18, 04107 Leipzig, Germany

## Abstract

Self-cleaving ribozymes are catalytic RNAs that cut themselves at a specific inter-nucleotide linkage. They serve as a model of RNA catalysis, and as an important tool in biotechnology. For most of the nine known structural classes of self-cleaving ribozymes, at least hundreds of examples are known, and some are present in multiple domains of life. By contrast, only four unique examples of the hairpin ribozyme class are known, despite its discovery in 1986. We bioinformatically predicted 941 unique hairpin ribozymes of a different permuted form from the four previously known hairpin ribozymes, and experimentally confirmed several diverse predictions. These results profoundly expand the number of natural hairpin ribozymes, enabling biochemical analysis based on natural sequences, and suggest that a distinct permuted form is more biologically relevant. Moreover, all novel hairpins were discovered in metatranscriptomes. They apparently reside in RNA molecules that vary both in size—from 381 to 5170 nucleotides—and in protein content. The RNA molecules likely replicate as circular single-stranded RNAs, and potentially provide a dramatic increase in diversity of such RNAs. Moreover, these organisms have eluded previous attempts to isolate RNA viruses from metatranscriptomes—suggesting a significant untapped universe of viruses or other organisms hidden within metatranscriptome sequences.

## INTRODUCTION

RNA is ubiquitous in viruses and organisms, and implements important tasks, such as coding for protein, catalyzing ribosomal protein synthesis (1,2) and gene regulation (3–6). In addition to its biological significance, RNA is an essential tool in many biotechnology applications, such as CRISPR–Cas9-based genomic manipulation (7), artificial sensors and engineered gene control (8,9). Among the most challenging tasks that RNA is known to perform is the catalysis of chemical reactions (10). Such catalytic RNAs, known as ribozymes, provide an opportunity to learn more about RNA biochemistry, structure and biology, and are biotechnology tools.

Nine structural classes of known ribozymes are self-cleaving ribozymes (11–13), which catalyze the breaking of a phosphodiester linkage at a specific position. The biological functions of some self-cleaving ribozymes are known (11,13), and include biochemical steps in the replication of organisms whose genome consists of a circular single-stranded RNA (14). Self-cleaving ribozymes are also known to function in many varieties of retrotransposons (15), which are self-replicating elements with a DNA stage. Ribozymes in retrotransposons separate the element from the rest of its transcript. Self-cleaving ribozymes are involved in gene regulation (16), and further regulatory examples have been proposed (11,13).

Three self-cleaving ribozyme classes are very broadly distributed, having examples in both bacteria and eukaryotes. An additional four classes have hundreds of known members, which provides an ability to understand their structural features and genetic locations based on diverse members. However, each of the remaining two classes, the Varkud satellite (VS) and hairpin ribozymes, are extremely rare. Only one example of the VS ribozyme class (17) has been found. The hairpin ribozyme has been validated in the satellite RNAs of only three viruses: the tobacco ringspot (18), the arabis mosaic (19) and the chicory yellow mottle virus (20). A fourth sequence was predicted computationally (21) in a distinct strain of tobacco ringspot virus satellite RNA. The lack of homologs severely hampered initial studies of the structural and biochemical properties of this ribozyme class. Despite this challenge, truncation and mutation analysis (22–25), *in vitro* selection (26,27), cross-linking (28,29) and other studies have compensated for the phylogenetic gap (30). However, our understanding of the biology of hairpin ribozymes has remained restricted to the four natural examples.

Two classes of ribozymes, the hammerhead and twister ribozymes, are found in nature in multiple permuted forms (11,13). Each permutation exhibits the same secondary structure, but the point at which the 5′ and 3′ ends of the molecules are located vary. As a result, the order in which the conserved elements occur is different in each permuted form.

**Figure 1.**
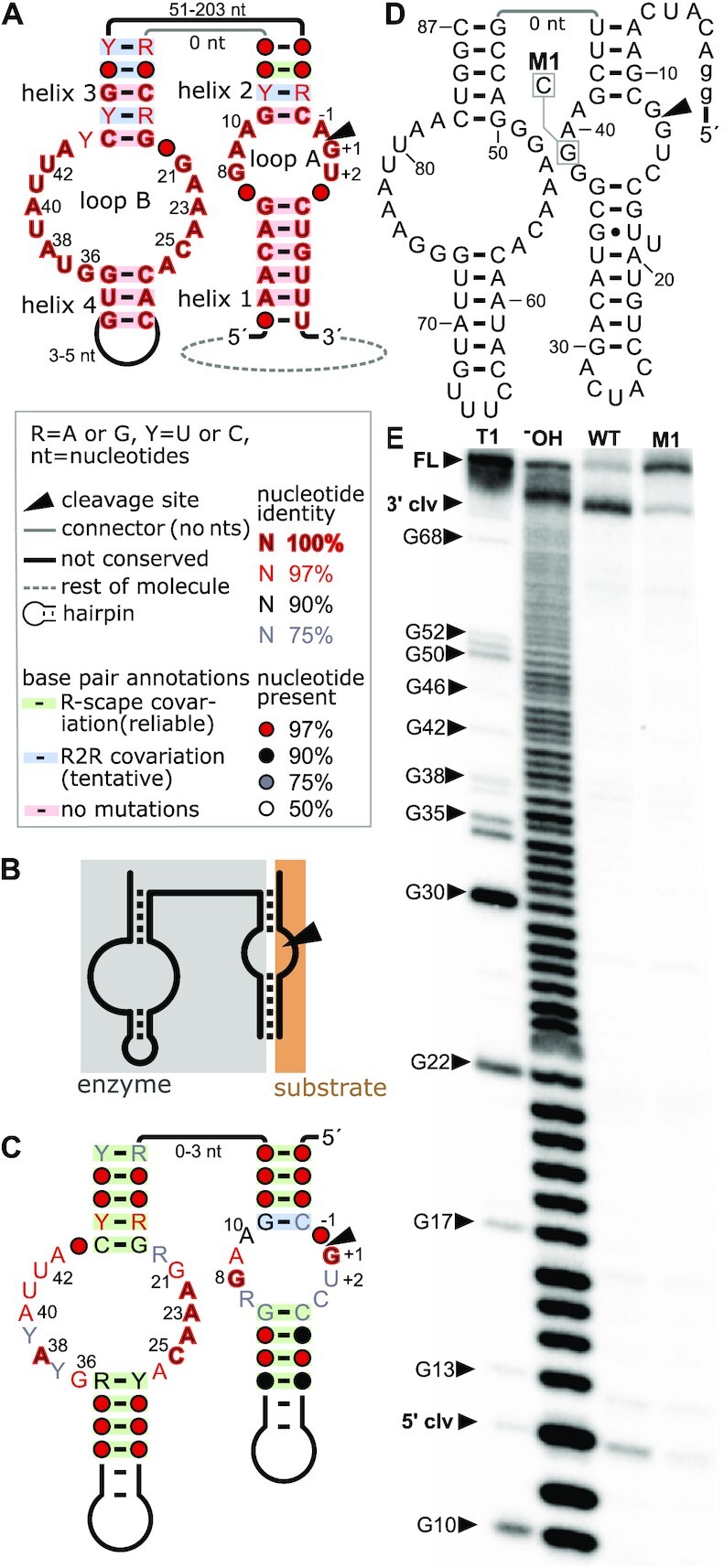
Previously known and novel hairpin ribozymes. (**A**) Consensus diagram of previously known hairpin ribozymes. Many nucleotides appear perfectly conserved (red nucleotides with black fill) merely because only four unique sequences were available. Helices, internal loops and nucleotides are labeled according to the standard nomenclature for hairpin ribozymes. The inset explains symbols used in the diagram and in parts (C) and (D). All four previously known hairpin ribozymes are part of small circular ssRNAs. (**B**) Schematic of bimolecular construct for ribozyme assays, based on the molecule depicted in part (A). The nucleotides corresponding to the upper line are removed, leading to two molecules: the ‘enzyme’ and the ‘substrate’ strand (which contains the cleavage site). (**C**) Consensus diagram of 941 unique novel hairpin ribozymes with a different permutation, but shown in the analogous graphical layout to part (A). Annotations are described in part (A). Due to the greater number and diversity of the newly found hairpin sequences, fewer nucleotides are perfectly conserved and most proposed base pairs are supported by statistically significant covariation, according to the R-scape software (62). (**D**) Secondary structure diagram of the hairpin ribozyme representative HPR 1. M1 indicates a G-to-C mutation leading to a strongly decreased ribozyme cleavage speed. Lowercase ‘g’ denotes artificial nucleotides added to aid transcription. (**E**) Hairpin ribozyme candidate HPR 1 (WT) and mutated variant (M1) were *in vitro* transcribed in the presence of [α-^32^P]-ATP for 5 minutes. Bands correspond to a 3′ cleavage product of 75 nucleotides and a 5′ cleavage product of 12 nucleotides. Full-length transcript is visible for M1 at 87 nucleotides. 5′-radioactively labeled M1 RNAs were used to create size standards by partial digestion with RNase T1 or partial alkaline hydrolysis (‘^−^OH’).

The hairpin ribozyme is generally represented as a large hairpin with internal loops that lack helices (Figure 1A). These loops contain nucleotides that participate in the reaction core (30,31). Because the known hairpin ribozymes occur in small circular RNAs, other permuted forms are equally possible. Originally, hairpin ribozymes were investigated as part of naturally occurring, circular satellite RNAs. Many later studies used a bimolecular construct that consists of an enzyme strand comprising the majority of the catalytic core and a substrate strand, which hybridizes to the enzyme strand and is cleaved. In hairpin ribozymes, the substrate and enzyme strands are typically as shown (Figure 1B) (23,32). By contrast, only some studies investigated the hairpin ribozyme as part of its natural four way helical junction or at least somewhat more extended sequence (31,^33^,^34^,35). To our knowledge, permutations were only considered in one study, although experimentally investigated constructs showed no activity (35).

We sought to take advantage of large sequence databases to find additional examples of hairpin ribozymes, and to explore the possibility that other permuted forms occur in nature. The discovery of many novel hairpin ribozymes permits a detailed biochemical analysis of the ribozymes based on natural sequences, and reveals these hairpins as more widespread than previously recognized. Additionally, such a discovery raises the opportunity to investigate the function of these ribozymes in the uncovered biological context. Moreover, in our case, we found hairpin ribozymes in uncharacterized metatranscriptomic sequences that probably correspond to novel virus-like organisms with unusual genomic characteristics. Thus, the results reveal the limits of current knowledge on organisms with RNA genomes, and suggest a need for new approaches to understand the variety of life on earth.

## MATERIALS AND METHODS

### Sequence databases

We conducted searches on RefSeq (36) version 85. We also downloaded de-novo-assembled metagenomic and metatranscriptomic sequences, predominantly from IMG/M (37) and GenBank (38). Environmental metadata was extracted from these sources.

### Searches for permuted ribozymes

Initial searches were conducted using pattern-based methods, specifically RNAMotif (39) version 3.1.1 or DARN! (40) version 1.0. Once we identified candidate ribozymes based on patterns, we conducted searches with Infernal (41) version 1.1.2. Other procedures are described in Results.

### Co-transcriptional ribozyme cleavage assays

Double-stranded DNA templates containing the T7 promoter sequence 5′-GAAATTAATACGACTCACTATAGG-3′ for *in vitro* transcription were generated using partially complementary oligonucleotides (Supplementary Table S1) and Phusion High Fidelity DNA polymerase (Thermo Scientific). Templates were purified by phenol-chloroform extraction and ethanol precipitation (42). *In vitro* transcription reactions contained 3.34 ng/μl purified DNA template, 80 mM HEPES (pH 7.5 at 23°C), 25 mM MgCl_2_, 2.5 mM of each NTP, 5 mM DTT, 0.005 U thermostable inorganic pyrophosphatase (NEB), 10 % DMSO and 250 ng/μl T7 RNA polymerase (lab preparation) and were incubated at 37°C for 2 h or indicated times. To generate radiolabeled transcripts, 0.25 μCi/μl [α-^32^P]-ATP (Hartmann Analytic) were added to the transcription reaction. Reactions were stopped by adding 3× RNA loading dye [10 mM Tris–HCl (pH 7.6 at 23°C), 80 % (v/v) formamide, 0.25 % bromophenol blue and 0.25 % xylene cyanol] and separated by denaturing (8 M urea) polyacrylamide gel electrophoresis (PAGE) for either immediate cleavage analysis or RNA purification. Unlabeled hairpin ribozyme transcripts containing the mutation M1 were separated by 10 % denaturing PAGE, isolated from the gel by incubation in crush-soak solution [10 mM Tris–HCl (pH 7.5 at 23°C), 200 mM NaCl, 1 mM EDTA] and precipitation with ethanol. Subsequently, 100 pmol purified hairpin M1 RNA were dephosphorylated using Antarctic phosphatase (NEB) according to the manufacturer's instructions and 5′ ^32^P-labeled with T4 polynucleotide kinase (NEB) using 0.1 μCi/μl [γ-^32^P]-ATP (Hartmann Analytic). Labeled hairpin M1 RNAs were separated by 10 % denaturing PAGE and purified as described above. To create size standards, labeled M1 RNAs were partially digested using RNase T1 or subjected to partial alkaline hydrolysis.

### Detection of a circular RNA containing hairpin ribozymes by RT-PCR amplification

The metatranscriptome sample (DNase I-treated total RNA isolated from an environment containing spruce litter) was kindly provided by Petr Baldrian (Czech Academy of Sciences, Prague, Czech Republic). After confirming RNA integrity (Supplementary Figure S1), 250 ng RNA were mixed with 80 pmol reverse transcription primer (Supplementary Table S1), incubated at 92°C for 2 min and chilled on ice for 1 min to anneal the primer to RNA. This mixture was used in 40 μl reverse transcription reactions with SuperScript IV (ThermoScientific) according to the manufacturer's instructions. Samples were incubated for 30 minutes at 55°C. The reactions were stopped by the addition of 3 μl 2 M NaOH, heating to 95°C for 3 min and neutralization using 3 μl 2 M HCl. Resulting cDNA was used in polymerase chain reactions catalyzed by 0.5 U Phusion DNA polymerase (ThermoScientific) in 1× Phusion buffer, 150 μM dNTPs, 500 nM of each forward and reverse primers (Supplementary Table S1). For a 50 μl PCR, 5 μl from the RT reaction were used as template. Annealing temperatures were calculated using the recommended ThermoScientific web tool ‘*T*_m_ calculator’ and PCR amplification was performed for 35 cycles of denaturing at 98°C for 30 s, annealing at primer-specific calculated temperature for 30 s, and elongation for 60 s at 72°C. PCR products were analyzed on a 1 % agarose gel stained with ethidium bromide (Supplementary Figure S2).

PCR products were used for ligation into the pJET1.2/blunt cloning vector and subsequently used to transform *Escherichia coli* TOP10 cells following manufacturer's instructions for the CloneJET PCR Cloning Kit (ThermoScientific). Clones containing RT products of expected sizes were identified by colony PCR using insert-specific primers (Supplementary Table S1) and separation on ethidium bromide-stained 1 % agarose gels. Plasmids were isolated using Gene JET Plasmid Miniprep kit (ThermoScientific), and sequenced with the Sanger sequencing (Microsynth AG) with primer pJET1.2-for (5′-CGACTCACTATAGGGAG-3′).

## RESULTS

### Prediction of novel hairpin ribozymes

Biochemical data (43,30) suggests that the catalytic core of the hairpin ribozyme depends on two internal loops whose nucleotides are critical for ribozyme function (Figure 1A). We speculated that, when a hairpin ribozyme is viewed from the 5′ to the 3′ end, these internal loops could occur in any order relative to each other, and one could be contained within the other (as in the typical representation of hairpin ribozymes). Moreover, each internal loop could be flipped, so that its unpaired regions occur in the opposite order. In total, we enumerated 16 theoretical permutations (Supplementary Figure S3A). We searched for these theoretical possibilities using the DARN! program (40) with search patterns we defined (Supplementary File 1, Supplementary Figure S4).

There were multiple predicted matches to our search patterns. However, due to the large number of sequences we searched, there is a significant possibility of false positive predictions. Some predicted homologs seemed clearly problematic, e.g. all base pairs were A-U or the sequences include extremely large insertions that did not fold compatibly with the expected secondary structure, and we eliminated those based on manual analysis. For each remaining prediction, we performed a homology search with that single sequence using the Infernal software (41) with the secondary structure predicted from the DARN! search. Where Infernal predicted homologs of a putative hairpin ribozyme, we analyzed the alignment it produced. To determine if the alignment is likely to correspond to a biological RNA, we exploited the phenomenon of covariation (Supplementary Figure S4).

Covariation refers to mutations to both nucleotides in a Watson–Crick base pair that result in another Watson–Crick base pair, i.e. the base pair is conserved despite mutations. Alignments can be analyzed for such covariation to determine if the alignment is likely to correspond to an RNA, and to determine its secondary structure (44). Alignments exhibiting compelling covariation, based on our manual analysis, were considered promising.

We found multiple promising alignments that all corresponded to the same novel permutation of hairpin ribozymes (Supplementary Figure S3A), and that each had multiple examples of covariation. We did not find any promising alignments for other permutations including the previously published permutation (Supplementary Figure S4). However, as a positive control, we conducted our search procedure on a sequence database that included RNA satellite genomes that are known to contain hairpin ribozymes in orientations compatible with the previously published permutation. In this case, we did find the previous permutation. Thus, although the two permutations have many conserved features in common (Supplementary Figure S3B), we only see support for the newly found permutation among our newly found hairpin ribozymes.

We took one of the promising alignments in the newly found permutation, and repeatedly searched for additional homologs with Infernal, incorporating these into the alignment for the next round of search. We used an *E*-value threshold of 10 in these searches, but manually eliminated predicted ribozymes that did not appear to fold well. An *E*-value of 10 means that 10 false positive predictions would be expected in a randomized sequence database. We selected this higher (less-stringent) *E*-value threshold in order to explore more diversity in hairpin structures. To validate this approach, we investigated the five predictions we accepted that had the highest (worst) *E*-values, which ranged from 4.8 to 7. Co-transcriptional cleavage assays described in the next section confirm that the predictions correspond to functional ribozymes. Four iterative searches (including the search of the original DARN! sequence) resulted in an alignment we called the ‘initial’ alignment. (All alignments produced in this work are explained in Supplementary Table S2 and are available in computer-readable Stockholm format in Supplementary File 2 and in PDF format in Supplementary File 3.) We also manually improved this alignment, especially in terms of the individual sequences’ predicted secondary structures, creating a ‘touched-up initial’ alignment (Supplementary Table S2, Supplementary Files 2 and 3).

As explained later, it quickly became apparent that most predicted hairpin ribozymes in the initial alignment occurred in metatranscriptomes associated with spruce trees. We therefore downloaded additional metatranscriptome datasets from environments similar to those found to contain many predicted hairpin ribozymes, and performed an additional search with a more stringent *E*-value threshold of 0.1. We eliminated sequences that were truncated on either the 5′ or 3′ end, presumably because of incomplete metatranscriptomic contigs, but otherwise did not manually edit this alignment. This search resulted in an ‘extra-spruce-initial’ alignment (Supplementary Table S2, Supplementary Files 2 and 3), which had 941 unique hairpin ribozyme sequences.

As a further control for false positives, we repeated the search procedure, but used an *E*-value threshold of 0.01 in iterative searches. The lower *E*-value threshold is expected to result in few false positives. We conducted eight iterations of this more stringent regime followed by a search of the extra sequences we downloaded with an *E*-value threshold of 0.1. The result was similar to that with an *E*-value threshold of 10, and yielded ∼1000 predicted hairpin ribozymes (Supplementary Table S2, Supplementary Files 2 and 3). In the remainder of this paper, we analyze the more diverse 941 hairpin ribozyme sequences from our original procedure. These 941 unique hairpin ribozymes show a much richer variety of sequence features (Figure 1C) in comparison to the four hairpin ribozymes previously known (Figure 1A).

### Novel hairpin ribozymes function *in vitro*

To test whether hairpin ribozymes of this new permutation cleave *in vitro*, we chose three diverse representatives from the 941 novel hairpin sequences (Figure 1D, E, Supplementary Figure S5). These representatives were chosen because they contained a relatively high number of highly conserved nucleotides believed to be important for cleavage of the originally described hairpin ribozyme permutation. Additionally, all tested representatives, despite their distinct permutation, conform well to the secondary structural features displayed by hairpin ribozymes, i.e. helices 1–4 as well as loops A and B (Figure 1A, C). We transcribed the hairpin candidates *in vitro* (see Materials and Methods for details) and separated the radiolabeled transcription products by polyacrylamide gel electrophoresis (PAGE, Figure 1E, Supplementary Figure S5). Distinct bands corresponding to 5′ and 3′ cleavage products were observed for all representatives. The cleavage reaction appears to be nearly complete, as no full-length product can be observed. Full-length transcript is only visible for an RNA variant (M1) where a highly conserved G nucleotide is mutated to a C (Figure 1D, Supplementary Figure S5). An analogous mutation was shown to decrease ribozyme cleavage speed by 300-fold in a previously studied hairpin ribozyme derived from tobacco ringspot virus satellite RNA (45). Thus, all tested hairpin ribozyme candidates effectively cleave during *in vitro* transcription and yield cleavage products that conform to the expected fragment sizes. Moreover, the M1 mutation significantly reduces ribozyme speed, as expected.

We next tested hairpin ribozymes that were predicted at high *E*-values during our search procedure. These sequences often had deviations from otherwise conserved nucleotides or had long, complex structures outside of the conserved core (Supplementary Figure S6). Co-transcriptional assays showed that these sequences are catalytically active (Supplementary Figure S7). We also tested five additional sequences that were selected because they had deviations in features that were previously established as important. These ribozymes are discussed in the next section.

### New hairpin ribozymes complement previous biochemical studies

**Figure 2.**
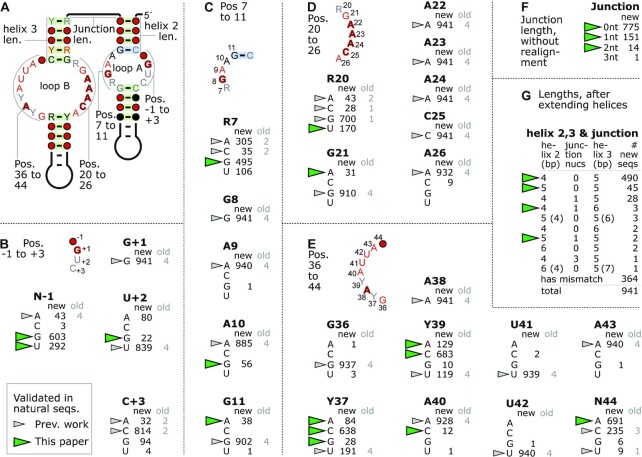
Detailed conservation analysis of 941 hairpin ribozymes. (**A**) The indicated seven regions of particular structural importance in the hairpin ribozyme are analyzed in parts B-G. Base pairs in non-Watson–Crick interactions are analyzed in Supplementary Figure S8. (**B**) The number of each of the four nucleotides observed in each indicated position is given. For perfectly conserved positions (e.g. G+1), only the relevant nucleotide is shown. Nucleotide positions are named after the conserved nucleotide in part A (‘N’ = no specific nucleotide was conserved) and the standard hairpin ribozyme position number. For example, the previous name U39 is now Y39 in this figure. ‘new’: numbers of sequences out of the 941 novel hairpin ribozymes. ‘old’: numbers based on the previously established four hairpin ribozymes. Nucleotides that were present in experimentally validated natural sequences prior to the current paper are indicated by gray triangles. Nucleotides present in any of the 941 sequences that we confirmed as functional are indicated by green triangles (details in Supplementary Table S3). These definitions and the boxed legend also applies to parts C–G. (**C**) Conservation of positions 7–11. (**D**) Conservation of positions 20–26. (**E**) Conservation of positions 36–44. (**F**) Lengths of the junction between helices 2 and 3. These calculations ignored the possibility of non-canonical pairs or indels in the computationally predicted helices, and the possibility that junction nucleotides could extend a helix. Old sequences are not analyzed because the junction is specific to our proposed permutation. (**G**) Analysis of helices 2 and 3 with the junction. Here, 364 sequences with a mismatch or indel in predicted helix 2 or 3 pairs were not analyzed. We added junction nucleotides to helix 2 or 3 if they would form Watson–Crick or G–U pairs. In rare cases, it was possible to add a given nucleotide to either helix 2 or helix 3, and the alternate possibilities are given in parentheses. Note: non-Watson–Crick base pair interactions are not depicted or analyzed in Figure 2, but are in Supplementary Figure S8 (see also Supplementary Table S4).

Our expanded set of 941 unique hairpin ribozymes provides additional information to evaluate previously determined biochemical data. Numerous biochemical studies and several crystal structures have enabled an in-depth analysis of the hairpin ribozyme core and nucleotides taking part in catalysis (30,31,46). These studies are corroborated and extended by the expanded phylogeny now available with 941 unique examples instead of four (Figure 1C, Figure 2, Supplementary Figure S8). Within the region depicted in Figure 1A, there are 43 positions with perfectly conserved nucleotides in all four previously published hairpin ribozymes. By contrast, the updated alignment has only seven such positions (Figure 1C), suggesting that these positions are especially important for ribozyme function.

There are several nucleotide positions in hairpin ribozymes that perform vital tasks, and should thus be quite intolerant of mutations. At minimum, these include A38 and G8, which act as general acid and base, respectively, in the acid-base catalyzed ribozyme cleavage reaction (45,47–51), as well as G+1 and C25 (Figure 2A) (31). Indeed, these four positions are perfectly conserved in all 941 predicted hairpin ribozymes. Nucleotide G+1 forms a Watson–Crick base pair with C25 (Figure 2B, D, Supplementary Figure S8A, D) that stabilizes the docking of loop A and B, and generates a splayed conformation required for in-line nucleophilic attack at the scissile phosphate (31,47). It was suggested that the G+1–C25 base pair is possibly further strengthened by an interaction with A9 (conserved in 940 of 941 sequences, Figure 2C), although an alteration of this nucleotide had only little effect on ribozyme folding in previous studies (47) and such a proposed triple interaction has not been observed in the crystal structure (31). Instead, the exocyclic amine of A9 could play a role in transition state stabilization (46).

In addition to G+1, G8, A38 and C25, the nucleotides A22, A23 and A24 (Figure 2D) are fully conserved with no exceptions in 941 new sequences. Previous mutation studies had already found that position A22 and A23 do not tolerate a mutation to any other nucleobase (52). For position A24, *in vitro* selection studies showed that this nucleotide was completely retained (27). Later, mutational studies confirmed that replacing A22, A23 or A24 with non-wild type nucleobases render the ribozyme either completely inactive or at least 150 times slower than the unmodified hairpin ribozyme (52).

Other nucleotide positions vary, and some of these nucleotides were previously assigned structurally important roles. We tested several deviations to nucleotides in important regions of the ribozyme (Supplementary Table S3), and found that all tested ribozymes were able to cleave. However, we generally did not investigate very rare variants (e.g. fewer than 1 % of the 941 hairpin ribozymes). Very rare mutations might result from sequencing errors in the underlying metatranscriptomic data, and also could correspond to pseudo-ribozymes that have recently acquired deleterious mutations, but are still present in the sample.

Previously studied hairpin ribozymes contain a four-way junction whose stems form two coaxial stacks. Two of these stems dock to form the active ribozyme core by bringing together loops A and B (31,33,46,53). We evaluated less-conserved positions by co-transcriptional cleavage assays (Supplementary Figures S9 and S10) and based on existing literature.

Most important positions in loops A and B, including less-conserved nucleotides, participate in non-standard base-pair interactions (Supplementary Figure S8). Most of these interactions can be classified according to Leontis-Westhof Notation (54), as in previous studies (55,56). Previous work has determined which nucleotide combinations are isosteric for different types of base pairs (54), i.e. which nucleotide combinations can have the same geometric relationship. We observe many isosteric nucleotide substitutions (Supplementary Figure S8), but also several that are not expected to be isosteric. For example, the G11–C-2 pair is a *cis* Watson–Crick interaction (Supplementary Figure S8B) with rare G11→A substitutions. The resulting A–C pair is not isosteric, but we confirmed that the relevant sequence is catalytic (Supplementary Figure S9). We also confirmed that other examples of mutations not expected to be isosteric are present in active ribozymes (Supplementary Figure S8, Supplementary Table S4). Beyond nucleotide substitutions that are not expected to be isosteric, four sequences with a G–G interaction in positions R20 and N44 should not be able to interact in the *cis* Watson–Crick mode observed at this position in the previously published hairpin ribozyme crystal structure (31). We presume that there is a certain degree of flexibility in the structure such that the precise spatial relationships observed in the previous crystal structure need not be rigorously conserved, or that the resulting spatial distortions are compensated by mutations elsewhere in the RNA. It is also possible that some of the substitutions we observed, e.g. the G–G pair just mentioned, lead to ribozymes that cleave less efficiently, but their cleavage speeds may be biologically adequate. Additionally, very rare nucleotide combinations could conceivably result from sequencing errors or pseudo-ribozymes.

Focusing on loop A, we analyzed the A10 and G11 positions, which also sometimes occur as an A10→G and G11→A variation (Figure 2C). In addition to their base pair interactions (Supplementary Figure S8), these nucleotides are involved in forming a ‘ribose zipper’ between the hydroxyl groups of A10, G11 and C24, A24 and N3 base atoms of A10 and A24 (Supplementary Figure S8) (57). When we assessed their ability for autocatalytic cleavage during transcription, we found the less common ribozyme variants A10→G and G11→A are still able to cleave themselves (Supplementary Figure S9). R7, also in loop A, is less conserved and most sequences contain either a G or A at this position (Figure 2C). While all nucleotides at this position have been found to support cleavage, the slight phylogenetic preference for purines at this position is in agreement with an early mutational study. It found that hairpin ribozymes with G7 or A7 nucleotides cleave slightly faster than representatives with pyrimidines at this position (58).

Except for G+1, nucleotides between positions -1 to +3 are far less conserved than the invariant nucleotides in the previously available four sequences suggested (Figure 1A). In the N-1 and C+3 positions (Figure 2A), for example, all four nucleotides are observed in the 941 hairpin ribozymes (Figure 2B). These findings are in accordance with mutation studies that found no significant reduction in cleavage speed for N-1 and only mildly slower cleavage speeds for non-wild type base substitutions at C+3 (58).

In loop B, positions 36–44 together with positions 20–26 (Figure 2D, E, Supplementary Figure S8C) form an irregular helix containing six non-canonical base pairs that are part of a cross-strand purine stack motif and a bulge S-turn (31,59). The helix starts with an interaction between R20-N44 as a *cis* Watson–Crick base pair which is most often followed by a sheared G21–A43 pair that marks the beginning of the cross-strand purine stack (Supplementary Figure S8). Sometimes, the G21 in this interaction is replaced by an A (Figure 2D), which can equally form an isosteric *trans* sugar-Hoogsteen interaction (Supplementary Figure S8C). We confirmed that natural, predicted hairpin ribozymes with this mutation efficiently cleave (Supplementary Figure S9), mirroring mutational studies in which the G21→A variant was also found to be active with a ∼15-fold reduced cleavage speed compared to wild type (52). This base pair is almost exclusively followed by a *trans* interaction between the Hoogsteen edge of A22 and the Watson–Crick edge of A41 (Figure 2D,E, Supplementary Figure S8C). Nucleotides U41 and U42 have been suggested to be important, for example by selection experiments where no mutations at these sites were identified (26), as well as several mutation studies (25,52,60), that showed U41 greatly facilitates cleavage and U42 is essential. The larger set of identified hairpin ribozymes underlines this test tube finding, as there are only one (U42) or two (U41) alternate nucleotides identified in 941 natural sequences (Figure 2E). These very rare nucleotide substitutions could, for example, result from sequencing errors or pseudo-ribozymes as mentioned above. A38 is perfectly conserved and has been found to form a single hydrogen bond with A24, in addition to its role as general acid in the acid-base catalyzed cleavage reaction (31,50).

In position 39, only a U nucleotide has previously been observed in natural hairpin ribozyme examples (Figures 1A and 2E). However, *in vitro* selection experiments and mutation studies systematically examined all base substitutions at this position (27,52). An A nucleotide lends activity comparable to U, while a G nucleotide is less efficient (27,52). Two independent studies even suggest a modest increase in ribozyme cleavage speed for the U39→C mutation (27,61). The 941 new hairpin ribozymes show a preference for C at this position (Figure 2E), with A and U more common than G. Thus, the previous *in vitro* data is reflected in nucleotide preferences in the larger number of natural sequences now available.

In terms of helical regions, the ribozyme core consists of four helices, numbered 1 to 4. In the previously established four hairpin ribozymes (Figure 1A), no nucleotides in helices 1 and 4 vary, and some in helices 2 and 3 are also invariant. Therefore, they do not exhibit covariation. Among the 941 new hairpin ribozymes, all but one base pair (G11–C-2) exhibit statistically significant covariation (Figure 1C) according to the R-scape program (62).

While most previous studies tested only the core region of the hairpin ribozyme containing helices 1–4 and loops A and B (Figure 1A), several studies have analyzed larger sequences that contain auxiliary domains in terms of their influence on ribozyme folding and activity (33–35,63–66). The hairpin ribozyme in the tobacco ring spot virus satellite RNA, which was frequently used for these studies, folds into a four-helix junction (33). This junction includes helices 2 and 3, which form part of the ribozyme core, and two other less-conserved helices. No unpaired nucleotides are present in this four-helix junction. It was found that the presence of the junction accelerates folding of the hairpin ribozyme by nearly 1000-fold (65). Moreover, it was also shown that the hairpin ribozyme that includes the four-helix junction efficiently cleaves *in vitro* at 10 mM MgCl_2_ with cleavage speeds that are about half that of the minimal core region under the same reaction conditions (33). At the same time, constructs containing the junction require ∼100–1000 times less Mg^2+^ for folding than the minimal hairpin ribozyme construct (33,34,64). The addition of magnesium ions induces a transition of the junction into an antiparallel conformation that brings loops A and B together, which is integral to ribozyme activity (63,64). Hairpin ribozymes were also previously known in RNA satellites of the arabis mosaic and chicory yellow mottle viruses. Proposed secondary structure models for these ribozymes also include multi-stem junctions broadly similar to those of the tobacco ring spot molecule (35). However, some unpaired nucleotides occur in the junctions and the chicory yellow mottle ribozyme has a proposed five-stem junction.

Our search patterns for hairpin ribozymes only modeled the core hairpin ribozyme. We subsequently used CMfinder (67) and manual inspection to find additional stems supported by covariation that might correspond to a multi-stem junction. However, we did not observe compelling evidence of additional stems. Perhaps the extended structures among the newly found hairpin ribozymes evolve too rapidly to be detected by comparative means. Another possibility is that the structure in Figure 1C does not require additional auxiliary stems.

We investigated the junction between helices 2 and 3 in the new hairpin ribozymes. Most junctions contain zero nucleotides, which is similar to the hairpin ribozyme in tobacco ringspot virus satellite RNA. There are some exceptions having one, two or even three unpaired nucleotides at the junction, according to automated alignments (Figure 2F).

In several cases, unpaired nucleotides predicted between helices 2 and 3 can participate in base pairs that would extend one or both of these helices. Even under the assumption that the helices can be extended, most sequences have a helix 2 and 3 of 4 and 5 bp, respectively, as in previously studied hairpin ribozymes (Figure 2G). (We did not analyze 364 predicted hairpin ribozymes with a non-canonical pair in helix 2 or 3, as it is quite possible that these could result in bulged nucleotides instead of pairs.) The most common helix lengths we observed (4 base pairs for helix 2 and 5 for helix 3) are consistent with biochemical experiments showing that truncating or extending these helices leads to decreased ribozyme activity (68,69). However, there are numerous cases among the 941 new hairpin ribozymes where helix 2 or 3 are longer than these standard lengths. We investigated three hairpin ribozyme examples predicted to include an extra base pair in either helix 2 or 3, with two examples containing an unpaired nucleotide between these helices, and found each representative to be active in co-transcriptional experiments (Supplementary Figures S10 and S11). Thus, helices 2 and 3 tolerate some variation in their lengths, although the majority of predicted hairpin ribozymes correspond to the lengths that were previously established to be optimal. Helices 1 and 4 have consistent lengths in the previously found four hairpin ribozymes, but their lengths vary greatly in the 941 new ribozymes. This variability is in agreement with experimental findings that the helices can be extended to at least 27 bp (helix 1) and 25 bp (helix 4) (70–72). The hairpins we tested had varying helix 1 and 4 lengths and were functional.

The phylogenetic diversity in the expanded set of hairpin ribozyme examples complements years of biochemical analysis of mutated hairpin ribozymes (22–25), functional ribozyme variants isolated by *in vitro* selection (26,27) and other experiments (30). Highly conserved nucleotides in the expanded consensus (Figure 1C; 97 % or 100 % conserved nucleotides in loops A and B) match previously published biochemical and structural evidence (30,31,50). Thus, our results based on 941 unique biological sequences are essentially consistent with previously established results, and further support their biological relevance. Given the fact that the most-critical nucleotides are 100 % conserved in the 941 ribozymes and given the overall similarities to previously determined hairpin ribozyme biochemistry, we infer that the newly found hairpin ribozymes implement essentially the same catalytic mechanism as previously determined. However, it also appears that additional structural variation is present in several positions that was not previously known in natural sequences.

### Hairpin ribozymes were detected only in RNA molecules

We did not find hairpins in genomic or transcriptomic sequences corresponding to any identified organism in the RefSeq nucleotide database (36). We also did not find any hairpins in metagenomes, i.e. sequences derived from DNA molecules. Rather, all sequences came from metatranscriptomes, and thus RNA. To confirm this finding, we searched metatranscriptomic and metagenomic sequences from environments that most frequently contain hairpin ribozymes. In total, 3210 hairpin ribozymes (including duplicate sequences in distinct contigs) were predicted in an automated search of 5933 megabases of metatranscriptomic data, but zero hairpin ribozymes were found in 20 750 megabases of metagenomic data from the same studies using the same search procedure (Supplementary Table S5).

To identify specific organisms containing hairpin RNAs, we searched for hairpin ribozymes within the Transcriptome Shotgun Assembly (TSA) sequence database (38). We found hairpin ribozymes in the transcriptomes of multiple organisms, including fungi, plants and metazoans (Supplementary Table S6).

Given that we found no hairpin ribozymes in DNA sequences, it is unlikely that the RNA molecules with hairpin ribozymes correspond to gene transcripts or retrotransposons (including retrozymes), which both have DNA forms. There are two caveats to this conclusion to consider. First, we cannot absolutely rule out the new hairpins as having biologically important DNA forms, although such forms were not detected. Second, additional hairpin ribozymes might be found in the future that are present in DNA forms. Indeed, many other self-cleaving ribozyme classes are found in genomic and metagenomic sequences, and examples have been established in mRNAs and retrotransposons (11,13). In view of these observations, we hypothesize that at least the hairpins found in this work occur in organisms that use RNA as their genome, and have no biological role as DNA.

### Hairpin ribozymes likely occur in circular single-stranded RNAs (ssRNAs)

Because we used assembled metatranscriptomic data, the sequences consist of ‘contigs’, which are fragments of a larger RNA molecule. In looking at the hairpin-containing contigs, we did not find any predicted protein-coding genes that match known conserved protein domains in the Conserved Domain Database (73). However, we observed hammerhead and twister ribozymes on the opposite strand to that of many hairpin ribozymes, based on established search procedures (21). This observation is significant in view of previously established ribozyme biology.

Previously known hairpin ribozymes occur in satellite RNAs, which are RNAs that depend on a helper virus to propagate. The virus provides essential functions such as coat proteins to package the satellite RNA (74). Circular satellite RNAs replicate in a process known as rolling-circle replication in which self-cleaving ribozymes assist by cleaving and ligating copies of the satellite RNA (11,13). Other self-cleaving ribozymes facilitate the replication of viroids, which are viruses that infect plants and whose genome is also a single-stranded circular RNA (74). In contrast to satellite RNAs, viroids only rely on the host organism for their replication and do not need additional molecular machinery from a helper virus (74).

The satellite RNAs that are known to contain hairpin ribozymes are circular, single-stranded molecules of 300–457 nucleotides in length and contain a hairpin ribozyme in one strand and a hammerhead ribozyme in the reverse strand (74). Each ribozyme plays a vital role in the rolling-circle replication of either the satellite RNA’s sense or its antisense strand, which explains why they occur on opposite strands. Although known satellites with hairpin ribozymes always contain a hammerhead ribozyme, some other satellites have only one hammerhead ribozyme, and use an asymmetrical version of rolling-circle replication. An intermediate stage during rolling-circle replication is that of a linear ssRNA molecule with multiple adjacent repeats of the complete genome, called ‘multimeric repeats’. Each repeat is then processed to separate it from other repeats and circularize it (75).

It is striking that contigs with hairpin ribozymes often contain ribozymes on each strand, a property similar to known self-cleaving ribozymes that participate in rolling-circle replication. We therefore hypothesized, first, that the hairpin-ribozyme contigs also correspond to circular ssRNA molecules that have an intermediate stage as multimeric repeats; second, that the ribozymes facilitate the replication of the molecules using asymmetrical or symmetrical rolling-circle replication; and third, that these circular molecules function as viroids, satellite RNAs or possibly viruses.

**Figure 3.**
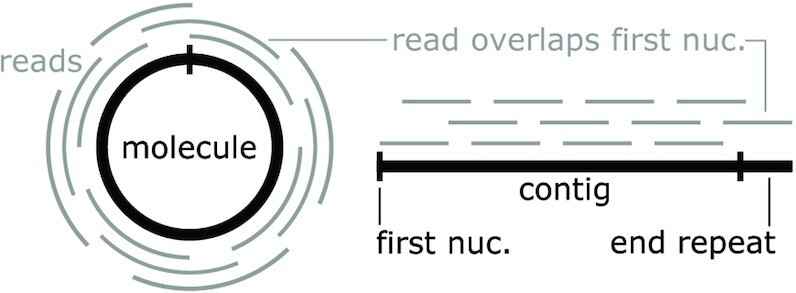
Circular molecules can lead to linear contigs with end repeats. (Left) A circular molecule (black) is subjected to high-throughput sequencing. The short vertical line indicates the first nucleotide in the resulting contig. The reads (gray) map to random sections of the molecule. (Right) The reads are assembled into a linear contig with an end repeat, and the first nucleotide (vertical black line) is repeated. In the diagram, one read overlaps the first nucleotide, and its presence leads to the end repeat, to which it partially maps.

To analyze whether the hairpin ribozymes occur in circular molecules, we took into account the fact that most software that assembles metagenomic/metatranscriptomic reads into contigs assumes that the molecules will be linear (76). If reads cover the entire circle, no linear molecule can accurately represent it. In this case, an essentially arbitrary nucleotide is selected as the first position. The read(s) that overlap this first position will then map partially to the beginning and partially to the end of the assembled linear sequence. In many sequence assemblers, this non-linear mapping will cause the sequence to be extended in the 3′ direction, so that all reads fully map to a contiguous, linear region. In such cases, a region at the 5′ end of the sequence will exactly match the extended 3′ end. The length of this matching region can depend on the length of reads and properties of the metatranscriptome assembler. We call these matching regions ‘end repeats’ (Figure 3).

We searched for end repeats of at least 75 nucleotides in contigs containing a predicted hairpin ribozyme, e.g. the first 75 and last 75 nucleotides in the contig are identical. The predicted hairpin ribozymes (including identical sequences in multiple contigs) were contained in 1653 contigs. Of these 1653 contigs, 524 (32 %) have end repeats, mostly of 123 nucleotides. End repeats are 100-fold more frequent in hairpin-containing contigs compared to other contigs (Supplementary Table S7), which further suggests that the hairpin-containing contigs are derived from circular molecules. Note that, due to incomplete sequencing coverage, we do not expect that 100 % of the contigs will be complete, and therefore it is expected that many will lack end repeats. Base calling errors can also cause a failure to detect end repeats, since we require an exact match. Additionally, our metatranscriptome sequences are aggregated from multiple studies, and some might use assemblers that do not produce end repeats long enough for us to recognize (76). We assumed that contigs with end repeats represent complete molecules in the following analysis.

We also compared each hairpin-containing contig to itself with BLAST. It would be conceivable that contigs could be formed of tandem repeats of a molecule that do not perfectly match each other due to sequencing errors or heterogeneous sequences. In this case, the apparently long contigs would correspond to smaller molecules. However, apart from end repeats, we did not find any other types of self-similarity in the sequences with BLAST (Supplementary Text). Therefore, the contigs do not consist of artifactual tandem repeats.

### Experimental analysis of a circular ssRNA containing a predicted hairpin ribozyme

Our analysis of metatranscriptomic data suggested that hairpin-containing molecules exist in circular forms and as multimeric repeats. To better support this conclusion, we used reverse transcription (RT) PCR to analyze RNA directly. Hairpin ribozymes are highly enriched in RNA from spruce-tree-associated environments known as spruce litter (77). So, we analyzed RNA that was isolated (Supplementary Figure S1; and see Methods) from this environment.

Complicating our analysis, metatranscriptomes contain a highly heterogenous mixture of different RNA molecules. We therefore decided to target a specific RNA molecule (i.e. contig) with RT-PCR. We chose a molecule that is relatively abundant in multiple spruce-litter-associated metatranscriptomes, is relatively short and that exhibits end repeats. Based on these criteria, we selected a 735-nucleotide contig with sequence accession Ga0247519_111615, available in the IMG/M database (37) metatranscriptome with accession 3300023556. This contig has an end repeat of 123 nucleotides, so we inferred that the corresponding RNA molecule is 735-123 = 612 nucleotides in length. To confirm that this contig contains a hairpin ribozyme, we verified that the predicted hairpin ribozyme in this contig is catalytically active *in vitro* (Supplementary Figure S11).

**Figure 4.**
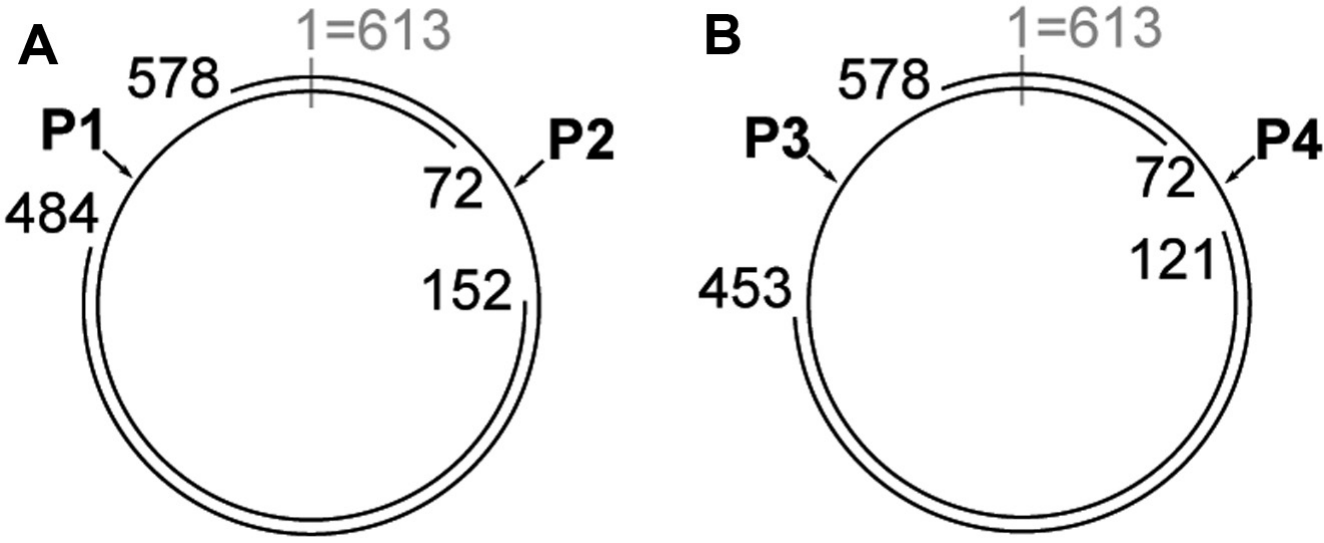
RT-PCR experiments detected RNA molecules containing hairpin ribozymes. RT-PCR experiments were conducted that targeted specific regions of a circular molecule that was predicted based on a metatranscriptome contig (see text). The sequences of these RT-PCR products matched the expected sequence perfectly. Other PCR products presumably reflected sequences of related organisms. (**A**) Sequences corresponding to the strand containing the hairpin ribozyme. The 612-nucleotide circular molecule is depicted with nucleotide position 1 at the 12 o’clock position. In all cases, PCR primers were at most 25 nucleotides long. One confirmed sequence (P1) started at position 152, ran through position 1 and ended at position 72. The other (P2) went from positions 578 to 484, again via the origin position. These sequences overlap and together span the entire circle. (**B**) Detection of the strand containing the reverse-complement of the hairpin ribozyme. Some coordinates differ from those in part (A) because of differences in primer locations. Two overlapping RT-PCR products were generated (P3, P4).

To detect the ssRNA molecule corresponding to this contig, and to determine its circularity, we performed reverse transcription experiments followed by PCR amplification. These two experiments were designed to yield two cDNAs/PCR products corresponding to two different, overlapping regions of the expected 612-nucleotide, hairpin-ribozyme-containing RNA. The RT-PCR experiments detected the expected molecules, and are thus consistent with the hypothesis that this RNA molecule is present as circular RNA or as linear multimeric molecule in the environmental sample (Figure 4A, Supplementary Figure S2). In particular, sequencing of the RT-PCR reactions revealed an exact match to the relevant parts of the expected 612-nucleotide RNA. Note that it is not possible to distinguish circular molecules from multimeric repeats using this experiment, but both are consistent with our hypothesis, and importantly, the RT-PCR results strongly suggest that the molecule is not linear. Similar experiments support the occurrence of the reverse-complement strand, in circular or multimeric form (Figure 4B). Although we hypothesize that the RNA molecule is single-stranded, reverse-complement strands are necessarily produced during rolling-circle replication. Therefore, it is expected that we would observe both strands.

### Hairpin ribozymes provide a window into as-yet unknown and diverse organisms

**Figure 5.**
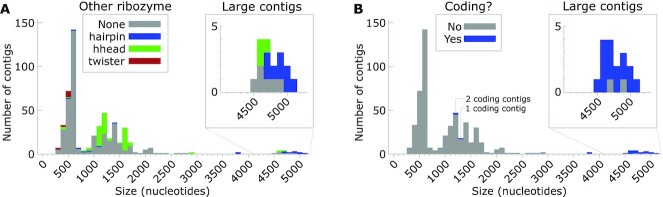
Size distribution, ribozyme and gene content of hairpin-containing contigs. (**A**) Histogram of all contigs with end repeats (i.e. that are believed to correspond to complete circular ssRNA molecules) that contain a hairpin ribozyme. The sizes of the inferred circular ssRNAs corresponding to each contig is plotted. The total heights of the bars indicate the number of contigs corresponding to a circular molecule in the relevant size range. The heights of each colored bar represent the number of those contigs with the relevant opposite strand property. ‘Other ribozyme’: ribozyme class present on the reverse strand, relative to the hairpin ribozyme. (‘hhead’ = type-III hammerhead ribozyme, ‘twister’ = type-P1 twister ribozyme.) Some contigs contain a hairpin ribozyme in both strand orientations. ‘Large contigs’: depicts the rarer large contigs. (**B**) Histogram of the same contigs as in part (A). ‘Coding’: indicates whether at least one region exhibited significant evidence of protein-coding, according to RNAcode.

In order to analyze the properties of complete contigs, we restricted our further analysis to contigs containing end repeats. The range of sizes of the inferred corresponding RNA is strikingly large: from 381 to 5170 nucleotides (Figure 5A). Most, but not all, small RNA satellites and viroids lack protein-coding genes. However, larger ssRNAs (e.g. viruses and larger RNA satellites) typically have such genes. We investigated whether the complete hairpin-containing contigs likely code for protein using the RNAcode software (78) (see Supplementary Text). Most large contigs (>4500 nucleotides) with hairpin ribozymes appear to encode proteins, but smaller contigs overwhelmingly do not (Figure 5B).

The metatranscriptomes in which hairpin ribozymes are found come from a variety of environments (Supplementary Tables S8, S9). Hairpin ribozymes occur most frequently in metatranscriptomes from environments associated with spruce trees (Supplementary Text).

**Figure 6.**
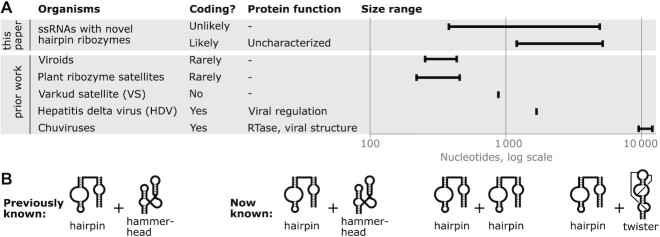
Circular ssRNAs inferred from hairpin-ribozyme-containing contigs have properties differing from previously known circular single-stranded RNA genomes. (**A**) Size ranges and coding potential. ‘This paper’: novel ssRNAs with hairpin ribozymes. ‘Prior work’: previously established circular ssRNA genomes. ‘Organisms’: novel or previously known organisms with circular ssRNA genomes. Hairpin-containing ssRNAs with predicted coding regions are analyzed separately from those that likely do not code. ‘Plant ribozyme satellites’: plant RNA satellites with self-cleaving ribozymes. ‘Coding?’: coding potential. ‘Protein function’: roles of proteins encoded by virus, where coding is typical. Not all Chuvirus protein functions are listed. ‘Size range’: shortest and longest known examples. Hairpin-containing ssRNAs have a much larger range of sizes than previously identified types of organisms. (**B**) Combinations of self-cleaving ribozymes known on opposite strands that involve hairpin ribozymes. ‘Previously known’: only the hairpin ribozyme on one strand and hammerhead ribozyme on the other was known. ‘Now known’: includes additional combinations found in this work.

We compared the hairpin-containing contigs to other organisms with replicating circular ssRNAs, to see how similar their genomic properties are. We have hypothesized that hairpin-containing contigs correspond to circular ssRNAs that can replicate via a rolling circle mechanism. Replicating RNAs use themselves as a template to replicate, in contrast to RNAs that are transcribed from a DNA template, such as gene transcripts. Some replicating forms of ssRNAs are known that form covalently bound circles, including viroids and circular satellite RNAs (74,79). We enumerated the sizes and coding potential of known replicating circular ssRNAs for the purposes of comparison (Figure 6A; and see Supplementary Text).

In comparison to these replicating circular ssRNAs from viroids, RNA satellites or viruses, hairpin-ribozyme-containing contigs show unusual properties (Figure 6A). Many of the inferred circular ssRNAs containing hairpins are larger than 4500 nucleotides. This size is more than twice as long as genomes of known viroids or circular RNA satellites. Although Chuviruses are larger, they contain reverse transcriptase genes. By contrast, the hairpin-containing contigs do not encode reverse transcriptases, or indeed any characterized protein domain. Moreover, the sizes of hairpin-containing molecules have a range of >10-fold, which is a larger ratio than the other known ssRNA forms.

The hairpin-containing contigs also differ in their use of self-cleaving ribozymes from previously characterized viroids or satellites (Figure 6B). Twister ribozymes have not previously been detected in viroids or satellite RNA. We observe 11 twister ribozymes in hairpin-containing contigs ranging from 394 to 564 nucleotides in length. There are also no previously known RNAs that have hairpin ribozymes on both strands, but 14 metatranscriptomic contigs ranging in length from 505 to 5170 nucleotides have this property.

In addition, we did not find any sequence similarity between the contigs with hairpin ribozymes and known ssRNA organisms, other than approximate matches between self-cleaving ribozymes (Supplementary Text). Thus, the hairpin-ribozyme-associated contigs might represent new lineages of organisms, and are certainly significantly diverged from currently characterized sequences.

### Search for permuted forms of other self-cleaving ribozyme classes

We investigated certain other classes of self-cleaving ribozymes that might have circularly permuted forms that have not yet been detected in biological sequences (Supplementary Table S10, Supplementary File 1). However, we did not find persuasive evidence of other not-yet-discovered permuted forms of ribozymes.

## DISCUSSION

To date, only four unique hairpin ribozyme sequences were found, despite the hairpin ribozyme's discovery >30 years ago and the vast amount of sequence data available today. Our results show that the hairpin ribozyme is more common than previously thought. The additional sequences provide data that allows for a comparative analysis of natural hairpin ribozymes. Additionally, the discovery of these hairpin ribozymes revealed organisms that likely consist of replicating, virus-like, circular ssRNAs. Moreover, these ssRNAs exhibit properties that are unlike currently known ssRNAs, and could suggest new lineages of organisms that have previously remained hidden. Separately, the vastly expanded occurrence of hairpin ribozymes might shed light on new biological significance of this ribozyme class.

We tentatively concluded that the hairpin ribozymes discovered in this paper are present in single-stranded circular RNA molecules. This hypothesis was suggested by the presence of self-cleaving ribozymes on opposite strands, and the precedent that this phenomenon is well-known in circular ssRNAs. The circularity of the molecules containing hairpin ribozymes was further supported by end repeats in metatranscriptome sequences and by our RT-PCR experiments. By contrast, there is no direct evidence that the molecules are predominantly single-stranded. However, if the molecules generally occur in double-stranded form, this would be the first observation of a self-cleaving ribozyme in double-stranded RNA (dsRNA). Therefore, the hypothesis of single-strandedness is more conservative.

In the context of a circular RNA, it could be argued that there is no one correct circular permutation. However, several of our hairpin ribozymes occur in molecules larger than 4000 nucleotides (Figure 5), which means that the permutation we propose (Figure 1C) is more likely to be biologically relevant than the alternative, which would have a roughly 4000-nucleotide insertion in it. By contrast, the previously known hairpin ribozymes are present in molecules ranging in size from 300 to 457 nucleotides, in which case there is more ambiguity.

There is already a large body of biochemical and structural data available for the hairpin ribozymes discovered in the 1980s (30,31). Many of these experiments are based on the investigation of ribozyme characteristics using bimolecular constructs (30). These bimolecular constructs used previously are generally of the same design that would be employed for the novel hairpin ribozyme permutation (Figure 1A,B,C). Therefore, we expect that the biochemical characteristics of the new hairpin ribozymes in their alternate permutation (e.g. speed or compatibility with different metal ions) are likely to be similar to those of previously studied hairpin ribozymes. Additionally, bimolecular constructs cannot be used to experimentally compare cleavage rates between permuted forms because the ribozymes become equivalent in a bimolecular context (Figure 1B).

With the novel hairpin ribozyme sequences, the VS ribozyme is the only remaining self-cleaving ribozyme class with a small number of known members. Indeed, the one known VS ribozyme sequence does not permit any comparative analysis at all, among natural sequences. It seems reasonable to speculate that additional VS ribozymes will eventually be found.

The newly discovered hairpin ribozymes are often on the reverse strand to another self-cleaving ribozyme. We observed examples of the hammerhead, twister and hairpin ribozymes on this reverse strand. The fact that different classes of ribozymes occur on the reverse strand suggests that any structural class is acceptable—and therefore that there might be novel self-cleaving ribozymes within the hairpin-containing contigs that lack a recognized ribozyme on the reverse strand. We are using this strategy to find additional self-cleaving ribozymes (I. Eckert, C.E. Weinberg, Z. Weinberg, in preparation). In previous work, we found genomic locations where hammerhead and twister ribozymes often occurred, and were apparently interchangeable. A search of these regions revealed the hatchet, pistol and twister-sister ribozyme classes (80).

We are also exploring experimental methods aimed at discovering natural self-cleaving ribozymes in different organisms and environments. In our strategy, the unique RNA ends generated by the self-cleaving reaction, namely a 2′, 3′ cyclic phosphate on the 5′ ribozyme fragment and a 5′ hydroxyl on the 3′ fragment, are exploited in specific adapter ligation methods for RNA-seq (V.J. Olzog, C. Gärtner, P.F. Stadler, J. Fallmann, C.E. Weinberg, submitted).

The new hairpin ribozymes, especially those found in transcriptomic contigs (Supplementary Table S6), provide a starting point to study the biology of the RNAs that use these ribozymes. Discovery of new types of viroids or RNA satellites is of potential economic importance. Viroids cause diseases in several vegetable, fruit and field crops with severe effects on crop quality and yield (74). RNA satellites are of considerable interest because they modulate the severity of disease symptoms caused by their helper virus. Such modulation ranges from complete suppression of the virus to severe aggravation of symptoms, including death of the host (19). It is also possible that the hairpin-containing RNAs are not viroids or satellites, for example, they might be viruses. Whatever their nature, the range in size of the RNAs containing hairpin ribozymes is striking. We are not aware of a lineage of virus, viroid or satellite whose genome sizes range from a few hundred to several thousand nucleotides. Therefore, the different size ranges we observed (Figure 5,6A) might correspond to different types of organisms. However, given that we observe these inferred RNA organisms in metatranscriptomes from similar environments (Supplementary Text), the RNAs from different sizes might relate to each other or play similar roles.

Cultivation-independent methods have revealed that a significant amount of sequence space in biology is unknown to science. Multiple studies have thus exploited high-throughput RNA sequences in order to more comprehensively understand RNA viruses (81–83). These studies have generally been focused on detecting genes that are characteristic of RNA viruses, especially viral reverse transcriptases. They have been remarkably successful at finding new viral lineages. However, none of these studies have identified the hairpin-containing contigs, even though at least one study (82) interrogated an environment that we observed to contain hairpin ribozymes. This oversight is, of course, a result of the need to target a protein-coding gene (e.g. reverse transcriptase) in order to identify viral contigs. Hairpin-containing contigs do not encode any known protein domain, so such a strategy cannot find them. Thus, our results support the suspicion that there is a significant universe of sequences that remain unknown to science, despite highly sophisticated, modern high-throughput sequencing technologies. In our case, these sequences probably correspond to viroids, RNA satellites or viruses, but they also show that there are other types of sequences that cannot be usefully classified by existing methods. Such sequences are effectively out of the reach of science, at present.

## DATA AVAILABILITY

All relevant data are available in supplementary materials. Key alignments from the Z. Weinberg group from papers accepted for publication are also available in the ZWD repository (https://bitbucket.org/zashaw/zashaweinbergdata/src/master).

## Supplementary Material

gkab454_Supplemental_Files
