# Supplementary material for: Identification of over 200-fold more hairpin ribozymes than previously known in diverse circular RNAs
Source: Nucleic Acids Res. 2021 Jun 7;49(11):6375–88. doi: 10.1093/nar/gkab454 (PMC8216279; doi:10.1093/nar/gkab454)
Supplement: gkab454_Supplemental_Files [file gkab454_supplemental_files.zip › 2021-05-10-hairpin-deluge--supplementary.docx]

**Identification of over 200-fold more hairpin ribozymes than previously known in diverse circular RNAs**

**Christina E. Weinberg^1^, V. Janett Olzog^1^, Iris Eckert^2^, Zasha Weinberg^2,*^**

^1^ Institute for Biochemistry, Leipzig University, Brüderstraße 34, 04103 Leipzig, Germany,

^2^ Bioinformatics Group, Department of Computer Science and Interdisciplinary Centre for Bioinformatics, Leipzig University, Härtelstraße 16–18, 04107 Leipzig, Germany

* To whom correspondence should be addressed: Tel: +49 341 97 16657; Email: zasha@bioinf.uni-leipzig.de

Supplementary File captions, Text, Tables and Figures

## Supplementary File captions

**Supplementary File 1**. Search patterns used to search for permuted forms of ribozymes. Patterns work with either DARN! or RNAMotif. See Supplementary Table 10 for details.

**Supplementary File 2**. Machine-readable alignments of hairpin ribozymes. This .zip archive includes the alignments described in Supplementary Table 2 in Stockholm format.

**Supplementary File 3.** Printable alignments of hairpin ribozymes. This PDF file contains the alignments described in Supplementary Table 2.

**Supplementary File 4**. Alignments of conserved proteins in hairpin contigs. This .zip archive includes protein alignments in computer-readable Stockholm format.

## Supplementary Text

### Hairpin-ribozyme containing contigs have no repeats or self-similarity other than end repeats.

We observed end repeats in many of the contigs containing hairpin ribozymes, but there might be other kinds of repetitive patterns within these sequences. We therefore compared all contigs that have a hairpin ribozyme and end repeats to themselves using BLAST with an E-value threshold of 0.1, to find repeated sequences other than end repeats. We found only one other example of self-similarity: a perfect match of 28 nucleotides in a single contig. Thus, the contigs have essentially no type of self-similarity other than end repeats. As mentioned in the main text, we believe that the end repeats are technical artifacts caused by how the sequence assembly process functions with circular RNA.

### Analysis of coding regions in contigs

We predicted which contigs likely encode proteins using the RNAcode software (1). RNAcode uses probabilistic models in order to determine if a multiple-sequence alignment likely contains a coding region. Based on this analysis, we see clear evidence of coding for most long (>4500 nucleotides) contigs and for a small minority of medium-sized (800-1800 nucleotides) contigs (Figure 5B). We see no support for coding in small (<800 nucleotides) contigs. As RNAcode is a comparative method, it might work better or worse depending on properties of its input alignment. Properties that are especially likely to influence results are the average percent identity of the sequences and the number of sequences (1). Based on these statistical features of the alignments in small-, medium- and large-sized contigs, we would have expected to find coding signals in small contigs, if they were to encode proteins (Supplementary Figure 12). These results suggest that the small contigs might not code for proteins. Thus, contigs with hairpin ribozymes have a dramatic size range, and some seem to code for proteins, while others likely do not.

The detailed methods in our application of RNAcode follow. We used RNAcode (1) version 0.3, which detects statistically significant evidence for evolutionary conservation of coding sequences. To generate alignments, we compared sequences using hmmsearch from HMMER version 3.2.1 (http://hmmer.org) with an E-value cutoff of 0.001. The queries to hmmsearch were composed by sliding a window of 200 nucleotides along each complete contig, advancing the windows 100 nucleotides at a time (i.e., nucleotides 1-200 in the first window, then 101-300, 201-400, etc.). For each window, all contigs were searched, and hmmsearch’s alignment was used as input to RNAcode. We initially applied an E-value threshold of 0.0001 to the RNAcode results, and the poorest RNAcode E-value observed that was better than this threshold was 10^-7^. Since there were 6,303 query windows, the worst alignment had an effective E-value of 10^-7^×6303 ≈ 0.0006. For each alignment with an E-value better than 0.0001, we extracted the corresponding protein sequence within the query window’s nucleotides, merging any overlapping or contiguous regions in the same protein. The resulting alignments, each containing one protein sequence, were then used with hmmsearch as queries of all ORFs within the contigs, with an E-value threshold of 10^-11^, to avoid low quality alignments. We then eliminated pairs of alignments that have any protein subsequence in common, retaining the alignment with fewer sequences, since it is likely to be of higher quality. Sequences were removed from the resulting alignments if they were likely to be truncated, i.e., if they had more than 3 gaps at the N- or C-terminus, or if their gene was within 50 nucleotides of the end of the containing contig. This procedure resulted in 23 alignments (Supplementary File 4).

### Environments enriched for hairpin ribozymes

For the most part, the environments in which contigs are enriched are essentially the same in different contig size ranges (Supplementary Table 9). Despite similar overall trends, large (>4000 nucleotides) contigs occur less frequently than smaller contigs. This apparent rarity might reflect the fact that assembling a larger contig requires more reads than a smaller contig (so we are less likely to find fully assembled larger contigs), or it could reflect actual biological differences. Despite their overall rarity, large contigs showed apparent enrichment in hydrated desert soil (2), in contrast to smaller contigs. However, since the relevant metatranscriptome contained relatively few sequences, it is not clear how statistically significant this result is.

### Replicating circular ssRNAs

Sequenced viroids range from 254 to 434 nucleotides in size and generally do not code for proteins. Plant RNA satellites containing known self-cleaving ribozymes range from 220 to 457 nucleotides, and also typically lack coding regions. The HDV ribozyme is found within the hepatitis delta virus (HDV), a satellite of the hepatitis B virus that infects humans. The HDV genome is roughly 1,700 nucleotides in length and includes a protein-coding gene (3). The Varkud satellite (VS) is found in some strains of the fungus *Neurospora*, and contains the VS ribozyme. The Varkud satellite is a circular ssRNA of 881 nucleotides that replicates via transcription from DNA, followed by reverse transcription into DNA (4). Its replication is dependent on genes provided by the V plasmid, and the Varkud satellite itself is not known to contain any protein-coding genes. Finally, some Chuviruses are circular (5). Their ssRNA genomes are several thousand nucleotides in length, and contain reverse transcriptase genes.

### Contigs with hairpins are not similar to known circular ssRNA genomes

To determine if the contigs containing novel hairpin ribozyme sequences were similar to previously characterized organisms, we compared these contigs to the genomes of organisms with circular ssRNA genomes. Because of conserved nucleotides in hairpin and hammerhead ribozymes, such ribozymes sometimes cause matches between two sequences, even if the non-ribozyme parts of the sequences are not similar to one another. Therefore, we removed regions of the hairpin-containing contigs that corresponded to either predicted hairpin or hammerhead ribozymes. Then, we compared the remaining parts of the sequences to circular ssRNA genomic sequences in RefSeq (6) version 98 (differing from the version used for other analysis in this work). All such genomes in RefSeq were in the ‘viral’ division of the database. The comparison was performed using NCBI’s blastn program (7) with the flag -word_size 7 and an E-value threshold of 0.001. We found no matches with these parameters.

## Supplementary Tables

**Supplementary Table 1**. Primers and oligonucleotides used in this study. Text like “Ga0247519_104852/800-730” refers to a part of a metatranscriptomic contig. In this case, Ga0247519_104852 is the sequence accession within the IMG/M metatranscriptome database (8), and the region has its 5′ end at nucleotide number 800 and its 3′ end at nucleotide 730. Because 800 is larger than 730, this region is located on the reverse-complement strand.

| Name | Sequence (5′ to 3′) | Purpose |
| --- | --- | --- |
| CEW153 | TAATACGACTCACTATAGGTTGACACGCATATGTGCGGTCCGGCCCATCCGGGTCTGG | Forward oligonucleotide (oligo) to create an *in vitro* transcription template of hairpin candidate HPR 2 from Ga0247519_104852/800-730 by overlap extension with a partly complementary reverse oligo. This forward oligo is compatible with the wild-type (wt) and M1 reverse oligo and it contains a T7 promotor followed by two G nucleotides that are part of the natural sequence (GG). The candidate sequence was extended by 15 naturally occurring nucleotides to increase the length of the 5′ cleavage fragment. |
| CEW154 | GCCAGTTAATATGCCAACTTTCGTTGTGTTTCCCTGGCGTGCTTCCCAGACCCGGATGGG | Reverse oligo to create an *in vitro* transcription template of hairpin candidate HPR 2 from Ga0247519_104852/800-730 by overlap extension with a partly complementary forward oligo CEW153. This reverse oligo is compatible with the wt. |
| CEW155 | GCCAGTTAATATGCCAACTTTCGTTGTGTTTCCCTGGCGTGCTTGCCAGACCCGGATGGG | Reverse oligo to create an *in vitro* transcription template of hairpin candidate HPR 2 mutant M1 from Ga0247519_104852/800-730 by overlap extension with a partly complementary forward oligo CEW153. |
| CEW156 | TAATACGACTCACTATAgGAACAGACACTTCAGGTCGGTCCGGTTACATGCCGGGAAGAC | Forward oligo to create an *in vitro* transcription template of hairpin candidate HPR 3 from Ga0179955_1048341/123-51 by overlap extension with a partly complementary reverse oligo CEW157. This forward oligo is compatible with the wt reverse oligo and it contains a T7 promotor followed by a G-nucleotide that is not part of the natural sequence (g). The candidate sequence was extended by 13 naturally occurring nucleotides to increase the length of the 5′ cleavage fragment. |
| CEW157 | GCCAGTTAATGTACCACGGTTCCAAATACGTGTGTTTCCCTGGCGGTCTTCCCGGCATGT | Reverse oligo to create an *in vitro* transcription template of hairpin candidate HPR 3 from Ga0179955_1048341/123-51 by overlap extension with a partly complementary forward oligo CEW156. This reverse oligo is compatible with the wt. |
| CEW158 | TAATACGACTCACTATAgGAACAGACACTTCAGGTCGGTCCGGTTACATGCCGGCAAGAC | Forward oligo to create an *in vitro* transcription template of hairpin candidate HPR 3 M1 from Ga0179955_1048341/123-51 by overlap extension with a partly complementary M1 reverse oligo CEW159. |
| CEW159 | GCCAGTTAATGTACCACGGTTCCAAATACGTGTGTTTCCCTGGCGGTCTTGCCGGCATGT | Reverse oligo to create an *in vitro* transcription template of hairpin candidate HPR 3 M1 from Ga0179955_1048341/123-51 by overlap extension with a partly complementary M1 forward oligo CEW158. |
| CEW160 | TAATACGACTCACTATAggACATCAAGCGGTCCGTTATGTCCATCAGACATGCGGGAAGC | Forward oligo to create an *in vitro* transcription template of hairpin candidate HPR 1 from Ga0247518_108423/758-837 by overlap extension with a partly complementary reverse oligo CEW161. This forward oligo is compatible with the wt reverse oligo and it contains a T7 promotor followed by two G nucleotides that are not part of the natural sequence (gg). The candidate sequence was extended by 5 naturally occurring nucleotides to increase the length of the 5′ cleavage fragment. |
| CEW161 | GCCAGTTAATTTCCCAATACAAAGGTATTGTGTTTCCCTGGCAAGCTTCCCGCATGTCTG | Reverse oligo to create an *in vitro* transcription template of hairpin candidate HPR 1 from Ga0247518_108423/758-837 by overlap extension with a partly complementary forward oligo CEW160. This reverse oligo is compatible with the wt. |
| CEW162 | TAATACGACTCACTATAggACATCAAGCGGTCCGTTATGTCCATCAGACATGCGGCAAGC | Forward oligo to create an *in vitro* transcription template of hairpin candidate HPR 1 RB1 from Ga0247518_108423/758-837 by overlap extension with a partly complementary M1 reverse oligo CEW163. |
| CEW163 | GCCAGTTAATTTCCCAATACAAAGGTATTGTGTTTCCCTGGCAAGCTTGCCGCATGTCTG | Reverse oligo to create an *in vitro* transcription template of hairpin candidate HPR 1 M1 from Ga0247518_108423/758-837 by overlap extension with a partly complementary M1 forward oligo CEW162. |
| CEW336 | CATCCAGACGTCGTCGAATCGC | Used for reverse transcription of metatranscriptomic RNA followed by PCR amplification with CEW340 and CEW341 to create PCR product of 564 bp that corresponds to a sequence that starts at position 121, runs through position 1 and ends at position 72 of sequence contig Ga0247519_111615 (Figure 4B, “P3”). |
| CEW340 | AACCAGTTGATTGCTGGAATGACTG | Used for reverse transcription of metatranscriptomic RNA followed by PCR with CEW346 and CEW341 to create PCR product of 533 bp that corresponds to a sequence that starts at position 152, runs through position 1 and ends at position 72 of contig Ga0247519_111615 (Figure 4A, “P1”). |
| CEW341 | GACCTCGTTCTGGAGCACCTTAC | Primer for nested PCRs of contig Ga0247519_111615 together with CEW340 or CEW346 |
| CEW343 | CTTCGGCTATGAGCGCCTAAG | Used for reverse transcription of metatranscriptomic RNA followed by PCR with CEW345 and CEW347 to create PCR product of 488 bp that corresponds to a sequence that starts at position 578, runs through position 1 and ends at position 453 of contig Ga0247519_111615 (Figure 4B, “P4”). |
| CEW344 | AAGTGTGCGCTGAAAAGTGC | Used for reverse transcription of metatranscriptomic RNA followed by PCR with CEW347 and CEW343 to create PCR product of 519 bp that corresponds to a sequence that starts at position 578, runs through position 1 and ends at position 484 of contig Ga0247519_111615 (Figure 4A, “P2”). |
| CEW345 | GGGTTGGTCCTGGTACAGC | Primer for nested PCR of contig Ga0247519_111615 together with CEW347. |
| CEW346 | GCAGCTCAACAGTACAGCGTAG | Primer for nested PCR of contig Ga0247519_111615 together with CEW341. |
| CEW347 | GGACTATGGGTTGACCAGACCC | Primer for nested PCR of contig Ga0247519_111615 together with CEW345. |
| CEW444 | TTAATACGACTCACTATAgGCAATTTGCAGTCATTCCAGCAATCAACTGGTTTAGAAGCA | Forward oligo to create PCR template of HP-contig from Ga0247519_111615/153-40 by overlap extension with a partly complementary reverse oligo CEW445. Forward primer with T7 promoter (CEW47) and reverse primer (CEW446) are needed to create full transcription template. |
| CEW445 | TCGCGCAAGCGATTCGACGACGTCTGGATGTTTCACTGACTTTGCTTCTAAACCAGTTGA | Reverse oligo to create PCR template of HP-contig from Ga0247519_111615/153-40 by overlap extension with a partly complementary forward oligo CEW444. Forward primer with T7 promoter (CEW47) and reverse primer (CEW446) are needed to create full transcription template. |
| CEW446 | TTAATGTCAGGTAAGGTGCTCCAGAACGAGGTCGCGCAAGCG | Reverse primer for PCR to create HP-contig from Ga0247519_111615/153-40. Forward primer with T7 promoter (CEW47) is used for this PCR to create full transcription template. |
| CEW447 | ggACGGAAGTCTGTCCTGATGCTGGTTGCTAACCAGCATACAGAGAAGACACCAGAGG | Forward oligo to create PCR template of HP-Ev7 from Ga0257136_1059838/554-424 by overlap extension with a partly complementary reverse oligo CEW448. Forward primer with T7 promoter (CEW450) and reverse primer (CEW449) are needed to create full transcription template. |
| CEW448 | GTGAGCTTCGCAGCTCACTCTTTACTAGCTTGGAGAGTGTTTCCTCTGGTGTCTTCT | Reverse oligo to create PCR template of HP-Ev7 from Ga0257136_1059838/554-424 by overlap extension with a partly complementary forward oligo CEW447. Forward primer with T7 promoter (CEW450) and reverse primer (CEW449) are needed to create full transcription template. |
| CEW449 | CCTTTCCAGGTTAATTTTCCCCTCCTCGCTATAGTGAGCTTCGCAGCTC | Reverse oligo to create PCR template of HP-Ev7 from Ga0257136_1059838/554-424 by overlap extension with a partly complementary forward oligo CEW447. Forward primer with T7 promoter (CEW450) and reverse primer (CEW449) are needed to create full transcription template. |
| CEW450 | GAAATTAATACGACTCACTATAggACGGAAGTCTGTCCT | Forward primer with T7 promoter for PCR to create HP-Ev7 from Ga0257136_1059838/554-424. Reverse primer CEW449 is used for this PCR to create full transcription template. |
| CEW451 | GAAATTAATACGACTCACTATAggCCCCATCTCTGTCTCTGATTCGGGATTTGCTAAATC | Forward oligo to create fragment 1 of HP-Ev6.6_Ga0247515_107373/543-736 by overlap extension with a partly complementary reverse oligo CEW452. Has to be combined with fragment 2 before final PCR. |
| CEW452 | AGGTAGAAAGGCCTGAGCAACGACACACTGAACCAATGCCTTGGGATTTAGCAAATCCCG | Reverse oligo to create fragment 1 of HP-Ev6.6_Ga0247515_107373/543-736 by overlap extension with a partly complementary forward oligo CEW451. Has to be combined with fragment 2 before final PCR. |
| CEW453 | CAGGCCTTTCTACCTTAGTGGTGTTTGATACCTGAGTGCTTCTTGCACTTGATCCACCCG | Forward oligo to create fragment 2 of HP-Ev6.6_Ga0247515_107373/543-736 by overlap extension with a partly complementary reverse oligo CEW452. Has to be combined with fragment 1 before final PCR. |
| CEW454 | AAGCCAATTCCTAAGTGTGTTTCCCTGGAAGATCTTCTTCTGATCCGGGTGGATCAAGTG | Reverse oligo to create fragment 2 of HP-Ev6.6_Ga0247515_107373/543-736 by overlap extension with a partly complementary forward oligo CEW451. Has to be combined with fragment 1 before final PCR. |
| CEW455 | CAATGTCCAGTTAATGTGCCACTTAAGCCAATTCCTAAGTGT | Reverse primer for PCR to create HP-Ev6.6_Ga0247515_107373/543-736. Forward primer with T7 promoter (CEW47) is used for this PCR to create full transcription template. Take care to assemble PCR template from fragment 1 and 2 first (CEW451-CEW454). |
| CEW456 | TTAATACGACTCACTATAgGCAATTTGCAGTCATTCCAGCAATCAACTGGTTTACAAGCA | Forward oligo to create PCR template of HP-contig M1 (cleavage deficient variant) from Ga0247519_111615/153-40 by overlap extension with a partly complementary reverse oligo CEW457. Forward primer with T7 promoter (CEW47) and reverse primer (CEW446) are needed to create full transcription template. |
| CEW457 | TCGCGCAAGCGATTCGACGACGTCTGGATGTTTCACTGACTTTGCTTGTAAACCAGTTGA | Reverse oligo to create PCR template of HP-contig M1 (cleavage deficient variant) from Ga0247519_111615/153-40 by overlap extension with a partly complementary forward oligo CEW456. Forward primer with T7 promoter (CEW47) and reverse primer (CEW446) are needed to create full transcription template. |
| CEW458 | TTAATACGACTCACTATAggAGAGTGGACTGGACCAAGGCTCATTCAGGTTGGATCTTGC | Forward oligo to create PCR template of HP-Ev5.6 from Ga0247519_108765/575-698 by overlap extension with a partly complementary reverse oligo CEW459. Forward primer with T7 promoter (CEW47) and reverse primer (CEW460) are needed to create full transcription template. |
| CEW459 | TGTTTTCCTGTCTGACTTCGCCATGACTCCAGGCAACCCTAGGATGCAAGATCCAACCTG | Reverse oligo to create PCR template of HP-Ev5.6 from Ga0247519_108765/575-698 by overlap extension with a partly complementary forward oligo CEW458. Forward primer with T7 promoter (CEW47) and reverse primer (CEW460) are needed to create full transcription template. |
| CEW460 | ACATTGACAGTTAATATGCCGACTCTCGGGCTTGAGCCGTGTTTTCCTGTCTGACTT | Reverse primer for PCR to create HP-Ev5.6 from Ga0247519_108765/575-698. Forward primer with T7 promoter (CEW47) is used for this PCR to create full transcription template. |
| CEW461 | TAATACGACTCACTATAggACAAGCTTCTGTCCCCATCACTATGTTCTGCATCTGCTTTC | Forward oligo to create fragment 1 of HP-Ev5_Ga0247512_104440/906-1087 by overlap extension with a partly complementary reverse oligo CEW462. Has to be combined with fragment 2 before final PCR. |
| CEW462 | TCTCTTCCTCTCCCACCACAGTGGCAGACGCCTGTTTTGCATTGAAAGCAGATGCAGAAC | Reverse oligo to create fragment 1 of HP-Ev5_Ga0247512_104440/906-1087 by overlap extension with a partly complementary forward oligo CEW461. Has to be combined with fragment 2 before final PCR. |
| CEW463 | GTGGGAGAGGAAGAGACCAGGGAAACATGCTGTAGTCTAGTCTGGGTTTACGGGTATGTA | Forward oligo to create fragment 2 of HP-Ev5_Ga0247512_104440/906-1087 by overlap extension with a partly complementary reverse oligo CEW464. Has to be combined with fragment 1 before final PCR. |
| CEW464 | GCTACCGAAATCTGGTTAGGTCTATGGCTGTAATGGCTACATACCCGTAAACCC | Reverse oligo to create fragment 2 of HP-Ev5_Ga0247512_104440/906-1087 by overlap extension with a partly complementary forward oligo CEW463. Has to be combined with fragment 1 before final PCR. |
| CEW465 | AAGATGCCAGTTAATGTGCTACCGAAATCTGG | Reverse primer for PCR to create HP-Ev5_Ga0247512_104440/906-1087. Forward primer with T7 promoter (CEW47) is used for this PCR to create full transcription template. Take care to assemble PCR template from fragment 1 and 2 first (CEW461-CEW464). |
| CEW466 | ATTAATACGACTCACTATAgGATATCTGCGGTCGGGATGTTGACTTATGTCACTCCTG | Forward oligo to create PCR template of HP-Ev4.8 from Ga0247519_103291/1376-1465 by overlap extension with a partly complementary reverse oligo CEW467. Forward primer with T7 promoter (CEW47) and reverse primer (CEW468) are needed to create full transcription template. |
| CEW467 | CATCGATGGTGTTTCCCCACACTGCTTCCAGGAGTGACATAAGTC | Reverse oligo to create PCR template of HP-Ev4.8 from Ga0247519_103291/1376-1465 by overlap extension with a partly complementary forward oligo CEW466. Forward primer with T7 promoter (CEW47) and reverse primer (CEW468) are needed to create full transcription template. |
| CEW468 | CGCTTTGTGGTTAATGTGCCCATCCATCGATGGTGTTTCC | Reverse primer for PCR to create HP-Ev4.8 from Ga0247519_103291/1376-1465. Forward primer with T7 promoter (CEW47) is used for this PCR to create full transcription template. |
| CEW469 | AAGCCAATTCCTAAGTGTGTTTCCCTGGAAGATCTTGTTCTGATCCGGGTGGATCAAGTG | Reverse oligo to create fragment 2 of HP-Ev6.6_M1_Ga0247515_107373/543-736 by overlap extension with a partly complementary forward oligo CEW451. Has to be combined with fragment 1 before final PCR. |
| CEW470 | CATCGATGGTGTTTCCCCACACTGCTTGCAGGAGTGACATAAGTC | Reverse oligo to create PCR template of HP-Ev4.8_M1 from Ga0247519_103291/1376-1465 by overlap extension with a partly complementary forward oligo CEW466. Forward primer with T7 promoter (CEW47) and reverse primer (CEW468) are needed to create full transcription template. |
| CEW471 | CACTATAggCAATATCTCTGTCTGCCTTCGGTAAGCGATTGACGATCGCACTAATGGCAA | Forward oligo to create PCR template of HP-L1 from Ga0310119_125223/188-324 by overlap extension with a partly complementary reverse oligo CEW472. Forward primer with T7 promoter (CEW473) and reverse primer (CEW474) are needed to create full transcription template. |
| CEW472 | TAGCCATCATCGGATGATTTCGTGTTTCTCTCACAATCTCTTCTTGCCATTAGTGCGATC | Reverse oligo to create PCR template of HP-L1 from Ga0310119_125223/188-324by overlap extension with a partly complementary forward oligo CEW471. Forward primer with T7 promoter (CEW473) and reverse primer (CEW474) are needed to create full transcription template. |
| CEW473 | GAAATTAATACGACTCACTATAggCAATATCTCTG | Forward primer with T7 promoter for PCR to create HP-L1 from Ga0310119_125223/188-324. Reverse primer CEW474 is used for this PCR to create full transcription template. |
| CEW474 | GATTGGTGAGGTAATGTGCCGAAATTCACTTTCTGAAGGTGACTAGCCATCATCGGATG | Reverse primer for PCR to create HP-L1 from Ga0310119_125223/188-324. Forward primer with T7 promoter (CEW473) is used for this PCR to create full transcription template. |
| CEW475 | TAATACGACTCACTATAgGCACACATCTGTCCTGAGTTATAAATTCAGAGAAGATGCAAA | Forward oligo to create PCR template of HP-L2 from Ga0138292_1031932/9-105 by overlap extension with a partly complementary reverse oligo CEW476. Forward primer with T7 promoter (CEW47) and reverse primer (CEW477) are needed to create full transcription template. |
| CEW476 | AATGTGCCGTATCTGAGCCTAGAGTTCGATACGTGTTTCCCGCTTTGCATCTTCTCTGAA | Reverse oligo to create PCR template of HP-L2 from Ga0138292_1031932/9-105 by overlap extension with a partly complementary forward oligo CEW475. Forward primer with T7 promoter (CEW47) and reverse primer (CEW477) are needed to create full transcription template. |
| CEW477 | GTTAAAAGCGGTAATGTGCCGTATCTG | Reverse primer for PCR to create HP-L2 from Ga0138292_1031932/9-105. Forward primer with T7 promoter (CEW47) is used for this PCR to create full transcription template. |
| CEW478 | TAGCCATCATCGGATGATTTCGTGTTTCTCTCACAATCTCTTGTTGCCATTAGTGCGATC | Reverse oligo to create PCR template of HP-L1 M1 from Ga0310119_125223/188-324by overlap extension with a partly complementary forward oligo CEW471. Forward primer with T7 promoter (CEW473) and reverse primer (CEW474) are needed to create full transcription template. |
| CEW479 | GAAATTAATACGACTCACTATAggCTCAACTGCTGTCCAGCGAGTTTCAACCCTCGCTGA | Forward oligo to create PCR template of HP-G21 from Ga0138276_1121602/324-415 by overlap extension with a partly complementary reverse oligo CEW480. Forward primer with T7 promoter (CEW47) and reverse primer (CEW481) are needed to create full transcription template. |
| CEW480 | AATATGCTGGCCAGTTAATCGGCCATGTTTTGCCTGCCTGCTTCTCAGCGAGGGTTGAAA | Reverse oligo to create PCR template of HP-G21 from Ga0138276_1121602/324-415 by overlap extension with a partly complementary forward oligo CEW479. Forward primer with T7 promoter (CEW47) and reverse primer (CEW481) are needed to create full transcription template. |
| CEW481 | AGGGTGCAGGGTAATATGCTGGCCAGTTAATC | Reverse primer for PCR to create HP-G21 from Ga0138276_1121602/324-415. Forward primer with T7 promoter (CEW47) is used for this PCR to create full transcription template. |
| CEW482 | AATATGCTGGCCAGTTAATCGGCCATGTTTTGCCTGCCTGCTTGTCAGCGAGGGTTGAAA | Reverse oligo to create PCR template of HP-G21 M1 variant from Ga0138276_1121602/324-415 by overlap extension with a partly complementary forward oligo CEW479. Forward primer with T7 promoter (CEW47) and reverse primer (CEW481) are needed to create full transcription template. |
| CEW483 | AATACGACTCACTATAggAAAACCCACTGTCCTAAGACTAATGTCTTAGGGAAATGGGTG | Forward oligo to create PCR template of HP-G11 from Ga0307919_1034281/532-446 by overlap extension with a partly complementary reverse oligo CEW484. Forward primer with T7 promoter (CEW47) and reverse primer (CEW485) are needed to create full transcription template. |
| CEW484 | GTTGTGAGTTAATGTACCTTCATATATAATGAAGTGTTTCCCTCACCCATTTCCCTAAGA | Reverse oligo to create PCR template of HP-G11 from Ga0307919_1034281/532-446 by overlap extension with a partly complementary forward oligo CEW483. Forward primer with T7 promoter (CEW47) and reverse primer (CEW484) are needed to create full transcription template. |
| CEW485 | TAGTTGTGAGTTAATGTACCT | Reverse primer for PCR to create HP-G11 from Ga0307919_1034281/532-446. Forward primer with T7 promoter (CEW47) is used for this PCR to create full transcription template. |
| CEW486 | GAAATTAATACGACTCACTATAggCGACCGGTCAGTCCCTTTCTTTTGAAAGGAGAGGAC | Forward oligo to create PCR template of HP-A10 from Ga0184572_113323/225-305 by overlap extension with a partly complementary reverse oligo CEW487. |
| CEW487 | TCCAGTAGGGGTAATGTGCCGTGTTATACACGTGTTTCACCCTAGGTCCTCTCCTTTCAA | Reverse oligo to create PCR template of HP-A10 from Ga0184572_113323/225-305 by overlap extension with a partly complementary forward oligo CEW486. |

**Supplementary Table 2**. RNA alignments produced in this study. The alignments are available in computer-readable form in Supplementary File 2 and as a printable PDF in Supplementary File 3. “Name”: the name given to the alignment, which is used in other supplementary data. “Description”: a brief description. “E-value threshold”: the E-value threshold used in searches. “Searched database”: the sequence database used. “First database”: the original collection of RefSeq, metagenomic and metatranscriptomic sequences. (The E-value thresholds in table rows related to this database are those described in Supplementary Figure 4C,D.) “Spruced-up database”: the first database augmented with additional spruce-tree-associated metatranscriptomic sequences that we downloaded. (The E-value thresholds related to this database are those of Supplementary Figure 4F.) We downloaded these additional sequences because we noticed that the hairpin ribozymes tended to be enriched in spruce-associated metatranscriptomes. “Number of sequences”: the number of sequences in the alignment. “Unique”: not counting exact duplicates, “Total”: including duplicates. “N/D”: not determined. Note: the “extra-spruce-stringent” alignment contains more predicted hairpin ribozymes than the “extra-spruce-initial” alignments, even though the E-values in its iterative construction were more stringent. We infer that the extra diversity of sequences in the initial alignment make this alignment better for detecting diverse sequences in the spruced-up database, but worse at detecting relatively typical sequences. If typical sequences are more common than diverse sequences, then more sequences will be found in the extra-spruce-stringent alignment.

| **Name** | **Description** | **E-value threshold** | **Searched database** | **Number of sequences** | |
| --- | --- | --- | --- | --- | --- |
|  |  |  |  | **Unique** | **Total** |
| initial | Our initial candidate, based on the first database. | 10 | First database | 84 | N/D |
| touched-up-initial | The “initial” alignment, with some manual adjustments of long hairpins that were not well aligned in Infernal’s automatic alignment. This alignment was not used for any subsequent searches or analysis. | Not applicable | Not applicable | 84 | N/D |
| extra-spruce-initial | The “initial” alignment searched against additional sequences. | 0.1 | Spruced-up database | 941 | 1700 |
| stringent | The same sequence match as in the “initial” alignment, but we used a much more stringent E-value threshold, to be more conservative with false positives. | 0.01 | First database | 59 | N/D |
| extra-spruce-stringent | The “stringent” alignment searched against additional sequences. | 0.1 | Spruced-up database | 1074 | 2902 |
| second-candidate | We found another sequence that led to convincing covariation. Since these sequences were very long, we did not pursue them further. Because some sequence contigs are still much longer than the aligned sequences, we believe that the alignment corresponds to the biologically most relevant permutation, as we argued in Discussion. Due to the low number of sequences, we were unable to completely determine the full secondary structure. We analyzed this candidate from the beginning with the spruced-up database. | 10 | Spruced-up database | 48 | 68 |
| TSA | Automated alignment of sequences present in the TSA database, using the “initial” alignment as a query. These results underlie Supplementary Table 6. | 0.1 | TSA (NCBI Web site) | N/D | 55 |
| Previously-published-four | This alignment summarizes what is known about the previously published four hairpin ribozymes. We used the consensus structure of the core region (i.e. the region depicted in Figure 1A) derived from previous publications. We used a partial secondary structure of the non-core region that has been proposed for the four previously known hairpin ribozymes (9). We did not attempt to infer further helices. | Not applicable | Previous publications | 4 | N/D |

**Supplementary Table 3**. Nucleotide deviations in natural sequences that we tested. This table provides details underlying Figure 2. “Deviations” refers to nucleotides in structurally important positions that were not previously tested in the context of natural sequences. “Nucleotide position”: refers to the positions defined in Figure 2. “Nucleotides/Lengths”: the specific nucleotide present at the given position (related to Figure 2B-E) or the lengths of helices and/or junctions (related to Figure 2F-G). “Tested molecule(s)”: the predicted natural hairpin ribozyme(s) that we tested related to this deviation. “S.Fig.”: Supplementary Figure. The depictions of hairpin ribozymes with poor E-values were altered manually to eliminate non-canonical base pairs or add Watson-Crick base pairs where possible. In this table, we depict the results of the automated alignments (related to Fig. 2F) or the automated base-pair procedure (related to Fig. 2G).

| **Nucleotide position** | **Nucleotide / Lengths** | **Tested molecule(s)** |
| --- | --- | --- |
| N-1 | G | HPR 1 (Fig. 1D), HPR 2 (S.Fig. 5), HPR 3 (S.Fig. 5), Ev 4.8 (S.Fig. 6) |
|  | U | Ev 7 (S.Fig. 6), Ev 6.6 (S.Fig. 6), Ev 5.6 (S.Fig. 6), Ev 5 (S.Fig. 6), G21🡪A (S.Fig. 9), G11🡪A (S.Fig. 9), L1 (S.Fig. 10), L2 (S.Fig. 10) |
| U+2 | G | Ev 5.6 (S.Fig. 6) |
| R7 | G | HPR 1 (Fig. 1D), HPR 2 (S.Fig. 5), HPR 3 (S.Fig. 5), Ev 4.8 (S.Fig. 6), G11🡪A (S.Fig. 9) |
| A10 | G | Ev 5 (S.Fig. 6), A10🡪G (S.Fig. 9) |
| G11 | A | G11🡪A (S.Fig. 9) |
| R20 | U | A10🡪G (S.Fig. 9), Contig (S.Fig. 11) |
| G21 | A | Ev 5.6 (S.Fig. 6), G21🡪A (S.Fig. 9) |
| Y37 | A | Ev 7 (S.Fig. 6) |
|  | C | HPR 2 (S.Fig. 5), Ev 6.6 (S.Fig. 6), Ev 5.6 (S.Fig. 6), Ev 5 (S.Fig. 6), Ev 4.8 (S.Fig. 6), A10🡪G (S.Fig. 9), G21🡪A (S.Fig. 9), L1 (S.Fig. 10), L2 (S.Fig. 10), Contig (S.Fig. 11) |
|  | G | HPR 1 (Fig. 1D) |
| Y39 | A | HPR 1 (Fig. 1D), Ev 7 (S.Fig. 6) |
|  | C | HPR 3 (S.Fig. 5), Ev 6.6 (S.Fig. 6), Ev 5 (S.Fig. 6), Ev 4.8 (S.Fig. 6), A10🡪G (S.Fig. 9), G11🡪A (S.Fig. 9), L1 (S.Fig. 10), L2 (S.Fig. 10), Contig (S.Fig. 11) |
| A40 | C | Contig (S.Fig. 11) |
| N44 | A | HPR 1 (Fig. 1D), HPR 2 (S.Fig. 5), HPR 3 (S.Fig. 5), Ev 6.6 (S.Fig. 6), Ev 5.6 (S.Fig. 6), Ev 5 (S.Fig. 6), Ev 4.8 (S.Fig. 6), G11🡪A (S.Fig. 9) |
| Junction (without realignment; related to Figure 2F) | 0nt | HPR 1 (Fig. 1D), HPR 2 (S.Fig. 5), HPR 3 (S.Fig. 5), Ev 7 (S.Fig. 6), Ev 4.8 (S.Fig. 6), A10🡪G (S.Fig. 9), G21🡪A (S.Fig. 9), G11🡪A (S.Fig. 9) |
|  | 1nt | Ev 6.6 (S.Fig. 6), Contig (S.Fig. 11) |
|  | 2nt | Ev 5 (S.Fig. 6), L1 (S.Fig. 10), L2 (S.Fig. 10) |
| Helix 2, junction, helix 3 (after extending helices; related to Figure 2G) | 4, 0, 5 | HPR 1 (Fig. 1D), HPR 2 (S.Fig. 5), HPR 3 (S.Fig. 5), Ev 4.8 (S.Fig. 6), A10🡪G (S.Fig. 9), G21🡪A (S.Fig. 9), G11🡪A (S.Fig. 9) |
|  | 5, 0, 5 | Contig (S.Fig. 11) |
|  | 4, 1, 6 | L2 (S.Fig. 10) |
|  | 5, 1, 5 | L1 (S.Fig. 10) |
|  | Has mismatch | Ev 7 (S.Fig. 6), Ev 6.6 (S.Fig. 6), Ev 5 (S.Fig. 6) |

**Supplementary Table 4.** Deviations in interacting nucleotides (other than those in the helices) that we tested. This table provides details underlying Supplementary Figure 8. “Nucleotide positions”: refers to the positions defined in Figure 2. “Nucleotides”: the specific nucleotides present at the given interacting positions. “Tested molecule(s)”: the predicted natural hairpin ribozyme(s) that we tested related to this deviation. “S.Fig.”: Supplementary Figure.

| **Nucleotide positions** | **Nucleotides** | **Tested molecule(s)** |
| --- | --- | --- |
| R7-C+3 | G-C | HPR 1 (Fig. 1D), HPR 2 (S.Fig. 5), HPR 3 (S.Fig. 5), Ev4.8 (S.Fig. 6), G11🡪A (S.Fig. 9) |
| G8-U+2 | G-G | Ev5.6 (S.Fig. 6) |
| A9-N-1 | A-G | HPR 1 (Fig. 1D), HPR 2 (S.Fig. 5), HPR 3 (S.Fig. 5), Ev4.8 (S.Fig. 6) |
|  | A-U | Ev7 (S.Fig. 6), Ev6.6 (S.Fig. 6), Ev5.6 (S.Fig. 6), Ev5 (S.Fig. 6), G21🡪A (S.Fig. 9), G11🡪A (S.Fig. 9), L1 (S.Fig. 10), L2 (S.Fig. 10) |
| G11-C-2 | A-C | G11🡪A (S.Fig. 9) |
| R20-N44 | G-A | HPR 1 (Fig. 1D), HPR 2 (S.Fig. 5), HPR 3 (S.Fig. 5), Ev7 (S.Fig. 6), Ev6.6 (S.Fig. 6), Ev5.6 (S.Fig. 6), Ev5 (S.Fig. 6), Ev4.8 (S.Fig. 6), G11🡪A (S.Fig. 9) |
|  | G-C | L2 (S.Fig. 10) |
|  | U-C | Contig (S.Fig. 11), A10🡪G (S.Fig. 9) |
| G21-A43 | A-A | Ev5.6 (S.Fig. 6), G21🡪A (S.Fig. 9) |
| A23-A40 | A-C | Contig (S.Fig. 11) |
| Ribose zipper:  A10, G11, A24, C25 | A, A, A, C | G11🡪A (S.Fig. 9) |
|  | G, G, A, C | Ev5 (S.Fig. 6), A10🡪G (S.Fig. 9) |

**Supplementary Table 5**. Comparable metagenomic (DNA) and metatranscriptomic (RNA) data from related environments that were searched for hairpin ribozymes. All data for these searches were downloaded from IMG/M (8). Searches used the cmsearch program of Infernal (10) with our “initial” hairpin alignment (Supplementary Table 2). Default settings of cmsearch were used, except that the E-value threshold was set to 1, which in our experience is somewhat permissive. Despite this E-value threshold, no hairpin ribozymes were predicted in metagenomes. Note: the results of these searches were based solely on the automated search at the given E-value threshold, and the database searched was smaller, since it was restricted to metagenomes and metatranscriptomes in the listed environments. Therefore, the results do not exactly match the numbers given in other searches and alignments. The table shows that the amount of metagenomic sequences for each environment is comparable to the amount of corresponding metatranscriptomic sequences. Additionally, there are hundreds of predicted hairpin ribozymes in each of the metatranscriptomes, but zero in the metagenomes. Therefore, these hairpin ribozymes are not present in DNA forms, or are present extremely rarely, in comparison to their greater abundance in RNA.

| **Description of environment** | **Metagenomes** | | | **Metatranscriptomes** | | |
| --- | --- | --- | --- | --- | --- | --- |
|  | **# predicted hairpin ribozymes** | **Megabases of sequence data** | **IMG/M accessions** | **# predicted hairpin ribozymes** | **Megabases of sequence data** | **IMG/M accessions** |
| Soil microbial communities from Bohemian Forest, Czech Republic | 0 | 962 | 3300023030, 3300024271, 3300022733, 3300023019, 3300022731, 3300028013, 3300028021, 3300028020, 3300028037, 3300028015 | 700 | 488 | 3300031866, 3300023677, 3300023544, 3300023537, 3300023542, 3300032119, 3300023662, 3300031829, 3300023541, 3300032028, 3300023656, 3300023551, 3300019165, 3300019184, 3300032121, 3300019194, 3300019185, 3300019186, 3300019158, 3300032072, 3300019164, 3300019178, 3300019187, 3300019189 |
| Spruce roots microbial communities from Bohemian Forest, Czech Republic | 0 | 979 | 3300023017, 3300023224, 3300024123, 3300028019, 3300022729, 3300033544, 3300033548, 3300033545, 3300033546, 3300033547 | 134 | 578 | 3300023664, 3300023680, 3300023559, 3300023563, 3300023666, 3300023688, 3300023684, 3300023553, 3300023668, 3300023558, 3300023690, 3300023682, 3300023560, 3300023564, 3300023562, 3300023689, 3300023561, 3300023556, 3300023686, 3300023557 |
| Spruce litter microbial communities from Bohemian Forest, Czech Republic | 0 | 390 | 3300022728, 3300022727, 3300024220, 3300024176, 3300023012 | 1,182 | 576 | 3300023678, 3300023669, 3300031809, 3300032027, 3300023533, 3300031874, 3300031827, 3300023675, 3300023673, 3300023654, 3300031817, 3300019160, 3300031868, 3300019180, 3300019174, 3300031891, 3300019159, 3300031955, 3300019173, 3300031871, 3300019156, 3300031808, 3300019155, 3300031870, 3300019168, 3300031869, 3300019170, 3300031826, 3300019167, 3300031956, 3300019171, 3300031872, 3300019157, 3300031791 |
| Spruce roots microbial communities from Maridalen Valley, Oslo, Norway | 0 | 655 | 3300033544, 3300033548, 3300033545, 3300033546, 3300033547 | 212 | 633 | 3300031633, 3300031615, 3300031634, 3300031666, 3300031614, 3300031632, 3300031690, 3300031678, 3300031636, 3300031667, 3300031686, 3300031664, 3300031592, 3300031591, 3300031635 |
| Soil microbial communities from Maridalen Valley, Oslo, Norway | 0 | 361 | 3300028013, 3300028021, 3300028020, 3300028037, 3300028015 | 639 | 1186 | 3300031042, 3300030982, 3300031044, 3300030815, 3300030759, 3300031017, 3300030885, 3300030874, 3300030814, 3300030940, 3300031040, 3300030862, 3300030832, 3300030873, 3300030813, 3300030913, 3300030884, 3300030978, 3300030805, 3300031041 |
| Dystrophic lake water microbial communities from Trout Bog Lake, Wisconsin, USA | 0 | 2235 | 3300013093, 3300013094 | 287 | 2259 | 3300012703, 3300016691, 3300012699, 3300016687, 3300016680, 3300016678, 3300016682, 3300016692, 3300013060, 3300012696, 3300012695, 3300012700, 3300012693, 3300012692, 3300016677, 3300012698, 3300012691, 3300012750, 3300016684, 3300012743, 3300012749, 3300012689, 3300016679, 3300012747, 3300013068, 3300012701, 3300012686, 3300012694 |
| Wetland microbial communities from Old Woman Creek Reserve in Ohio, USA | 0 | 15168 | 3300027890, 3300027871, 3300009111, 3300009131, 3300027877, 3300009179 | 56 | 213 | 3300010131, 3300010138 |

**Supplementary Table 6**. Hairpin ribozymes predicted in the Transcriptome Shotgun Assembly (TSA) Sequence Database. We downloaded nucleotide sequences from the TSA on May 29, 2020. We then searched them using the cmsearch program (10) using the “initial” hairpin ribozyme alignment (Supplementary Table 2) and default parameters for the cmsearch program, except that an E-value threshold of 0.1 was used. In our experience with various RNA alignments, most cmsearch predictions are biologically valid at this E-value threshold. The resulting alignment is described in Supplementary Table 2 and provided in Supplementary Files 2 and 3. “TSA prefix”: 4-letter code used to identify a particular sample. “Organism”: taken from the TSA entry. “Notes”: are based on the relevant paper. “PubMed ID”: identifies the paper related to the sample. These IDs can be entered into PubMed (https://pubmed.ncbi.nlm.nih.gov) to retrieve the paper. “# hairpins predicted”: number of hairpin ribozymes predicted by cmsearch. Identical sequences in the same strand of a given contig were counted only once. “Best (lowest) E-value”: the best E-value among the predicted hairpin ribozymes. Lower values imply a more significant (better) prediction. “Accessions containing predictions”: contig accessions with predicted ribozymes are listed. When a given accession contains more than one predicted ribozyme, the reason for this is given. “Accession of genome”: we attempted to find a genome of the relevant species, to search for DNA examples of hairpin ribozymes. “*” the genome was produced by the same group as the transcriptome. Blank cells indicate that we were unable to find a relevant genome. We searched genomes for hairpin ribozymes using the same parameters as for the TSA sequences, but found no matches, even at an E-value threshold of 1.

| **TSA prefix** | **Organism** | **Notes** | **PubMed ID** | **# hairpins predicted** | **Best (lowest) E-value** | **Accessions containing predictions** | **Accession of genome** |
| --- | --- | --- | --- | --- | --- | --- | --- |
| GAOU | *Trifolium pratense* (plant; red clover) | Leaves from plants grown in drought conditions were sampled. | 24912738 | 1 | 0.01 | GAOU01012890 | GCA_900292005.1 |
| GEHE | *Leucocoprinus* sp. HH-2015d (fungus) | Fungus has symbiotic relationship with ants, but was cultivated in pure culture. | 27436133 | 2 | 0.00028 | GEHE01004364, GEHE01005692 |  |
| GEHG | *Leucocoprinus* sp. HH-2015c (fungus) | Fungus has symbiotic relationship with ants, but was cultivated in pure culture. | 27436133 | 1 | 0.048 | GEHG01006370 |  |
| GELB | *Sparassis latifolia* minxiu NO.1 (fungus; cauliflower mushroom) | Fungus was grown in lab conditions. | 29682127 | 4 | 0.000044 | GELB01000625, GELB01009586 (3 hairpin ribozymes, in both strands. The predicted ribozymes located on the same strand are not near to each other.) | GCA_002607745 *, GCA_009812315 |
| GFHZ | *Saccharum officinarum* (plant; sugar cane), various strains | Leaf, stalk and root samples of various strains were combined, so hairpin ribozymes cannot be attributed to a specific part or strain of plant. | 30072760 | 3 | 0.011 | GFHZ01110905 (2 hairpin ribozymes in opposite strands), GFHZ01111161 |  |
| GFLP | *Saccharum officinarum* (plant; sugar cane), various strains | Leaf, stalk and root samples of various strains were combined, so hairpin ribozymes cannot be attributed to a specific part or strain of plant. | 30072760 | 15 | 0.00004 | GFLP01288341, GFLP01286833, GFLP01012994, GFLP01441414, GFLP01288095, GFLP01329911, GFLP01271181 (2 hairpin ribozymes in opposite strands), GFLP01149597, GFLP01283058 (2 hairpin ribozymes in opposite strands), GFLP01851030, GFLP01149597, GFLP01287108, GFLP01290863 |  |
| GFLV | *Formica exsecta* (ant; narrow-headed ant) | Samples collected from field colonies of ants. | 29177112 | 1 | 0.00061 | GFLV01046660 | GCA_003651465 * |
| GFZV | *Auricularia auricula-judae* breed Wujin, Quanjin or Banjin (ear fungus) | Fruiting bodies were collected | 30635591 | 1 | 0.038 | GFZV01012863 (3 identical subsequences in the same strand) | GCA_002092955 |
| GGEZ | *Heterobasidion occidentale* PFC5313 (root-rotting fungus) | Grown in lab conditions. Isolated on August 26, 2005 in Ouray County, Colorado by Worrall. | 29611344 | 2 | 0.0009 | GGEZ01023376 (2 hairpin ribozymes in opposite strands) |  |
| GIFI | *Halisarca dujardini* (marine sponge) | Sponges were captured in the wild. Paper implies genome is available from same group, but was not present in GenBank when we checked. | 32084159 | 1 | 0.022 | GIFI01514496 (2 identical sub-sequences in this contig) |  |
| GIFJ | *Halisarca dujardini* (marine sponge) | Sponges were captured in the wild. | 32084159 | 1 | 0.001 | GIFJ01641300 |  |
| HALR | *Platynereis dumerilii* (Dumeril's clam worm) | Grown in lab with mixture of artificial and natural sea water. | 30767890 | 2 | 0.008 | HALR01192984, HALR01183481 |  |
| HAMY | ? | Plant litter metagenome. | 31557276 | 10 | 0.00038 | HAMY010092562, HAMY010944827, HAMY011136267, HAMY010289101, HAMY011241849, HAMY010478751, HAMY010507670, HAMY010552344, HAMY010496177, HAMY010040351 |  |

**Supplementary Table 7.** End repeats are more common in hairpin-ribozyme-containing contigs than in other contigs. We analyzed contigs within spruce litter metatranscriptomes, to allow a clear comparison between hairpin-ribozyme-containing and other contigs. We selected spruce litter metatranscriptomes because they are enriched for hairpin ribozymes.

| **Type of contigs** | **Number of contigs** | **Number of contigs with end repeats of at least 75 nucleotides** | **Percentage of contigs with end repeats** |
| --- | --- | --- | --- |
| All contigs in spruce litter metatranscriptomes | 1,210,750 | 3,637 | 0.3% |
| Contigs in spruce litter metatranscriptomes that contain a hairpin ribozyme | 360 | 113 | 31% |

**Supplementary Table 8.** Number and frequency of hairpin ribozymes in different metatranscriptomic samples. Note: Hairpin ribozyme predictions reflect the 941 ribozymes in the “extra-spruce-initial” alignment (Supplementary Table 2). Rows are sorted in decreasing frequencies of hairpin ribozymes. “Accession”: the accession of the metatranscriptome in the IMG/M database (8). More detailed metadata is available on the IMG/M Web site. Metatranscriptomes in which hairpins did not occur are not shown. “Mb”: total size of the given metatranscriptome in megabases. “Habitat”: description of the environment from which the sample was isolated. “Location”: where the sample was taken. “All hairpins”: statistics of all predicted hairpin ribozymes in the given environment. “#”: number of hairpin ribozymes in the metatranscriptome. “#/Mb”: number divided by the number of megabases of sequence data. Thus, this value is the frequency of hairpin ribozymes within the given metatranscriptome. “Hairpin contigs, <800 nucs”. Contigs with end repeats that contain at least one hairpin ribozyme were counted. In this case, short contigs (fewer than 800 nucleotides in the inferred circular RNA molecule) were considered. “#”: number of such contigs. “#/Mb”: frequency of such contigs. “Hairpin contigs, 800-1800 nucs”: same as previous columns, but for middle-sized contigs (800-1800 nucleotides). “Hairpin contigs, ≥4000 nucs”: same as previous columns, but for large contigs (at least 4000 nucleotides).

|  |  |  |  | **All hairpins** | | **Hairpin contigs, <800 nucs** | | **Hairpin contigs, 800-1800 nucs** | | **Hairpin contigs, ≥4000 nucs** | |  |
| --- | --- | --- | --- | --- | --- | --- | --- | --- | --- | --- | --- | --- |
| **Accession** | **Mb** | **Habitat** | **Location** | **#** | **#/Mb** | **#** | **#/Mb** | **#** | **#/Mb** | **#** | **#/Mb** | |
| 3300023537 | 7 | forest soil | Czech Republic | 15 | 2.214 | 2 | 0.295 | 1 | 0.148 |  |  | |
| 3300019155 | 8 | spruce litter | Czech Republic | 17 | 2.089 | 2 | 0.246 | 5 | 0.614 |  |  | |
| 3300019157 | 6 | spruce litter | Czech Republic | 10 | 1.624 | 1 | 0.162 | 2 | 0.325 |  |  | |
| 3300019162 | 14 | spruce rhizosphere | Czech Republic | 22 | 1.624 | 1 | 0.074 | 2 | 0.148 |  |  | |
| 3300019170 | 9 | spruce litter | Czech Republic | 14 | 1.518 | 2 | 0.217 | 1 | 0.108 |  |  | |
| 3300023673 | 9 | spruce litter | Czech Republic | 14 | 1.506 | 3 | 0.323 | 1 | 0.108 |  |  | |
| 3300019158 | 12 | forest soil | Czech Republic | 15 | 1.260 | 4 | 0.336 | 2 | 0.168 |  |  | |
| 3300023654 | 8 | spruce litter | Czech Republic | 10 | 1.255 | 1 | 0.125 | 1 | 0.125 |  |  | |
| 3300019176 | 13 | spruce rhizosphere | Czech Republic | 16 | 1.204 | 2 | 0.150 | 3 | 0.226 |  |  | |
| 3300019164 | 17 | forest soil | Czech Republic | 20 | 1.202 | 3 | 0.180 | 4 | 0.240 |  |  | |
| 3300023669 | 6 | spruce litter | Czech Republic | 7 | 1.185 | 2 | 0.339 | 1 | 0.169 |  |  | |
| 3300019168 | 7 | spruce litter | Czech Republic | 8 | 1.172 |  |  | 1 | 0.147 |  |  | |
| 3300019181 | 14 | spruce rhizosphere | Czech Republic | 16 | 1.138 | 2 | 0.142 | 2 | 0.142 |  |  | |
| 3300019161 | 15 | spruce rhizosphere | Czech Republic | 17 | 1.136 | 3 | 0.200 | 4 | 0.267 |  |  | |
| 3300023552 | 13 | spruce rhizosphere | Czech Republic | 14 | 1.104 | 3 | 0.237 | 4 | 0.315 |  |  | |
| 3300019156 | 10 | spruce litter | Czech Republic | 11 | 1.073 | 3 | 0.293 |  |  |  |  | |
| 3300023542 | 8 | forest soil | Czech Republic | 9 | 1.068 | 2 | 0.237 | 1 | 0.119 |  |  | |
| 3300019171 | 9 | spruce litter | Czech Republic | 9 | 0.984 | 1 | 0.109 | 2 | 0.219 |  |  | |
| 3300019160 | 10 | spruce litter | Czech Republic | 10 | 0.981 | 3 | 0.294 | 1 | 0.098 |  |  | |
| 3300023678 | 15 | spruce litter | Czech Republic | 14 | 0.933 | 1 | 0.067 | 3 | 0.200 |  |  | |
| 3300019190 | 24 | spruce rhizosphere | Czech Republic | 22 | 0.931 | 3 | 0.127 | 3 | 0.127 |  |  | |
| 3300019166 | 14 | spruce rhizosphere | Czech Republic | 13 | 0.897 | 2 | 0.138 | 2 | 0.138 |  |  | |
| 3300019167 | 7 | spruce litter | Czech Republic | 6 | 0.896 | 1 | 0.149 |  |  |  |  | |
| 3300019186 | 22 | forest soil | Czech Republic | 19 | 0.883 | 1 | 0.046 | 4 | 0.186 |  |  | |
| 3300023536 | 8 | spruce rhizosphere | Czech Republic | 7 | 0.867 | 1 | 0.124 | 3 | 0.371 |  |  | |
| 3300031956 | 18 | spruce litter | Czech Republic | 15 | 0.848 | 2 | 0.113 | 1 | 0.057 |  |  | |
| 3300019180 | 15 | spruce litter | Czech Republic | 13 | 0.841 |  |  | 2 | 0.129 |  |  | |
| 3300019177 | 12 | spruce rhizosphere | Czech Republic | 10 | 0.823 | 2 | 0.165 | 3 | 0.247 |  |  | |
| 3300019179 | 16 | spruce rhizosphere | Czech Republic | 13 | 0.806 | 2 | 0.124 | 1 | 0.062 |  |  | |
| 3300023660 | 10 | spruce rhizosphere | Czech Republic | 8 | 0.800 | 1 | 0.100 | 1 | 0.100 |  |  | |
| 3300031791 | 16 | spruce litter | Czech Republic | 13 | 0.795 | 2 | 0.122 |  |  |  |  | |
| 3300019178 | 11 | forest soil | Czech Republic | 9 | 0.783 | 1 | 0.087 | 3 | 0.261 |  |  | |
| 3300023675 | 12 | spruce litter | Czech Republic | 9 | 0.761 | 2 | 0.169 | 1 | 0.085 |  |  | |
| 3300031809 | 19 | spruce litter | Czech Republic | 14 | 0.755 | 2 | 0.108 | 3 | 0.162 |  |  | |
| 3300031826 | 30 | spruce litter | Czech Republic | 22 | 0.746 | 2 | 0.068 | 2 | 0.068 |  |  | |
| 3300019165 | 16 | forest soil | Czech Republic | 12 | 0.734 | 3 | 0.184 | 1 | 0.061 |  |  | |
| 3300019192 | 26 | spruce rhizosphere | Czech Republic | 18 | 0.705 | 3 | 0.117 | 3 | 0.117 |  |  | |
| 3300030884 | 18 | spruce roots | Oslo, Norway | 12 | 0.676 | 1 | 0.056 | 4 | 0.225 | 1 | 0.056 | |
| 3300031663 | 37 | forest soil | Indiana, USA | 25 | 0.671 | 1 | 0.027 | 7 | 0.188 |  |  | |
| 3300023547 | 12 | spruce rhizosphere | Czech Republic | 8 | 0.664 | 1 | 0.083 |  |  | 1 | 0.083 | |
| 3300023533 | 6 | spruce litter | Czech Republic | 4 | 0.657 | 1 | 0.164 |  |  |  |  | |
| 3300019183 | 20 | spruce rhizosphere | Czech Republic | 13 | 0.639 | 4 | 0.197 | 5 | 0.246 |  |  | |
| 3300030832 | 20 | spruce roots | Oslo, Norway | 13 | 0.639 |  |  | 1 | 0.049 |  |  | |
| 3300031874 | 20 | spruce litter | Czech Republic | 12 | 0.613 | 1 | 0.051 | 1 | 0.051 | 1 | 0.051 | |
| 3300019182 | 22 | spruce rhizosphere | Czech Republic | 13 | 0.594 | 2 | 0.091 | 4 | 0.183 |  |  | |
| 3300031817 | 25 | spruce litter | Czech Republic | 15 | 0.592 | 2 | 0.079 | 3 | 0.118 |  |  | |
| 3300030805 | 34 | spruce roots | Oslo, Norway | 20 | 0.587 | 3 | 0.088 | 4 | 0.117 |  |  | |
| 3300019173 | 10 | spruce litter | Czech Republic | 6 | 0.580 |  |  | 1 | 0.097 |  |  | |
| 3300023541 | 5 | forest soil | Czech Republic | 3 | 0.573 | 1 | 0.191 |  |  |  |  | |
| 3300019188 | 26 | spruce rhizosphere | Czech Republic | 15 | 0.572 | 3 | 0.114 | 1 | 0.038 |  |  | |
| 3300019189 | 25 | forest soil | Czech Republic | 14 | 0.565 | 2 | 0.081 | 2 | 0.081 |  |  | |
| 3300031870 | 20 | spruce litter | Czech Republic | 11 | 0.552 | 1 | 0.050 | 4 | 0.201 |  |  | |
| 3300030978 | 22 | spruce roots | Oslo, Norway | 12 | 0.551 | 1 | 0.046 | 1 | 0.046 |  |  | |
| 3300023656 | 7 | forest soil | Czech Republic | 4 | 0.551 | 1 | 0.138 | 2 | 0.275 |  |  | |
| 3300030913 | 22 | spruce roots | Oslo, Norway | 12 | 0.538 | 4 | 0.179 |  |  |  |  | |
| 3300023556 | 27 | spruce roots | Czech Republic | 14 | 0.526 | 1 | 0.038 | 1 | 0.038 |  |  | |
| 3300023551 | 12 | forest soil | Czech Republic | 6 | 0.518 | 1 | 0.086 |  |  |  |  | |
| 3300019185 | 22 | forest soil | Czech Republic | 11 | 0.499 | 2 | 0.091 | 1 | 0.045 |  |  | |
| 3300031042 | 10 | spruce roots | Oslo, Norway | 5 | 0.499 | 2 | 0.200 | 1 | 0.100 |  |  | |
| 3300023677 | 12 | forest soil | Czech Republic | 6 | 0.494 | 1 | 0.082 | 1 | 0.082 |  |  | |
| 3300019159 | 12 | spruce litter | Czech Republic | 6 | 0.493 | 1 | 0.082 |  |  |  |  | |
| 3300031044 | 28 | spruce roots | Oslo, Norway | 14 | 0.492 | 1 | 0.035 | 3 | 0.105 |  |  | |
| 3300019174 | 10 | spruce litter | Czech Republic | 5 | 0.488 | 2 | 0.195 |  |  |  |  | |
| 3300032027 | 15 | spruce litter | Czech Republic | 7 | 0.477 | 1 | 0.068 | 1 | 0.068 |  |  | |
| 3300019184 | 17 | forest soil | Czech Republic | 8 | 0.477 | 2 | 0.119 |  |  |  |  | |
| 3300031829 | 19 | forest soil | Czech Republic | 9 | 0.470 | 1 | 0.052 | 3 | 0.157 |  |  | |
| 3300023539 | 9 | spruce rhizosphere | Czech Republic | 4 | 0.469 | 1 | 0.117 |  |  |  |  | |
| 3300031869 | 22 | spruce litter | Czech Republic | 10 | 0.451 | 1 | 0.045 | 2 | 0.090 |  |  | |
| 3300019187 | 21 | forest soil | Czech Republic | 9 | 0.435 | 3 | 0.145 | 2 | 0.097 |  |  | |
| 3300030873 | 33 | spruce roots | Oslo, Norway | 14 | 0.425 | 1 | 0.030 | 1 | 0.030 |  |  | |
| 3300032121 | 38 | forest soil | Czech Republic | 16 | 0.423 | 2 | 0.053 | 1 | 0.026 |  |  | |
| 3300023662 | 12 | forest soil | Czech Republic | 5 | 0.413 | 1 | 0.083 |  |  |  |  | |
| 3300031827 | 10 | spruce litter | Czech Republic | 4 | 0.406 |  |  | 1 | 0.102 |  |  | |
| 3300019194 | 25 | forest soil | Czech Republic | 10 | 0.402 | 1 | 0.040 | 4 | 0.161 |  |  | |
| 3300031868 | 36 | spruce litter | Czech Republic | 14 | 0.392 | 4 | 0.112 | 3 | 0.084 |  |  | |
| 3300032028 | 29 | forest soil | Czech Republic | 11 | 0.377 | 1 | 0.034 | 3 | 0.103 |  |  | |
| 3300023672 | 22 | spruce rhizosphere | Czech Republic | 8 | 0.370 | 2 | 0.092 | 1 | 0.046 |  |  | |
| 3300023559 | 30 | spruce roots | Czech Republic | 11 | 0.363 | 2 | 0.066 |  |  |  |  | |
| 3300031808 | 36 | spruce litter | Czech Republic | 13 | 0.361 | 3 | 0.083 | 2 | 0.056 |  |  | |
| 3300031866 | 43 | forest soil | Czech Republic | 15 | 0.350 | 2 | 0.047 | 3 | 0.070 |  |  | |
| 3300031891 | 35 | spruce litter | Czech Republic | 12 | 0.346 | 2 | 0.058 |  |  |  |  | |
| 3300023666 | 26 | spruce roots | Czech Republic | 9 | 0.345 | 1 | 0.038 | 2 | 0.077 |  |  | |
| 3300023544 | 12 | forest soil | Czech Republic | 4 | 0.340 | 1 | 0.085 |  |  |  |  | |
| 3300031872 | 27 | spruce litter | Czech Republic | 9 | 0.332 | 1 | 0.037 | 2 | 0.074 |  |  | |
| 3300031871 | 35 | spruce litter | Czech Republic | 11 | 0.318 | 2 | 0.058 | 2 | 0.058 |  |  | |
| 3300031814 | 17 | spruce rhizosphere | Czech Republic | 5 | 0.302 | 2 | 0.121 | 2 | 0.121 |  |  | |
| 3300032120 | 38 | spruce rhizosphere | Czech Republic | 11 | 0.291 | 1 | 0.026 | 4 | 0.106 |  |  | |
| 3300023664 | 22 | spruce roots | Czech Republic | 6 | 0.276 | 2 | 0.092 |  |  |  |  | |
| 3300030885 | 40 | spruce roots | Oslo, Norway | 11 | 0.272 | 1 | 0.025 |  |  |  |  | |
| 3300032072 | 38 | forest soil | Czech Republic | 10 | 0.266 | 1 | 0.027 | 1 | 0.027 |  |  | |
| 3300030712 | 64 | soil | Risofladan, Finland | 17 | 0.266 | 5 | 0.078 | 1 | 0.016 |  |  | |
| 3300031955 | 43 | spruce litter | Czech Republic | 11 | 0.256 | 2 | 0.047 | 1 | 0.023 |  |  | |
| 3300023558 | 29 | spruce roots | Czech Republic | 7 | 0.241 | 1 | 0.034 |  |  |  |  | |
| 3300030982 | 22 | spruce roots | Oslo, Norway | 5 | 0.230 | 1 | 0.046 |  |  |  |  | |
| 3300030874 | 49 | spruce roots | Oslo, Norway | 11 | 0.223 | 3 | 0.061 | 1 | 0.020 |  |  | |
| 3300031634 | 22 | spruce roots | Oslo, Norway | 5 | 0.222 | 1 | 0.044 | 1 | 0.044 |  |  | |
| 3300023553 | 23 | spruce roots | Czech Republic | 5 | 0.215 |  |  |  |  |  |  | |
| 3300031017 | 33 | spruce roots | Oslo, Norway | 7 | 0.213 | 2 | 0.061 |  |  |  |  | |
| 3300023668 | 29 | spruce roots | Czech Republic | 6 | 0.207 | 2 | 0.069 |  |  |  |  | |
| 3300030942 | 11 | spruce rhizosphere | Czech Republic | 2 | 0.187 | 1 | 0.093 |  |  |  |  | |
| 3300023686 | 28 | spruce roots | Czech Republic | 5 | 0.181 | 1 | 0.036 | 1 | 0.036 |  |  | |
| 3300023564 | 35 | spruce roots | Czech Republic | 6 | 0.170 | 1 | 0.028 |  |  |  |  | |
| 3300031686 | 55 | spruce roots | Oslo, Norway | 9 | 0.163 | 2 | 0.036 |  |  |  |  | |
| 3300010131 | 109 | wetland | Ohio, USA | 17 | 0.156 | 4 | 0.037 | 3 | 0.028 |  |  | |
| 3300031632 | 13 | spruce roots | Oslo, Norway | 2 | 0.156 |  |  |  |  |  |  | |
| 3300023682 | 27 | spruce roots | Czech Republic | 4 | 0.150 |  |  |  |  |  |  | |
| 3300023684 | 27 | spruce roots | Czech Republic | 4 | 0.149 |  |  |  |  |  |  | |
| 3300022502 | 75 | forest soil | Massachusetts, USA | 11 | 0.147 |  |  | 2 | 0.027 |  |  | |
| 3300023680 | 21 | spruce roots | Czech Republic | 3 | 0.143 | 3 | 0.143 |  |  |  |  | |
| 3300023557 | 28 | spruce roots | Czech Republic | 4 | 0.142 | 2 | 0.071 |  |  |  |  | |
| 3300031041 | 28 | spruce roots | Oslo, Norway | 4 | 0.142 |  |  |  |  |  |  | |
| 3300032119 | 59 | spruce forest soil | Czech Republic | 8 | 0.136 | 1 | 0.017 | 1 | 0.017 |  |  | |
| 3300022505 | 82 | spruce forest soil | Massachusetts, USA | 11 | 0.134 | 1 | 0.012 | 5 | 0.061 | 1 | 0.012 | |
| 3300030759 | 69 | spruce roots | Oslo, Norway | 9 | 0.131 | 1 | 0.015 | 4 | 0.058 |  |  | |
| 3300020064 | 164 | groundwater | Colorado, USA | 21 | 0.128 | 2 | 0.012 | 1 | 0.006 | 1 | 0.006 | |
| 3300023561 | 34 | spruce roots | Czech Republic | 4 | 0.119 |  |  |  |  |  |  | |
| 3300023562 | 34 | spruce roots | Czech Republic | 4 | 0.119 | 1 | 0.030 |  |  |  |  | |
| 3300023560 | 34 | spruce roots | Czech Republic | 4 | 0.117 | 1 | 0.029 | 2 | 0.059 |  |  | |
| 3300030718 | 103 | soil | Risofladan, Finland | 12 | 0.116 | 2 | 0.019 |  |  |  |  | |
| 3300023690 | 35 | spruce roots | Czech Republic | 4 | 0.114 | 1 | 0.029 |  |  |  |  | |
| 3300031636 | 46 | spruce roots | Oslo, Norway | 5 | 0.109 |  |  |  |  |  |  | |
| 3300031635 | 46 | spruce roots | Oslo, Norway | 5 | 0.108 |  |  |  |  |  |  | |
| 3300031678 | 37 | spruce roots | Oslo, Norway | 4 | 0.108 | 2 | 0.054 |  |  |  |  | |
| 3300031592 | 48 | spruce roots | Oslo, Norway | 5 | 0.105 |  |  | 2 | 0.042 |  |  | |
| 3300023563 | 29 | spruce roots | Czech Republic | 3 | 0.105 | 1 | 0.035 |  |  |  |  | |
| 3300022499 | 89 | forest soil | Massachusetts, USA | 9 | 0.101 |  |  | 4 | 0.045 |  |  | |
| 3300023688 | 30 | spruce roots | Czech Republic | 3 | 0.101 | 1 | 0.034 |  |  |  |  | |
| 3300030716 | 52 | soil | Risofladan, Finland | 5 | 0.095 |  |  |  |  |  |  | |
| 3300031690 | 43 | spruce roots | Oslo, Norway | 4 | 0.093 |  |  | 1 | 0.023 |  |  | |
| 3300031615 | 57 | root nodule | Oslo, Norway | 5 | 0.088 | 1 | 0.018 |  |  | 1 | 0.018 | |
| 3300021856 | 287 | river | Pennsylvania, USA | 25 | 0.087 | 10 | 0.035 | 1 | 0.003 |  |  | |
| 3300031664 | 35 | spruce roots | Oslo, Norway | 3 | 0.085 | 1 | 0.028 | 1 | 0.028 |  |  | |
| 3300030730 | 484 | forest soil | Indiana, USA | 40 | 0.083 | 1 | 0.002 | 5 | 0.010 | 3 | 0.006 | |
| 3300031667 | 62 | spruce roots | Oslo, Norway | 5 | 0.081 |  |  | 1 | 0.016 |  |  | |
| 3300012751 | 87 | lake | Quebec, Canada | 7 | 0.081 | 2 | 0.023 |  |  |  |  | |
| 3300022508 | 211 | forest soil | Massachusetts, USA | 17 | 0.081 | 2 | 0.009 | 1 | 0.005 | 3 | 0.014 | |
| 3300031040 | 89 | spruce roots | Oslo, Norway | 7 | 0.079 | 2 | 0.023 |  |  |  |  | |
| 3300012691 | 71 | dystrophic lake | Wisconsin, USA | 5 | 0.070 | 2 | 0.028 |  |  |  |  | |
| 3300010138 | 104 | wetland | Ohio, USA | 7 | 0.067 | 1 | 0.010 |  |  |  |  | |
| 3300030814 | 45 | spruce roots | Oslo, Norway | 3 | 0.067 | 1 | 0.022 | 1 | 0.022 |  |  | |
| 3300012754 | 95 | lake | Quebec, Canada | 6 | 0.063 | 1 | 0.010 |  |  |  |  | |
| 3300023689 | 32 | spruce roots | Czech Republic | 2 | 0.062 |  |  | 1 | 0.031 |  |  | |
| 3300031591 | 34 | spruce roots | Oslo, Norway | 2 | 0.059 |  |  |  |  |  |  | |
| 3300030815 | 121 | spruce roots | Oslo, Norway | 7 | 0.058 | 1 | 0.008 | 1 | 0.008 |  |  | |
| 3300016687 | 86 | dystrophic lake | Wisconsin, USA | 5 | 0.058 |  |  |  |  |  |  | |
| 3300000829 | 160 | wet-up | Moab, USA | 9 | 0.056 |  |  |  |  | 4 | 0.025 | |
| 3300012699 | 110 | dystrophic lake | Wisconsin, USA | 6 | 0.055 |  |  |  |  |  |  | |
| 3300020066 | 128 | groundwater | Colorado, USA | 7 | 0.055 |  |  |  |  | 1 | 0.008 | |
| 3300031633 | 39 | spruce roots | Oslo, Norway | 2 | 0.052 |  |  |  |  |  |  | |
| 3300030813 | 175 | spruce roots | Oslo, Norway | 9 | 0.051 |  |  |  |  |  |  | |
| 3300019206 | 118 | wastewater | Illinois, USA | 6 | 0.051 |  |  |  |  |  |  | |
| 3300030940 | 100 | spruce roots | Oslo, Norway | 5 | 0.050 | 1 | 0.010 | 2 | 0.020 |  |  | |
| 3300012411 | 329 | lake | Tavastia Proper, Finland | 16 | 0.049 | 2 | 0.006 | 1 | 0.003 |  |  | |
| 3300016682 | 62 | dystrophic lake | Wisconsin, USA | 3 | 0.049 | 2 | 0.032 |  |  |  |  | |
| 3300012686 | 106 | dystrophic lake | Wisconsin, USA | 5 | 0.047 | 1 | 0.009 |  |  |  |  | |
| 3300019236 | 208 | wastewater | Illinois, USA | 9 | 0.043 |  |  |  |  |  |  | |
| 3300019202 | 97 | wastewater | Niigata Prefecture, Japan | 4 | 0.041 | 2 | 0.021 |  |  |  |  | |
| 3300022510 | 98 | forest soil | Massachusetts, USA | 4 | 0.041 |  |  |  |  |  |  | |
| 3300031677 | 49 | forest soil | Indiana, USA | 2 | 0.041 | 1 | 0.020 | 1 | 0.020 |  |  | |
| 3300000750 | 229 | dry out | Moab, USA | 9 | 0.039 |  |  |  |  |  |  | |
| 3300016678 | 28 | dystrophic lake | Wisconsin, USA | 1 | 0.035 |  |  | 1 | 0.035 |  |  | |
| 3300021853 | 57 | estuarine | Oregon, USA | 2 | 0.035 |  |  |  |  |  |  | |
| 3300016684 | 59 | dystrophic lake | Wisconsin, USA | 2 | 0.034 | 1 | 0.017 |  |  |  |  | |
| 3300022749 | 30 | freshwater | Pennsylvania, USA | 1 | 0.033 |  |  |  |  |  |  | |
| 3300012694 | 95 | dystrophic lake | Wisconsin, USA | 3 | 0.032 |  |  |  |  |  |  | |
| 3300031666 | 32 | spruce roots | Oslo, Norway | 1 | 0.032 | 1 | 0.032 |  |  |  |  | |
| 3300031614 | 64 | spruce roots | Oslo, Norway | 2 | 0.031 | 1 | 0.016 |  |  |  |  | |
| 3300012701 | 130 | dystrophic lake | Wisconsin, USA | 4 | 0.031 | 1 | 0.008 |  |  |  |  | |
| 3300031074 | 33 | plant litter | California, USA | 1 | 0.030 | 1 | 0.030 |  |  |  |  | |
| 3300012770 | 169 | lake | Quebec, Canada | 5 | 0.030 |  |  |  |  |  |  | |
| 3300028623 | 174 | saline water | British Columbia, Canada | 5 | 0.029 |  |  |  |  |  |  | |
| 3300029680 | 70 | saline water | British Columbia, Canada | 2 | 0.029 |  |  |  |  |  |  | |
| 3300022507 | 123 | forest soil | Massachusetts, USA | 3 | 0.024 |  |  |  |  |  |  | |
| 3300022722 | 403 | forest soil | Massachusetts, USA | 9 | 0.022 |  |  | 2 | 0.005 |  |  | |
| 3300022509 | 135 | forest soil | Massachusetts, USA | 3 | 0.022 |  |  |  |  |  |  | |
| 3300030862 | 227 | spruce roots | Oslo, Norway | 5 | 0.022 |  |  | 1 | 0.004 |  |  | |
| 3300031071 | 46 | plant litter | California, USA | 1 | 0.022 | 1 | 0.022 |  |  |  |  | |
| 3300022726 | 697 | forest soil | Massachusetts, USA | 15 | 0.022 |  |  | 3 | 0.004 | 1 | 0.001 | |
| 3300019203 | 142 | wastewater | Hyogo Prefecture, Japan | 3 | 0.021 | 1 | 0.007 |  |  |  |  | |
| 3300003684 | 99 | dead zone in bay | Sandusky Bay, USA | 2 | 0.020 |  |  |  |  |  |  | |
| 3300031560 | 104 | saline water | British Columbia, Canada | 2 | 0.019 |  |  |  |  |  |  | |
| 3300019222 | 158 | wastewater | Hyogo Prefecture, Japan | 3 | 0.019 | 1 | 0.006 |  |  |  |  | |
| 3300028670 | 105 | saline water | British Columbia, Canada | 2 | 0.019 | 1 | 0.009 |  |  |  |  | |
| 3300012689 | 110 | dystrophic lake | Wisconsin, USA | 2 | 0.018 |  |  |  |  |  |  | |
| 3300024532 | 169 | river | Louisiana, USA | 3 | 0.018 | 1 | 0.006 |  |  |  |  | |
| 3300019223 | 171 | wastewater | Niigata Prefecture, Japan | 3 | 0.018 | 2 | 0.012 |  |  |  |  | |
| 3300029689 | 114 | saline water | British Columbia, Canada | 2 | 0.018 | 1 | 0.009 |  |  |  |  | |
| 3300029667 | 57 | wastewater | Wisconsin, USA | 1 | 0.017 |  |  |  |  |  |  | |
| 3300019209 | 118 | wastewater | Saitama Prefecture, Japan | 2 | 0.017 | 1 | 0.008 |  |  |  |  | |
| 3300003693 | 118 | Avena fatua | California, USA | 2 | 0.017 |  |  |  |  |  |  | |
| 3300006596 | 180 | wastewater | Illinois, USA | 3 | 0.017 |  |  |  |  |  |  | |
| 3300023703 | 122 | river | Georgia, USA | 2 | 0.016 |  |  |  |  |  |  | |
| 3300012766 | 132 | lake | Quebec, Canada | 2 | 0.015 |  |  |  |  |  |  | |
| 3300019216 | 134 | wastewater | Illinois, USA | 2 | 0.015 |  |  |  |  |  |  | |
| 3300012747 | 72 | dystrophic lake | Wisconsin, USA | 1 | 0.014 |  |  |  |  |  |  | |
| 3300019205 | 146 | wastewater | Illinois, USA | 2 | 0.014 |  |  |  |  |  |  | |
| 3300012692 | 75 | dystrophic lake | Wisconsin, USA | 1 | 0.013 |  |  |  |  |  |  | |
| 3300028619 | 150 | saline water | British Columbia, Canada | 2 | 0.013 | 1 | 0.007 |  |  |  |  | |
| 3300031590 | 77 | forest soil | Indiana, USA | 1 | 0.013 |  |  |  |  |  |  | |
| 3300028621 | 161 | saline water | British Columbia, Canada | 2 | 0.012 | 1 | 0.006 |  |  |  |  | |
| 3300024851 | 178 | river | Louisiana, USA | 2 | 0.011 |  |  |  |  |  |  | |
| 3300012749 | 92 | dystrophic lake | Wisconsin, USA | 1 | 0.011 |  |  |  |  |  |  | |
| 3300012696 | 96 | dystrophic lake | Wisconsin, USA | 1 | 0.010 |  |  |  |  |  |  | |
| 3300019226 | 193 | wastewater | Illinois, USA | 2 | 0.010 |  |  |  |  |  |  | |
| 3300022185 | 292 | wetland | Ohio, USA | 3 | 0.010 |  |  |  |  |  |  | |
| 3300029638 | 108 | saline water | British Columbia, Canada | 1 | 0.009 |  |  |  |  |  |  | |
| 3300012698 | 108 | dystrophic lake | Wisconsin, USA | 1 | 0.009 |  |  |  |  |  |  | |
| 3300023700 | 114 | river | Georgia, USA | 1 | 0.009 |  |  |  |  |  |  | |
| 3300006590 | 114 | wastewater | Illinois, USA | 1 | 0.009 |  |  |  |  |  |  | |
| 3300006935 | 115 | rainforest soil | Amazon Forest, Brazil | 1 | 0.009 |  |  |  |  |  |  | |
| 3300029691 | 119 | saline water | British Columbia, Canada | 1 | 0.008 | 1 | 0.008 |  |  |  |  | |
| 3300000833 | 359 | wet-up | Moab, USA | 3 | 0.008 |  |  |  |  |  |  | |
| 3300019213 | 127 | wastewater | Saitama Prefecture, Japan | 1 | 0.008 |  |  |  |  |  |  | |
| 3300012703 | 128 | dystrophic lake | Wisconsin, USA | 1 | 0.008 |  |  |  |  |  |  | |
| 3300029693 | 133 | saline water | British Columbia, Canada | 1 | 0.008 |  |  |  |  |  |  | |
| 3300028620 | 135 | saline water | British Columbia, Canada | 1 | 0.007 |  |  |  |  |  |  | |
| 3300019212 | 148 | groundwater | Colorado, USA | 1 | 0.007 |  |  |  |  |  |  | |
| 3300012768 | 150 | lake | Quebec, Canada | 1 | 0.007 |  |  |  |  |  |  | |
| 3300028668 | 153 | saline water | British Columbia, Canada | 1 | 0.007 |  |  |  |  |  |  | |
| 3300012769 | 154 | lake | Quebec, Canada | 1 | 0.007 |  |  |  |  |  |  | |
| 3300012524 | 311 | salinity gradient | Chesapeake Bay, USA | 2 | 0.006 |  |  |  |  |  |  | |
| 3300022467 | 973 | corn rhizosphere | Michigan, USA | 6 | 0.006 |  |  | 1 | 0.001 | 1 | 0.001 | |
| 3300024542 | 167 | river | Louisiana, USA | 1 | 0.006 | 1 | 0.006 |  |  |  |  | |
| 3300010154 | 671 | creek | Wisconsin, USA | 4 | 0.006 |  |  |  |  |  |  | |
| 3300016766 | 340 | coastal | Georgia, USA | 2 | 0.006 |  |  |  |  |  |  | |
| 3300020076 | 348 | corn rhizosphere | Michigan, USA | 2 | 0.006 |  |  |  |  |  |  | |
| 3300019241 | 176 | tropical peat soil | Colombia | 1 | 0.006 |  |  |  |  |  |  | |
| 3300012774 | 178 | lake | Quebec, Canada | 1 | 0.006 |  |  |  |  |  |  | |
| 3300019217 | 180 | wastewater | Niigata Prefecture, Japan | 1 | 0.006 | 1 | 0.006 |  |  |  |  | |
| 3300019227 | 183 | wastewater | Hyogo Prefecture, Japan | 1 | 0.005 | 1 | 0.005 |  |  |  |  | |
| 3300019225 | 185 | wastewater | Saitama Prefecture, Japan | 1 | 0.005 |  |  |  |  |  |  | |
| 3300012775 | 185 | lake | Quebec, Canada | 1 | 0.005 | 1 | 0.005 |  |  |  |  | |
| 3300012776 | 204 | lake | Quebec, Canada | 1 | 0.005 |  |  |  |  |  |  | |
| 3300019231 | 216 | wastewater | Illinois, USA | 1 | 0.005 |  |  |  |  |  |  | |
| 3300019247 | 227 | wastewater | Illinois, USA | 1 | 0.004 |  |  |  |  |  |  | |
| 3300011333 | 682 | corn rhizosphere | Wisconsin, USA | 3 | 0.004 |  |  |  |  |  |  | |
| 3300012777 | 229 | lake | Quebec, Canada | 1 | 0.004 |  |  |  |  |  |  | |
| 3300024536 | 242 | river | Georgia, USA | 1 | 0.004 |  |  |  |  |  |  | |
| 3300024867 | 254 | river | Georgia, USA | 1 | 0.004 |  |  |  |  |  |  | |
| 3300020070 | 1032 | corn rhizosphere | Michigan, USA | 4 | 0.004 |  |  |  |  |  |  | |
| 3300024572 | 291 | river | Georgia, USA | 1 | 0.003 |  |  |  |  |  |  | |
| 3300010152 | 588 | delta | Oklahoma, USA | 2 | 0.003 |  |  |  |  |  |  | |
| 3300024486 | 907 | cow rumen | Palmerston North, New Zealand | 1 | 0.001 |  |  |  |  |  |  | |
| 3300024345 | 971 | cow rumen | Palmerston North, New Zealand | 1 | 0.001 |  |  |  |  |  |  | |
| 3300020082 | 1147 | corn rhizosphere | Michigan, USA | 1 | 0.001 |  |  |  |  |  |  | |

**Supplementary Table 9.** Number and frequency of hairpin ribozymes in different environments. The data from Supplementary Table 8 is aggregated by the “Habitat” column. The meanings of the columns are the same as in Supplementary Table 8. Rows are sorted in decreasing frequencies of hairpin ribozymes.

|  |  | **All hairpins** | | **Hairpin contigs, <800 nucs** | | **Hairpin contigs, 800-1800 nucs** | | **Hairpin contigs, ≥4000 nucs** | |
| --- | --- | --- | --- | --- | --- | --- | --- | --- | --- |
| **Mb** | **Habitat** | **#** | **#/Mb** | **#** | **#/Mb** | **#** | **#/Mb** | **#** | **#/Mb** |
| 354 | spruce rhizosphere | 255 | 0.720339 | 42 | 0.118644 | 48 | 0.135593 | 1 | 0.002825 |
| 576 | spruce litter | 366 | 0.635417 | 54 | 0.09375 | 50 | 0.086806 | 1 | 0.001736 |
| 2398 | spruce roots | 353 | 0.147206 | 56 | 0.023353 | 38 | 0.015847 | 2 | 0.000834 |
| 3049 | forest soil | 398 | 0.130535 | 46 | 0.015087 | 70 | 0.022958 | 8 | 0.002624 |
| 439 | groundwater | 29 | 0.066059 | 2 | 0.004556 | 1 | 0.002278 | 2 | 0.004556 |
| 527 | wetland | 27 | 0.051233 | 5 | 0.009488 | 3 | 0.005693 |  | 0 |
| 30 | freshwater | 1 | 0.033333 |  | 0 |  | 0 |  | 0 |
| 99 | dead zone in bay | 2 | 0.020202 |  | 0 |  | 0 |  | 0 |
| 2260 | dystrophic lake | 42 | 0.018584 | 7 | 0.003097 | 1 | 0.000442 |  | 0 |
| 2297 | lake | 42 | 0.018285 | 6 | 0.002612 | 1 | 0.000435 |  | 0 |
| 660 | (desert soil) wet-up | 12 | 0.018182 |  | 0 |  | 0 | 4 | 0.006061 |
| 118 | Avena fatua | 2 | 0.016949 |  | 0 |  | 0 |  | 0 |
| 587 | dry out | 9 | 0.015332 |  | 0 |  | 0 |  | 0 |
| 1892 | saline water | 22 | 0.011628 | 5 | 0.002643 |  | 0 |  | 0 |
| 240 | estuarine | 2 | 0.008333 |  | 0 |  | 0 |  | 0 |
| 6454 | wastewater | 47 | 0.007282 | 9 | 0.001394 |  | 0 |  | 0 |
| 671 | creek | 4 | 0.005961 |  | 0 |  | 0 |  | 0 |
| 376 | plant litter | 2 | 0.005319 | 2 | 0.005319 |  | 0 |  | 0 |
| 4182 | corn rhizosphere | 16 | 0.003826 |  | 0 | 1 | 0.000239 | 1 | 0.000239 |
| 636 | salinity gradient | 2 | 0.003145 |  | 0 |  | 0 |  | 0 |
| 735 | delta | 2 | 0.002721 |  | 0 |  | 0 |  | 0 |
| 15042 | soil | 34 | 0.00226 | 7 | 0.000465 | 1 | 6.65E-05 |  | 0 |
| 523 | tropical peat soil | 1 | 0.001912 |  | 0 |  | 0 |  | 0 |
| 26175 | river | 37 | 0.001414 | 12 | 0.000458 | 1 | 3.82E-05 |  | 0 |
| 5741 | coastal | 2 | 0.000348 |  | 0 |  | 0 |  | 0 |
| 2891 | rainforest soil | 1 | 0.000346 |  | 0 |  | 0 |  | 0 |
| 8462 | cow rumen | 2 | 0.000236 |  | 0 |  | 0 |  | 0 |

**Supplementary Table 10**. Searches for other permutations of known self-cleaving ribozymes other than hairpin ribozymes. Hairpin ribozyme permutations that we searched for are described in Supplementary Figure 3A. “Ribozyme structural class”: one of the nine currently known self-cleaving ribozyme classes (11–13). We did not search for hammerhead or twister permutations, since all expected circular permutations are known. We did not search for permuted *glmS* ribozymes, because it is not a constitutive self-cleaving ribozyme, but is rather a riboswitch-ribozyme. The VS ribozyme did not seem a promising candidate because of its relatively large structure (in comparison to other self-cleaving ribozymes). We reported searches for hairpin permutations in the main text. “Description of permutation”: we formed circular permutations by making the 5′ and 3′ openings in the terminal loop of another stem. The stem names can be found in (13), and papers cited therein. The text “opening in P1” means that the terminal loop of the “P1“ stem forms the 5′ and 3′ openings of the permuted molecule. “Software used”: we used either RNAMotif (14) or DARN! (15) for the searches. All searches were conducted on all sequences in RefSeq and our collected metagenomic and metatranscriptomic sequences. Conserved features of the ribozymes were found based on alignments in the ZWD repository (16) at https://bitbucket.org/zashaw/zashaweinbergdata/src/master/. The patterns can be found in Supplementary File 1. The searches of the P2 and P3 permutations of twister-sister ribozymes on the complete RefSeq database uncovered 125,181 and 28,074 matches, respectively. Therefore, we were not able to analyze all predicted matches. We did not find any promising predictions of previously unknown permutations based on the searches listed in this table.

| **Ribozyme structural class** | **Description of permutation** | **Software used** |
| --- | --- | --- |
| Hatchet | Opening at P1 | RNAMotif |
| Hatchet | Opening at P4 | RNAMotif |
| HDV | Opening at P1, based on conserved features in the HDV-F-prausnitzii alignment in the ZWD repository, which is based on a previously reported HDV ribozyme (17) | DARN! |
| HDV | Opening at P1, based on conserved features in the RAGATH-2-HDV alignment in the ZWD repository, which is based on a previously reported alignment (18) | DARN! |
| HDV | Opening at P4. (In this case, the conserved patterns of available HDV ribozyme alignments were combined.) | DARN! |
| Pistol | Opening at P3 | DARN! |
| twister-sister | Opening at P2 | DARN! |
| twister-sister | Opening at P3 | DARN! |
| twister-sister | Opening at P5 | DARN! |

## Supplementary Figures


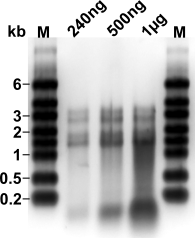
**Supplementary Figure 1.** Analysis of total RNA isolated from spruce litter. RNA integrity was confirmed by separation of 240 ng, 500 ng and 1000 ng of total RNA on 1% agarose gel stained with ethidium bromide. M: the RiboRuler high range (ThermoScientific) was used as a marker. Because RNA was isolated from an environment, a mixture of eukaryotic and prokaryotic RNAs is expected. Prominent bands around 2 kb likely correspond to prokaryotic 16S rRNA (~ 1.5 kb) and eukaryotic 18S rRNA (~ 1.9 kb). Bands around 3 kb likely represent prokaryotic 23S rRNA (~2.9 kb) and eukaryotic (plant) 25S rRNA (~3.7 kb). This analysis indicates that bacterial as well as plant species are likely present in this environment.

**Supplementary Figure 2.** PCR amplification of cDNA derived from predicted circular RNA corresponding to contig Ga0247519_111615 (see text). (**A**) PCR products amplified from a reverse transcription reaction (using primer CEW344) carried out with (+) or without (-) reverse transcriptase (RT). Primers CEW347 and CEW343 were used to create PCR product of 519 bp that corresponds to a sequence that starts at position 578, runs through position 1 and ends at position 484 of contig Ga0247519_111615 (Figure 4A, “P2”). PCR products were separated on a 1% agarose gel. “n.c.”: negative control, i.e. no template was used in the PCR reaction. “M”: the 2log ladder (NEB) was used as a size standard. Lanes shown are part of the same agarose gel. (**B**) PCR products amplified from a reverse transcription reaction (using primer CEW340) carried out with (+) or without (-) reverse transcriptase (RT). Primers CEW346 and CEW341 were used to create PCR product of 533 bp that corresponds to a sequence that starts at position 152, runs through position 1 and ends at position 72 of contig Ga0247519_111615 (Figure 4A, “P1”). Lanes shown are part of the same agarose gel. (**C**) PCR products amplified from a reverse transcription reaction (using primer CEW343) carried out with (+) or without (-) reverse transcriptase (RT). Primers CEW345 and CEW347 were used to create PCR product of 488 bp that corresponds to a sequence that starts at position 578, runs through position 1 and ends at position 453 of contig Ga0247519_111615 (Figure 4B, “P4”). Lanes shown are part of the same agarose gel. (**D)** PCR products amplified from a reverse transcription reaction (using primer CEW336) carried out with (+) or without (-) reverse transcriptase (RT). Primers CEW341 and CEW340 were used to create PCR product of 564 bp that corresponds to a sequence that starts at position 121, runs through position 1 and ends at position 72 of contig Ga0247519_111615 (Figure 4B, “P3”). Lanes shown are part of the same agarose gel.


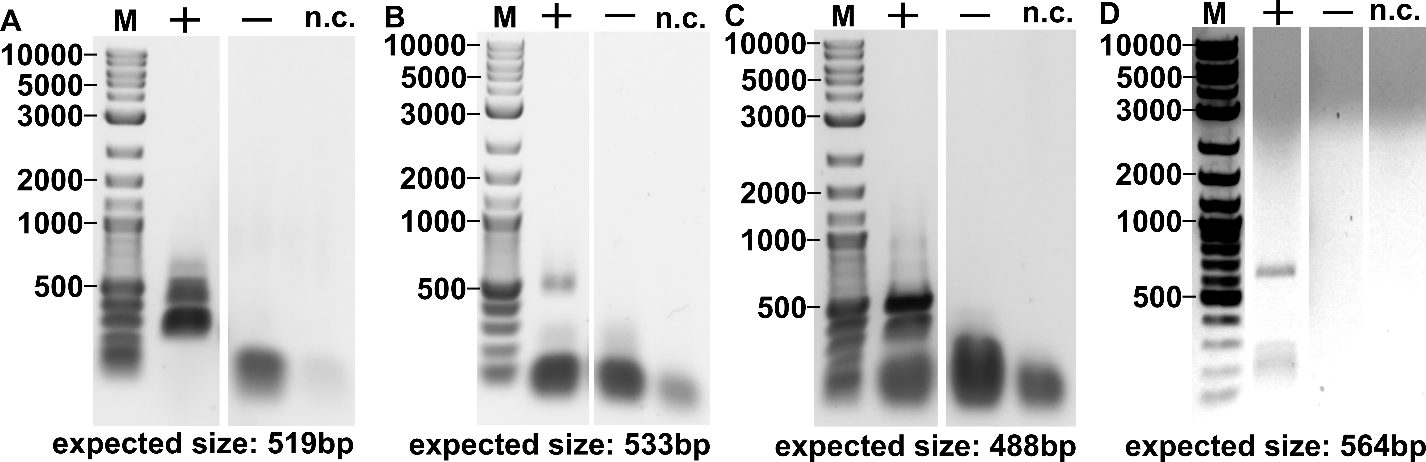


**Supplementary Figure 3**. Hairpin permutations. (**A**) Sixteen theoretical permutations of hairpin ribozymes are shown. The schematic depictions follow the layout in Figure 1. However, in the lower two rows, helices 3 and 4 are exchanged by rotating them and the intervening internal loop by 180 degrees. The previously proposed permutation is outlined in blue, as is the permutation for the 941 unique hairpin sequences we found. Note: it is not necessary to also consider exchanging helices 1 and 2, as the resulting permutation is already present within the 16 permutations shown. For example, exchanging helices 1 and 2 in the top, left permutation (corresponding to the previously published permutation) is equivalent to the bottom, left permutation. DARN! search patterns for each of the hairpin permutations are provided (Supplementary File 1). (**B**) The previously published permutation and the newly found permutation are distinguished by the location of a “distinguishing conserved region” (DCR, shaded in light gray). The DCR appears either at the 3′ end (previously established permutation) or the 5′ end (newly found permutation). Each permutation is depicted according to its secondary structure and in a linear version (without base pairs). The nucleotides shown are arbitrary and just intended to illustrate the permutations. The annotations “potentially long insertion” indicate the location at which we often observe large insertions of many nucleotides. The amount of conservation in the DCR is less than that of the remaining part of the hairpin ribozyme (which includes loop B and half of loop A). It is possible that the relatively small DCR can appear to occur 5′ or 3′ to the rest of the ribozyme structure, a possibility that could make the permutation of an individual sequence potentially unclear. However, we consistently observed potential for DCRs that occur 5′ to the main conserved features (corresponding to the newly found permutation), and did not observe consistent potential 3′ to the main conserved features (previously established permutation).


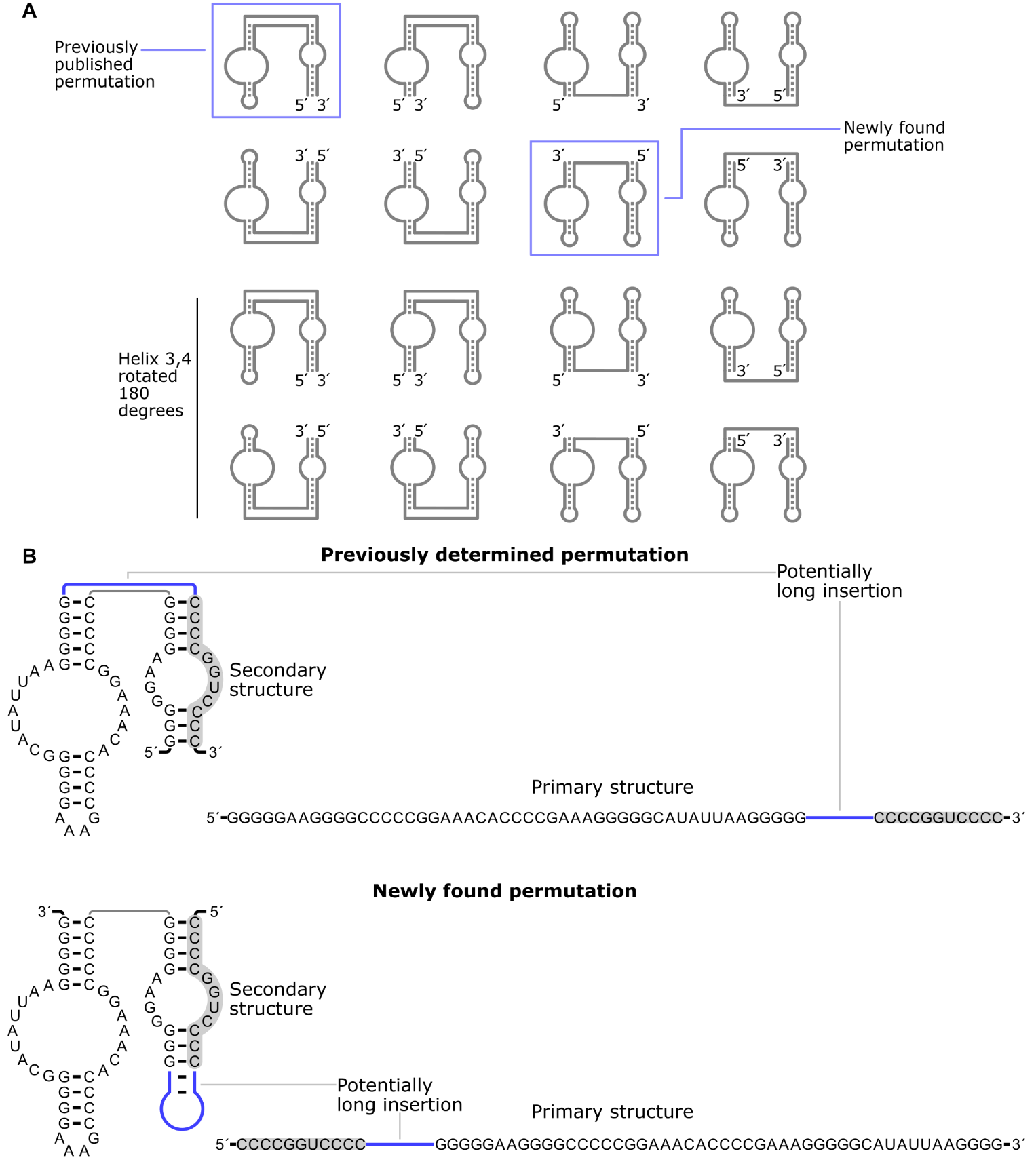


**Supplementary Figure 4**. Schematic of our workflow to find hairpin ribozymes. (**A**) We enumerated 16 possible permuted forms of hairpin ribozymes. See also Supplementary Figure 3A. (**B**) For each of the 16 permutations, we created a pattern using the DARN! software (15) to specify sequences conforming to that hairpin ribozyme permutation. We searched for matches to each of these patterns within a sequence database defined in Materials and Methods. (**C**) For each sequence match to one of our permutation patterns, we manually analyzed the structural match and nucleotide composition, and eliminated subjectively improbable predictions. For the remaining predictions, we used the single sequence along with the expected hairpin-ribozyme-like secondary structure to search for similar sequences using the Infernal software (10) with an E-value threshold of 10, resulting in a multiple-sequence alignment. (In a later analysis, we instead used a lower, more stringent E-value threshold; see Supplementary Table 2.) We manually analyzed the resulting alignments for convincing covariation (i.e. covariation in the context of a likely correct alignment), and rejected sequence matches from part B that did not yield additional sequences (thus trivially lacking covariation), alignments that did not have convincing covariation and alignments that did not have plausibly conserved stems corresponding to the hairpin ribozyme structure. We also rejected some individual sequences from each accepted alignment that did not seem to fit the hairpin structure based on a subjective analysis. Only sequences corresponding to the new permutation proposed in this paper passed this stage. (**D**) We repeatedly used the resulting alignments in Infernal searches with an E-value threshold of 10 and manual elimination of implausible sequence matches, until the search produced no new hits. (**E**) This resulted in an alignment for each sequence from part B that was not rejected. At this stage, we rejected alignments that largely contained the same hits as other alignments, and ended up with only two alignments. (The second alignment was not further analyzed, but is described in Supplementary Table 2 and available in Supplementary Files 2 and 3.) (**F**) We downloaded additional metatranscriptomic sequences focusing on spruce-related environments, and conducted an automated search with Infernal, this time with a more stringent E-value threshold of 0.1. We did not manually analyze this alignment, except that we removed sequences that appeared to be truncated at the 5′ or 3′ end.

**
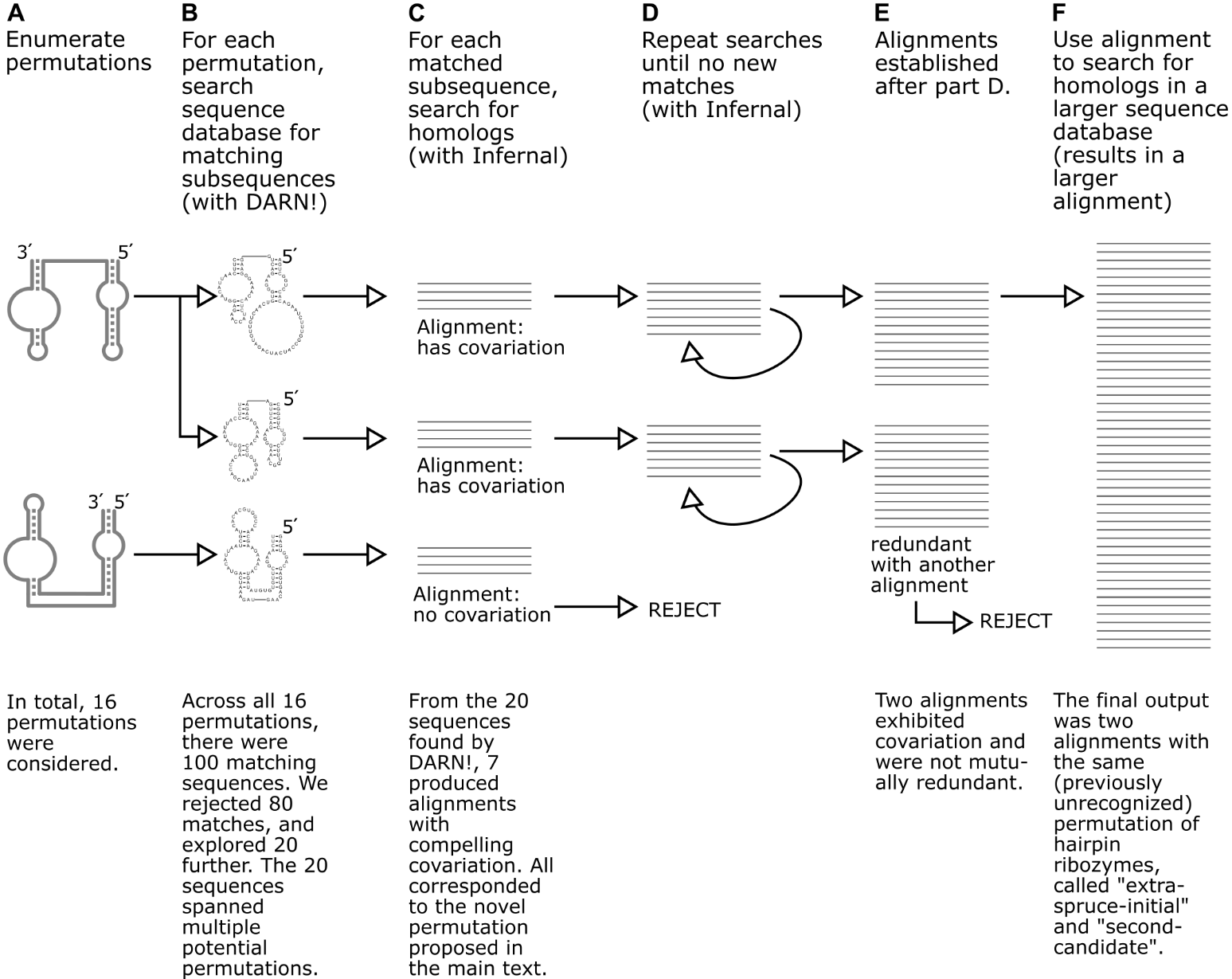
**

**Supplementary Figure 5.** Secondary structure and *in vitro* cleavage of two additional hairpin ribozyme representatives. Secondary structure diagram of hairpin ribozyme representative HPR 2 (**A**) and HPR 3 (**B**). M1 indicates a nucleotide exchange leading to a strongly decreased ribozyme cleavage speed (19). Annotations are the same as in Figure 1D. (**C**) Hairpin ribozyme candidate HPR 2 was *in vitro* transcribed in the presence of [α-^32^P]-ATP for 60 minutes (WT). Bands correspond to a 3′ cleavage product of 66 nucleotides and a 5′ cleavage product of 20 nucleotides. (**D**) Hairpin ribozyme candidate from HPR 3 was *in vitro* transcribed in the presence of α-^32^P-ATP for 60 minutes (WT). Bands correspond to a 3′ cleavage product of 68 nucleotides and a 5′ cleavage product of 20 nucleotides. 5′-radioactively labeled M1 RNAs were used to create size standards by partial digestion with RNase T1 or partial alkaline hydrolysis (“ˉOH”).

**
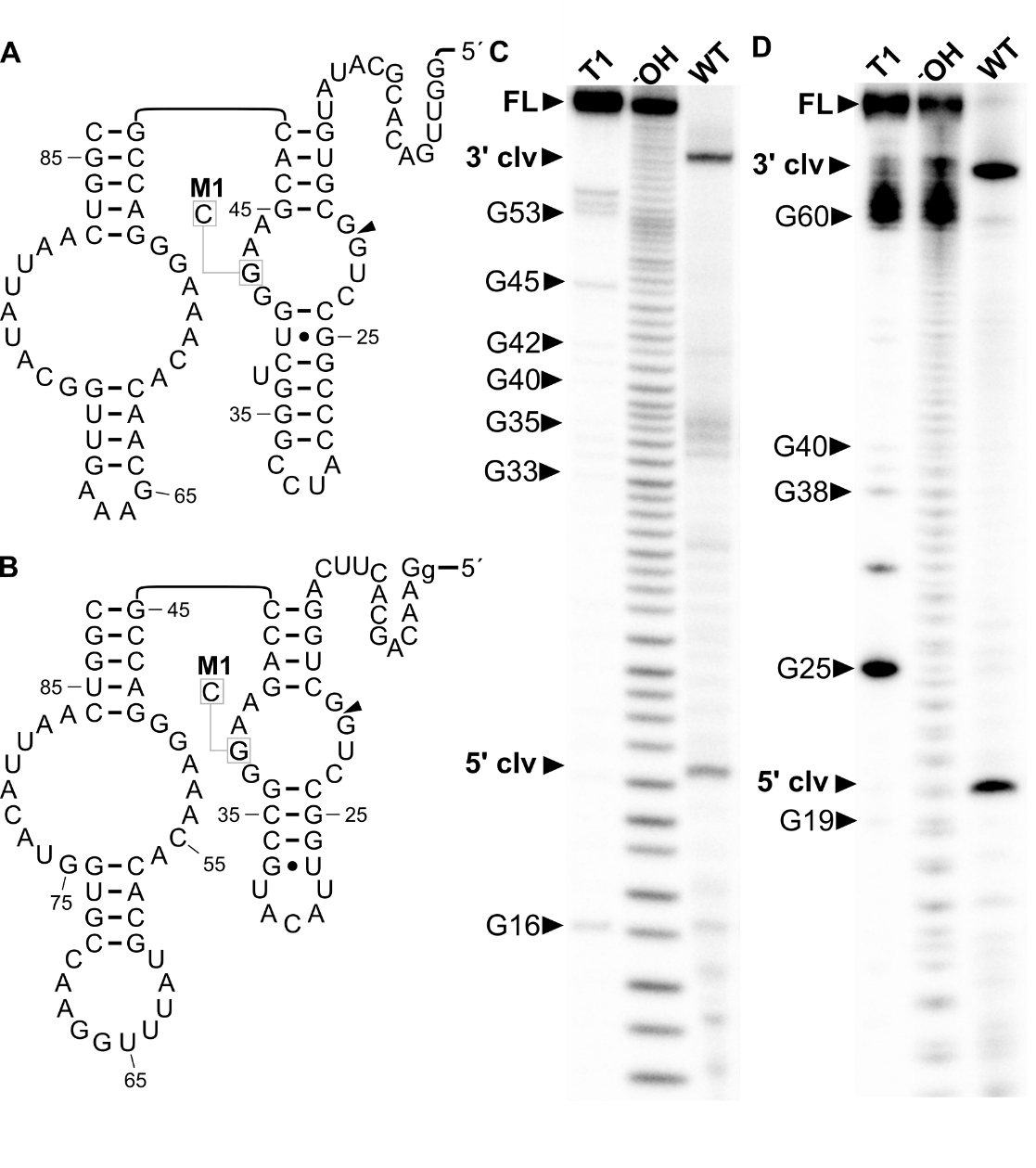
**

**Supplementary Figure 6.** Secondary structure of hairpin ribozyme representatives that were identified with the five worst E-values (Ev) during homology searches. Secondary structure diagrams of hairpin ribozyme representatives with an E-value of 4.8 (**A**), 5 (**B**), 5.6 (**C**), 6.6 (**D**) and 7 (**E**). M1 indicates a nucleotide exchange in the representatives Ev6.6 and Ev4.8 leading to decreased ribozyme cleavage speed (Supplementary Figure 7). Annotations are as in Figure 1D. We manually determined a possible secondary structure for each representative.


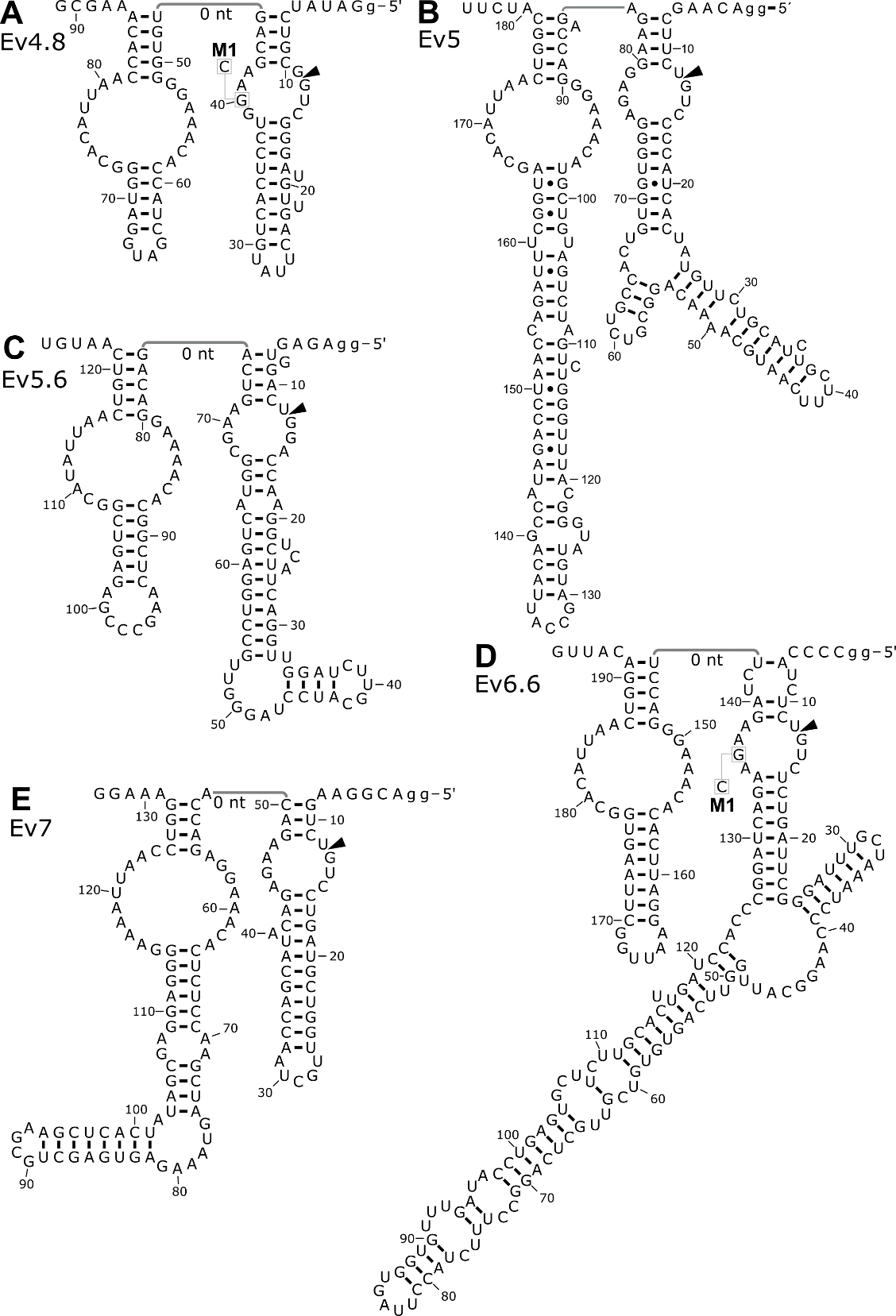


**Supplementary Figure 7.** *In vitro* cleavage of hairpin ribozyme representatives that were identified with the five worst E-values (Ev) during homology searches (Supplementary Figure 6). (**A**) Hairpin ribozyme representatives were *in vitro* transcribed in the presence of [α-^32^P]-ATP for 60 minutes. In a 10% denaturing PAGE, full length (FL) and 3′ cleavage products were separated. The 3′ cleavage products have a size of 121 nucleotides (Ev7), 184 nucleotides (Ev6.6), 114 nucleotides (Ev5.6), 172 nucleotides (Ev5) and 79 nucleotides (Ev4.8), respectively. Hairpin ribozyme variant Ev6.6 M1 and Ev4.8 M1 show a stronger full-length band compared to the WT illustrating its reduced cleavage speed. We used its 3ʹ cleavage product (radioactively labeled at the 5ʹ-end) to create size standards by partial digestion with RNase T1 or partial alkaline hydrolysis (“ˉOH“). (**B**) For the detection of the 5′ cleavage product, a 15% denaturing PAGE was used. The 5′ cleavage products have a size of 12 nucleotides (Ev7, Ev6.6, Ev5.6, Ev5) or 11 nucleotides (Ev4.8).

**
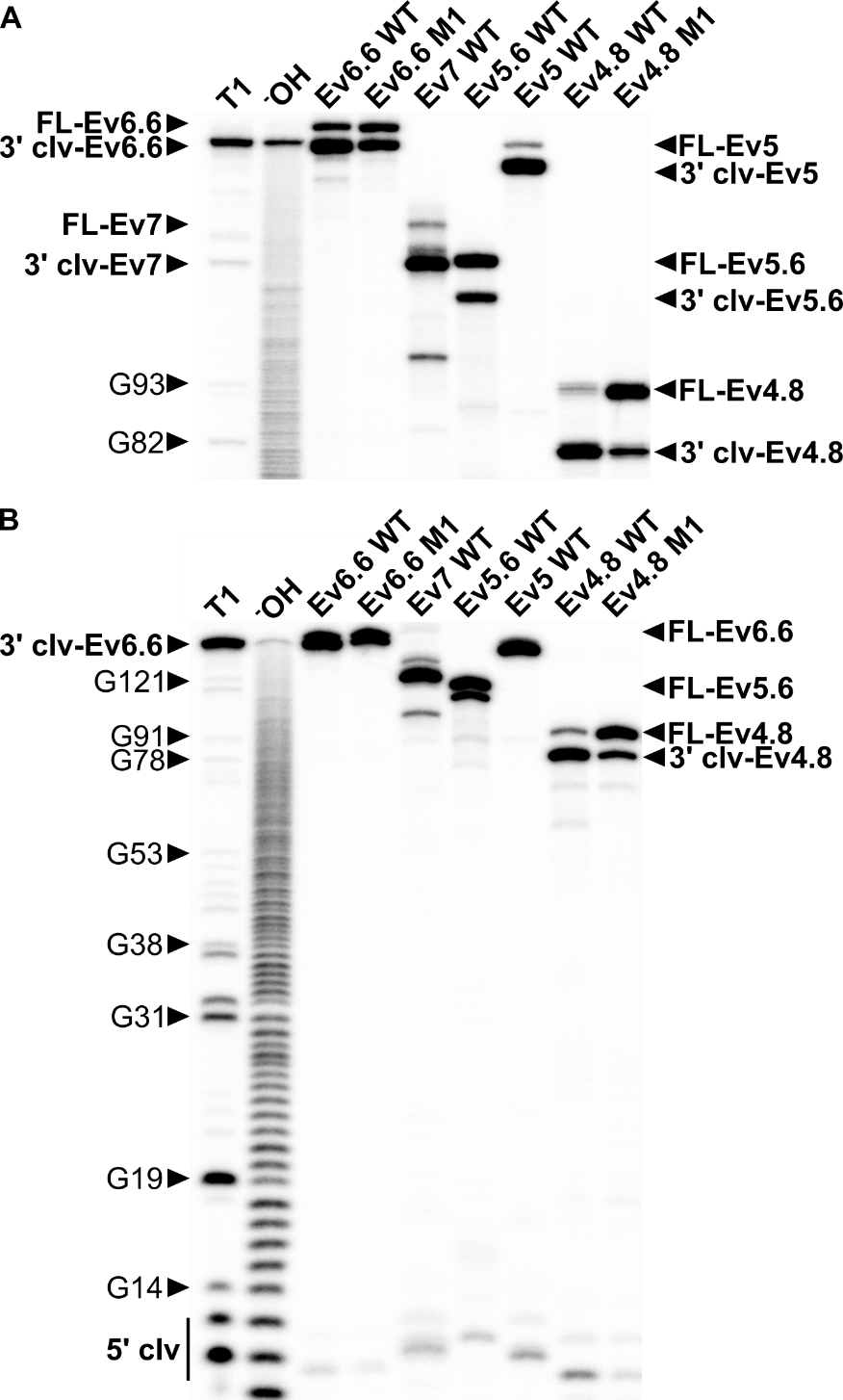
**

**Supplementary Figure 8.** Analysis of non-standard interactions in 941 hairpin ribozymes. (**A**) The newly found hairpin ribozymes are drawn to emphasize nucleotide interactions that do not correspond to standard Watson-Crick pairing. Base pairs are indicated using Leontis-Westhof Notation (20) where possible, as shown in the legend. The interactions depicted here are based on a crystal structure of a hairpin ribozyme (21) and reanalysis of the structure using Leontis-Westhof Notation (22,23). An asterisk indicates an interaction based on the crystal structure (21) that was not assigned a specific type in Leontis-Westhof Notation. The ribose zipper depicted is not further analyzed, because it is not a pair-wise interaction. (**B**) Non-standard interactions in loop A. The frequencies of nucleotide combinations are depicted in the table labeled with interacting nucleotides (e.g., “R7” and “C+3”), which refer to the numbering scheme in part A. Black numbers are the number of occurrences within the 941 hairpin ribozymes. Green triangles indicate new nucleotide combinations present in candidate hairpin ribozymes that we experimentally tested (Supplementary Table 4). Gray numbers reflect the four previously established hairpin ribozymes. For characterized base-pair geometries in Leontis-Westhof Notation, an isostericity matrix is provided, which is adapted from a previous study (20). The isostericity matrices indicate which combinations of nucleotides adopt compatible positions according to a particular type of interaction. Spatially compatible pairs are indicated by isostericity classes, called “I1”, “I2”, etc. “i2” indicates that wobble pairs are not symmetric (21). Blank spaces in isostericity matrices indicate pairs that were not predicted to occur and were not observed in any available atomic-resolution structure. Cells in the isostericity matrices are flanked by gray vertical lines when they were observed in the four previously known hairpin ribozymes. Those found only in the 941 new hairpin ribozymes are boxed in solid black. (**C**) Non-standard interactions in loop B. Annotations are the same as in part B. The A24-A38 pairing is not associated with a known isostericity matrix. (**D**) A base-pair interaction between loops A and B. Annotations are the same as in part B.

**
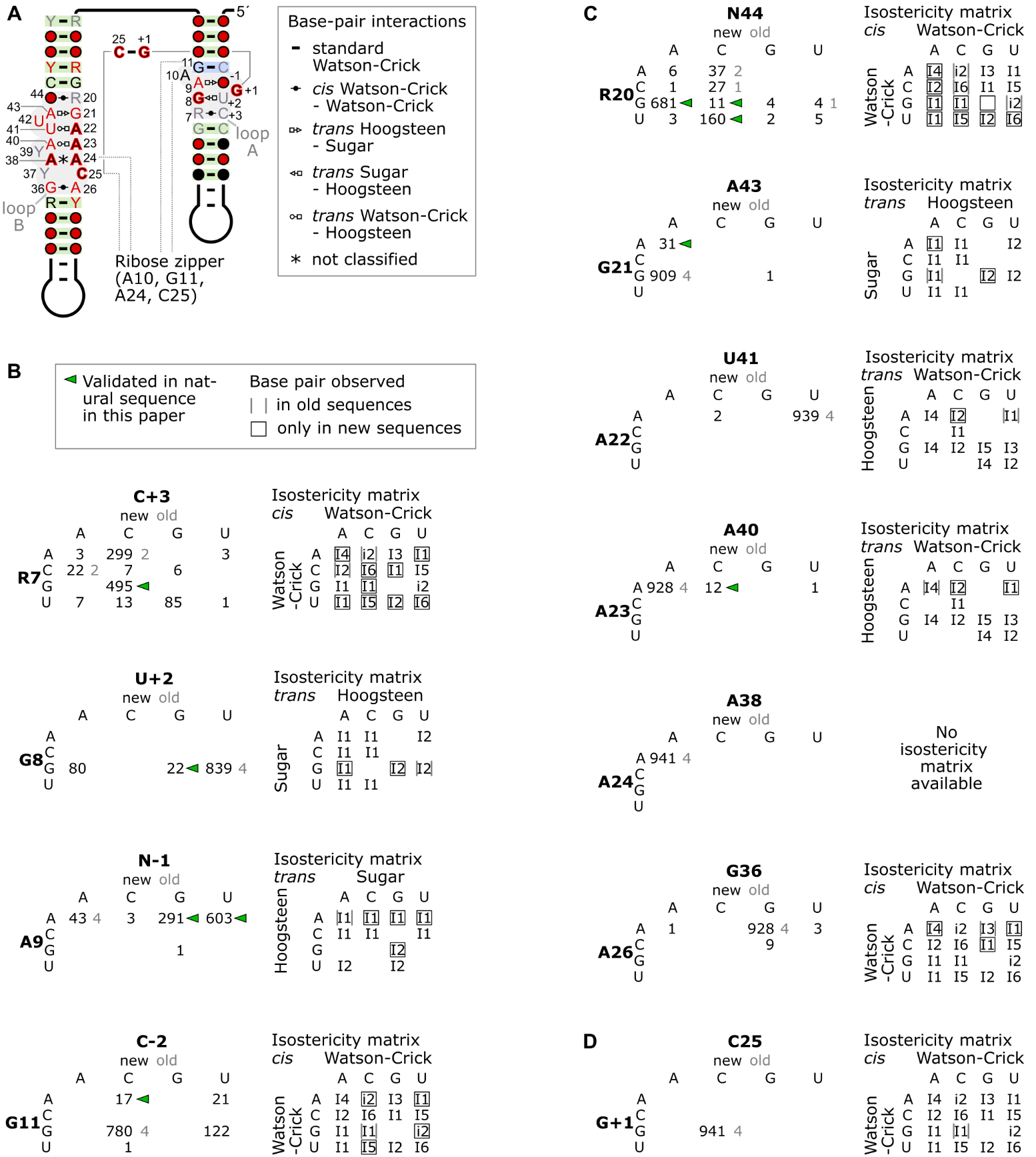
**

**Supplementary Figure 9.** Secondary structure and *in vitro* cleavage of hairpin ribozyme representatives with the natural mutations at position A10, G11 or G21. (**A**) Secondary structure diagram of hairpin ribozyme representative with the mutation A10🡪G. (**B**) Secondary structure diagram of hairpin ribozyme representative with the mutation G11🡪A. (**C**) Secondary structure diagram of hairpin ribozyme representative with the mutation G21🡪A. Red nucleotides indicate deviations from conserved positions. M1 indicates a nucleotide exchange leading to decreased ribozyme cleavage speed. Other annotations are as in Figure 1D. (**D**) Hairpin ribozyme representatives were *in vitro* transcribed in the presence of [α-^32^P]-ATP for 60 minutes. Representatives are denoted based on the nucleotide position (A10, G11 or G21) that is mutated. Bands correspond to a 3′ cleavage product of 71 nucleotides and a 5′ cleavage product of 12 nucleotides for the representative A10 (see part A). The representative G11 (see part B) cleaves itself into a 3′ cleavage product of 77 nucleotides and a 5′ cleavage product of 12 nucleotides. The 5′ cleavage product of G21 (see part C) has a size of 82 nucleotides and the 3′ cleavage product has a size of 12 nucleotides. Reactions were separated by 15% denaturing PAGE. Reduction in cleavage speed for M1 is not apparent for the sample taken after 60 minutes of incubation. The difference in cleavage speed between WT and M1 might only be detectable for this example at shorter incubation times for this representative. (**E**) A 10% denaturing PAGE was used for better separation of the full-length transcript (FL) from the 3′ cleavage product. 5′-radioactively labeled G21 M1 RNA was used to create size standards by partial digestion with RNase T1 or partial alkaline hydrolysis (“ˉOH“).


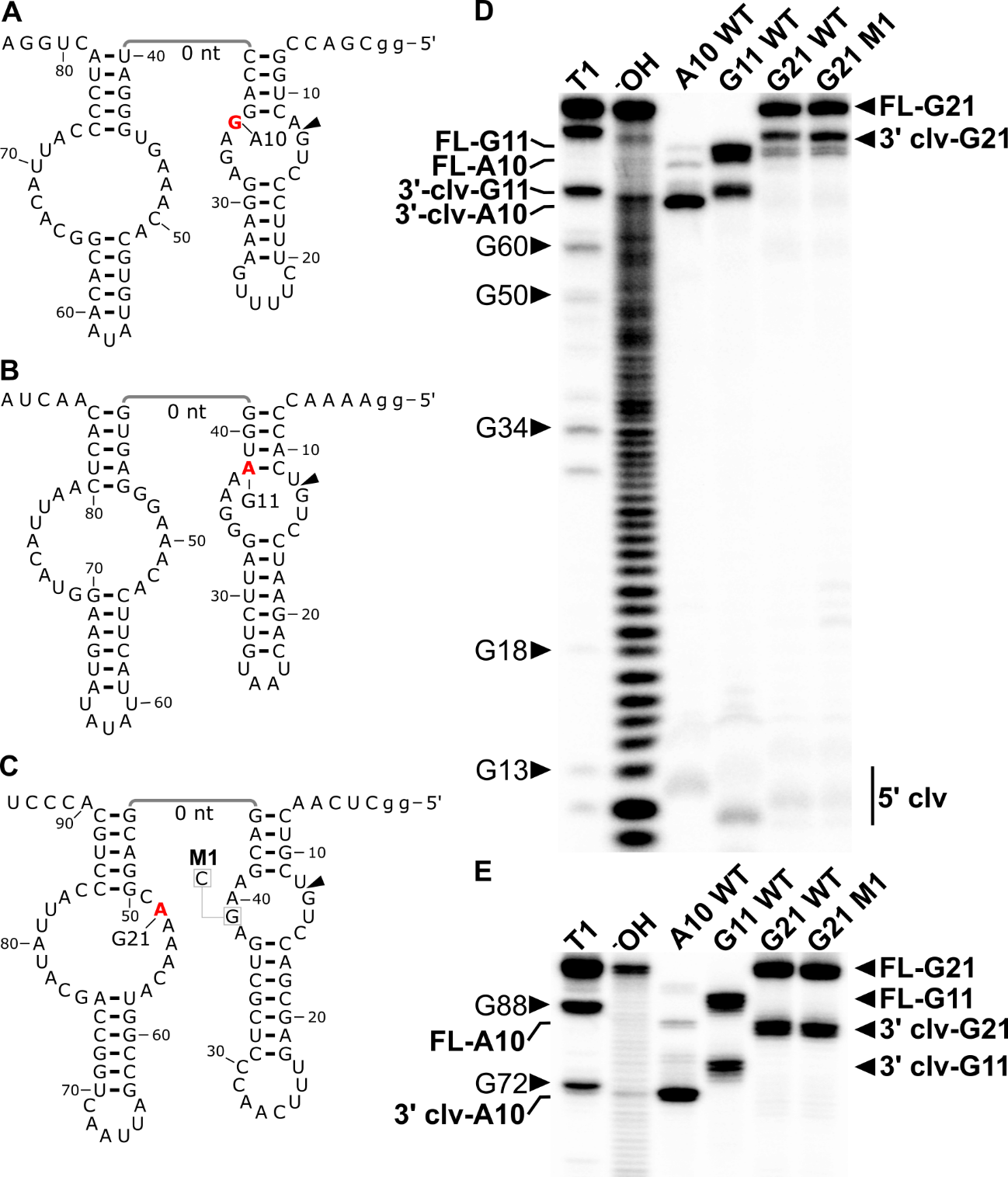


**Supplementary Figure 10.** Secondary structure and *in vitro* cleavage of hairpin ribozyme representatives L1 and L2, which contain unusual lengths of helices 2 or 3, according to our predictions. (**A**) Secondary structure diagram of hairpin ribozyme representative L1. Red nucleotides participate in extra base pairs, or are unpaired nucleotides within the junction. M1 indicates a nucleotide exchange leading to decreased ribozyme cleavage speed. Other annotations are as in Figure 1D. (**B**) Secondary structure diagram of hairpin ribozyme representative L2. (**C**) Hairpin ribozyme representatives L1 and L2 were *in vitro* transcribed in the presence of [α-^32^P]-ATP for 60 minutes. Bands correspond to a 3′ cleavage product of 127 nucleotides and a 5′ cleavage product of 12 nucleotides for the representative L1 or to a 3′ cleavage product of 87 nucleotides and a 5′ cleavage product of 11 nucleotides for the representative L2. Reactions were separated by 15% denaturing PAGE. (**D**) A 10% denaturing PAGE was used for better separation of the full-length transcript (FL) from the 3′ cleavage product. 5′-radioactively labeled L1 M1 RNA was used to create size standards by partial digestion with RNase T1 or partial alkaline hydrolysis (“^-^OH“).


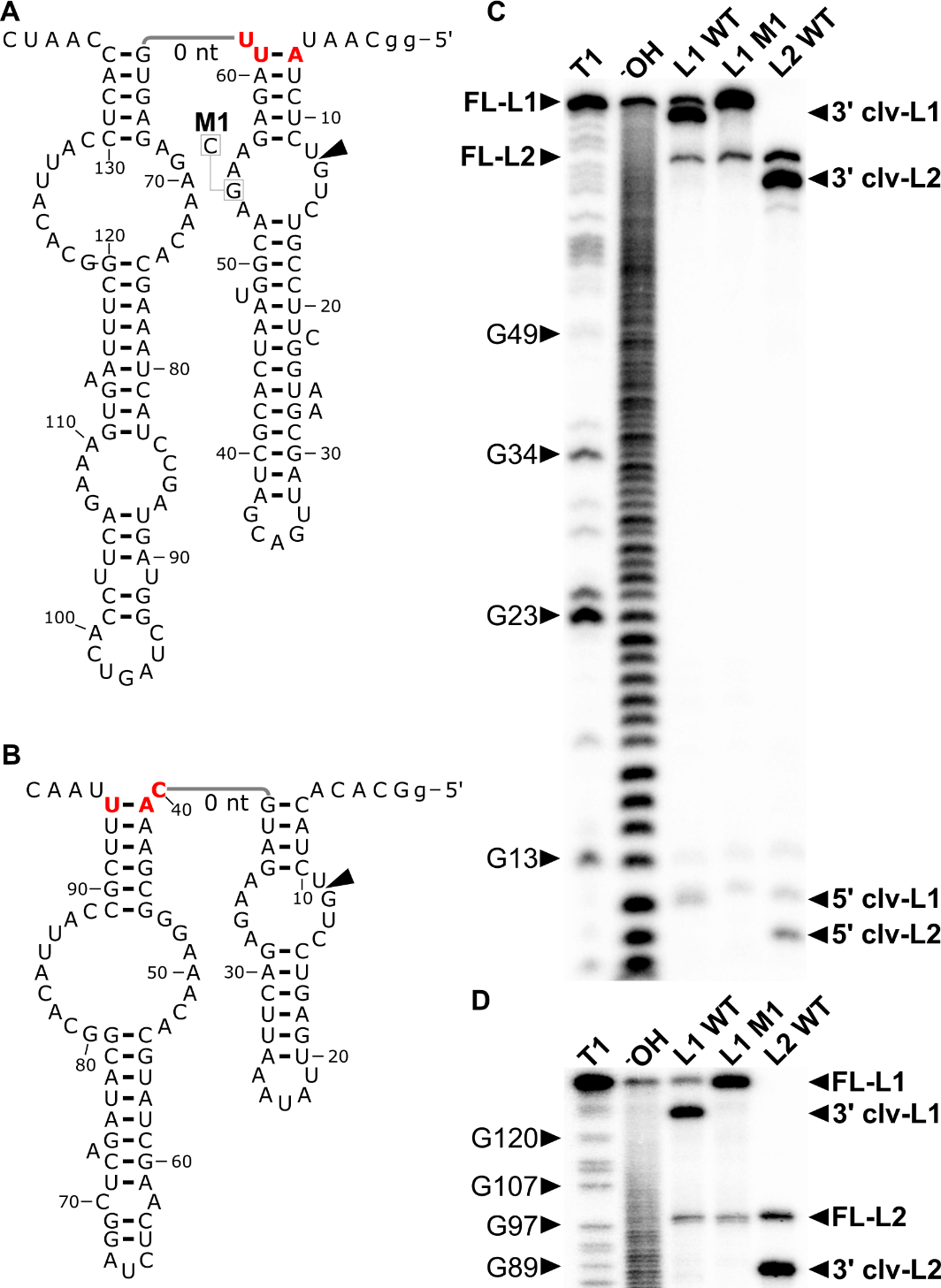


**Supplementary Figure 11.** Secondary structure and *in vitro* cleavage of a hairpin ribozyme found in contig with sequence accession Ga0247519_111615. (**A**) Secondary structure diagram of hairpin ribozyme in contig with sequence accession Ga0247519_111615. Helix 2 was extended by one base pair based on adjacent A and U nucleotides. M1 indicates a nucleotide exchange leading to a ribozyme variant with decreased cleavage speed. Other annotations are as in Figure 1D. (**B**) Hairpin ribozyme in contig was *in vitro* transcribed in the presence of [α-^32^P]-ATP for 60 minutes. Bands correspond to a 3′ cleavage product of 104 nucleotides and a 5′ cleavage product of 11 nucleotides. Reactions were separated by 15% denaturing PAGE. 5′-radioactively labeled M1 RNA was used to create size standards by partial digestion with RNase T1 (“T1”) or partial alkaline hydrolysis (“ˉOH“). (**C**) A 10% denaturing PAGE was used for better separation of the full-length transcript (FL) from the 3′ cleavage product.


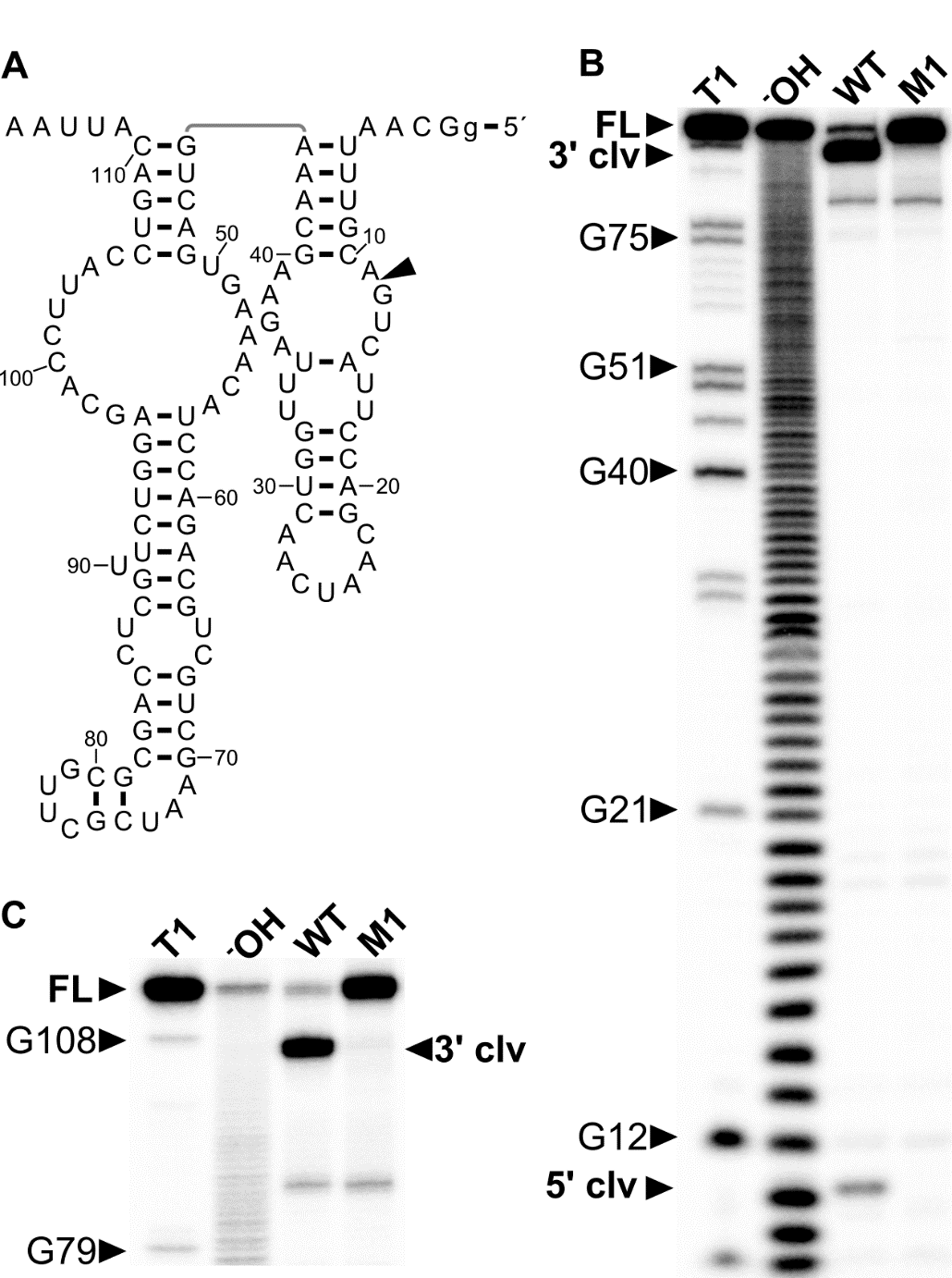


**Supplementary Figure 12.** Statistics of input alignments for RNAcode for small (<800-nucleotide), medium (800-1800-nucleotide) and large (>4000-nucleotide) contigs. (**A**) 2-D histograms depicting the number of input alignments with different ranges of two relevant statistics: (1) the number of sequences in the alignment and (2) the average percent identity of the alignment. This plot uses only large contigs, i.e. contigs whose inferred circular ssRNA has more than 4000 nucleotides. The logarithm to the base 2 of the number of sequences is depicted on the X-axis. All alignments had fewer than 2^8^=256 sequences. The Y-axis depicts the percent identity, calculated by the esl-alistat program in the Infernal (10) version 1.1.2 software package. The numbers of corresponding alignments are written in each cell, and colors are chosen according to the color bar in part **F**. Note that all cells with more than 100 sequences are depicted in the same light gray color, and cells with zero alignments are black. The definitions and scales of the X- and Y-axes are the same in subparts **B-E** of this figure. (**B**) The same information as in part **A**, but for medium contigs (800-1800 nucleotides). (**C**) The same information as in part **A**, but for small contigs (fewer than 800 nucleotides). (**D**) Alignments from large contigs that led to RNAcode E-values lower (i.e., better) than 10^-7^ are depicted like in part **A**. The histogram thus shows that a high proportion of alignments with %ID values lower than 70 and with 2^2^=4 or more sequences were classified as statistically significant by RNAcode. (**E**) The same information as in part **D**, but for medium contigs with RNAcode E-values lower (better) than 10^-7^. No small contigs led to a significant E-value, despite the presence of several alignments with properties similar to those of large contigs that were classified as likely coding, as shown in part **C**. (**F**) The color scale used in parts **A**-**E**.


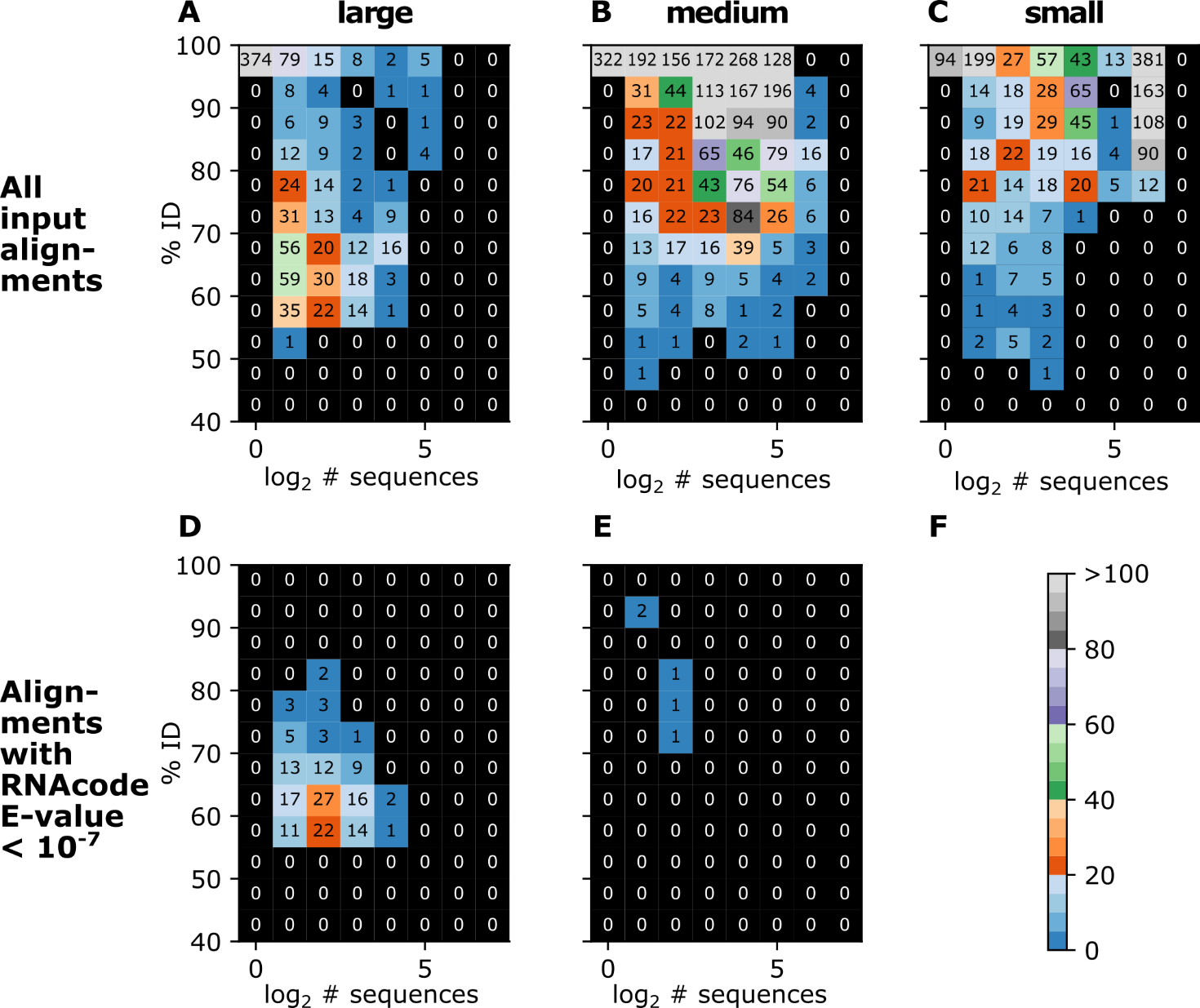


References

1. Washietl, S., Findeiß, S., Müller, S.A., Kalkhof, S., Bergen, M. von, Hofacker, I.L., Stadler, P.F. and Goldman, N. (2011) RNAcode: robust discrimination of coding and noncoding regions in comparative sequence data, *RNA,* **17,** 578–594.

2. Nunes da Rocha, U., Cadillo-Quiroz, H., Karaoz, U., Rajeev, L., Klitgord, N., Dunn, S., Truong, V., Buenrostro, M., Bowen, B.P. and Garcia-Pichel, F. *et al.* (2015) Isolation of a significant fraction of non-phototroph diversity from a desert Biological Soil Crust, *Front. Microbiol.,* **6,** 277.

3. Taylor, J.M. (2006) Structure and replication of hepatitis delta virus RNA, *Curr. Top. Microbiol. Immunol.,* **307,** 1–23.

4. Kennell, J.C., Saville, B.J., Mohr, S., Kuiper, M.T., Sabourin, J.R., Collins, R.A. and Lambowitz, A.M. (1995) The VS catalytic RNA replicates by reverse transcription as a satellite of a retroplasmid, *Genes Dev.,* **9,** 294–303.

5. Li, C.-X., Shi, M., Tian, J.-H., Lin, X.-D., Kang, Y.-J., Chen, L.-J., Qin, X.-C., Xu, J., Holmes, E.C. and Zhang, Y.-Z. (2015) Unprecedented genomic diversity of RNA viruses in arthropods reveals the ancestry of negative-sense RNA viruses, *eLife,* **4,** e05378.

6. O'Leary, N.A., Wright, M.W., Brister, J.R., Ciufo, S., Haddad, D., McVeigh, R., Rajput, B., Robbertse, B., Smith-White, B. and Ako-Adjei, D. *et al.* (2016) Reference sequence (RefSeq) database at NCBI: current status, taxonomic expansion, and functional annotation, *Nucleic Acids Res.,* **44,** D733-D745.

7. Altschul, S.F., Madden, T.L., Schaffer, A.A., Zhang, J., Zhang, Z., Miller, W. and Lipman, D.J. (1997) Gapped BLAST and PSI-BLAST: a new generation of protein database search programs, *Nucleic Acids Res.,* **25,** 3389–3402.

8. Chen, I.-M.A., Chu, K., Palaniappan, K., Pillay, M., Ratner, A., Huang, J., Huntemann, M., Varghese, N., White, J.R. and Seshadri, R. *et al.* (2019) IMG/M v.5.0. An integrated data management and comparative analysis system for microbial genomes and microbiomes, *Nucleic Acids Res.,* **47,** D666-D677.

9. Bajaj, P., Steger, G. and Hammann, C. (2011) Sequence elements outside the catalytic core of natural hairpin ribozymes modulate the reactions differentially, *Biol. Chem.,* **392,** 593–600.

10. Nawrocki, E.P. and Eddy, S.R. (2013) Infernal 1.1: 100-fold faster RNA homology searches, *Bioinformatics,* **29,** 2933–2935.

11. Jimenez, R.M., Polanco, J.A. and Lupták, A. (2015) Chemistry and biology of self-cleaving ribozymes, *Trends Biochem. Sci.,* **40,** 648–661.

12. Lee, K.-Y. and Lee, B.-J. (2017) Structural and biochemical properties of novel self-cleaving ribozymes, *Molecules,* **22**.

13. Weinberg, C.E., Weinberg, Z. and Hammann, C. (2019) Novel ribozymes. Discovery, catalytic mechanisms, and the quest to understand biological function, *Nucleic Acids Res.,* **47,** 9480–9494.

14. Macke, T.J., Ecker, D.J., Gutell, R.R., Gautheret, D., Case, D.A. and Sampath, R. (2001) RNAMotif, an RNA secondary structure definition and search algorithm, *Nucleic Acids Res.,* **29,** 4724–4735.

15. Zytnicki, M., Gaspin, C. and Schiex, T. (2008) DARN! A weighted constraint solver for RNA motif localization, *Constraints,* **13,** 91–109.

16. Eckert, I. and Weinberg, Z. (2020) Discovery of 20 novel ribosomal leader candidates in bacteria and archaea, *BMC Microbiol.,* **20,** 130.

17. Webb, C.H., Riccitelli, N.J., Ruminski, D.J. and Lupták, A. (2009) Widespread occurrence of self-cleaving ribozymes, *Science,* **326,** 953.

18. Weinberg, Z., Kim, P.B., Chen, T.H., Li, S., Harris, K.A., Lünse, C.E. and Breaker, R.R. (2015) New classes of self-cleaving ribozymes revealed by comparative genomics analysis, *Nat. Chem. Biol.,* **11,** 606–610.

19. Pinard, R., Hampel, K.J., Heckman, J.E., Lambert, D., Chan, P.A., Major, F. and Burke, J.M. (2001) Functional involvement of G8 in the hairpin ribozyme cleavage mechanism, *EMBO J.,* **20,** 6434–6442.

20. Leontis, N.B., Stombaugh, J. and Westhof, E. (2002) The non-Watson-Crick base pairs and their associated isostericity matrices, *Nucleic Acids Res.,* **30,** 3497–3531.

21. Rupert, P.B. and Ferre-D'Amare, A.R. (2001) Crystal structure of a hairpin ribozyme-inhibitor complex with implications for catalysis, *Nature,* 780–786.

22. Laing, C. and Schlick, T. (2009) Analysis of four-way junctions in RNA structures, *J. Mol. Biol.,* **390,** 547–559.

23. Reymond, C., Beaudoin, J.-D. and Perreault, J.-P. (2009) Modulating RNA structure and catalysis: lessons from small cleaving ribozymes, *Cell. Mol. Life Sci.,* **66,** 3937–3950.
