# Supplementary material for: Identification of over 200-fold more hairpin ribozymes than previously known in diverse circular RNAs
Source: Nucleic Acids Res. 2021 Jun 7;49(11):6375–88. doi: 10.1093/nar/gkab454 (PMC8216279; doi:10.1093/nar/gkab454)
Supplement: gkab454_Supplemental_Files [file gkab454_supplemental_files.zip › Supplementary-File-3.pdf]

Supplementary File 3
(printable multiple-sequence alignments of hairpin ribozymes)
Identification of over 200-fold more hairpin ribozymes than previously known in diverse circular RNAs

Christina E. Weinberg, V. Janett Olzog, Iris Eckert & Zasha Weinberg

Note:

- The various alignments presented here are explained in Supplementary Table 2.
- Machine-readable versions of these alignments are available in Stockholm format in Supplementary File 2.
- The presentation and explanatory text of this supplementary data on novel RNA motifs follows the pattern of a presentation of previously found conserved RNA motifs (Weinberg, *et al.*, 2010).

Contents

1 hairpin-touched-up-initial **3**
1.1 Multiple-sequence alignment . . . 3

2 hairpin-initial **8**
2.1 Multiple-sequence alignment . . . 8

3 hairpin-extra-spruce-initial **12**
3.1 Multiple-sequence alignment . . . 12

4 hairpin-stringent **66**
4.1 Multiple-sequence alignment . . . 66

5 hairpin-extra-spruce-stringent **68**
5.1 Multiple-sequence alignment . . . 68

6 hairpin-second-candidate **129**
6.1 Multiple-sequence alignment . . . 129

7 hairpin-TSA **132**
7.1 Multiple-sequence alignment . . . 133

8 hairpin-previously-published-four **136**
8.1 Notes . . . 136
8.2 Multiple-sequence alignment . . . 136

1 hairpin-touched-up-initial

For a description of this alignment, see Supplementary Table 2

1.1 Multiple-sequence alignment

Each ribozyme in this alignment is identified by its genomic location in the form SEQID/START-END. SEQID (the sequence accession) is derived from sources such as IMG/M or GenBank. START is the coordinate of the $5'$ nucleotide of the hairpin ribozyme, and END corresponds to the $3'$ nucleotide. If START>END, then the ribozyme is on the reverse complement strand.

Nucleotides proposed to basepair as part of the consensus structure are shaded in color when they comprise Watson-Crick or G-U pairs. Otherwise they are shaded gray. Conserved stems are also indicated at the bottom of the alignment by angle brackets, where matching $<$ and $>$ denote base-paired columns. Below these angle brackets, the symbol "2" denotes base pairs exhibiting covariation according to the statistically well-founded R-scape method. "1" denotes base pairs exhibiting covariation according to R2R's simplistic method. "0" denotes base pairs that are not observed to mutate and "?" denotes base pairs that have a significant frequency of non-canonical nucleotides for Watson-Crick or G-U pairs ($> 5\%$). Below these base pair annotation is the consensus sequence: "R" = "A" or "G", "Y" = "C" or "U", red nucleotides: nucleotide identity conserved more than 97% of the time, black nucleotides: 90%, gray nucleotides: 75%, red circle (•): nucleotide is present 97% of the time, black circle (•): 90%, gray circle (•): 75%, white circle (◦): 50%. All percentages of sequences just described (e.g. 97% conserved) assume that sequences have been weighted by the GSC algorithm implemented by the Infernal software package.

The alignment begins on the next page.

alignment positions 1 · · · 134

```
Ga0257140_1130477/77-287           CAUUUGAGUUGUUAUAACUUCUUC..................AUGGAUAAAGCAUACUCUCA.UAUCGGC....................UUAA........................................
Ga0247516_125012/316-428           CUUCUGUCCGUCAAA.......................................................................................................................
Ga0247529_117158/253-331           AGUCUGUCCCAG..........................................................................................................................
Ga0179947_1156513/270-495          CAACUGUCCACGGAGGAUGUGUAUAGGAAAGAGAUCAUCUAUACAAA...CCUGA..............................GCGCUUAAAUU...AGCGUACGAAGUUUUACAC.UCGUUCUCGUUUAAA
Ga0247519_105128/410-263           GGUCUGUCCUUUGGCAG.AG......................UGGA....CUUUCGG.................................CUUGAUCGU...................................
Ga0257141_1138773/402-200          GUGCGGUCCUAGCAUC......................................................................................................................
Ga0247527_109126/592-695           UUGCAGUCAUUCCAG.......................................................................................................................
Ga0247519_111615/148-45            UUGCAGUCAUUCCAG.......................................................................................................................
Ga0247513_116034/391-494           UUGCAGUCAUUCCAG.......................................................................................................................
Ga0257136_1044348/475-684          CAUUUGAGUUGUUAUAACUUCUUC..................AUGGAUA.AAGCAUACUCUCA.UAUCGGC...................UUAA........................................
Ga0247529_134913/224-64            AAUCUGUCCCUACUUCG.................................GGGGU...................................GCCAA.......................................
Ga0247519_110701/607-447           AAUCUGUCCCUACUUCG.................................GGGGU...................................GCCAA.......................................
Ga0247530_109035/489-319           CUUCUGUCUCAGUCAAG.........................GG......UUCC....................................CAAGCCCAU...................................
Ga0247526_105246/689-508           UCUCUGUCUCUGAUUCG.................................GGUUU...................................UGCUA.......................................
Ga0247515_107373/548-731           UCUCUGUCUCUGAUUCG.................................GGAUU...................................UGCUA.......................................
JGI12272J11983_1072695/645-539     CGAUUGAGCUGGG.........................................................................................................................
Ga0257141_1118781/153-31           AGUCUGUCCUG.AUGCU.....................................................................................................................
Ga0247522_108409/684-554           GUCCUGUCCUCUAGG.......................................................................................................................
Ga0257141_1100114/291-412          AGUCUGUCCUG.ACG.......................................................................................................................
Ga0179955_1013331/439-311          CGUCUGUCUCGAC.........................................................................................................................
Ga0247519_101377/1717-1907         GAGUUGUCCGUGUGGAG.........................UU......GACCACAAG...............................CCUA........................................
Ga0247512_104440/911-1082          CUUCUGUCCCCAUCAC..........................UA......UGUUCUGCAUCUG...........................CUUU........................................
Ga0179944_1246019/249-162          UCAUUGUCCUAAGACU......................................................................................................................
Ga0255059_10691632/166-76          UAUCAGUGUGUGG.........................................................................................................................
Ga0255062_11230321/200-110         UAUCAGUGUGUGG.........................................................................................................................
Ga0247525_114510/199-318           UGUCUGUCCCGAAC............................UA......CAU.....................................CAGGGU......................................
JGI12272J11983_1345183/364-258     UUUCUGUCCCACAGUCC.........................G.......UGC.....................................GUAAA.......................................
Ga0247521_112559/317-205           UCGCUGUGGUUUAUG.......................................................................................................................
JGI12272J11983_1072695/40-150      UCUUUGUCCUCGC.........................................................................................................................
Ga0247513_128543/241-71            CUUCUGUCUCGGUCAA..................................GGGUU...................................CCCA........................................
Ga0247528_108647/438-562           AUACUGUCUGAUU.........................................................................................................................
Ga0247517_121471/387-499           CAGCUGUGGUUGGAUG......................................................................................................................
Ga0247515_120448/91-178            CAGCUGUCCU............................................................................................................................
Ga0247518_120275/202-289           CAGCUGUCCU............................................................................................................................
Ga0247526_117235/152-313           UUUCUGUCCCUACUUCG.................................GGGGU...................................GCCAA.......................................
Ga0179947_1148619/378-303          UAAUUGAUC..CUCU.......................................................................................................................
Ga0179947_1156513/31-105           AGAUUGAUC..CUCU.......................................................................................................................
Ga0247519_107918/217-58            AAUCUGUCCCUACUUCG.................................GGGGU...................................GCCAA.......................................
Ga0179944_1246019/41-131           CCAUUGUGUCUACC........................................................................................................................
Ga0247529_103231/270-430           AAUCUGUCCCUACUUCG.................................GGGGU...................................GCCAA.......................................
Ga0247526_110221/603-443           AAUCUGUCCCUACUUCG.................................GGGGU...................................GCCAA.......................................
Ga0179944_1119724/70-159           CGGUUGGGAGAG.CUCU.....................................................................................................................
Ga0247521_125910/353-214           UGACGGUACUCA..GAC.....................................................................................................................
Ga0247524_111427/50-210            AAUCUGUCCCUACUUCG.................................GGGGU...................................GCCAA.......................................
Ga0247527_115808/280-440           CGUCUGUCCCUACUUCG.................................GGGGU...................................GCCAA.......................................
Ga0247526_101949/462-575           GGACUGGACCAGGGCUC.....................................................................................................................
Ga0247524_101842/357-244           GGACUGGACCAAGGCUC.....................................................................................................................
JGI12272J11983_1356304/1548-1654   CUGUUGAGCUGGG.........................................................................................................................
Ga0257141_1089020/433-224          CAUUUGAGUUGUUAU...........................AACUUCUUCAUGGAUAAAGCAUACUCUCA.UAUCGGC...........UUAA........................................
Ga0247519_108765/580-693           GGACUGGACCAAGGC.......................................................................................................................
Ga0257136_1059838/549-429          AGUCUGUCCUG.AUGCU.....................................................................................................................
Ga0247518_108423/758-837           AAGCGGUCCGU...........................................................................................................................
Ga0247519_108375/286-206           UAGCGGUCCAGGGGU.......................................................................................................................
Ga0247521_124040/388-309           AAGCUGUCCGU...........................................................................................................................
Ga0247518_137824/301-231           GUGCGGUCCCAUC.........................................................................................................................
Ga0247531_104861/1082-1012         GUGCGGUCCCAUC.........................................................................................................................
Ga0247523_120120/325-403           UAGCGGUCCGCAUGUG......................................................................................................................
Ga0247526_116548/279-354           AUGCGGUCGGAGGAU.......................................................................................................................
Ga0247520_104849/891-821           GUACGGUCCCAUC.........................................................................................................................
Ga0247529_111070/303-228           AUGCGGUCGGGAGAU.......................................................................................................................
Ga0247516_124175/202-272           UAGCGGUCUACAC.........................................................................................................................
Ga0247519_113845/334-409           AUGCGGUCGGGAGAU.......................................................................................................................
Ga0247519_118271/334-405           UAGCGGUCCGG...........................................................................................................................
Ga0179955_1048341/123-51           GGUCGGUCCGGU..........................................................................................................................
Ga0247524_126530/125-198           UUACGGUCCAUCC.........................................................................................................................
Ga0247514_114593/280-211           AGGCAGUCCCUC..........................................................................................................................
Ga0247527_116942/161-88            AAGCGGGGCGGCA.........................................................................................................................
Ga0247514_107497/1071-1140         GAGCAGUCCCUC..........................................................................................................................
Ga0179944_1005311/261-382          GGUCGGUCCUUCCGG.......................................................................................................................
Ga0179934_1155244/114-26           GGAUGGUCCG............................................................................................................................
Ga0179955_1195671/481-563          AUGCGGUAUGCAUCA.......................................................................................................................
Ga0179948_1254202/198-296          AGUCGGUCCACAC.........................................................................................................................
Ga0179944_1204933/216-89           GGUCAGUCCUUCAGG.......................................................................................................................
Ga0247529_116306/255-362           GCGCUGUCCUGGCAC.......................................................................................................................
Ga0179944_1191552/284-411          CGUCGGUCCAUCGGG.......................................................................................................................
Ga0179944_1313268/129-232          GUUCGGUCCUC..GA.......................................................................................................................
Ga0247526_117664/434-499           CAGCGGUCCAUG..........................................................................................................................
JGI12272J11983_1319142/230-159     GUGCGGUCGGGGG.........................................................................................................................
Ga0247531_104608/1256-1177         AAGCGGUCCGU...........................................................................................................................
Ga0247519_104852/800-730           GUGCGGUCCGG...........................................................................................................................
Ga0247524_105844/561-637           UAGUGGUCCAGA..........................................................................................................................
Ga0247524_121352/374-303           UAGCGGUCCGG...........................................................................................................................
Ga0247519_103291/1381-1460         CUGCGGUCGGGA..........................................................................................................................
Ga0179934_1197833/207-115          AGUCGGUCCACAG.........................................................................................................................
                                   <<<<....<<<<<<<<<<<<<<<<<<<<<<<<<<<<<<<<<<........<<<<<<<<<<<<<<<<<<<<<<<<<<<<<<<<<<<<<<<<.........>>>>><<<<<<<.....>>>>>>>...........
                                   2221....22222221..111...0000..00..000.0000........1111....1.....11.1....0000000......00000.........000000000.00.....00.0000...........
                                   ●●●C●GUYY●●●●○○-----------------------------------------------------------------------------------------------------------------------
```

alignment positions 135 · · · 281

```
Ga0257140_1130477/77-287           ...............GCCGGUGGCAUGGGUACGUUUAAACA...............................UGUG.UAG....AGUGCAA...AGGUUCCUU......AAAA..................................
Ga0247516_125012/316-428           ...................................AUGAA................................GCACCU...............................AGGGUUCAA.............................
Ga0247529_117158/253-331           .............................................................................................................GCAAUCAAA.............................
Ga0179947_1156513/270-495          ..............................UCAGGAA...................................UCAAGCGGAUUGUUUAUA...................UUA...................................
Ga0247519_105128/410-263           ............................CCGAGAG.....................................GGGGCC...............................AAGU..................................
Ga0257141_1138773/402-200          ...................................CAA..................................ACACACACGGGCCAAAACAACCCAACAGUGACAACCAGAAAA.................................
Ga0247527_109126/592-695           .............................................................................................................CAUUUUA...............................
Ga0247519_111615/148-45            .............................................................................................................CAAUCAA...............................
Ga0247513_116034/391-494           .............................................................................................................CAAUCAA...............................
Ga0257136_1044348/475-684          ..............GCCGGUGGCAUGGGUACGUUUAAACA................................UGU.GUAG....AGUGCAGA...GGUUCCU.......UAAAA.................................
Ga0247529_134913/224-64            ..............................ACCCCAAGCACA..............................AGCUGA.........CCGUCAU...............AUGUG.................................
Ga0247519_110701/607-447           ..............................ACCCCAAGCACA..............................AGCUGA.........CCGUCAU...............AUGUG.................................
Ga0247530_109035/489-319           ...............................GGAACA...................................GG.UAUCU.CGGUUAAUGGUCAUCACA..........CGGCCUCGUGCAA.........................
Ga0247526_105246/689-508           ..............................AAACCCAAGGCAUU............................GGUUCAGUGUGU...CGUUGCUCGGGCCUUUCUUACCUAAAC.................................
Ga0247515_107373/548-731           ..............................AAUCCCAAGGCAUU............................GGUUCA.GUGUGUCGU.UGCUCAGGCCUUUC..UACCUUAGU.................................
JGI12272J11983_1072695/645-539     ...................................AUAACUG..............................UGGAUUGCAUC..........................UAUGCU................................
Ga0257141_1118781/153-31           ........................................................................CGGU.................................UGCUA.................................
Ga0247522_108409/684-554           ...................................AUGAA................................GCACUAGGCGAGGUU.GCGGA................AAGAC.................................
Ga0257141_1100114/291-412          ...................................C....................................UCGGC................................UGCUA.................................
Ga0179955_1013331/439-311          ...................................AAC..................................GUCUC................................ACUCAAAUA.............................
Ga0247519_101377/1717-1907         ..........................UUCGUGGUCU........GUAAGGUGGCUGAAAUAAUGCCUUACGACCCGCCGUUGACUAAAGUUG..CCUC...........UUCG..................................
Ga0247512_104440/911-1082          ......................CA.AUGCAAAACA.....................................GGC..................................GUCU..................................
Ga0179944_1246019/249-162          .............................................................................................................UGGA..................................
Ga0255059_10691632/166-76          ...................................AA...................................GAGA.................................UGAGC.................................
Ga0255062_11230321/200-110         ...................................AA...................................GAGA.................................UGAGC.................................
Ga0247525_114510/199-318           ................................AUG.....................................CAACAGGACCUUCC.......................UUCAC.................................
JGI12272J11983_1345183/364-258     ................................GCA...................................AUGCC..................................AAGC..................................
Ga0247521_112559/317-205           ...................................GUAAA................................GCC..................................UUAAUU................................
JGI12272J11983_1072695/40-150      ........................................................................AUCCGU......GUGCGU...................AUA...................................
Ga0247513_128543/241-71            ..............................AGCCCAUGGAACA.............................GG.UAUCU.CGGUUAAUGGUCAUCACA..........CGGCCUCGUGCAA.........................
Ga0247528_108647/438-562           ...................................UC...................................GUAUGG...............................UUUGUAUU..............................
Ga0247517_121471/387-499           ...................................GUAAA................................GCC..................................UUUAGU................................
Ga0247515_120448/91-178            .............................................................................................................UAACU.................................
Ga0247518_120275/202-289           .............................................................................................................UAACU.................................
Ga0247526_117235/152-313           ..............................ACCCCAAGCACA..............................AGCUGA........ACCGCGAU...............AUGCG.................................
Ga0179947_1148619/378-303          .............................................................................................................ACAUUG................................
Ga0179947_1156513/31-105           .............................................................................................................ACAUU.................................
Ga0247519_107918/217-58            ..............................ACCCCAAGCACA..............................AGCUGA........ACUGCGAU...............AUGAG.................................
Ga0179944_1246019/41-131           .............................................................................................................GUAA..................................
Ga0247529_103231/270-430           ..............................ACCCCAAGCACA..............................AGCUGA........ACUGCGAU...............AUGAG.................................
Ga0247526_110221/603-443           ..............................ACCCCAAGCACA..............................AGCUGA.........CCGCCAU...............AUGUG.................................
Ga0179944_1119724/70-159           .............................................................................................................ACUCACAUA.............................
Ga0247521_125910/353-214           ...................................C....................................GGUUUCGUCUACCCACCUC..................ACUCUUUUUACAA.........................
Ga0247524_111427/50-210            ..............................ACCCCAAGCACA..............................AGCUGA.........CCGUCAU...............AUGUG.................................
Ga0247527_115808/280-440           ..............................ACCCCAAGCACA..............................AGCUGC.........CAU.CCAU..............AUGUG.................................
Ga0247526_101949/462-575           ...................................AAUU.................................AGGCUGGAUCUUG........................CAUCC.................................
Ga0247524_101842/357-244           ...................................AUUC.................................AGGUUGGAUCUUG........................CAUCC.................................
JGI12272J11983_1356304/1548-1654   ...................................ACAACCG..............................UGGAUUGCAUCG.........................UCGC..................................
Ga0257141_1089020/433-224          ......GCCGGUGGCAUGGGUACGUUUAAA.CAUG.....................................UGUAG.AGU............................GCAGAGGUUCCUUAAAAAGGUUUCAAACUUGGAUUUAA
Ga0247519_108765/580-693           ...................................UCA..................................UUCAGGU........UGGAU.................CUUGC.................................
Ga0257136_1059838/549-429          ........................................................................GGU..................................UGCUA.................................
Ga0247518_108423/758-837           ...................................U....................................AUGUC................................CAUCA.................................
Ga0247519_108375/286-206           ........................................................................AUAG.................................UAGA..................................
Ga0247521_124040/388-309           ...................................U....................................AUGUC................................CAUCA.................................
Ga0247518_137824/301-231           .............................................................................................................UUCG..................................
Ga0247531_104861/1082-1012         .............................................................................................................UCCG..................................
Ga0247523_120120/325-403           .............................................................................................................UAAUC.................................
Ga0247526_116548/279-354           .............................................................................................................CCUAG.................................
Ga0247520_104849/891-821           .............................................................................................................UCCG..................................
Ga0247529_111070/303-228           .............................................................................................................CUUAG.................................
Ga0247516_124175/202-272           .............................................................................................................UUAU..................................
Ga0247519_113845/334-409           .............................................................................................................CUUAG.................................
Ga0247519_118271/334-405           .............................................................................................................GACAUUA...............................
Ga0179955_1048341/123-51           .............................................................................................................UACAU.................................
Ga0247524_126530/125-198           .............................................................................................................GAUGA.................................
Ga0247514_114593/280-211           .............................................................................................................UUUUU.................................
Ga0247527_116942/161-88            .............................................................................................................UGCA..................................
Ga0247514_107497/1071-1140         .............................................................................................................UACG..................................
Ga0179944_1005311/261-382          ...................................G....................................CAUCACAG.............................CGCAUGA...............................
Ga0179934_1155244/114-26           ...................................AAG..................................GC...................................AGAA..................................
Ga0179955_1195671/481-563          .............................................................................................................UAA...................................
Ga0179948_1254202/198-296          .............................................................................................................ACGUACUAAUA...........................
Ga0179944_1204933/216-89           ...................................G....................................CAUCAUUGCU...........................UACAC.................................
Ga0247529_116306/255-362           ...................................UUUU.................................GACCUUCGAU...........................ACGG..................................
Ga0179944_1191552/284-411          ...................................G....................................CAUCACAG.............................CGCUUGA...............................
Ga0179944_1313268/129-232          ...................................UGAAAC...............................CCCU.................................GGCC..................................
Ga0247526_117664/434-499           .............................................................................................................UUAU..................................
JGI12272J11983_1319142/230-159     .............................................................................................................ACCCC.................................
Ga0247531_104608/1256-1177         ...................................U....................................AUGUC................................CAUCA.................................
Ga0247519_104852/800-730           ........................................................................CCC..................................AUCC..................................
Ga0247524_105844/561-637           ...................................CCA..................................GU...................................GAAU..................................
Ga0247524_121352/374-303           ...................................A....................................UC...................................AUCU..................................
Ga0247519_103291/1381-1460         ...................................U....................................GUUGAC...............................UUAU..................................
Ga0179934_1197833/207-115          ...................................AAUCU................................UUGGC..CAUC..........................AUCA..................................
                                   >>>>>>>>>>>>>>>>>>>>>>>>>>>>>>>>>>>.........<<<<<<<<..........>>>>>>>>..<<<<<<<<<<<<<<<<<<<<<<<<<<<<<<<<<<<<<......................................
                                   ......0000000....1.11.....1....1111.........00000000..........00000000..22221..................11..11.....101......................................
                                   -----------------------------------o------------------------------------oooo---------------------------------●●●●o---------------------------------
```

alignment positions 282 · · · 428

```
Ga0257140_1130477/77-287          ......AAGGUUUCAAACUUGCAUUUAAACUACUACAAAC.................................GAAGAA...AUAACAAUGAAGAUG..ACCAGGGAAACAUGACGUACA...........................
Ga0247516_125012/316-428          ...............................AGGUGUGGG..........................................UUUGACGAGAAGAAUG.UCCGGUGAAACACAGCCUGGG...........................
Ga0247529_117158/253-331          .....................................................................................CUGGGGAAGACU..GAUGGCAAAACACGGGAAAA............................
Ga0179947_1156513/270-495         ...................UAUAGACACAAUGCUUGAC.................AUAGUUGAGAUUGAUCCUC..UACAUUAGAGGUGAGAAGUUG..AUGAGGGAAACAUCUCAG..............................
Ga0247519_105128/410-263          ...............................GGCUUCGCUUUGAUCUUAAAAUUG......................CUGCU.CCAAAGGGAAGACUUGAAGAGAGAAACACCUGUG..............................
Ga0257141_1138773/402-200         UGGU.GUCAUUGUUGGG.........GGAGAGUGUGU...........................................CGAUGCUAGAGAAGCAC..GCAGGUGAAACCACCGCUAUCUGUAAACAUCCAAUCCAACAGUGUGUU
Ga0247527_109126/592-695          ..................................................................................CUGGUUUAGAAGCAAA.GUCAGUGAAACAUCCAGA.CGUCGUCGAAUCG................
Ga0247519_111615/148-45           ..................................................................................CUGGUUUAGAAGCAAA.GUCAGUGAAACAUCCAGA.CGUCGUCGAAUCG................
Ga0247513_116034/391-494          ..................................................................................CUGGUUUAGAAGCAAA.GUCAGUGAAACAUCCAGA.CGUCGUCGAAUCG................
Ga0257136_1044348/475-684         .......AGGUUUCAAACUUGCAUUUAAACUACUACAAAC.................................GAAGAA...AUAACAAUGAAGAUG..ACCAGGGAAACAUGACGUACA...........................
Ga0247529_134913/224-64           ...............AUGGCGGACCACUUCCUCAGCUAC.........................................CGAAGUAGGAGAAGAAU..UCCAGGGAAACACUCCAUC....UUGGAUU..................
Ga0247519_110701/607-447          ...............AUGGCGGACCACUUCCUCAGCUAC.........................................CGGAGUAGGAGAAGAAU..UCCAGGGAAACACUCCAUC....UUGGGUU..................
Ga0247530_109035/489-319          ..........UGUGUUUGUCCUUAGCCUCAGAUCUCCAG.........................................CUUGGCUGAAGAAGAAGC.GUCAGGGAAACACGUAGG..............................
Ga0247526_105246/689-508          GGUUG.UUAGACCUGAGUGCUUUGCAC.UU..GAUCCACC........................................CGGAUCAGAAGAAGAUCU.UCCAGGGAAACACGCUUAGG............................
Ga0247515_107373/548-731          GGUG.UUUGAUACCUGAGUGCUUCUUGCACUUGAUCCACC........................................CGGAUCAGAAGAAGAUCU.UCCAGGGAAACACACUUAGG............................
JGI12272J11983_1072695/645-539    ..........................GAUGUUGGCCA...............................................CCCAGUGAGGUCU..UCCAGGGAAACACAGUGG..............................
Ga0257141_1118781/153-31          .................................ACCG...........................................AGCGUACAGAGAAGACA..CCAGAGGAAACACUCUCCAAGCUAGUAAAGAGUGAGCU..........
Ga0247522_108409/684-554          ................UCCGCUGGCUUCAG..GGUGUGUG..........................................CCUAGAGAGAAGGAUU.GCCGGUGAAACAUGCUCGC.............................
Ga0257141_1100114/291-412         ................................GUCGAA............................................CGUACAGAGAAGACA..CCAGAGGAAACACUCCC..ACGCCA......CCUUGUA..........
Ga0179955_1013331/439-311         ................................GAGACCACCAAUC.......................................GUCGAAGAAGACG..GGACAAGAAACACCGCGUAGUUUUUGAAA...CUAUA...........
Ga0247519_101377/1717-1907        ...........GGGGUUUGACUG.....GACGGUGGGCGUGC......................................CUCCAUACGGGAAGCUC..UGCAGUAAAACAUCUUCAGCG...........................
Ga0247512_104440/911-1082         ..................................GCCACU.........................................GUGGUGGGAGAGGAAGAGACCAGGGAAACAUGCUGUAGUCUAGUCUGGGUUUACGGGUAUGUA...
Ga0179944_1246019/249-162         .................................................................................AGUCUUAGGGAAGUGA..GCAAGUGAAACAUGCAGAAGUAG.........................
Ga0255059_10691632/166-76         .................................UCUCAA.............................................CCACAUGAAGAUA..GCCAGGGAAACAUACCCGGAA...........................
Ga0255062_11230321/200-110        .................................UCUCGA.............................................CCACAUGAAGAUA..GCCAGGGAAACAUACCCGGAA...........................
Ga0247525_114510/199-318          .......................GGAAGGUCCUGUUGACU...........................................GUUCGGGGAAGACA..ACCAGGGAAACACACCGG..............................
JGI12272J11983_1345183/364-258    ..................................GGCUA.........................................GGA.UGUGGCGAAGAAAU.GGGAGGGAAACACAUCAGCGU...........................
Ga0247521_112559/317-205          ..................................GGCA............................................CAUAGGCUGAAGCGA..GUGAGGGAAACAUGUAAUUGUGAGUGGUUG..................
JGI12272J11983_1072695/40-150     ...................GCGCAAUCCCAAGCGGGUAGGA...........................................GCGAGAGAAGAGAU.GGGAGGGAAACAUGCUGGAGU...........................
Ga0247513_128543/241-71           ..........UGUGUUUGUCCUUAGCCUCAGAUCUCCAGC.........................................UUGGCUGAAGAAGAAGC.GUCAGGGAAACACGUAGG..............................
Ga0247528_108647/438-562          ...............................CCAUACAU.............................................AGUCAAGAAGUAU..UCCAAGGAAACACACCACUUUGAUCAUCGUGCUAU.............
Ga0247517_121471/387-499          ..................................GGCA...........................................CAUUCGGCUGAAGCUG..GUGAGGGAAACAUAGUGUUGUGGCUAGAU...................
Ga0247515_120448/91-178           .......................................................................................AGGGAAGCUG..CUUGGUGAAACACUUUUGUGUUGGUA......UCC.............
Ga0247518_120275/202-289          .......................................................................................AGGGAAGCUG..CUUGGUGAAACACUUUUGUGUUCUCGUC....................
Ga0247526_117235/152-313          ...............AUCGCGGCACACUUCCUCAGCUAC.........................................CGGAGUAGGAGAAGAAU..UCCAGGGAAACACUCUGUC....UUGGGUU..................
Ga0179947_1148619/378-303         ..................................................................................AGAGGUGAGAGGUUG..AUGAGGGAAACAUCACAG..............................
Ga0179947_1156513/31-105          ..................................................................................AGAGGUGAGAAGUUG..AUGAGGGAAACAUCUCAG..............................
Ga0247519_107918/217-58           ...............AUCGCAGAACACUUCCUCAGCUAC.........................................CGGAGUAGGAGAAGAAU..UCCAGGGAAACACUCCAUC.....UGGGUU..................
Ga0179944_1246019/41-131          ...................................................................................GGUAGACGAAAUGG..CUAAGGGAAACACUAGAAA....ACCCCCU..................
Ga0247529_103231/270-430          ...............AUCGCAGAACACUUCCUCAGCUAC.........................................CGGAGUAGGAGAAGAAU..UCCAGGGAAACACUCCAUCGU....GGGUU..................
Ga0247526_110221/603-443          ...............AUGACGGAACACUUCCUCAGCUAC.........................................CGGAGUAGGAGAAGAAU..UCCAGGGAAACACUCCAUCAU....GGGUU..................
Ga0179944_1119724/70-159          ................................................................................AGAGCCUC.CGAAGCCG..GCCAGGGAAACAUGCUUCUAAC..........................
Ga0247521_125910/353-214          ..................GAGGUGGUCAGGC.AGACUC..........................................GUCAGUGAGUGAAGUCA..GUGAGGGAAACACUGGA...GAAGG.......................
Ga0247524_111427/50-210           ...............AUGGCGGACCACUUCCUCAGCUAC.........................................CGGAGUAGGAGAAGAAU..UCCAGGGAAACACUCCAU....CUUGGGCU..................
Ga0247527_115808/280-440          ..............AUGGAAUGGGUGAUCCCUCAGCUAC.........................................CGAGGUAGGAGAAGAAU..CCCAGGGAAACACCGUAGC.....CUGGCU..................
Ga0247526_101949/462-575          ........................UAGGGUU..GCCUA..........................................GAGUCAUGGCGAAGUCA..GGCAGGAAAACACGGCUC..............................
Ga0247524_101842/357-244          ........................UAGGGUU..GCCUG..........................................GAGUCAUGGCGAAGUCA..GACAGGAAAACACGGCUC..............................
JGI12272J11983_1356304/1548-1654  .........................UGAUGUUGGCCA...............................................CCCAGUGAGGCAU..ACCAGGGAAACACUUGGG..............................
Ga0257141_1089020/433-224         ............................ACUACUACAAACGAAGAA....................................AUAACAAUGAAGAUG..ACCAGGGAAACAUGACGUACA...........................
Ga0247519_108765/580-693          .................AUCCUAGGGUU..GCCUGGA.............................................GUCAUGGCGAAGUCA..GACAGGAAAACACGGCUC..............................
Ga0257136_1059838/549-429         ..................................ACC...........................................AGCAUACAGAGAAGACA..CCAGAGGAAACACUCUCCAAGCUAGUAAAGAGUGAGCU..........
Ga0247518_108423/758-837          ................................GACAU.................................................GCGGGAAGCUU..GCCAGGGAAACACAAUAC..............................
Ga0247519_108375/286-206          .................................CUAU.............................................ACAUCUGGGAAGCUA..GCCAGGGAAACACCACU...............................
Ga0247521_124040/388-309          ................................GACAU.................................................GCGGGAAGCUU..GCCAGGGAAACACAAUAC..............................
Ga0247518_137824/301-231          ....................................................................................GAUGAGGAAGCAC..GCCAGGGAAACACAAUC...............................
Ga0247531_104861/1082-1012        ....................................................................................GAUGAGGAAGCAC..GCCAGGGAAACACAAUC...............................
Ga0247523_120120/325-403          .................................................................................CACAUGUAGGAAGCUA..GCCAGGGAAACAUCAACC..............................
Ga0247526_116548/279-354          ..................................................................................AUCCUCGGGAAGCAU..GACAGGGAAACACAUCU...............................
Ga0247520_104849/891-821          ....................................................................................GAUGAGGAAGUAC..GCCAGGGAAACACAAUC...............................
Ga0247529_111070/303-228          ..................................................................................AUCUUCGGGAAGCAU..GACAGGGAAACACAUCUG..............................
Ga0247516_124175/202-272          ....................................................................................GUGUGGGAAGCUA..GCGAGGGAAACAUGCAUA..............................
Ga0247519_113845/334-409          ..................................................................................AUCUUCGGGAAGCAU..GGCAGGGAAACACAUCUG..............................
Ga0247519_118271/334-405          ......................................................................................CCGGGAAGCUA..ACCAGGGAAACAUAGACU..............................
Ga0179955_1048341/123-51          .....................................................................................GCCGGGAAGACC..GCCAGGGAAACACACG................................
Ga0247524_126530/125-198          ....................................................................................GGAUGGGAAGUAA..CUCAGGGAAACACACAUU..............................
Ga0247514_114593/280-211          .....................................................................................GAGGAGAAGCCA..GCCAGGGAAACACAUUC...............................
Ga0247527_116942/161-88           ....................................................................................UGUCGCGAAGCUU..GCCAGGGAAACAUCGUUUU.............................
Ga0247514_107497/1071-1140        .....................................................................................GAGGAGAGGCUC..GCCAGGGAAACACGCAUC..............................
Ga0179944_1005311/261-382         .............................CUGUG.UGAGG..........................................CCGGGAGGGAAGACC..GCGAGGGAAACACGGC....UAAGCAUCCUACUCC.............
Ga0179934_1155244/114-26          ...................................GC..................................................CGGGAAAUCC..GUGAGGGAAACAUGC.CGUUAGAAUG......................
Ga0179955_1195671/481-563         ..................................................................................UGGUGCAUGAAGCAU..GGUAACGAAACACUCAGACUU...........................
Ga0179948_1254202/198-296         ....................................................................................GUGUGGGAAGACA..GCUAGCAAAACACCGCGUUUAAUAUGUUUA..................
Ga0179944_1204933/216-89          ...........................AGCGAUG.UGAGG..........................................CUUGAAGGGAAGACC..GCCAGGGAAACACGUAGUC.UCAGCAUCCUGCC...............
Ga0247529_116306/255-362          ...........................AUCGAAGGUU.............................................GUGUCAGGGAAGCAC..GGUAGGGAAACAUGCGUCGCAG..........................
Ga0179944_1191552/284-411         .............................CUGUG.UGAGG..........................................CUCGAUGGGAAGACA..GCCAGGGAAACACGCAGUC.UCAGCAUCCUACUCC.............
Ga0179944_1313268/129-232         .................................AGGG.............................................UUGAGGGGGAAGAAC..GCCAGGGAAACAUGCAUCGGCGCUC.......................
Ga0247526_117664/434-499          .....................................................................................CAUGGGAAGCUG..GCGAGGGAAACACACU................................
JGI12272J11983_1319142/230-159    ....................................................................................CCCCUGGAAGCAC..GGCAGGGAAACAUCAUAC..............................
Ga0247531_104608/1256-1177        ................................GACAU.................................................GCGGGAAGCUU..GCCAGGGAAACACAAUAC..............................
Ga0247519_104852/800-730          ..................................GGGU................................................CUGGGAAGCAC..GCCAGGGAAACACAAC................................
Ga0247524_105844/561-637          ...................................ACGA..............................................UCUGGGAAACUA..GGUAGGGAAACAUCAUC...............................
Ga0247524_121352/374-303          ...................................GA.................................................UCGGGAAGCUA..GGUGGGGAAACAUCAUG...............................
Ga0247519_103291/1381-1460        ...............................GUC.AC................................................UCCUGGAAGCAG..UGUGGGGAAACACCAUC...............................
Ga0179934_1197833/207-115         ..........................GAUGUUGUCAA...............................................CUGUGGGAAGACU..GGAAGGGAAACACUCU................................
                                  >>>>>>>>>>>>>>>>>>>>>>>>>>>>>>>>>>>>>..................>>>>>>>>>>>>>>>>>>>>>>>>>>>>>>>>>>....>>>>..<<<<<.......<<<<<<<<<<<<<<<<<<<<<<<<<<<<<<<<<<<<
                                  101....11..11...................12222..................0000.000..00..0000...111..12222222....1222..22221.......222222...............1..1.....111000
                                  ---------------------------------○○○○---------------------------------------------○○●●●●R●GAAG●●●--R●●AGGGAAACAY●●●●●○-----------------------------
```

alignment positions 429 · · · 488

Ga0257140_1130477/77-287	GUAA.................................UGUGCGUUAGCACAUUAACUGGU
Ga0247516_125012/316-428	AUUAACCUC............................CCCAGACUGGGAUAUUACCCGGA
Ga0247529_117158/253-331	UAAA..................................UUCCCCCGGCACAUUACCCAUC
Ga0179947_1156513/270-495	GUAU....................................CUGAGAGCAUAUUAACUCAU
Ga0247519_105128/410-263	AUUAACGA...............................CCACAGGGUAUAUUACCUCUU
Ga0257141_1138773/402-200	GAAA......AACACGAUGU.GG.UUCGGUG.CCAAAGAUAG.GGUGCAUAUUACCCUGC
Ga0247527_109126/592-695	CUUG......................CG...CGACCUCGUUCUGGAGCACCUUACCUGAC
Ga0247519_111615/148-45	CUUG......................CG...CGACCUCGUUCUGGAGCACCUUACCUGAC
Ga0247513_116034/391-494	CUUG......................CG...CGACCUAGUUCUGGAGCACCUUACCUGAC
Ga0257136_1044348/475-684	GUAA.................................UGUGCGUUAGCACAUUAACUGGU
Ga0247529_134913/224-64	AUUAC.......................AGUCCAAGGGUGAUGGAGGCACAUUAACUGGA
Ga0247519_110701/607-447	AUUAC.......................AGCCCAAGGGUGAUGGAGGCACAUUAACUGGA
Ga0247530_109035/489-319	CUAUUUGGCA..............................CCUACGGCACAUUAACUGAC
Ga0247526_105246/689-508	AAUUGG................................UCUAAGUGGCACAUUAACUGGA
Ga0247515_107373/548-731	AAUUGG................................CUUAAGUGGCACAUUAACUGGA
JGI12272J11983_1072695/645-539	CUUAGCAA................................CCACUGGCAUAUUAACUGGA
Ga0257141_1118781/153-31	GCGA................AGCUCACU.....AUAGCGAGGAGGGGAAAAUUAACCUGG
Ga0247522_108409/684-554	CCGCCGAA...............................GCGAGUAGCACAUUACCCGGU
Ga0257141_1100114/291-412	UACAAGC.............UGCAAGGCUCGA.UGGCGGAAAGGAGGAAAAUUAACCUGG
Ga0179955_1013331/439-311	AUAG.................UAUGACUUUUUC....AUACCGCGGGCACAUUACUGUCC
Ga0247519_101377/1717-1907	AUUGGC...............................GGCUGAAGAGCACAUUACCUGCU
Ga0247512_104440/911-1082	GCCAU........UACAG..CCAUAGACCUA.ACCAGAUUUCGGUAGCACAUUAACUGGC
Ga0179944_1246019/249-162	UGUA...............................CUACUUCUGUAGCACAUUACCUUGC
Ga0255059_10691632/166-76	CGAA.................................UUCCGAGUAGCACCUUAACUGGC
Ga0255062_11230321/200-110	CGAA.................................UUCCGAGUAGCACCUUAACUGGC
Ga0247525_114510/199-318	CAAG....................................CCGGUGGCACAUUAACUGGU
JGI12272J11983_1345183/364-258	AGCG.................................AC.CUGAUGGAAAAUUAACUCCC
Ga0247521_112559/317-205	AUUU........................CAACUGCU.ACCAUUGCAGUAAAUUAACUCAC
JGI12272J11983_1072695/40-150	AGCG.................................AC.CCAGUAGCACAUUAACUCCC
Ga0247513_128543/241-71	CUAUUUGGCA..............................CCUACGGCACAUUAACUGAC
Ga0247528_108647/438-562	AGCUA..................AUAGCACUCACAUUA..UUGGUGGCACAUUACUUGGA
Ga0247517_121471/387-499	AGU..........................AUCUAGUUACCAUACUAGAAUAUUAACUCAC
Ga0247515_120448/91-178	UUUAA..................GG........ACCCCUACAAAAGGCAAAUUAUCCAAG
Ga0247518_120275/202-289	CUUGAAA.......................GACGG..CUACAAAAGGCAAAUUAUCCAAG
Ga0247526_117235/152-313	AUUAC.......................AGCCCAAGGGUGACAGAGGCACAUUAACUGGA
Ga0179947_1148619/378-303	GUAA....................................CUGUGAGUAUAUUAACUCAU
Ga0179947_1156513/31-105	GUAU....................................CUGAGAGCAUAUUAACUCAU
Ga0247519_107918/217-58	AUUAU.......................AGCCCA.GGGUGAUGGAGGCACAUUAACUGGA
Ga0179944_1246019/41-131	UAAC........................AGGGAGU...GAUUAUGGGUAAAUUAACUUAG
Ga0247529_103231/270-430	AUUAU.......................AGCCC.AGGGUGAUGGAGGCACAUUAACUGGA
Ga0247526_110221/603-443	AUUAC.......................AGCCCAAGGGUGAUGGAGGCACAUUAACUGGA
Ga0179944_1119724/70-159	AAU.................................GUUGGACGUAGCACAUUAACUGGC
Ga0247521_125910/353-214	UUAUCAC..........................CCUUCAAAUCCAGGCACAUUAACUCAC
Ga0247524_111427/50-210	AUUAC.......................AGCCCAAGGGUGAUGGAGGCACAUUAACUGGA
Ga0247527_115808/280-440	AUUAC.......................GGCCAGUGGGUGCUACGGGCACAUUAACUGGG
Ga0247526_101949/462-575	AAACCCGA................................GAGUCGGCACAUUAACUGUC
Ga0247524_101842/357-244	AAACCCGA................................GAGUCGGCACAUUAACUGUC
JGI12272J11983_1356304/1548-1654	ACUAGCAA................................UCCAAGGCACAUUAACUGGU
Ga0257141_1089020/433-224	GUAA.................................UGUGCGUUAGCACAUUAACUGGU
Ga0247519_108765/580-693	AAGCCCGA................................GAGUCGGCAUAUUAACUGUC
Ga0257136_1059838/549-429	GCGA................AGCUCACU.....AUAGCGAGGAGGGGAAAAUUAACCUGG
Ga0247518_108423/758-837	CUUU....................................GUAUUGGGAAAUUAACUGGC
Ga0247519_108375/286-206	GUU......................................AGUGGGAAGAUUAACUGGC
Ga0247521_124040/388-309	CUUU....................................GUAUUGGGACAUUAACUGGC
Ga0247518_137824/301-231	UUUAU....................................GAUUGGCACAUUAACUGGC
Ga0247531_104861/1082-1012	UUUAU....................................GAUUGGCACAUUAACUGGC
Ga0247523_120120/325-403	UUCG....................................GGUUGAGCACAUUAACUGGU
Ga0247526_116548/279-354	AUAUC....................................AGAUGGCAAAUUAACUGUC
Ga0247520_104849/891-821	UUUAU....................................GAUUGGCACAUUAACUGGC
Ga0247529_111070/303-228	UAU.....................................CAGAUGGCAAAUUAACUGUC
Ga0247516_124175/202-272	CGU.....................................UAUGCAGCAUAUUAACUCGC
Ga0247519_113845/334-409	UAU.....................................CAGAUGGCAAAUUAACUGUC
Ga0247519_118271/334-405	GACAA...................................AGUCUAGUAAAUUAACUGGU
Ga0179955_1048341/123-51	UAUUUGGAAC................................CGUGGUACAUUAACUGGC
Ga0247524_126530/125-198	GGCAA...................................AAUGUGGGAAAUUAACUGAG
Ga0247514_114593/280-211	UUAAU....................................GGAUGGAAAAUUAACUGGC
Ga0247527_116942/161-88	UCUU...................................AAAACGAGCAUAUUAACUGGC
Ga0247514_107497/1071-1140	UCCA....................................GAUGUGGCAUAUUAACUGGC
Ga0179944_1005311/261-382	UUUC...................GG...GGGAUGACAGC...GCCGGUACAUUAACUCGC
Ga0179934_1155244/114-26	AAAAA...........................CAUUCUAUCGAGCAGCACAUUAACUCAC
Ga0179955_1195671/481-563	UAUUUC...............................AAGUCUGAGGCACAUUACUUACC
Ga0179948_1254202/198-296	UUUGGUU.....................UAAGC.......ACGCGGGCAAAUUACCUAGC
Ga0179944_1204933/216-89	UUUC.....................GG.GGGAUGAUCGCGACUGCGGUAAAUUAACUGGC
Ga0247529_116306/255-362	CCGU................................CUGCUACGCAGCACAUUAACUACC
Ga0179944_1191552/284-411	UUUC...................GG...GGGAUGAUCGCGACUACGGUACAUUAACUGGC
Ga0179944_1313268/129-232	ACGUAG...........................GAGCGUCGUUGUAGCACAUUAACUGGC
Ga0247526_117664/434-499	AUAU......................................AGUGGGAAAUUAACUCGC
JGI12272J11983_1319142/230-159	GUU.....................................GUAUGAGCACAUUAACUGCC
Ga0247531_104608/1256-1177	CUUU....................................GUAUUGGGACAUUAACUGGC
Ga0247519_104852/800-730	GAAA......................................GUUGGCAUAUUAACUGGC
Ga0247524_105844/561-637	UUUG.....................................GAUGAGUAAAUUAACUACC
Ga0247524_121352/374-303	AUUAC....................................CAUGAGGAAAUUAACCACC
Ga0247519_103291/1381-1460	GAUG.....................................GAUGGGCACAUUAACCACA
Ga0179934_1197833/207-115	ACCA......................................AGAGGUACAUUAACUUCC
	..........>>>>>>>>>>>>>>>>>>>>>>>>>>>>>>>>>>>>.........>>>>>
	..........000111.....1..1...............222222.........12222
	●●●●----------------------------------○○●●●●RGYA●AUUAACU●●Y

2 hairpin-initial

For a description of this alignment, see Supplementary Table 2

2.1 Multiple-sequence alignment

Each ribozyme in this alignment is identified by its genomic location in the form SEQID/START-END. SEQID (the sequence accession) is derived from sources such as IMG/M or GenBank. START is the coordinate of the 5′ nucleotide of the hairpin ribozyme, and END corresponds to the 3′ nucleotide. If START>END, then the ribozyme is on the reverse complement strand.

Nucleotides proposed to basepair as part of the consensus structure are shaded in color when they comprise Watson-Crick or G-U pairs. Otherwise they are shaded gray. Conserved stems are also indicated at the bottom of the alignment by angle brackets, where matching $<$ and $>$ denote base-paired columns. Below these angle brackets, the symbol “2” denotes base pairs exhibiting covariation according to the statistically well-founded R-scape method. “1” denotes base pairs exhibiting covariation according to R2R’s simplistic method. “0” denotes base pairs that are not observed to mutate and “?” denotes base pairs that have a significant frequency of non-canonical nucleotides for Watson-Crick or G-U pairs ($> 5\%$). Below these base pair annotation is the consensus sequence: “R” = “A” or “G”, “Y” = “C” or “U”, red nucleotides: nucleotide identity conserved more than 97% of the time, black nucleotides: 90%, gray nucleotides: 75%, red circle (•): nucleotide is present 97% of the time, black circle (•): 90%, gray circle (•): 75%, white circle (◦): 50%. All percentages of sequences just described (e.g. 97% conserved) assume that sequences have been weighted by the GSC algorithm implemented by the Infernal software package.

The alignment begins on the next page.

78 duplicate sequences are not shown in the alignment

alignment positions 1 · · · 150

```
Ga0257140_1130477/77-287             .....CAUUUGAGUUGUUAU.....AAC..................................................UUCUUC...............................AUGGAUAAAGCAUACUCUCAUAUCGGCUUAAGCCG
Ga0247516_125012/316-428             .....CUUCUGUCCGUCAAA.....AUGAA................................................GCACCU...............................AGGGUUCAA..........................
Ga0247529_117158/253-331             .....AGUCUGUCCCAG..................................................................................................GCAAUCAAA..........................
Ga0179947_1156513/270-495            .....CAACUGUCCAC.........GGAG.................................................GAUGUGUAUAGGAAAGAGAUCAUCUAU..........ACAAACCUGAGCGCUUAAAUUAGCGUACGAAGUUU
Ga0247519_105128/410-263             .....GGUCUGUCCUUUGGCAG.AG..........................................................................................UGGACUUUCGGCUUGAUCGUCCGAGAGGGGGCCAA
Ga0257141_1138773/402-200            .....GUGCGGUCCUAGCAUC....CAA..................................................ACACACACGGGCCAAAACAACCCAACAGUGACAACCAGAAAA..............................
Ga0247527_109126/592-695             .....UUGCAGUCAUUCCAG.....CA........................................................................................UUUUA..............................
Ga0247519_111615/148-45              .....UUGCAGUCAUUCCAG...............................................................................................CAAUCAA............................
Ga0247513_116034/391-494             .....UUGCAGUCAUUCCAG...............................................................................................CAAUCAA............................
Ga0257136_1044348/475-684            .....CAUUUGAGUUGUUAU.....AAC..................................................UUCUUC...............................AUGGAUAAAGCAUACUCUCAUAUCGGCUUAAGCCG
Ga0247529_134913/224-64              .....AAUCUGUCCCUACUUCG...GGGGUGCCAAACCCCAAGCACA...............................AGCUGA.........CCGUCAU...............AUGUG..............................
Ga0247519_110701/607-447             .....AAUCUGUCCCUACUUCG...GGGGUGCCAAACCCCAAGCACA...............................AGCUGA.........CCGUCAU...............AUGUG..............................
Ga0247530_109035/489-319             .....CUUCUGUCUCAGUCAAG...GGUUCCCAAGCCCAUGGAACA................................GG.UAUCU.CGGUUAAUGGUCAUCACA..........CGGCCUCGUGCAA......................
Ga0247526_105246/689-508             .....UCUCUGUCUCUGAUUCG...GGUUUUGCUAAAACCCAAGGCAUU.............................GGUUC.AGUGUGUCGUUGCUCGGGCCU..........UUCUUACCUAAACGGUUGUU...............
Ga0247515_107373/548-731             .....UCUCUGUCUCUGAUUCG...GGAUUUGCUAAAUCCCAAGGCAUU.............................GGUUCA.GUGUGUCGUU.GCUCAGGCCUUUC.UACC.UUAGU..............................
JGI12272J11983_1072695/645-539       .....CGAUUGAGCUGGG.......AUAACUG..............................................UGGAUUGCAUC..........................UAUGCU.............................
Ga0257141_1118781/153-31             .....AGUCUGUCCUG.AUGCU........................................................CGGU.................................UGCUA..............................
Ga0247522_108409/684-554             .....GUCCUGUCCUCUAGG.....AUGAA................................................GCACUAGGCGAGGUU.GCGGA................AAGAC..............................
Ga0257141_1100114/291-412            .....AGUCUGUCCUG.ACG.....CU...................................................CGGC.................................UGCUA..............................
Ga0179955_1013331/439-311            .....CGUCUGUCUCGAC.......AAC..................................................GUCUC................................ACUCAAAUA..........................
Ga0247519_101377/1717-1907           .....GAGUUGUCCGUGUGGAG...UUGACCACAAGCCUAUUCGUGGUCUGUAAGGUGGCUGAAAUAAUGCCUUACGACCCGCCGUUGACUAAAGUUG..CCUC...........UUCG...............................
Ga0247512_104440/911-1082            .....CUUCUGUCCCCAUCAC....UA...................................................UGUUCUGCAUCUG........................CUUU...............................
Ga0179944_1246019/249-162            .....UCAUUGUCCUAAGACU..............................................................................................UGGA...............................
Ga0255059_10691632/166-76            .....UAUCAGUGUGUGG.......AA...................................................GAGA.................................UGAGC..............................
Ga0255062_11230321/200-110           .....UAUCAGUGUGUGG.......AA...................................................GAGA.................................UGAGC..............................
Ga0247525_114510/199-318             .....UGUCUGUCCCGAAC......UACAUCAGGGUAUG.......................................CAACAGGACCUUCC.......................UUCAC..............................
JGI12272J11983_1345183/364-258       .....UUUCUGUCCCACA.......G....................................................UCC..................................GUGCGUAAAGCAAUGCCAAGCGGCUA.........
Ga0247521_112559/317-205             .....UCGCUGUGGUUUAUG.....GUAAA................................................GCC..................................UUAAUU.............................
JGI12272J11983_1072695/40-150        .....UCUUUGUCCUCGC............................................................AUCCGU...............................GUGCGUAUAGCGCAAUCCCAA..............
Ga0247513_128543/241-71              .....CUUCUGUCUCGGUCAAG...GGUUCCCAAGCCCAUGGAACA................................GG.UAUCU.CGGUUAAUGGUCAUCACA..........CGGCCUCGUGCAA......................
Ga0247528_108647/438-562             .....AUACUGUCUGAUU.......UC...................................................GUAUGG...............................UUUGUAUU...........................
Ga0247517_121471/387-499             .....CAGCUGUGGUUGGAUG....GUAAA................................................GCC..................................UUUAGU.............................
Ga0247515_120448/91-178              .....CAGCUGUCCU....................................................................................................UAACU..............................
Ga0247518_120275/202-289             .....CAGCUGUCCU....................................................................................................UAACU..............................
Ga0247526_117235/152-313             .....UUUCUGUCCCUACUUCG...GGGGUGCCAAACCCCAAGCACA...............................AGCUGA........ACCGCGAU...............AUGCG..............................
Ga0179947_1148619/378-303            .....UAAUUGAUC..CUCU...............................................................................................ACAUUG.............................
Ga0179947_1156513/31-105             .....AGAUUGAUC..CUCU...............................................................................................ACAUU..............................
Ga0247519_107918/217-58              .....AAUCUGUCCCUACUUC....GGGGGUGCCAAACCCCAAGCACA..............................AGCUGA........ACUGCGAU...............AUGAG..............................
Ga0179944_1246019/41-131             .....CCAUUGUGUCUACC................................................................................................GUAA...............................
Ga0247529_103231/270-430             .....AAUCUGUCCCUACUUCG...GGGGUGCCAAACCCCAAGCACA...............................AGCUGA........ACUGCGAU...............AUGAG..............................
Ga0247526_110221/603-443             .....AAUCUGUCCCUACUUCG...GGGGUGCCAAACCCCAAGCACA...............................AGCUGA.........CCGCCAU...............AUGUG..............................
Ga0179944_1119724/70-159             .....CGGUUGGGAGAG.CUCU.............................................................................................ACUCACAUA..........................
Ga0247524_111427/50-210              .....AAUCUGUCCCUACUUCG...GGGGUGCCAAACCCCAAGCACA...............................AGCUGA.........CCGUCAU...............AUGUG..............................
Ga0247521_125910/353-214             .....UGACGGUACUCA..GAC...CG...................................................GUUUCGUCUACCCACCUC...................ACUCUUUUUACAA......................
Ga0247527_115808/280-440             .....CGUCUGUCCCUACUUCG...GGGGUGCCAAACCCCAAGCACA...............................AGCUGC.........CAU.CCAU..............AUGUG..............................
Ga0247526_101949/462-575             .....GGACUGGACCAGGGCUC...AAUU.................................................AGGCUGGAUCUUG........................CAUCC..............................
Ga0247524_101842/357-244             .....GGACUGGACCAAGGCUC...AUUC.................................................AGGUUGGAUCUUG........................CAUCC..............................
JGI12272J11983_1356304/1548-1654     .....CUGUUGAGCUGGG.......ACAACCG..............................................UGGAUUGCAUCG.........................UCGC...............................
Ga0257141_1089020/433-224            .....CAUUUGAGUUGUUAU.....AA...................................................CUUC.................................UUCAUGGAUAAAGCAUACUCUCAUAUCGGCUUAAG
Ga0247519_108765/580-693             .....GGACUGGACCAAGGC.....UCA..................................................UUCAGGU........UGGAU.................CUUGC..............................
Ga0257136_1059838/549-429            .....AGUCUGUCCUG.AUGCU........................................................GGU..................................UGCUA..............................
Ga0247518_108423/758-837             ACAUCAAGCGGUCCGU.........U....................................................AUGUC................................CAUCA..............................
Ga0247519_108375/286-206             .....UAGCGGUCCAGGGGU..........................................................AUAG.................................UAGA...............................
Ga0247521_124040/388-309             .....AAGCUGUCCGU.........U....................................................AUGUC................................CAUCA..............................
Ga0247518_137824/301-231             .....GUGCGGUCCCAUC.................................................................................................UUCG...............................
Ga0247531_104861/1082-1012           .....GUGCGGUCCCAUC.................................................................................................UCCG...............................
Ga0247523_120120/325-403             .....UAGCGGUCCGCAUGU.....G.........................................................................................UAAUC..............................
Ga0247526_116548/279-354             .....AUGCGGUCGGAGGAU.....C.........................................................................................CUAG...............................
Ga0247520_104849/891-821             .....GUACGGUCCCAUC.................................................................................................UCCG...............................
Ga0247529_111070/303-228             .....AUGCGGUCGGGAGAU.....C.........................................................................................UUAG...............................
Ga0247516_124175/202-272             .....UAGCGGUCUACAC.................................................................................................UUAU...............................
Ga0247519_113845/334-409             .....AUGCGGUCGGGAGAU.....C.........................................................................................UUAG...............................
Ga0247519_118271/334-405             .....UAGCGGUCCGG...................................................................................................GACAUUA............................
Ga0179955_1048341/123-51             .....GGUCGGUCCGGU..................................................................................................UACAU..............................
Ga0247524_126530/125-198             .....UUACGGUCCAUCC.................................................................................................GAUGA..............................
Ga0247514_114593/280-211             .....AGGCAGUCCCUC..................................................................................................UUUUU..............................
Ga0247527_116942/161-88              .....AAGCGGGGCGGCA.................................................................................................UGCA...............................
Ga0247514_107497/1071-1140           .....GAGCAGUCCCUC..................................................................................................UACG...............................
Ga0179944_1005311/261-382            .....GGUCGGUCCUUCCGG.....G....................................................CAUCACAG.............................CGCAUGA............................
Ga0179934_1155244/114-26             .....GGAUGGUCCG..........AAG..................................................GC...................................AGAA...............................
Ga0179955_1195671/481-563            .....AUGCGGUAUGCAUCA...............................................................................................UAA................................
Ga0179948_1254202/198-296            .....AGUCGGUCCACAC.................................................................................................ACGUACUAAUA........................
Ga0179944_1204933/216-89             .....GGUCAGUCCUUCAGG.....G....................................................CAUCAUUGCU...........................UACAC..............................
Ga0247529_116306/255-362             .....GCGCUGUCCUGGCAC.....UUUU.................................................GACCUUCGAU...........................ACGG...............................
Ga0179944_1191552/284-411            .....CGUCGGUCCAUCGGG.....G....................................................CAUCACAG.............................CGCUUGA............................
Ga0179944_1313268/129-232            .....GUUCGGUCCUC..GA.....UGAAAC...............................................CCCU.................................GGCC...............................
Ga0247526_117664/434-499             .....CAGCGGUCCAUG..................................................................................................UUAU...............................
JGI12272J11983_1319142/230-159       .....GUGCGGUCGGGGG.................................................................................................ACCCC..............................
Ga0247531_104608/1256-1177           .....AAGCGGUCCGU.........U....................................................AUGUC................................CAUCA..............................
Ga0247519_104852/800-730             .....GUGCGGUCCGG..............................................................CCC..................................AUCC...............................
Ga0247524_105844/561-637             .....UAGUGGUCCAGA........CCA..................................................GU...................................GAAU...............................
Ga0247524_121352/374-303             .....UAGCGGUCCGG.........A....................................................UC...................................AUCU...............................
Ga0247519_103291/1381-1460           .....CUGCGGUCGGGA........U....................................................GUUGAC...............................UUAU...............................
Ga0179934_1197833/207-115            .....AGUCGGUCCACAG.......AAUCU................................................UUGGC..CAUC..........................AUCA...............................
                                     .....<<<<....<<<<<<<<<<<<.....................................................<<<<<<<<<<<<<<<<<<<<<<<<<<<<<<<<<<<<<...................................
                                     .....2221....222222211.00.....................................................2222...................1...1.....0000...................................
                                     -----●●●C●GUYY●●○○○○-----○○---------------------------------------------------○○○○---------------------------------●●●●○------------------------------
```

alignment positions 151 · · · 300

```
Ga0257140_1130477/77-287          GUGGCAUGGGUACGUUUAAACAUGUGUAGAGUGCAAAGGUUCCUUAAAAAAAGGUUUCAAACUUGCAUUUAAACUACUACAAAC.................................GAAGAA..................AUAACAAUG
Ga0247516_125012/316-428          .....................................................................................................................AGGUGUGGG...............UUUGACGAG
Ga0247529_117158/253-331          ................................................................................................................................................CUGGGG
Ga0179947_1156513/270-495         UACACUCGUUCUCGUUUAAAUCAGGAAUCAAGCGGAUUGUUUAUAUUAUAUAGACACAAUGCUUGAC.............................AUAGUUGAGAUUGAUCCUC..UACAUUAGAG..................GUGAG
Ga0247519_105128/410-263          GUGGCUUCGCUUUGAUCUUAAAAUUG..............................................................................................................CUGCU.CCAAAGGG
Ga0257141_1138773/402-200         ......................................................................................UGGU.GUCAUUGUUGG..........GGAGAGUGUGU................CGAUGCUAGAG
Ga0247527_109126/592-695          .............................................................................................................................................CUGGUUUAG
Ga0247519_111615/148-45           .............................................................................................................................................CUGGUUUAG
Ga0247513_116034/391-494          .............................................................................................................................................CUGGUUUAG
Ga0257136_1044348/475-684         GUGGCAUGGGUACGUUUAAACAUGUGUAGAGUGCAGAGGUUCCUUAAAAAAGGUUUCAAACUUGCAUUUAAACUACUACAAAC..................................GAAGAA..................AUAACAAUG
Ga0247529_134913/224-64           .....................................................................................................AUGGCGGACCACUUCCUCAGCUAC..............CGAAGUAGGAG
Ga0247519_110701/607-447          .....................................................................................................AUGGCGGACCACUUCCUCAGCUAC..............CGGAGUAGGAG
Ga0247530_109035/489-319          ................................................................................................UGUGUUUGUCCUUAGCCUCAGAUCUCCAG..............CUUGGCUGAAG
Ga0247526_105246/689-508          ................................................................................................AGACCUGAGUG.CUUUGCACUUGAUCCACC.............CGGAUCAGAAG
Ga0247515_107373/548-731          .......................................................................................GGUGUUUGAUACCUGAGUGCUUCUUGCACUUGAUCCACC.............CGGAUCAGAAG
JGI12272J11983_1072695/645-539    ................................................................................................................GAGUUGGCCA.....................CCCAGUG
Ga0257141_1118781/153-31          .......................................................................................................................ACCG................AGCGUACAGAG
Ga0247522_108409/684-554          ......................................................................................................UCCGCUGGCUUCAG..GGUGUGUG...............CCUAGAGAG
Ga0257141_1100114/291-412         .......................................................................................................................GUCGAA................CGUACAGAG
Ga0179955_1013331/439-311         ......................................................................................................................GAGACCACCAAUC............GUCGAAG
Ga0247519_101377/1717-1907        .................................................................................................GGGGUUUGACUG.....GACGGUGGGCGUGC...........CUCCAUACGGG
Ga0247512_104440/911-1082         ..............................................................................................................CAA.UGCAAAACAGGCGUCUGCCACU....GUGGUGGGAG
Ga0179944_1246019/249-162         ............................................................................................................................................AGUCUUAGGG
Ga0255059_10691632/166-76         .......................................................................................................................UCUCAA..................CCACAUG
Ga0255062_11230321/200-110        .......................................................................................................................UCUCGA..................CCACAUG
Ga0247525_114510/199-318          .............................................................................................................GGAAGGUCCUGUUGACU................GUUCGGGG
JGI12272J11983_1345183/364-258    ........................................................................................................................GGA....................UGUGGCG
Ga0247521_112559/317-205          ........................................................................................................................GGCA.................CAUAGGCUG
JGI12272J11983_1072695/40-150     .....................................................................................................................GCGGGUAGG...............A.GCGAGAG
Ga0247513_128543/241-71           ................................................................................................UGUGUUUGUCCUUAGCCUCAGAUCUCCAG..............CUUGGCUGAAG
Ga0247528_108647/438-562          .....................................................................................................................CCAUACAU..................AGUCAAG
Ga0247517_121471/387-499          ........................................................................................................................GGCA................CAUUCGGCUG
Ga0247515_120448/91-178           ..................................................................................................................................................AGGG
Ga0247518_120275/202-289          ..................................................................................................................................................AGGG
Ga0247526_117235/152-313          .....................................................................................................AUCGCGGCACACUUCCUCAGCUAC..............CGGAGUAGGAG
Ga0179947_1148619/378-303         .............................................................................................................................................AGAGGUGAG
Ga0179947_1156513/31-105          .............................................................................................................................................AGAGGUGAG
Ga0247519_107918/217-58           .....................................................................................................AUCGCAGAACACUUCCUCAGCUACC..............GGAGUAGGAG
Ga0179944_1246019/41-131          ..............................................................................................................................................GGUAGACG
Ga0247529_103231/270-430          .....................................................................................................AUCGCAGAACACUUCCUCAGCUAC..............CGGAGUAGGAG
Ga0247526_110221/603-443          .....................................................................................................AUGACGGAACACUUCCUCAGCUAC..............CGGAGUAGGAG
Ga0179944_1119724/70-159          ............................................................................................................................................AGAGCUC.CG
Ga0247524_111427/50-210           .....................................................................................................AUGGCGGACCACUUCCUCAGCUAC..............CGGAGUAGGAG
Ga0247521_125910/353-214          .........................................................................................................GAGGUGGUCAGGC.AGACUC..............GUCAGUGAGUG
Ga0247527_115808/280-440          ....................................................................................................AUGGAAUGGGUGAUCCCUCAGCUAC..............CGAGGUAGGAG
Ga0247526_101949/462-575          ..............................................................................................................UAGGGUU..GCCUA...............GAGUCAUGGCG
Ga0247524_101842/357-244          ..............................................................................................................UAGGGUU..GCCUG...............GAGUCAUGGCG
JGI12272J11983_1356304/1548-1654  ...............................................................................................................UGAUGUUGGCCA....................CCCAGUG
Ga0257141_1089020/433-224         CCGGUGGCAUGGGUACGUUUAAACAUGUGUAGAGUGCAGAGGUUCCUUAA.AAAGGUUUCAAACUUGGAUUUAAACUACUACAAAC.................................GAAGAA................AUAACAAUG
Ga0247519_108765/580-693          .......................................................................................................AUCCUAGGGUU..GCCUGGA..................GUCAUGGCG
Ga0257136_1059838/549-429         ........................................................................................................................ACC................AGCAUACAGAG
Ga0247518_108423/758-837          ......................................................................................................................GACAU......................GCGGG
Ga0247519_108375/286-206          .......................................................................................................................CUAU..................ACAUCUGGG
Ga0247521_124040/388-309          ......................................................................................................................GACAU......................GCGGG
Ga0247518_137824/301-231          ...............................................................................................................................................GAUGAGG
Ga0247531_104861/1082-1012        ...............................................................................................................................................GAUGAGG
Ga0247523_120120/325-403          ................................................................................................................C............................ACAUGUAGG
Ga0247526_116548/279-354          .............................................................................................................................................AUCCUCGGG
Ga0247520_104849/891-821          ...............................................................................................................................................GAUGAGG
Ga0247529_111070/303-228          .............................................................................................................................................AUCUUCGGG
Ga0247516_124175/202-272          ...............................................................................................................................................GUGUGGG
Ga0247519_113845/334-409          .............................................................................................................................................AUCUUCGGG
Ga0247519_118271/334-405          .................................................................................................................................................CCGGG
Ga0179955_1048341/123-51          ................................................................................................................................................GCCGGG
Ga0247524_126530/125-198          ...............................................................................................................................................GGAUGGG
Ga0247514_114593/280-211          ................................................................................................................................................GAGGAG
Ga0247527_116942/161-88           ...............................................................................................................................................UGUCGCG
Ga0247514_107497/1071-1140        ................................................................................................................................................GAGGAG
Ga0179944_1005311/261-382         ...................................................................................................................CUGUG.UGAGG...............CCGGGAGGG
Ga0179934_1155244/114-26          .........................................................................................................................GC.......................CGGG
Ga0179955_1195671/481-563         .............................................................................................................................................UGGUGCAUG
Ga0179948_1254202/198-296         ...............................................................................................................................................GUGUGGG
Ga0179944_1204933/216-89          .................................................................................................................AGCGAUG.UGAGG...............CUUGAAGGG
Ga0247529_116306/255-362          .................................................................................................................AUCGAAGGUU..................GUGUCAGGG
Ga0179944_1191552/284-411         ...................................................................................................................CUGUG.UGAGG...............CUCGAUGGG
Ga0179944_1313268/129-232         .......................................................................................................................AGGG..................UUGAGGGGG
Ga0247526_117664/434-499          ................................................................................................................................................CAUGGG
JGI12272J11983_1319142/230-159    ...............................................................................................................................................CCCCUGG
Ga0247531_104608/1256-1177        ......................................................................................................................GACAU......................GCGGG
Ga0247519_104852/800-730          ........................................................................................................................GGGU.....................CUGGG
Ga0247524_105844/561-637          .........................................................................................................................ACGA...................UCUGGG
Ga0247524_121352/374-303          .........................................................................................................................GA......................UCGGG
Ga0247519_103291/1381-1460        .....................................................................................................................GUC.AC.....................UCCUGG
Ga0179934_1197833/207-115         ................................................................................................................GAUGUUGUCAA....................CUGUGGG
                                  ......................................................................................>>>>>>>>>>>>>>>>>>>>>>>>>>>>>>>>>>>>>.............>>>>>>>>>>>>..
                                  ......................................................................................0000.....1...1...................2222.............00.112222222..
                                  -----------------------------------------------------------------------------------------------------------------------oooo------------------ooooo••R•G
```

alignment positions 301 · · · 428

```
Ga0257140_1130477/77-287        AAGAUG..ACCAGGGAAACAUGACGUACA...........................GUAA.............................................UGUGCGUUAGCACAUUAACUGGU
Ga0247516_125012/316-428        AAGAAUG.UCCGGUGAAACACAGCCUGGG...........................AUUAACCUC........................................CCCAGACUGGGAUAUUACCCGGA
Ga0247529_117158/253-331        AAGACU..GAUGGCAAAACACGGGAAAA............................UAAA..............................................UUCCCCCGGCACAUUACCCAUC
Ga0179947_1156513/270-495       AAGUUG..AUGAGGGAAACAUCUCAG..............................GUAU................................................CUGAGAGCAUAUUAACUCAU
Ga0247519_105128/410-263        AAGACUUGAAGAGAGAAACACCUGUG..............................AUUAACGA...........................................CCACAGGGUAUAUUACCUCUU
Ga0257141_1138773/402-200       AAGCAC..GCAGGUGAAACCACCGCUAUCUGUAAACAUCCAAUCCAACAGUGUGUUGAAA..................AACACGAUGU.GG.UUCGGUG.CCAAAGAUAG.GGUGCAUAUUACCCUGC
Ga0247527_109126/592-695        AAGCAAA.GUCAGUGAAACAUCCAGA.CGUCGUCGAAUCG................CUUG...................................CG...CGACCUCGUUCUGGAGCACCUUACCUGAC
Ga0247519_111615/148-45         AAGCAAA.GUCAGUGAAACAUCCAGA.CGUCGUCGAAUCG................CUUG...................................CG...CGACCUCGUUCUGGAGCACCUUACCUGAC
Ga0247513_116034/391-494        AAGCAAA.GUCAGUGAAACAUCCAGA.CGUCGUCGAAUCG................CUUG...................................CG...CGACCUAGUUCUGGAGCACCUUACCUGAC
Ga0257136_1044348/475-684       AAGAUG..ACCAGGGAAACAUGACGUACA...........................GUAA.............................................UGUGCGUUAGCACAUUAACUGGU
Ga0247529_134913/224-64         AAGAAU..UCCAGGGAAACACUCCAUC....UUGGAUU..................AUUAC...................................AGUCCAAGGGUGAUGGAGGCACAUUAACUGGA
Ga0247519_110701/607-447        AAGAAU..UCCAGGGAAACACUCCAUC....UUGGGUU..................AUUAC...................................AGCCCAAGGGUGAUGGAGGCACAUUAACUGGA
Ga0247530_109035/489-319        AAGAAGC.GUCAGGGAAACACGUAGG..............................CUAUUUGGCA..........................................CCUACGGCACAUUAACUGAC
Ga0247526_105246/689-508        AAGAUCU.UCCAGGGAAACACGCUUAGG............................AAUUGG............................................UCUAAGUGGCACAUUAACUGGA
Ga0247515_107373/548-731        AAGAUCU.UCCAGGGAAACACACUUAGG............................AAUUGG............................................CUUAAGUGGCACAUUAACUGGA
JGI12272J11983_1072695/645-539  AGGUCU..UCCAGGGAAACACAGUGG..............................CUUAGCAA............................................CCACUGGCAUAUUAACUGGA
Ga0257141_1118781/153-31        AAGACA..CCAGAGGAAACACUCUCCAAGCUAGUAAAGAGUGAGCU..........GCGA............................AGCUCACU.....AUAGCGAGGAGGGGAAAAUUAACCUGG
Ga0247522_108409/684-554        AAGGAUU.GCCGGUGAAACAUGCUCGC.............................CCGCCGAA............................................GCGAGUAGCACAUUACCGGU
Ga0257141_1100114/291-412       AAGACA..CCAGAGGAAACACUCCC..ACGCCA......CCUUGUA..........UACAAGC.........................UGCAAGGCUCGA.UGGCGGAAAGGAGGAAAAUUAACCUGG
Ga0179955_1013331/439-311       AAGACG..GGACAAGAAACACCGCGUAGUUUUGAAA....................CUAUAAUAGUAUGACUU.........................UUUC....AUACCGCGGCACAUUACUGUCC
Ga0247519_101377/1717-1907      AAGCUC..UGCAGUAAAACAUCUUCAGCG...........................AUUGGC...........................................GGCUGAAGAGCACAUUACCUGCU
Ga0247512_104440/911-1082       AGGAAGAGACCAGGGAAACAUGCUGUAGUCUAGUCUGGGUUUACGGGUAUGUA...GCCAU....................UACAG..CCAUAGACCUA.ACCAGAUUUCGGUAGCACAUUAACUGGC
Ga0179944_1246019/249-162       AAGUGA..GCAAGUGAAACAUGCAGAAGUAG.........................UGUA...........................................CUACUUCUGUAGCACAUUACCUUGC
Ga0255059_10691632/166-76       AAGAUA..GCCAGGGAAACAUACCGGAA............................CGAA.............................................UUCCGAGUAGCACCUUAACUGGC
Ga0255062_11230321/200-110      AAGAUA..GCCAGGGAAACAUACCGGAA............................CGAA.............................................UUCCGAGUAGCACCUUAACUGGC
Ga0247525_114510/199-318        AAGACA..ACCAGGGAAACACACCGG..............................CAAG................................................CCGGUGGCACAUUAACUGGU
JGI12272J11983_1345183/364-258  AAGAAAU.GGGAGGGAAACACAUCAGCGU...........................AGCG.............................................AC.CUGAUGGAAAAUUAACUCCC
Ga0247521_112559/317-205        AAGCGA..GUGAGGGAAACAUGUAAUUGUGAGUGGUUG..................AUUU....................................CAACUGCU.ACCAUUGCAGUAAAUUAACUCAC
JGI12272J11983_1072695/40-150   AAGAGAU.GGGAGGGAAACAUGCUGGAGU...........................AGCG.............................................AC.CCAGUAGCACAUUAACUCCC
Ga0247513_128543/241-71         AAGAAGC.GUCAGGGAAACACGUAGG..............................CUAUUUGGCA..........................................CCUACGGCACAUUAACUGAC
Ga0247528_108647/438-562        AAGUAU..UCCAAGGGAAACACACCACUUUGAUCAUCGUGCUAU............AGCUA..............................AUAGCACUCACAUUA..UUGGUGGCACAUUACUUGGA
Ga0247517_121471/387-499        AAGCUG..GUGAGGGAAACAUAGUGUUGUGGCUAGAU...................AGU......................................AUCUAGUUACCAUACUAGAAUAUUAACUCAC
Ga0247515_120448/91-178         AAGCUG..CUUGGUGAAACACUUUUGUGUUGGUA......UCC.............UUUAA..............................GG........ACCCCUACAAAAGGCAAAUUAUCCAAG
Ga0247518_120275/202-289        AAGCUG..CUUGGUGAAACACUUUUGUGUUC.......UCGUC.............CUUGAAA............................GACGG.........CUACAAAAGGCAAAUUAUCCAAG
Ga0247526_117235/152-313        AAGAAU..UCCAGGGAAACACUCUGUC.........UUGGGUU.............AUUAC..............................AGCCCAAGG.....GUGACAGAGGCACAUUAACUGGA
Ga0179947_1148619/378-303       AGGUUG..AUGAGGGAAACAUCACAG..............................GUAA................................................CUGUGAGUAUAUUAACUCAU
Ga0179947_1156513/31-105        AAGUUG..AUGAGGGAAACAUCUCAG..............................GUAU................................................CUGAGAGCAUAUUAACUCAU
Ga0247519_107918/217-58         AAGAAU..UCCAGGGAAACACUCCAUC.........UGGGUU..............AUUAU..............................AGC......CCAGGGUGAUGGAGGCACAUUAACUGGA
Ga0179944_1246019/41-131        AAAUGG..CUAAGGGAAACACUAGAAA.........ACCCCCU.............UAAC...............................AGGGAGU........GAUUAUGGGUAAAUUAACUUAG
Ga0247529_103231/270-430        AAGAAU..UCCAGGGAAACACUCCAUCGU.........GGGUU.............AUUAU..............................AGC......CCAGGGUGAUGGAGGCACAUUAACUGGA
Ga0247526_110221/603-443        AAGAAU..UCCAGGGAAACACUCCAUCAU....GGGUU..................AUUAC...................................AGCCCAAGGGUGAUGGAGGCACAUUAACUGGA
Ga0179944_1119724/70-159        AAGCCG..GCCAGGGAAACAUGCUUCUAACA.........................A..............................................UGUUGGACGUAGCACAUUAACUGGC
Ga0247524_111427/50-210         AAGAAU..UCCAGGGAAACACUCCAU.........CUUGGGCU.............AUUAC..............................AGCCCAAGGGU.....GAUGGAGGCACAUUAACUGGA
Ga0247521_125910/353-214        AAGUCA..GUGAGGGAAACACUGGA.............GAAGG.............UUAUCAC............................CCUUCAA..........AUCCAGGCACAUUAACUCAC
Ga0247527_115808/280-440        AAGAAU..CCCAGGGAAACACCGUAGC.....CUGGCU..................AUUAC...................................GGCCAGUGGGUGCUACGGGCACAUUAACUGGG
Ga0247526_101949/462-575        AAGUCA..GGCAGGAAAACACGGCUC..............................AAACCCGA............................................GAGUCGGCACAUUAACUGUC
Ga0247524_101842/357-244        AAGUCA..GACAGGAAAACACGGCUC..............................AAACCCGA............................................GAGUCGGCACAUUAACUGUC
JGI12272J11983_1356304/1548-1654 AGGCAU..ACCAGGGAAACACUUGGG..............................ACUAGCAA............................................UCCAAGGCACAUUAACUGGU
Ga0257141_1089020/433-224       AAGAUG..ACCAGGGAAACAUGACGUACA...........................GUAA.............................................UGUGCGUUAGCACAUUAACUGGU
Ga0247519_108765/580-693        AAGUCA..GACAGGAAAACACGGCUC..............................AAGCCCGA............................................GAGUCGGCAUAUUAACUGUC
Ga0257136_1059838/549-429       AAGACA..CCAGAGGAAACACUCUCCAAGCUAGUAAAGAGUGAGCU..........GCGA............................AGCUCACU.....AUAGCGAGGAGGGGAAAAUUAACCUGG
Ga0247518_108423/758-837        AAGCUU..GCCAGGGAAACACAAUAC..............................CUUU................................................GUAUUGGGAAAUUAACUGGC
Ga0247519_108375/286-206        AAGCUA..GCCAGGGAAACACCACU...............................GUU..................................................AGUGGGAAGAUUAACUGGC
Ga0247521_124040/388-309        AAGCUU..GCCAGGGAAACACAAUAC..............................CUUU................................................GUAUUGGGACAUUAACUGGC
Ga0247518_137824/301-231        AAGCAC..GCCAGGGAAACACAAUC...............................UUUAU................................................GAUUGGCACAUUAACUGGC
Ga0247531_104861/1082-1012      AAGCAC..GCCAGGGAAACACAAUC...............................UUUAU................................................GAUUGGCACAUUAACUGGC
Ga0247523_120120/325-403        AAGCUA..GCCAGGGAAACAUCAACC..............................UUCG................................................GGUUGAGCACAUUAACUGGU
Ga0247526_116548/279-354        AAGCAU..GACAGGGAAACACAUCU...............................AUAUC................................................AGAUGGCAAAUUAACUGUC
Ga0247520_104849/891-821        AAGUAC..GCCAGGGAAACACAAUC...............................UUUAU................................................GAUUGGCACAUUAACUGGC
Ga0247529_111070/303-228        AAGCAU..GACAGGGAAACACAUCUG..............................UAU.................................................CAGAUGGCAAAUUAACUGUC
Ga0247516_124175/202-272        AAGCUA..GCGAGGGAAACAUGCAUA..............................CGU.................................................UAUGCAGCAUAUUAACUCGC
Ga0247519_113845/334-409        AAGCAU..GGCAGGGAAACACAUCUG..............................UAU.................................................CAGAUGGCAAAUUAACUGUC
Ga0247519_118271/334-405        AAGCUA..ACCAGGGAAACAUAGACU..............................GACAA...............................................AGUCUAGUAAAUUAACUGGU
Ga0179955_1048341/123-51        AAGACC..GCCAGGGAAACACACG................................UAUUUGGAAC............................................CGUGGUACAUUAACUGGC
Ga0247524_126530/125-198        AAGUAA..CUCAGGGAAACACACAUU..............................GGCAA...............................................AAUGUGGGAAAUUAACUGAG
Ga0247514_114593/280-211        AAGCCA..GCCAGGGAAACACAUUC...............................UUAAU................................................GGAUGGAAAAUUAACUGGC
Ga0247527_116942/161-88         AAGCUU..GCCAGGGAAACAUCGUUUU.............................UCUU...............................................AAAACGAGCAUAUUAACUGGC
Ga0247514_107497/1071-1140      AGGCUC..GCCAGGGAAACACGCAUC..............................UCCA................................................GAUGUGGCAUAUUAACUGGC
Ga0179944_1005311/261-382       AAGACC..GCGAGGGAAACACGGC.UAAGCAUCCUACUCC................UUUC..................................GG...GGGAUGACAGCGCCGGUACAUUAACUCGC
Ga0179934_1155244/114-26        AAAUCC..GUGAGGGAAACAUGC.CGUUAGAAUG......................AAAAA.......................................CAUUCUAUCGAGCAGCACAUUAACUCAC
Ga0179955_1195671/481-563       AAGCAU..GGUAACGAAACACUCAGACUU...........................UAUUUC...........................................AAGUCUGAGGCACAUUACUUACC
Ga0179948_1254202/198-296       AAGACA..GCUAGCAAAACACCGCGUUUAAUAUGUUUA..................UUUGGUU.................................UAAGC.......ACGCGGGCAAAUUACCUAGC
Ga0179944_1204933/216-89        AAGACC..GCCAGGGAAACACGUAGUC.UCAGCAUCCUGCC...............UUUC................................GG.GGGAUGAUGCGCGACUGCGGUAAAUUAACUGGC
Ga0247529_116306/255-362        AAGCAC..GGUAGGGAAACAUGCGUCGCAG..........................CCGU............................................CUGCUACGCAGCACAUUAACUACC
Ga0179944_1191552/284-411       AAGACA..GCCAGGGAAACACGCAGUC.UCAGCAUCCUACUCC.............UUUC..............................GG...GGGAUGAUGCGCGACUACGGUACAUUAACUGGC
Ga0179944_1313268/129-232       AAGAAC..GCCAGGGAAACAUGCAUCGGCGCUC.......................ACGUAG.......................................GAGCGUCGUUGUAGCACAUUAACUGGC
Ga0247526_117664/434-499        AAGCUG..GCGAGGGAAACACACU................................AUAU..................................................AGUGGGAAAUUAACUCGC
JGI12272J11983_1319142/230-159  AAGCAC..GGCAGGGAAACAUCAUAC..............................GUU.................................................GUAUGAGCACAUUAACUGCC
Ga0247531_104608/1256-1177      AAGCUU..GCCAGGGAAACACAAUAC..............................CUUU................................................GUAUUGGGACAUUAACUGGC
Ga0247519_104852/800-730        AAGCAC..GCCAGGGAAACACAAC................................GAAA..................................................GUUGGCAUAUUAACUGGC
Ga0247524_105844/561-637        AAACUA..GGUAGGGAAACAUCAUC...............................UUUG.................................................GAUGAGUAAAUUAACUACC
Ga0247524_121352/374-303        AAGCUA..GGUGGGGAAACAUCAUG...............................AUUAC................................................CAUGAGGAAAUUAACCACC
Ga0247519_103291/1381-1460      AAGCAG..UGUGGGGAAACACCAUC...............................GAUG.................................................GAUGGGCACAUUAACCACA
Ga0179934_1197833/207-115       AAGACU..GGAAGGGAAACACUCU................................ACCA..................................................AGAGGUACAUUAACUUCC
                                ..>>>>..<<<<<.......<<<<<<<<<<<<<<<<<<<<<<<<<<<<<<<<<<<<......................>>>>>>>>>>>>>>>>>>>>>>>>>>>>>>>>>>>>.........>>>>>
                                ..1222..22221.......2222222..............11.1.....111000......................000111.....1.11..............2222222.........12222
                                AAG•••--R••AGGGAAACAY••••◦◦-----------------------------••••---------------------------------------------◦◦◦•••••RGYA•AUUAACU••Y
```

3 hairpin-extra-spruce-initial

For a description of this alignment, see Supplementary Table 2

3.1 Multiple-sequence alignment

Each ribozyme in this alignment is identified by its genomic location in the form SEQID/START-END. SEQID (the sequence accession) is derived from sources such as IMG/M or GenBank. START is the coordinate of the 5′ nucleotide of the hairpin ribozyme, and END corresponds to the 3′ nucleotide. If START>END, then the ribozyme is on the reverse complement strand.

Nucleotides proposed to basepair as part of the consensus structure are shaded in color when they comprise Watson-Crick or G-U pairs. Otherwise they are shaded gray. Conserved stems are also indicated at the bottom of the alignment by angle brackets, where matching $<$ and $>$ denote base-paired columns. Below these angle brackets, the symbol "2" denotes base pairs exhibiting covariation according to the statistically well-founded R-scape method. "1" denotes base pairs exhibiting covariation according to R2R's simplistic method. "0" denotes base pairs that are not observed to mutate and "?" denotes base pairs that have a significant frequency of non-canonical nucleotides for Watson-Crick or G-U pairs ($> 5\%$). Below these base pair annotation is the consensus sequence: "R" = "A" or "G", "Y" = "C" or "U", red nucleotides: nucleotide identity conserved more than 97% of the time, black nucleotides: 90%, gray nucleotides: 75%, red circle (•): nucleotide is present 97% of the time, black circle (•): 90%, gray circle (•): 75%, white circle (◦): 50%. All percentages of sequences just described (e.g. 97% conserved) assume that sequences have been weighted by the GSC algorithm implemented by the Infernal software package.

The alignment begins on the next page.

759 duplicate sequences are not shown in the alignment

alignment positions 1 · · · 150

```
Ga0247541_102964/376-269       AGUCUGAG.U.U.CAU.GG........UU.........................................................................................................CC..GAG...CC
Ga0182091_1018330/100-226      AUGCAGUA.U.U.UC............GA.A.A......................................................................................CUAGU.C.G..A.GCGC..............
Ga0316035_118720/195-42        UACUUGUC.U.U.UUG...........UA.C.AU.CC..................................................................................UUGGA.A.U..GUGUGUCUAA..CUAC.UCG
Ga0179944_1246019/41-131       CCAUUGUG.U.C.UAC.C.....................................................................................................................................
Ga0265747_125480/96-202        CCUUUGAG.U.U.AC...A.AU.....CA.A.GC.C...................................................................................................GACA..............
Ga0265757_115889/161-280       CCUCUGAG.U.U.UGU.AC........AA.A.UA.UU..................................................................................CAC............................
Ga0180107_1031754/4443-4551    CGUUUGUC.C.C.GUU.AC.CG.....CC.U.AU.GACA.GGCUA..........................................................................GCC............................
Ga0180107_1229893/426-318      AGUCUGAG.U.U.GAA.AU.C......U.......................................................................................................................
Ga0222728_1038816/522-635      GUGCUGUC.C.G.GAU...........GA.U....................................................................................................................
Ga0179955_1195671/481-563      AUGCGGUA.U.G.CAU.CA................................................................................................................................
Ga0265751_100560/274-382       CAUCGGUC.C.U.AUC.GU........GC..........................................................................................UCCGU.C....CU..................
Ga0265751_124015/160-14        CGUCUGAG.U.U.AAG.GU........AU.G........................................................................................CGUGU.C........................
Ga0316029_122532/20-172        UGGCGGUC.U.U.GUU.AA........GG.C.UA.GGUA.CGCCCUUACUUG..U.AAG.G...UA.A.......GG......A..UG.UAGAA.........................UGCUC.G.C..GCACGA..............
Ga0316031_106349/204-348       UGGCGGUC.U.U.GUU.GG.GC.....U.....A.GGUG.CGCCCCUACUUG..U.AAG.G...UA.A.......GG......G..GG.CAGAG.........................ACGCG.A........................
Ga0265759_136872/100-7         UUAUUGUU.C.G.A.............AG.U........................................................................................UGG............................
Ga0138291_1027114/229-62       CAUUUGAG.U.G.U.C.UC.AG.....AC.U.CU..U..................................................................................AAAUA.G.G..UGUUGUCCAG..GGG.....
Ga0138291_1177333/356-213      CGUUUGAG.U.G.UUU.AC........AC.U........................................................................................UAACU...G..GUCAGC......UUGC.A..
Ga0079074_1152161/13-203       CCGUUGAG.U.A.ACG...........UC.U.UC..G..................................................................................AUUUA.U........................
Ga0242657_1353977/135-311      CUUUUGUC.C.U.GAA.AG........CA......................................................................................................................
Ga0255293_1180932/373-295      CCGCUGUG.U.U.AUA.UG.CA.....UA......................................................................................................................
Ga0307482_1147486/107-4        UAACGGUC.C.C.AAG.AC.A............C...............GUG..G.UA.............................................................CCACC..........................
Ga0242647_1001226/1011-1115    UGAUUGAG.C.U.GGG...........UA.A.AC.....................................................................................AGUGG.A........................
Ga0316032_128094/136-241       GUGUUGAG.U.C.GGG............U.A.A...............................................................................................CAGUG..GAUU.ACA
Ga0184598_118707/299-404       UUGUUGAG.C.U.GGG.U...........A.A................................................................................................CAGUG..GAUU.GCA
Ga0213850_1470830/321-202      CGUCUGAG.U.G.UGC.AU........CA.U.G......................................................................................UCCCA..........................
Ga0307482_1003156/2075-2177    UGUCAGUC.U....CC.CU.AG.....AA.C..C.UUCG.GGUUGCCAAGUG..G.CU.........................................................................................
Ga0307482_1000291/592-698      UGUCAGUC.U....CC.CU.AG.....GA.C.UC.UUCG.AGUUGCCAAGUG..G.CU.........................................................................................
Ga0247532_117056/43-239        CGGUUGUC.C.G.GCA.AG.UG.....UG.A...........................................................................................GA.C........................
Ga0184582_120088/374-570       CGGUUGUC.C.G.GCA.AG.UG.....UG.A...........................................................................................GA.C........................
Ga0265742_1013613/579-453      AGUCAGUA.C.C.AGG...........UG.G.CG.CACU.ACACGCGCGCAA..A.UU.........................................................................................
Ga0265748_110851/279-405       AGUCAGUA.C.C.AGG...........UG.G.CG.CACU.ACAUGCGCGCAA..A.UU.........................................................................................
Ga0307482_1000262/4292-4390    UGUUUGAG.U.A.CUA.CC.G......AG.A.U......................................................................................ACCC...........................
Ga0307484_100453/182-280       UGUUUGAG.U.A.CUA.CC.G......AG.A.U......................................................................................ACCC...........................
Ga0247538_120078/87-186        GAUUUGAG.C.A.CUG.G..........G.G.UA.UCAA.UGCCAAGU...................................................................................................
Ga0307484_109934/368-484       AGUCUGAG.U.U.AAU.AC.UA.....GA.U........................................................................................CGC............................
Ga0184572_111224/187-319       AACCUGUC.U.A...........................................................................................................UCUGG.A.U..CGGUGCCACC..GCGU....
Ga0265744_102237/225-365       CUUCUGUC.U.C.AUC.GG........AG.A.AG.....................................................................................GCUCC..........................
Ga0265758_100384/785-1040      CUUCUGUC.C.U.ACU.GG........AG.A.AG.....................................................................................ACUCU.......AGCCUAAGC..UAGU.G..
Ga0265753_1005391/776-521      CUUCUGUC.C.U.ACU.GG........AG.A.AG.....................................................................................ACUCU.A.G..CCUAAGCUAG..UGUG....
Ga0265759_100623/780-1036      CUUCUGUC.C.U.ACU.GG........AG.A.AG.....................................................................................ACUCU.A.G..CCUAAGCUAG..UGUG....
Ga0247535_113123/229-119       CAGCUGUC.C.U.CUC.CU.CG.............................................................................................................................
Ga0316036_112404/482-376       CAGCUGUC.C.U.UCC.UC................................................................................................................................
Ga0247515_120448/91-178        CAGCUGUC.C.U.......................................................................................................................................
Ga0247518_120275/202-289       CAGCUGUC.C.U.......................................................................................................................................
Ga0307482_1000311/1469-1363    UCUCGGUC.C.G.GUA.UC.C.......U.C........................................................................................CGAUU.A.G..GUG.................
Ga0184603_150346/629-518       GUGUUGUC.C.U.GAA.......................................................................................................GUCCG..........................
Ga0307484_102855/507-659       GAGUGGUC.C.U.UGA.GC.G......AA.A.AG.CCCC.GGUGUCGAGGUC..U.UCG............................................................GAUCU.G.G..CCGUGG..............
Ga0316029_101396/632-732       UGACGGUC.C.U.AUG.AC.ACG.UG.GU.U.AC.....................................................................................GCC............................
Ga0184582_100659/424-334       GGUCAGAG.C.A.AG.............A.U.UC.CGG.................................................................................GAGCC.U........................
Ga0265754_1040230/51-148       GGUUUGAG.C.A.GAA.CC.UG.....AA..........................................................................................GAAAU.A........................
Ga0316039_120091/354-252       CUACUGUC.C.C.A..............U.G.AA.AG..................................................................................CAACC.C.G..C...................
Ga0307921_1050750/452-358      UUGUUGUC.C.C.AGA.AC.UC.....A...........................................................................................CUCU...........................
Ga0265759_141590/154-258       CGUUUGAG.U.A.GGA.UG........CU.A........................................................................................CUGUC.U........................
Ga0184602_130775/1275-1431     UUACUGUC.C.C.UCU..G.AG..AU.GA.A..........................................................................GCCUUA..AAA...GCC............................
Ga0213850_1052387/326-212      UGUCUGAG.U.U.GAG.UU...........A........................................................................................AACC...........................
Ga0213850_1393213/656-542      UGUCUGAG.U.U.GAG.UU...........A........................................................................................AACC...........................
Ga0184578_112042/249-140       AAUCUGUC.C.A.U.........................................................................................................CUUUU.C.G..C...................
Ga0316033_100036/207-98        AAUCUGUC.C.A.U.........................................................................................................CUUUU.C.G..C...................
Ga0247534_100007/116-225       AAUCUGUC.C.A.U.........................................................................................................CUUUU.C.G..C...................
Ga0184569_109084/220-111       AAUCUGUC.C.A.U.........................................................................................................CUUUU.C.G..C...................
Ga0206355_1134705/241-332      AGUCUGAG.U.U.GUU.UA.CG.............................................................................................................................
Ga0265754_1007706/448-552      UGCUUGUC.C.U.ACA.GG........GU.G.G........................................................................................UUC.G........................
Ga0213850_1056422/14-139       UGUCUGUC.U.C.GAC...........AU.G.........................................................................................UGGA..........................
Ga0180107_1033011/786-679      GGCUUGUC.C.C.ACU.AC........GG..........................................................................................UUCAC.U...CGUGAA...............
Ga0307483_1000720/1178-1064    CCUCUGGA.C.U.GAU.UU.CGU.GG.GA.C........................................................................................CACCA..........................
Ga0265755_100088/1451-1216     GUUCUGUC.C.G.UGA.GG...U.AG.AA.G.AC.AGGG.UGGCCAAUGGUC..C.U...................A......A..GG.UUUAAA...CGUGAGAGCGUAG..GGA...GCUCC.A.C..AGCCCAGCCU..G.GC.U..
Ga0310119_125223/193-319       UCUCUGUC.U.G.CCU.UC.GG.....UA.A........................................................................................GCGAU..........................
Ga0265754_1037910/322-179      UCUCUGUC.C.U.UAG.AG........A...........................................................................................UGAAG.C.G..CUAGCGUG............
Ga0316053_162144/16-125        UAUCGGUC.C...CAG.AU.UC.....GU.U.GG.UCUU.GACCAAAUCCGA..A.G..........................................................................................
Ga0242654_10007932/343-135     UUGCUGUC.U.U.GGA.UG........CG.G.UG.GGCC.AAGCCA.........................................................................UCC............................
Ga0153880_1315932/142-46       AGUUUGAG.G.U.CUC.UU.GU.....GA..........................................................................................UACCA.U.G..C...................
Ga0265750_1009277/794-905      CGUUUGAG.G.U.UCC.UU........CA.A........................................................................................CUUUG.UCG..CAC.................
Ga0265756_100928/447-557       CGUUUGAG.G.U.UCC.UU........CA.A........................................................................................CUUUG.UCG..CAC.................
Ga0265745_1004075/525-659      CGUUUGAG.G.U.UCC.UU........CA.A........................................................................................CUUUG.UCG..CAC.................
Ga0138291_1147840/370-201      CAUUUGAG.U.G.UU..UU.A......AA.C.UC.....................................................................................UUAAA.U.A..GGUGUUGUCC...GAA.GGG
Ga0138291_1027295/231-62       CAUUUGAG.U.G.UU..UU.A......AA.C.UC.....................................................................................UUAAA.U.A..GGUGUUGUCC...GAA.GGG
Ga0184602_109114/416-317       UCACUGUG.U.G.UGC.GU.AC.....CU.A....................................................................................................................
Ga0153880_1354682/355-454      UCACUGUG.U.G.UGC.GU.AC.....CU.A....................................................................................................................
Ga0242649_1016743/464-237      AAUCUGAG.U.A.GCU.GG........GC.U.AC.UGGA.GAGCG..........................................................................AAAGC.U........................
Ga0179943_1118543/65-150       ACGUUGUC.C.U.UAA.GA........U.......................................................................................................................
Ga0184568_108419/492-395       UGACAGUC.U.C.AUA.AG.U......CA.A.GA.CA..................................................................................GGCC...........................
Ga0265756_117960/316-418       GUUCAGAC.C.A.CAU.UG.UU.................................................................................................UCUUC.G........................
Ga0265759_100202/139-258       ACACUGUC.C.A.UAU.AG........A...........................................................................................UGAGG.C.U..GUAGG...............

                               <<<<.....<.<.<<<.<<.<<<.<<.............................................................................................<<<<<.<.<..<<<<<<<<<<..<<<<.<<<
                               2221.....2.2.222.22....................................................................................................2222...........................
                               ●●●C●GUC-C-●-●●●-○○--------○○-○--------------------------------------------------------------------------------------○○○○---------------------------
```

```
Ga0265748_107452/280-387        AGUCUGUC.C.A.CAU......................................................................................................UGUCG..........................
Ga0307484_149222/136-234        UCACGGUC.C.A.UGG.UG.U........................CAAGAC..A................................................................UGGU...........................
Ga0316038_107364/55-195         CCGCUGUC.C.U.UUU...........GA.........................................................................................AUUUA.U.U..CGAUUCGAAU..GAAC....
Ga0184593_123391/157-297        CCGCUGUC.C.U.UUU...........GA.........................................................................................AUUUA.U.U..CGAUUCGAAU..GAAC....
Ga0242652_1159268/284-150       UCUCUGUC.C.C.UUG...........GA.A.U............UAAGCG..A.UA.............................................................GGCUU.G.CGCCCCGUC..............
Ga0310117_113093/724-511        GGUUUGUC.C.G.UGA.AG........GU.G.AA.GGCC.AGGCAGGUGAG..A.AGUCC..UA.A......GG............................................CCGGA.G.A..AUGGGGUUC...........
Ga0179944_1119724/70-159        CGGUUGGG.A.G.AG..CU.CU.................................................................................................................................
Ga0316036_104840/706-578        CGUUUGAG.U.G.AUU...........G....G.....................................................................................AUCA....U..GCUAAUGAUU..CAGC.UCC
Ga0247538_106858/350-156        GUCCUGUC.C.C.AAG.AG........AU.G.........AAGCUCUAGGCG..A...............................................................GUAGG.A.G..GCUACCACUG..........
Ga0180107_1215312/308-184       GUCCUGUC.C.A.UAG.GU........UG.A.AC....................................................................................GCUU.......AGGCUGCAUG..UGA...C.
Ga0247534_100003/3147-3245      GUUCGGUC.C.C.AUA.AC.UCA.AG.............................................................................................................................
Ga0247519_101377/1717-1907      GAGUUGUC.C.G.UGU.GG.AG.....UU.G.AC.CACA.AGCCUAUUCGUG..G.UCU.G..UA.A......GG.....U..GG.CUGAAA..U..AAUGCCUUA..CGA.......CCCGC.C.G..UUGACUAAAG..UUG...CC
Ga0184588_143621/642-452        GAGUUGUC.C.G.UGU.GG.AG.....UU.G.AC.CACA.AGCCUAUUCGUG..G.UCU.G..UA.A......GG.....U..GG.CUGAAA..U..AAUGCCUUA..CGA.......CCCGC.C.G..UUGACUAAAG..UUG...CC
Ga0265758_100017/4028-4134      UAUCGGUC.C...CUA.UG.AU.....GGCA.AU.GGCC.AGAUCCAAGUGG...................................................................................................
Ga0265751_102572/231-125        UAUCGGUC.C...CUA.UG.AU.....GGCA.AU.GGCC.AGAUCCAAGUGG...................................................................................................
Ga0310106_100226/1503-1738      GUUCUGUC.C.G.UGA.GG........UA.G.AA.GACA.GGGUGGCCAAUG..A.UC..C..UA.A......GG.....U..UU.AAACGU..G..AGAGCGUAG..GGA.......GCUCC.A....CAGCCCAGCC..UGGC.UGG
Ga0184596_112078/314-191        AUACUGUC.U.G.AUU...........UC.........................................................................................GUAUG.G........................
Ga0247528_108647/438-562        AUACUGUC.U.G.AUU...........UC.........................................................................................GUAUG.G........................
Ga0184602_108471/49-173         AUACUGUC.U.G.AUU...........UC.........................................................................................GUAUG.G........................
Ga0316031_112736/216-74         CCGCGGUC.C.U.CUC.AU........AA.G.C.....................................................................................CCAUC.G.G..AGA.................
Ga0184575_106581/174-318        CCGCGGUC.C.U.CUC.AU........AA.G.C.....................................................................................CCAUC.G.G..AGA.................
Ga0184582_129189/472-619        CUUCUGUC.C.C.GUG.CG...G.AG..A.A.GG.CUCC.AGCCAUGGUGUG..G.UCU.G..........................................................................................
Ga0180052_116799/419-501        AGUCUGUC.U.C.GC.............A..........................................................................................................................
Ga0179955_1013331/439-311       CGUCUGUC.U.C.GAC...........AA.C.......................................................................................GUCUC..........................
Ga0157569_1058012/474-586       GGUCUGAC.C.A.CCU.AG........AG.C.A.....................................................................................UU.............................
Ga0157562_1164332/672-560       GGUCUGAC.C.A.CCU...........AU.A........................................................................................................................
Ga0265746_1084801/20-158        GGUCUGUC.U.U.GCU......................................................................................................CCCCA.A.C......................
Ga0265752_102479/223-81         GGUCUGUC.U.U.GCU......................................................................................................CCCCA.A.C......................
Ga0265756_107066/520-660        GGUCUGUC.U.U.GCU......................................................................................................CCCCA.A.C......................
Ga0247541_102884/37-209         UCUCUGUC.C.C.GGU.UC.UG......G.A.UU.CCCG.AAAUCCAUGGGU..A...............................................................UAGGC.G.G..GUAGGA..............
Ga0247516_125012/316-428        CUUCUGUC.C.G.UCA.AA........AU.G.AA....................................................................................GCACC.U........................
Ga0213850_1071966/331-222       UGCCUGAG.U.G.CGG......................................................................................................AGCUU.A.G..ACG.................
Ga0257141_1100114/291-412       AGUCUGUC.C.U.G.A.CG........CU.........................................................................................CGGC...........................
Ga0265740_1020297/298-150       UGUUUGUC.U.U.GUA...........A..........................................................................................ACACG.C........................
Ga0307482_1064439/425-316       AAUCAGUC.C.U.AUC.AU.UG...A............................................................................................UGGU...........................
Ga0138292_1031932/14-100        CAUCUGUC.C.U.GAG...........UU..........................................................................................................................
Ga0138291_1022124/340-254       CAUCUGUC.C.U.GAG...........UU..........................................................................................................................
Ga0184582_127657/238-384        GGGUUGUC.C.U.AUU.CU........GA.G............UCCGCGGUU..G.UU............................................................GGCAA.C.G..CGGGUCCUCA..A.......
Ga0184570_113141/244-341        UGUUUGAG.C.C.UUC...........AG.C.......................................................................................UCUC...........................
Ga0310119_152175/242-389        GCUCUGUC.C.U.UUC.UA..G..AU.GA.A....GCCU.AAAGACCUCGGG..U.CC.............................................................................................
Ga0184576_119948/531-664        UUCCUGUC.C.C.AGA.G.........UG.A.U....................................................................UAAGCAC..................U..AGGCC...GA..AGC..CUG
Ga0316034_129624/151-284        UUCCUGUC.C.C.AGA.G.........UG.A..................................................................U...UAAGCAC..................U..AGGCC...GA..AGC..CUG
Ga0307482_1134687/287-181       AUGCGGUC.C.C.AGG.U..........A.A........................................................................................C...............AGUG..GAUU.CAC
Ga0307484_104189/399-292        AUGCGGUC.C.C.AGG...........UA.A.AC.AG...................................................................................................UGGAUUC..AUCU.C..
Ga0138282_1242808/325-475       GAUCGGUC.C....GG.AGGCGC.GC.UG.C.AC....................................................................................GUCUG.C........................
Ga0180047_1081041/176-42        CGUUUGAG.U.U.GGU.A..CUC.CG............................................................................................CUCAA.G.A..UUCACAGAUU..CGGA....
Ga0265752_100023/1618-1523      CAUCGGUC.C.C.AUA.GU........C..........................................................................................C........................
Ga0242649_1051170/34-143        CAUUUGUC.C.U.GUU...........U..........................................................................................GAUGU.G.C..AUCUUUG.............
Ga0242654_10030584/1049-1243    CUUCUGUC.C.A.CAU.GU.GUU.AA............................................................................................GCGCU.A.G..GGUCGGUCUAACCAAC.UCC
Ga0307484_102966/727-825        AGUCUGUC.C.U.AU.............U...GU.GCCA.AUGCUUCAAGCG..A................................................................................................
Ga0307921_1020571/133-221       UGACUGAG.U.C.GAG.UU...................................................................................................CUGCG.G....A...................
Ga0184573_113483/125-229        CAUCUGUC.C.U.GCA.UA.GC..UG....G.......................................................................................CGAG..U........................
Ga0184576_116834/47-151         CAUCUGUC.C.U.GCA.UA.G......CU.........................................................................................GGCGA.G....U...................
Ga0184595_109216/770-611        CUUCUGUC.U.G.CAC.GU........GU.A.A.....................................................................................AACGU.A.G..CCAAGAUCGA..........
Ga0316040_154359/245-85         CUUCUGUC.U.G.CAC.GU........GU.A.UA....................................................................................AACGU.A.G..CCAAGAUCG...........
Ga0184595_116949/555-395        CUUCUGUC.U.G.CAC.GU........GU.A.UG....................................................................................AACGU.A.G..CCAAGAUCGA...CU...GG
Ga0247553_104889/634-563        AAGCUGUC.A.G.GUU.GA....................................................................................................................................
Ga0316046_107255/463-562        ACGCUGUC.C.C.CUU.UC.U......G..........A.UACCUGAAAGUG..................................................................CACU...........................
Ga0307484_102693/441-627        GUCCUGUC.U.C.GUA.CG...................................................................................................AUCAUGC.G..UAGGCACAUC..CGAC.CGU
Ga0184568_114257/261-413        CUUCUGUC.C.U.UGA.GU........GA.G.AA.G..................................................................................CCC.....A..GGCCGGGGA...........
Ga0316047_106478/13-108         CCACUGUC.C.U.GG.......................................................................................................GGUUC.U........................
Ga0242654_10024002/775-526      GGUCUGAG.G.U.CAC.AA.UC................................................................................................ACUUC.C.A..U....CC.............
Ga0316039_101269/650-841        UGUUUGGG.C.C.GGU...........UU.C........................................................................UUCCUUA..UGA..................................
Ga0265743_102316/615-423        UGUUUGGG.C.C.GGU...........UU.C........................................................................UUCCUUA..UGA..................................
Ga0210323_1072445/691-543       GUCCUGUC.C.A.CCA.UA.G..........................UAGUA..G...............................................................CGCUA.G.C..CUUGCCAAGA..CUGCAUUC
Ga0224712_10069322/1099-1357    CGUCUGUC.C.U.CCC.AG.GAC.AG.CA.G.U....................................................GG.AUGGCA........................CGCGA.U.U..CAAGCCAUCG..U.GC.A..
Ga0213850_1371453/186-298       UGUCGGUC.C.A.AAC.AG.AU.....UG.U.G......................................................................................................................
Ga0153880_1056505/290-430       GGCUUGAG.U.G.AUG.AU........GC.G.......................................................................................GGAUG.U.A..UUAGGUGACC..CACC.C..
Ga0179940_1033563/296-410       CGCCUGUC.C.U.CGA...........AC.A.......................................................................................UCCUU.U.C..GGCGUA..............
Ga0265743_143797/241-325        UGUCGGUC.C.C.UGA.AG.AG...................................................................................................U...........................
Ga0242654_10006748/25-247       GCGCUGUC.U.C.GGG.CC.GG.....GC.G.UA.CCCA.AGACAAAUCGGG..U.ACC.G..UA.A...................................................GGCUG.G.A..UUGACCAGUG....UC.GCG
Ga0138278_1149859/65-166        CGGCUGUC.U.G.UCG.CC.A......AU..........................................................................................................................
Ga0138278_1149142/191-91        CGGCUGUC.C.G.UUG.CG.A.......A..........................................................................................................................
Ga0138277_1147417/463-362       CGGCUGUC.U.G.UCG.CC.A......AU..........................................................................................................................
Ga0138278_1065026/314-214       CGGCUGUC.C.G.UUG.UC.A......AU..........................................................................................................................
Ga0138277_1089473/408-304       CGGCUGUC.C.G.UUG.CG.A.......A..........................................................................................................................
Ga0138277_1022592/399-305       CGGCUGUC.C.G.UUG.UC.A.......A..........................................................................................................................
Ga0157559_1099797/360-209       GGUUGGUC.C.G.AGC.GG........CU.U.AU.G..U.AAUAUACAUGAG..................................................................GCCUG.C.G..CUGGACGGCU..CCGU.A..
Ga0157572_1089974/360-209       GGUUGGUC.C.G.AGC.GG........CU.U.AU.G..U.AAUAUACAUGAG..................................................................GCCUG.C.G..CUGGACGGCU..CCGU.A..
Ga0242646_1007472/516-317       GUCCUGUC.C.A.AUG.UG........UU.A.AC.GCUC.AGGCC.........................................................................AUACC.G.A..CGAAUCGG............
Ga0184583_104740/342-477        GUCCUGUC.C.U.UAC.AG........AU.GAAG.CGCU.AGGCGACCCCGU..A.UC............................................................AGC............................
Ga0242654_11312609/202-83       AAACGGUC.C.G...............AG.A.......................................................................................GAGUA.G.U......................
Ga0184568_109070/428-286        GUUCUGUC.C.U.UUG.CA........GU.C.GA.G..................................................................................CUCUA.G.G..CAGUAGAAUA..CCAC.CUC
Ga0224712_10002011/1564-1454    UGUCUGUC.C.U.UAU.AU.CG.....CG.G.AU.CUCC.GGAUCUAGUCCC...................................................................................................
Ga0222728_1000095/4027-4133     UUUCUGUC.C.C.UAA.CU.C......GC.........................................................................................GCAC...........................
Ga0307482_1068632/529-339       UGUCUGUC.C.A.CUA.GA.CG.....GA.U........................................................................................................................
Ga0157560_1170105/128-245       GGGCGGUC.C.U.ACA.GU........UA.........................................................................................GUCUU..........................
Ga0316031_107021/319-214        UUUCUGUC.C.A.CCA.AU.C....................................................................................................C...........................
Ga0316047_100015/734-626        UUUCUGUC.U.C.CAG.AU.C..................................................................................................CG.U.G.A..GCGGUUG.............

                                <<<<.....<.<.<<<.<<.<<<.<<............................................................................................<<<<<.<.<..<<<<<<<<<<..<<<<.<<<
                                2221.....2.2.222.22...................................................................................................2222...........................
                                ●●●C●GUC–C–●–●●●–○○––––––––○○–○–––––––––––––––––––––––––––––––––––––––––––––––––––––––––––––––––––––––––––––––––––––––––○○○○–––––––––––––––––––––––––––
```

```
Ga0247533_100162/1643-1535      UUUCUGUC.U.C.CAG.AU.C.....................................................................................CG..........................
Ga0180108_1209503/1932-2029     GGUCUGUC.C.U.AGC.AC.U.....................................................................................GUG.........................
Ga0265753_1009881/576-819       GUUCUGUC.C.A.AUG.AG.C......UG.C.AA.....................UU.................................................CUCCA.G.G..CGGCGUUAAC..CGUC.U..
Ga0265741_101522/997-754        GUUCUGUC.C.A.AUG.AG.C......UG.C.AA.....................UU.................................................CUCCA.G.G..CGGCGUUAAC..CGUC....
Ga0184583_103998/587-740        AUUCUGUC.C.A.UC..GG.CG..AU.GA...AG.CGCU.AGGCA.............................................................GAUGC.C.C..GUUUCAACCC..AUC...UC
Ga0247521_125910/353-214        UGACGGUA.C.U.CA...G.AC.....CG.............................................................................GUUUC.G.U..CUACCCACCU..C.......
Ga0184569_106253/218-80         UGACGGUA.C.U.CA...G.ACC.GG.U...............................................................................UU.........................
Ga0316039_119781/347-457        UCUCAGUC.C.C.UAU..........GA.U............................................................................CGU.........................
Ga0184572_112077/359-259        UCUCGGUC.C.C.ACC.AU.......GA.C.AC.GCCC.AAGCUUUCGG.........................................................................................
Ga0257141_1118781/153-31        AGUCUGUC.C.U.G.A.UG.CU....................................................................................CGGU........................
Ga0257136_1059838/549-429       AGUCUGUC.C.U.G.A.UG.CU....................................................................................GGU.........................
Ga0138283_1149501/173-59        CGACGGUC.C.C.CCC..........................................................................................CGUCU.C.....................
Ga0184597_126105/545-362        GAUCUGUC.U.U.UGU.AC........GU.U............................................................................CAGC.C.C..UUAGUU.............
Ga0213850_1245299/581-409       AGAUUGUC.C.G.UAG...........UC.A.AC.AACA.AAUAUAAUUC........................................................................................
Ga0179948_1254202/198-296       AGUCGGUC.C.A.CAC..........................................................................................................................
Ga0180108_1093556/523-685       UCGUUGUC.C.G.AGA.GA........GG.G.AU.UCCU.AGGCUGACACGC......................................................................................
Ga0180107_1321405/371-533       UCGUUGUC.C.G.AGA.GA........GG.G.AU.UCCU.AGGC..............................................................UGACA.C.G..CUGGGAUGA.........
Ga0242641_1001197/544-746       CUUCUGUC.C.C.UUA.GG........AG.A.AG.GCUC.AGGCAUGGACCG..A...................................UA..CGC...AGGGA.G.U..CCCACCAGGG..UGU...UU
Ga0184597_115944/155-80         UAUCUGUA.U.C.UGG.UU.......................................................................................................................
Ga0184595_100702/414-262        AUUCUGUC.C.A.UCG.GU.GA...U..G...........AA................................................................GCGCU.......AGGCGAAUG..CCCG.UUU
Ga0184601_118759/683-531        AUUCUGUC.C.A.UCG.GU.G...AU.GA............A................................................................GCGCU.......AGGCGAAUG..CCCG.UUU
Ga0184592_125105/67-219         AUUCUGUC.C.A.UCG.GU.G...AU.GA............A................................................................GCGCU.......AGGCGAAUG..CCCG.UUU
Ga0257141_1089020/433-224       CAUUUGAG.U.U.GUU.AU........AA.............................................................................CUUC........................
Ga0265743_103007/262-368        CUGUUGAG.U.C.GG............................................................................................GUUA.A.C..AGUGGA.............
Ga0222728_1000095/4541-4435     UGUUUGAG.C.U.G.............................................................................................GGUUA.A.C..AGUGGA.............
Ga0247537_109082/42-148         UAUUUGAG.C.U.GGG...........................................................................................CAA........................
Ga0247533_100162/1117-1223      UGUUUGAG.C.U.G............................................................................................GGCAA........................
Ga0265757_100063/1489-1724      GUUCUGUC.C.A.UGA.GG........UA.G.AA.GACA.GGGUGGCCAAUG..G.UC.......................................................C.U....................
Ga0265747_118257/34-269         GUUCUGUC.C.G.UGA.GG........UA.G.AA.GACA.GGGUGGCCAAUG..G.UC.......................................................C.U..AAGGU.............
Ga0265745_1000046/1698-1933     GUUCUGUC.C.G.UGA.GG........UA.G.AA.GACA.GGGU..............................................................GGCCA.A.U..GAUUCUAA....GGU.....
Ga0115595_1153837/320-197       GGUCGGUC.C.U.AGC.CG.C......AU.G...........................................................................CCCG........................
Ga0242657_1026087/460-322       GUCCUGUC.C.A.UGG.GG........AU.G...................................................................................AAGCGCUAGG..CUU...UG
Ga0265751_100417/1480-1588      UGGCUGUC.C.G.CA..GC.UG.....GU.C.AU........................................................................AUCCU.C.....................
Ga0265756_100485/1108-1000      UGGCCGUC.C.G.CA..GC.UG.....GU.C.AU........................................................................AUCCU.C.....................
Ga0265751_100393/1594-1698      UUUCUGUC.C.U.ACA.CC........CG.U.GU.GGCA.ACACA.............................................................AUC.........................
Ga0307921_1011071/674-791       CGACUGAG.G.U.UGA.CU.......................................................................................CGUCU.C............A..CAAC.ACA
Ga0307919_1094420/160-285       CGACUGAG.G.U.GU..UC.UC....................................................................................UACUC.U............G..AAAC.UCG
Ga0316034_107719/314-205        GUUCGGUC.C.U.CAU.AU........UC.G.U..........................................................................GCG.C.....................
Ga0265742_1065119/361-273       AUGCUGUC.C.G.CAC.AA........AU.............................................................................................................
Ga0180107_1164033/560-431       AGUCCGUC.C.U.UCG.CG........UC.A.U.........................................................................UGCGU.U....CACACCCUUG..GGGU.GGA
Ga0242641_1001148/1456-1256     CGUCUGUC.C.C.GCU.UC.UG.....GA.C.UC.CCAA.AGUCCAUGGGUA...................................UGAAUUC..GGA...UAUAC.C.A..GGCACUCUAU..UGCA.G..
Ga0307482_1009758/562-669       CAUCUGUC.C.C.AAC.GU.CG......A.G...........CCAUAUGGC..A.....................................................AU.CC.C.....................
Ga0247527_109126/592-695        UUGCAGUC.A.U.UCC.AG........CA.............................................................................................................
Ga0247519_111615/148-45         UUGCAGUC.A.U.UCC.AG.......................................................................................................................
Ga0247513_116034/391-494        UUGCAGUC.A.U.UCC.AG.......................................................................................................................
Ga0213850_1290372/434-275       UCACUGUC.U.C.AUC...........UU.U.AG................AG.....................................................ACGAC.A.C..CGG................
Ga0138276_1121602/329-410       CUGCUGUC.C.A.GCG.AG........UU.U...........................................................................................................
Ga0138278_1041650/183-102       CUGCUGUC.C.A.GCG.AA.U.....................................................................................................................
Ga0138277_1137481/570-489       CUGCUGUC.U.A.GCA.AA........C..............................................................................................................
Ga0138278_1041788/293-378       CUGCUGUC.C.A.GCA.AA........C..............................................................................................................
Ga0138277_1142657/273-361       CUGCUGUC.C.A.GCA.AA........C..............................................................................................................
Ga0138275_1223952/371-283       CUGCUGUC.C.A.GCA.AA........C..............................................................................................................
Ga0213850_1450246/199-116       AGGCUGUC.C.C.GCG.GU.......................................................................................................................
Ga0213850_1307525/101-186       AGGCUGUC.C.G.CGG...........UG.............................................................................................................
Ga0265756_118952/425-318        GGGCUGUC.C.C.AG.............U.U.U.........................................................................GG.................U..GA......
Ga0180107_1244437/408-507       GAUCUGUC.C.A.AAG.CC........CA.G.AA.GG.....................................................................AUGU........................
Ga0265742_1051722/222-295       UUGCUGUC.U.C.GGC...........UU.G...........................................................................................................
Ga0242657_1051800/416-321       CAAUUGUC.C.C.AGG.UC........UA.U.A.........................................................................UGCGU.......................
Ga0307484_102966/353-250        UAACGGUC.C.C.AUG...........AC.................ACGUG..G.UUU...............................................CGCC........................
Ga0184568_108419/50-147         AGUCUGUC.C.U.UUU.UG.UC.....GA.U...........................................................................................................
Ga0184568_103677/484-581        GGUUUGAG.U.C.UUU.UG.CC.....UA.U...........................................................................ACUUC.......................
Ga0307484_102709/562-403        ACUCUGUC.C.C.AAG.AG........AG.A.AG.GUCU.AGCCCUGCGGGC..C.CCA.G...CA........................................................................
Ga0127503_10678853/419-333      UGCUUGUC.C.U.GUC.UC........GA.U.U.........................................................................................................
Ga0307919_1065410/76-162        UGCUUGUC.C.U.GU...........................................................................................CUCG........................
Ga0242652_1038498/365-470       UAUCUGUC.C.U.GUG.GU........AC.C...........................G...UU.A.......GG...............................CUUUG.G.....................
Ga0307484_101264/723-941        CUUCUGUC.C.C.UUG.AG........AG.A...........................................................................GAGCU.............CA..GGC...C.
Ga0247534_101560/228-122        GGUCUGUC.C.C.CUG.AC.CG.....C...............UCUAAAGG..A....................................................GCU.........................
Ga0307484_109931/110-215        AUGUUGAG.C.G.CA.............G.A.........................................................CUAC..AGA...GGAUU.......................
Ga0184595_116681/414-266        GUUCUGUC.C.C.CAG.AG.A......UG.A.AG.UGCU.AGGCC.............................................................GUAGC.C.C..GUGUCAUCUA..GCCG.C..
Ga0265749_100155/962-1187       GUACUGUC.C.A.CAA...........UG.G.U.............UGAAGC..G.UUA.G...GC.C.......GG......A..GC.CUGGAUCAGA...ACUGCCGUC..AGA...UUGAC.G.U..ACGCUCGA.....CCU.C..
Ga0310115_109341/244-400        GUCCUGUC.C.A.CAA...........UG.............................................................................GUUGA.A.G..CGUUAGGCCG..GAG.....
Ga0310111_106996/679-905        GUCCUGUC.C.A.CAA...........UG.G.U.............UGAAGC..G.UUA.G...GC.C.......GG......A..GC.CUGGAUCAGA...ACUGCCGUC..AGA...UU.GA.C.G..CACGCUCGAC..UUC.....
Ga0265756_101315/275-501        GUCCUGUC.C.A.CAA...........UG.G.UU.........................................GA.............................AGCGU.U.A..GGCC....GG..AGCC.UGG
Ga0265751_100874/820-594        GUCCUGUC.C.A.CAA...........UG.G.UU.........................................GAA...........GCGUUA..GGC...................C.....GG..AGCC.UGG
Ga0265744_120119/40-266         GUCCUGUC.C.A.CAA...........UG.G.UU.........................................GAA...........GCGUUA..GGC...................C.....GG..AGCC.UGG
Ga0310102_105010/226-452        GUCCUGUC.C.A.CAA...........UG.G.UU.........................................GA.............................AGCGU.U.A..GGCC....GG..AGCC.UGG
Ga0247534_103639/475-250        GUCCUGUC.C.A.CAA...........UG.G.UU.........................................GA.............................AGCGU.U.A..GGCC....GG..AGCC.UGG
Ga0265741_101833/434-208        GUCCUGUC.C.A.CAA...........UG.G.UU.GA......................................................................AGCGU.U.A..GGCCG....G..AGCC.UGG
Ga0242647_1002114/801-1026      GUCCUGUC.C.A.CAA...........UG.G.UU.GA......................................................................AGCGU.U.A..GGCCGGAGCC..UGG.....
Ga0265752_104585/383-158        GUCCUGUC.C.A.CAA...........UG.G.UU.GA......................................................................AGCGU.U.A..GGCCGGAGCC..UGG.....
Ga0310107_109330/1076-850       GUCCUGUC.C.A.CAA...........UG.G.UU.............GAAGC..G.UUA.G...GC.C..........................................................GG..AGCC.UGG
Ga0115595_1051440/308-419       UGACGGUC.C.U.UUU.AC........AU.A.U.........................................................................GACGC.A.....................
Ga0115594_1054328/389-276       UGACGGUC.C.U.UUU.AC........UU.A.AU........................................................................GACGC.A.....................
Ga0307482_1048753/383-528       GUCCUGUC.C.A.CCA...........UU.G.A....................................................................A..UGA...AGCGC.......................
Ga0307480_1006257/684-599       CGGCGGUC.C.A.CCC.AG........................................................................................GCAC.C.....................
Ga0184595_116802/212-325        AGGCGGUC.U.G.GCC.GU........U..............................................................................UUAU........................
Ga0307919_1071894/312-31        CUUUUGUC.C.G.CUU.UC........UA...........AUAACAUACGUA..C...................................................UCACC.C.A..AGGUGUGUCU..UCGG.CU.
Ga0307921_1036773/354-73        CUUUUGUC.C.G.CUU.UC........UA...........AUAACAUACGUA..C...................................................UCACC.C.A..AGGUGUGUCU..UCGG.CU.
Ga0307482_1023665/889-1191      GAUCUGUC.C.C.CCU.CC.....AG.GG.A.CC.UGCACAGCACCGAGGGC..A.AAA.GCGAUA.ACAGCCAGGGGAGAU.CCGG.AUGAAA...C...GACGGCAUG..CGA...GGAG.......ACCAGUAAUG..GUU...CG

                                <<<<.....<.<.<<<.<<.<<<.<<................................................................................<<<<<.<.<..<<<<<<<<<<..<<<<.<<<
                                2221.....2.2.222.22.......................................................................................2222........................
```

```
Ga0180108_1022680/3902-3998    AGUCAGUC.C.U.AAA.GA.U......GU.A.AG...............................................................................CGGU..........................
Ga0184599_137402/105-300       GGUCAGUC.C.U.AUC.AC.UAU.GU.CA.G.AG.GAGA.GAAUU............U.......................................................GCGUC.U.U..GGAAGCGAGC..........
Ga0157569_1080558/455-605      CGACUGUC.C.C.GCG.UC.AG.....UC.....................................................................................CGAUU.G.........................
Ga0184601_113231/247-149       UGUCAGUA.C.C.AUG.CU........GA.....................................................................................GGGGC.G.........................
Ga0242652_1002960/413-574      GUUCUGUC.C.C.UUC.UU.C......GG.G.UU.CCCA.AACCCCAGGUAC..A.C.........................................................................................
Ga0307484_101303/704-866       GUUCUGUC.C.C.UUC.UU.C......GG.G.UU.CCCA.AACCCCAGGUAC..A.C.........................................................AGUGG.C.A..CGGAGG..UG..GUGC.U..
Ga0242641_1025149/251-89       GUUCUGUC.C.C.UUC.UU.C......GG.G.UU.CCCA.AACCCCAGGUAC..A.C.........................................................AGUGG.C.....ACGGAGGAG..GUGC.U..
Ga0307482_1023050/594-496      GCUCGGUC.C.U.GGC.AU........UC.A.AA.AA...................................................................CGA...GAC..........................
Ga0307484_100434/1330-1210     UGUUUGUC.C.G.GUG.AC.C......AA.U...................................................................................CGGG..........................
Ga0153880_1432604/289-395      GGUCGGUC.U.U.GGU...........AG.A.AC................................................................................CACGC.U.........................
Ga0157559_1128929/201-87       GGUCUGUC.C.A.CC..AU........AA.....................................................................................................................
Ga0157560_1113202/155-269      GGUCUGUC.C.A.CC..AU........AA.....................................................................................................................
Ga0157566_1012736/497-384      GGUCUGUC.C.U.CC..AU...............................................................................................................................
Ga0157579_1155702/323-436      GGUCUGUC.C.U.CC..AU...............................................................................................................................
Ga0157562_1157806/189-74       GGUCUGUC.C.U.CC..AU...............................................................................................................................
Ga0180047_1032966/152-37       GGUCUGUC.C.U.CC..AU...............................................................................................................................
Ga0307484_166952/62-163        UGUCUGUG.U.U.ACU.AU........GC.G.AA..............GAA..A.C..........................................................GGGU..........................
Ga0005851_1178990/477-379      GGUCGGUC.C.U.C.............GA.C...................................................................................CGAUU.G.C..UUUCAU..............
Ga0153880_1191383/455-340      GAUCUGUC.C.U.AUU.UC........GA.....................................................................................GGUAA.........................
Ga0213850_1282564/267-350      GAGCAGUC.C.C.CC............G......................................................................................GCCCC.........................
Ga0315817_133437/498-413       GAGCAGUC.C.C.CC...................................................................................................GACCC.C.......................
Ga0315826_123252/262-347       GAGCAGUC.C.C.CC............G......................................................................................GCCCC.........................
Ga0242657_1008904/1267-1104    CAGCUGUC.C.U.UCA.GG.A......GG.U.UC.......................................................................................U.G..CU..................
Ga0222728_1009902/1182-968     GAGUUGUC.C.U.ACA.AG.UG...G.GU.U.GA.GCCC.AUACUUCUAGUG..G.AAU.G...AAGA.......GG......U..UA.AUGCAA...C...ACGGCUGGA..AUG...CCGG..........................
Ga0115594_1106220/441-334      GGUCGGUC.C.U.AGU...........CA.U...................................................................................ACACG.A.........................
Ga0180107_1307934/226-315      UUUCUGUC.C.G.GG............CA.U...................................................................................CU............................
Ga0184577_115871/202-369       CUCCUGUC.C.U.AAG.AG...............................................................................................AUGAA.G.C..GCUAGGCAG....AAG.UCG
Ga0206356_11590518/224-68      UGACGGUC.G.C.GCG.CU........GG.A.UA.AA.................U..........................................................CGUGU.A.........................
Ga0222728_1062510/166-66       CUGCGGUC.U.U.CCC...........GC.G...................................................................................CUAUU.A....UAGAGU..............
Ga0316029_101202/440-551       UAUUUGUC.U.C.UGG.GG........AA........................G.CGU.G...UA.C.......GG......U..GG.U....................A...CGA...AGGUC.........................
Ga0307482_1017422/1387-1206    UGUCUGUC.C.C.UAG.UU.CGG.GA.AU.U.AA.CCAA.AUUCCCAGGUUA..G.U.........................................................AGCUA.C.A..AGUUGU..............
Ga0307484_100889/412-594       UGUCUGUC.C.C.UAG.UU.CG.....GG.A.AU.U..A.ACCAAAUUCCCA..G...........................................................GUUAG.C.A..GUUACCAAGU..UGU...GU
Ga0255062_11230321/200-110     UAUCAGUG.U.G.UGG...........AA.....................................................................................GAGA..........................
Ga0255059_10691632/166-76      UAUCAGUG.U.G.UGG...........AA.....................................................................................GAGA..........................
Ga0265744_100073/1235-1401     UAUCUGUC.U.C.UGU.UA.UG......G.A.AU.CCCA.AAUUCCACGGGU..A.UG...............................GACC...U...ACAGCC..........AAUGG.C.........CCGAG..GGGC....
Ga0265757_113703/332-416       UUUCUGUC.C.G.C.............AA.A...................................................................................................................
Ga0265756_158829/72-188        UGACGGUC.C.G.GAU.CA........AA.C.........................................................A...AAUGUCU..........UUCG.U.........................
Ga0184597_100195/107-221       UGGCUGGA.C.C.A.............UA.U...................................................................................GCUCA.A.U..CAGG..............
Ga0184594_121503/721-607       GGACUGGA.C.C.A.............UA.U...................................................................................GCUCA.A.U..CAGG..............
Ga0184582_121032/1005-1119     GGACUGGA.C.C.A.............UA.U...................................................................................GCUCA.A.U..CAGG..............
Ga0184602_104873/671-785       GGAUUGGA.C.C.A.............UA.U...................................................................................GCUCA.A.U..CAGG..............
Ga0184589_132372/822-708       GGACUGGA.C.C.A.............UA.U...................................................................................GCUCA.A.U..CAGG..............
Ga0184580_125558/64-178        GGACUGGA.C.C.A.............UA.U...................................................................................GCUCA.A.U..CAGG..............
Ga0184596_101726/395-281       GGAUUGGA.C.C.A.............UA.U...................................................................................GCUCA.A.U..CAGG..............
Ga0247541_109256/178-292       GGACUGGA.C.C.A.............UA.U...................................................................................GCUCA.A.U..CAGG..............
Ga0184584_108444/253-366       GGACUGGA.C.C.AAG.GC.UC.....AU.U...................................................................................CAGG..........................
Ga0184582_124913/742-629       GGACUGGA.C.C.AAG.GC.UC.....AU.U...................................................................................CAGG..........................
Ga0184600_135218/318-205       GGACUGGA.C.C.AGG.GC.UC.....AU.U...................................................................................CAGG..........................
Ga0247541_100074/611-498       GGACUGGA.C.C.AGG.GC.UC.....AU.U.U.................................................................................AGG...........................
Ga0247551_101122/731-844       GGACUGGA.C.C.AGG.GC.UC.....AA.U.U.................................................................................AGGC..........................
Ga0247526_101949/462-575       GGACUGGA.C.C.AGG.GC.UC.....AA.U.U.................................................................................AGGCU.G.G..AUCUUG..............
Ga0247524_101842/357-244       GGACUGGA.C.C.AAG.GC.UC.....AU.U.C.................................................................................AGGUU.G.G..AUCUUG..............
Ga0247519_108765/580-693       GGACUGGA.C.C.AAG.GC........UC.A...................................................................................UUCAG.G.U.........UG..GAU.....
Ga0180107_1134582/382-542      UGUCUGUC.C.U.GA..CG.CCA.AG.AA.U.GC.UACU.UGGUCAGUAGCA..G.UAU.G...C.................................................UUCCA.C.U..G.....CUGU..AUGC.C..
Ga0265747_125301/405-306       CGUCAGUC.C.U.U.U.CU........GG.....................................................................................................................
Ga0157593_1101573/244-147      UGACUGUC.C.C.AUG.AG........GA.G.C.................................................................................................................
Ga0184599_121568/40-155        UGACUGUC.C.U.AUG.AG........GA.G.C.................................................................................................................
Ga0316040_116783/379-494       UGACUGUC.C.U.AUG.AG........GA.G.C.................................................................................................................
Ga0157560_1091679/440-538      UGACUGUC.C.C.AUG.AG........GA.G.C.................................................................................................................
Ga0222728_1165579/392-259      UGGUUGAG.U.U.CGA.CA.C......AC.A...................................................................................CGUGU.U....GUUAUCAACA..CCUU.C..
Ga0224712_10601975/393-502     CGUCUGUC.C.C.UAA.AG.UC.....GC.G.GG.AGAA.AUCCCAAUCUCA..A.GC........................................................................................
Ga0224712_10010317/1973-2082   CGUCUGUC.C.C.UAA.AG.UC.....GC.........G.GGAUUUAUCCCA..A...........................................................................................
Ga0307482_1048660/422-625      CUUCUGUC.C.C.GUU.GG........AG.A.G.......AGCCUAGGCAGG..G.UCU.G...GU.A.......GA......C..GU.GAGUAU...U...............................................
Ga0179934_1155244/114-26       GGAUGGUC.C.G...............AA.G...................................................................................GC............................
Ga0307484_101172/602-495       GUGUUGUC.C.G.AUU.UC.GUC.AU........................................................................................GGG...........................
Ga0242654_10068518/113-218     GUGUUGUC.C.G.AUU.UC..................................................................................................GU.C.A..UGGGGU..............
Ga0242647_1002406/915-1021     GUGUUGUC.C.G.AUU.UC..................................................................................................GU.C.A..UGGGGU..............
Ga0222729_1010500/11-117       GUGUUGUC.C.G.AUU.UC..................................................................................................GU.C.A..UGGGGU..............
Ga0307482_1202605/45-154       UUUCGGUC.C...UAU.GA.CC.....CU.C...................................................................................UCUC..........................
Ga0184603_139562/141-280       GAUCUGUC.C.C.UUG...........GA.G.G.................................................................................................................
Ga0242646_1001122/826-995      GAUCUGUC.C.G.CGCACA.C......GG.A.UU.CCUG.AAAUCCUAAGGU..A.U.........................................................GGCCG.G.G..AAAGGCUACA..GCU..CAU
Ga0310107_144222/327-436       UAUCGGUC.C.C.AUU.AC...............................................................................................GCUGC.........................
Ga0265751_124807/392-283       UAUCGGUC.C.C.AUU.AC...............................................................................................GCUGC.........................
Ga0184572_105168/388-281       UUUCGGUC.C.U.CCA.GU.U.......C.G...................................................................................UGCG..........................
Ga0316036_127178/124-231       UUUCGGUC.C.U.CCA.GU.U.......C.G...................................................................................UGCG..........................
Ga0222728_1003482/1262-1107    GUUCUGUC.C.U.GCG.UC........G..G.AC.UCUA.AGCCCGACAGAG..G.UA........................................................GUGUA.G.....UGGCCU............
Ga0265743_100639/33-191        UCGCUGUC.C.C.AGU.AG........GA.G.UA.AG.............................................................................CCCGA.........................
Ga0307484_105374/518-723       ACUCUGUC.C.C.UUU.AG.C......GG.A.AU.UCCC.AAAUUCCACGGG..............................................................UGGUU.U.U..ACGGAUUGUU..GCG..UGU
Ga0265742_1015608/410-491      GAUUUGUC.C.U.CUC.UC...............................................................................................................................
Ga0316030_102139/629-482       GUUCUGGA.C.A.GAA.AU.AC.....CA.U...................................................................................GCUGC.........................
Ga0265749_106268/131-212       GUGCUGUC.C.C.AAC...........AA.C...................................................................................................................
Ga0184573_100752/140-46        GAUCUGUC.C.U.GC...U.CA.....AA.A.......A.GGUCUCAUAGUU..A...........................................................................................
Ga0157560_1010344/357-456      UUACGGUC.C.U.AGC.UU...............................................................................................................................
Ga0157593_1006201/239-338      UUACGGUC.C.U.AGC.UU...............................................................................................................................
Ga0180047_1087048/281-180      UUACGGUC.C.U.AGC.UU...............................................................................................................................
Ga0157565_1016893/233-334      UUACGGUC.C.U.AGC.UU...............................................................................................................................
Ga0184568_100511/100-195       AGUCUGUC.C.G.CUG.AG........AU.G..........................................................AAGCCUAG..CGG..........................................
Ga0265759_114856/370-262       CGUCUGUC.C.U.AAG...........AA.U...................................................................................................................
Ga0222729_1080556/362-213      AAUCUGUC.C.U.U.............UA.....................................................................................GCCUA.C.......................

                               <<<<.....<.<.<<<.<<.<<<.<<........................................................................................<<<<<.<.<..<<<<<<<<<<..<<<<.<<<
                               2221.....2.2.222.22...............................................................................................2222............................
                               •••C•GUC-C-•-••••-○○--------○○-○--------------------------------------------------------------------------------○○○○----------------------------
```

```
Ga0247541_102707/328-184     CCUCUGUC.C.U.CUG.AA.G.......U.U.GA.UCUA.GUUCGUGAAACU..AGAC............................................................GC................CUG..GUAC.UCC
Ga0307921_1020694/406-483    CCACUGUC.C.U.AAG.AC..................................................................................................................................
Ga0307919_1034281/527-451    CCACUGUC.C.U.AAG.AC..................................................................................................................................
Ga0184568_108869/934-1072    CUUCUGUC.C.A.UGU.UG........AG.A.A.................GA..C.UC............................................................CACGC.C.A..U...GACGUG..GUUG.A..
Ga0206356_10036221/150-306   AGUCUGUC.A.U.AA...C.C...UG.GA.........................................................................................GUCUC..........................
Ga0247554_104968/233-375     CGUUUGAG.G.U.UGG.AU.AAC.GU.CA.........................................................................................CGC...A.U..AGCUAG..............
Ga0265753_1030448/124-358    CUUCUGUC.C.C.CUU.GG........AG.A.AG....................................................................................GUUCC.A.G..GCUUGGCCUG..UGUC.CCA
Ga0032354_1086862/128-19     ACCCUGUC.C.U.GAU..C.CGA.AA............................................................................................CUGAC.A........................
Ga0213850_1119088/23-236     AGUCGGUC.C.U.UAC.AC.AU.....A..........................................................................................GCGUG.G....ACAAUGAAAU..CAAC.ACC
Ga0222728_1000079/1837-1731  CUGUUGAG.U.U.GGG...........UU.A.AC.UG.................................................................................UGGA...........................
Ga0247535_100068/719-828     GUUCGGUC.C.U.CCA...........G..........................................................................................UUCG.U.G..CGCGGA..............
Ga0265744_100861/398-567     UGUCUGUC.U.C.CAA.UU.CG.....GA.A.UU.GACC.AAAUUCCAAGGU..C.AU............................................................GGGUC.A.....GAAUAUCGC...CGA.GUG
Ga0316038_100042/1676-1569   UCUCUGUC.C.U.GAG..U.CU.....C..........................................................................................GUCC...........................
Ga0184578_101068/480-310     CUUCUGUC.U.C.AGU.CA.A......GG.G.UU.CCCA.AGCCCAUGGAAC..A...............................................................GG.UA.U....CUCGGUUAAU..GGUC.AUC
Ga0265759_100473/818-988     CUUCUGUC.U.C.AGU.CA.A......GG.G.UU.CCCA.AGCCCAUGGAAC..A...............................................................GG.UA.U....CUCGGUUAAG..UGUC.AUC
Ga0265756_100592/1458-1288   CUUCUGUC.U.C.AGU.CA.A......GG.G.UU.CCCA.AGCCCAUGGAAC..A...............................................................GG.UA.U.C..U.CGGUUAAG..UGUC.AUC
Ga0265746_1003835/1049-1219  CUUCUGUC.U.C.AGU.CA.A......GG.G.UU.CCCA.AGCCCAUGGAAC..A...............................................................GG.UA.U.C..U.CGGUUAAG..UGUC.AUC
Ga0247536_100773/637-807     CUUCUGUC.U.C.AGU.CA.A......GG.G.UU.CCCA.AGCCCAUGGAAC..A...............................................................GG.UA.U.C..U.CGGUUAAU..GGUC.AUC
Ga0184573_112336/323-153     CUUCUGUC.U.U.GGU.CA.A......GG.G.UU.CCCA.AGCCCAUGGAAC..A...............................................................GG.UA.U.C..U.CGGUUAAU..GGUC.AUC
Ga0247530_109035/489-319     CUUCUGUC.U.C.AGU.CA.AG.....GG.U.UC.CCAA.GCCCAUGGAACA..................................................................GG.UA.U.C..U.CGGUUAAU..GGUC.AUC
Ga0247513_128543/241-71      CUUCUGUC.U.C.GGU.CA.AG.....GG.U.UC.CCAA.GCCCAUGGAACA..................................................................GG.UA.U.C..U.CGGUUAAU..GGUC.AUC
Ga0247541_101130/558-391     CUUCUGUC.U.C.CUC.CU.G......GG.G.CU.CCCA.AGCCCAAAGAGC..A...............................................................GGAAU.C.....UCGGUUAAG..CGC..CAG
Ga0213850_1278242/600-506    CGUCGGUC.U.C.GG............A..........................................................................................GCCC...........................
Ga0213850_1524759/286-380    CGUCGGUC.U.C.GG.............A.........................................................................................GCCC...........................
Ga0180107_1045142/740-541    CUUCUGUC.U.U.CAC.UU.CC.....GG.A.CU.CCCA.AAGUCCAUGGGU..................................................AUGCAACC..AGA...UGCUG.C.A..AGUGUCCUGU..CUUU.GUU
Ga0307484_102002/698-552     UUCCUGUC.C.U.ACA.AG........AU.G.AA....................................................................................GCGC...........................
Ga0265759_115288/4-102       AGACAGUC.C.A.CAG.GU........CA.U.CA...................................................................................................................
Ga0316029_136733/88-8        AUGUUGUC.C.C...............GA.........................................................................................GGC............................
Ga0242646_1008525/229-119    CAGCUGUC.C.G.CU......................................................................................................................................
Ga0115594_1180137/311-161    GCUCGGUU.C.C.GCG.AC........UG.C.AC.UGGA.AAGUGCGGUGUG..A...............................................................GGCCG.C.U..CGCGGUGA....GCUU.C..
Ga0179947_1156513/270-495    CAACUGUC.C.A.C.............GG.A.G.....................................................................................GAUGU.G.U..AUAGGAAAGA..GAUC.AUC
Ga0179948_1263234/366-292    AGAUUGAU.C....CU.CU..................................................................................................................................
Ga0179947_1148619/378-303    UAAUUGAU.C....CU.CU..................................................................................................................................
Ga0257141_1138773/402-200    GUGCGGUC.C.U.AGC.AU.C......CA.A.......................................................................................ACACA.C.A..CGGGCCAAAA..CAAC.CCA
Ga0242654_10401151/84-171    CAUCUGUC.C.................GA.U.......................................................................................CUCC...........................
Ga0184603_114388/264-401     UCACUGUC.C.G.AUU.UG.U...GA.CA.A.......................................................................................CUCUC.G.A..A......UCU..AUCG.C..
Ga0316052_118887/313-177     UCACUGUC.C.G.GUU.UG.U...GA.CA.A.......................................................................................CUCUC.G.A........AUCU..AUCG.C..
Ga0079039_1671802/197-34     CGUCGGUC.C.U.UUC.CG.GG.....CU.C.CA.CCCC.UUCCAAAGGUGU..A.GUU.U...UA.A......GG......C..CC...............................GUCCC...G..GGGACCAAAC..AGA.....
Ga0247542_100007/609-500     UUUCGGUC.C.U.ACA.CC...................................................................................................UCUCU.C........................
Ga0247552_100007/4311-4420   UAUCGGUC.C.U.ACA.CC...................................................................................................UCUCU.C.A..U...................
Ga0184586_103737/25-134      UUUCGGUC.C.U.ACA.CC...................................................................................................UCUCU.C........................
Ga0184594_103311/207-316     UAUCGGUC.C.U.ACA.CC...................................................................................................UCUCU.C........................
Ga0265756_101275/650-541     UAUCGGUC.C.U.ACA.CC...................................................................................................UCUCU.C........................
Ga0247541_100010/3268-3377   UUUCGGUC.C.U.ACA.CC...................................................................................................UCUCU.C........................
Ga0184599_154003/184-293     UUUCGGUC.C.U.ACA.CC...................................................................................................UCUCU.C........................
Ga0184580_121439/573-464     UUUCGGUC.C.U.ACA.CC...................................................................................................UCUCU.C........................
Ga0184582_108821/1050-1159   UAUCGGUC.C.U.ACA.CC...................................................................................................UCUCU.C........................
Ga0184575_101094/586-477     UAUCGGUC.C.U.ACA.CC...................................................................................................UCUCU.C........................
Ga0184598_129111/512-403     UAUCGGUC.C.U.ACA.CC...................................................................................................UCUCU.C........................
Ga0184596_107732/313-205     UAUCGGUC.C.U.ACA.CC...................................................................................................UCUCU.C........................
Ga0247554_100006/1592-1485   UAUCGGUC.C.U.ACA.CC...................................................................................................UCUCU.C........................
Ga0247534_100047/1899-2008   UAUCGGUC.C.U.ACA.CC...................................................................................................UCUCU.C.A..UGGAG...............
Ga0184597_128933/573-464     UAUCGGUC.C.U.ACA.CC...................................................................................................UCUCU.C........................
Ga0213850_1302171/343-113    CGUCUGUC.C.U.CCA.GG.GGGGAU.AA.A.GU.CUGA.AAUUCCGAGCAC......................AG......G..GG.CUG...........................CUUGA.A.A..UUACCCAAAA..........
Ga0213850_1513954/279-196    GGACGGUC.C.C.GGG.GU..................................................................................................................................
Ga0153880_1231581/116-241    CAGCUGUC.C.C...............AA.U.UG.GCCC.AUCCACGUGGUG..G.UC...........................................................................................
Ga0247541_104364/333-188     GAUUUGUA.C.U.UAG.UU.GAU.CC.AA.A.AG.AGAA.ACCUUAAGAGGU..A.UUC.U...U.....................................................UCAUU.A.U..CCGC................
Ga0138277_1028338/433-516    CUUCUGUC.C.G.A.............AG.A.A.....................................................................................CUAUA..........................
Ga0265752_101001/521-687     AUUCUGUC.C.C.UUG.AG.AG.....AA.A.G.....................................................................................CUCUA.G.G..CAUGGC..............
Ga0307482_1449537/408-223    GUUCUGUC.C.C.UGC.AA.GU..GG.CC.U.UU.UACA.AGACCAGGAAUG..A...............................................................GGGUC.U.U..UAGGUGAACC..GUGC.G..
Ga0257136_1044348/475-684    CAUUUGAG.U.U.GUU.AU........AA.C.......................................................................................UUCUU.C........................
Ga0257138_1055324/266-476    CAUUUGAG.U.U.GUU.AU........AA.C.......................................................................................UUCUU.C........................
Ga0257140_1130477/77-287     CAUUUGAG.U.U.GUU.AU........AA.C.......................................................................................UUCUU.C........................
Ga0127502_10711632/191-294   UAUCGGUC.C.A.GAU.AC.UGG.CG.AA.A.G..........CCU........................................................................GGG............................
Ga0184568_105547/129-239     UACUUGUC.C.C.UUC...........UG.C.U.....................................................................................GACUU.G.A..UCUCUAUG............
Ga0206353_11976089/292-380   GUUCGGUC.C.G.C.C.AA.CG.....G.........................................................................................................................
Ga0184577_108791/182-295     CGUCGGUG.C.G.GCA.CU.C......AA.G.AA....................................................................................CCUCA..........................
Ga0242649_1050441/340-170    GGGUUGUC.C.C.GAC.GA........GA.C.......................................................................................UGG............................
Ga0179934_1197833/207-115    AGUCGGUC.C.A.CAG...........AA.U.CU....................................................................................UUGGC......CAUC................
Ga0180107_1174136/218-102    UAGCGGUC.C.U.ACG.AG.A......UU.A.AG....................................................................................ACCAG.A.A..GAGACAAAGG...ACG.U..
Ga0224712_10002011/1106-1211 UAGUUGAG.C.U.GGG.UU.AAC.AG.UG.G.A.....................................................................................UCUCC.U........................
Ga0224712_10010317/2429-2325 UUGUUGAG.C.U.GGG.UA.ACA.GU.GG.A.......................................................................................UCUCU.C........................
Ga0206355_1311803/144-334    UGUCUGUC.G.U.CAU.AU.CC.....G..........................................................................................ACGGU.U.G..G.AUGG..............
Ga0307482_1417159/374-296    AGACGGUC.C...UAU.CA........U.........................................................................................................................
Ga0213850_1396191/936-678    CGUCUGUC.C.U.CCC.AG.GGG.CA............................................................................................AGUCA.G.A..CGGUCUGCGU..CGUA.UGC
Ga0222728_1287830/12-211     CAUCUGUA.C.U.CCA...........CA.C.AG.AGCA.AGUCUCAAACAG..U.AG...............................UGAAA...C...AACUACUUG..GUA...GUCGC.C........................
Ga0242652_1169169/12-211     CAUCUGUA.C.U.CCA...........CA.C.AG.AGCA.AGUCUCAAACAG..U.AG...............................UGAAA...C...AACUACUUG..GUA...GUCGC.C........................
Ga0242654_11312699/30-229    CAUCUGUA.C.U.CCA...........CA.C.A.....................................................................................GAACA.G.G..CCUCAAACAG..UAG.....
Ga0265759_104327/339-243     CCUCUGUC.C.U.CCC...........G.........................................................................................................................
Ga0265756_100717/910-1050    GUUCUGUC.C.A.ACG.UG........GU.G.AC....................................................................................AGUCC.U.A..GGGA................
Ga0265757_113513/277-362     GUUCUGUC.U...CCC.AC........UA.A......................................................................................................................
Ga0242654_10024002/836-915   ACUCUGUC.U.C.AAG...........CA.G.G....................................................................................................................
Ga0184573_101763/767-477     CUUCUGUC.C.C.UGA.UG.GA.....GA.A.GA.CUCC.AGGCAUGACGAA..G.CA............................................................UACCC.G.G..GAC.....AU..GAGA.ACA
Ga0242654_10701525/53-296    UCUCUGUC.C.C.UGCAUU.CG.....GA.C.UC.CCAA.AGUCCAUGGGUA..U...............................................................AGCGG.U.G..UUAUGUGCCA..CGUC.A..
Ga0247529_117158/253-331     AGUCUGUC.C.C.AG......................................................................................................................................
Ga0326631_117973/181-293     CCUCGGUC.C.U...............GA.G.U...AGC.AUCCUCUCACUG..................................................................UUCGC.A........................
Ga0316038_107116/33-174      UAUCGGUC.C.U.UCA.UC.UA..AC............................................................................................UGAG...........................
Ga0316037_108094/19-160      UAUCGGUC.C.U.UCA.UC.UA..AC............................................................................................UGAG...........................
Ga0247527_116942/161-88      AAGCGGGG.C.G.GCA.....................................................................................................................................

                             <<<<.....<.<.<<<.<<.<<<.<<............................................................................................<<<<<.<.<..<<<<<<<<<<..<<<<.<<<
                             2221.....2.2.222.22...................................................................................................2222...........................
                             ●●●C●GUC-C-●-●●●-○○--------○○-○---------------------------------------------------------------------------------------○○○○---------------------------
```

```
Ga0138278__1022898/219-127      CGUCGGUC.C.U.C.............AA...........................................................................................GGUC...........................
Ga0127503__11028958/51-130      CGUCUGUC.A.U.UUU.GU.CA.....UA..........................................................................................................................
Ga0307482__1623775/241-347      CGUCUGUC.C.U.UGA.AA.C..........................................................................................................UGUC...........................
Ga0307484__111921/444-550       UGUCUGUC.C.U.UGA.AA.C..........................................................................................................UGUC...........................
Ga0307484__101582/1018-1124     CGUCUGUC.C.U.UGA.AA.C..........................................................................................................UGUC...........................
Ga0265742__1021317/385-459      CGUCAGUC.C.U.GGA.CA........GA..........................................................................................................................
Ga0157569__1089063/252-138      GGUCUGUC.C.A.CC..AU........AA..........................................................................................................................
Ga0153880__1457361/236-121      GGUCUGUC.C.U.CCA.UA........AA..........................................................................................................................
Ga0256352__1087084/23-118       GGUCUGUC.C.U.CCA.CU........AA..........................................................................................................................
Ga0307482__1000325/24-131       GAUCGGUC.C.U.CUG.UC........GC......U....................................................................................GUCUU.C....C...................
Ga0157560__1147189/148-60       GUACGGUC.C.A.AGC............G...........................................................................................CGGCA.A........................
Ga0247519__105128/410-263       GGUCUGUC.C.U.UUG.GC.AG..AG.............................................................................................................................
Ga0184573__113193/903-756       GGUCUGUC.C.U.UUG.GC.AG..AG.............................................................................................................................
Ga0157593__1013623/299-215      CGUUUGUC.C.C...............AA.G.AC.....................................................................................................................
Ga0184593__126886/349-213       CCUCUGUC.C.C.ACA.UA.CG.....UA.U.CC.UCCA.GACACAAUGGUC....................................................................UUUUU.G......GCC...............
Ga0184568__117306/561-697       CCUCUGUC.C.C.ACA.CG.CG.....UA.U.CC.UCCA.GACACAAUGGUC....................................................................UUUUU.G......GCC...............
Ga0184597__120210/233-147       AGUCGGUC.C.C.GC............G...........................................................................................GCAC...........................
Ga0184570__100371/69-155        AGUCAGUC.C.C.GC............G...........................................................................................GCAC...........................
Ga0157562__1160399/232-315      GGUCGGUC.C.C.AUA.GA.UU......G..........................................................................................................................
Ga0265744__114151/132-24        UCGCGGUC.C.A.UUA.GA.A......GC.G.A......................................................................................CUGG...........................
Ga0265743__119695/104-184       GCUCGGUC.C.G.UGC.AC........G...........................................................................................................................
Ga0265743__101198/448-619       CGUCUGUC.U.U.CAG.UC.CGG.AA.UU.U.GA.CUAA.AU.UCCAAGGUC..A.GU..............................................................GGGUC.A....GAAAUAUAGC..C.AG.UGG
Ga0316046__121611/330-214       AGUCUGAG.C.A.CA.............C.U.AU.ACCA.AACUUCU.GUUG..G.UC.............................................................................................
Ga0265757__120140/89-204        AGUCUGAG.C.A.CA.............C.U.GU.CCUA.AACUUCUGUUAG..G................................................................................................
Ga0138279__1083071/419-326      CUGCGGUC.C.G.UAG...........GG.U.........................................................................................CCGA.......CGCGC...............
Ga0310106__100998/1608-1690     AUGCGGUC.C.U.AGA.AG....................................................................................................................................
Ga0265756__102054/286-395       GUUCGGUC.C.C.AGA.AU.CC.........................................................................................................GUGUA.C.G..UUGAG...............
Ga0180108__1209503/2438-2343    AGACAGUC.C.G.UGA.CU........GA.A.GA.C....................................................................................AGGG...........................
Ga0180107__1057591/210-107      UAGUUGUC.C.A.GAU.AU.A......UA.U.CC.UGAA.ACCCUCGAGAGG..G................................................................................................
Ga0307482__1074296/478-354      CCACUGUC.U.C.GAG...........CU.G.........................................................................................C............................
Ga0180108__1022680/4248-4153    GAAUUGUC.C.G.UAG.CC.A......UU.A.U.......................................................................................GGAG...........................
Ga0247519__103291/1381-1460     CUGCGGUC.G.G.GA............U...........................................................................................GUUGA.C........................
Ga0184590__126350/300-380       CUGCGGUC.G.G.GA............UG.U........................................................................................UGAC...........................
Ga0247551__100996/477-556       CUGCGGUC.G.G.GA............UG.U........................................................................................UGAC...........................
Ga0316035__144545/195-301       CCUCGGUC.C.A.UUG.AC........UU.U........................................................................................................................
Ga0316049__154968/227-319       UUACGGUC.C.U.A.U.GC........AA.C.A......................................................................................UUGGA.U........................
Ga0184593__115334/333-240       UUACGGUC.C.C.A.U.G..........U.A........................................................................................ACAUC.G.A......................
Ga0307921__1005950/551-460      UGUCUGUC.C.A.GG............GA.U.UC.....................................................................................GCAUU..........................
Ga0242647__1014694/423-336      GAUUGGUC.C.A.GCU.AG........CA..........................................................................................GCA............................
Ga0184580__102899/442-582       CCUCGGUC.C.U.UGA.UG.GA.............AGCC.GCCCCAUUGUCA........................G...........................................GGGG...........................
Ga0265743__108998/150-253       UUUCUGUC.C.C.ACA.GU.C......CG..........................................................................................................................
Ga0316050__103193/566-460       UAUCUGUC.C.C.UCA.GU.C......CG..........................................................................................................................
Ga0316030__100208/805-700       UAUCUGUC.C.A.UCA.GU.C..................................................................................................................................
Ga0307482__1000262/4800-4699    UCUCUGUC.C...CAU.AC.G......AU.C.AU.....................................................................................GCACA.A........................
Ga0307482__1018929/1182-1081    CCUCUGUC.C...CAU.AC.G......AU.C.AU.....................................................................................GCACA.A........................
Ga0153880__1260556/441-589      GCUCGGUC.C.A.AGC...........AC.A.G......................................................................................CACUA.C....C...................
Ga0180044__1087599/197-316      UUUCUGUC.C.C.UAC.GG.CG.....G...........................................................................................GAGUC..........................
Ga0127502__11371108/362-252     UCUUUGUC.C.U.ACA.GU.C...........C......................................................................................GUCC...........................
Ga0184588__106948/865-640       GUCCUGUC.C.C.CUG.GA.CG........G.AA.C...................................................................................................................
Ga0247533__100438/1040-888      UGUCUGUC.C.C.GGA.CU.C......AU.C.A......................................................................................................................
Ga0127502__10189886/163-55      UGUCGGUC.C.G.UUC.UC....................................................................................................................................
Ga0079074__1318576/412-571      UGUUUGUC.C.C.UUC.GG.C......AC.U.GC.UGCA.AAACGCACAGCU..G.CCU.G...........................................................AGGC.C.C..GCUGCCGGAA..CAAA.C..
Ga0115594__1212468/8-118        GGUCGGUA.C.U.GUG.UU.C......UU.A.AG.....................................................................................UGCU...........................
Ga0115594__1134035/652-542      GGUCGGUA.C.U.GUG.UU.C......UU.A.AG.....................................................................................UGCU...........................
Ga0316030__105840/623-504       AAGCUGUC.C.C.U.............AA.A........................................................................................GGGGG.G.U..UUGCAGAACA..UUGA....
Ga0316033__110175/528-409       AAGCUGUC.C.C.U.............AA.A........................................................................................GGGGG.G.G..UUGCAGAA............
Ga0228708__1128185/228-344      UGACGGUC.U.C.UCG.UU........UU..........................................................................................UAAUG.G.G..UUG.................
Ga0126318__10199518/109-204     UAGCGGUC.G.C.GCG.CU.A..................................................................................................CCAUC..........................
Ga0265745__1000697/596-785      UCUCUGUC.C.C.UGU.UA.CG.....GA.C.UC.CUGA.AGUCCUAAGGUA..U.................................................................AG.CC.A............GGU..AGU.GUG
Ga0265747__105373/294-105       UCUCUGUC.C.C.UGU.UA.CG.....GA.C.UC.CUGA.AGUCCUAAGGUA..U.................................................................AG.CC.A............GGU..AGU.GUG
Ga0265745__1001084/890-701      UCUCUGUC.C.C.UGU.UA.CG.....GA.C.UC.CUGA.AGUCCUAAGGUA..U.................................................................AGCCA.G.............GU..AGU.GUG
Ga0265745__1000512/1043-854     UCUCUGUC.C.C.UGU.UA.CG.....GA.C.UC.CUGA.AGUCCUUAGGUA..U.................................................................GGCCA..............GGU..AGU.GUG
Ga0310117__104105/1058-869      UCUCUGUC.C.C.UGU.UA.CG.....GA.C.UC.CUGA.AGUCCUUAGGUA..U.................................................................AG.CC.A............GGU..AGU.GUG
Ga0184587__113157/56-168        GCGCUGUC.C.C.UCU.GU.AG..AU.GA.A........................................................................................GCCU...........................
Ga0206356__10600373/215-21      CGUCUGUC.A.U.CAG...........GA..........................................................................................ACCU...........................
Ga0307920__126542/349-261       UUUCGGUC.C.C.AAC.UC.GCA.AG.............................................................................................................................
Ga0265742__1001154/131-376      CGUCUGUC.C.C.ACC...........CA.U.GU.UAGA.GAAUCCAACGAC..U.U.....................................GACU..A..ACGGUCUUA..UAG...ACCU....G..AGUGAU..............
Ga0307482__1127544/505-398      GGUCUGUC.C.G.CGG...........G...........................................................................................GCCAG.C........................
Ga0127503__11058765/362-444     UAGUUGUC.C.U.G.............GA.A.AG.....................................................................................CCAUC..........................
Ga0210323__1050507/418-532      GUUCGGUC.C.U.CAG.C..........U.A........................................................................................................................
Ga0242647__1002063/315-62       UGUCUGUC.C.C.CUC.AG........AG.A.AG.ACUC.AGGCCUAAGCUA..A.CGU.A..UA.......................................................GCUUC.U.G..AUACGG..............
Ga0307484__100992/499-246       UGUCUGUC.C.C.CUC.AG........AG.A.AG.ACUC.AGGCCUAAGCUA..A.CGU.A..UA.......................................................GCUUC.U.G..AUACGG..............
Ga0247534__117055/74-212        CCUCUGUC.C.U.CUG.GA........GA.G........................................................................................CUC.....................AAU.CGA
Ga0129331__1494381/292-368      CGUCGGUC.C.G...............GA.G........................................................................................CCC............................
Ga0242646__1000880/695-856      GUUCUGUC.C.C.UAC.UC.CG.....GG.G.GU.UGCC.AAACCCCAAGCA..A.CA..........A......GG.......U..GG.C............................................GAU..GUU....
Ga0180107__1174136/376-571      CGUCUGUC.C.C.CAA.GC........UU.C.UU.GGCA.GCCACCAUACUC..U.UU.............................................................GAGU...........................
Ga0307921__1050271/251-412      CAUUUGUC.C.G.CUC.CU.C......AA.A..C.......UACAUAAAGUA..G.................................................................GUAUA.C.A..AAGCAACACG..UGC.....
Ga0247524__126530/125-198       UUACGGUC.C.A.UCC.......................................................................................................................................
Ga0242646__1001007/790-609      UCACUGUC.C.C.CAC.UU.CG.....GA.A.UA.GCUC.GAAUUCCAAGAG..C.UC..............................................................GGUCG.A.G..GCCAUAU.............
Ga0213850__1280121/544-351      UGUCUGUC.C.C.CCU.GG.AC.....AA.................................................................................................................CCAAUU..CCAC.UCU
Ga0307919__1109364/247-158      UGUCGGUC.C.U.CUC.UC........A...........................................................................................................................
Ga0184600__147964/253-339       AGUCGGUC.C...CAU.CG........UA.C........................................................................................................................
Ga0316036__107710/408-322       AGUCGGUC.C...CAU.CG........UA.C........................................................................................................................
Ga0184598__138497/440-355       UGUCGGUC.C.C.AUC.GU........A...........................................................................................................................
Ga0184589__121067/502-587       UGUCGGUC.C.C.AUC.GU........A...........................................................................................................................
Ga0184601__135946/406-321       UGUCGGUC.C.C.AUC.GU........A...........................................................................................................................
Ga0115595__1093158/7-137        AGGCGGUC.U.U.CAC.UC...........G.GU.....................................................................................CCCAG.C........................
Ga0005851__1006483/274-172      GGUCGGUC.C.U.C.............GA.U.C......................................................................................UCAGA.A.G..GUUGUUC.............
Ga0247522__108409/684-554       GUCCUGUC.C.U.CUA.GG........AU.G.AA.....................................................................................GCACU.A.G..GCGAGGUU.G..CGGA....

                                <<<<.....<.<.<<<.<<.<<<.<<.............................................................................................<<<<<.<.<..<<<<<<<<<<..<<<<.<<<
                                2221.....2.2.222.22............................................................................................................2222...........................
                                ●●●C●GUC-C-●-●●●-○○--------○○-○-----------------------------------------------------------------------------------------○○○○---------------------------
```

```
Ga0310119_105137/743-673          CUGCGGUC.C.U.CUA.................................................................................................................................
Ga0247529_116306/255-362          GCGCUGUC.C.U.GGC.AC........UU.U.U........................................................................GACCU.U.C..GAU.................
Ga0316046_116376/278-384          GCGCUGUC.C.C.GGC.AC........UU.G..........................................................................AACCU.U.C..GAU.................
Ga0247534_102735/124-18           GCGCUGUC.C.U.GGC.AC........UU.G..........................................................................AGCCU.U.C..GAU.................
Ga0247540_102569/210-104          GCGCUGUC.C.U.GGC.AC........UU.G..........................................................................AGCCU.U.C..GAU.................
Ga0316037_112281/325-219          GCGCUGUC.C.U.GGC.AC........UU.U.UG.......................................................................ACCUU.C.G..A...................
Ga0184576_110594/6-113            GCGCUGUC.C.U.GGC.AC........UU.U.UG.C.....................................................................CCUUC.G.A..U...................
Ga0316049_114344/181-288          GCGCUGUC.C.U.GGC.AC........UU.U.U...............................................................GA...CCUUC.G.A..U...................
Ga0115594_1200134/154-31          GGUCGGUC.C.C.AAC.CG........CA.U.G........................................................................CCCUU.G.........................
Ga0307480_1000549/913-1064        GUACUGUC.C.C.UGC.GG........GA.G.AA.AG....................................................................CUCGA...........................
Ga0242647_1000589/378-557         GGAUUGUC.C.G.GUU...........AC......UCG.GUCCAACGCUUU..C.UCC.G...GA.U......AG.....C..CG.CGGAAU.............CCCCA...........................
Ga0222729_1000922/1729-1550       GGAUUGUC.C.G.GUU...........AC......UCG.GUCCAACGCUUU..C.UCC.G...GA.U......AG.....C..CG.CGGAAU.............CCCCA...........................
Ga0182091_1104347/98-194          UGUUUGUC.C.C.UC.............A.A..........................................................................GAAG............................
Ga0316037_128286/253-347          GGUUUGUC.C.C.UCA.........................................................................................GAGC............................
Ga0180107_1040049/1100-1010       UUUCUGUC.C.G.GC.............G............................................................................UCGC............................
Ga0242641_1014724/551-384         GUUCUGUC.U.C.AUC.UU.CC....GA.G.GU.CUAA.AACCCCGAGGCC..A.A................................................GUGC.U.G..CGAGUCCUGU..GUGC.G..
Ga0242641_1002366/499-331         GUUCUGUC.U.C.AUC.UU.CC....GA.G.GU.CUAA.AACCCCGAGGCC..A..................................................AGUGC.U.G..CGAGUCCUGU..GUGC.G..
Ga0213850_1508869/496-402         CGUCUGUC.C.U.U.............CA.A..........................................................................CUGCG.A.........................
JGI12272J11983_1072695/40-150     UCUUUGUC.C.U.CGC.........................................................................................AUCCG.U.........................
Ga0307482_1020622/600-404         CUUCUGUC.C.C.CUG.UU.C.....AG.G.UU.ACCU.AAAUUCCACGGG..U.UA................................................UG.GC.C.G..UUAACAUUGU..GUA...AG
Ga0242657_1009512/1576-1466       CCUCGGUC.C.C.ACA.........................................................................................GUCCG...........................
Ga0184577_116723/415-311          GAUCAGUC.C.C.AUU.CC.CU...................................................................................UCGGC...........................
Ga0184576_109717/432-536          GAUCAGUC.C.C.AUU.CC.CU....UC.G.GC.UACC.AACCG.........................................................................................
Ga0184572_113323/230-300          GGUCAGUC.C.C.UUU...........C.........................................................................................................
Ga0307354_155926/276-116          UGUUUGUC.C.C.UUU.GG........CA.C..........................................................................UGCUG.C.....AACU.............
Ga0265756_100433/485-286          UCGCUGUC.C.C.AGU.AG........GA.G.UA.AG....................................................................CCCGA...........................
Ga0265751_100477/874-675          UCGCUGUC.C.C.AGU.AG........GA.G.UA.AG....................................................................CCCGA...........................
Ga0180045_119797/166-305          GUUCGGUC.C.A.UAA.CC.........A.C.................................................................GCCUUU..A....GGU.G.U.G..C...GUGAG..CCCG.C..
Ga0222728_1380294/134-238         GAGCGGUC.C.U.GGG...........AC.U.AC.AG....................................................................UGGAU.U.G..CGCG................
Ga0265758_104499/242-367          CCUCGGUC.U.C.CCG.GA.AG....AG.C....CCA.CCUCUCGUGUAG..A.GGU.G...UA.........................................UGAC............................
Ga0079039_1551950/237-315         AGUUUGUC.C.A.GCG.CG......................................................................................CU..............................
Ga0157570_1034241/151-229         UGUCGGUC.C.U.GCG...........AU.G......................................................................................................
Ga0157571_1064089/212-134         UGUCGGUC.C.U.GCG...........AU.G......................................................................................................
Ga0179951_1211246/478-633         CGUCGGUC.C.A.UAA.CU........UA.G.AA.U.................................................................................................
Ga0179950_1197742/286-441         CGUCGGUC.C.A.UAA.CU........UA.G.A..............................A......G.............................................................
Ga0184578_120646/150-246          CAGCUGUC.C.A.AG.............A............................................................................CUAUU.C.U.......................
Ga0247526_105246/689-508          UCUCUGUC.U.C.UGA.UU.CG....GG.U.UU.UGCU.AAAACCCAAGGC..A.UU................................................GGUUC...A..GUGUGUCGUU..GCUC.GGG
Ga0247538_100442/1363-1181        UCUCUGUC.U.C.UGA.UU.CG....GG.A.UU.UGCU.AAAUCCCAAGGC..A.UU................................................GGUUC.A...GUGUGUCGUU...GCU.CAG
Ga0247515_107373/548-731          UCUCUGUC.U.C.UGA.UU.CG....GG.A.UU.UGCU.AAAUCCCAAGGC..A.UU................................................GGUUC.A...GUGUGUCGUU...GCU.CAG
Ga0247538_100551/721-542          UCUCUGUC.U.C.UGA.UU.CG....GG.A.UU.UGCU.AAAUUCCAAGGC..A.UU................................................GGUUC.A...GUGUGUCGUU....GC.UCA
Ga0184588_131258/156-253          GAGCGGUC.C.A.UGC...........AA.A.UC.......................................................................U.................UU..GGC...CA
Ga0316047_100860/1040-888         UGUCUGUC.C.C.GGA.CU.C......AU.C.AU........GCACUAGGC..A.AGU.G...AC.A......GG.....U..AG.C..................AACGC.U.........................
Ga0242657_1476020/160-244         GAUCUGUC.C.C.AGU...........CA........................................................................................................
Ga0184602_130623/1321-1100        AUUCUGUC.C.C.CGU.UU.CCG.GU.GC.C.UA.AAAG.CAACCGCAGGCG..A.CCU..............................................GUCUU.U.G..UACGUUCAGC....CC.UUA
Ga0153880_1410217/288-397         UAUUUGUC.C.U.CCC.AC.AGA.AG...................................................................................U.G.C..G....G.............
Ga0316049_129960/371-262          CGUCUGUC.C.C.UUG.GG........UU.G......................................................................................................
Ga0265747_125875/320-395          GUUCGGUC.C.C.CGC.GU........AG.G......................................................................................................
Ga0242647_1002473/759-521         UCUCUGUC.C.C.UGU.UA.CG....GA.C.UC.CCGA.AGUCCAUGGGUA..U...................................................AGGCU.G.G..UAAUGUGUCA..CGUC.C..
Ga0242641_1004288/32-112          GUACGGUC.C.G.CG............U.............................................................................CGAG............................
Ga0247553_101094/709-629          GUACGGUC.C.A.C.............GU............................................................................GGAU............................
Ga0127503_10814644/496-352        CCUCUGUC.C.A.UGA.UA.GG..UG.AA.A.AC.GGCA.......UAGAA..A.CC................................................GUUU............................
Ga0179939_1199779/67-205          GGUCGGUC.C.C.CGA.UU..................................................................................................................
Ga0180107_1021361/468-373         GUUCGGUC.C.C.ACA.CG........GA............................................................................UCCU............................
Ga0180107_1218358/314-219         UUACGGUC.C.C.AU.....UC.....GG.A..........................................................................GUCU............................
Ga0265758_100138/1527-1633        UGUUUGAG.U.C.GGG...........AU.U.AC.AG....................................................................UGGAU.U.G.......................
Ga0265752_100416/442-337          CGUUUGAG.U.C.GGG...........AU.U.AC.AG....................................................................UGGAU.U.G..CACC................
Ga0247534_117029/65-195           UCGCUGUC.C.U.GCU...........AA.C.CU.AACC.GAACCU.............................................................C...........................
Ga0187793_1322799/72-151          CGUCGGUC.C.U.GAG.CU..................................................................................................................
Ga0307921_1005950/791-877         GGUCGGUC.C.C.UCU.C.........AG.A.AC...................................................................................................
Ga0180047_1128081/151-64          AGUCGGUC.C.A.AGC...........AA.A.A........................................................................UCCU............................
Ga0157593_1125711/213-128         AGUCGGUC.C.A.GC............AA.A.A........................................................................UCCU............................
Ga0157569_1032488/120-205         AGUCGGUC.C.A.GC............AA.A.A........................................................................UCCU............................
Ga0157565_1077069/156-67          AGUCGGUC.C.A.AGC...........AA.A.A........................................................................UCCU............................
Ga0157593_1023702/238-314         AGUCUGUC.C.C.AGG...........CA........................................................................................................
Ga0255267_1268184/91-184          AGUCUGUC.C.C.CUC.CG.C......CU.G.U............AAU.....UC..................................................CACC............................
Ga0307919_1081825/191-74          AUUCUGUC.C.C.AAG.AU.A......C.............................................................................CUCGG.C.........................
Ga0307921_1095896/178-61          AUUCUGUC.C.C.AAG.AU.A......C.............................................................................CUCGG.C.........................
Ga0316040_172648/178-66           CAUCUGUC.C.C.AGG.AU.A......C.............................................................................GGCC............................
Ga0184577_110850/246-358          CAUCUGUC.C.C.AGG.AU.A......C.............................................................................GGCC............................
Ga0307920_113994/130-9            AUUCUGUC.C.C.AGG.AU.A......C.............................................................................GGCGG...........................
Ga0307920_113994/654-539          AUUCUGUC.C.C.AGG.AU.A......C.............................................................................GGC.............................
Ga0307921_1037919/342-457         AUUCUGUC.C.C.AGG.AU.A......C.............................................................................GGC.............................
Ga0242646_1001980/23-280          GGUCUGAC.C.C.CCG.CG.U...UG...G.G.........................................................UUC..CGA...AGUUA.ACG..UUAAGGAAUA..CCCA.CGC
Ga0179935_1139075/165-78          CCAUUGUC.C.U.CGA.CU..................................................................................................................
Ga0179944_1246019/249-162         UCAUUGUC.C.U.AAG.AC.U................................................................................................................
Ga0180044_1069332/269-187         CGACUGUC.C.G.UGU.GC........A.........................................................................................................
Ga0157564_1065218/271-353         CGACUGUC.C.G.UGU.GC........A.........................................................................................................
Ga0180044_1047678/444-368         CGACUGUC.C.C.AGG.UU..................................................................................................................
Ga0180047_1061805/548-622         GGACUGUC.C.C.AGA.GU..................................................................................................................
Ga0157569_1073490/184-258         GGACUGUC.C.C.AGA.GU..................................................................................................................
Ga0213850_1394829/253-105         AGUCUGUC.C.U.UCA.GU.........U.G.UA.UUAU.AUACAAUACGGC..C..................................................GC........CUGA....AG..CAGU.ACU
Ga0255293_1138691/440-213         GAGUGGUC.U...CGC.AC.AUU.CG.GA.C.GA.GCAU.ACGUUACAGUCU..C..................................................UU....U.U..GAACGGACAG..G.......
Ga0222728_1000079/1192-1299       CUUUUGUC.C.U.CCA.GU.C......................................................................................CG...........................
Ga0081246_1201717/153-259         UUUCUGUC.C.C.ACA.GU........................................................................................CC...........................
Ga0222728_1216887/51-157          CUUCGGUC.C.U.CCA.GU........................................................................................CC...........................
Ga0265754_1141843/290-185         AGUUUGUC.C.C.AGA.GU.C......................................................................................C............................
JGI12272J11983_1345183/364-258    UUUCUGUC.C.C.ACA...........G.............................................................................UCC.............................
Ga0242647_1005934/451-617         UCUCUGUC.U.C.CAU.CG.CG....GU.U.AC.CACA.GACCUUGGUAAG..G.CC................................................GGGUA.A.....GGUGUCACU..UGUA.C..
Ga0115594_1187657/870-960         UGUCUGUC.C.U.ACC.AA........UA........................................................................................................

                                  <<<<.....<.<.<<<.<<.<<<.<<...............................................................................<<<<<.<.<..<<<<<<<<<<..<<<<.<<<
                                  2221.....2.2.222.22......................................................................................2222.............................
                                  ●●●C●GUC-C-●-●●●-○○--------○○-○----------------------------------------------------------------------------○○○○-----------------------------
```

```
Ga0115594_1174394/642-729      UGUCUGUC.C.U.ACC.AA........UA.........................................................................................................................
Ga0184569_112764/943-836       AGUUGGUC.C.C.UCA.GU.CC......G..........................................................................................UGUG...........................
Ga0265750_1013321/389-496      UAUUUGUC.C.C.AGA.GU.CC......G..........................................................................................UGUG...........................
Ga0265757_139522/177-70        UAUUUGUC.C.C.AGA.GU.CC......G..........................................................................................UGUG...........................
Ga0316029_116217/368-261       AGUUGGUC.C.C.UCA.GU.C......C..........................................................................................................................
Ga0316047_100016/2681-2574     AGUUGGUC.C.C.UCA.GU.C......C..........................................................................................................................
Ga0256352_1101982/155-277      UGUCUGUC.C.C.UCU.C.........GA.G.AG.CA..................................................................................AUCUC.C........................
Ga0256350_1100736/283-405      UGUCUGUC.C.C.UCU.C.........GA.G.AG.CA..................................................................................AUCUC.C........................
Ga0079039_1443516/199-38       CGUCGGUC.C.U.UUC.CA.GG.....CU.......CCA.CCCCUUCCAAAGGUG.UA.............................................................GUUUU.G.......UGGCC............
Ga0115595_1072788/334-441      GGUCGGUC.C.U.AGG...........AU.U.AG.....................................................................................UGCG...........................
Ga0115594_1022316/346-239      GGUCGGUC.C.U.AGG...........AU.U.AG.....................................................................................UGCG...........................
Ga0129331_1058147/189-99       GUUCUGUC.C.C.CAU.AU........AA.G.......................................................................................................................
Ga0265751_100127/176-103       AUACGGUC.C.U.GA............UG.........................................................................................................................
Ga0307921_1043977/91-164       UAUUUGUC.C.A.A.............CA...........................................................................................U.............................
Ga0307919_1048575/242-90       CAUUUGUC.C.G.CUC.CU.CA.....AA..........................................................................................CACGC.G....AGAUGCGUGG..........
Ga0242646_1001086/737-571      AAUCUGUC.C.A.CGU.UU.GC.....GG.A.UU.CCUG.AAAUCCUAAGGU..A.U..............................................................GGCCA.G....GUAGUAUGCU..UGGA.GAG
Ga0265752_111922/418-343       UUUCUGUC.C.G.CGU.UU........GU.........................................................................................................................
Ga0157562_1134247/69-160       GUUCUGUC.C.C.UCU...........AU.G.A......................................................................................AGCCC..........................
Ga0157559_1047848/128-41       GUUCGGUC.C.C.UCU...........AU.G.A......................................................................................AGCCC..........................
Ga0179944_1313268/129-232      GUUCGGUC.C.U.C...GA........UG.A.AA.C...................................................................................CCCU...........................
Ga0213850_1259428/445-342      GUUCUGUC.C.U.CUA...........UG.A........................................................................................AGCCC.C........................
Ga0153880_1167367/224-121      GUUCUGUC.C.U.CUA...........UG.A........................................................................................AGCCC.C........................
Ga0153880_1058095/344-241      GUUCUGUC.C.U.CUA...........UG.A........................................................................................AGCCC.C........................
Ga0179943_1178349/195-298      GUUCGGUC.C.U.C.............GA.U.GA.....................................................................................AACCC.C........................
Ga0307921_1075885/188-36       CAUUUGUC.C.G.CUC.CU.UA.....AA.C.G........................................................................GCCUUG..CGCCGGCCA............................
Ga0307921_1062541/180-338      CAUUUGUC.C.G.CUC.CC.CAU.AACAA.A.GC.GUCA.AAACGCUUUAGC..G................................................................CCA............................
Ga0242641_1014474/336-257      CCACGGUC.C.C.AUC.CG...................................................................................................................................
Ga0307919_1064641/225-73       CAUUUGUC.C.G.CUC.CU.UA.....AA.C.G........GCCUUGCGCCG..G................................................................CCACA.A........................
Ga0265759_108120/277-351       GGUCUGUC.U.C.ACU...........UU.C.......................................................................................................................
Ga0265753_1005277/856-1057     UCUCUGUC.C.G.CGC.GU.UA..AG.....................................................................................................A..CUCCCUCAGU..CUUC.GGG
Ga0247523_120120/325-403       UAGCGGUC.C.G.CAU.GU........G..........................................................................................................................
Ga0228707_1100139/344-194      UCACUGUC.U.C.AUC...........UU.U.AG....................................................................................................................
Ga0316049_154526/155-74        UCUCUGUC.C.C.GG............UA.C.U......................................................................................UAUU...........................
Ga0316035_177575/224-142       UCUCUGUC.C.C.GG............UA.C.......................................................................................................................
Ga0307920_126403/177-486       CUUUUGUC.C.G.CUU.UC.UAA.UA.AC.A.UA.CGUA.CUCACCCAAGGU..G.UGU.C...UU.C.......GG..............................CUCU..CGA...CU..U.U.U..CAGACGAGAG..ACUG.GGG
Ga0138280_1140856/341-479      GGUCGGUC.C...GGA.GG.CGC.GG.UG.C.........................................................................................AUU...........................
Ga0247539_101233/414-526       UCGCUGUG.G.U.UUA.UG........GU.A.AA.....................................................................................GCC............................
Ga0184569_108885/200-88        UCGCUGUG.G.U.UUA.UG........GU.A.AA.....................................................................................GCC............................
Ga0247521_112559/317-205       UCGCUGUG.G.U.UUA.UG........GU.A.AA.....................................................................................GCC............................
Ga0184599_154888/232-155       CUGCGGUC.C.G.G..............A.U........................................................................................GGGU...........................
Ga0180108_1143860/199-116      CUGCUGUC.A.G.G.A.GA.UU.....AG.G.......................................................................................................................
Ga0247524_121352/374-303       UAGCGGUC.C.G.G.............A...........................................................................................UC.............................
Ga0206356_11002907/329-148     UGUCUGUC.A.U.AGG.CU........GG.A........................................................................................CCCUC..........................
Ga0265743_103007/946-842       AUUCAGUC.C.U.CUA.UU.C......G............................................................................UGCAUUA..GCA...AUCC...........................
Ga0316033_101029/151-49        GGGCGGUC.C.G.GAU.GG........GA.C..A.GAAG.A..............................................................................GAUCG..........................
Ga0179937_1126086/216-325      GGUCGGUC.C.G.UAG.CG.CG................................................................................................................................
Ga0307482_1050048/176-95       AUGUGGUC.C.G.GGG.C.......................................................................................................CCC.C........................
JGI12272J11983_1319142/230-159 GUGCGGUC.G.G.GGG......................................................................................................................................
Ga0180107_1285918/13-113       UAGCGGUC.C.G.GGU.AG.GG.................................................................................................GUCUC.G.G..UAACAC..............
Ga0265756_108694/382-277       CUCCUGUC.C.A.AGG.AG.CGU.AA............................................................................................................................
Ga0265746_1140777/214-319      CUCCUGUC.C.A.AGG.AG.CGU.AA............................................................................................................................
Ga0184574_101359/409-493       CAGCGGUC.C.U.UCA.C....................................................................................................................................
Ga0184568_108556/391-307       CAGCGGUC.C.U.UCA.C....................................................................................................................................
Ga0316030_107573/391-475       CAGCGGUC.C.U.UCA.C....................................................................................................................................
Ga0180107_1031754/139-57       CAUCUGUC.C...CUA.AC.A.................................................................................................................................
Ga0307919_1029784/253-144      CCACUGUC.C.A.UG............A...........................................................................................CGUA...........................
Ga0307921_1042549/343-452      CCACUGUC.C.A.UG............A...........................................................................................CGUA...........................
Ga0307482_1021658/484-563      AAGCGGUC.C...UAC.AC....................................................................................................UUC............................
Ga0179943_1102267/215-90       GGUCGGUC.C.G.UCG...........G...........................................................................................GGCAU.C.A..CAGCG...............
Ga0079074_1279626/114-274      UGUUUGUC.C.C.UUU...........GG.C.AC.UGCU.GCAACUUUGCAC..A.UCU.G...UU.U.......G...........................................AGGCC.C.G..CUGCCGGAAC..AAAC.CAG
Ga0307921_1019702/90-184       UAUCUGUC.C.C.GGA...........CA.A.A......................................................................................CUGCU..........................
Ga0307920_133961/233-140       UAUCUGUC.C.C.GGA...........CA.C........................................................................................CUGCU..........................
Ga0316035_179807/270-184       CAGCGGUCCU.G.C.CG..........UG.A.......................................................................................................................
Ga0256338_1343513/152-228      CCUCGGUC.C.U.CAC...........UU.A.......................................................................................................................
Ga0255268_1432700/127-51       CCUCGGUC.C.U.CAC...........UU.G.......................................................................................................................
Ga0153880_1067318/492-566      ACUCUGUC.C.C.GUA......................................................................................................................................
Ga0247533_103075/417-305       CCUCGGUC.C.C.GUC.GG.G......AA.C.U......................................................................................CGCCC.U........................
Ga0247541_102964/56-168        CCUCGGUC.C.C.GUC.GG.G......AA.C.U......................................................................................CGCCC.U........................
Ga0184589_110648/1041-881      UUUCUGUC.C.C.UCC.UU.CG.....GG.G.GU.GCCA.AACCCCAAGCAC..A................................................................AGCUG.............CCA..U.CC.AU.
Ga0184580_124121/239-79        UUUCUGUC.C.C.UAC.UC.CG.....GG.G.GU.GCCA.AACCCCAAGCAC..A................................................................AGCUG.............CCA..U.CC.AU.
Ga0184598_101324/530-690       UUUCUGAC.C.C.UAC.UC.CG.....GG.G.GU.GCCA.AACCCCAAGCAC..A................................................................AGCUG.............CCA..U.CC.AU.
Ga0184588_120624/266-426       AAUCUGUC.C.C.UAU.UU.CG.....GG.G.GU.GCCA.AACCCCAAGCAC..A................................................................AGCUG.............CCA..U.CC.AU.
Ga0184579_106194/996-1156      AGUCUGUC.C.C.UAU.UU.CG.....GG.G.GU.GCCA.AACCCCAAGCAC..A................................................................AGCUG.............CCA..U.CC.AU.
Ga0265758_100254/356-516       AGUCUGUC.C.C.UAU.UU.CG.....GG.G.GU.GCCA.AACCCCAAGCAC..A................................................................AGCUG.............CCA..U.CC.AU.
Ga0184597_106861/196-36        AAUCUGUC.C.C.UAU.UU.CG.....GG.G.GU.GCCA.AACCCCAAGCAC..A................................................................AGCUG.............CCA..U.CC.AU.
Ga0184600_132066/568-408       AGUCUGUC.C.C.UAU.UU.CG.....GG.G.GU.GCCA.AACCCCAAGCAC..A................................................................AGCUG.............CCA..U.CC.AU.
Ga0316031_108320/216-376       AAUCUGUC.C.C.UAU.UU.CG.....GG.G.GU.GCCA.AACCCCAAGCAC..U................................................................AGCUG.............CCA..U.CC.AU.
Ga0184596_109261/979-1139      CGUCUGUC.C.C.UAC.UU.CG.....GG.G.GU.GCCA.AACCCCAAGCAC..A................................................................AGCUG.............CCA..U.CC.AU.
Ga0316035_107544/354-514       CGUCUGUC.C.C.UAC.UU.CG.....GG.G.GU.GCCA.AACCCCAAGCAC..A................................................................AGCUG.............CCA..U.CC.AU.
Ga0247527_115808/280-440       CGUCUGUC.C.C.UAC.UU.CG.....GG.G.GU.GCCA.AACCCCAAGCAC..A................................................................AGCUG.C............CA..U.CC.AU.
Ga0247519_107918/217-58        AAUCUGUC.C.C.UAC.UU.C......GG.G.GG.UGCC.AAACCCCAAGCA..C.A..............................................................AGCUG.A...........ACU..GCGA.U..
Ga0184589_112135/40-200        UAUCUGUC.C.C.UAC.UU.CG.....GG.G.GU.GCCA.AACCCCAAGCAC..A................................................................AGCUG.A...........ACU..GCGA.U..
Ga0247551_100195/490-329       UUUCUGUC.C.C.UAC.UU.CG.....GG.G.GU.GCCA.AACCCCAAGCAC..A................................................................AGCUG.A...........ACC..GCGA.U..
Ga0247526_117235/152-313       UUUCUGUC.C.C.UAC.UU.CG.....GG.G.GU.GCCA.AACCCCAAGCAC..A................................................................AGCUG.A...........ACC..GCGA.U..
Ga0326631_101563/544-705       AGUCUGUC.C.C.UAC.UU.CG.....GG.G.GU.GCCA.AACCCCAAGCAC..A................................................................AGCUG.A...........ACC..GCGA.U..
Ga0184596_111007/525-686       AGUCUGUC.C.C.UAC.UU.CG.....GG.G.GU.GCCA.AACCCCAAGCAC..A................................................................AGCUG.A...........ACU..GCGA.U..
Ga0184573_110190/115-276       AAUCUGUC.C.C.UAC.UU.CG.....GG.G.GU.GCCA.AACCCCAAGCAC..A................................................................AGCUG.A...........ACU..GCGA.U..
Ga0316033_144939/167-328       AGUCUGUC.C.C.UAC.UU.CG.....GG.G.GU.GCCA.AACCCCAAGCAC..A................................................................AGCUG.A...........ACU..GCGA.U..
Ga0247552_101742/612-452       AAUCUGUC.C.C.UAC.UU.CG.....GG.G.GU.GCCA.AACCCCAAGCAC..A................................................................AGCUG.A............CC..GCCA.U..

                               <<<<.....<.<.<<<.<<.<<<.<<.............................................................................................<<<<<.<.<..<<<<<<<<<<..<<<<.<<<
                               2221.....2.2.222.22....................................................................................................2222...........................
                               ●●●C●GUC-C-●-●●●-○○--------○○-○----------------------------------------------------------------------------------------○○○○---------------------------
```

```
Ga0247545_112960/183-343           AAUCUGUC.C.C.UAC.UU.CG.....GG.G.GU.GCCA.AACCCCAAGCAC..A................................................................AGCUG.A............CC..GCAA.U..
Ga0247541_103784/123-283           AAUCUGUC.C.C.UAC.UU.CG.....GG.G.GU.GCCA.AACCCCAAGCAC..A................................................................AGCUG.A............CC..GCAA.U..
Ga0247524_111427/50-210            AAUCUGUC.C.C.UAC.UU.CG.....GG.G.GU.GCCA.AACCCCAAGCAC..A................................................................AGCUG.A............CC..GUCA.U..
Ga0247545_104341/406-246           AAUCUGUC.C.C.UAC.UU.CG.....GG.G.GU.GCCA.AACCCCAAGCAC..A................................................................AGCUG.A............CC..GCCA.U..
Ga0184602_114416/11-171            AAUCUGUC.C.C.UAC.UU.CG.....GG.G.GU.GCCA.AACCCCAAGCAC..A................................................................AGCUG.A............CC..GCCA.U..
Ga0316038_111670/11-171            AAUCUGUC.C.C.UAC.UU.CG.....GG.G.GU.GCCA.AACCCCAAGCAC..A................................................................AGCUG.A............CC..GCCA.U..
Ga0184586_133850/368-528           AAUCUGUC.C.C.UAC.UU.CG.....GG.G.GU.GCCA.AACCCCAAGCAC..A................................................................AGCUG.A............CC..GCCA.U..
Ga0184600_123882/188-28            AAUCUGUC.C.C.UAC.UU.CG.....GG.G.GU.GCCA.AACCCCAAGCAC..A................................................................AGCUG.A............CC..GUCA.U..
Ga0184574_121290/269-109           AAUCUGUC.C.C.UAC.UU.CG.....GG.G.GU.GCCA.AACCCCAAGCAC..A................................................................AGCUG.A............CC..GCCA.U..
Ga0316032_107575/215-55            AAUCUGUC.C.C.UAC.UU.CG.....GG.G.GU.GCCA.AACCCCAAGCAC..A................................................................AGCUG.A............CC..GUCA.U..
Ga0247519_110701/607-447           AAUCUGUC.C.C.UAC.UU.CG.....GG.G.GU.GCCA.AACCCCAAGCAC..A................................................................AGCUG.A............CC..GUCA.U..
Ga0247526_110221/603-443           AAUCUGUC.C.C.UAC.UU.CG.....GG.G.GU.GCCA.AACCCCAAGCAC..A................................................................AGCUG.A............CC..GCCA.U..
Ga0247529_134913/224-64            AAUCUGUC.C.C.UAC.UU.CG.....GG.G.GU.GCCA.AACCCCAAGCAC..A................................................................AGCUG.A............CC..GUCA.U..
Ga0247529_103231/270-430           AAUCUGUC.C.C.UAC.UU.CG.....GG.G.GU.GCCA.AACCCCAAGCAC..A................................................................AGCUG.A...........ACU..GCGA.U..
Ga0184580_114961/306-146           AAUCUGUC.C.C.UAC.UU.CG.....GG.G.GU.GCCA.AACCCCAAGCAC..A................................................................AGCUG.A...........ACU..GCGA.U..
Ga0184603_126764/80-240            AAUCUGUC.C.C.UAC.UU.CG.....GG.G.GU.GCCA.AACCCCAAGCAC..A................................................................AGCUG.C...........ACU..GCGA.U..
Ga0247550_114736/167-7             AAUCUGUC.C.C.UAC.UU.CG.....GG.G.GU.GCCA.AACCCCAAGCAC..A................................................................AGCUG.A...........ACU..GCGA.U..
Ga0184603_111918/241-82            AAUCUGUC.C.C.UAC.UU.CG.....GG.G.GU.GCCA.AACCCCGAGCAC..A................................................................AGCUG.A...........ACU..GCGA.U..
Ga0247538_100424/120-281           AAUCUGUC.C.C.UAC.UU.CG.....GG.G.GU.GCCA.AACCCCAAGCAC..A................................................................AGCUG.A...........ACU..GCGA.U..
Ga0247547_102464/189-29            AGUCUGUC.C.C.UAC.UU.CG.....GG.G.GU.GCCA.AACCCCAAGCAC..A................................................................AGCUG.A...........CUG..C.CG.U..
Ga0247551_100828/546-387           AGUCUGUC.C.C.UAC.UU.CG.....GG.G.GU.GCCA.AACCCCAAGCAC..A................................................................AGCUG.A...........CUG..C.CG.U..
Ga0247538_100480/788-947           AAUCUGUC.C.C.UAC.UU.CG.....GG.G.GU.GCCA.AACCCCAAGCAC..A................................................................AGCUG.A...........CUG..C.CG.U..
Ga0247550_100850/456-297           AAUCUGUC.C.C.UAC.UU.CG.....GG.G.GU.GCCA.AACCCCAAGCAC..A................................................................AGCUG.A...........CUG..C.CG.U..
Ga0247536_101914/360-201           AGUCUGUC.C.C.UAC.UU.CG.....GG.G.GU.GCCA.AACCCCAAGCAC..A................................................................AGCUG.A...........CUG..C.CG.U..
Ga0247553_110698/526-367           AGUCUGUC.C.C.UAC.UU.CG.....GG.G.GU.GCCA.AACCCCAAGCAC..A................................................................AGCUG.A...........CUG..C.CG.U..
Ga0153880_1114976/150-383          GAUCUGUC.C.................GA.U.CC.CACC.ACCCCCCCCACA..A.UU....................................................G..CGA...GUACA.C.A..AUGACUCAAA..C.......
Ga0180052_189627/240-346           GGUCGGUC.C.C.AUC.AU.CC..AC.............................................................................................CGCGG.C.U......................
Ga0184598_136114/144-251           UUGUUGAG.C.U.GGG...........AU.U.AC.AG..................................................................................UGGAU.U.G..C...................
Ga0222728_1011988/841-947          AUGUUGAG.C.U.GGG...........AU.U.AC.AG..................................................................................UGGAU.U.G..CGCU................
Ga0265751_160438/108-215           UGUCAGUC.C.A.C.............AG.U.GAGCACA.AACGCUCACA.....................................................................ACCGC.G........................
Ga0247514_107497/1071-1140         GAGCAGUC.C.C.UC........................................................................................................_...............................
Ga0180107_1129325/244-60           GUUCUGUC.C.C.UUG.UG...G.AG.UC.U.GC.UCAA.GACUCAGGCAGC..G.UU..............................................................UUCAG.U.U..AUGACUCG............
Ga0242646_1002014/11-270           GGUCUGUC.C.U.UAU.CC.U......AU.A.CU.CCCU.CACCCCGAACAG..A...........A.A.......GA......G..GG.GAG...........................CUGCC.C....ACCCAUUAAA..GUGG.AAC
Ga0222728_1008526/24-283           GGUCUGUC.C.U.UAU.CC.U......AU.A.CU.CCCU.CACCCCGAACAG..A...........A.A.......GA......G..GG.GAG...........................CUGCC.C....ACCCAUUAAA..GUGG.AAC
Ga0222728_1008391/1281-1023        GGUCUGUC.C.U.UAU.CC.U......AU.A.CU.CCCU.CACCCCGAACAG..A...........A.A.......GA......G..GG.GAG...........................CUGCC.C....ACCCAUUAAA..GUGG.AAC
Ga0265759_100017/4211-4288         ACGCAGUC.C.C.AU..CA.U..................................................................................................  C............................
Ga0247512_104440/911-1082          CUUCUGUC.C.C.CAU.CA.C......UA..........................................................................................UGUUC.U.G..CAUCUG..............
Ga0265756_131580/159-58            CUCCUGUC.C.C.AGA.AC.UA.....UA.......................................................................................... A.............................
Ga0310119_169621/253-177           GGACGGUC.C...CCC.AU........CC.U................................................................................................................
Ga0247516_124175/202-272           UAGCGGUC.U.A.CAC.......................................................................................................................................
Ga0247534_100772/309-234           GAACGGUC.C.G.U.............A...........................................................................................CAAC...........................
Ga0222728_1054736/119-193          UAGCGGUC.C.C.UAC.AA....................................................................................................................................
Ga0180106_1297567/444-515          CAGCGGUC.C.C.UAG.GA.C..................................................................................................................................
Ga0242647_1000066/2638-2719        AAACGGUC.C.C.CUA.CU....................................................................................................CUCU...........................
Ga0307484_104189/90-171            UUACGGUC.C.G.ACG.AA.C......C...........................................................................................................................
Ga0316048_109289/45-149            AAGUUGAG.C.U.GGG...........AU.U.AC.AG..................................................................................UGGAU.U.G..CACA................
Ga0265750_1038125/531-427          AAGUUGAG.C.U.GGG...........AU.U.AC.AG..................................................................................UGGAU.U.G..CACA................
Ga0247537_109516/130-26            AAGUUGAG.C.U.GGG...........AU.U.AC.AG..................................................................................UGGAU.U.G..CACA................
Ga0310115_103434/744-848           AAGUUGAG.C.U.GGG...........AU.U.AC.AG..................................................................................UGGAU.U.G..CACA................
Ga0307482_1009758/1126-1021        UUGUUGAG.C.U.GGG...........CA.A.CU.G...................................................................................UGGAU.U.G..CAUCG...............
JGI12272J11983_1356304/1548-1654   CUGUUGAG.C.U.GGG...........AC.A.AC.CG..................................................................................UGGAU.U.G..CAUCG...............
JGI12272J11983_1072695/645-539     CGAUUGAG.C.U.GGG...........AU.A.AC.UG..................................................................................UGGAU.U.G..CAUC................
Ga0242657_1009512/820-926          AGAUUGAG.C.U.GGG...........UA.A.AC.UG..................................................................................UGGAU.U.G..CAUC................
Ga0316037_172473/89-196            UCGCUGAG.C.C.GGG...........UA.A.AC.UG..................................................................................UGGAU.U.A..CACGA...............
Ga0184592_121084/378-271           CUGUUGAG.C.C.GGG...........UA.A.AC.UG..................................................................................UGGAU.U.A..CACGA...............
Ga0184569_113518/504-609           UUGUUGAG.C.C.GGG...........AA.A..................................................................................CGG...UGGAU.U.G..CACG................
Ga0184602_114837/392-286           UUGUUGAG.C.U.GGG...........CA.A.AC.AG..................................................................................UGGAU.U.G..CACGA...............
Ga0316030_102131/386-280           UUGUUGAG.C.U.GGG...........UA.A.AC.AG..................................................................................UGGAU.U.G..CACGG...............
Ga0310118_104334/336-443           CUGUUGAG.U.C.GGG...........UU.A.AC.UG..................................................................................UGGAU.U.A..CAGAU...............
Ga0180108_1146973/462-263          UUGUUGUC.C.G.GAG.GA........UA.A.AG.....................................................................................UGGGC.U.U..UUGCCUGCAG..AGGG.GUC
Ga0184580_111164/678-749           CAGCGGUC.C.C.CUC.......................................................................................................................................
Ga0242647_1006852/526-598          UAGCGGUC.C.C.CAG...........UG.U........................................................................................................................
Ga0179939_1059820/107-216          GGUCGGUC.C.A.ACA.GC........UU..........................................................................................................................
Ga0115594_1156252/268-370          UGUCUGUC.C.G.UAC.AA.AU.....AA.G........................................................................................GGC............................
Ga0115594_1006122/120-29           UGUCUGUC.C.U.ACC...........AA..........................................................................................................................
Ga0115595_1180708/226-317          UGUCUGUC.C.U.ACC...........AA..........................................................................................................................
Ga0115594_1211214/299-389          CGUCUGUC.C.G.CAC...........AA..........................................................................................................................
Ga0115594_1007070/120-30           UGUCUGUC.C.G.UAC...........A...........................................................................................................................
Ga0115595_1181384/176-86           UGUCUGUC.C.G.UAC...........A...........................................................................................................................
Ga0115594_1166236/119-19           UGUCUGUC.C.G.UAC...........AA..........................................................................................................................
Ga0115594_1157594/155-254          UGUCUGUC.C.G.UAC.AA.AU.....AA.A........................................................................................GGC............................
Ga0256352_1182139/180-81           UGUCUGUC.C.G.UAC.AA.AU.....AA.G........................................................................................GGC............................
Ga0115594_1182529/183-84           UGUCUGUC.C.G.UAC.AA.AU.....AA.A........................................................................................GGC............................
Ga0265746_1064626/389-458          CGUCUGUC.C.U.ACC.......................................................................................................................................
Ga0115594_1016490/326-410          UGUCUGUC.C.G.CAC.AC.CG.....A...........................................................................................................................
Ga0115595_1007417/328-412          UGUCUGUC.C.G.CAC.GC.CG.....A...........................................................................................................................
Ga0228708_1103570/188-285          GGUCGGUC.C.G.ACU.GU....................................................................................................GUCG...........................
Ga0265751_121301/189-266           CGUCAGUC.C.G.GUG.UU....................................................................................................................................
Ga0179944_1005311/261-382          GGUCGGUC.C.U.UCC.GG........G...........................................................................................CAUCA.C.A..G...................
Ga0179944_1191552/284-411          CGUCGGUC.C.A.UCG.GG........G...........................................................................................CAUCA.C.A..G...................
Ga0179943_1150468/216-89           GGUCGGUC.C.U.UCA.GG........G...........................................................................................CAUCA.U.U..GCU.................
Ga0179944_1204933/216-89           GGUCAGUC.C.U.UCA.GG........G...........................................................................................CAUCA.U.U..GCU.................
Ga0307482_1020425/440-614          GAUCUGUC.C.C.UAG.UU.UG...G.GU.A.UU.UGUC.AAAUACCAAGAC..A.UC..............................................................AGCUC.A....CUCAUAUGCG..........
Ga0242646_1001395/255-509          CGUCUGUC.C.C.CAC.CA.UC.....UG.U.GC.UACC.GAUGUUAACGUU..............A.A.......GG......U..AGACACACG...C...ACCGCCCAACCCGA...CCUCA.A.G..GGAAUUCGUA..GAGG.A..
Ga0184578_112031/156-44            CAGCUGUG.G.U.UGG.AU........GG.U.......................................................................AAA..............GCCU...........................
Ga0184577_120131/220-108           CAGCUGUG.G.U.UGG.AU........GG.U.......................................................................AAA..............GCCU...........................
Ga0247517_121471/387-499           CAGCUGUG.G.U.UGG.AU.G......GU.A.AA.....................................................................................GCC............................
Ga0247539_107237/153-229           CUACGGUC.C...CUA.AU.AU.....C...........................................................................................................................
Ga0265758_109576/83-157            AGGCUGUC.C.C.ACC.AU........GC.C........................................................................................................................

                                   <<<<.....<.<.<<<.<<.<<<.<<.............................................................................................<<<<<.<.<..<<<<<<<<<<..<<<<.<<<
                                   2221.....2.2.222.22....................................................................................................2222...........................
                                   ●●●C●GUC-C-●-●●●-○○--------○○-○--------------------------------------------------------------------------------------○○○○----------------------------
```

```
Ga0184577_107529/254-327  GUGCGGUC.CAG.GUU.AU..................................................................................................................................
Ga0213850_1246156/171-47  CCUCUGUC.C.C.U.............GA.C.A.....................................................................................GGG.A.G....UUGCUG..............
Ga0326631_172366/284-207  CUUCUGUC.C.A.UGU...........UA...................................................................................CGA..................................
Ga0310114_149639/68-153  CGUCGGUC.C.C.AUC.AC.C................................................................................................................................
Ga0184599_128731/188-273  CGUCGGUC.C.C.AUC.AC.C................................................................................................................................
Ga0310119_131358/533-447  CGUCGGUC.C.C...............AU.........................................................................................CACC...........................
Ga0247526_116548/279-354  AUGCGGUC.G.G.AGG.AU........C.........................................................................................................................
Ga0247519_113845/334-409  AUGCGGUC.G.G.GAG.AU........C.........................................................................................................................
Ga0247529_111070/303-228  AUGCGGUC.G.G.GAG.AU........C.........................................................................................................................
Ga0184580_116530/249-177  CUGCGGUC.C.A.GAC...........AU........................................................................................................................
Ga0247524_105844/561-637  UAGUGGUC.C.A.GA............CC.A.......................................................................................GU.............................
Ga0184593_119849/313-238  UAGCGGUC.C.A.G.............G..........................................................................................GUAC...........................
Ga0184599_140898/774-849  UAGCGGUC.C.A.G.............G..........................................................................................GUAC...........................
Ga0316049_111814/256-181  UAGCGGUC.C.A.G...GG.UG.....CA........................................................................................................................
Ga0316034_100493/254-174  UAGCGGUC.C.A.GGU.GU........AU.A.......................................................................................GU.............................
Ga0184600_118565/177-97  UAGCGGUC.C.A.GGU.GU........AU.A.......................................................................................GU.............................
Ga0184602_122864/595-515  UAGCAGUC.C.A.GGG.GU........C..........................................................................................AUGUA..........................
Ga0184596_126546/83-162  UAGCGGUC.C.A.GA.............G.G.A.....................................................................................AUGUA..........................
Ga0184597_129399/366-446  UAGCGGUC.C.A.GGG.GU.GU.....AG........................................................................................................................
Ga0184576_117703/380-460  UAGCGGUC.C.A.GGG.GU.GU.....AG........................................................................................................................
Ga0184590_111002/143-223  UAGCGGUC.C.A.GA............GA.U.......................................................................................AUAU...........................
Ga0247551_103383/322-242  UAGCGGUC.C.A.GAG.GU.AU....................................................................................................A.G........................
Ga0184597_111165/262-182  UAGCGGUC.C.A.GGG.GU.....................................................................................................AUA.G........................
Ga0184570_116436/278-198  CAGCGGUC.C.A.GGG.GU.....................................................................................................AUA.G........................
Ga0316037_169124/279-199  CAGCGGUC.C.A.GGG.GU.....................................................................................................AUA.G........................
Ga0247543_103170/244-164  UAGCGGUC.C.A.GAG............A.U.......................................................................................AUAG...........................
Ga0184589_120451/249-329  UAGCGGUC.C.A.GAG............A.U.......................................................................................AUAG...........................
Ga0247519_108375/286-206  UAGCGGUC.C.A.GGG.GU...................................................................................................AUAG...........................
Ga0184587_105985/71-151  UAGCGGUC.C.A.GGG.GU........CA.U......................................................................................................................
Ga0184593_101086/682-762  UAGCGGUC.C.A.GAG.GU........CA........................................................................................................................
Ga0184602_121948/324-245  CAGCGGUC.C.G.U.............GG.........................................................................................CUGUA..........................
Ga0184597_110829/1090-1011  CAGCGGUC.C.G...............UG.G.......................................................................................CUGUA..........................
Ga0184578_129447/314-235  CAGCGGUC.C.G.U.............GG.........................................................................................CUG............................
Ga0184588_113246/331-252  CAGCGGUC.C.G...............UG.G.......................................................................................CUG............................
Ga0316051_1003245/138-56  CAGCGGUC.C.A.GGG.CG.....................................................................................................U............................
Ga0316046_122965/221-142  CAGCGGUC.C.A.GGG.CG.....................................................................................................U............................
Ga0184600_105920/81-160  CAGCGGUC.C.GUGGC.UG..................................................................................................................................
Ga0184603_125382/9-88  CAGCGGUC.C.A.GG.......................................................................................................GUAUA..........................
Ga0247550_100138/208-129  CAGCGGUC.C.A.G.............G..........................................................................................GUAUA..........................
Ga0316049_122425/209-288  CAGCGGUC.C.A.G.............GG.C.......................................................................................GU.............................
Ga0032354_1062690/2659-2586  AUGCGGUC.C...CCC.UG.CA...............................................................................................................................
Ga0179955_1048341/123-51  GGUCGGUC.C.G.GU......................................................................................................................................
Ga0138277_1023917/182-18  GGUCGGUC.C...AGA.GG.CGC.GC.AU.C.AC....................................................................................GUCUG.A.U..GUAU................
Ga0138282_1020546/737-573  GGUCGGUC.C...AGA.GG.CGC.GC.AU.C.AC....................................................................................GUCUG.A.U..GUAU................
Ga0242641_1105635/265-351  CGUCGGUC.C.A.GUG.UC........A.........................................................................................................................
Ga0242654_10795375/263-349  CGUCGGUC.C.A.GUG.UC........A.........................................................................................................................
Ga0247554_113506/254-161  UAACGGUC.C.C.AUA.UG........UC.C.A.....................................................................................AUGG...........................
Ga0265745_1000618/793-552  CAUCUGUC.C.C.UGU.UA.CG.....GA.C.UU.CGAA.GUCCAUAGAUGA..A.....G...GC.A.......GU......U..GG.CAGUGC...U...AUCACAGC........GGCCA.U.G..CUGGAUGAUG..UUU...CC
Ga0265742_1001052/322-80  CAUCUGUC.C.C.UGU.UA.CG.....GA.C.UU.UCGA.AGUCCAUGGAAA..A...A.G...GC.A.......GU......U..GG.CAGUGC...U...AUCACAGC........GGCCA.C.G..CUGGAUGAUG..UUU...CC
Ga0310113_106868/972-730  CAUCUGUC.C.C.UGU.UA.CG.....GA.C.UU.UCGA.AGUCCAUUGAAA..A...A.G...GC.A.......GU......U..GG.CAGUGC...U...AUCACAGC........GGCCA.C.G..CUGGAUGAUG..UUU...CC
Ga0265750_1009219/272-30  CAUCUGUC.C.C.UGU.UA.CG.....GA.C.UU.UCGA.AGUCCAUUGAAA..A...A.G...GC.A.......GU......U..GG.CAGUGC...U...AUCACAGC........GGCCA.C.G..CUGGAUGAUG..UUU...CC
Ga0265740_1001378/1084-842  CAUCUGUC.C.C.UGU.UA.CG.....GA.C.UU.UCGA.AGUCCAUUGAAA..A...A.G...GC.A.......GU......U..GG.CAGUGC...U...AUCACAGC........GGCCA.C.G..CUGGAUGAUG..UUU...CC
Ga0265752_100504/930-688  CAUCUGUC.C.C.UGU.UA.CG.....GA.C.UU.UCGA.AGUCCAUUGAAA..A...A.G...GC.A.......GU......U..GG.CAGUGC...U...AUCACAGC........GGCCA.C.G..CUGGAUGAUG..UUU...CC
Ga0265747_100653/495-253  CAUCCGUC.C.C.UGU.UA.CG.....GA.C.UU.UCGA.AGUCCAUUGAAA..A...A.G...GC.A.......GU......U..GG.CAGUGC...U...AUCACAGC........GGCCA.C.G..CUGGAUGAUG..UUU...CC
Ga0265757_104669/135-376  CAUCUGUC.C.C.UGU.UA.C......GG.A.CU.UCCA.AGUCCAUAGAUG..A...A.G...GC.A.......GU......U..GG.CAGUGC...U...AUCACAGC........GGCCA.U.G..CUGGAUGAUG..UUU...CC
Ga0265742_1088739/51-292  CAUCUGUC.C.C.UGU.UA.C......GG.A.CU.UCCA.AGUCCAUAGAUG..A...A.G...GC.A.......GU......U..GG.CAGUGC...U...AUCACAGC........GGCCA.U.G..CUGGAUGAUG..UUU...CC
Ga0265747_100509/1277-1036  CAUCUGUC.C.C.UGU.UA.C......GG.A.CU.UCCA.AGUCCAUAGAUG..A...A.G...GC.A.......GU......U..GG.CAGUGC...U...AUCACAGC........GGCCA.U.G..CUGGAUGAUG..UUU...CC
Ga0265752_100314/1466-1225  CAUCUGUC.C.C.UGU.UA.C......GG.A.CC.UCCA.AGUCCAUAGAUG..A...A.G...GC.A.......GU......U..GG.CAGUGC...U...AUCACAGC........GGCCA.U.G..CUGGAUGAUG..UUU...CC
Ga0213850_1450246/323-606  CAUCUGUC.C.U.CCC.CC.C.U.G.............................................................................................GGAAG.C.G..CACCGCCCAA..AAC....U
Ga0157593_1101573/285-478  UAGCUGUC.C.C.CCA.CA........CG.U.CC.CACA.GGAUG.........................................................................GGCG...........................
Ga0222728_1000125/1315-1238  UCGCAGUC.C.U.CUA.CC.AU..................................................................................................C............................
Ga0242654_10000586/1273-1196  UCGCAGUC.C.U.CAU.GC.AC.....AU........................................................................................................................
Ga0265756_100130/1310-1239  UAGCGGUC.C.C.AGC.AU..................................................................................................................................
Ga0307482_1008742/1212-1288  UCGCAGUC.C.C.CGC.AU........UU........................................................................................................................
Ga0242654_10040657/1018-942  CCGCAGUC.C.C.UAC.GC.A.......A........................................................................................................................
Ga0307484_100842/1194-1271  ACGCAGUC.C.C.UAU.GC.A......C..U......................................................................................................................
Ga0307482_1009223/670-508  UGACUGUC.C.C.CAC.UU.CGG.GU....U.GC.UCAA.AGCCCAAGAGCA..C...............................................................GGUUG.A.G..GCC.................
Ga0307484_108094/186-350  UGACUGUC.C.C.CAC.UU.CGG.GU.UU.G.CU.CAAA.GCCCAAGAGCAC..................................................................GGUUG.A.G..GCC.................
Ga0247514_114593/280-211  AGGCAGUC.C.C.UC......................................................................................................................................
Ga0184583_106441/296-227  UAGCUGUC.C.C.CAC.....................................................................................................................................
Ga0242654_10672177/244-165  AUGCGGUC.C.C.ACU...........A..........................................................................................GCC............................
Ga0126318_10229384/609-688  CUGCGGUC.C.A.GCA.AC.UU...............................................................................................................................
Ga0247551_122551/29-115  CUGCGGUC.C.A.AUA.UA...........A.U.....................................................................................AGGGG.G........................
Ga0316048_107802/239-324  CUGCGGUC.C.A.AUA.UA...........A.U.....................................................................................AGGGG.G........................
Ga0242641_1002064/1203-1116  AUGCUGUC.C.A.GCU.UC....................................................................................................UCAG.C.G......................
Ga0307482_1026216/884-813  AUGCGGUC.C...CUA.UC..................................................................................................................................
Ga0307482_1636410/270-341  AUGCGGUC.C...CUA.UC..................................................................................................................................
Ga0307482_1016877/168-97  AUGCGGUC.C...CCA.UC..................................................................................................................................
Ga0180107_1106188/424-495  AUGCGGUC.C...GCC.GA..................................................................................................................................
Ga0307482_1000354/2063-1987  AUGCGGUC.C...GCA.GC........AU.G......................................................................................................................
Ga0184587_107783/167-95  AUGCGGUC.C.U.GGU.GG..................................................................................................................................
Ga0184585_128240/467-390  GUGCGGUC.C...UCC.AC.AA...............................................................................................................................
Ga0184596_102741/625-701  AUACGGUC.C...CCU.AU.G......UC........................................................................................................................
Ga0247539_100086/1763-1850  GGUCGGUC.C.C.CUU.AU.CAU.GG..A........................................................................................................................
Ga0184584_115572/267-354  GGUCGGUC.C.C.CUU.AU.CAU.GG..A........................................................................................................................
Ga0184579_105257/1735-1648  GGUCGGUC.C.C.CUU.AU.CAU.GG..A........................................................................................................................
Ga0307482_1023270/273-197  UUGCGGUC.C...GCC.CC.A................................................................................................................................
Ga0307482_1219330/19-95  GUGCGGUC.C.C.CAU.AC.C................................................................................................................................
Ga0242657_1207391/462-383  GUGCGGUC.C.A.CAC.CA.UA...............................................................................................................................

                          <<<<.....<.<.<<<.<<.<<<.<<............................................................................................<<<<<.<.<..<<<<<<<<<<..<<<<.<<<
                          2221.....2.2.222.22...................................................................................................2222...........................
                          ●●●C●GUC-C-●-●●●-○○--------○○-○---------------------------------------------------------------------------------------○○○○---------------------------
```

```
Ga0247520_104849/891-821       GUACGGUC.C.C.AUC......................................................................................................................................
Ga0247531_104861/1082-1012     GUGCGGUC.C.C.AUC......................................................................................................................................
Ga0247518_137824/301-231       GUGCGGUC.C.C.AUC......................................................................................................................................
Ga0307482_1202605/470-395      CGUCGGUC.C...CCU.UG.C.................................................................................................................................
Ga0307921_1021783/160-261      AUUCUGUC.C.C.GU............AC.A.A......................................................................................GCGCU.U........................
Ga0242657_1054283/541-620      GUGCUGUC.C.G.G.............CA.G.......................................................................................................................
Ga0242647_1005188/439-360      GUGCUGUC.C.G.G.............CA.G.......................................................................................................................
Ga0153880_1391790/62-140       GUUCUGUC.C.C.GGU...........UA.G.......................................................................................................................
Ga0153880_1379927/66-144       GUUCUGUC.C.C.GGU...........UA.G.......................................................................................................................
Ga0307482_1023665/853-778      CGUCUGUC.C.C.ACA.GU........G..........................................................................................................................
Ga0242646_1047905/92-182       GGUCUGUC.U.C.UC..UG........UA.U.AU.CA..................................................................................GCC............................
Ga0307921_1024041/406-334      UUUCUGUC.C.C.GUA......................................................................................................................................
Ga0307919_1039507/513-440      UUUCUGUC.C.C.G.............UA.C.......................................................................................................................
Ga0307919_1084008/308-378      AUUCUGUC.C.C.G.............UA.C.......................................................................................................................
Ga0247525_114510/199-318       UGUCUGUC.C.C.GAA.C.........UA.C.AU.CAGG.GUAUG..........................................................................CAACA.G.G..ACCUUCC.............
Ga0184603_107262/477-596       UGUCUGUC.C.C.GAA.C.........UA..............CAUCAGGGU..A.UG.............................................................CAACA.G.G..ACCUUCC.............
Ga0184585_151549/495-376       UGUCUGUC.C.C.GAA.C.........UA..............CAUCAGGGU..A.UG.............................................................CAACA.G.G..ACCUUCC.............
Ga0310119_113172/381-306       CGUCGGUC.C.C.AUU.CC.C.................................................................................................................................
Ga0310114_133004/56-132        CGUCGGUC.C.C.AUU.CC.C.................................................................................................................................
Ga0184603_148109/996-920       CGUCGGUC.C.C.AUU.CC.C.................................................................................................................................
Ga0184599_110049/275-349       CGUCGGUC.C.C.AUU.CC.C.................................................................................................................................
Ga0184587_132427/565-485       CGUCGGUC.C.C.UUU.CC.C.................................................................................................................................
Ga0265756_135986/152-290       GUUUUGUC.C.C.GCG.CC........AA.A......CA.A..............................................................................CCCGC.U........................
Ga0247519_104852/800-730       GUGCGGUC.C.G.G.........................................................................................................CCC............................
Ga0213850_1274705/160-417      CAUCUGUC.C.A.UAC.CG.AGC.CG..........CUU.GAGAUCAAGCUG..A.CU.............................................................GCCCC.A.A..UUUCGGCGUG..AGAC.CCC
Ga0265747_100454/1395-1324     UAGCGGUC.C.G.UUA...........A..C.......................................................................................................................
Ga0247519_118271/334-405       UAGCGGUC.C.G.G........................................................................................................................................
Ga0242654_10135580/213-336     CAGUAGUC.C.A.GCA.AU........GA.C.UA.UG..................................................................................UGCGC.C.U..AUCG................
Ga0184576_110410/277-348       CUGCGGUC.C.A.GGG.GU...................................................................................................................................
Ga0247535_105396/277-348       CUGCGGUC.C.A.GGG.GU...................................................................................................................................
Ga0265758_104558/436-365       UAGCGGUC.C...UCC.CU...................................................................................................................................
Ga0247526_117664/434-499       CAGCGGUC.C.A.UG.......................................................................................................................................
Ga0316037_123461/289-368       AAGCGGUC.C.G.U.............C...........................................................................................AUGU.C........................
Ga0247543_100863/642-721       AAGCGGUC.C.G.U.............U...........................................................................................AUGUC..........................
Ga0247521_124040/388-309       AAGCUGUC.C.G.U.............U...........................................................................................AUGUC..........................
Ga0184589_120335/11-90         AAGCUGUC.C.G.U.............U...........................................................................................AUGUC..........................
Ga0247518_108423/758-837       AAGCGGUC.C.G.U.............U...........................................................................................AUGUC..........................
Ga0247531_104608/1256-1177     AAGCGGUC.C.G.U.............U...........................................................................................AUGUC..........................
Ga0265750_1365815/134-55       AAGCGGUC.C.G.U.............C...........................................................................................AUGU.C........................
Ga0184597_121517/262-183       AAGCGGUC.C.G.U.............C...........................................................................................AUGU.C........................
Ga0265747_102820/448-376       GAGCGGUC.C.C.UUC......................................................................................................................................
Ga0247554_105388/388-316       GAGCGGUC.C.C.UUC......................................................................................................................................
Ga0307482_1015330/1021-1093    UAGCGGUC.C.G.GA............AA.G.......................................................................................................................
Ga0184600_102725/60-135        UCGCGGUC.C.C.CAA.CA........CU.........................................................................................................................
Ga0184602_122974/190-265       UCGCGGUC.C.C.CAA.CA.C.................................................................................................................................
Ga0316038_112263/442-356       UUGCGGUC.C.U.CC............A...........................................................................................GGCC...........................
Ga0307484_161007/136-39        UAGCGGUC.C.A.UGG...........AG.A.GG.GGUA.AACCCCCUCAGU...................................................................GGA............................
Ga0307482_1033688/516-422      UAGCGGUC.C.A.UG............AG.G.GG.GGUU.AACCCC........................................................................................................
Ga0316031_132099/178-294       UCUCGGUC.C.C.UUG.AC.C..................................................................................................AUCGC.C.G..CCGGGCUGGC..CCG.....
Ga0213850_1220553/385-463      GGACGGUC.C.C.ACC.AU........AA.G.U.....................................................................................................................
Ga0180107_1307618/257-174      CGUCUGUC.C.G.CUC.UU...................................................................................................................................
Ga0265747_119090/8-84          UGUCGGUC.C.G.CGC.UU...................................................................................................................................
Ga0242646_1115622/191-109      CAGCGGUC.C.A...............CG.G........................................................................................AUUGU.G........................
Ga0184570_114412/209-338       GGUUGGUC.C.U.UCA.AU.CAG.AG.............................................................................................AU.............................
Ga0265757_143567/145-70        CGUUUGUC.C.U.GCU......................................................................................................................................
Ga0184568_109812/391-283       CGUCUGUC.C.U.A.............AA..........................................................................................CACU...........................
Ga0184568_104528/922-1000      UGUCUGUC.C.U.AC............GU.G.......................................................................................................................
                               <<<<.....<.<.<<<.<<.<<<.<<.............................................................................................<<<<<.<.<..<<<<<<<<<<..<<<<.<<<
                               2221.....2.2.222.22....................................................................................................2222...........................
                               ●●●C●GUC-C-●-●●●-○○--------○○-○----------------------------------------------------------------------------------------○○○○---------------------------
```

alignment positions 151 · · · 300

```
Ga0247541_102964/376-269       A.CA............CUU.AU................................................................................................................................
Ga0182091_1018330/100-226      ................GCA.AU................................................................................................................................
Ga0316035_118720/195-42        U.A.............AAG.GU.UC.UA.G.....A.....AGCCU........................................................................................................
Ga0179944_1246019/41-131       ................GUA.A.................................................................................................................................
Ga0265747_125480/96-202        ................AUA.GU.CA.AA.G.....C.....A............................................................................................................
Ga0265757_115889/161-280       ................UUU.C.................................................................................................................................
Ga0180107_1031754/4443-4551    ................AAG.U.................................................................................................................................
Ga0180107_1229893/426-318      .................CC.GC.....................................................CUGC..GG..G.........UGGUUAG.....A.UCAAGUGAU................................
Ga0222728_1038816/522-635      ................UUC.AA.GA.UC..........................................................................................................................
Ga0179955_1195671/481-563      ................UAA...................................................................................................................................
Ga0265751_100560/274-382       ..................................................................................G..A.........CGGAUAU.....A.CCAAGUG..................................
Ga0265751_124015/160-14        ................AUU.GC................................................................................................................................
Ga0316029_122532/20-172        ................AGA.A.................................................................................................................................
Ga0316031_106349/204-348       ................AGA.A.................................................................................................................................
Ga0265759_136872/100-7         .......A.UAUCCA.UUU.GU.UG.G...........................................................................................................................
Ga0138291_1027114/229-62       U.CCUUGG..UUCAC.UAU.AU................................................................................................................................
Ga0138291_1177333/356-213      .....UGA..GUCCC.UAG.AU................................................................................................................................
Ga0079074_1152161/13-203       ...........................................UUUUUU.CCUUGGAUUA.CUAUUG..G.GAGUUCC...CC..A.........GUAAUUU.....A.GACCGGUGAA..AACCC........GGC...A..ACCCAUU
Ga0242657_1353977/135-311      ................UAA.UG.AU.AA.G.....A.....AGAGUUAC.GCUUGCGAUG.AUGCAA..G.CAGCUAC...GA..A.........UUGAUUU.....A.UAGUGGU..............................AGUC
Ga0255293_1180932/373-295      ................UUA.U.................................................................................................................................
Ga0307482_1147486/107-4        ................AAG.C.................................................................................................................................
Ga0242647_1001226/1011-1115    ................UUG.CA................................................................................................................................
Ga0316032_128094/136-241       U.CU............AUG.U.................................................................................................................................

                               <.<<<<<<.<<<<<<.......................................................................................................................................
                               ......................................................................................................................................................
                               ----------------●●●-●○--------------------------------------------------------------------------------------------------------------------------------
```

```
Ga0184598_118707/299-404       C.C...........A.AUG.U.................................................................................................................................
Ga0213850_1470830/321-202      ................GGA.AC................................................................................................................................
Ga0307482_1003156/2075-2177    ................U.....................................................................................................................................
Ga0307482_1000291/592-698      ................UAA.AU................................................................................................................................
Ga0247532_117056/43-239        ................UGG.U......................UGUGUG.GUCUUGAAUG.ACCCAG..G.UGGCUCU...GC..UGUCUGCAAGAUGAAUU.....G.ACAUCUU.................................C
Ga0184582_120088/374-570       ................UGG.UU.GU.GU.G.....G.....UCUUGAAU.GACCCAGGUG.GUUCUA..C.UGUCUGC...AA..G.........AUGAAUU.....G.ACAUCUUCCG..AC...........................
Ga0265742_1013613/579-453      A.AGAUGU.CGAGUG.GGC.U.................................................................................................................................
Ga0265748_110851/279-405       A.AGAUGU.CGAGUG.GGC.U.................................................................................................................................
Ga0307482_1000262/4292-4390    ................AAG.C.................................................................................................................................
Ga0307484_100453/182-280       ................AAG.C.................................................................................................................................
Ga0247538_120078/87-186        ......................................................................................................................................................
Ga0307484_109934/368-484       ................UCU.A.................................................................................................................................
Ga0184572_111224/187-319       ................GCA.A.................................................................................................................................
Ga0265744_102237/225-365       ................AGG.CA.................................................UGGCCUG...GG..U.........GAGAAGU.....A.AUAGCUU..................................
Ga0265758_100384/785-1040      ................UGC.GU.UU.CU.G.....A.....ACCCGAUA.GCAAAUCGGG.GUGCUC..U.UCCCACC...CU..C.........GUGGUGU.....C.CAGCCCUUCC..UGCGA........GGA...A..GGAACUG
Ga0265753_1005391/776-521      ................CGU.UU.CU.GA.A.....C.....CCGAUAGC.AAAUC..GGG.GUGCUC..U.UCCCACC...CU..C.........GUGGUGU.....C.CAGCCCUUUC..UGAGU........GAA...A..GGAACUG
Ga0265759_100623/780-1036      ................CGU.UU.CU.GA.A.....C.....CCGAUAGC.AAAUCGGGGU.GCUCUU..C.CCACCCU...CG..U.........GGUGUCC.....A.GCCCUUCCUG..UAACG........GAA...G..GAACUGC
Ga0247535_113123/229-119       ................UA....................................................................................................................................
Ga0316036_112404/482-376       ................GUA.C.................................................................................................................................
Ga0247515_120448/91-178        ................UAA.CU................................................................................................................................
Ga0247518_120275/202-289       ................UAA.CU................................................................................................................................
Ga0307482_1000311/1469-1363    ...........ACCC.AAG.U.................................................................................................................................
Ga0184603_150346/629-518       .................UG.CG.UG.UG.U.....U.....AACGUAAU.CCCAAG..............................................................................................
Ga0307484_102855/507-659       ................ACU.UG.CC.GG..........................................................................................................................
Ga0316029_101396/632-732       ................GAG.C.................................................................................................................................
Ga0184582_100659/424-334       ................UUU.A.................................................................................................................................
Ga0265754_1040230/51-148       ................UUG.AU..A.AA.A.....U.....AU...........................................................................................................
Ga0316039_120091/354-252       ................AAG.GG................................................................................................................................
Ga0307921_1050750/452-358      ................UCU.AC................................................................................................................................
Ga0265759_141590/154-258       ................AUG.GA................................................................................................................................
Ga0184602_130775/1275-1431     ................UC....................................................................................................................................
Ga0213850_1052387/326-212      ................UGC.CA................................................................................................................................
Ga0213850_1393213/656-542      ................UGC.CA................................................................................................................................
Ga0184578_112042/249-140       ................CUC.CU.UU.AG.G.....A.....GAAUUUCA.A...................................................................................................
Ga0316033_100036/207-98        ................CUC.CU.UU.AG.G.....A.....GAAUUUCA.A...................................................................................................
Ga0247534_100007/116-225       ................CUC.CU.UC.GG.G.....G.....GAAUUUCA.A...................................................................................................
Ga0184569_109084/220-111       ................CUC.CU.UC.GG.G.....G.....GAAUUUCA.A...................................................................................................
Ga0206355_1134705/241-332      ................AAU.GU................................................................................................................................
Ga0265754_1007706/448-552      ................AUC.CU..............................................................................AU.....A.UCAAGUG..................................
Ga0213850_1056422/14-139       ................CUC.U.................................................................................................................................
Ga0180107_1033011/786-679      ................CUC.U.................................................................................................................................
Ga0307483_1000720/1178-1064    ................AAG.CG................................................................................................................................
Ga0265755_100088/1451-1216     .................................................................GG..G.ACCCCCC...UA..C................................................................
Ga0310119_125223/193-319       ................UGA.CG................................................................................................................................
Ga0265754_1037910/322-179      ..................A.UG.GC.........................UUAGCGGUGG.AAUCA....................................................................................
Ga0316053_162144/16-125        .....................................................U................................................................................................
Ga0242654_10007932/343-135     ................CUU.AG.CG.GG.G.....A.....AUGGCCCU.GUAAAGUGGG.AUUAAC..U.CCUAUCC...UU..G.........CUUACCG.....A.ACCCGGAGAA..UGGGG........CGA...A..ACUCCUC
Ga0153880_1315932/142-46       ................GUG.GU.......A........................................................................................................................
Ga0265750_1009277/794-905      ................AUG.GU................................................................................................................................
Ga0265756_100928/447-557       ................AUG.GU................................................................................................................................
Ga0265745_1004075/525-659      ................AUG.GU................................................................................................................................
Ga0138291_1147840/370-201      U.CUGUGG........AUC.UC.UA.UA.U.....G.....AGUAUCCG.A...................................................................................................
Ga0138291_1027295/231-62       U.CUGU.......GG.AUC.UC.UA.UA.U.....G.....AGUAUCCG.A...................................................................................................
Ga0184602_109114/416-317       ................CUA.GA................................................................................................................................
Ga0153880_1354682/355-454      ................CUA.GA................................................................................................................................
Ga0242649_1016743/464-237      .................................................................CU..C.UGGUGGG...UA..U.........CGGUAGC.....G.AACCGAUAUG..UGUCU........AAA...U..GAUACGA
Ga0179943_1118543/65-150       ................GGG.AA................................................................................................................................
Ga0184568_108419/492-395       ................AAG.U.................................................................................................................................
Ga0265756_117960/316-418       ................AUG.GU................................................................................................................................
Ga0265759_100202/139-258       ................CCG.AA................................................................................................................................
Ga0265748_107452/280-387       ................ACC.CG.AA.AG.G.....G.....AGU..........................................................................................................
Ga0307484_149222/136-234       ................UAG.CA................................................................................................................................
Ga0316038_107364/55-195        ................AUU.U.................................................................................................................................
Ga0184593_123391/157-297       ................AUU.U.................................................................................................................................
Ga0242652_1159268/284-150      ................GAA.A.........................................................................................GACG....................................
Ga0310117_113093/724-511       ..........................................GACUCCU.CUGA..........GAU..C.CGGUACG...GC..A.........GCGAUCC.....U.AAGGCACGUA..AAGCU........GG..............
Ga0179944_1119724/70-159       ................ACU.CA.CA.UA..........................................................................................................................
Ga0316036_104840/706-578       C.U.............UUG.AA................................................................................................................................
Ga0247538_106858/350-156       ........................................................AACG.CAGCUA..C.CUGCAGG...GC..U.........ACUCGCC.....C.UCACCGUGCA..A............................
Ga0180107_1215312/308-184      ................CCA.A.................................................................................................................................
Ga0247534_100003/3147-3245     ................AUA.GA.GU.UA.G.....C.....AA...........................................................................................................
Ga0247519_101377/1717-1907     U.C.............UUC.G.................................................................................................................................
Ga0184588_143621/642-452       U.C.............UUC.G.................................................................................................................................
Ga0265758_100017/4028-4134     ................AUU.A.................................................................................................................................
Ga0265751_102572/231-125       ................AUU.A.................................................................................................................................
Ga0310106_100226/1503-1738     G.AC............CCC.CC.UA.UA.U.....G.....AUCUGCGA.CCAAUGUACG.UACCGG..C.AGAGG..........................................................................
Ga0184596_112078/314-191       ................UUU.GU.AU.U...........................................................................................................................
Ga0247528_108647/438-562       ................UUU.GU.AU.U...........................................................................................................................
Ga0184602_108471/49-173        ................UUU.GU.AU.U...........................................................................................................................
Ga0316031_112736/216-74        ................CUC.UC................................................................................................................................
Ga0184575_106581/174-318       ................CUC.UC................................................................................................................................
Ga0184582_129189/472-619       .......................................................GUAGA.CUGUUG..U.CUGCAGU...CC..U.........UAGCCUA.....A.AGGAGUG..................................
Ga0180052_116799/419-501       ................CUG.GU.AA.UA.A.....U.....A............................................................................................................
Ga0179955_1013331/439-311      ................ACU.CA.AA.UA..........................................................................................................................
Ga0157569_1058012/474-586      ......................................................................................................................................................
Ga0157562_1164332/672-560      ................GCA.UU................................................................................................................................
Ga0265746_1084801/20-158       ................AUC.AA................................................................................................................................
Ga0265752_102479/223-81        ................AUC.AA................................................................................................................................
Ga0265756_107066/520-660       ................AUC.AA................................................................................................................................

                               <.<<<<<<.<<<<<<.......................................................................................................................................
                               ......................................................................................................................................................
                               ----------------●●●-●○--------------------------------------------------------------------------------------------------------------------------------
```

```
Ga0247541_102884/37-209      ....................AC.AG.CA.C.....C.....AGUGGCCA.CUAUUGUGUU.CAGCUG..G.AAACAGG...CU..C................................................................
Ga0247516_125012/316-428     ................AGG.GU.UC.AA..........................................................................................................................
Ga0213850_1071966/331-222    ................CUU.UU.UA.AA.G.....C.....AA...........................................................................................................
Ga0257141_1100114/291-412    ................UGC.UA................................................................................................................................
Ga0265740_1020297/298-150    ................AUA.CU...........................................UG..A.AAGCUCG...AG..A.........UUUUUUA.....C.UCAAAAGCUG..AAUAG........AUG...G..GAUG...
Ga0307482_1064439/425-316    ................UUC.CA.UC.G...........................................................................................................................
Ga0138292_1031932/14-100     ................AUA.A.................................................................................................................................
Ga0138291_1022124/340-254    ................AUA.A.................................................................................................................................
Ga0184582_127657/238-384     ................AGC.G.................................................................................................................................
Ga0184570_113141/244-341     ................AAU.GA................................................................................................................................
Ga0310119_152175/242-389     ................U.....................................................................................................................................
Ga0184576_119948/531-664     U.CU............UCA.U.................................................................................................................................
Ga0316034_129624/151-284     U.CU............UCA.C.................................................................................................................................
Ga0307482_1134687/287-181    C.UU............AAG.U..G..............................................................................................................................
Ga0307484_104189/399-292     ................AAG.U.................................................................................................................................
Ga0138282_1242808/325-475    .........................................AGUAUUUC.C...................................................................................................
Ga0180047_1081041/176-42     ................UUU.G.................................................................................................................................
Ga0265752_100023/1618-1523   ................AAG.A.................................................................................................................................
Ga0242649_1051170/34-143     ................CAC.GC.C..............................................................................................................................
Ga0242654_10030584/1049-1243 A.CUCUC....CCCU.GUA.........................................................CC...AU..U.........GUGAUCU.....U.AUCGGUUUCC..AGUAC........AUC...G..GA.....
Ga0307484_102966/727-825     ................AG....................................................................................................................................
Ga0307921_1020571/133-221    ................UUA.U..U..............................................................................................................................
Ga0184573_113483/125-229     ................UGC.UA................................................................................................................................
Ga0184576_116834/47-151      ................UGC.UA..A.............................................................................................................................
Ga0184595_109216/770-611     .......................................................................................................................................CU...G..GUUACUC
Ga0316040_154359/245-85      ......................................................................................................................................ACU...G..GUUACUC
Ga0184595_116949/555-395     U.CA............CUC.UU.GC.CA.A.....G.....AGUUUCCU.AUCCA...............................................................................................
Ga0247553_104889/634-563     ................AUU.U.................................................................................................................................
Ga0316046_107255/463-562     ................CAG.AU.U..............................................................................................................................
Ga0307484_102693/441-627     U.CGGCUA..GCCC..AUC.CU.U...........................................................................CUG.....U.AAUUCCCGUG..AGGGA........GAA...G..GAGUAAG
Ga0184568_114257/261-413     ................AGG.CC.GA.AU.G.....G.....CGCGACCU.UU..................................................................................................
Ga0316047_106478/13-108      ................CUA.UG................................................................................................................................
Ga0242654_10024002/775-526   ................UAC.CC.AC.CU.A.....C.....CGUAUAAU.AUGGUGACGU.AAGGCA..G.CCCCAAC...AA..A.......................AAUUGUUAGA..GAUUC........CGU...U..GUUAAUC
Ga0316039_101269/650-841     ................AUA.GA.CU.GG.G.....A.....ACCAUCUU.GCAUGGUAUA.CGUAAU..C.CCAUUGU...AG..A.........AAGUCGC.....A.CCCAGAGAUG..GGAGG........UG..............
Ga0265743_102316/615-423     ................AUA.GA.CU.GG.G.....A.....ACCAUCUU.GCAUGGUAUA.CGUAAU..C.CCGUUGU...AG..A.........AAGUCGC.....A.CUCCAGUGUG..GAGA.........................
Ga0210323_1072445/691-543    A.C.............CUC.G.................................................................................................................................
Ga0224712_10069322/1099-1357 ................GCG.CC.CA.AA.U.....G.....CAACUCCG.AUAAGGACCU.CAUUGU..A.UAAAUCG...GC............AAACCUG.....G.GAACGGGUGG..AGCCG........GUA...U..GGGACCU
Ga0213850_1371453/186-298    ................UCC.UU................................................................................................................................
Ga0153880_1056505/290-430    ................UUG.A.................................................................................................................................
Ga0179940_1033563/296-410    ................GUA.A.................................................................................................................................
Ga0265743_143797/241-325     ................AGC.GA................................................................................................................................
Ga0242654_10006748/25-247    U.UC............CAG.GA........................................AACUC..G.AGAUCCC...AC..U.........CCUAUGA.....A.AUCCGGUUGA..AAGCC........GGA.............
Ga0138278_1149859/65-166     ................UUG.AC................................................................................................................................
Ga0138278_1149142/191-91     ................UUC.AU................................................................................................................................
Ga0138277_1147417/463-362    ................UUG.AC................................................................................................................................
Ga0138278_1065026/314-214    ................UUA.UG................................................................................................................................
Ga0138277_1089473/408-304    ................UUC.AU................................................................................................................................
Ga0138277_1022592/399-305    ................UUC.AC................................................................................................................................
Ga0157559_1099797/360-209    ................CCA.GU................................................................................................................................
Ga0157572_1089974/360-209    ................CCA.GU................................................................................................................................
Ga0242646_1007472/516-317    .....CUG.ACACUG.AUG.GU................................................................................................................................
Ga0184583_104740/342-477     U.C.............AGU.UA................................................................................................................................
Ga0242654_11312609/202-83    ................UUG.AA................................................................................................................................
Ga0184568_109070/428-286     C.C.............AUA.AA................................................................................................................................
Ga0224712_10002011/1564-1454 ................AAG.U.................................................................................................................................
Ga0222728_1000095/4027-4133  ................GUC.C.................................................................................................................................
Ga0307482_1068632/529-339    ..........AACCG.AAU.AC.CC.............................................................................................................................
Ga0157560_1170105/128-245    .................UC.GA.UA.............................................................................................................................
Ga0316031_107021/319-214     ................GUG.C...............................GUCGGCAG.AACCAA..G.CGGUUAG........................................................................
Ga0316047_100015/734-626     ................CUC.AA.UC.............................................................................................................................
Ga0247533_100162/1643-1535   ................UGG.GC.GU.AU.G.....C.....CUAAUCCA.AG..................................................................................................
Ga0180108_1209503/1932-2029  ................AGC.A.................................................................................................................................
Ga0265753_1009881/576-819    ....AGCA.AUACC..AGG.CU.AG.AU.G.....U.....AG.................................AG...AG..C.........CGUAUGC.....A.CUAUUUGGUA..AGUCA........UCC...U..AAGCCCC
Ga0265741_101522/997-754     ...UAGCA.AUACC..AGG.CU.AG.AU.G.....U.....AGAGAGCC.GUAUGC......GCUAU..U.UGG.........................................UAAG..UCAUC........CUA...A..GCCCCUC
Ga0184583_103998/587-740     C.C.............CAU.G.................................................................................................................................
Ga0247521_125910/353-214     ................ACU.CU.UU.UU.A.....C.....AA...........................................................................................................
Ga0184569_106253/218-80      C.GUCUAC.CCACCUCACU.CU.UU.UU.A.....C.....AA...........................................................................................................
Ga0316039_119781/347-457     ................GUG.CG.UU.UA.G.....C.....AUAAUCCC.AA..................................................................................................
Ga0184572_112077/359-259     ......................................................................................................................................................
Ga0257141_1118781/153-31     ................UGC.UA................................................................................................................................
Ga0257136_1059838/549-429    ................UGC.UA................................................................................................................................
Ga0138283_1149501/173-59     ..................A.AG.CC.GA.G.....A.....AUUUAAUC.UC..................................................................................................
Ga0184597_126105/545-362     ................UGG.GA.AA.GA.G.....G.....AACUCUCC.CCCGAGAC.G.CUCGAG..A.AAAGAAC...UC..U.........UGGCUGA.....C.ACA......................................
Ga0213850_1245299/581-409    ................ACA.AA.AG.UU.G.....C.....GA.........................................................................AUC..AUGUC........UGG...C..GUCCGUA
Ga0179948_1254202/198-296    ................ACG.UA.CU.AA.U.....A..................................................................................................................
Ga0180108_1093556/523-685    .................UG.GG.AU.GG.A.....C.....CGAAGCCC.AUCUGGGUGU.AGCGCG..U.UCACUCA...GC..C................................................................
Ga0180107_1321405/371-533    .............................G.....C.....CGAAGCCC.AUCUGGGUGU.AGCGCG..U.UC.............................................................................
Ga0242641_1001197/544-746    G.CCCUGG..UGACG.CGG.GG................................................................................................................................
Ga0184597_115944/155-80      ................UGU.AA................................................................................................................................
Ga0184595_100702/414-262     C.AACCCG.UCUCC..CCA.U.................................................................................................................................
Ga0184601_118759/683-531     C.AACCCG.UCUCC..UCA.U.................................................................................................................................
Ga0184592_125105/67-219      C.AACCCG.UCUCC..UCA.U.................................................................................................................................
Ga0257141_1089020/433-224    ................UUC.AU.GG.AU.A.....A.....AGCAUACU.CUCAUAUCGG.CUUAAG..C.CGGUGGC...AU..G.........GGUACGU.....U.UAAACAUGUG..UAGAG........UGC...A..GAGGUUC
Ga0265743_103007/262-368     ................UUG.CA.UC.AA.U........................................................................................................................
Ga0222728_1000095/4541-4435  ................UUG.CA................................................................................................................................
Ga0247537_109082/42-148      ................CAG.UG.GA.UU.G.....C.....AUCAAAUG.UCGAUGU.............................................................................................
Ga0247533_100162/1117-1223   ................CUG.UG.GA.UU.G.....C.....AUCAAAUG.UCGAUGU.............................................................................................
Ga0265757_100063/1489-1724   ................AAG.GU.UU.AA.A.....C.....GUGAGAGC.GUAGGGAGCU.CCACAG..CCCAGCUUG...GC..U.........GGGACCCCCCUAC.AUGACCUGCG..ACCAA........UGU...A..CGUACCG
Ga0265747_118257/34-269      ................UUA.AA.CG.UG.A.....G.....AGCGUAGGGAGCUCCACAG.CCCAGC..U.UGGCUGG...GA..C.........CCCCCUA.....C.AUGACCUGCG..ACCAA........UGU...A..CGUACCG

                             <.<<<<<<.<<<<<<.......................................................................................................................................
                             ......................................................................................................................................................
                             ----------------●●●-●○--------------------------------------------------------------------------------------------------------------------------------
```

```
Ga0265745_1000046/1698-1933    ................UUA.AA.CG.UG.A.....G.....AGCGUAGGGAGCUCCACAG.CCCAGC..C.UGGCUGG...GA..C.........CCCCCUA.....C.AUAAUCUG.................................
Ga0115595_1153837/320-197      ................UGC.CU.UU.............................................................................................................................
Ga0242657_1026087/460-322      A.CACCUU.AUACCC.CAA.C.................................................................................................................................
Ga0265751_100417/1480-1588     ................AGU.CG................................................................................................................................
Ga0265756_100485/1108-1000     ................AGU.CG................................................................................................................................
Ga0265751_100393/1594-1698     ................CAA.GC................................................................................................................................
Ga0307921_1011071/674-791      U.................G.AU.CU.............................................................................................................................
Ga0307919_1094420/160-285      C.G.............AUC.C.................................................................................................................................
Ga0316034_107719/314-205       .......................GG.UU.A.....G.....CGUAAUCC.C...................................................................................................
Ga0265742_1065119/361-273      ................UUG.AU.GG.AA..........................................................................................................................
Ga0180107_1164033/560-431      U.C.............UUA.C.................................................................................................................................
Ga0242641_1001148/1456-1256    ................CUG.UU...................CGAGUUAC.ACCUCAGGUG.UGCUUG..CAUGGCUUG...CA..C.........AUGAUC.................................................
Ga0307482_1009758/562-669      ................AAG.C.................................................................................................................................
Ga0247527_109126/592-695       ................UUU.UA................................................................................................................................
Ga0247519_111615/148-45        ................CAA.UC.AA.............................................................................................................................
Ga0247513_116034/391-494       ................CAA.UC.AA.............................................................................................................................
Ga0213850_1290372/434-275      ................CAU.AC.GU.UA......................................A..A.ACGAGCC...GA..U.........UGGUCCU.....G.ACAAGGA..................................
Ga0138276_1121602/329-410      ................CAA.CC................................................................................................................................
Ga0138278_1041650/183-102      ................UUC.GA................................................................................................................................
Ga0138277_1137481/570-489      ................UUC.AA.GA.............................................................................................................................
Ga0138278_1041788/293-378      ................UUC.AA.CA.............................................................................................................................
Ga0138277_1142657/273-361      ................UUC.AA.CA.............................................................................................................................
Ga0138275_1223952/371-283      ................UUC.AA.CA.............................................................................................................................
Ga0213850_1450246/199-116      ................GUA.CA................................................................................................................................
Ga0213850_1307525/101-186      ................UAC.AA................................................................................................................................
Ga0265756_118952/425-318       ................UUC.AU.UA.UU.G.....A.....ACCAUUAU.U...................................................................................................
Ga0180107_1244437/408-507      ................CAG.UG................................................................................................................................
Ga0265742_1051722/222-295      ................UAA.G.................................................................................................................................
Ga0242657_1051800/416-321      ................UAG.CA................................................................................................................................
Ga0307484_102966/353-250       ................UAG.U.................................................................................................................................
Ga0184568_108419/50-147        ...........ACCA.UAA.GC.A..............................................................................................................................
Ga0184568_103677/484-581       ................AAG.C.................................................................................................................................
Ga0307484_102709/562-403       ................AUG.GC.AA.CC.G.....G.....GUGAUUCC.CUGU................................................................................................
Ga0127503_10678853/419-333     ................UAC.AA................................................................................................................................
Ga0307919_1065410/76-162       ................AUU.AA................................................................................................................................
Ga0242652_1038498/365-470      ................GAG.A.................................................................................................................................
Ga0307484_101264/723-941       ................GAG.C......A.A.....G.....AGCGUCAA.GUCCCGUGUC.AAUUCA..C.GGGCCAG...UC..C.........GUAUUCC.....A.GACUGGGUAG..G............................
Ga0247534_101560/228-122       ................AGC.CA................................................................................................................................
Ga0307484_109931/110-215       ................GCA.CG................................................................................................................................
Ga0184595_116681/414-266       ................UUG.CG................................................................................................................................
Ga0265749_100155/962-1187      ................UGG.AU.AA.AC.G.....G.....CUUGUCCG.UU.....GGU.CUCGAG..G.GCGCACG...CU..A.........UCGUCCG.....A.UUACUCG..................................
Ga0310115_109341/244-400       ..................................................................................................................................................CCUG
Ga0310111_106996/679-905       ................UGG.AU.AA.AC.G.....G.....CU.......................................................................................................UGUC
Ga0265756_101315/275-501       A.UCAGAA.CUGCCG.UCA.GA.UU.GA...............CGUACG.CUCGACUCUU.GGAUAC..A.CGGCUUG...UC..C.........GUA....................................................
Ga0265751_100874/820-594       A.UCAGAA.CUGCCG.UCAGAU.UG.AC.G.....C.....ACGCUCGA.CUCUUGGAUG.A..........................................................................AC..G..GCUUGUC
Ga0265744_120119/40-266        A.UCAGAA.CUGCCGUCAG.AU.UG.AC.G.....C.....ACGCUCGA.CUCUUGGAUG...........................................................................AAC..G..GCUUGUC
Ga0310102_105010/226-452       A.UCAGAA.CUGCCG.UCA.GA.UUGAC.G.....C.....ACGCUCGA.CUCUUGG......AUAA.....................................................................AC..G..GCUUGUC
Ga0247534_103639/475-250       A.UCAGAA.CUGCCG.CCA.GAUUG.AC.G.....C.....ACGCUCGA.CUCUUG...................................................................GAU.........AAC..G..GCUUGUC
Ga0265741_101833/434-208       A.UCAGAA.CUGCCG.UCA.GAUUG.AC.G.....C.....ACGCUCGA.CUCUUGGAUG...........................................................................AAC..G..GCUUGUC
Ga0242647_1002114/801-1026     A.UCAGAA.CUGCCG.UCA.GA.UUGAC.G.....C.....ACGCUCGA.CUCUUGG......AUAA.....................................................................AC..G..GCUUGUC
Ga0265752_104585/383-158       A.UCAGAA.CUGCCG.UCA.GAUUG.AC.G.....C.....ACGCUCGA.CUCUUGGAUG.A..........................................................................AC..G..GCUUGUC
Ga0310107_109330/1076-850      A.UCAGAA.CUGCCG.UCA.GA.UU.GACG.....C.....ACGCUCGA.C..............UU..C.UGGA.................................................UG.........AAC..G..GCUUGUC
Ga0115595_1051440/308-419      ................CCA.C.................................................................................................................................
Ga0115594_1054328/389-276      ................CCA.C.................................................................................................................................
Ga0307482_1048753/383-528      ................UAG.GC.AG.UU.G.....C.....CCGUUUCU.GUGGGGUUUU.ACGCCACAA.CGGCUUC...GA..U.........GCGU...................................................
Ga0307480_1006257/684-599      ................GUU.UU................................................................................................................................
Ga0184595_116802/212-325       ................UCU.GU................................................................................................................................
Ga0307919_1071894/312-31       ..........CUCGA.CUU.UU.UC.AG.A.....CGAGAGACUGGGGU.CCACCAACCG.GGUAGG..G.AAGUUUU...GU..U.........UCCUAGC.....A.CCCUGGGGUA..CAAAC.........UAU..G..UUACGAC
Ga0307921_1036773/354-73       ..........CUCGA.CUU.UU.UC.AG.ACGAGAG.....ACUGGGGU.CCACCAACCG.GGUAGG..G.AAGUUUU...GU..U.........UCCUAGC.....A.CCCUGGGGUA..CAAAC.........UAU..G..UUACGAC
Ga0307482_1023665/889-1191     A.CAAUCG.CGGCCU.AUA.GC.CG.GA.A.....G.....CGAGUCCG.UAC..................CGGUACC...CACAU.........GCUACAG.....C.AACACUGUGG..UAGAG.........GGU............
Ga0180108_1022680/3902-3998    ................UAG.CA................................................................................................................................
Ga0184599_137402/105-300       ................UGC.CG.CA.CC.G.....A.....AUACACUA.ACAGUGACGA.GUUGCA..G.CCGCAAC........................................................................
Ga0157569_1080558/455-605      ................AUU.GA................................................................................................................................
Ga0184601_113231/247-149       ................UGC.GA.GA.............................................................................................................................
Ga0242652_1002960/413-574      ................AGU.GG.CA.AU.G.....G.....AG...........................................................................................................
Ga0307484_101303/704-866       ................CUC.UG................................................................................................................................
Ga0242641_1025149/251-89       ................CUC.CG................................................................................................................................
Ga0307482_1023050/594-496      ................UAG.CA................................................................................................................................
Ga0307484_100434/1330-1210     ................AAG.G........................................................................................................U........CCA..C..GCGCCUG
Ga0153880_1432604/289-395      ................AGG.UA................................................................................................................................
Ga0157559_1128929/201-87       ................CUU.A.................................................................................................................................
Ga0157560_1113202/155-269      ................CUU.A.................................................................................................................................
Ga0157566_1012736/497-384      ................AAA.UA................................................................................................................................
Ga0157579_1155702/323-436      ................AAG.UA................................................................................................................................
Ga0157562_1157806/189-74       ................AAA.UA................................................................................................................................
Ga0180047_1032966/152-37       ................AAA.UA................................................................................................................................
Ga0307484_166952/62-163        ................CAG.CG................................................................................................................................
Ga0005851_1178990/477-379      ................CCA.U.................................................................................................................................
Ga0153880_1191383/455-340      ................AC....................................................................................................................................
Ga0213850_1282564/267-350      ................CGA.AA................................................................................................................................
Ga0315817_133437/498-413       ................CGA.A.................................................................................................................................
Ga0315826_123252/262-347       ................CGA.AA................................................................................................................................
Ga0242657_1008904/1267-1104    ................CAC.GA.AU.GC.G.....C.....CGAAACCU.....................................................................................................
Ga0222728_1009902/1182-968     ................AGU.GU.GU.AG.G.....C.....CUUUUCUC.CCCACACCCC.CGAUCG...................................................................................
Ga0115594_1106220/441-334      ................CGC.CA.A..............................................................................................................................
Ga0180107_1307934/226-315      ................ACC.AG................................................................................................................................
Ga0184577_115871/202-369       U.UGAUGC..GACAA.GUA.CC..U.UG.G.....A.....CU...........................................................................................................
Ga0206356_11590518/224-68      ................AAG.AC.GU.AC..........................................................................................................................
Ga0222728_1062510/166-66       ................AUU.CA................................................................................................................................

                               <.<<<<<<.<<<<<<.......................................................................................................................................
                               ......................................................................................................................................................
                               ----------------●●●-●○--------------------------------------------------------------------------------------------------------------------------------
```

```
Ga0316029_101202/440-551             ................AAG.C.................................................................................................................................
Ga0307482_1017422/1387-1206          ................GGC.CG.GU.AU.C.....C..................................................................................................................
Ga0307484_100889/412-594             C.A.............GGU.AU.CC.............................................................................................................................
Ga0255062_11230321/200-110           ................UGA.GC................................................................................................................................
Ga0255059_10691632/166-76            ................UGA.GC................................................................................................................................
Ga0265744_100073/1235-1401           ................GAC.CA................................................................................................................................
Ga0265757_113703/332-416             ................CUA.UC................................................................................................................................
Ga0265756_158829/72-188              ................GAA.AA.AG.............................................................................................................................
Ga0184597_100195/107-221             ................UUG.GA.UC.UU.G.....C.....AUCCUAGA.GUUG................................................................................................
Ga0184594_121503/721-607             ................UUG.GA.UC.UU.G.....C.....AUCCUAGA.GUUG................................................................................................
Ga0184582_121032/1005-1119           ................UUG.GA.UC.UU.G.....C.....AUCCUAGA.GUUG................................................................................................
Ga0184602_104873/671-785             ................UUG.GA.UC.UU.G.....C.....AUCCUAGA.GUUG................................................................................................
Ga0184589_132372/822-708             ................UUG.GA.UC.UC.G.....C.....AUCCUAGA.GUUG................................................................................................
Ga0184580_125558/64-178              ................UUG.GA.UC.UC.G.....C.....AUCCUAGA.GUUG................................................................................................
Ga0184596_101726/395-281             ................UUG.GA.UC.UU.G.....C.....AUCCUAGA.GUUG................................................................................................
Ga0247541_109256/178-292             ................UUG.GA.UC.UU.G.....C.....AUCCUAGA.GUUG................................................................................................
Ga0184584_108444/253-366             ................UUG.GA.UC.UU.G.....C.....AUCCUAGG.GUUG................................................................................................
Ga0184582_124913/742-629             ................UUG.GA.UC.UU.G.....C.....AUCCUAGG.GUUG................................................................................................
Ga0184600_135218/318-205             ................UUG.GA.UC.UU.G.....C.....AUCCUAGG.GUUG................................................................................................
Ga0247541_100074/611-498             ................CUG.GA.UC.UU.G.....C.....AUCC.........................................................................................................
Ga0247551_101122/731-844             .................UG.GA.UC.UU.G.....C.....AUCC.........................................................................................................
Ga0247526_101949/462-575             ................CAU.CC................................................................................................................................
Ga0247524_101842/357-244             ................CAU.CC................................................................................................................................
Ga0247519_108765/580-693             ................CUU.GC................................................................................................................................
Ga0180107_1134582/382-542            ................CCC.C................................................C.UGGCUUG........................................................................
Ga0265747_125301/405-306             ................UAG.UA..A.............................................................................................................................
Ga0157593_1101573/244-147            ................UCA.AA................................................................................................................................
Ga0184599_121568/40-155              ................UCA.AA................................................................................................................................
Ga0316040_116783/379-494             ................UCA.AA................................................................................................................................
Ga0157560_1091679/440-538            ................UCA.AA................................................................................................................................
Ga0222728_1165579/392-259            ................CUC.A.................................................................................................................................
Ga0224712_10601975/393-502           ................GAG.UA................................................................................................................................
Ga0224712_10010317/1973-2082         ...................................................................................................................UCUC..AA...........................
Ga0307482_1048660/422-625            ............................................ACUCU.CACAGGGCCG.UGAGCC..G.UAGCUAG...AA..C.........CGAGUGA.....U.UUCGGACUGG..UCCGC........GCA...G..GUCUGUC
Ga0179934_1155244/114-26             ................AGA.A.................................................................................................................................
Ga0307484_101172/602-495             ................GCA.GU.CG.UA.A.....A.....AG...........................................................................................................
Ga0242654_10068518/113-218           ................GUC.GG.UA.AA..........................................................................................................................
Ga0242647_1002406/915-1021           ................GUC.AG.UA.AA..........................................................................................................................
Ga0222729_1010500/11-117             ................GUC.GA.UA.AA..........................................................................................................................
Ga0307482_1202605/45-154             ................UGG.A.....GA.G.....U.....AGCCU...............AAGC.....................................................................................
Ga0184603_139562/141-280             ................AAA.A.................................................................................................................................
Ga0242646_1001122/826-995            G.UAGUG.........ACA.CA................................................................................................................................
Ga0310107_144222/327-436             ................AUC.UC.CG.AU.G.....C.....AUAUGCCA.A...................................................................................................
Ga0265751_124807/392-283             ................AUC.UU.CG.AU.G.....C.....AUAUGCCA.A...................................................................................................
Ga0184572_105168/388-281             ................UAU..........G.....A.....CGCAAUCC.CAAGC...............................................................................................
Ga0316036_127178/124-231             ................UAU..........G.....A.....CGCAAUCC.CAAGC...............................................................................................
Ga0222728_1003482/1262-1107          ................UCC.UG................................................................................................................................
Ga0265743_100639/33-191              ................AGG.CC................................................................................................................................
Ga0307484_105374/518-723             A.CA............UGU.G.................................................................................................................................
Ga0265742_1015608/410-491            ................ACA.AA................................................................................................................................
Ga0316030_102139/629-482             ................AGG.CC.UU.GC.G.....C.....UUAGUCAA.AU..................................................................................................
Ga0265749_106268/131-212             ..................G.GA.GG.GU.G.....G.....AC...........................................................................................................
Ga0184573_100752/140-46              ................UUG.A.................................................................................................................................
Ga0157560_1010344/357-456            ................CAA.A.................................................................................................................................
Ga0157593_1006201/239-338            ................CAA.A.................................................................................................................................
Ga0180047_1087048/281-180            ................CAC.A.................................................................................................................................
Ga0157565_1016893/233-334            ................CAC.A.................................................................................................................................
Ga0184568_100511/100-195             ................UGU.G.................................................................................................................................
Ga0265759_114856/370-262             ................CGA.AA.GA.AA..........................................................................................................................
Ga0222729_1080556/362-213            ................UCA.U.................................................................................................................................
Ga0247541_102707/328-184             U.C.............UCA.C.................................................................................................................................
Ga0307921_1020694/406-483            ................UUU.AU................................................................................................................................
Ga0307919_1034281/527-451            ................UAA.U.................................................................................................................................
Ga0184568_108869/934-1072            ................CUU.GA................................................................................................................................
Ga0206356_10036221/150-306           ................AUG.C.................................................................................................................................
Ga0247554_104968/233-375             ................UUA.UA................................................................................................C..UAGAC........UGC...G..G......
Ga0265753_1030448/124-358            U.G...........A.CUC.A........A.....C.....UGUCACUA.ACCGGGGCUG.CGUCAG..U.UCGCAG......................................AGAG..AAAUC........AAA...A..GUUCUCG
Ga0032354_1086862/128-19             ................CGU.GA................................................................................................................................
Ga0213850_1119088/23-236             G.CU............CAC.CU.GC.UG.G.....C.....AUCACCUU.CUA.........GCUUG..A.UAGCACU...GG..U.........AUCUCUA.....A.ACCAGGAGGG..UGG..........................
Ga0222728_1000079/1837-1731          ................UUG.CA.UC.AA.A.....G..................................................................................................................
Ga0247535_100068/719-828             ................UAG.CG.UA.AU..........................................................................................................................
Ga0265744_100861/398-567             G.CA............AUA.C...........................................CCU..G.UG.............................................................................
Ga0316038_100042/1676-1569           ..............A.UUC.G......U.G.....G.....AAUCCCAA.G...................................................................................................
Ga0184578_101068/480-310             A.CA............CGG.CC.UC.GU.G.....C.....AA...........................................................................................................
Ga0265759_100473/818-988             A.CA............CGG.CC.UC.GU.G.....C.....AA...........................................................................................................
Ga0265756_100592/1458-1288           A.CA............CAG.CC.UC.GU.G.....C.....AA...........................................................................................................
Ga0265746_1003835/1049-1219          A.CA............CGG.CC.UC.GU.G.....C.....AA...........................................................................................................
Ga0247536_100773/637-807             A.CA............CGG.CC.UC.GU.G.....C.....AA...........................................................................................................
Ga0184573_112336/323-153             A.CA............CGG.CC.UC.GU.G.....C.....AA...........................................................................................................
Ga0247530_109035/489-319             A.CA............CGG.CC.UC.GU.G.....C.....AA...........................................................................................................
Ga0247513_128543/241-71              A.CA............CGG.CC.UC.GU.G.....C.....AA...........................................................................................................
Ga0247541_101130/558-391             C.C.......U.ACG.GCC.U.................................................................................................................................
Ga0213850_1278242/600-506            ................GCG.AC.G..............................................................................................................................
Ga0213850_1524759/286-380            ................GCG.GA.G..............................................................................................................................
Ga0180107_1045142/740-541            C.CCGUAG..CCCUU.UAU.A.................................................................................................................................
Ga0307484_102002/698-552             ................UAG.GC.GG.AA.G.....C.....CCGUAGAA.CUAGUGGGCG.AACCA....................................................................................
Ga0265759_115288/4-102               .....................A.GC.AA.G.....A.....GCAGUGCU.....................................................................................................
Ga0316029_136733/88-8                ................UCA.CU................................................................................................................................
Ga0242646_1008525/229-119            ................UAC.UA................................................................................................................................

                                     <.<<<<<<.<<<<<<.......................................................................................................................................
                                     ......................................................................................................................................................
                                     ----------------●●●-●○--------------------------------------------------------------------------------------------------------------------------------
```

```
Ga0115594_1180137/311-161     ................GCC.A.................................................................................................................................
Ga0179947_1156513/270-495     U.AU............ACA.AA.CC.UG.A.....G.....CGCUUAAA.UUAGCGUACG.AAGUUU..U.ACACUCG...UU..C.........UCGUUUA.....A.AUCAGGAAUC..AAGCG........GAU...U..GUUUAUA
Ga0179948_1263234/366-292     ................ACA.UU................................................................................................................................
Ga0179947_1148619/378-303     ................ACA.UU.G..............................................................................................................................
Ga0257141_1138773/402-200     A.CAGUGA.CAACCA.GAA.AA................................................................................................................................
Ga0242654_10401151/84-171     ................GCA.A.................................................................................................................................
Ga0184603_114388/264-401      ................GAA.A.................................................................................................................................
Ga0316052_118887/313-177      ................GAA.A.................................................................................................................................
Ga0079039_1671802/197-34      ................CCA.GA................................................................................................................................
Ga0247542_100007/609-500      ................AUG.GA.GA.UA.G.....C..................................................................................................................
Ga0247552_100007/4311-4420    ..................G.GA.GA.UA.G.....C..................................................................................................................
Ga0184586_103737/25-134       ................AUG.GA.GA.UA.G.....C..................................................................................................................
Ga0184594_103311/207-316      ................AUG.GA.GA.UA.G.....C..................................................................................................................
Ga0265756_101275/650-541      ................AUG.GA.GA.UA.G.....C..................................................................................................................
Ga0247541_100010/3268-3377    ................AUG.GA.GA.UA.G.....C..................................................................................................................
Ga0184599_154003/184-293      ................AUG.GA.GA.UA.G.....C.....CC...........................................................................................................
Ga0184580_121439/573-464      ................AUG.GA.GA.UA.G.....C..................................................................................................................
Ga0184582_108821/1050-1159    ................AUG.GA.GA.UA.G.....C..................................................................................................................
Ga0184575_101094/586-477      ................AUG.GA.GA.UA.G.....C..................................................................................................................
Ga0184598_129111/512-403      ................AUG.GA.GA.UA.G.....C..................................................................................................................
Ga0184596_107732/313-205      ................AUG.GA.GA.UA.G.....C..................................................................................................................
Ga0247554_100006/1592-1485    ................AUG.GA.GA.UA.G.....C..................................................................................................................
Ga0247534_100047/1899-2008    ................AUA.AC................................................................................................................................
Ga0184597_128933/573-464      ................AUG.GA.GA.UA.G.....C..................................................................................................................
Ga0213850_1302171/343-113     ................CCG.CA.CG.GU.G.....G.....GUGCAUAC.GCGUAGUCGG.CUUCGG..U.UGAUAGC...GC..A.........GGGAUGA.....U.GACAGUUGUG..UCUAG........GGC...A..GCUAUCG
Ga0213850_1513954/279-196     ................AUA.CA................................................................................................................................
Ga0153880_1231581/116-241     ................UGG.UC................................................................................................................................
Ga0247541_104364/333-188      ................GUU.AA................................................................................................................................
Ga0138277_1028338/433-516     ................GAA.A.................................................................................................................................
Ga0265752_101001/521-687      ................CAG.AA.....................................................GUG...GG..A.........CCGUAAA.....C.CCAAGCCCAC..GUGGC........UCU...G..GUGUCUC
Ga0307482_1449537/408-223     A.C.............CUG.CU...................................UAG.GUCCCA..G.UGGCUCU...GC..U.........AC.....................................................
Ga0257136_1044348/475-684     ................AUG.GA.UA.AA.G.....C.....AUACUCUC.AUAUCGGCUU.AAGCCG..G.UGGCAUG...GG..U.........ACGUUUA.....A.ACAUGUGUAG..AGUGC........AGA...G..GUUCCUU
Ga0257138_1055324/266-476     ................AUG.GA.UA.AA.G.....C.....AUACUCUC.AUAUCGGCUU.AAGCCG..G.UGGCAUG...GG..U.........ACGUUUA.....A.ACAUGUGUAG..AGUGC........AGA...G..GUUCCUU
Ga0257140_1130477/77-287      ................AUG.GA.UA.AA.G.....C.....AUACUCUC.AUAUCGGCUU.AAGCCG..G.UGGCAUG...GG..U.........ACGUUUA.....A.ACAUGUGUAG..AGUGC........AAA...G..GUUCCUU
Ga0127502_10711632/191-294    ................GAA.GU................................................................................................................................
Ga0184568_105547/129-239      ................GUG.A......................C..........................................................................................................
Ga0206353_11976089/292-380    ................UGA.U..U..............................................................................................................................
Ga0184577_108791/182-295      ................GGC.AA.G..............................................................................................................................
Ga0242649_1050441/340-170     ................AGA.A........G.....C.....GUUAGCUC.CCGGUAUCUG.AGCCAA..C.UCACCUG...AA..A.........UCCGGUG.....G.AAAUCCGGAG..AAUGG........UGU...G..AGGCCAG
Ga0179934_1197833/207-115     ................AUC.A.................................................................................................................................
Ga0180107_1174136/218-102     ................AAG.UC................................................................................................................................
Ga0224712_10002011/1106-1211  ................AAG.C.................................................................................................................................
Ga0224712_10010317/2429-2325  ................AAG.C.................................................................................................................................
Ga0206355_1311803/144-334     ................ACU.AC.AA.UU.G.....G.....AACGUUAG.U...................................................................................................
Ga0307482_1417159/374-296     ................UUC.AU................................................................................................................................
Ga0213850_1396191/936-678     C.C.............GCC.AA.AC.GC.A.....C.....AAUGGGCC.ACCAGAGUGG.GAACGG..U.GGGCGCU...GU.GA.........GAGAUCA.....C.UCAUCAAAAG..ACGUGAAUACCUCAAU...U..GUACAUA
Ga0222728_1287830/12-211      ................CCA.AC.UC.............................UGGAUG.AUUAUU..C.AGACUAU...AA..G.........UCGUUUA.....A.UCU......................................
Ga0242652_1169169/12-211      ................CCA.AC.UC.............................UGGAUG.AUUAUU..C.AGACUAU...AA..G.........UCGUUUA.....A.UCU......................................
Ga0242654_11312699/30-229     ...UGAAA.CAACU....................................ACUUGGU.............................................................................................
Ga0265759_104327/339-243      ................UUG.G.................................................................................................................................
Ga0265756_100717/910-1050     .................AC................................................................................UCU.....U.AAAACAAGAG..CAGAG........GGU...G..AUACCUC
Ga0265757_113513/277-362      ................ACC.UA................................................................................................................................
Ga0242654_10024002/836-915    ................UUA.AG................................................................................................................................
Ga0184573_101763/767-477      U.CAUUGGGCUGCGG.UGA.UU.CG.CA.G.....A.....UGUGUACA.GCUAAAACCA.UCGGUU..G.UCGUUUG...GG..U.........C.GUUCC.....C.GACCUAGCGA..UAUCG........UCA...G..GUCUCCA
Ga0242654_10701525/53-296     ................AUC.CA.CU.UG.A.....C.....UGUGUGUU.GUCCUAUUGU.CACUUU..G.UGACUCC...GG..A.........CCUCUCC.....AGCUCACUG..................................
Ga0247529_117158/253-331      ................GCA.AU.CA.AA..........................................................................................................................
Ga0326631_117973/181-293      ................GUG.A.................................................................................................................................
Ga0316038_107116/33-174       ................UUU.A.................................................................................................................................
Ga0316037_108094/19-160       ................UUU.A.................................................................................................................................
Ga0247527_116942/161-88       ................UGC.A.................................................................................................................................
Ga0138278_1022898/219-127     ................AUU.CA.AA.UA..........................................................................................................................
Ga0127503_11028958/51-130     ................AUC.UA................................................................................................................................
Ga0307482_1623775/241-347     ................UUA.AA.U..............................................................................................................................
Ga0307484_111921/444-550      ................UUA.GA.U..............................................................................................................................
Ga0307484_101582/1018-1124    ................UUA.GA.U..............................................................................................................................
Ga0265742_1021317/385-459     ................AUC.CA................................................................................................................................
Ga0157569_1089063/252-138     ................CUU.A.................................................................................................................................
Ga0153880_1457361/236-121     ................AUA.A.................................................................................................................................
Ga0256352_1087084/23-118      ................AUU.AA................................................................................................................................
Ga0307482_1000325/24-131      .................AG...................................................................................................................................
Ga0157560_1147189/148-60      ................CAA.G.................................................................................................................................
Ga0247519_105128/410-263      ................UGG.AC.UU.UC.G.....G.....CUUGAUCG.UCCGAGAGGG.GGCCAA..G.UGGCUUC...GC..U.........UUGAUCU.....U.AAAAUUG..................................
Ga0184573_113193/903-756      ................UGG.AC.UU.UC.G.....G.....GUUGAUCG.ACCGAGAGGG.GGCCAA..G.UGGCUUC...GC..U.........UUGAUCU.....U.AAAAUUG..................................
Ga0157593_1013623/299-215     ................UUC.GA.AA.UA.A.....G.....A............................................................................................................
Ga0184593_126886/349-213      ................UGC.CA................................................................................................................................
Ga0184568_117306/561-697      ................UGC.CA................................................................................................................................
Ga0184597_120210/233-147      ................AAA.CG................................................................................................................................
Ga0184570_100371/69-155       ................AGA.CG................................................................................................................................
Ga0157562_1160399/232-315     ................AUG.A.................................................................................................................................
Ga0265744_114151/132-24       ................AUA.GU................................................................................................................................
Ga0265743_119695/104-184      ................CGG.UA................................................................................................................................
Ga0265743_101198/448-619      C.AA............UAC.UC.....................CGUG.......................................................................................................
Ga0316046_121611/330-214      ................CUA.AU................................................................................................................................
Ga0265757_120140/89-204       ................CUC.UA.AU.U...........................................................................................................................
Ga0138279_1083071/419-326     ..............A.CUA.AA................................................................................................................................
Ga0310106_100998/1608-1690    ................UGA.UC................................................................................................................................
Ga0265756_102054/286-395      ................UAC.AA................................................................................................................................
Ga0180108_1209503/2438-2343   ................UAG.UA................................................................................................................................
Ga0180107_1057591/210-107     ................AUA.AC.AG.AA.G.....G..................................................................................................................

                              <.<<<<<<.<<<<<<.......................................................................................................................................
                              ......................................................................................................................................................
                              ----------------●●●-●○--------------------------------------------------------------------------------------------------------------------------------
```

```
Ga0307482_1074296/478-354      ................GUC...................................................................................................................................
Ga0180108_1022680/4248-4153    ................CAG.CG................................................................................................................................
Ga0247519_103291/1381-1460     ................UUA.U.................................................................................................................................
Ga0184590_126350/300-380       ................UUU.AU................................................................................................................................
Ga0247551_100996/477-556       ................UUA.U.................................................................................................................................
Ga0316035_144545/195-301       ................UUG.UC................................................................................................................................
Ga0316049_154968/227-319       ................UUG.U.................................................................................................................................
Ga0184593_115334/333-240       ................AUG.UG.U..............................................................................................................................
Ga0307921_1005950/551-460      ................UGC.GU.............................................................................CUA.....U..........................................
Ga0242647_1014694/423-336      ................UUG.C.................................................................................................................................
Ga0184580_102899/442-582       ................AUG.UC.GC.AA.G.....A.....CAUAUCUC....................................................G.....A.AAG......................................
Ga0265743_108998/150-253       ................UGC.G.................................................................................................................................
Ga0316050_103193/566-460       ................UGC.GU.AA.AA.G.....C.....AAUGUCAA.GUGACUA.............................................................................................
Ga0316030_100208/805-700       ................CGU.GC.GA.AA.G.....C.....AAUGUCAA.GC..................................................................................................
Ga0307482_1000262/4800-4699    ................GUC.G.................................................................................................................................
Ga0307482_1018929/1182-1081    ................GUC.G.................................................................................................................................
Ga0153880_1260556/441-589      ................AUC.GU................................................................................................................................
Ga0180044_1087599/197-316      ................GAG.UC................................................................................................................................
Ga0127502_11371108/362-252     ................UUG.UC.CA.GA.G.....G.....AAUCCCAA.GU..................................................................................................
Ga0184588_106948/865-640       ................GGA.UC.GA.AA.U.....C.....CGCGAUCU.UCCCAAGCGC.UAGCUA..A.GCGCAUC...AA..C.........CGGUUUA.....C.CCAGUUGA.................................
Ga0247533_100438/1040-888      ................UGC.AC.UA.GG.C.....A.....AGUGACAG.GUAGCAACGC.UGACAC..A.GGGUGUU...AG..A.........ACCUAGU.....U.ACAAAAG..................................
Ga0127502_10189886/163-55      ................AAG.AG................................................................................................................................
Ga0079074_1318576/412-571      .........CAACCA.GUC.AU.A..............................................................................................................................
Ga0115594_1212468/8-118        ................CCC.CA................................................................................................................................
Ga0115594_1134035/652-542      ................CCC.CA................................................................................................................................
Ga0316030_105840/623-504       ................CGU.G.................................................................................................................................
Ga0316033_110175/528-409       ......................................................................................................................................................
Ga0228708_1128185/228-344      ................UUG.AU.CA.AA..........................................................................................................................
Ga0126318_10199518/109-204     ................UAC.G.................................................................................................................................
Ga0265745_1000697/596-785      U.C.............AGC.AC.........................GC.GCCACGCGUG.AUGUGU..C.CAGGUUG...GG..U.........GA.....................................................
Ga0265747_105373/294-105       U.C.............AGC.AC.........................GC.GCCACGCGUG.AUGUGU..C.CAGGUUG...GG..U.........GA.....................................................
Ga0265745_1001084/890-701      U.C.............AGC.AC.........................GC.GCCACGCGUG.AUGUGU..U.CAGGUUG...GG..U.........G......................................................
Ga0265745_1000512/1043-854     U.C.............AGC.AC.........................GC.GCCACGCGUG.AUGUGU..C.CAGGUUG...GG..U.........G......................................................
Ga0310117_104105/1058-869      U.C.............AGC.A.........................CGC.ACCACGCGUG.AUGUGU..C.CAGGUUG...GG..U.........GAU....................................................
Ga0184587_113157/56-168        ................AAA.GA.CC.UC..........................................................................................................................
Ga0206356_10600373/215-21      ................AAG.CC.GG.AU.G.....G.....AUCAUAAA.GUCCUGGC............................................................................................
Ga0307920_126542/349-261       ................GUG.AA.UA.AA..........................................................................................................................
Ga0265742_1001154/131-376      .......................AC.GG.G.....G.....CCUUUGUC.CCGUAGUCCG.AACACU..G.UCGUCCC...AA..A.........CCCUGGU.....A.AC............CGC........ACU...G..CGGCAUA
Ga0307482_1127544/505-398      ................UUU.UC................................................................................................................................
Ga0127503_11058765/362-444     ................GCG.A.................................................................................................................................
Ga0210323_1050507/418-532      ................AUG.A.................................................................................................................................
Ga0242647_1002063/315-62       ................GCC.CA.CU.UG.G.....A.....UUUGGCUA.GCCGGAAACC.GGUGCG..C.CGGUAGU...AC..G.........UCGCCUU.....A.ACCUUGAAGGCCUACCG........AAC...G..GUUAUCG
Ga0307484_100992/499-246       ................GCC.CA.CU.UG.G.....A.....UUUGGCUA.GCCGGAAACC.GGUGCG..C.CGGUAGU...AC..G.........UCGCCUU.....A.ACCUUGAAGGCCUACCG........AAC...G..GUUAUCG
Ga0247534_117055/74-212        C.C...........A.CAC.A..A..............................................................................................................................
Ga0129331_1494381/292-368      ................AGU.AU................................................................................................................................
Ga0242646_1000880/695-856      ................GUG.AC................................................................................................................................
Ga0180107_1174136/376-571      ................UUU.AA.AA.UG.G.....G.....AG...........................................................................................................
Ga0307921_1050271/251-412      .................................................................AC..A.CGGAACC...AA..G.........ACUUUCG.....U.CUAAUCACG................................
Ga0247524_126530/125-198       ................GAU.GA................................................................................................................................
Ga0242646_1001007/790-609      .................AU.GA...........................................UG..U.UUACCCG...GG..U.........GAUUCCG.....G.ACCUCU...................................
Ga0213850_1280121/544-351      G.CA............UCG.CA.UG.AA.G.....U.....AUUAGCAC.ACGUUGUGCUUAGACUG..C.AUGCGGG...GU..G.........GGCUUGU.....G.ACCACCACUG............................CUG
Ga0307919_1109364/247-158      .................UU.AA.UC.AA..........................................................................................................................
Ga0184600_147964/253-339       ................AUG.UA................................................................................................................................
Ga0316036_107710/408-322       ................AUG.UA................................................................................................................................
Ga0184598_138497/440-355       ................CUU.G.................................................................................................................................
Ga0184589_121067/502-587       ................CUU.G.................................................................................................................................
Ga0184601_135946/406-321       ................CUU.G.................................................................................................................................
Ga0115595_1093158/7-137        ................UAU.C.................................................................................................................................
Ga0005851_1006483/274-172      ................UUC.U.................................................................................................................................
Ga0247522_108409/684-554       ................AAG.AC................................................................................................................................
Ga0310119_105137/743-673       ................AUU.CU................................................................................................................................
Ga0247529_116306/255-362       ................ACG.G.................................................................................................................................
Ga0316046_116376/278-384       ................ACG.G.................................................................................................................................
Ga0247534_102735/124-18        ................ACG.G.................................................................................................................................
Ga0247540_102569/210-104       ................ACG.G.................................................................................................................................
Ga0316037_112281/325-219       ................UCG.GA................................................................................................................................
Ga0184576_110594/6-113         ................ACG.G.................................................................................................................................
Ga0316049_114344/181-288       ................ACG.G.................................................................................................................................
Ga0115594_1200134/154-31       ................CCU.UU................................................................................................................................
Ga0307480_1000549/913-1064     ................AAG.CC................................................................................................................................
Ga0242647_1000589/378-557      ................AUG.AA................................................................................................................................
Ga0222729_1000922/1729-1550    ................AUG.AA................................................................................................................................
Ga0182091_1104347/98-194       ................AAU.CA.AA.UA..........................................................................................................................
Ga0316037_128286/253-347       ................ACU.CA.AA.UA..........................................................................................................................
Ga0180107_1040049/1100-1010    ................AUU.A.................................................................................................................................
Ga0242641_1014724/551-384      ................AAC.UG................................................................................................................................
Ga0242641_1002366/499-331      ................GAA.CU.G..............................................................................................................................
Ga0213850_1508869/496-402      ................UCA.AA................................................................................................................................
JGI12272J11983_1072695/40-150  ................GUG.CG.UA.UA.G.....C.....GCAAUCCC.AA..................................................................................................
Ga0307482_1020622/600-404      ACCAGUAC..CACCG.AUG.CU.CA.CG.A.....C.....AUGUGAAC.C...................................................................................................
Ga0242657_1009512/1576-1466    ................UGC.GC................................................................................................................................
Ga0184577_116723/415-311       ................UAU.CA................................................................................................................................
Ga0184576_109717/432-536       ................AUU.CC................................................................................................................................
Ga0184572_113323/230-300       ................UUU.U.................................................................................................................................
Ga0307354_155926/276-116       ................UUG.CA...................................CAG.CUGUUU..G.AGGCCCG...CU..G.........CC.....................................................
Ga0265756_100433/485-286       ................AGG.CC................................................................................................................................
Ga0265751_100477/874-675       ................AGG.CC................................................................................................................................
Ga0180045_119797/166-305       ................GAA.CC.GU.GA.A.....C.....AA...........................................................................................................
Ga0222728_1380294/134-238      ................AAC.U.................................................................................................................................

                               <.<<<<<<.<<<<<<.......................................................................................................................................
                               ...............
                               ----------------●●●-●○-----------------------------------------------------------------------------------------------------------------------------
```

```
Ga0265758_104499/242-367         ................AUG.GA................................................................................................................................
Ga0079039_1551950/237-315        ................UUC.GA................................................................................................................................
Ga0157570_1034241/151-229        ................UGU.AC................................................................................................................................
Ga0157571_1064089/212-134        ................UGU.AC................................................................................................................................
Ga0179951_1211246/478-633        ................UAG.GA.UG.AA.A.....G..................................................................................................................
Ga0179950_1197742/286-441        ................UAG.GA.UG.AA.A.....G.....A............................................................................................................
Ga0184578_120646/150-246         ................AUG.GA................................................................................................................................
Ga0247526_105246/689-508         C.CU............UUC.UU.AC.CU.A.....A.....ACGGUUGU.U...................................................................................................
Ga0247538_100442/1363-1181       G.CCUUUC..UACC..UAA.AC................................................................................................................................
Ga0247515_107373/548-731         G.CCUUUC..UACC..UUA.GU................................................................................................................................
Ga0247538_100551/721-542         G.GCCUUU.CAACC..UAA.AU................................................................................................................................
Ga0184588_131258/156-253         U.C.............AUC.A.................................................................................................................................
Ga0316047_100860/1040-888        ................GGC.AC................................................................................................................................
Ga0242657_1476020/160-244        ................UUG.A.................................................................................................................................
Ga0184602_130623/1321-1100       G.U.............UUG.GG.AC.GA.G.....G.....AACUCGUC.CUCCGAGACG.CUCGAG..G.AGAACUC...UU..G.........GCUGACA.....C.AAAUCUC..................................
Ga0153880_1410217/288-397        ................UUA.UA................................................................................................................................
Ga0316049_129960/371-262         ................CAA.AG................................................................................................................................
Ga0265747_125875/320-395         ................CGA.G.................................................................................................................................
Ga0242647_1002473/759-521        ................AAA.GC.CA.AU.U.....G.....AACGUAUG.UCUCCAGUGG.UCCGCG..A.GGACUCU...GG..U.........AGGUCUU.....C.ACUCAAUCGG..UGGCG........GACUUGC..CUGACUG
Ga0242641_1004288/32-112         ................UCA.AG................................................................................................................................
Ga0247553_101094/709-629         ................UCA.AU.GA.C...........................................................................................................................
Ga0127503_10814644/496-352       U.CUUGGA.CCACC..CUC.G.................................................................................................................................
Ga0179939_1199779/67-205         .................GA.C.................................................................................................................................
Ga0180107_1021361/468-373        ................GAG.C.................................................................................................................................
Ga0180107_1218358/314-219        ................AAG.C.................................................................................................................................
Ga0265758_100138/1527-1633       ..........UACCA.AUG.C.................................................................................................................................
Ga0265752_100416/442-337         ................AUG.CC................................................................................................................................
Ga0247534_117029/65-195          ................UCG.AU.AA.AA.A.....G.....AG...........................................................................................................
Ga0187793_1322799/72-151         ................GAU.G.................................................................................................................................
Ga0307921_1005950/791-877        ................AUC.AG................................................................................................................................
Ga0180047_1128081/151-64         ................CUC.GA................................................................................................................................
Ga0157593_1125711/213-128        ................CGC.GA................................................................................................................................
Ga0157569_1032488/120-205        ................CGC.GA................................................................................................................................
Ga0157565_1077069/156-67         ................CUC.GA................................................................................................................................
Ga0157593_1023702/238-314        ................AUC.AA................................................................................................................................
Ga0255267_1268184/91-184         ................ACA.AA................................................................................................................................
Ga0307919_1081825/191-74         ................CUA.G.................................................................................................................................
Ga0307921_1095896/178-61         ................CUA.G.................................................................................................................................
Ga0316040_172648/178-66          ................UUA.C.................................................................................................................................
Ga0184577_110850/246-358         ................UUG.C.................................................................................................................................
Ga0307920_113994/130-9           ................GUU.GA................................................................................................................................
Ga0307920_113994/654-539         ................UUA.C.................................................................................................................................
Ga0307921_1037919/342-457        ................UUG.C.................................................................................................................................
Ga0242646_1001980/23-280         A.CCGCCC.AACCCG.AGC.UC.AA.GG.G.....A.....AU........UCGUAGAGC.ACGCAU..U.UUGACAG...CG..A.........CCCUUGA.....G.AGAGGAGCUC..CAGUC........AGU...A..GUGACCA
Ga0179935_1139075/165-78         ................UGG.A.................................................................................................................................
Ga0179944_1246019/249-162        ................UGG.A.................................................................................................................................
Ga0180044_1069332/269-187        ................CGU.C.................................................................................................................................
Ga0157564_1065218/271-353        ................CGU.C.................................................................................................................................
Ga0180044_1047678/444-368        ................CGU.C.................................................................................................................................
Ga0180047_1061805/548-622        ................UGC.AA................................................................................................................................
Ga0157569_1073490/184-258        ................UGC.AA................................................................................................................................
Ga0213850_1394829/253-105        A.C.............GCC.A.................................................................................................................................
Ga0255293_1138691/440-213        ................UAU.GG.UG.UU.C.....A.....CAACACCU.CACAAGCUUG.AUAAGC..U.UGGCAUUCAUGC..C.........UGGAAGU.....G.AGUAUGUGUA..AUGUG........UAC...U..GAUGAUA
Ga0222728_1000079/1192-1299      ................UGC.GU.UG.UU.G.....C.....AAUCCCAA.G...................................................................................................
Ga0081246_1201717/153-259        ................GUG.CG.UA.AA.G.....C.....AAUCCCAA.GC..................................................................................................
Ga0222728_1216887/51-157         ................GUG.CG.UA.AA.G.....C.....AAUCCCAA.GC..................................................................................................
Ga0265754_1141843/290-185        ................GUG.CG.UA.AA.G.....C.....AGUGCCAA.GCGGCUA.............................................................................................
JGI12272J11983_1345183/364-258   ................GUG.CG.UA.AA.G.....C.....AAUGCCAA.GCGGCUA.............................................................................................
Ga0242647_1005934/451-617        ................UCC.AC................................................................................................................................
Ga0115594_1187657/870-960        ................AGU.AA................................................................................................................................
Ga0115594_1174394/642-729        ................AGU.AA................................................................................................................................
Ga0184569_112764/943-836         ................UUU.GA................................................................................................................................
Ga0265750_1013321/389-496        ................UUU.GA................................................................................................................................
Ga0265757_139522/177-70          ................UUU.GA................................................................................................................................
Ga0316029_116217/368-261         ................GUG.UG.UU.UG.A.....C.....ACAAUCCC.AAGU................................................................................................
Ga0316047_100016/2681-2574       ................GUG.UG.UU.UG.A.....C.....ACAAUCCC.AAGU................................................................................................
Ga0256352_1101982/155-277        ................GGC.UC................................................................................................................................
Ga0256350_1100736/283-405        ................GGC.UC................................................................................................................................
Ga0079039_1443516/199-38         .......................................................................CGCCCUG...GU..G.........ACCGUCA.....G.ACCAGUUCUG..CGUUC........................
Ga0115595_1072788/334-441        ................ACG.CC................................................................................................................................
Ga0115594_1022316/346-239        ................ACG.CC................................................................................................................................
Ga0129331_1058147/189-99         ................UCG.AA................................................................................................................................
Ga0265751_100127/176-103         ................UUU.AA.....C..........................................................................................................................
Ga0307921_1043977/91-164         ................UUA.AA.U..............................................................................................................................
Ga0307919_1048575/242-90         ..CC............ACA.AC.UC.UA.A.....G.....CAACACGU.U...............................................................................................CCUA
Ga0242646_1001086/737-571        C.CAUUGA........UUC.U.................................................................................................................................
Ga0265752_111922/418-343         ................GUG.G.................................................................................................................................
Ga0157562_1134247/69-160         ................CGG.UC................................................................................................................................
Ga0157559_1047848/128-41         ................CGG.UC................................................................................................................................
Ga0179944_1313268/129-232        ................GGC.C.................................................................................................................................
Ga0213850_1259428/445-342        ................UGG.CC................................................................................................................................
Ga0153880_1167367/224-121        ................UGG.CC................................................................................................................................
Ga0153880_1058095/344-241        ................UGG.CC................................................................................................................................
Ga0179943_1178349/195-298        ................UGG.CC................................................................................................................................
Ga0307921_1075885/188-36         ................CAA.CU.CU.AA.G.....C.....AACACGUU.CCAUAGU..............UGGAACG...GC..C.........UAGCAGA.....U.UUAU.....................................
Ga0307921_1062541/180-338        ................CAA.CU.CU.AA.G.....C.....AACACCUC.CUAUCGGAGG.GCCUAG..C.AGAUUUA...AG...................................................................
Ga0242641_1014474/336-257        ................UUC.UU................................................................................................................................
Ga0307919_1064641/225-73         ................CUC.UA.....................................................AGC...AA..C.........ACGUUCC.....A.UAAUUGGAAC..GGCCU........AGC...A..GAUUU..
Ga0265759_108120/277-351         ................AUA.UA................................................................................................................................

                                 <.<<<<<<.<<<<<<.......................................................................................................................................
                                 ......................................................................................................................................................
                                 ----------------●●●-●○--------------------------------------------------------------------------------------------------------------------------------
```

```
Ga0265753_1005277/856-1057       U.A.............UGG.CC............................................................U..U.........GGAUCGA.....A.AUCCGUGGUU..UAGGU........UAA...U..GGUGCAA
Ga0247523_120120/325-403         ................UAA.UC................................................................................................................................
Ga0228707_1100139/344-194        ...AGACG.ACACCG.CUA.AG.UU.AU.A.....G..................................................................................................................
Ga0316049_154526/155-74          ................CUU.UG................................................................................................................................
Ga0316035_177575/224-142         ................UAA.UU.CU.UU.G.....A.....AUUUU........................................................................................................
Ga0307920_126403/177-486         U.CCA..C.CAACCG.GGU.AG.GG.AA.G.....U.....UUUGUUUC.CUAGCACCCU.GGGGUA..C.AAACUAU...GU..U.........ACGAUUC.....A.CUAGGAGCUA..AAGCU........CGU...G..AAUUAAU
Ga0138280_1140856/341-479        ................UCA.G................................................................C.........ACUAUUU.....C.CAAGUGCGAG..CGAAA........GAA...U..GACGCUC
Ga0247539_101233/414-526         ................UUA.AU.U..............................................................................................................................
Ga0184569_108885/200-88          ................UUA.AU.U..............................................................................................................................
Ga0247521_112559/317-205         ................UUA.AU.U..............................................................................................................................
Ga0184599_154888/232-155         ................GAC.A.................................................................................................................................
Ga0180108_1143860/199-116        ................CAC.UG................................................................................................................................
Ga0247524_121352/374-303         ................AUC.U.................................................................................................................................
Ga0206356_11002907/329-148       ................GUG.UA................................................................................................................................
Ga0265743_103007/946-842         ................CAA.GC................................................................................................................................
Ga0316033_101029/151-49          ................AAG.C.................................................................................................................................
Ga0179937_1126086/216-325        ................AGC.GC................................................................................................................................
Ga0307482_1050048/176-95         ................UUU.CG................................................................................................................................
JGI12272J11983_1319142/230-159   ................ACC.CC................................................................................................................................
Ga0180107_1285918/13-113         ................GUA.A.................................................................................................................................
Ga0265756_108694/382-277         ................UCU.AA................................................................................................................................
Ga0265746_1140777/214-319        ................UCU.AA................................................................................................................................
Ga0184574_101359/409-493         ................AUC.U.................................................................................................................................
Ga0184568_108556/391-307         ................AUC.U.................................................................................................................................
Ga0316030_107573/391-475         ................AUC.U.................................................................................................................................
Ga0180107_1031754/139-57         ................CUA.UG................................................................................................................................
Ga0307919_1029784/253-144        ................CUA.GG................................................................................................................................
Ga0307921_1042549/343-452        ................CUA.GG................................................................................................................................
Ga0307482_1021658/484-563        ................UUA.C.................................................................................................................................
Ga0179943_1102267/215-90         ................CUU.GA................................................................................................................................
Ga0079074_1279626/114-274        C.C...........A.GUC.AU.A..............................................................................................................................
Ga0307921_1019702/90-184         ................GGA.G.................................................................................................................................
Ga0307920_133961/233-140         ................GGA.G.................................................................................................................................
Ga0316035_179807/270-184         ................UUC.A.................................................................................................................................
Ga0256338_1343513/152-228        ................UUC.A.................................................................................................................................
Ga0255268_1432700/127-51         ................UUC.A.................................................................................................................................
Ga0153880_1067318/492-566        ................AAA.UC................................................................................................................................
Ga0247533_103075/417-305         ................UUA.U.................................................................................................................................
Ga0247541_102964/56-168          ................UUA.UC................................................................................................................................
Ga0184589_110648/1041-881        ................AUG.UG................................................................................................................................
Ga0184580_124121/239-79          ................AUG.UG................................................................................................................................
Ga0184598_101324/530-690         ................AUG.UG................................................................................................................................
Ga0184588_120624/266-426         ................AUG.CG................................................................................................................................
Ga0184579_106194/996-1156        ................AUG.CG................................................................................................................................
Ga0265758_100254/356-516         ................AUG.CG................................................................................................................................
Ga0184597_106861/196-36          ................AUG.UG................................................................................................................................
Ga0184600_132066/568-408         ................AUG.UG................................................................................................................................
Ga0316031_108320/216-376         ................AUG.UG................................................................................................................................
Ga0184596_109261/979-1139        ................AUG.UG................................................................................................................................
Ga0316035_107544/354-514         ................AUG.UG................................................................................................................................
Ga0247527_115808/280-440         ................AUG.UG................................................................................................................................
Ga0247519_107918/217-58          ................AUG.AG................................................................................................................................
Ga0184589_112135/40-200          ................AUG.CG................................................................................................................................
Ga0247551_100195/490-329         ................AUG.CG................................................................................................................................
Ga0247526_117235/152-313         ................AUG.CG................................................................................................................................
Ga0326631_101563/544-705         ................AUG.UA................................................................................................................................
Ga0184596_111007/525-686         ................AUG.AG................................................................................................................................
Ga0184573_110190/115-276         ................AUG.AG................................................................................................................................
Ga0316033_144939/167-328         ................AUG.AG................................................................................................................................
Ga0247552_101742/612-452         ................AUG.UG................................................................................................................................
Ga0247545_112960/183-343         ................AUG.UG................................................................................................................................
Ga0247541_103784/123-283         ................AUG.UG................................................................................................................................
Ga0247524_111427/50-210          ................AUG.UG................................................................................................................................
Ga0247545_104341/406-246         ................AUG.UG................................................................................................................................
Ga0184602_114416/11-171          ................AUG.UG................................................................................................................................
Ga0316038_111670/11-171          ................AUG.UG................................................................................................................................
Ga0184586_133850/368-528         ................AUG.UG................................................................................................................................
Ga0184600_123882/188-28          ................AUG.CG................................................................................................................................
Ga0184574_121290/269-109         ................AUG.UG................................................................................................................................
Ga0316032_107575/215-55          ................AUG.UG................................................................................................................................
Ga0247519_110701/607-447         ................AUG.UG................................................................................................................................
Ga0247526_110221/603-443         ................AUG.UG................................................................................................................................
Ga0247529_134913/224-64          ................AUG.UG................................................................................................................................
Ga0247529_103231/270-430         ................AUG.AG................................................................................................................................
Ga0184580_114961/306-146         ................AUG.AG................................................................................................................................
Ga0184603_126764/80-240          ................AUG.AG................................................................................................................................
Ga0247550_114736/167-7           ................AUG.AG................................................................................................................................
Ga0184603_111918/241-82          ................AUG.AG................................................................................................................................
Ga0247538_100424/120-281         ................AUG.AG................................................................................................................................
Ga0247547_102464/189-29          ................AUG.AG................................................................................................................................
Ga0247551_100828/546-387         ................AUG.AG................................................................................................................................
Ga0247538_100480/788-947         ................AUG.AG................................................................................................................................
Ga0247550_100850/456-297         ................AUG.AG................................................................................................................................
Ga0247536_101914/360-201         ................AUG.AG................................................................................................................................
Ga0247553_110698/526-367         ................AUG.AG................................................................................................................................
Ga0153880_1114976/150-383        ...................................A.....AAAGUAGG.GAAGUAUCAC.GCAUAU..U.UUGAACC...GG..G.........AGCAUUG.....C.UCAACCGACA..AACUU........UGG...C..GCACAAC
Ga0180052_189627/240-346         ................AGU.UA..G.............................................................................................................................
Ga0184598_136114/144-251         ...........ACCA.AAU.GU................................................................................................................................
Ga0222728_1011988/841-947        ................AUU.GU.C..............................................................................................................................

                                 <.<<<<<<.<<<<<<.......................................................................................................................................
                                 ......................................................................................................................................................
                                 ----------------●●●-●○--------------------------------------------------------------------------------------------------------------------------------
```

```
Ga0265751_160438/108-215           ................AGA.AG....................................................................................................................................
Ga0247514_107497/1071-1140         ................UAC.G.....................................................................................................................................
Ga0180107_1129325/244-60           ................UUU.GA.UU.UG.....................G.UCAUCGAUCA.AAUCUU..G.CGAUUAC...AG..U.........GCCAUUG...................................................
Ga0242646_1002014/11-270           A.GA............CAC.AU.UC.GU.G......C.....UACGUGCU.UGACUUCGAG.CGCAAG..C.AAGCAUC...AU..U.........CCACAUA.....A.AAACCAUAAA..AAAAG.........AUA...G..G........
Ga0222728_1008526/24-283           A.GA............CAC.AU.UC.GU.G......C.....UACGUGCU.UGACUUCGAG.CGCAAG..C.AAGCAUC...AU..U.........CCACAUA.....A.AAACCAUAAA..AAAAG.........AUA...G..G........
Ga0222728_1008391/1281-1023        A.GA............CAC.AU.UC.GU.G......C.....UACGUGCU.UGACUACGAG.CGCAAG..C.AAGCAUC...CU..U.........CCACAUA.....A.AAACCA....................................UA
Ga0265759_100017/4211-4288         ................AAU.GC....................................................................................................................................
Ga0247512_104440/911-1082          ................CUU.U.....................................................................................................................................
Ga0265756_131580/159-58            ................UUC.AA..A.................................................................................................................................
Ga0310119_169621/253-177           ................UUU.GU....................................................................................................................................
Ga0247516_124175/202-272           ................UUA.U.....................................................................................................................................
Ga0247534_100772/309-234           ................CGU.GC....................................................................................................................................
Ga0222728_1054736/119-193          ................UUG.AA.U..................................................................................................................................
Ga0180106_1297567/444-515          ................CUC.GU....................................................................................................................................
Ga0242647_1000066/2638-2719        ................CGU.U.....................................................................................................................................
Ga0307484_104189/90-171            ................UUG.CU.AA.A...............................................................................................................................
Ga0316048_109289/45-149            ................AAC.U.....................................................................................................................................
Ga0265750_1038125/531-427          ................AAC.U.....................................................................................................................................
Ga0247537_109516/130-26            ................AAC.U.....................................................................................................................................
Ga0310115_103434/744-848           ................AAC.U.....................................................................................................................................
Ga0307482_1009758/1126-1021        ................AUG.U.....................................................................................................................................
JGI12272J11983_1356304/1548-1654   ................UCG.C.....................................................................................................................................
JGI12272J11983_1072695/645-539     ................UAU.GC.U..................................................................................................................................
Ga0242657_1009512/820-926          ...U............AUG.C.....................................................................................................................................
Ga0316037_172473/89-196            ................AUA.UC....................................................................................................................................
Ga0184592_121084/378-271           ................AUA.UC....................................................................................................................................
Ga0184569_113518/504-609           ..............A.ACG.C.....................................................................................................................................
Ga0184602_114837/392-286           ................ACG.C.....................................................................................................................................
Ga0316030_102131/386-280           ................AUG.C.....................................................................................................................................
Ga0310118_104334/336-443           ................AAG.C......................G..............................................................................................................
Ga0180108_1146973/462-263          A.C.............GUC.CA.UA.UU.U.................................................................................................GAU........UGU...U..GAGCUUA
Ga0184580_111164/678-749           ................CUU.CA....................................................................................................................................
Ga0242647_1006852/526-598          ................AAC.AU....................................................................................................................................
Ga0179939_1059820/107-216          ................AGC.UC....AG..............................................................................................................................
Ga0115594_1156252/268-370          ................GUA.UC....................................................................................................................................
Ga0115594_1006122/120-29           ................CUU.AA.UA.AC.G......A.....AA..............................................................................................................
Ga0115595_1180708/226-317          ................CUU.AA.UA.AC.G......A.....AA..............................................................................................................
Ga0115594_1211214/299-389          ................AUA.AA.GC.UC.A......A.....A...............................................................................................................
Ga0115594_1007070/120-30           ................UUA.AU.AA.AC.G......A.....AA..............................................................................................................
Ga0115595_1181384/176-86           ................UUA.AU.AA.AC.G......A.....AA..............................................................................................................
Ga0115594_1166236/119-19           ................AUA.AA.GG.CA.G......U.....AUC.............................................................................................................
Ga0115594_1157594/155-254          ................AUA.CC....................................................................................................................................
Ga0256352_1182139/180-81           ................GUA.CC....................................................................................................................................
Ga0115594_1182529/183-84           ................GUA.CC....................................................................................................................................
Ga0265746_1064626/389-458          ................AUC.CG....................................................................................................................................
Ga0115594_1016490/326-410          ................CUA.AA....................................................................................................................................
Ga0115595_1007417/328-412          ................CUA.AA....................................................................................................................................
Ga0228708_1103570/188-285          ................AUU.U.....................................................................................................................................
Ga0265751_121301/189-266           ................UUG.AA.G..................................................................................................................................
Ga0179944_1005311/261-382          ................CGC.AU.GA.................................................................................................................................
Ga0179944_1191552/284-411          ................CGC.UU.GA.................................................................................................................................
Ga0179943_1150468/216-89           ................UAC.AC....................................................................................................................................
Ga0179944_1204933/216-89           ................UAC.AC....................................................................................................................................
Ga0307482_1020425/440-614          C.CAGGAC.UCAAUA.CUC.UG....................................................................................................................................
Ga0242646_1001395/255-509          ................CGC.AU..........................................................UUUG...AC..A........ACGACCC....G.CAAGGAGCUC..UAGUC........AGU...G..GUGACCA
Ga0184578_112031/156-44            ................UUA.G.....................................................................................................................................
Ga0184577_120131/220-108           ................UUA.G.....................................................................................................................................
Ga0247517_121471/387-499           ................UUU.AG.U..................................................................................................................................
Ga0247539_107237/153-229           ................AAA.U.....................................................................................................................................
Ga0265758_109576/83-157            ................UUG.UU....................................................................................................................................
Ga0184577_107529/254-327           ................UUU.G.....................................................................................................................................
Ga0213850_1246156/171-47           ................AUU.AG.GC.AG.A......C.....ACCCAUGC.C......................................................................................................
Ga0326631_172366/284-207           ................GCU.AC....................................................................................................................................
Ga0310114_149639/68-153            ................UAC.CA....................................................................................................................................
Ga0184599_128731/188-273           ................UAC.CA....................................................................................................................................
Ga0310119_131358/533-447           ................UAC.CA....................................................................................................................................
Ga0247526_116548/279-354           ................CUA.G.....................................................................................................................................
Ga0247519_113845/334-409           ................UUA.G.....................................................................................................................................
Ga0247529_111070/303-228           ................UUA.G.....................................................................................................................................
Ga0184580_116530/249-177           ................AGA.UA....................................................................................................................................
Ga0247524_105844/561-637           ................GAA.U.....................................................................................................................................
Ga0184593_119849/313-238           ................AGA.AU....................................................................................................................................
Ga0184599_140898/774-849           ................AGA.AU....................................................................................................................................
Ga0316049_111814/256-181           ..................G.AA.U..................................................................................................................................
Ga0316034_100493/254-174           ................AGA.GU....................................................................................................................................
Ga0184600_118565/177-97            ................AGA.GU....................................................................................................................................
Ga0184602_122864/595-515           ................GAC.UA....................................................................................................................................
Ga0184596_126546/83-162            ................GAC.UA....................................................................................................................................
Ga0184597_129399/366-446           ................UAG.AA....................................................................................................................................
Ga0184576_117703/380-460           ................UAG.AA....................................................................................................................................
Ga0184590_111002/143-223           ................UAG.A.....................................................................................................................................
Ga0247551_103383/322-242           ................UAG.A.....................................................................................................................................
Ga0184597_111165/262-182           ................UAG.A.....................................................................................................................................
Ga0184570_116436/278-198           ................UAG.A.....................................................................................................................................
Ga0316037_169124/279-199           ................UAG.A.....................................................................................................................................
Ga0247543_103170/244-164           ................UAG.A.....................................................................................................................................
Ga0184589_120451/249-329           ................UAG.A.....................................................................................................................................
Ga0247519_108375/286-206           ................UAG.A.....................................................................................................................................
Ga0184587_105985/71-151            ..................G.UA....................................................................................................................................

                                   <.<<<<<<.<<<<<<...........................................................................................................................................
                                   ..........................................................................................................................................................
                                   ----------------●●●-●○------------------------------------------------------------------------------------------------------------------------------------
```

```
Ga0184593_101086/682-762         .................UG.UA...................................................................................................................................
Ga0184602_121948/324-245         ................GAA.UA...................................................................................................................................
Ga0184597_110829/1090-1011       ................GAA.UA...................................................................................................................................
Ga0184578_129447/314-235         ................UAG.AA.UA.UG.............................................................................................................................
Ga0184588_113246/331-252         ................UAG.AA.UA.G..............................................................................................................................
Ga0316051_1003245/138-56         ................UGG.AA.UA.U..............................................................................................................................
Ga0316046_122965/221-142         ................UGG.AA.UA.U..............................................................................................................................
Ga0184600_105920/81-160          ................UAG.AA.UA.AA.............................................................................................................................
Ga0184603_125382/9-88            ................AGA.A....................................................................................................................................
Ga0247550_100138/208-129         ................AGA.A....................................................................................................................................
Ga0316049_122425/209-288         ................AAG.AA.UA................................................................................................................................
Ga0032354_1062690/2659-2586      ................CUC.G....................................................................................................................................
Ga0179955_1048341/123-51         ................UAC.AU...................................................................................................................................
Ga0138277_1023917/182-18         ................UUC.CA......................................................................................................AAUGC........AGC...G..AUUCUUA
Ga0138282_1020546/737-573        ................UUC.CA......................................................................................................AAUGC........AGC...G..AUUCUUA
Ga0242641_1105635/265-351        ................UCA.GA...................................................................................................................................
Ga0242654_10795375/263-349       ................UCA.GA...................................................................................................................................
Ga0247554_113506/254-161         ................UAG.CA...................................................................................................................................
Ga0265745_1000618/793-552        U.A.............UUG.UC.UU.AU.G.....A.....CUCUGGAU.CUCUCCACUC.GCCCGG..A.UGGCACA...CU..U........UCCUCAG.....U..............................................
Ga0265742_1001052/322-80         U.G.............UUG.UC.UU.AC.G.....A.....CUCUGGAC.CUCUCCUCGU.GAUCGG..A.UGGCACA...CU..U........UCCUCAG.....U..............................................
Ga0310113_106868/972-730         U.G.............UUG.UC.UU.AC.G.....A.....CUCUGGAC.CUCUCCUCGU.GAUCGG..A.UGGCACA...CU..U........UCCUCAG.....U..............................................
Ga0265750_1009219/272-30         U.U.............UCG.UC.UU.AU.G.....A.....CUCUGGAC.CUCUCCUCGU.GAUCGG..A.UGGCACA...CU..U........UCCUCAG.....U..............................................
Ga0265740_1001378/1084-842       U.U.............UCG.UC.UU.AU.G.....A.....CUCUGGAC.CUCUCCUCGU.GAUCGG..A.UGGCACA...CU..U........UCCUCAG.....U..............................................
Ga0265752_100504/930-688         U.U.............UCG.UC.UU.AU.G.....A.....CUCUGGAC.CUCUCCUCGU.GAUCGG..A.UGGCACA...CU..U........UCCUCAG.....U..............................................
Ga0265747_100653/495-253         U.U.............UCG.UC.UU.AU.G.....A.....CUCUGGAC.CUCUCCUCGU.GAUCGG..A.UGGCACA...CU..U........UCCUCAG.....U..............................................
Ga0265757_104669/135-376         U.A.............UUG.UC.UU.AU.G.....A.....CUCUGGAU.CUCUCCACUC.GCCCGG..A.UGGCACA...CU..U........UCCUCAG.....U..............................................
Ga0265742_1088739/51-292         U.A.............UUG.UC.UU.AU.G.....A.....CUCUGGAU.CUCUCCACUC.GCCCGG..A.UGGCACA...CU..U........UCCUCAG.....U..............................................
Ga0265747_100509/1277-1036       U.A.............UUG.UC.UU.AU.G.....A.....CUCUGGAU.CUCUCCACUC.GCCCGG..A.UGGCACA...CU..U........UCCUCAG.....U..............................................
Ga0265752_100314/1466-1225       U.A.............UUG.UC.UU.AU.G.....A.....CUCUGGAU.CUCUCCACUC.GCCCGG..A.UGGCACA...CU..U........UCCUCAG.....U..............................................
Ga0213850_1450246/323-606        C.CA..AC.CUACCG.UCU.GA.AC.CG.A.....C.....CCCACUUC.UUAGUGG...............................................................GG..UAGGG........GAC...G..GAUGAGC
Ga0157593_1101573/285-478        ................UAG.UU.........................................................................................................CA........UAG...G..GUUGCUU
Ga0222728_1000125/1315-1238      ................ACA.U....................................................................................................................................
Ga0242654_10000586/1273-1196     ................AUG.U....................................................................................................................................
Ga0265756_100130/1310-1239       ................CAC.UG...................................................................................................................................
Ga0307482_1008742/1212-1288      ................UUC.AU...................................................................................................................................
Ga0242654_10040657/1018-942      ................GCA.A....................................................................................................................................
Ga0307484_100842/1194-1271       ................UUC.A....................................................................................................................................
Ga0307482_1009223/670-508        ................AUA.UA.UG.AU.G.....U.....UUAUCCGG.GUAAUUCCGG.GACC..........UC...AU..C.................................................................
Ga0307484_108094/186-350         ................AUA.UA.UG.AU.G.....U.....UUAUCCGG.GUAAUUCCGG.GACC..........UC...AU..C.................................................................
Ga0247514_114593/280-211         ................UUU.UU...................................................................................................................................
Ga0184583_106441/296-227         ................UUU.U....................................................................................................................................
Ga0242654_10672177/244-165       ................UUU.AC...................................................................................................................................
Ga0126318_10229384/609-688       ................UCA.AC...................................................................................................................................
Ga0247551_122551/29-115          ................GUU.UA...................................................................................................................................
Ga0316048_107802/239-324         ................GUU.A....................................................................................................................................
Ga0242641_1002064/1203-1116      ................AUU.UA...................................................................................................................................
Ga0307482_1026216/884-813        ................CUA.AG...................................................................................................................................
Ga0307482_1636410/270-341        ................CUU.AG...................................................................................................................................
Ga0307482_1016877/168-97         ................CUU.GA...................................................................................................................................
Ga0180107_1106188/424-495        ................UUU.UA...................................................................................................................................
Ga0307482_1000354/2063-1987      ................UAU.U....................................................................................................................................
Ga0184587_107783/167-95          ................ACA.A....................................................................................................................................
Ga0184585_128240/467-390         ................UCC.AU...................................................................................................................................
Ga0184596_102741/625-701         ................CUA.G....................................................................................................................................
Ga0247539_100086/1763-1850       ................UUU.AU...................................................................................................................................
Ga0184584_115572/267-354         ................UUU.AU...................................................................................................................................
Ga0184579_105257/1735-1648       ................UUU.AU...................................................................................................................................
Ga0307482_1023270/273-197        ................CUA.AA.C.................................................................................................................................
Ga0307482_1219330/19-95          ................AGU.AU...................................................................................................................................
Ga0242657_1207391/462-383        ................UCA.CA...................................................................................................................................
Ga0247520_104849/891-821         ................UCC.G....................................................................................................................................
Ga0247531_104861/1082-1012       ................UCC.G....................................................................................................................................
Ga0247518_137824/301-231         ................UUC.G....................................................................................................................................
Ga0307482_1202605/470-395        ................UGU.GC...................................................................................................................................
Ga0307921_1021783/160-261        ................AGA.GG.GA.G..............................................................................................................................
Ga0242657_1054283/541-620        ................UAA.CU...................................................................................................................................
Ga0242647_1005188/439-360        ................UAA.CU.UA.AA.............................................................................................................................
Ga0153880_1391790/62-140         ................AUC.AA...................................................................................................................................
Ga0153880_1379927/66-144         ................AUG.AA...................................................................................................................................
Ga0307482_1023665/853-778        ................GUG.G....................................................................................................................................
Ga0242646_1047905/92-182         ................AUC.C....................................................................................................................................
Ga0307921_1024041/406-334        ................CUA.AA...................................................................................................................................
Ga0307919_1039507/513-440        ................UAA.AA...................................................................................................................................
Ga0307919_1084008/308-378        ................AUU.AU...................................................................................................................................
Ga0247525_114510/199-318         ................UUC.AC...................................................................................................................................
Ga0184603_107262/477-596         ................UUU.AC...................................................................................................................................
Ga0184585_151549/495-376         ................UUC.AC...................................................................................................................................
Ga0310119_113172/381-306         ................UCU.C....................................................................................................................................
Ga0310114_133004/56-132          ................UCU.C....................................................................................................................................
Ga0184603_148109/996-920         ................UCU.C....................................................................................................................................
Ga0184599_110049/275-349         ................UCU.C....................................................................................................................................
Ga0184587_132427/565-485         ................UCU.C....................................................................................................................................
Ga0265756_135986/152-290         ................CUU.GC...................................................................................................................................
Ga0247519_104852/800-730         ................AUC.C....................................................................................................................................
Ga0213850_1274705/160-417        C.C.............UCA.UC.UC.......................U.AAGUUGACCU.GCUAAC..G.CGGGAAC...AA..C........UGGUUUG.....A.GUAAGUGGGG..GGGAG........UUC...UCCGUAACUG
Ga0265747_100454/1395-1324       ................UAU.G....................................................................................................................................
Ga0247519_118271/334-405         ................GAC.AU.UA................................................................................................................................
Ga0242654_10135580/213-336       ................UUC.AC................................................................................................GAUAGG..AAAUU........AAC...A..GUUUCUA
Ga0184576_110410/277-348         ................UCA.C....................................................................................................................................

                                 <.<<<<<<.<<<<<<..........................................................................................................................................
                                 .........................................................................................................................................................
                                 ----------------●●●-●○-----------------------------------------------------------------------------------------------------------------------------------
```

```
Ga0247535_105396/277-348     ................UCA.C.................................................................................................................................
Ga0265758_104558/436-365     ................CAA.C.................................................................................................................................
Ga0247526_117664/434-499     ................UUA.U.................................................................................................................................
Ga0316037_123461/289-368     ................CAU.CA................................................................................................................................
Ga0247543_100863/642-721     ................CAU.CA................................................................................................................................
Ga0247521_124040/388-309     ................CAU.CA................................................................................................................................
Ga0184589_120335/11-90       ................CAU.CA................................................................................................................................
Ga0247518_108423/758-837     ................CAU.CA................................................................................................................................
Ga0247531_104608/1256-1177   ................CAU.CA................................................................................................................................
Ga0265750_1365815/134-55     ................CAU.UA................................................................................................................................
Ga0184597_121517/262-183     ................CAU.CA................................................................................................................................
Ga0265747_102820/448-376     ................UUC.GA................................................................................................................................
Ga0247554_105388/388-316     ................UUC.GA................................................................................................................................
Ga0307482_1015330/1021-1093  ................CAA.GA.UA.UA..........................................................................................................................
Ga0184600_102725/60-135      ................UUG.U.................................................................................................................................
Ga0184602_122974/190-265     ................UUU.GU................................................................................................................................
Ga0316038_112263/442-356     ................AUG.GC................................................................................................................................
Ga0307484_161007/136-39      ................GUU.A.................................................................................................................................
Ga0307482_1033688/516-422    ................CAG.U.................................................................................................................................
Ga0316031_132099/178-294     ................CGU.U.................................................................................................................................
Ga0213850_1220553/385-463    ................GUU.AA................................................................................................................................
Ga0180107_1307618/257-174    ................GCU.UA................................................................................................................................
Ga0265747_119090/8-84        .................UA.GG.UG.UA.A........................................................................................................................
Ga0242646_1115622/191-109    ................AAU.U.................................................................................................................................
Ga0184570_114412/209-338     ................UCG.AA................................................................................................................................
Ga0265757_143567/145-70      ................UUG.GA................................................................................................................................
Ga0184568_109812/391-283     ................GCA.A.................................................................................................................................
Ga0184568_104528/922-1000    ................CAA.AA................................................................................................................................
                             <.<<<<<<.<<<<<<.......................................................................................................................................
                             ......................................................................................................................................................
                             ----------------●●●-●○--------------------------------------------------------------------------------------------------------------------------------
```

alignment positions 301 · · · 450

```
Ga0247541_102964/376-269     ...................................................................................UGUGGUGU.CC.GGU.........................................C.UUUG.A.A.
Ga0182091_1018330/100-226    ....................................................................................................GCGCAU.C.GA...C.UAGAA......................GA.A.A.
Ga0316035_118720/195-42      ....................................................................................UACGAG.......A.CACA.UC.A......C.CUACGUGU..................CAA.A.A.
Ga0179944_1246019/41-131     .............................................................................................................................................GGUA.G.A.
Ga0265747_125480/96-202      ..................................................................................................................G......................AUU.AAGU.A.A.
Ga0265757_115889/161-280     ....................................................................................................................GUGAC..................G.UACA.A.A.
Ga0180107_1031754/4443-4551  ....................................................................................................................GGUGA................UGG.UAAC.G.G.
Ga0180107_1229893/426-318    .........................................................................................................................................CGA.UUUC.A.A.
Ga0222728_1038816/522-635    ..................................................................CA.AA......................................................................CAUC.C.G.
Ga0179955_1195671/481-563    ...........................................................................................................................................U.GGUG.C.A.
Ga0265751_100560/274-382     ..............................................................................................................G...U.GAA....................G.CGAU.A.G.
Ga0265751_124015/160-14      .............................................................................................................GA...C..CG....................A.CCUU.A.A.
Ga0316029_122532/20-172      ....................................................................................................UUGUGC.A.UA...A.GCGAGCGGCUGC...........C.UAAC.A.A.
Ga0316031_106349/204-348     .............................................................................................................UU...G.CGUACU...............GCU.CAAC.A.A.
Ga0265759_136872/100-7       ........................................................................UGGA.A.U....................................ACAAG.......................U.C.G.
Ga0138291_1027114/229-62     ........................................................................GUGAUA.CCGAAGGUUGCC.UC.CUGGGGCGAUG.G.CU...A.UUUAAUG..............CUG.AAAG.C.A.
Ga0138291_1177333/356-213    ........................................................................GGGAUC.UCGAU.....GU.AG.UUU.GGCUGU.........G.UUAAA..................G.UACA.C.A.
Ga0079074_1152161/13-203     G..AAU.....A.....GGA..AAUA.....AAUGGUU......CUU...............................................................................................CGU.U.A.
Ga0242657_1353977/135-311    C..AAC.....A.....U..............ACAAUA......UGUUAUGUAGAU...UAU..CAA........................................................................U.UUUC.A.G.
Ga0255293_1180932/373-295    ..................................................................................................................U.A....................UGU.GUGU.G.A.
Ga0307482_1147486/107-4      ..............................................................................................................G...G.UGGGAGG..............CU..ACUU.G.G.
Ga0242647_1001226/1011-1115  .............................................................................................................UC...G.ACUGAUGUUGGCCA............CCC.A.G.
Ga0316032_128094/136-241     ...................................................................................CGAUGUUG.GC.CA.............................................CCC.G.A.
Ga0184598_118707/299-404     ........................................................................C...........GGUGUUG.GC.C.............................................ACCC.A.G.
Ga0213850_1470830/321-202    ..............................................................................................................U...G.GGAACC................GU.GCAU.U.A.
Ga0307482_1003156/2075-2177  ....................................................................................................................AAG..................CUA.GGG..U.U.
Ga0307482_1000291/592-698    .........................................................................................................................................CUA.GGGU.U...
Ga0247532_117056/43-239      C..GAC.....C.....UCU..UCUC.....UACUGUC......UUCGCUGUCUUC...GGA..UA............................................A...U.GACAG...............GUAC.UUGU.C.G.
Ga0184582_120088/374-570     ...........C.....UCU..UCUC.....UACUGUC......UUCGCUGUCUUC...G............................................................GAUAAUGACAG.....GUAC.UUGU.C.G.
Ga0265742_1013613/579-453    ........................................................................CACUCG.ACGUC..........................................................CCU.G.G.
Ga0265748_110851/279-405     ........................................................................CACUCG.ACGUC..........................................................CCU.G.G.
Ga0307482_1000262/4292-4390  ..................................................................................................................G.GGUUGUGA..............C..GUAG.U.A.
Ga0307484_100453/182-280     ..................................................................................................................G.GGUUGUGA..............C..GUAG.U.A.
Ga0247538_120078/87-186      ..................................................................................................................G.GAUGUAGA.................CCAG.U.G.
Ga0307484_109934/368-484     ....................................................................................................................GCGCAA...............UAG.UAUC.A.A.
Ga0184572_111224/187-319     .........................................................................................AU.GC.GGG.AGUACAG.G.UU...U.AGA...........................U.G.
Ga0265744_102237/225-365     .................................................ACCC........................................U.CAG.CCUAAA.....G...G.AGUGA................C.C.AGAU.G.A.
Ga0265758_100384/785-1040    C..GUC.....A.....CGG..GGAU.....CACCGGA......AUCUUAGUUCCU...ACG..GAUC.AGC..............CCUAA.GA.GCC.GCACUAG.U.CC...A.AGGAGUGA....C..........C.CAAU.A.G.
Ga0265753_1005391/776-521    C..GUC.....A.....CGG..GGAU.....CACCGGA......AUCUUAGUUCCU...ACG..GAUC.AGC..............CCUAA.GA.GCC.GCACUAG.U.CCAAGG.AGUGAC.................C.CAAU.A.G.
Ga0265759_100623/780-1036    G..UCA.....C.....GGG..GAUCACCGGAAUCUUA......GUUCCUACGGAUCAGCCC..UAAG.AGC.................CG.CA.CUA.GUCCAAG........G.AGUGAC.................C.CAAU.A.G.
Ga0247535_113123/229-119     .........................................................................................................................................CGA.GGGG.G.G.
Ga0316036_112404/482-376     ...........................................................................................................................................G.AGGG.A.G.
Ga0247515_120448/91-178      ..................................................................................................................................................A.G.
Ga0247518_120275/202-289     ..................................................................................................................................................A.G.
Ga0307482_1000311/1469-1363  ..................................................................................................................G.GGUGAU...............CGG.AUUC.C.G.
Ga0184603_150346/629-518     ..............................................................................................................C...G.GGUAGG...................AUUC.A.G.
Ga0307484_102855/507-659     ............................................................CC..AAAC.UAC............................CCA.GG.C.UG...G.UUCGCA................CG.CUCU.G.G.
Ga0316029_101396/632-732     ....................................................................................................................GGCUA..............CACGU...AU.A.G.
Ga0184582_100659/424-334     .............................................................................................................AG...G.CUCUCC.....................CU.U.G.
Ga0265754_1040230/51-148     .............................................................................................................UA...U.UUC..................CAA.GGUA.U.G.
Ga0316039_120091/354-252     .........................................................................................................C.C.GA...G.UUGAC.......................U.G.G.
Ga0307921_1050750/452-358    ..................................................................................................................A......................GAG.UUCA.G.G.

                             ........................................................................>>>>>>.>>>>>>>>>>>>.>>.>>>.>>>>>>>.>.>>...>.>>>...............>>>>>>.>>>>.>.>.
                             ..................................................................................................................2.222....................2.2222.2.2.
                             ------------------------------------------------------------------------------------------------------------------○-○○○○-------------------○-○◐●●-●-G-
```

```
Ga0265759_141590/154-258     ...................................................................................................................A.GACAGUGU.............AUC.AUCC.U.A.
Ga0184602_130775/1275-1431   ...............................................................................................................A...A.GGUCCU............AUGCUC..AGA.G.U.
Ga0213850_1052387/326-212    .....................................................................................................................GUU....................C.ACUC.A.A.
Ga0213850_1393213/656-542    .....................................................................................................................GUU....................C.ACUC.A.A.
Ga0184578_112042/249-140     ..........................................................................................................G.C.GA...A.UAGAAUCG....................A.U.G.
Ga0316033_100036/207-98      ..........................................................................................................G.C.GA...A.UAGAAUCG....................A.U.G.
Ga0247534_100007/116-225     ..........................................................................................................G.C.GA...A.UAGAAUCG....................A.U.G.
Ga0184569_109084/220-111     ..........................................................................................................G.C.GA...A.UAGAAUCG....................A.U.G.
Ga0206355_1134705/241-332    ..........................................................................................................................................CGU.AUAC.A.A.
Ga0265754_1007706/448-552    ............................................................................................................A.UG...A.A......................C.CUGU.A.G.
Ga0213850_1056422/14-139     ...............................................................................................................U...U.CACCACCUUAACC.............GUC.G.A.
Ga0180107_1033011/786-679    .....................................................................................................UCCAAG.C.GG...A.GAA....................G.UAGU.G.G.
Ga0307483_1000720/1178-1064  ...............................................................................................................U...G.GAGACUGUAACCCA....AUACG..AGUC.A.G.
Ga0265755_100088/1451-1216   ..................AU..GACC.....UGCGACC......AAUGUACGUACC...GGC..AGAG..................G...GC.UC.CGG.UUGGUAU...CG...G.AGCCAGAAAAGCCAA.C.CU...C.UUCA.C.G.
Ga0310119_125223/193-319     ...............................................................................................................A...U.CGCA.................CUA.AUGG.C.A.
Ga0265754_1037910/322-179    ....................................CUU.....C......................................................C.GCGCUG...GC...U.UCAAAGGCGUGUG..........C.UCUA.A.C.
Ga0316053_162144/16-125      ...................................................................................................................C.GGUA.................GAA.UCUG.U.G.
Ga0242654_10007932/343-135   U..GUA.....A.....UCC..GGUA.....UAGGCUA......GCG......................................................................GGAGAC.................C.AUCC.A.A.
Ga0153880_1315932/142-46     ............................................................................................................C.AU...G.GUACA................ACA.AGAG.A.C.
Ga0265750_1009277/794-905    ........................................................................................................GUG.C.UC...A.AAG....................G.CGGA.A.C.
Ga0265756_100928/447-557     ........................................................................................................GUG.C.UC...A.AAG....................G.CGGA.A.C.
Ga0265745_1004075/525-659    ........................................................................................................GUG.C.UC...A.AAG....................G.CGGA.A.C.
Ga0138291_1147840/370-201    ................................................................................CAGUUGCCUCUU.CG.GGG.CGAUGGC.U.AU...U.UAAUG................UUA.AAAG.C.A.
Ga0138291_1027295/231-62     .........................................................................CA.......GUUGCCUCUU.CG.GGG.CGAUGGC.U.AU...U.UAAUG................CUG.AGAG.C.A.
Ga0184602_109114/416-317     ..........................................................................................................................................GUG.CGCA.C.A.
Ga0153880_1354682/355-454    ..........................................................................................................................................GUG.CGCA.C.A.
Ga0242649_1016743/464-237    C..ACA.....A.....AAA..GUGA.....AAACGUU......UACGAGGCUUAC...CAA..G.........................................C.C.UG...U.UUUCACAA...............C.CAGA.U.A.
Ga0179943_1118543/65-150     ............................................................................................................................................U.CUUA.A.G.
Ga0184568_108419/492-395     ...................................................................................................................G.GCU...................GC.UUAU.G.A.
Ga0265756_117960/316-418     ..............................................................................................................CG...A.UGGUGAC..............AAC.AACG.U.G.
Ga0265759_100202/139-258     ......................GCCC.....AAAUAC.................................................................CG..U.G.GG...U.UCAAACG................C.UAUA.U.G.
Ga0265748_107452/280-387     ...................................................................................................................G.UCAAGCGACUAUGAC...........AUG.U.G.
Ga0307484_149222/136-234     ...................................................................................................................A.CCA....................C.ACUA.U.G.
Ga0316038_107364/55-195      ...........................................................................................GU....UU.CUUCGUG.U.UG...A.UUAGAC.................A.CAAG.G.G.
Ga0184593_123391/157-297     ...........................................................................................GU....UU.CUUCGUG.U.UG...A.UUAGAC.................A.CAAG.G.G.
Ga0242652_1159268/284-150    ..........................................................................................UG.CU.CGU.AAAAUUC.G.UG...U.GC.......................CCAA.G.G.
Ga0310117_113093/724-511     ............................................................................UCC.UGAGGGUGA.UU.UC.C..................U.CGGGGAUACCGGUCC.A......C.UUCA.C.G.
Ga0179944_1119724/70-159     ..........................................................................................................................................AGA.GCCU.C...
Ga0316036_104840/706-578     .....................................................................................AAGGAGU.U..CUG..CACU..........A.GUCAG.....................AUU.C.A.
Ga0247538_106858/350-156     .........................................................................CGGUCA.CGGUACUGUGUA.AC.CAG.UGGCUUC.U.........AAGGAGUGUG............C.UCUU.G.G.
Ga0180107_1215312/308-184    .................................................GUCUCAC...AUC..AAA................................................G.AGCAGU.................A.CCUA.U.G.
Ga0247534_100003/3147-3245   ........................................................................................................................................CUCG..UUGU.G.G.
Ga0247519_101377/1717-1907   .....................................................................................GGGGUUU.GA.CUG......GA.C.GG...U.GGGCGUGC.............CUC.CAUA.C.G.
Ga0184588_143621/642-452     .....................................................................................GGGGUUC.GA.CUG......GA.C.GG...U.GGGCGUGC.............CUC.CAUA.C.G.
Ga0265758_100017/4028-4134   .........................................................................................................................U................AUC.AUAG.C.G.
Ga0265751_102572/231-125     .........................................................................................................................U................AUC.AUAG.C.G.
Ga0310106_100226/1503-1738   ....................................................................................GCUCCAGU.UG.G........UA.U.UG...G.AGCCAGAAAAGCCAA.C....CUC.UUCA.C.G.
Ga0184596_112078/314-191     ..............................................................................................................CC...A.UACAU.....................AGU.C.A.
Ga0247528_108647/438-562     ..............................................................................................................CC...A.UACAU.....................AGU.C.A.
Ga0184602_108471/49-173      ..............................................................................................................CC...A.UACAU.....................AGU.C.A.
Ga0316031_112736/216-74      ..............................................GUUAACGAAU...AAC..GACC....................................UAU.C.CA...A.UGGAG..................A.UGAG.A.G.
Ga0184575_106581/174-318     ..............................................GUUAACGAAU...AAC..GACC....................................UAU.C.CA...A.UGGAG..................A.UGAA.A.G.
Ga0184582_129189/472-619     .........................................................................................................................A.............C....C.CCGC.G.G.
Ga0180052_116799/419-501     ...............................................................................................................................................AGC.G.A.
Ga0179955_1013331/439-311    ...............................................................................................................G...A.GACCACCAAUC...............GUC.G.A.
Ga0157569_1058012/474-586    ..................GU..UUAC.....AACAAUA......UAAUACCCUAGC...GAC..AACU.UAA....................................................................C.CAGG.U.G.
Ga0157562_1164332/672-560    ..................GU..UUAC.....AACAAUA......UAAUACCCUAGC...GAC..AACU..................................................UAACC....................AGG.U.G.
Ga0265746_1084801/20-158     ..............................................................................................................UU...G.AGGAAUGUUAU...............AGC.A.A.
Ga0265752_102479/223-81      ..............................................................................................................UU...G.AGGAAUGUUAU...............AGC.A.A.
Ga0265756_107066/520-660     ..............................................................................................................UU...G.AGGAAUGUUAU...............AGC.A.A.
Ga0247541_102884/37-209      .....................................................................................................UC.CGC.C.CG...U.CUGC.................CAG.AUCC.G.G.
Ga0247516_125012/316-428     ..............................................................................................................AG...G.UGUGGG.................U.UUGA.C.G.
Ga0213850_1071966/331-222    ........................................................................................................UAU.C.U....A.GCUCGACC..................UCG.C.A.
Ga0257141_1100114/291-412    ...................................................................................................................G.UCGAA..................C.GUAC.A.G.
Ga0265740_1020297/298-150    ....AA.....A.....UAU..AUUC.....AUCCAUU......CUC....................................................................G.UGUAU.....................UGC.A.A.
Ga0307482_1064439/425-316    ...............................................................................................................G...A.CCAAGUGG..........UCUUGU.UGAU.A.G.
Ga0138292_1031932/14-100     ..............................................................................................................................................AUUC.A.G.
Ga0138291_1022124/340-254    ..............................................................................................................................................AUUC.A.G.
Ga0184582_127657/238-384     ..............................................................................................U.UGA.GGAGGAG.C.AU...A.GCCAUUGAGCUCCGU.A......C.GAAU.A.G.
Ga0184570_113141/244-341     .................................................................AGA.AAC...........................................G.AGA.......................GAA.G.G.
Ga0310119_152175/242-389     .......................................................................................................................................AUGC.U.AGAU.U.G.
Ga0184576_119948/531-664     ....................................................................................AGACUGUG.CU.UCA.AA....................GGUGUGCGC...........CUCU.G.G.
Ga0316034_129624/151-284     ....................................................................................AGACUGUG.CU.UCA.AA....................GGUGUGCGC...........CUCU.G.G.
Ga0307482_1134687/287-181    .....................................................................................AGGUGUG.GC.C.............................................ACCU.G.G.
Ga0307484_104189/399-292     .........................................................................................GAG.GU.GUG.GCCA.......................................CCU.G.G.
Ga0138282_1242808/325-475    ...AAA.....U.....GCG..AGCA.....ACUGUAC......AAUGCUCAA.........................................................GC...A.GAUG..............ACGCGC.UACA.A.G.
Ga0180047_1081041/176-42     ..........................................................................................UC.CG.CUG.U....GA.U.AU...C.GAGAU.............CGCAGU.UGCC.A.A.
Ga0265752_100023/1618-1523   ..............................................................................................................GG...G.CUAGCAGUCU.............G.CUAU.G.G.
Ga0242649_1051170/34-143     ....................................................................................................CAAGUGG.G.UA...U.AUC.......................AGC.A.G.
Ga0242654_10030584/1049-1243 ..........................................................................GGGAU.CGGGAGUGUUG.......G.GCUUAA..U.UG...G.CGUAGU.............UC....CGUG.U.G.
Ga0307484_102966/727-825     ...................................................................................................................G.GGC......................CUUU.A.G.
Ga0307921_1020571/133-221    ..............................................................................................................UU...G.CAG......................AGUC.G.A.
Ga0184573_113483/125-229     ..............................................................................................................A....C.UCGCUACA..........UUGGCU.AUGC.G.G.
Ga0184576_116834/47-151      .......................................................................................................CU.....CG...C.UACAUUG..............ACU.AUGC.G.G.
Ga0184595_109216/770-611     U..UGC.....C.....AAG..AGUU.....UCCUAUC......CAUGAAC......................................UUG.GA.UCG.GUCAAAA.U.CG...U.GUU....................G.CGUG.U.A.
Ga0316040_154359/245-85      U..UGC.....C.....AAG..AGUU.....UCCUAUC......CAUGAAC......................................UUG.GA.UCG.GUCAAAA.U.CG...U.GUU....................G.CGUG.U.A.
Ga0184595_116949/555-395     ....................................................................................UGAACUUG.GA.UCG.GUCUAAA.U.CG...U.GUU....................G.CGUG.U.A.
Ga0247553_104889/634-563     ............................................................................................................................................U.CAGC.U.U.
Ga0316046_107255/463-562     ...................................................................................................................A.GUGAGC....................GAG.G.G.

                             .........................................................................>>>>>>.>>>>>>>>>>>>.>>.>>>.>>>>>>>.>.>>...>.>>>...............>>>>>>.>>>>.>.>.
                             .........................................................................______.____________.__.___._______._.__...2.222....................2.2222.2.2.
                             -------------------------------------------------------------------------------------------------------------------o-oooo-------------------o-oo●●-●-G-
```

```
Ga0307484_102693/441-627       .......................................................................CGGAC.UACUUAGCCGG............C.UA.C.GA...U.GAUAGCGUUAG............C.GUGG.G.A.
Ga0184568_114257/261-413       .......................................................................GGUCC.CUGUCAUCGACC.CA.GUC.CUCAGA............GGGAG.................CG.CUCA.A.G.
Ga0316047_106478/13-108        ...........................................................................................................AG...A.ACCAAU................AG.UACC.A.G.
Ga0242654_10024002/775-526     A..UAUCAACGA.....AGG..UUUA....AACAUGCAGAUGCGACAUUUAAACC...GCA..UCCC.UAC..........................GGAAAUA.A.GG...A.AGAACC...............GAA.UGUG.A.C.
Ga0316039_101269/650-841       ........................AU....ACGUCGC......CUAGCUUCAGAU...GUC..UACA.GA..................................................................GU.CGCC.G.G.
Ga0265743_102316/615-423       ..................GG..UGGU....UUACUGC......CUAGCUUCAGAU...GUC..UACA.AAG..................................................................U.CGCC.G.G.
Ga0210323_1072445/691-543      ..................................................................................GUGAAGA.GG.UUG......CG.G.CU...A.AACAGCGUUUGC.............GUGG.U.G.
Ga0224712_10069322/1099-1357   A..ACA.....A.....GGU..UCCU....CAGAGCC......UUAGAUGACGAG...CCA..CAGU.AAU.............................GUGG.A.AU...C.GCGUGAGGAUGCCAC.UGCUGUCC.UGAG.A.G.
Ga0213850_1371453/186-298      .......................................................................................................................................GUC.UGUU.U.G.
Ga0153880_1056505/290-430      .......................................................................................GG.GG.GGC.AACACCG...UC...A.UCCACGG................A.UCGU.C.A.
Ga0179940_1033563/296-410      ..................................................................................................UACGCC.C.AG...G.GGUAGC....................UCG.A.G.
Ga0265743_143797/241-325       ................................................................................................................A.CU...................CUC.UGAC.G.G.
Ga0242654_10006748/25-247      ..................................................................................GAAUGAGG.UG.CAC.....GA.U.CG...A.GGCAGUGUUCGCGAG.A....CC..GUCC.G.AA
Ga0138278_1149859/65-166       .......................................................................................................................................GUG.GCAA.C.A.
Ga0138278_1149142/191-91       .......................................................................................................................................GUC.GCAA.C.G.
Ga0138277_1147417/463-362      .......................................................................................................................................GUG.GCAA.C.A.
Ga0138278_1065026/314-214      ........................................................................................................................................UG.GCAA.C.G.
Ga0138277_1089473/408-304      .......................................................................................................................................GUC.GCAA.C.G.
Ga0138277_1022592/399-305      .......................................................................................................................................GUG.GCAA.C.G.
Ga0157559_1099797/360-209      ........................................................................................UAC.GG.UGA.CCCCAG.U.G...A.GGCUC.................C.CGCG.A.G.
Ga0157572_1089974/360-209      ........................................................................................UAC.GG.UGA.CCCCAG.U.G...A.GGCUC.................C.CGCG.A.G.
Ga0242646_1007472/516-317      ...........A.....GAA..AGCC....AACCCUU......GCAAAAGGGAAA...GCC..CACA.ACACAGUGA.AGGUUAUGUUUC.CA.GGU.UAAACAG.A.....G.UAUACAG................C.GCAU.U.G.
Ga0184583_104740/342-477       ..................................................................................GAGCU...AC.GGC.UUCA......AG...G.CGU.....G............UGC.UGUG.A.G.
Ga0242654_11312609/202-83      ....................................................................................................AUAC.C.A........................CUACUC......C.G.
Ga0184568_109070/428-286       ..................................................................................GGGUUG..........UUGGGG.C.UA...C.GAGGAGACAUGA...........U.GCAA.A.G.
Ga0224712_10002011/1564-1454   ................................................................................................................G.GGUA.................UGG.UAUA.A.G.
Ga0222728_1000095/4027-4133    ................................................................................................................G.UGCAAUCCAAGCGGU.A.....GA.GUUA.G.G.
Ga0307482_1068632/529-339      .......................................................................UGGU.G.U........................................................UGU.CUAG.U.G.
Ga0157560_1170105/128-245      .........................................................................................................G.UA...A.GAC....................G.CUGU.A.G.
Ga0316031_107021/319-214       .........................................................................................................................................G.AUGG.U.G.
Ga0316047_100015/734-626       ....................................C.............................................................AAG.............CGGUA.................GG.ACUG.G.A.
Ga0247533_100162/1643-1535     ..................................................................................................................CGGUAG.................G.ACUG.G.A.
Ga0180108_1209503/1932-2029    ................................................................................................................G.ACAAGCGUCUG...........AG.UGUU.A.G.
Ga0265753_1009881/576-819      U..CUA.....A.....AGGCUAGAU....GGCCCCU......AUGGUGUACCGG...ACC..UAGC.UAG.GGUG......AAUCCUAG.UU.GUG.AUCGCAA.A.....G.GAGAG.................GG.UCAU.U.G.
Ga0265741_101522/997-754       U..AAA.....G.....GCU..AGAU....GGCCCCU......AUGGCGUACCGG...ACC..UAGC.UAG.GGUG......AAUCCUAG.UU.GUG.AUCGCAA.A.....G.GAGAG.................GG.UCAU.U.G.
Ga0184583_103998/587-740       ..................................................................................GGGAUCG.GU.GGU....U.AC...GG...C.UUCAAAGCGUG..........UGC.CCGG.U.G.
Ga0247521_125910/353-214       ............................................................................................G.AGG.UGGUCAG.G.C...A.GACUC................GUC.AGUG.A.G.
Ga0184569_106253/218-80        .................................................................G.....AGGUGG.UCAGG.................................CAGA............CUCGUC.AGUG.A.G.
Ga0316039_119781/347-457       ..................................................................................................................GCGGGUAG...............A.UAUA.G.G.
Ga0184572_112077/359-259       ..................................................................................................................GGAUG..................G.UGGU.G.G.
Ga0257141_1118781/153-31       ................................................................................................................A.CCG..................AGC.GUAC.A.G.
Ga0257136_1059838/549-429      ..................................................................................................................ACC..................AGC.AUAC.A.G.
Ga0138283_1149501/173-59       ............................................................................................................A...G.GCGAGUCA..................GGG.G.G.
Ga0184597_126105/545-362       ...............................................AAUCUCAU...GUG..UAAC.CAC...........................GUAC.AA.G.G...A.UUACCCG................G.UACG.G.G.
Ga0213850_1245299/581-409      C..AGU.....G.....CAA..AUGA....UGCACCU......UGUUAAUGGUAC...UAA..GACG.UAC.....................................G...A.GUGA.....................GCUA.C.G.
Ga0179948_1254202/198-296      ............................................................................................................................................GUG.U.G.
Ga0180108_1093556/523-685      .........................................................................................................C.GU...GAUCAAAAGGUUA............U.CUCU.C.G.
Ga0180107_1321405/371-533      ............................................................................................A.CUC.AGCC.CG.U.G...A.UCAAAAGGUUA............U.CUCU.C.G.
Ga0242641_1001197/544-746      ..............................................AAUCUAAAU...UCC..UACC....CGUCCU.UCGUGACAGUCG.UA.UCC.....GG.A......U.CCUAAAGAGCGACCC.U..........CU.G.G.
Ga0184597_115944/155-80        .........................................................................................................................................A.ACCA.G.A.
Ga0184595_100702/414-262       ........................................................................GGGGA.UCGGUGGUUACG.G........CU............UCAAGGCGUG...........UGC.CCGG.U.G.
Ga0184601_118759/683-531       ........................................................................GGGGA.UCGGUGGUUACG.G........CUUC.A.AG...G.CGU.......G..........UGC.CCGG.U.G.
Ga0184592_125105/67-219        ........................................................................GGGGA.UCGGUGGUUACG.G........CUUC.A.AG...G.CGU.......G..........UGC.CCGG.U.G.
Ga0257141_1089020/433-224      C..UUA.....A......AA..AGGU....UUCAAAC......UUGGAUUUAAAC...UAC..UACA.AAC.........................................G.AAGAA..................A.UAAC.A.A.
Ga0265743_103007/262-368       .................................................................................................GUCG.AU.G.UU...G.GCCA......................CCC.G.A.
Ga0222728_1000095/4541-4435    .................................................................UC.AAC...........................GUCGAU.G.UU...G.GCCACC......................C.A.G.
Ga0247537_109082/42-148        ............................................................................................................U...G.GCCA......................CCC.A.G.
Ga0247533_100162/1117-1223     ............................................................................................................U...G.GCCACC......................C.A.G.
Ga0265757_100063/1489-1724     G..CAG.....A......................................................................GGGCUUC.GG.UUG.GUAUCG.....A...A.GCCAGAAAAGCCAAC...C...UC.UUCA.G.G.
Ga0265747_118257/34-269        G..CAG.....A.....GGG..UUUC....GGUUGGU......AUCGAAGCCAGA...AAA..GCCA.............................................A.CCU....................C.UUCA.U.G.
Ga0265745_1000046/1698-1933    ................................CGACC......AAUGUACGUACC...GGC..AGAG.GGC..................A.CC.GAG.UGGUAUC.G.G...G.GCCAGAAAAGCCAACCU......C.UUCA.C.G.
Ga0115595_1153837/320-197      ................................................................................................................C.AGG...................GC.GGCU.A.G.
Ga0242657_1026087/460-322      .......................................................................GGGUA....GUUGUCUUCA.AG.GCG.UGUG...................................C.CCCA.U.G.
Ga0265751_100417/1480-1588     U..UAA.....A...............................................................................................GA...G.GAUAGU...................CGUG.C.G.
Ga0265756_100485/1108-1000     U..UAA.....A...............................................................................................GA...G.GAUAGU...................CGUG.C.G.
Ga0265751_100393/1594-1698     ..................................................................................................................GGUA..................GG.GUGU.A.G.
Ga0307921_1011071/674-791      ....................................................................................AUGUG..................GA...G.ACUAGU.................A.GUCA.A.C.
Ga0307919_1094420/160-285      ..................................................................................UGCGAG....................G...A.GUAAUUGUAUC..........GAG.AUAC.A.C.
Ga0316034_107719/314-205       .....................................................................................................AA....GC...G.GGUAG..................A.UAUG.A.G.
Ga0265742_1065119/361-273      .........................................................................................................................................U.UGUG.C.G.
Ga0180107_1164033/560-431      ..................................................................................GGUCCUCAUU.CAA.GAGUUUG.C.AG...C..CAAG..................C.GCUA.A.G.
Ga0242641_1001148/1456-1256    ......................................................................................CUG.CG.CUU.GU...CC.U.GG...U.UUAC.................CAG.AAGC.G.G.
Ga0307482_1009758/562-669      ................................................................................................................G.GGUA.................UGG.CGUU.G.G.
Ga0247527_109126/592-695       .........................................................................................................................................C.UGGU.U.U.
Ga0247519_111615/148-45        .........................................................................................................................................C.UGGU.U.U.
Ga0247513_116034/391-494       .........................................................................................................................................C.UGGU.U.U.
Ga0213850_1290372/434-275      .......................................................................................................C.U.GG...U.GUC....................G.UGAU.G.A.
Ga0138276_1121602/329-410      .........................................................................................................................................C.UCGC.U.G.
Ga0138278_1041650/183-102      .......................................................................................................................................CAU.UCGC.U.G.
Ga0138277_1137481/570-489      .........................................................................................................................................U.UUGC.U.A.
Ga0138278_1041788/293-378      .........................................................................................................................................U.UUGC.U.G.
Ga0138277_1142657/273-361      .........................................................................................................................................U.UUGC.U.G.
Ga0138275_1223952/371-283      .........................................................................................................................................U.UUGC.U.G.
Ga0213850_1450246/199-116      .........................................................................................................................................A.UCGC.G.G.
Ga0213850_1307525/101-186      ............................................................................................................................................UCG.C.G.
Ga0265756_118952/425-318       ............................................................................................................................................CUU.G.G.
Ga0180107_1244437/408-507      ................................................................................................................A.CAUGA..................G.GUUU.U.G.
Ga0265742_1051722/222-295      ............................................................................................................................................GCC.G.A.
Ga0242657_1051800/416-321      ............................................................................................................A...C.GCA....................G.AUCU.G.G.

                               .......................................................................>>>>>>.>>>>>>>>>>>>.>>.>>>.>>>>>>>.>.>>...>.>>>..............>>>>>>.>>>>.>.>.
                               ................................................................................................................2.222..........................2.2222.2.2.
                               ----------------------------------------------------------------------------------------------------------------o-oooo----------------------o-ooo••-•-G-
```

```
Ga0307484_102966/353-250     ...............................................................................................................G.GCGGGUGGCUA...............CAU.G.G.
Ga0184568_108419/50-147      .....................................................................UGGU.............................................................GGC.ACUA.A.G.
Ga0184568_103677/484-581     ...........................................................................................................G...A.AGU..................GGC.ACUA.G.A.
Ga0307484_102709/562-403     ...............................CAUGC.....AAGGCUUUCGCU...AAA..UGAC.GAC...................................................................C.UCUU.G.G.
Ga0127503_10678853/419-333   ........................................................................................................................................G.AGUC.A.G.
Ga0307919_1065410/76-162     ...............................................................................................................C.GAGAG......................UC.A.G.
Ga0242652_1038498/365-470    ..........................................................................................................CC...A.AAG....................A.CCAC.A.G.
Ga0307484_101264/723-941     .....A....A.....UGA..UGGU....ACCUACC.....GUCUCGACGACA...UCC..U......................GUUG.UC.AGA.CGACCGU.C.UC...G.CUCAAAGAGUGUG..........C.UCAA.G.G.
Ga0247534_101560/228-122     .................................................................................................................AGCGGUGA.............UGG.UCAG.G.G.
Ga0307484_109931/110-215     ...........................................................................................................A...A.UUUCGUGUUGGCCAC............UG.C.G.
Ga0184595_116681/414-266     .....................................................................................GCG.AC.UUG.AU...AC...GA...C.UUCGAGGCAUG..........UGC.UCUG.A.G.
Ga0265749_100155/962-1187    .....................................................................................GAG.GU.UCU.CCAGCU.........U.CAAGACGUAUGCC.............UUG.U.G.
Ga0310115_109341/244-400     G..AUC....A.....GAA..CUGC....CCCGAUU.....ACUCGG...........................................A.GGU.UCUCCAG.C.UU...C.AAAGACGUAUGCC.............UUG.U.G.
Ga0310111_106996/679-905     C..GUA....A......GU..CUCG....AGGGCGC.....ACGCUAUCGUCC...GAU..UACU....CGGAGG.UUC..................U.CCAG.C.UU...C.AAAGACGUAUGCC.............UUG.U.G.
Ga0265756_101315/275-501     ..........A......GU..CUCA....AGGGCGC.....ACGCUAUCGUCC...GAU..UACU....CGGAGG.UUC...UCCAGC.UU.CAA.AGACGUA........U.GCC.......................UUG.U.G.
Ga0265751_100874/820-594     C..GUA....A......GU..CUCG....AGGGCGC.....ACGCUAUCGUCC...GAU..UACU....CGGAGG.UUC...UCCAGC.UU.CAA.AGACGUA........U.GCC.......................UUG.U.G.
Ga0265744_120119/40-266      C..GUA....A......GU..CUCA....AGGGCGC.....ACGCUAUCGUCC...GAU..UACU....CGGAGG.UUC...UCCAGC.UU.CAA.AGACGUA........U.GCC.......................UUG.U.G.
Ga0310102_105010/226-452     C..GUA....A......GU..CUCA....AGGGCGC.....ACGCUAUCGUCC...GAU..UACU....CGGAGG.UUC...UCCAGC.UU.CAA.AGACGUA........U.GCC.......................UUG.U.G.
Ga0247534_103639/475-250     C..GUA....A......GU..CUCA....AGGGCGC.....ACGCUAUCGUCC...GAU..UACU....CGGAGG.UUC...UCCAGC.UU.CAA.AGACGU...................AUGC.............CUUG.U.G.
Ga0265741_101833/434-208     C..GUA....A......GU..CUCA....AGGGCGC.....ACGCUAUCGUCC...GAU..UACU....CGGAGG.UUC...UCCAGC.UU.CA....CAGAC.G.UA...U.GCC.......................UUG.U.G.
Ga0242647_1002114/801-1026   C..GUA....A......GU..CUCA....AGGGCGC.....ACGCUAUCGUCC...GAU..UACU....CGGAGG.UUC.......UC.CA.GCU.UCAAGAC.G.UA...U.GCC.......................UUG.U.G.
Ga0265752_104585/383-158     C..GUA....A......GU..CUCG....AGGGCGC.....ACGCUAUCGUCC...GAU..UACU....CGGAGG.UUC.......UC.CA.GCU.UCAAGAC.G.UA...U.GCC.......................UUG.U.G.
Ga0310107_109330/1076-850    C..GUA....A......GU..CUCG....AGGGCGC.....ACGCUAUCGUCC...GAU..UACU....CGGAGG.UUC...UCCAGC.UU.CAA....................AGACGUAUGCC.............UUG.U.G.
Ga0115595_1051440/308-419    ..........................................................................................................UA...C.GUC....................G.UAAA.A.G.
Ga0115594_1054328/389-276    ..........................................................................................................UA...C.GUC....................G.UAAA.A.G.
Ga0307482_1048753/383-528    ...........................................................................................................A...U.GCUC......................UGG.U.G.
Ga0307480_1006257/684-599    ...........................................................................................................G...G......................GCC.CGGG.C.G.
Ga0184595_116802/212-325     ...............................................................................................................A.UAAAG..................A.CGGU.C.G.
Ga0307919_1071894/312-31     U..CAC....U.....AGG..AUCG....AAAGAUC.....GUGAGUUAAUCA...CAU..AACAGUACUCGCGC.UUAGCCUAAGCU.GA.AGC.GGUACAG.U.GU...G.UGAAAUCU...............G.AAUG.U.G.
Ga0307921_1036773/354-73     U..CAC....U.....AGG..AUCG....AAAGAUC.....GUGAGUUAAUCA...CAU..GACAGUACUCGCGC.UUAGCCUAAGCU.GA.AGC.GGUACAG.U.GU...G.UGAAAUCU...............G.AAUG.U.G.
Ga0307482_1023665/889-1191   .....................................................................GGGCCGGAGGCCGCCGCAA.GCUCUG.CACGAGC.A.AC...C.CUCGGUAUGUGCUGG.U.CUG..G.GAGG.G.G.
Ga0180108_1022680/3902-3998  ...............................................................................................................A.CCG...................AU.CUUU.A.G.
Ga0184599_137402/105-300     .................GA..ACGC....CAAUCUC.....CAUGACAUGC......................................AC.CUC.GUU.UAG...AG...A.UGUCU...UA........G..CAU.UGAU.A.G.
Ga0157569_1080558/455-605    ............................................GAUUCAAUU...UCA..AUCA.AAC.....................................AA...A.UCGAAC...................GCGC.G.G.
Ga0184601_113231/247-149     ..........................................................................................................CC...C.CCCAAUGA...............U.GCAU.G.G.
Ga0242652_1002960/413-574    .................AG..GUGC....UUCCAUG.....UACCAACCUAUC...CAC..UAC......................................................................CGA.AGUA.G.G.
Ga0307484_101303/704-866     .....................................................................................A.G.UG.CUU.AUCCU.....UC...C.ACUAC................CGA.AGUA.G.G.
Ga0242641_1025149/251-89     .....................................................................................AGU.AC.C.C.AUCCU.....UC...C.ACUAC................CGA.AGUA.G.G.
Ga0307482_1023050/594-496    .................................................................................................................GUCC...................A.UGUC.A.G.
Ga0307484_100434/1330-1210   G..AAA....A............................................................................................................................GG.UCAU.C.G.
Ga0153880_1432604/289-395    ..........................................................................................................GG...C.GUGUAGGG...............C..UCC.A.A.
Ga0157559_1128929/201-87     ........................................................................................................................................A.UAGG.U.G.
Ga0157560_1113202/155-269    ........................................................................................................................................A.UAGG.U.G.
Ga0157566_1012736/497-384    ........................................................................................................................................A.UAGG.A.G.
Ga0157579_1155702/323-436    ........................................................................................................................................A.UAGG.A.G.
Ga0157562_1157806/189-74     ........................................................................................................................................A.UAGG.A.G.
Ga0180047_1032966/152-37     ........................................................................................................................................A.UAGG.A.G.
Ga0307484_166952/62-163      ...............................................................................................................A.CCUA...................A.UAGU.A.A.
Ga0005851_1178990/477-379    .................................................................................................AUGAAU.C.UA...A.UCGCAA......................A.A.G.
Ga0153880_1191383/455-340    .................GG..UUG...................................................................................U...U.ACCAAUGAA..............G.AAUU.A.G.
Ga0213850_1282564/267-350    ...........................................................................................................G...G.GUCAG......................GU.G.G.
Ga0315817_133437/498-413     ..........................................................................................................AG...G.GUCAG......................GU.G.G.
Ga0315826_123252/262-347     ...........................................................................................................G...G.GUCAG......................GU.G.G.
Ga0242657_1008904/1267-1104  ...............................................................................................................U.GGCAAAG...............UC.CUGA.A.G.
Ga0222728_1009902/1182-968   ......................CAC....GACAAUG.....GUUUAAUGGAAC...AAC..CACG.AUC.............................................GGUC.............CAACAC.UUGU.A.G.
Ga0115594_1106220/441-334    ..........................................................................................................UC...G.UGU.......................CCU.A.G.
Ga0180107_1307934/226-315    ..................................................................................................................AGUCAG...................UCC.C.G.
Ga0184577_115871/202-369     .....................................................................UUGUCC.UUGCGAACGAUG.C....G.UCCAUCG.G.CU...U.CAUGGCGUGUG............C.UCUU.A.G.
Ga0206356_11590518/224-68    ..........................................................................................................UA...C.ACGAGC.................A.CCGC.G.C.
Ga0222728_1062510/166-66     .................................................................................................ACUCAA.A.UA...A.UAGUCU....................GUG.A.A.
Ga0316029_101202/440-551     ...........................................................................................................G...A.CCAAGC.................U.CCCA.G.A.
Ga0307482_1017422/1387-1206  .................AU..ACUC....UGUGUGC.....AUCCUGCUCAAU...CAC..AACU.G.............................AUUUACG.A.GU...A.GCU...............UACCGA.GCUA.G.G.
Ga0307484_100889/412-594     .................AU..ACUC....UGUGUGC.....GUCCUGCUCAAU...CAC..AAC.................UGAUUUA.CG.A........GU.A.GC...U.UAC..................CGA.GCUA.G.G.
Ga0255062_11230321/200-110   ...............................................................................................................U.CUCGA.....................CCA.C.A.
Ga0255059_10691632/166-76    ...............................................................................................................U.CUCAA.....................CCA.C.A.
Ga0265744_100073/1235-1401   ......................................................................................GU.CC.C.C.CGAUUUC.C.GC...C.AGUGCUCCACUCCGC......CGU.AGCA.G.A.
Ga0265757_113703/332-416     .............................................................................................................................................G.C.G.
Ga0265756_158829/72-188      ..........................................................................................................AC...G.AAA....................U.AAUC.C.G.
Ga0184597_100195/107-221     ...................................................................................................CCUG........G.AGUAG.......................U.G.G.
Ga0184594_121503/721-607     ...................................................................................................CCUG........G.AGUAG.......................U.G.G.
Ga0184582_121032/1005-1119   ...................................................................................................CCUG........G.AGUAG.......................U.G.G.
Ga0184602_104873/671-785     ...................................................................................................CCUG........G.AGUAG.......................U.G.G.
Ga0184589_132372/822-708     ...................................................................................................CCUG........G.AGUAG.......................U.G.G.
Ga0184580_125558/64-178      ...................................................................................................CCUG........G.AGUAG.......................U.G.G.
Ga0184596_101726/395-281     ...................................................................................................CCUG........G.AGUAG.......................U.G.G.
Ga0247541_109256/178-292     ...................................................................................................CCUG........G.AGUAG.......................U.G.G.
Ga0184584_108444/253-366     ...............................................................................................................C.CUG..................GAG.UCAU.G.G.
Ga0184582_124913/742-629     ...............................................................................................................C.CUG..................GAG.UCAU.G.G.
Ga0184600_135218/318-205     ...............................................................................................................C.CUG..................GAG.UCAU.G.G.
Ga0247541_100074/611-498     .................................................................................................UAGGGU.U......G.CCUA.................GAG.UCAU.G.G.
Ga0247551_101122/731-844     .................................................................................................UAGGGU.U......G.CCUA.................GAG.UCAU.G.G.
Ga0247526_101949/462-575     .................................................................................................UAGGGU.U......G.CCUA.................GAG.UCAU.G.G.
Ga0247524_101842/357-244     .................................................................................................UAGGGU.U......G.CCUG.................GAG.UCAU.G.G.
Ga0247519_108765/580-693     .......................................................................................A.UC.CUA.GGGUU...G.CC...U.GGA....................G.UCAU.G.G.
Ga0180107_1134582/382-542    .............................................................................................CA.GACCUUC.A.UU...G.GAAAGUC...........CUAGGC...UC.A.G.
Ga0265747_125301/405-306     ..................................................................AAC...................................................................A.GAAG.A.G.
Ga0157593_1101573/244-147    ........................................................................................................................................C.UCAU.G.G.
Ga0184599_121568/40-155      ........................................................................................................................................C.UCAU.A.G.
Ga0316040_116783/379-494     ........................................................................................................................................C.UCAU.A.G.

                             .....................................................................>>>>>>.>>>>>>>>>>>>.>>.>>>.>>>>>>>.>.>>...>.>>>...............>>>>>>.>>>>.>.>.
                             ...............................................................................................................2.222....................2.2222.2.2.
                             ---------------------------------------------------------------------------------------------------------------o-oooo-------------------o-o●●●-●-G-
```

```
Ga0157560_1091679/440-538      .......................................................................................................................................C.UCAU.G.G.
Ga0222728_1165579/392-259      ......................................................................................GAA.A...........AC.U.AG...U.AUGAGG.............CG..GUCG.A.A.
Ga0224712_10601975/393-502     .....................................................................................................................................GAC.CUUA.G.G.
Ga0224712_10010317/1973-2082   ...........................................................................................................GC...G.AGUA...............GAC.CUUA.G.G.
Ga0307482_1048660/422-625      A..CCU.....A................................................C..CAU..........................................G...A.CUCAAAGGGUGUG........C.CAAC.G.G.
Ga0179934_1155244/114-26       ..................................................................................................................GC..........................C.G.
Ga0307484_101172/602-495       ............................................................................................................C...C.CUUAG................U.GAGU.C.G.
Ga0242654_10068518/113-218     ...................................................................................................GCCCUU.A.G...U........................GAGU.C.G.
Ga0242647_1002406/915-1021     ...................................................................................................GCCCUU.A.G...U........................GAGU.C.G.
Ga0222729_1010500/11-117       ...................................................................................................GCCCUU.A.G...U........................GAGU.C.G.
Ga0307482_1202605/45-154       ................................................................................................................A.GGU.............GACGGU.CAUA.U.G.
Ga0184603_139562/141-280       .........................................................................................................................................CCAA.G.G.
Ga0242646_1001122/826-995      .............................................................................CUGCUACAUGUC.UC.UGG.AUCC.UU.C.UC...A.GCCGC..............CGU.GGCG.U.G.
Ga0310107_144222/327-436       ............................................................................................................G...U.GGUGAA...............G.UAAU.G.G.
Ga0265751_124807/392-283       ............................................................................................................G...U.GGUGAA...............G.UAAU.G.G.
Ga0184572_105168/388-281       ................................................................................................................G.GGUA................GA.AUGG.A.G.
Ga0316036_127178/124-231       ................................................................................................................G.GGUA................GA.AUGG.A.G.
Ga0222728_1003482/1262-1107    ................................................AUUGUGU...AAC..AACU.AAC....................A.GGG.A....AC.C.CU...A.CGCCUA.............C.G.GCGC.A.G.
Ga0265743_100639/33-191        ............................................................................................................U...U.GGGUGUGC.............C.UGCU.G.G.
Ga0307484_105374/518-723       .................................................................................UAGACACC.GA.CUC.AUCCCUU...AA...A.CCUAG...............GC.UAAA.G.G.
Ga0265742_1015608/410-491      .......................................................................................................................................G.AGGG.G.G.
Ga0316030_102139/629-482       ............................................................................................................G...U.AGC................GUA..UUC.U.G.
Ga0265749_106268/131-212       ..........................................................................................................................................GUU.G.G.
Ga0184573_100752/140-46        .....................................................................................................................................UGA.UCGC.A.G.
Ga0157560_1010344/357-456      .......................................................................................................................................A.AGCU.A.G.
Ga0157593_1006201/239-338      .......................................................................................................................................A.AGCU.A.G.
Ga0180047_1087048/281-180      .......................................................................................................................................A.AGCU.A.G.
Ga0157565_1016893/233-334      .......................................................................................................................................A.AGCU.A.G.
Ga0184568_100511/100-195       .......................................................................................................................................C.UCAG.C.G.
Ga0265759_114856/370-262       ..........................................................................................................................................CUU.A.G.
Ga0222729_1080556/362-213      ...........................................................................................................GU...A.GGUAAC....................U.A.G.
Ga0247541_102707/328-184       ..................................................................................GAGGGGG.A.......................ACGGCACC...........UCU.UCGA.A.G.
Ga0307921_1020694/406-483      .......................................................................................................................................G.UCUU.A.G.
Ga0307919_1034281/527-451      .......................................................................................................................................G.UCUU.A.G.
Ga0184568_108869/934-1072      ......................................................................................UCA.GC.CUU.AGUCUAA.A.GG...A.GUGAC................C.AACU.U.G.
Ga0206356_10036221/150-306     ............................................................................................................G...A.GACCU...............GG.UCUU.A.U.
Ga0247554_104968/233-375       ..................................................................................................................UACA............UCGUUA.UUUA.A.C.
Ga0265753_1030448/124-358      G..AGG.....A.....GAU...............AG.....GAUUAUCCCCCU...UCC..CAUA.AGCUCACAG.UUGUAGGACUAA.AC.CUA.AA.........G...G.AAUGAC...............C.CUAG.G.G.
Ga0032354_1086862/128-19       ...........................................................................................................UG...U.CAGACC..........UUCCG...AUU.A.G.
Ga0213850_1119088/23-236       .................................................UAUAAU...UAC..AACC.AGC..........AAAGGUUG......G.UCACUCG.UUUG...A.CGC................AUG.UGUA.A.G.
Ga0222728_1000079/1837-1731    ..................................................................................................UCGAUG...UU...G.GCCA....................CCC.A.A.
Ga0247535_100068/719-828       ..................................................................................................CCCAAG.C.GG...G.UAG..................A.AUGG.A.G.
Ga0265744_100861/398-567       .................................................................................UAUCCCUC.GA.GCU.CUAUUAC.U.CG...A.CCCUAC.............CGG.GUUG.G.A.
Ga0316038_100042/1676-1569     ............................................................................................................C...G.GGUAGA.................GCUC.A.GU
Ga0184578_101068/480-310       .................................................................................UGUGUUUG.UC.CUU.AGCCUCG.G.AU...C.UCCAG..............CUU.GGCU.G.A.
Ga0265759_100473/818-988       .................................................................................UGUGUUUG.UC.CUU.AGCCUCG.G.AU...C.UCCAG..............CUU.GGCU.G.A.
Ga0265756_100592/1458-1288     .................................................................................UGUGUUUG.UC.CUU.AGCCUCA.G.AU...C.UCCAG..............CUU.GGCU.G.A.
Ga0265746_1003835/1049-1219    .................................................................................UGUGUUUG.UC.CUU.AGCCUCA.G.AU...C.UCCAG..............CUU.GGCU.G.A.
Ga0247536_100773/637-807       .................................................................................UGUGUUUG.UC.CGU.AGCCUCA.G.AU...C.UCCAG..............CUU.GGCU.G.A.
Ga0184573_112336/323-153       .................................................................................UGUGUUUG.UC.CUU.AGCCUCA.G.AU...C.UCCAG..............CUU.GGCU.G.A.
Ga0247530_109035/489-319       .................................................................................UGUGUUUG.UC.CUU.AGCCUCA.G.AU...C.UCCAG..............CUU.GGCU.G.A.
Ga0247513_128543/241-71        .................................................................................UGUGUUUG.UC.CUU.AGCCUCA.G.AU...C.UCCAG..............CUU.GGCU.G.A.
Ga0247541_101130/558-391       ......................................................................CGUAG.......GUCUAUG.UG.UCU.GAUCGAU.C.GA...C.UCCAA..............CCA.GGUU.G.A.
Ga0213850_1278242/600-506      ................................................................................................................G.GGCGG....................CC.G.A.
Ga0213850_1524759/286-380      ................................................................................................................G.GGCGG....................CC.G.A.
Ga0180107_1045142/740-541      ......................................................................CAGGGU.CUCCUGGAUUCA.CA.ACG.GGCCA.....GC...A.GCGCAAGGCAUGCGC.C...GG.AGUG.A.A.
Ga0307484_102002/698-552       ..................................................................................................CUUUUC.C.GG...C.UUCGAGGCGUGU........GC.UUGU.A.G.
Ga0265759_115288/4-102         ..................................................................................................................CU...................A.CCUG.U.G.
Ga0316029_136733/88-8          ..................................................................................................................GCCGA.......................G.G.
Ga0242646_1008525/229-119      ...........................................................................................................................................AG.C.G.
Ga0115594_1180137/311-161      ......................................................................................GAA.GC.GUG.CGCCCCG.G.GC...C.CCCCUC...............C.UCGC.G.G.
Ga0179947_1156513/270-495      U..UAU.....A.....UAG..ACAC.....AAUGCUU......GAC..................................AUAGUUGA.GA.UUG.AUCCUC....UA...C.AUUAGAG...................G.U.G.
Ga0179948_1263234/366-292      .......................................................................................................................................A.GAGG.U.G.
Ga0179947_1148619/378-303      .......................................................................................................................................A.GAGG.U.G.
Ga0257141_1138773/402-200      ......................................................................UGGU.G.UCAUUGUUGGG............GGAG.A.GU...G.UGU................CGA.UGCU.A.G.
Ga0242654_10401151/84-171      ................................................................................................................G.GAGGA.......................A.G.
Ga0184603_114388/264-401       ......................................................................................GCG.AC.CG..........A.UG...A.GAGGAU..........UCAAAC.AAAA.A.G.
Ga0316052_118887/313-177       ......................................................................................GCG.AC.CGA...........UG...A.GAGGAU..........UCAAAC.GAAA.C.G.
Ga0079039_1671802/197-34       ........................................................................................U.CU.GU.....ACUC.C.GA...G.GACACUC............CCA.GGAA.A.G.
Ga0247542_100007/609-500       ...................................................................................................CCAAG.U.GG...G.UGAUGG...............G.GUGU.A.G.
Ga0247552_100007/4311-4420     ...................................................................................................CCAAG.U.GG...G.UGACGG...............G.GUGU.A.G.
Ga0184586_103737/25-134        ...................................................................................................CCAAG.U.GG...G.UGAUGG...............G.GUGU.A.G.
Ga0184594_103311/207-316       ...................................................................................................CCAAG.U.GG...G.UGAUGG...............G.GUGU.A.G.
Ga0265756_101275/650-541       ...................................................................................................CCAAG.U.GG...G.UGAUGG...............G.GUGU.A.G.
Ga0247541_100010/3268-3377     ...................................................................................................CCAAG.U.GG...G.UGAUGG...............G.GUGU.A.G.
Ga0184599_154003/184-293       .....................................................................................................AAG.U.GG...G.UGAUGG...............G.GUAU.A.G.
Ga0184580_121439/573-464       ...................................................................................................CCAAG.U.GG...G.UGAUGG...............G.GUGU.A.G.
Ga0184582_108821/1050-1159     ...................................................................................................CCAAG.U.GG...G.UGAUGG...............G.GUGU.A.G.
Ga0184575_101094/586-477       ...................................................................................................CCAAG.U.GG...G.UGAUGG...............G.GUGU.A.G.
Ga0184598_129111/512-403       ...................................................................................................CCAAG.U.GG...G.UGAUGG...............G.GUGU.G.G.
Ga0184596_107732/313-205       ...................................................................................................CCAAG.U.GG...G.UGAUGG...............G.GUGU.A.G.
Ga0247554_100006/1592-1485     ...................................................................................................CCAAG.U.GG...G.UGAUGG...............G.GUGU.A.G.
Ga0247534_100047/1899-2008     ...................................................................................................CCAAG.U.GG...G.UGAUGG...............G.GUGU.A.G.
Ga0184597_128933/573-464       ...................................................................................................CCAAG.U.GG...G.UGAUGG...............G.GUGU.A.G.
Ga0213850_1302171/343-113      G..UAU.....A........................................................................................AAAG.U.UU...U.CAGAUUGAAUGAA......CCC.CUGG.G.G.
Ga0213850_1513954/279-196      .......................................................................................................................................A.UCCC.G.GA
Ga0153880_1231581/116-241      ..............................................................................................................................................G.G.
Ga0247541_104364/333-188       ....................................................................................................UCAG.A.UA...A.UGA..............GGUCA.ACUA.A.G.
Ga0138277_1028338/433-516      ............................................................................................................U...A.UAGCAAG...................U.A.G.

                               ......................................................................>>>>>>.>>>>>>>>>>>>.>>.>>>.>>>>>>>.>.>>...>.>>>.............>>>>>>.>>>>.>.>.
                               ................................................................................................................2.222..................2.2222.2.2.
                               ----------------------------------------------------------------------------------------------------------------○-○○○○-----------------○-○○●●-●-G-
```

```
Ga0265752_101001/521-687       C..CAU.....A......................................................................................ACCUGG.C.UU...A.AGGAGUG..............UGC.UCAA.G.C.
Ga0307482_1449537/408-223      ...................................................................................U..CGC.AC.GAG.UAACCGACA.AG...A.UCC...............CCUGCU.UGCA.G.G.
Ga0257136_1044348/475-684      A..AAA.....A.....GGU..UUCA.....AACUUGC......AUUUAAACUACU...ACA..AAC........................................GA...A.GAA....................A.UAAC.A.A.
Ga0257138_1055324/266-476      A..AAA.....A.....AGG..UUUC.....AAACUUG......CAU...UUAAAC...UAC..UACA.AAC...................................GA...A.GAA....................A.UAAC.A.A.
Ga0257140_1130477/77-287       A..AAA.....A.....AGG..UUUC.....AAACUUG......CAUUUAAACUAC...UAC..AAAC.......................................GA...A.GAA....................A.UAAC.A.A.
Ga0127502_10711632/191-294     ..................................................................................................................CCCGAU............C....G.UAUC.U.G.
Ga0184568_105547/129-239       .................................................................................................AUAG.AA.U.GA...A.GUUGAUGCCUG...............UAA.G.G.
Ga0206353_11976089/292-380     .......................................................................................................................................CGU.U..G.C.G.
Ga0184577_108791/182-295       .............................................................................................................U..G.AGG...................GA.GCGU.C.G.
Ga0242649_1050441/340-170      C..UUG.....A......................................................................................................CCAAA..................U.CGUC.G.G.
Ga0179934_1197833/207-115      ....................................................................................................GAUG.U.UG...U.CAA.......................CUG.U.G.
Ga0180107_1174136/218-102      ......................................................................................ACG.UG.CUC......AU.A.AC...U.GGU...................UC.UCGU.A.G.
Ga0224712_10002011/1106-1211   ...........................................................................................................GG...G.AGAAGG.C...........C.....ACCC.A.G.
Ga0224712_10010317/2429-2325   ...........................................................................................................GA...G.AGGAG.GC...............C.ACCC.A.G.
Ga0206355_1311803/144-334      ..................................................................................................CCUGCC.C.AG...U.UGU..................GGA.UUAG.A.C.
Ga0307482_1417159/374-296      .........................................................................................................................................U.GAUA.U.G.
Ga0213850_1396191/936-678      G..AAC.....C.....GAG..CGUA.....GACAGGU......GUGUUAAACACC...ACAGGUACC.AAG...........AG.AGA.UG.CCU.G....AG.A.CA...G.ACU...............UGCCCC.UGGG.G.G.
Ga0222728_1287830/12-211       ............................................................G..AAAG.ACC....................................GA...A.GACCUGCUCGGCAUC.C.........UGG.A.G.
Ga0242652_1169169/12-211       ............................................................G..AAAG.ACC....................................GA...A.GACCUGCUCGGCAUC.C.........UGG.A.G.
Ga0242654_11312699/30-229      ..................AG..UCGC.....CCCAACU......CUGGAUGAUUAU...UCA..GACU.AAA.AGUCG.UCUAAUCUGAAA.GA.CCG..AAGG.C.CU...G.UUCGGCAU..CC..............UGG.A.G.
Ga0265759_104327/339-243       ............................................................................................................................................GGG.A.G.
Ga0265756_100717/910-1050      A..C................................................................................................UGUC.A.GG...G.ACU..................AUC.GCGU.U.G.
Ga0265757_113513/277-362       .........................................................................................................................................G.UGGG.U.A.
Ga0242654_10024002/836-915     ...........................................................................................................................................CCUU.G.A.
Ga0184573_101763/767-477       C..UGA.....G.....CAC..AGGA.....GGAGAUG......GUAGUAUCCCCC...UGC..CAA..............UGUUCGAG.AG.UUC.CCAUGCC.U.AG...U.CUAAAGGAGUGACCC.A..........CA.G.G.
Ga0242654_10701525/53-296      ..................GU..GGUG.....GUACUUG......CCUGACUGGAAA...ACC..AACC.AGU...............UG......G.GU...AA.C.AA...C.ACCCUGC..............CGA.AGCA.G.G.
Ga0247529_117158/253-331       .............................................................................................................................................CU.G.G.
Ga0326631_117973/181-293       ...........................................................................................................UG...C.GAA.........................A.A.G.
Ga0316038_107116/33-174        ................................................................................................................C.UCAA..............GUAUAG.AUAU.A.G.
Ga0316037_108094/19-160        ................................................................................................................C.UCAA..............GUAUAG.AUAU.A.G.
Ga0247527_116942/161-88        ............................................................................................................................................UGU.C.G.
Ga0138278_1022898/219-127      ................................................................................................................G.ACCA........................G.A.G.
Ga0127503_11028958/51-130      .......................................................................................................................................UGA.UAAA.A.U.
Ga0307482_1623775/241-347      ................................................................................................................G.ACAUGGGUUAGCGAC.C....UGU.UUCU.U.G.
Ga0307484_111921/444-550       ................................................................................................................G.GCAGAGGUCAGCGAC.C....UGU.UUCU.U.G.
Ga0307484_101582/1018-1124     ................................................................................................................G.ACAGAGGUCAGCGAC.C....UGU.UUCU.U.G.
Ga0265742_1021317/385-459      .........................................................................................................................................U.GUCC.A.G.
Ga0157569_1089063/252-138      .........................................................................................................................................A.UAGG.U.G.
Ga0153880_1457361/236-121      .........................................................................................................................................U.AAGG.A.G.
Ga0256352_1087084/23-118       ...........................................................................................................................................UAGG.A.G.
Ga0307482_1000325/24-131       ......................................................AC...AUA..UACC.AAG...................................UG...G.UGAA...................G.AUAG.A.G.
Ga0157560_1147189/148-60       ...........................................................................................................UU...G.CCG.......................UCU.U.G.
Ga0247519_105128/410-263       ....................................................................................................................................CUGCU..CCAA.A.G.
Ga0184573_113193/903-756       ....................................................................................................................................CUGCU..CCAA.A.G.
Ga0157593_1013623/299-215      ....................................................................................................................................CU.....UCAA.G.G.
Ga0184593_126886/349-213       ..................................................................................................UGUCUG.U.AA...A.GA...................CGU.G.GU.G.G.
Ga0184568_117306/561-697       ..................................................................................................UGUCUG.U.AA...A.GA...................CG..UGGU.G.G.
Ga0184597_120210/233-147       ................................................................................................................G.UGCAG......................GC.G.G.
Ga0184570_100371/69-155        ................................................................................................................G.UGCAG......................GC.G.G.
Ga0157562_1160399/232-315      .......................................................................................................................................AAU.CUAU.G.G.
Ga0265744_114151/132-24        ................................................................................................................C.CAGCA.................UU.CUAA.U.G.
Ga0265743_119695/104-184       .........................................................................................................................................G.UGUU.C.G.
Ga0265743_101198/448-619       .................................................................................UUCCCCCU.AG.GCU.CUAUUAC.C.UG...G.CCC...............UACCGG.GCUG.G.A.
Ga0316046_121611/330-214       ............................................................................................................................................CUG.U.G.
Ga0265757_120140/89-204        .............................................................................................................................................UG.U.G.
Ga0138279_1083071/419-326      .......................................................................UG...........................CGGG.A.....U.CGGUG.....................CUA.C.G.
Ga0310106_100998/1608-1690     .........................................................................................................................................C.UUCU.G.G.
Ga0265756_102054/286-395       ...................................................................................................UUCGA.A.GU...C.GAUA..................GG.AUCU.G.G.
Ga0180108_1209503/2438-2343    ................................................................................................................C.CCU....................A.GUCA.C.G.
Ga0180107_1057591/210-107      .......................................................................................................................................UGU.AUAU.U.G.
Ga0307482_1074296/478-354      ...................A..UUCA.....UUUAUGA......GUGAACAGAAAC...AAA..AACA.CAA.................................................................U.ACUG.C.A.
Ga0180108_1022680/4248-4153    ................................................................................................................C.UCU...................UG.GUUA.U.G.
Ga0247519_103291/1381-1460     ...........................................................................................................GU...C..AC........................UC.C.U.
Ga0184590_126350/300-380       ................................................................................................................G.UCA.......................CUC.C.U.
Ga0247551_100996/477-556       ................................................................................................................G.UCA.......................CUC.C.U.
Ga0316035_144545/195-301       .........................................................................................................................................G.UCAA.U.G.
Ga0316049_154968/227-319       ...........................................................................................................AU...C.CAACGUC................G.UAUU.A.G.
Ga0184593_115334/333-240       ......................................................................................................AU.C.CG...A.UGUCGU....................AUU.G.G.
Ga0307921_1005950/551-460      ...........................................................................................................UA...A.UGC........................CC.U.G.
Ga0242647_1014694/423-336      ..................................................................................................................UGCAA..................C.CAAC.U.G.
Ga0184580_102899/442-582       ................................................................................................................A.GAUCGCU...........CU.UCC.CUCA.C.G.
Ga0265743_108998/150-253       ..................AG..AGCA.....AUCCAAG......C......................................................................GGUA.................GG.AUGU.G.G.
Ga0316050_103193/566-460       ........................................................................................................................................GG.AUGA.G.G.
Ga0316030_100208/805-700       ................................................................................................................G.ACUA..................GG.AUGA.U.G.
Ga0307482_1000262/4800-4699    .......................................................................................................C.U.GU...G.UUCGC..................G.UAUG.C.G.
Ga0307482_1018929/1182-1081    .......................................................................................................C.U.GU...G.UUCGC..................G.UAUG.C.G.
Ga0153880_1260556/441-589      .......................................................................................................G.U.GU...A.GUGUAU....A..............AGCU.U.G.
Ga0180044_1087599/197-316      ............................................................................................................G...A.CUCAAC...............CGG.CAAG.G.G.
Ga0127502_11371108/362-252     ................................................................................................................G.GGUA..................GG.AUGU.A.G.
Ga0184588_106948/865-640       ..................................................................................................................GGGCA.............AUCC.U.CCAG.G.G.
Ga0247533_100438/1040-888      ............................................................................................................G...U.GUUAG.................GA..GCC.G.G.
Ga0127502_10189886/163-55      .........................................................................................................................................G.AAAA.C.G.
Ga0079074_1318576/412-571      .......................................................................UGGUUG...................C.C..CAGC.G.....C.CCC..................CUC.CGAA.G.G.
Ga0115594_1212468/8-118        ................................................................................................................A.GCA..................CGA.ACAC.A.G.
Ga0115594_1134035/652-542      ................................................................................................................A.GCA..................CGA.ACAC.A.G.
Ga0316030_105840/623-504       .......................................................................................UC.AA.AUG.UCUACAA.A.CC...C.CCCAA.......................A.G.G.
Ga0316033_110175/528-409       ..................................CAUU......GACGUGUCAAAU...GUC..UA...................................CAA.A.CC...C.CCCAA.......................A.G.G.
Ga0228708_1128185/228-344      .....................................................................................................CAU.C.CC...A.UUG....................A.GCGU.G.A.
Ga0126318_10199518/109-204     ............................................................................................................G...A.UGGAGUA...............UG.GUGC.G.C.

                               ......................................................................>>>>>>.>>>>>>>>>>>>.>>.>>>.>>>>>>>.>.>>...>.>>>...............>>>>>>.>>>>.>.>.
                               ................................................................................................................2.222....................2.2222.2.2.
                               ----------------------------------------------------------------------------------------------------------------o-oooo--------------------o-oo●●-●-G-
```

```
Ga0265745_1000697/596-785      .................................................................UACC.AAC...........CGGCUCCC.UG....................A.UCUCACUCCUGCCUG.C....CGU.AGCA.G.G.
Ga0265747_105373/294-105       .................................................................UACC.AAC...........CGGCUCCC.UG....................A.UCUCACUCCUGCCUG.C....CGU.AGCA.G.G.
Ga0265745_1001084/890-701      ......................................................AU...ACC..AACC.................GACUCCC.UG.AUC.UCACUCC.U......C.GCUGC................CGU.AGCA.G.G.
Ga0265745_1000512/1043-854     ..............................................................A..UACC.AAC...........CGGCUCCC.UG.AUC.UCACUUC.U........GCCCGC...............CGU.AGCA.G.G.
Ga0310117_104105/1058-869      ..................................................................ACC.AAC...........................CGGCUCCC.C.UG..A.UCUCACUCCUGCCUG.C....CGU.AGCA.G.G.
Ga0184587_113157/56-168        ...................................................................................................................A.GGUCCU............AUGCU..CAGA.G.G.
Ga0206356_10600373/215-21      ...................................................................................................................C.GGUACGGUCAC................UUG.A.U.
Ga0307920_126542/349-261       .......................................................................................................................................UUCGCG.AGCG.G.G.
Ga0265742_1001154/131-376      G..UCC...................................................................................AGG.AA.C.G.AGUCAAG.C.U....A.GGUCCUUCUCAACC.........U.UGGU.G.G.
Ga0307482_1127544/505-398      ...............................................................................................................GU..U.GGUCUCUAAUG...............CCG.C.G.
Ga0127503_11058765/362-444     ................................................................................................................G..A.UGGAAA......................C.A.G.
Ga0210323_1050507/418-532      ............................................................................................................................................A.ACUG.A.G.
Ga0242647_1002063/315-62       C..ACA.....G.....CGA..UAGC.....UAAGUAG......AACAGGACCAAG...UAC..AAUA.GC...............................CUAAA....AG..A..GUGAC.................C.UGUG.G.G.
Ga0307484_100992/499-246       C..ACA.....G.....CGA..UAGC.....UAAGUAA......GGUCGGACCAAG...UAC..AAUA......................................GC.C.UA..A.AAGAGUGA..............CC.UGCG.G.G.
Ga0247534_117055/74-212        .....................................................................................GGUCGA..........................GAGACAA...........CU.CUU.CCAA.A.G.
Ga0129331_1494381/292-368      .....................................................................................................................GGGAA.........................A.G.
Ga0242646_1000880/695-856      ...........................................................................................AA.CG.CUC.ACAUUCC.C.CC..A..CUAC................CGA.AGUA.G.G.
Ga0180107_1174136/376-571      ...................................................................................................................C.CUC....................G.CUUG.G.G.
Ga0307921_1050271/251-412      .............................................................................................G.CA.CGG.CCUAGC....U..A.UACA..................GA.GGAC.U.G.
Ga0247524_126530/125-198       ...............................................................................................................................................GGA.U.G.
Ga0242646_1001007/790-609      .....................................................................................................GUAUGGC.U.UC..A.UCCACC...............CG..UGUG.G.G.
Ga0213850_1280121/544-351      C..AUU.....A.....AAA..U.............................................................UGCGGCUU.UG.GAU.......................................GUU.UAGG.G.G.
Ga0307919_1109364/247-158      ............................................................................................................................................G.AGAG.A.G.
Ga0184600_147964/253-339       ............................................................................................................................................C.GAUG.U.G.
Ga0316036_107710/408-322       ............................................................................................................................................C.GAUG.U.G.
Ga0184598_138497/440-355       ...........................................................................................................................................UA.CGAU.G.G.
Ga0184589_121067/502-587       ...........................................................................................................................................UA.CGAU.G.G.
Ga0184601_135946/406-321       ...........................................................................................................................................UA.CGGU.G.G.
Ga0115595_1093158/7-137        ...............................................................................................................GC..U.GGGUCU.................G.CGUG.A.G.
Ga0005851_1006483/274-172      .....................................................................................................UAACAGC.U.UU..G.UGAA.....................UGAG.A.G.
Ga0247522_108409/684-554       ............................................................................................UC.CG.CUG.GCUUCAG...G..G.UGUGUG.................C.CUAG.A.G.
Ga0310119_105137/743-673       ...............................................................................................................................................UAG.A.G.
Ga0247529_116306/255-362       .........................................................................................................AUC.G.AA..G.GUU....................G.UGUC.A.G.
Ga0316046_116376/278-384       .........................................................................................................AUC.G.AA..G.GUU....................G.UGUC.G.G.
Ga0247534_102735/124-18        .........................................................................................................AUC.G.AA..G.GCU....................G.UGUC.A.G.
Ga0247540_102569/210-104       .........................................................................................................AUC.G.AA..G.GUU....................G.UGUC.A.G.
Ga0316037_112281/325-219       ...........................................................................................................U.C.GA..A.GGUU...................G.UGUC.A.G.
Ga0184576_110594/6-113         ...........................................................................................................A.U.CG..A.AGGUU..................G.UGUC.A.G.
Ga0316049_114344/181-288       ...........................................................................................................A.U.CC..A.AGG.CU.................G.UGUC.A.G.
Ga0115594_1200134/154-31       ................................................................................................................C..A.GGG....................C.GGUU.G.G.
Ga0307480_1000549/913-1064     ................................................................................................................U..U.GAGUGUGC...............U.CGCA.G.G.
Ga0242647_1000589/378-557      ................................................................................................................U..G.GGGUACG...............AA.AGAU.C.G.
Ga0222729_1000922/1729-1550    ................................................................................................................U..G.GGGUACG...............AA.AGAU.C.G.
Ga0182091_1104347/98-194       ...................................................................................................................C.UUCA.......................GA.G.G.
Ga0316037_128286/253-347       ...................................................................................................................G.CUC.......................AGA.G.G.
Ga0180107_1040049/1100-1010    ...................................................................................................................G.CGAAU..................C.AGUC.C.G.
Ga0242641_1014724/551-384      ..........................................................................................CGC.AU.GUU.CGACUCU.C.UC..A..CAAA................GG..AGAU.G.A.
Ga0242641_1002366/499-331      ..........................................................................................CGC.AU.GUU.CGACUCU.C.UC..A..CAAA................GG..AGAU.G.A.
Ga0213850_1508869/496-402      ...............................................................................................................UA..G.CAGAG.......................A.A.G.
JGI12272J11983_1072695/40-150  ...............................................................................................................GC..G.GGUAGG.................A..GCG.A.G.
Ga0307482_1020622/600-404      ...........................................................................UCGUGU.GUAUUGCCUAUC.AC.ACG.CU..GA.C.AG..U.CUAAC.................UG.ACAG.G.G.
Ga0242657_1009512/1576-1466    ............................................GUUUAGCGUAAU...UCC..AAG.............................................U..G.GAUAGG.A..................UGU.G.G.
Ga0184577_116723/415-311       ................................................................................................................A..C.CGAUGCC...............AG..GAAU.G.G.
Ga0184576_109717/432-536       ..........................................................................................................................................AG..GAAU.G.G.
Ga0184572_113323/230-300       .............................................................................................................................................GAAA.G.G.
Ga0307354_155926/276-116       ..................GG..AACA.....AACCAGC....................................................CAG.UC.AUA.UGGCUUC.C.GC..G.GCACUCCU...............C.CAAA.G.G.
Ga0265756_100433/485-286       ................................................................................................................U..U.GGGUGUGC...............C.UGCU.G.G.
Ga0265751_100477/874-675       ................................................................................................................U..U.GGGUGUGC...............C.UGCU.G.G.
Ga0180045_119797/166-305       ..........................................................................................GCC.AG.UUC.ACACUAG.C.AC..U..CUCC....................GUUU.U.G.
Ga0222728_1380294/134-238      ........................................................................................................CGCG.U.UG..G.CCA.......................CCC.A.G.
Ga0265758_104499/242-367       ...................................................................................................................G.UCA..........AC.U....CUU.CCGC.G.A.
Ga0079039_1551950/237-315      .....................................................................................................................G....................AG..GCGC.U.G.
Ga0157570_1034241/151-229      ...............................................................................................................................................UGC.A.G.
Ga0157571_1064089/212-134      ...............................................................................................................................................UGC.A.G.
Ga0179951_1211246/478-633      ..........................................................................................................................................AAG.GUUA.U.G.
Ga0179950_1197742/286-441      ...........................................................................................................................................AG.GUUA.U.G.
Ga0184578_120646/150-246       .............................................................................................................A.GA..A.UAGAA......................CU.U.G.
Ga0247526_105246/689-508       .....................................................................................AGACCUGA.GU.G.C.UUUGCAC.U.UG..A.UCCACC...............CGG.AUCA.G.A.
Ga0247538_100442/1363-1181     ...........................................................................GGUGU.UUGAUACCUGAG.UG.CUU.C.UGCAC.U.UG..A.UCCACC...............CGG.AUCA.G.A.
Ga0247515_107373/548-731       ...........................................................................GGUGU.UUGAUACCUGAG.UG.CUU.CUUGCAC.U.UG..A.UCCACC...............CGG.AUCA.G.A.
Ga0247538_100551/721-542       ...........................................................................GGCUG.UUACCCCUGAGU....G.C.UUUGCAC.U.UG..A.UCCACC...............CGG.AUCA.G.A.
Ga0184588_131258/156-253       .....................................................................................GAUGUUG.UC.AAA............................................GCA.U.G.
Ga0316047_100860/1040-888      ...............................AGGAUG.......CUGGAACCUAGU...UAC..AAAA...........................................GG..U.GUUAG.................GA..GCC.G.G.
Ga0242657_1476020/160-244      .............................................................................................................................................GAUU.G.G.
Ga0184602_130623/1321-1100     ..........................................................................................AUG.UG.UAA.CCACGUA.C.AA..G.GAUU..............ACCCGG.UACG.G.G.
Ga0153880_1410217/288-397      ......................................................................................................CACUAU.G.CA..C.AUCAACGUCUG.............A.UGCG.A.G.
Ga0316049_129960/371-262       ............................................................................................................................................C.CUAG.G.G.
Ga0265747_125875/320-395       ............................................................................................................................................A.CGCG.G.G.
Ga0242647_1002473/759-521      U..UAA.....A.......................................UC.....................................AGU.UG.GGU.CACC.AA.C.C...G.CCUGC................CGU.AGCA.G.G.
Ga0242641_1004288/32-112       ...................................................................................................................U.UCGCU.....................CCG.U.G.
Ga0247553_101094/709-629       ................................................................................................................G..A.UCC.........................G.U.G.
Ga0127503_10814644/496-352     ...........................................................................GGUGG.AC....................................................CAUCCA.AUCA.U.G.
Ga0179939_1199779/67-205       ............................................................................................................................................A.AUCG.G.G.
Ga0180107_1021361/468-373      ...................................................................................................................A.GGGCGUGA...............U.GUGU.G.G.
Ga0180107_1218358/314-219      ...................................................................................................................A.GACGAUGA...............A.GUAU.G.G.
Ga0265758_100138/1527-1633     ...........................................................................CGGUA.............................U.UG..G.CCA.......................CCC.G.A.
Ga0265752_100416/442-337       ........................................................................................................GGUG.U.UG..G.CCA.......................CCC.G.A.
Ga0247534_117029/65-195        ........................................................................................................................ACUGGGAUCGA.........U.GAGC.A.G.
Ga0187793_1322799/72-151       ............................................................................................................................................A.GCUC.A.G.

                               ...........................................................................>>>>>>.>>>>>>>>>>>>.>>.>>>.>>>>>>>.>.>>..>.>>>...............>>>>>>.>>>>.>.>.
                               ...................................................................................................................2.222....................2.2222.2.2.
                               -------------------------------------------------------------------------------------------------------------------o-oooo-------------------o-oo●●-●-G-
```

```
Ga0307921_1005950/791-877      ..............................................................................................................................GAGA.G.G.
Ga0180047_1128081/151-64       ......................................................................................................A.GGAA...................GCU.U.G.
Ga0157593_1125711/213-128      ......................................................................................................A.GGAA....................GC.U.G.
Ga0157569_1032488/120-205      ......................................................................................................A.GGAA....................GC.U.G.
Ga0157565_1077069/156-67       ......................................................................................................A.GGAA...................GCU.U.G.
Ga0157593_1023702/238-314      ...............................................................................................................................ACU.G.G.
Ga0255267_1268184/91-184       ........................................................................................................GGUGAU.............GA.GGAG.G.G.
Ga0307919_1081825/191-74       ..................................................................................................GC..U.GUGAUA............CUG.UCUU.G.G.
Ga0307921_1095896/178-61       ..................................................................................................GC..U.GUGAUA............CUG.UCUU.G.G.
Ga0316040_172648/178-66        ......................................................................................................G.GCCAUA............CUG.UCCU.G.G.
Ga0184577_110850/246-358       ......................................................................................................G.GCCAUA............CUG.UCCU.G.G.
Ga0307920_113994/130-9         ......................................................................................................C.GCCAUA............CUG.UCCU.G.G.
Ga0307920_113994/654-539       ........................................................................................................GCCAUA............CUG.UCCU.G.G.
Ga0307921_1037919/342-457      ........................................................................................................GCCAUA............CUG.UCCU.G.G.
Ga0242646_1001980/23-280       A..GAA....A..................CCACUC....AUG.......................CGGG......AA.....A.GC.GAG.ACCACGG.C.UG...A.GCUAAAGAAAAGC.....CGGCAC.GCGG.G.G.
Ga0179935_1139075/165-78       ............................................................................................................................A.AUCG.A.G.
Ga0179944_1246019/249-162      ...........................................................................................................................AG.UCUU.A.G.
Ga0180044_1069332/269-187      ...........................................................................................................................UG.CACA.C.G.
Ga0157564_1065218/271-353      ...........................................................................................................................UG.CACA.A.G.
Ga0180044_1047678/444-368      ............................................................................................................................A.ACCU.G.G.
Ga0180047_1061805/548-622      ............................................................................................................................A.UUCU.G.G.
Ga0157569_1073490/184-258      ............................................................................................................................A.UUCU.G.G.
Ga0213850_1394829/253-105      ...........................................................................GUAGUGU.UG.UUU....CCG.........AUAU.............CCU.CUGA.A.G.
Ga0255293_1138691/440-213      A..GGU....A....................................................................CAC.CC.GUG.UCCUCGC.A.AU...A.UAAAGUU........CGUAUG.UGUG.U.A.
Ga0222728_1000079/1192-1299    ......................................................................................................U.GGGUA..............GG.AUGG.A.G.
Ga0081246_1201717/153-259      ......................................................................................................G.GGUAG...............G.AUGU.G.G.
Ga0222728_1216887/51-157       ......................................................................................................G.GGUAG...............G.AUGG.A.G.
Ga0265754_1141843/290-185      ........................................................................................................G...................G.AUCU.G.G.
JGI12272J11983_1345183/364-258 ........................................................................................................GGA....................UGU.G.G.
Ga0242647_1005934/451-617      ..............................GUAC.....AA..................................UGUGUGUA.AU.CCC.UGGAUCC.C.UC...A.GCCAC............CGU.GGUG.G.A.
Ga0115594_1187657/870-960      ............................................................................................................................U.GGGU.A.G.
Ga0115594_1174394/642-729      ............................................................................................................................U.GGGU.A.G.
Ga0184569_112764/943-836       ......................................................................................................C.ACAAUCCCAAGCGGG.U....AGG.AUGA.G.G.
Ga0265750_1013321/389-496      ......................................................................................................C.ACAAUCCCAAGCGGG.U....AGG.AUCU.G.G.
Ga0265757_139522/177-70        ......................................................................................................C.ACAAUCCCAAGCGGG.U....AGG.AUCU.G.G.
Ga0316029_116217/368-261       ......................................................................................................G.GGUA...............GG.AUGA.G.G.
Ga0316047_100016/2681-2574     ......................................................................................................G.GGUA...............GG.AUGA.G.G.
Ga0256352_1101982/155-277      ..................................................................................................GA..G.GAU...................GAGA.C.G.
Ga0256350_1100736/283-405      ..................................................................................................GA..G.GAU...................GAGA.C.G.
Ga0079039_1443516/199-38       ................................................................................................C.UA..G.GACACUC...........CCC.GGAA.A.G.
Ga0115595_1072788/334-441      ............................................................A.AA......................................C.GCA....................CCU.A.G.
Ga0115594_1022316/346-239      ............................................................A.AA......................................C.GCA....................CCU.A.G.
Ga0129331_1058147/189-99       ............................................................................................................................U.GGUG.G.G.
Ga0265751_100127/176-103       ............................................................................................................................G.ACUC.A.G.
Ga0307921_1043977/91-164       ............................................................................................................................A.AGUU.U.G.
Ga0307919_1048575/242-90       A..UGG....A....................................................A.................C.GG.CCU.AGCAGAU.U.UA..U.GUGGAC............UGA.GGAC.U.G.
Ga0242646_1001086/737-571      .......................................................................UCAAUGUUU.UC.CG.AGG.AUCCUGC.C.CC..C.GCC...............GC..GGCG.U.G.
Ga0265752_111922/418-343       ............................................................................................................................A.GACG.C.G.
Ga0157562_1134247/69-160       ...................................................................................................A..G.GCUG...................AGG.G.G.
Ga0157559_1047848/128-41       ...................................................................................................A..G.GCUG...................AGG.G.G.
Ga0179944_1313268/129-232      ......................................................................................................A.GGG.................U.UGAG.G.G.
Ga0213850_1259428/445-342      ..................................................................................................AG..G.GCU....................GAG.A.G.
Ga0153880_1167367/224-121      ..................................................................................................AG..G.GCU....................GAG.A.G.
Ga0153880_1058095/344-241      ..................................................................................................AG..G.GCU....................GAG.A.G.
Ga0179943_1178349/195-298      ..................................................................................................AG..G.GUU....................GAG.G.G.
Ga0307921_1075885/188-36       ......................................................................................................G.UGGAC.............UAA.GGAC.U.G.
Ga0307921_1062541/180-338      ........................................................................................................UGGAC.............UGG.GGAC.U.G.
Ga0242641_1014474/336-257      ............................................................................................................................C.GGAU.GCG.
Ga0307919_1064641/225-73       ..................................................................................................AU..G.UGGAC.............UAA.GGAC.U.G.
Ga0265759_108120/277-351       ...............................................................................................................................GGU.G.A.
Ga0265753_1005277/856-1057     C..GCC....A.....GC..ACGA....CGCUACU....GUUGCCCUGCCC..UCC..UCC.............AUGUUUGG.AG.AUU.CAGGCCG.C.....................UU..U.CGCG.U.G.
Ga0247523_120120/325-403       ............................................................................................C...............................A.CAUG.U.A.
Ga0228707_1100139/344-194      .........................................CGAUUGGUCC..UGC..CAAG.GACUGGUGU.C..................................................G.UGAU.G.A.
Ga0316049_154526/155-74        ......................................................................................................A.AUAU..................UCCU.G.G.
Ga0316035_177575/224-142       ..............................................................................................................................UCCU.G.G.
Ga0307920_126403/177-486       CACAUG....A....CAG..UACU....CACACCA....ACUUUCCGAACC..UCA..GAGG.AUACGGA.G.UAA.UGGCG...................G.UACAGUGUGUGAAAU.C....UG.AACG.U.G.
Ga0138280_1140856/341-479      A..AGC....A...........................................................................................A.AUG.............ACGCGC.UACA.A.G.
Ga0247539_101233/414-526       ........................................................................................................GGCA................C.AUAG.G.C.
Ga0184569_108885/200-88        ........................................................................................................GGCA................C.AUAG.G.C.
Ga0247521_112559/317-205       ........................................................................................................GGCA................C.AUAG.G.C.
Ga0184599_154888/232-155       ......................................................................................................A.CCCA.....................U.A.G.
Ga0180108_1143860/199-116      ........................................................................................................................UCAAAU.CUAC.C.U.
Ga0247524_121352/374-303       .........................................................................................................GA......................U.C.G.
Ga0206356_11002907/329-148     ...................................................................................................G..A.GGGCCG..............G.UCUU.A.U.
Ga0265743_103007/946-842       ......................................................................................................G.GGUA...............GA.AUAG.A.G.
Ga0316033_101029/151-49        ...................................................................................................C..G.AUCAUA..............C.CAUCUC.G.
Ga0179937_1126086/216-325      ..........................................................................................................................CAC.GCUA.C.G.
Ga0307482_1050048/176-95       ..................................................................................................GG..G.G.....................GUCC.C.A.
JGI12272J11983_1319142/230-159 ...............................................................................................................................CCC.C.U.
Ga0180107_1285918/13-113       ..........................................................................................GUGAUA.A.CA..A.GAG..............CCC.UUAU.U.G.
Ga0265756_108694/382-277       ........................................................................................................................UUUCGC.UCCA.A.G.
Ga0265746_1140777/214-319      ........................................................................................................................UUUCGC.UCCA.A.G.
Ga0184574_101359/409-493       ..............................................................................................................................GUGA.A.G.
Ga0184568_108556/391-307       ..............................................................................................................................GUGA.A.G.
Ga0316030_107573/391-475       ..............................................................................................................................GUGA.A.G.
Ga0180107_1031754/139-57       ...........................................................................................................................UG.UUAG.U.G.
Ga0307919_1029784/253-144      ......................................................................................................U.ACG.....................CA.U.G.
Ga0307921_1042549/343-452      ......................................................................................................U.ACG.....................CA.U.G.
Ga0307482_1021658/484-563      ........................................................................................................GAA.................G.UGUA.U.G.

                               .................................................................>>>>>>.>>>>>>>>>>>>.>>.>>>.>>>>>>>.>.>>...>.>>>...............>>>>>>.>>>>.>.>.
                               ......................................................................................................2.222...................2.2222.2.2.
                               ------------------------------------------------------------------------------------------------------o-oooo------------------o-oooo-o-G-
```

```
Ga0179943_1102267/215-90        ......................................................................................................CUGUG.U.GA...G.GCU.......................CGA.C.G.
Ga0079074_1279626/114-274       .........................................................................U..........GG.................CUUC.C.GC...G.GCACUCCU...............C.CAAA.G.G.
Ga0307921_1019702/90-184        ...............................................................................................................A...G.CAG.......................UCC.G.G.
Ga0307920_133961/233-140        ...............................................................................................................A...G.CAG.......................UCC.G.G.
Ga0316035_179807/270-184        ............................................................................................................................................C.GGAC.G.A.
Ga0256338_1343513/152-228       ...............................................................................................................................................GUG.A.G.
Ga0255268_1432700/127-51        ...............................................................................................................................................GUG.A.G.
Ga0153880_1067318/492-566       ...............................................................................................................................................UAC.G.G.
Ga0247533_103075/417-305        ..............................................................................................................AA...G.GCGAUA...............CCU.CUUC.G.G.
Ga0247541_102964/56-168         ..............................................................................................................A....G.GCGAUA...............CCU.CUUC.G.G.
Ga0184589_110648/1041-881       ........................................................................................AUGG.AA.UGG.GUGAUCC.C.UC...A.GCUAC................CGA.AGUA.G.G.
Ga0184580_124121/239-79         ........................................................................................AUGG.AA.UGG.GUGAUCC.C.UC...A.GCUAC................CGA.AGUA.G.G.
Ga0184598_101324/530-690        ........................................................................................AUGG.AA.UGG.GUGAUCC.C.UC...A.GCUAC................CGA.AGUA.G.G.
Ga0184588_120624/266-426        ........................................................................................AUGG.AA.UGG.GUGAUCC.C.UC...A.GCUAC................CGA.AGUA.G.G.
Ga0184579_106194/996-1156       ........................................................................................AUGG.AA.UGG.GUGAUCC.C.UC...A.GCUAC................CGA.AGUA.G.G.
Ga0265758_100254/356-516        ........................................................................................AUGG.AA.UGG.GUGAUCC.C.UC...A.GCUAC................CGA.AGUA.G.G.
Ga0184597_106861/196-36         ........................................................................................AUGG.AA.UGG.GUGAUUC.C.UC...A.GCUAC................CGA.AGUA.G.G.
Ga0184600_132066/568-408        ........................................................................................AUGG.AA.UGG.GUGAUUC.C.UC...A.GCUAC................CGA.AGUA.G.G.
Ga0316031_108320/216-376        ........................................................................................AUGG.AA.UGG.GUGAUUC.C.UC...A.GCUAC................CGA.AGUA.G.G.
Ga0184596_109261/979-1139       ........................................................................................AUGG.AA.UGG.GUGAUUC.C.UC...A.GCUAC................CGA.GGUA.G.G.
Ga0316035_107544/354-514        ........................................................................................AUGG.AA.UGG.GUGAUCC.C.UC...A.GCUAC................CGA.AGUA.G.G.
Ga0247527_115808/280-440        ........................................................................................AUGG.AA.UGG.GUGAUCC.C.UC...A.GCUAC................CGA.GGUA.G.G.
Ga0247519_107918/217-58         .........................................................................................AUC.GC.AGA.ACACUUC.C.UC...A.GCUACC................GG.AGUA.G.G.
Ga0184589_112135/40-200         .........................................................................................AUC.GC.GGC.ACACUUC.C.UC...A.GCUAC................CGG.AGUA.G.G.
Ga0247551_100195/490-329        .........................................................................................AUC.GU.GGC.ACACUUC.C.UC...A.GCUAC................CGG.AGUA.G.G.
Ga0247526_117235/152-313        .........................................................................................AUC.GC.GGC.ACACUUC.C.UC...A.GCUAC................CGG.AGUA.G.G.
Ga0326631_101563/544-705        .........................................................................................AUC.GC.AGC.ACACUUC.C.UC...A.GCUAC................CGG.AGUA.G.G.
Ga0184596_111007/525-686        .........................................................................................AUC.AC.AGA.ACACUUC.C.UC...A.GCUAC................CGG.AGUA.G.G.
Ga0184573_110190/115-276        .........................................................................................AUC.GC.AGA.ACACUUC.C.UC...A.GCUAC................CGG.AGUA.G.G.
Ga0316033_144939/167-328        .........................................................................................AUC.AC.AGA.ACACUUC.C.UC...A.GCUAC................CGG.AGUA.G.G.
Ga0247552_101742/612-452        .........................................................................................AUG.AC.GGA.ACACUUC.C.UC...A.GCUAC................CGG.AGUA.G.G.
Ga0247545_112960/183-343        .........................................................................................AUA.GC.GGA.CCACUUC.C.UC...A.GCUAC................CGG.AGUA.G.G.
Ga0247541_103784/123-283        .........................................................................................AUA.GC.GGA.CCACUUC.C.UC...A.GCUAC................CGG.AGUA.G.G.
Ga0247524_111427/50-210         .........................................................................................AUG.GC.GGA.CCACUUC.C.UC...A.GCUAC................CGG.AGUA.G.G.
Ga0247545_104341/406-246        .........................................................................................AUG.AC.GGA.ACACUUC.C.UC...A.GCUAC................CGG.AGUA.G.G.
Ga0184602_114416/11-171         .........................................................................................AUG.AC.GGA.CCACUUC.C.UC...A.GCUAC................CGG.AGUA.G.G.
Ga0316038_111670/11-171         .........................................................................................AUG.AC.GGA.CCACUUC.C.UC...A.GCUAC................CGG.AGUA.G.G.
Ga0184586_133850/368-528        .........................................................................................AUG.AC.GGA.CCACUUC.C.UC...A.GCUAC................CGG.AGUA.G.G.
Ga0184600_123882/188-28         .........................................................................................AUG.GC.GGG.CCACUUC.C.UC...A.GCUAC................CGG.AGUA.G.G.
Ga0184574_121290/269-109        .........................................................................................AUA.GC.GGA.CCACUUC.C.UC...A.GCUAC................CGG.AGUA.G.G.
Ga0316032_107575/215-55         .........................................................................................AUG.GC.GGA.CCACUUC.C.UC...A.GCUAC................CGA.AGUA.G.G.
Ga0247519_110701/607-447        .........................................................................................AUG.GC.GGA.CCACUUC.C.UC...A.GCUAC................CGA.AGUA.G.G.
Ga0247526_110221/603-443        .........................................................................................AUG.AC.GGA.ACACUUC.C.UC...A.GCUAC................CGG.AGUA.G.G.
Ga0247529_134913/224-64         .........................................................................................AUG.GC.GGA.CCACUUC.C.UC...A.GCUAC................CGA.AGUA.G.G.
Ga0247529_103231/270-430        .........................................................................................AUC.GC.AGA.ACACUUC.C.UC...A.GCUAC................CGG.AGUA.G.G.
Ga0184580_114961/306-146        .........................................................................................AUC.GC.AGA.ACACUUC.C.UC...A.GCUAC................CGG.AGUA.G.G.
Ga0184603_126764/80-240         .........................................................................................AUC.GC.AGA.ACACUUC.C.UC...A.GCUAC................CGG.AGUA.G.G.
Ga0247550_114736/167-7          .........................................................................................AUC.GC.AGA.ACACUUC.C.UC...A.GCUAC................CGG.AGUA.G.G.
Ga0184603_111918/241-82         .........................................................................................AUC.GC.AGA.ACACUUC.C.UC...A.GCUAC................CGG.AGUA.G.G.
Ga0247538_100424/120-281        .........................................................................................AUC.GC.AGA.ACACUUC.C.UC...A.GCUAC................CGG.AGUA.G.G.
Ga0247547_102464/189-29         .........................................................................................ACG.AG.CAG.GCACUUC.C.UC...A.GCUAC................CGG.AGUA.G.G.
Ga0247551_100828/546-387        .........................................................................................ACG.AG.CAG.GCACUUC.C.UC...A.GCUAC................CGG.AGUA.G.G.
Ga0247538_100480/788-947        .........................................................................................ACG.AG.CAG.GCACUUC.C.UC...A.GCUAC................CGG.AGUA.G.G.
Ga0247550_100850/456-297        .........................................................................................ACG.AG.CAG.GCACUUC.C.UC...A.GCUAC................CGG.AGUA.G.G.
Ga0247536_101914/360-201        .........................................................................................ACG.AG.CAG.GCACUUC.C.UC...A.GCUAC................CGG.AGUA.G.G.
Ga0247553_110698/526-367        .........................................................................................ACG.AG.CAG.GCACUUC.C.UC...A.GCUAC................CGG.AGUA.G.G.
Ga0153880_1114976/150-383       U..UGC.....A.....GAG..ACAC.....UAUGCUC......UACAAGGGCACG...CAC.GUAC................................................G.UACAGGC................A.GGAA.A.G.
Ga0180052_189627/240-346        ..............................................................................................................GC...C.GCGCAA.............GUAGGAGGGGU.G.G.
Ga0184598_136114/144-251        ........................................................................CGGU..............................G.U.UG...G.CCA.......................CCC.A.G.
Ga0222728_1011988/841-947       .......................................................................................................GGCG.U.UG...G.CCA.......................CCC.A.G.
Ga0265751_160438/108-215        ..............................................................................................................CG...C.GGUAG.......................G.U.G.
Ga0247514_107497/1071-1140      ................................................................................................................................................GA.G.G.
Ga0180107_1129325/244-60        ......................................................................................AGUGCA.UU.....GU...CU.G.AC...U.GGAAC.............CUC..C.AUAA.G.G.
Ga0242646_1002014/11-270        .........................U.....AAAAUA...............................................UGUGUUAU.CC.UUG.GGAGACU.U.GG...A.UAGCAUGAACA...........AGG.GAUA.A.G.
Ga0222728_1008526/24-283        .........................U.....AAAAUA...............................................UGUGUUAU.CC.UUG.GGAGACU.U.GG...A.UAGCAUGAACA...........AGG.GAUA.A.G.
Ga0222728_1008391/1281-1023     A..AAC.....A.....GAU..AGGU.....AAAAUA...............................................UGUGUUAU.CC.UUG.AGAGACU.U.GG...A.UAGCAUGAACA...........AAG.GAUA.A.G.
Ga0265759_100017/4211-4288      ...................................................................................................................G.......................AU.GUAU.G.G.
Ga0247512_104440/911-1082       .....................................................................................................CAA.UG.C.AA...A.ACAGGCGUCUGCCAC.U.....GU.GGUG.G.G.
Ga0265756_131580/159-58         .....................................................................................................................UU...................UAG.CUCU.G.G.
Ga0310119_169621/253-177        ............................................................................................................................................A.UGGG.A.G.
Ga0247516_124175/202-272        ...............................................................................................................................................GUG.U.G.
Ga0247534_100772/309-234        ...................................................................................................................G.UUG.........................A.C.G.
Ga0222728_1054736/119-193       ............................................................................................................................................U.UGUA.GCG.
Ga0180106_1297567/444-515       ...........................................................................................................................................GU.UCUA.G.A.
Ga0242647_1000066/2638-2719     ...................................................................................................................A.GAGUUUG...................UAG.G.G.
Ga0307484_104189/90-171         ..........................................................................................................................................AGU.UCGG.A.G.
Ga0316048_109289/45-149         .......................................................................................................UGUG.U.UG...G.CCA.......................CCC.A.G.
Ga0265750_1038125/531-427       .......................................................................................................UGUG.U.UG...G.CCA.......................CCC.A.G.
Ga0247537_109516/130-26         .......................................................................................................UGUG.U.UG...G.CCA.......................CCC.A.G.
Ga0310115_103434/744-848        .......................................................................................................UGUG.U.UG...G.CCA.......................CCC.A.G.
Ga0307482_1009758/1126-1021     ......................................................................................................CGAUG.U.UG...G.CCA.......................CCC.A.G.
JGI12272J11983_1356304/1548-1654 .....................................................................................................UGAUG.U.UG...G.CCA.......................CCC.A.G.
JGI12272J11983_1072695/645-539  .......................................................................................................GAUG.U.UG...G.CCA.......................CCC.A.G.
Ga0242657_1009512/820-926       ....................................................................................G..................GAUG.U.UG...G.CCA.......................CCC.A.G.
Ga0316037_172473/89-196         ......................................................................................................GCGUG.U.UG...G.CCA.......................CCC.G.G.
Ga0184592_121084/378-271        ......................................................................................................GCGUG.U.UG...G.CCA.......................CCC.G.G.
Ga0184569_113518/504-609        .........................................................................U.............................CGUG.U.UG...G.CCA.......................CCC.G.G.
Ga0184602_114837/392-286        ......................................................................................................UCGUG.U.UG...G.CCA.......................CCC.A.G.
Ga0316030_102131/386-280        ......................................................................................................UCGUG.U.UG...G.CCA.......................CCC.A.G.

                                .........................................................................>>>>>>.>>>>>>>>>>>>.>>.>>>.>>>>>>>.>.>>...>.>>>...............>>>>>>.>>>>.>.>.
                                ...................................................................................................................2.222....................2.2222.2.2.
                                -------------------------------------------------------------------------------------------------------------------o-oooo-------------------o-ooo●●-●-G-
```

```
Ga0310118_104334/336-443         ......................................................................................................UCUG.U.UG...G.CCA.......................CCC.G.A.
Ga0180108_1146973/462-263        C..CGA.....A.....AGG..UGAC.....CAUUCAG......GCGUGACUGUCC...UAA..GUCC.AGU............GUGUUCU.UC.UUC....GUGA.G.GG...A.CCGAC..................U.CAUC.C.G.
Ga0184580_111164/678-749         ..............................................................................................................................................GAG.G.GU
Ga0242647_1006852/526-598        ..............................................................................................................................................CUG.G.GC
Ga0179939_1059820/107-216        ...........................................................................................................................................G.CUGU.U.G.
Ga0115594_1156252/268-370        ....................................................................................................................GCCACA...............AAU.GGUA.C.G.
Ga0115594_1006122/120-29         ...........................................................................................................................................U.CGGU.A.G.
Ga0115595_1180708/226-317        ...........................................................................................................................................U.CGGU.A.G.
Ga0115594_1211214/299-389        ...........................................................................................................................................U.CGUG.C.G.
Ga0115594_1007070/120-30         ...........................................................................................................................................U.GGUA.C.G.
Ga0115595_1181384/176-86         ...........................................................................................................................................U.GGUA.C.G.
Ga0115594_1166236/119-19         ....................................................................................................................GCCACAA...............AU.CGUA.C.G.
Ga0115594_1157594/155-254        ....................................................................................................................GCCACAA...............AU.CGUA.C.G.
Ga0256352_1182139/180-81         ....................................................................................................................GCCACA...............AGU.CGUA.C.G.
Ga0115594_1182529/183-84         ....................................................................................................................GCCACAA...............AU.CGUA.C.G.
Ga0265746_1064626/389-458        ..............................................................................................................................................GGU.A.G.
Ga0115594_1016490/326-410        .........................................................................................................................................CG...GUG.C.G.
Ga0115595_1007417/328-412        .........................................................................................................................................CG...GUG.C.G.
Ga0228708_1103570/188-285        ..................................................................................................................C.GAU....................G.CAUU.C.G.
Ga0265751_121301/189-266         ...........................................................................................................................................A.ACAU.C.G.
Ga0179944_1005311/261-382        .........................................................................................................C.U.GU...G..UGAGG.................C.CGGG.A.G.
Ga0179944_1191552/284-411        .........................................................................................................C.U.GU...G..UGAGG.................C.UCGA.U.G.
Ga0179943_1150468/216-89         .......................................................................................................AGC.G.AU...G..UGAGG.................C.UUGA.A.G.
Ga0179944_1204933/216-89         .......................................................................................................AGC.G.AU...G..UGAGG.................C.UUGA.A.G.
Ga0307482_1020425/440-614        ........................................................................UGUU.C.UUCCUGCC......C.CGC.AUGUUAG.G.GU...A.GCUAA.............C..CAA.GCUA.G.G.
Ga0242646_1001395/255-509        A..GAA.....A.....UCA..CACA.....UGCGGGA......AAGCGAGACCAC...GCC..CAU.............................AG.GAAAUAU.C.CU...U.AGG..................GAU.GGUG.G.G.
Ga0184578_112031/156-44          ..................................................................................................................U.GGCA..................CA.UUCG.G.C.
Ga0184577_120131/220-108         ..................................................................................................................U.GGCA..................CA.UUCG.G.C.
Ga0247517_121471/387-499         ....................................................................................................................GGCA..................CA.UUCG.G.C.
Ga0247539_107237/153-229         ....................................................................................................................G....................AUA.UUAG.U.G.
Ga0265758_109576/83-157          ...........................................................................................................................................G.UGGU.G.G.
Ga0184577_107529/254-327         ...........................................................................................................................................A.UGAC.C.G.
Ga0213850_1246156/171-47         .............................................................................................U.GG...CAG.CC.U.CU...U.CCCA.......................AG.G.G.
Ga0326631_172366/284-207         ..............................................................................................................................................ACA.U.G.
Ga0310114_149639/68-153          ..........................................................................................................................................GG.UGGC.G.G.
Ga0184599_128731/188-273         ..........................................................................................................................................GG.UGGC.G.G.
Ga0310119_131358/533-447         ..................................................................................................................G.GUG........................CC.G.G.
Ga0247526_116548/279-354         ...........................................................................................................................................A.UCCU.C.G.
Ga0247519_113845/334-409         ...........................................................................................................................................A.UCUU.C.G.
Ga0247529_111070/303-228         ...........................................................................................................................................A.UCUU.C.G.
Ga0184580_116530/249-177         ..............................................................................................................................................GUC.U.G.
Ga0247524_105844/561-637         .....................................................................................................................ACGA......................UC.U.G.
Ga0184593_119849/313-238         ..................................................................................................................G.UACAGA......................C.U.G.
Ga0184599_140898/774-849         ..................................................................................................................G.UACAGA......................C.U.G.
Ga0316049_111814/256-181         ..............................................................................................................A..........................CAC.UAAC.U.G.
Ga0316034_100493/254-174         .....................................................................................................................AC....................A.CGUC.U.G.
Ga0184600_118565/177-97          .....................................................................................................................AC....................A.CGUC.U.G.
Ga0184602_122864/595-515         ..............................................................................................................U............................A.CAUC.U.G.
Ga0184596_126546/83-162          ..............................................................................................................U...A.CA.........................UC.U.G.
Ga0184597_129399/366-446         ..............................................................................................................U..........................ACA.CGUC.U.G.
Ga0184576_117703/380-460         ..............................................................................................................U..........................ACA.CGUC.U.G.
Ga0184590_111002/143-223         ..................................................................................................................A.UAUAC.....................AUC.U.G.
Ga0247551_103383/322-242         .............................................................................................................CU..........................AUA.CAUC.U.G.
Ga0184597_111165/262-182         .............................................................................................................CU...A.U......................A.CAUC.U.G.
Ga0184570_116436/278-198         .............................................................................................................CU...A.U......................A.CAUC.U.G.
Ga0316037_169124/279-199         .............................................................................................................CU...A.U......................A.CAUC.U.G.
Ga0247543_103170/244-164         ..................................................................................................................C.UAUAC.....................AUC.U.G.
Ga0184589_120451/249-329         ..................................................................................................................C.UAUAC.....................AUC.U.G.
Ga0247519_108375/286-206         ..................................................................................................................C.UAU....................A.CAUC.U.G.
Ga0184587_105985/71-151          ..................................................................................................................G.ACUAU..................A.CACC.U.G.
Ga0184593_101086/682-762         ..................................................................................................................G.ACUAU..................A.CAUC.U.G.
Ga0184602_121948/324-245         ..............................................................................................................U...A.CAGA........................A.C.G.
Ga0184597_110829/1090-1011       ..............................................................................................................U...A.CAGAC.........................U.G.
Ga0184578_129447/314-235         ....................................................................................................................CAG........................AA.C.G.
Ga0184588_113246/331-252         ..................................................................................................................A.CAGA........................C.U.G.
Ga0316051_1003245/138-56         ....................................................................................................................A......................C.GAAC.U.G.
Ga0316046_122965/221-142         ....................................................................................................................A......................C.GAAC.U.G.
Ga0184600_105920/81-160          ...........................................................................................................................................C.AGUC.U.G.
Ga0184603_125382/9-88            ..............................................................................................................U...A.UACGA......................UC.U.G.
Ga0247550_100138/208-129         ..............................................................................................................U...A.UACAA......................AC.U.G.
Ga0316049_122425/209-288         ....................................................................................................................UAC......................AAAC.U.G.
Ga0032354_1062690/2659-2586      .........................................................................................................................................UGC.AGGG.U.G.
Ga0179955_1048341/123-51         ...............................................................................................................................................GC.C.G.
Ga0138277_1023917/182-18         A..GAA.....A..........................................................................................GAAC.G.GC...A.GAUG..............GCGCGC.UACA.A.G.
Ga0138282_1020546/737-573        A..GAA.....A..........................................................................................GAAC.G.GC...A.GAUG..............GCGCGC.UACA.A.G.
Ga0242641_1105635/265-351        ...........................................................................................................................................G.ACAC.U.G.
Ga0242654_10795375/263-349       ...........................................................................................................................................G.ACAC.U.G.
Ga0247554_113506/254-161         ..................................................................................................................C.CAU....................C.AUAU.G.G.
Ga0265745_1000618/793-552        ....................................................................................UUGGGUA.AA.CCG.AAUUGGG.C.GU...C.GCCACCGCCUGC.........CGU.AGCA.G.G.
Ga0265742_1001052/322-80         ....................................................................................UUGGGUA.AA.CCG.AAUUGGG.C.GA...C.GCCUCCGCCUGC.........CGU.AGCA.G.G.
Ga0310113_106868/972-730         ....................................................................................UUGGGUA.AA.CCG.AAUUGGG.C.GA...C.GCCUCCGCCUGC.........CGU.AGCA.G.G.
Ga0265750_1009219/272-30         ....................................................................................UUGGGUA.AA.CCG.AAUUGGG.C.GU...C.GCCACCGCCUGC.........CGU.AGCA.G.G.
Ga0265740_1001378/1084-842       ....................................................................................UUGGGUA.AA.CCG.AAUUGGG.C.GA...C.GCCUCCGCCUGC.........CGU.AGCA.G.G.
Ga0265752_100504/930-688         ....................................................................................UUGGGUA.AA.CCG.AAUUGGG.C.GA...C.GCCUCCGCCUGC.........CGU.AGCA.G.G.
Ga0265747_100653/495-253         ....................................................................................UUGGGUA.AA.CCG.AAUUGGG.C.GA...C.GCCUACGCCUGC.........CGU.AGCA.G.G.
Ga0265757_104669/135-376         ....................................................................................UUGGGUA.AA.CCG.AAUUGGG.C.GA...C.GCCUCCGCCUGC.........CGU.AGCA.G.G.
Ga0265742_1088739/51-292         ....................................................................................UUGGGUA.AA.CCG.AAUUGGG.C.GU...C.GCCACCGCCUGC.........CGU.AGCA.G.G.
Ga0265747_100509/1277-1036       ....................................................................................UUGGGUA.AA.CCG.AAUUGGG.C.GU...C.GCCACCGCCUGC.........CGU.AGCA.G.G.
Ga0265752_100314/1466-1225       ....................................................................................UUGGGUA.AA.CCG.AAUUGGG.C.GU...C.GCCACCGCCUGC.........CGU.AGCA.G.G.

                                 ........................................................................>>>>>>.>>>>>>>>>>>>.>>.>>>.>>>>>>>.>.>>...>.>>>...............>>>>>>.>>>>.>.>.
                                 ..................................................................................................................2.222....................2.2222.2.2.
                                 ------------------------------------------------------------------------------------------------------------------o-oooo-------------------o-o●●●-●-G-
```

```
Ga0213850_1450246/323-606      U..GAG.....AGCUCCAAG..UGGG.....AGCAUCC......CUAGUGUUUAAC...CUG..UACG.UAC...........UGGACUUG.UC.CUG.AGUACUG...GC...U.UU................CCACG..GGGG.A.G.
Ga0157593_1101573/285-478      A..UGA.....A..................................................................................................C...G.GCU...............U.GUGG.G.G.
Ga0222728_1000125/1315-1238    ..................................................................................................................G.....................AU..GUAG.A.G.
Ga0242654_10000586/1273-1196   ........................................................................................................................................GU..UAUG.A.G.
Ga0265756_100130/1310-1239     .........................................................................................................................................A.UGUU.G.G.
Ga0307482_1008742/1212-1288    ........................................................................................................................................GA.UGUG.G.G.
Ga0242654_10040657/1018-942    .......................................................................................................................................UUG.CGUA.G.G.
Ga0307484_100842/1194-1271     ........................................................................................................................................UG.UGUA.G.G.
Ga0307482_1009223/670-508      .......................................................................................................GGC.U.UC...A.UCCAC...............CCG..UGUG.G.G.
Ga0307484_108094/186-350       .......................................................................................................GGC.U.UC...A.UCC...............ACCCG..UGUG.G.G.
Ga0247514_114593/280-211       .............................................................................................................................................GA.G.G.
Ga0184583_106441/296-227       ............................................................................................................................................GUG.G.G.
Ga0242654_10672177/244-165     ....................................................................................................................GGC.......................GGU.G.A.
Ga0126318_10229384/609-688     .......................................................................................................................................AAG.UUGC.U.A.
Ga0247551_122551/29-115        .............................................................................................................CC...C.CCU...................A.AUAU.U.G.
Ga0316048_107802/239-324       .............................................................................................................CC...C.CCU...................A.AUAU.U.G.
Ga0242641_1002064/1203-1116    ...........................................................................................................C.GC...U.G...................GG.AUUU.U.G.
Ga0307482_1026216/884-813      .........................................................................................................................................G.AUAG.U.G.
Ga0307482_1636410/270-341      .........................................................................................................................................G.AUAG.U.G.
Ga0307482_1016877/168-97       .........................................................................................................................................G.AUGG.U.G.
Ga0180107_1106188/424-495      .........................................................................................................................................U.CGGU.U.G.
Ga0307482_1000354/2063-1987    ........................................................................................................................................UG.CUGU.U.G.
Ga0184587_107783/167-95        .........................................................................................................................................U.CACC.A.G.
Ga0184585_128240/467-390       .......................................................................................................................................UUG.UGGA.U.G.
Ga0184596_102741/625-701       .......................................................................................................................................ACA.UAGG.C.G.
Ga0247539_100086/1763-1850     ......................................................................................................................................UCAUGG.UAAG.GUG.
Ga0184584_115572/267-354       ......................................................................................................................................UCAUGG.UAAG.GUG.
Ga0184579_105257/1735-1648     ......................................................................................................................................UCAUGG.UAAG.GUG.
Ga0307482_1023270/273-197      .......................................................................................................................................CUG.GGGU.U.G.
Ga0307482_1219330/19-95        ........................................................................................................................................GG.UGUG.GCG.
Ga0242657_1207391/462-383      .......................................................................................................................................UAU.GUUG.U.G.
Ga0247520_104849/891-821       ............................................................................................................................................GAU.G.A.
Ga0247531_104861/1082-1012     ............................................................................................................................................GAU.G.A.
Ga0247518_137824/301-231       ............................................................................................................................................GAU.G.A.
Ga0307482_1202605/470-395      ........................................................................................................................................GC.AAGG.C.G.
Ga0307921_1021783/160-261      .............................................................................................................AA...G.CGC...............................AC.G.G.
Ga0242657_1054283/541-620      .........................................................................................................................................U.GAAC.C.G.
Ga0242647_1005188/439-360      ..............................................................................................................................................C.C.G.
Ga0153880_1391790/62-140       ...........................................................................................................................................GACC.G.G.
Ga0153880_1379927/66-144       ...........................................................................................................................................GACC.G.G.
Ga0307482_1023665/853-778      .........................................................................................................................................A.CUGU.G.G.
Ga0242646_1047905/92-182       ....................................................................................................................GGCCG..................C.AGGA.G.A.
Ga0307921_1024041/406-334      ............................................................................................................................................UUC.G.G.
Ga0307919_1039507/513-440      ............................................................................................................................................UUC.G.G.
Ga0307919_1084008/308-378      ..............................................................................................................................................C.G.G.
Ga0247525_114510/199-318       ...................................................................................................GGAAGGU.C.CU...G.UUGACU...................GUUC.G.G.
Ga0184603_107262/477-596       ...................................................................................................GGAAGGU.C.CU...G.UUGACU...................GUUC.G.G.
Ga0184585_151549/495-376       ...................................................................................................GGAAGGU.C.CU...G.UUGACU...................GUUC.G.G.
Ga0310119_113172/381-306       ........................................................................................................................................GG.GUUU.G.G.
Ga0310114_133004/56-132        ........................................................................................................................................GG.GUUU.G.G.
Ga0184603_148109/996-920       ........................................................................................................................................GG.GUUU.G.G.
Ga0184599_110049/275-349       ........................................................................................................................................GG.GUUU.G.G.
Ga0184587_132427/565-485       ........................................................................................................................................GG.GAUU.G.G.
Ga0265756_135986/152-290       .............................................................................................................AG...C.AGGAAAC..................G.GUUC.G.G.
Ga0247519_104852/800-730       ....................................................................................................................GGGU............................C.U.G.
Ga0213850_1274705/160-417      U..UUU.....A.....UAA..AGGA.....CCCUUGU......CAU.....................................GAGGUGA.UC.CUG.GGC.......UU...G.UGAA..............GGGCUG.GGUA.U.G.
Ga0265747_100454/1395-1324     ............................................................................................................................................UGA.C.G.
Ga0247519_118271/334-405       ..............................................................................................................................................C.C.G.
Ga0242654_10135580/213-336     G...................................................................................................AACU.....GA...C.GCA....................C.UUGC.U.G.
Ga0184576_110410/277-348       .........................................................................................................................................A.CCCA.U.G.
Ga0247535_105396/277-348       .........................................................................................................................................A.CCUA.U.G.
Ga0265758_104558/436-365       .........................................................................................................................................A.GGGA.U.G.
Ga0247526_117664/434-499       .............................................................................................................................................CA.U.G.
Ga0316037_123461/289-368       .............................................................................................................GA...C.AU............................G.C.G.
Ga0247543_100863/642-721       ..............................................................................................................G...A.CAU...........................G.C.G.
Ga0247521_124040/388-309       ..............................................................................................................G...A.CAU...........................G.C.G.
Ga0184589_120335/11-90         ..............................................................................................................G...A.CAU...........................G.C.G.
Ga0247518_108423/758-837       ..............................................................................................................G...A.CAU...........................G.C.G.
Ga0247531_104608/1256-1177     ..............................................................................................................G...A.CAU...........................G.C.G.
Ga0265750_1365815/134-55       .............................................................................................................GA...C.AU............................G.C.G.
Ga0184597_121517/262-183       .............................................................................................................GA...C.AU............................G.C.G.
Ga0265747_102820/448-376       ............................................................................................................................................GAA.G.G.
Ga0247554_105388/388-316       ............................................................................................................................................GAG.G.G.
Ga0307482_1015330/1021-1093    .............................................................................................................................................UC.C.G.
Ga0184600_102725/60-135        .........................................................................................................................................U.GUUG.G.G.
Ga0184602_122974/190-265       ........................................................................................................................................G..GUUG.G.G.
Ga0316038_112263/442-356       ..................................................................................................................G.GCCCG....................GG.G.G.
Ga0307484_161007/136-39        ....................................................................................................................UCCA....................CCA.U.G.
Ga0307482_1033688/516-422      ....................................................................................................GGAG.....UU...A.UCCAC....................CA.U.G.
Ga0316031_132099/178-294       ..........................................................................................U.GG.GUU.GGCUCAU.U.GG...U.GUUUU.................GG.UCAG.G.G.
Ga0213850_1220553/385-463      .........................................................................................................................................G.UGGU.G.G.
Ga0180107_1307618/257-174      .........................................................................................................................................A.GGAG.C.G.
Ga0265747_119090/8-84          .........................................................................................................................................C.AGCA.C.G.
Ga0242646_1115622/191-109      .............................................................................................................CA...C.AAUAAC................................U.G.
Ga0184570_114412/209-338       .....................................................................................................................AUCA.............UUCUGA.AAGA.A.G.
Ga0265757_143567/145-70        ............................................................................................................................................AGC.A.G.
Ga0184568_109812/391-283       ..................................................................................................................A.GUGGA..........................U.A.G.
Ga0184568_104528/922-1000      .............................................................................................................................................GU.A.G.
                               ........................................................................>>>>>>.>>>>>>>>>>>>.>>.>>>.>>>>>>>.>.>>...>.>>>...............>>>>>>.>>>>.>.>.
                               ..................................................................................................................2.222....................2.2222.2.2.
                               ------------------------------------------------------------------------------------------------------------------○-○○○○-------------------○-○●●●-●-G-
```

alignment positions 451 · · · 600

```
Ga0247541_102964/376-269      UGAAGACUA..AGAGAAGAAACAUCAUCAU......................................UUUCACU..........................................................................A
Ga0182091_1018330/100-226     CGAGGCAU...ACUGGUGAAACACUUCGCGUCUGUGGACAC...........................AAUUAA................................................................GUGCAGACAG.C
Ga0316035_118720/195-42       AGAAAGUA...UUGAGGGAAACAUUUAUGUUCG...................................AGUUCCUGAA....................................................................CGAA
Ga0179944_1246019/41-131      CGAAAUGG...CUAAGGGAAACACUAGAAA.ACCCCCU..............................UAAC.....................................................................AGGGAGUGA
Ga0265747_125480/96-202       UGAAGAGG...AUAAGGGAAACAUUUUAUAAGU...................................UGUUGU..................................................................ACUU
Ga0265757_115889/161-280      UGAAGAGG...ACAUGAGAAACAUGCCGA.AAAGAGCUGACCUAC.......................AUU...............................................................GUAGGUGU.CUUUUGA
Ga0180107_1031754/4443-4551   AGAAGACU...GUGGGGGAAACAUUAUACGAGU...................................AACG..........................................................................AC.C
Ga0180107_1229893/426-318     UGAAGACU...GAGAGGGAAACAUCGAAG.......................................CCUAACGAA.........................................................................
Ga0222728_1038816/522-635     AGAAGCAC...AGACAGGAAACACGGCA.........CAAGUA....GGA..................UUCC.........................................................UCCCCAGUACUUGAUUUCCAA
Ga0179955_1195671/481-563     UGAAGCAU...GGUAACGAAACACUCAGACUU....................................UAUUUC.........................................................................AAG
Ga0265751_100560/274-382      GGAAGAUG...UCUGGUGAAACAAUACCAU......................................CUAACGA..........................................................................G
Ga0265751_124015/160-14       UGAAGACAG..AGUGGGGAAACAUUUGUCAGCAUGUCUGCUGUACUGGGGCCGG..............GAAAUCAAA.................................................CCGGA.CCAGUAUGGUGAAUGUGUU
Ga0316029_122532/20-172       AGAAGCCA...GAUGAGGAAACACUGAGGUU.....................................ACA.............................................................................AA
Ga0316031_106349/204-348      AGAAGCCA...GAUGAGGAAACACUGA...UG.GAAG...............................AAUUAA..............................................................CUUCACAA
Ga0265759_136872/100-7        UGAAGUAG...GUCAGGGAAACACUGC.........................................ACAUAA............................................................................
Ga0138291_1027114/229-62      UGAAGAUG...AUCAGGGAAACAUUUCGCAUCA...................................GUAA..........................................................................UGGU
Ga0138291_1177333/356-213     UGAAGACG...AACAGGGAAACAUUUGUAGUGC...................................UUAGU.........................................................................GUGC
Ga0079074_1152161/13-203      UGAAGCAG...GUCAGGGAAACAUGUUAUAAGAAAUAGU.............................UUUUAU............................................................GCUAUUUCUU
Ga0242657_1353977/135-311     GGAAGAAG...UGAGGUGAAACAUAUAGUAU.....................................CUCUUUAA..........................................................................GU
Ga0255293_1180932/373-295     CGAAGCGG...GCGCGUGAAACAUCUC.........................................AUU...............................................................................
Ga0307482_1147486/107-4       GGAAGUUA...CACGGUGAAACACGUAAUC......................................GUAGCGA..........................................................................G
Ga0242647_1001226/1011-1115   UGAGGUCU...CCUGGUGAAACAUUCUGU.......................................AUUGGGCC..........................................................................
Ga0316032_128094/136-241      UGAGGCAU...CAUGGGGAAACACACAGU.......................................CUUGACAC..........................................................................
Ga0184598_118707/299-404      UGAGGCUA...CUUGGGGAAACACGUGGA.......................................CUUGACCC..........................................................................
Ga0213850_1470830/321-202     UGAAGACU...GUCAGGGAAACACAAGGAAGUAUCUUUCAAC..........................CUCA.................................................................GUUGAAU.GUGCU
Ga0307482_1003156/2075-2177   GGAGGACA...GUCGGUGAAACAUCAUGCGU.....................................AACG............................................................................AC
Ga0307482_1000291/592-698     GGAGGACA...GUCGGUGAAACAUCAUACAGU....................................AACG...........................................................................AC.
Ga0247532_117056/43-239       GGAAACCU...CUAGGUGAAACACGCGUGUAG....................................UUAGCGC........................................................................CUG
Ga0184582_120088/374-570      GGAAACCU...CUAGGUGAAACACAUGUGUAG....................................UUAGCGC........................................................................CUA
Ga0265742_1013613/579-453     CGAAGACUC.GACGAAGAAACAUGCGCGGGA.....................................UGCC...........................................................................UCC
Ga0265748_110851/279-405      CGAAGACUC.GACGAAGAAACAUGCGCGGGA.....................................UGCC...........................................................................UCC
Ga0307482_1000262/4292-4390   UGAAGACU...GGGAGGGAAACACAGGGA.......................................CUUAGCGAA.........................................................................
Ga0307484_100453/182-280      UGAAGACU...GGGAGGGAAACACAGGGA.......................................CUUAGCGAC.........................................................................
Ga0247538_120078/87-186       UGAGGAUC...GGGAGGGAAACACGCAGA.......................................AUUAACGAC.........................................................................
Ga0307484_109934/368-484      UGAAGACA...CUAAGGGAAACAUACAAGG.AAACGAG.GAGC.........................CGU.................................................................GCUCACUCUAACAU
Ga0184572_111224/187-319      GGAAGGUA...GUCAGGGAAACAUGUCAUGUCAGGGGAGGUC..........................AACC.................................................................GAUCUUCCGUG.C
Ga0265744_102237/225-365      AGAAGAAGG..AUCGGUGAAACACGACCGC......................................CCGCCGAA.........................................................................G
Ga0265758_100384/785-1040     AGAAGAAGU..AGUGGUGAAACACACCG.ACCUU..................................GGC..........................................................................AAGGC
Ga0265753_1005391/776-521     AGAAGAAGU..AGUGGUGAAACACACCG.ACCUU..................................GGC..........................................................................AAGGC
Ga0265759_100623/780-1036     AGAAGAAGC..AGUGGUGAAACACACCG.ACCUU..................................GGC..........................................................................AAGGC
Ga0247535_113123/229-119      GGAAGCUG...CCUGAAGAAACACUUAUGGU.CAC.CUCUUACCGCU.....................GAA.............................................................AGCGAUGUGAGUGUGCGC
Ga0316036_112404/482-376      GGAAGCUG...CCUGAAGAAACACUUAUGGU.CAC.CCCUGUU.........................GCCACUAAA...........................................................GGCAGGUCGUGCGC
Ga0247515_120448/91-178       GGAAGCUG...CUUGGUGAAACACUUUUGUGUUGGUAUCC............................UUUAA..................................................................GG..ACCCCUA
Ga0247518_120275/202-289      GGAAGCUG...CUUGGUGAAACACUUUUGUGUUCUCGUC.............................CUUGAAA.................................................................GACG..GCUA
Ga0307482_1000311/1469-1363   GGAAGAGU...GAGGGCAAAACACAUGUG.......................................ACUAACGAC.........................................................................
Ga0184603_150346/629-518      GGAAGCACU..GAGGGAGAAACACGUUAG.......................................AUUAGCGAC.........................................................................
Ga0307484_102855/507-659      GGAAACUCUG.CGAAGAGAAACACUCAGUC......................................UUUUGGGC.........................................................................G
Ga0316029_101396/632-732      GGAAGUCU...GGCGGUGAAACACUUAGG.......................................ACUAGCGAC.........................................................................
Ga0184582_100659/424-334      UGAGGACC...GGUGGGGAAACAUGCUCC.......................................UGU...............................................................................
Ga0265754_1040230/51-148      UGAAGACC...GAAGGGGAAACAUGCA.........................................UAUU..............................................................................
Ga0316039_120091/354-252      GGAAGUAG...AUGAGGGAAACACUUACGGUAGU..................................CUCG.........................................................................GCUUC
Ga0307921_1050750/452-358     GGAAGCAA...GUGGAGGAAACAUUAGUAGUUGC..................................GCU..........................................................................GUAAC
Ga0265759_141590/154-258      UGAAGACG...GUUGGUGAAACACGA.CCUGCC...................................CUUUU..........................................................................GGCA
Ga0184602_130775/1275-1431    GGAAGUAU...CCAGGGGAAACAUGCGUAC....UGGCCUCUUG.AC.CUGCU...CGGC........UUAU...............................................GCUAUACAGCAGCGUGCGGGAGUCCAAGGUG
Ga0213850_1052387/326-212     UGAAGACA...CCUGGUGAAACACAGAC..GAGAUCGC.UCGAGUC.......................UAU..............................................................GGCAGGACGAGAUCUCC
Ga0213850_1393213/656-542     UGAAGACA...CCUGGUGAAACACAGAC..GAGAUCGCUCGACUCU.......................AUGGCA............................................................GGAGGA...GAUCUCC
Ga0184578_112042/249-140      GGAAGAUU...AGGAGGGAAACAUCUAGACG.....................................UUGCGU..........................................................................C.
Ga0316033_100036/207-98       GGAAGAUU...AGGAGGGAAACAUCUAGACG.....................................UCGCGU..........................................................................C.
Ga0247534_100007/116-225      GGAAGAUU...AGGAGGGAAACAUCUAGACG.....................................UUGCGU..........................................................................C.
Ga0184569_109084/220-111      GGAAGAUU...AGGAGGGAAACAUCUAGACG.....................................UCGCGU..........................................................................C.
Ga0206355_1134705/241-332     UGAAAACU...GUGAGGGAAACACGCCAACAAUGA.................................UCG.........................................................................UCAUUG
Ga0265754_1007706/448-552     AGAGGGCA...GUCGGUGAAACAUUAUAAC......................................UUAACGC..........................................................................G
Ga0213850_1056422/14-139      GGAAGACA...GGACAAGAAACACCGCGUC.......GCUGGUCAUCC....................AGCGA..........................................................GAAUAGCCAUCGCUUUGAG
Ga0180107_1033011/786-679     GGAAGGCG...UCCGGUGAAACAUCCAUAU.C....................................UUGC...........................................................................GAA
Ga0307483_1000720/1178-1064   CGAAGAGA...GGGAGGGAAACACUCAACAGU....................................GGUC...........................................................................GC.
Ga0265755_100088/1451-1216    GGAAGAACU..UGAAGUGAAACACUGUUGGAGU...................................UAACU..........................................................................GCCC
Ga0310119_125223/193-319      AGAAGAGAUU.GUGAGAGAAACACGAAAU.CAUCCGAUGAUGG..........................CUAGUCA.......................................................CCUUCAGAAAGUGA
Ga0265754_1037910/322-179     GGAAGAGA...CCUGGGGAAACAUGCAUCGCCU...................................AACGC..........................................................................GGGC
Ga0316053_162144/16-125       GGAAGAUCU..GAUAGUGAAACAUUCUCGCUU....................................GACG...........................................................................AA.
Ga0242654_10007932/343-135    GGAAGCAA...UCGAGCAAAACAUGCG.GCGUC...................................GACUGGCG......................................................................GACG
Ga0153880_1315932/142-46      UGAAGACU...AGCGAGGAAACAUGCC.........................................GUUU..............................................................................
Ga0265750_1009277/794-905     UGAAGACG...UCCAGGGAAACAUUUUUAGCACA..................................GUUAA........................................................................UGUGC
Ga0265756_100928/447-557      UGAAGACG...UCCAGGGAAACAUUUUUAGCACA..................................GUAA.........................................................................UGUGC
Ga0265745_1004075/525-659     UGAAGACG...UCCAGGGAAACAUUUUCAU.GCAUUAGC..AUAGCUU....................UAC............................................................AAGCUAACAGUUAGUGCUA
Ga0138291_1147840/370-201     UGAAGAUG...AUCAGGGAAACAUUUCGCAUCA...................................GUAA..........................................................................UGGU
Ga0138291_1027295/231-62      UGAAGAUG...AUCAGGGAAACAUUUCGCAUCA...................................GUAA..........................................................................UGGU
Ga0184602_109114/416-317      UGAAGUGAA..GUCAGGGAAACAUACUCUGUUCCAGC...............................UUGC......................................................................GCUGUAAU
Ga0153880_1354682/355-454     UGAAGUGAA..GUCAGGGAAACAUACUCUGUUCCAGC...............................UCUC......................................................................GCUGUAAU
Ga0242649_1016743/464-237     UGAAGAUU...GUCAGGGAAACACUGUCAGUACA..ACUGAUUGGAAAC...................CAUCGAU.......................................................GUUUUCAGUUGGGAAUGUGC
Ga0179943_1118543/65-150      GGAAGCGG...CUUGGUGAAACAUACAAAGUAG...................................UUUA..........................................................................CUAC
Ga0184568_108419/492-395      AGAGGUCG...GACGGUGAAACACUUAGAAGU....................................AACG...........................................................................AC.
Ga0265756_117960/316-418      UGAAGGACA..GGGAGUGAAACACGGCAAC......................................GUCGCGA..........................................................................G
Ga0265759_100202/139-258      AGAAGUGA...CCAGAGGAAACACACCAGAGC....................................AGCG...........................................................................GUG
Ga0265748_107452/280-387      GGAAGACU...AGGAGGGAAACACCUAUGCGU....................................CGCG...........................................................................AC.
Ga0307484_149222/136-234      GGAAGUAC...ACUGGGGAAACAUGCAAGGG.....................................GUAGCG..........................................................................UC
Ga0316038_107364/55-195       GGAAGCGG...GUCAGUGAAACAUUAGUG.UCGUUCCGG.............................AAAUCAAA................................................................CCGGUAUGCU

                              ....>>>>...<<<<<.......<<<<<<<<<<<<<<<<<<<<<<<<<<<<<<<<<<<<<<<<<<<<<..........................................>>>>>>>>>>>>>>>>>>>>>>>>>>>>>>>>>>>>>>>>
                              ....1222...22222.......2222222..............................1..1100...........................................0011..1..............................2
                              RGAAG●●●---R●●RGRGAAACAY●●●●○○○-------------------------------------●●●○○-----------------------------------------------------------------------------○○
```

```
Ga0184593_123391/157-297      GGAAGCGG...GUCAGUGAAACAUUAGUG.UCGUUCCGG..............................AAAUCACA..............................................................CCGGUAUGCU
Ga0242652_1159268/284-150     AGAAGAUU...GUUGGUGAAACAUGGAUGGCC.....................................UUAGCC......................................................................GAGC
Ga0310117_113093/724-511      GGAAGACCU..AGACGGGAAACACUGGUUAGC.....................................AUAGUCCG.....................................................................GC.
Ga0179944_1119724/70-159      CGAAGCCG...GCCAGGGAAACAUGCUUCUAACA....................................A..........................................................................UGUUG
Ga0316036_104840/706-578      UGAAGACG...CUCAGGGAAACACAAUAGACACC...................................UCA........................................................................GGUGC
Ga0247538_106858/350-156      AGAAGGAAU..GCUGGUGAAACACCACGCGCGU....................................CGCUGA......................................................................ACGC
Ga0180107_1215312/308-184     AGAAGGAA...CUAGGGGAAACACACCAGUCU.....................................GCCG.........................................................................AGA
Ga0247534_100003/3147-3245    GGAAGAUCC..GAUGGUGAAACAUCAUAG........................................CGUUGCGAA.......................................................................
Ga0247519_101377/1717-1907    GGAAGCUC...UGCAGUAAAACAUCUUCAGCG.....................................AUUGGC.......................................................................GGC
Ga0184588_143621/642-452      GGAAGCUC...UGCAGUAAAACAUCUUCAGC......................................GAUUGGCG......................................................................GC
Ga0265758_100017/4028-4134    GGAAGAUA...AUGGGGGAAACAUUUUGG........................................AUUUACGCA.......................................................................
Ga0265751_102572/231-125      GGAAGAUA...AUGGGGGAAACAUUUUGG........................................AUUCACGCA.......................................................................
Ga0310106_100226/1503-1738    GGAAGAACU..UGAAGUGAAACACUGUUGGGG.....................................UUAACUG......................................................................UCC
Ga0184596_112078/314-191      AGAAGUAU...UCCAAGGAAACACACCACUUUGAUCAUCGUACUAU.......................AGCU...........................................................AUAGCACUCACAUUAU.
Ga0247528_108647/438-562      AGAAGUAU...UCCAAGGAAACACACCACUUUGAUCAUCGUGCUAU.......................AGCUA..........................................................AUAGCACUCACAUUA..
Ga0184602_108471/49-173       AGAAGUAU...UCCAAGGAAACACACCACUUUGAUCAUUGUGCUAU.......................AGCUA..........................................................AUAGCACUCACAUUAU.
Ga0316031_112736/216-74       GGAAGCGA...GGAGAAGAAACACGAAAAGAUCAAUGCGCC............................AGUGA...............................................................GGCGCAAGC..C
Ga0184575_106581/174-318      GGAAGCGA...GGAGAAGAAACACGAGGAGGAUUCUGAAGCC...........................AGUGA..............................................................GGCUUCGGC..CU
Ga0184582_129189/472-619      UGAAGAAG...CUAGGUGAAACAUGCGUGACCC....................................UUGGCC......................................................................GGGU
Ga0180052_116799/419-501      GGAAGACU...GACGGUGAAACACUUAGAUA......................................AAA...........................................................................UA
Ga0179955_1013331/439-311     AGAAGACG...GGACAAGAAACACCGCGUAGUUUUGAAA..............................CUAUAAUAGUAUGACUU....................................................UUUC....AUA
Ga0157569_1058012/474-586     GGAAGACC...CUCGGUGAAACACCGCCC........................................UAGG............................................................................
Ga0157562_1164332/672-560     GGAAGACC...CUCGGUGAAACACCGCCC........................................UAGG............................................................................
Ga0265746_1084801/20-158      GGAAGACU...GUCGGUGAAACAAACAAG......GAGUAGAACCAUA..AAGGAG.............UUAC.................................................CUCCAUCAUAUGGUA.UACUUUACCUA
Ga0265752_102479/223-81       GGAAGACU...GUCGGUGAAACAAACAAG......GAGUAGAACCAUAC..AAGGA.............AUAUUUCA.............................................UCCAUCAGUAUGGCA.UACUUUACCUA
Ga0265756_107066/520-660      GGAAGACU...GUCGGUGAAACAAACAAG.GAGUAGA....ACCAUAC..AAGGAU.............UAUC.................................................AUCCAUCAGUAUGGUAUAAUUUACCUA
Ga0247541_102884/37-209       AGAAGAUUC..CCCGGUGAAACACAGC..........................................AUUUAAUGGUCAAC..................................................................
Ga0247516_125012/316-428      AGAAGAAUG..UCCGGUGAAACACAGCCUGGG.....................................AUUAACCUC....................................................................CCC
Ga0213850_1071966/331-222     UGAAAGCA...GCAAGGGAAACAUAUGCACA......................................GUCAA.........................................................................UG
Ga0257141_1100114/291-412     AGAAGACA...CCAGAGGAAACACUCCC..ACGCCA.....CCUUGUA.....................UACAAGC......................................................UGCAAGGCUCGAUGGCGGA
Ga0265740_1020297/298-150     GGAAGACA...AACGGGGAAACAUGCUUU........................................UGA.............................................................................
Ga0307482_1064439/425-316     AGAGGAUUC..GAUGGGGAAACACUUUGGG.......................................CGUAACGAA......................................................................C
Ga0138292_1031932/14-100      AGAAGAUGCA.AAGCGGGAAACACGUAUC.GAACU..................................CUA.......................................................................GGCUCA
Ga0138291_1022124/340-254     AGAAGAUGCA.AAGCGGGAAACACGUAU.CGAACU..................................CUA.......................................................................GGUUCA
Ga0184582_127657/238-384      AGAAGCCUUA.UCUAGAGAAACACAUGAG........................................CGUAACGUA.......................................................................
Ga0184570_113141/244-341      UGAAGACA...GCUAGUGAAACAUAUUAUUCC.....................................UACCUU.......................................................................GGA
Ga0310119_152175/242-389      AGAAGAGCA..GCAAGGGAAACAUGCGUG.....CAUGCAUCGUUACCACAGUGGUG............AAU..................................................CACC....GGUAGCGGAGUAUGAGGCA
Ga0184576_119948/531-664      AGAAGGAUA..UUCGGUGAAACACAUUCGA.......................................CCAGCCGAA......................................................................U
Ga0316034_129624/151-284      AGAAGGAUA..UUCGGUGAAACACAUUCGA.......................................CCAGCCGAA......................................................................U
Ga0307482_1134687/287-181     AGAAGCAU...GGAAGUGAAACACGUCGA........................................AUUAACGAC.......................................................................
Ga0307484_104189/399-292      AGAAGCAU...GGAAGUGAAACACGUCGA........................................AUUAACGAC.......................................................................
Ga0138282_1242808/325-475     GGAAGAUC...GGCAGGGAAACACGCCGCUCAACAUCU...............................CAUC...................................................................GGAUG..GA
Ga0180047_1081041/176-42      UGAAGACG...GAGGGUGAAACACG.CUUGGACC...................................CUUUU......................................................................GGUUC
Ga0265752_100023/1618-1523    GGAAGAUA...GAUGGUGAAACAUCGUAGAGU.....................................UGCG.........................................................................AC.
Ga0242649_1051170/34-143      GGAAGAUG...AGAGGAGAAACAUUUAUAGAGU....................................AACG.........................................................................ACC
Ga0242654_10030584/1049-1243  AGAAGAAGA..UCCGGUGAAACAUGCACUAUCCUG..................................ACC.......................................................................UAGG.U
Ga0307484_102966/727-825      GGAAGACU...AGGAGGGAAACAUUUGCUAUU.....................................AGCG.........................................................................AA.
Ga0307921_1020571/133-221     UGAAGUUC...GUGGGGGAAACAUGUAC.........................................CUUUGA..........................................................................
Ga0184573_113483/125-229      AGAAGAUG...AAUGGUGAAACACGCUAA........................................UAAUAAU.........................................................................
Ga0184576_116834/47-151       AGAAGAUG...AAUGGUGAAACACGCUAA........................................UAAUAAU.........................................................................
Ga0184595_109216/770-611      AGAAGAAGU..UACGGUGAAACAUGCACUGUUU....................................UGGC........................................................................GGAC
Ga0316040_154359/245-85       AGAAGAAGU..UACGGUGAAACAUGCACUGU......................................CUUGGCGA......................................................................AC
Ga0184595_116949/555-395      AGAAGAAGC..UACGGUGAAACAUGCACUGGCU....................................UGGC........................................................................GGUC
Ga0247553_104889/634-563      GGAAGCUU...UAUGGUGAAACAUGCA..........................................UUAU............................................................................
Ga0316046_107255/463-562      AGAAGCGU...GCUGGUGAAACAUCCAC.........................................UUAU............................................................................
Ga0307484_102693/441-627      UGAAGGAU...CCGGAGGAAACACGC.CAGCU.C...................................UGCC.......................................................................GAAGC
Ga0184568_114257/261-413      AGAAGAAGC..GAUGGAGAAACAUGCCAGC.......................................CUGCCGAA.......................................................................G
Ga0316047_106478/13-108       AGAAGUGG...GUAGGGGAAACCUUGGGCAU......................................AGACUA........................................................................AU
Ga0242654_10024002/775-526    UGAAGACU...GCGAGGGAAACAUGCCGCUCCGCUGGAAGAAC..........................UGUU..............................................................GUUCUUA....GGA
Ga0316039_101269/650-841      UGAAGACA...GAGAGGGAAACACAAGGCUU......................................CAGCGUA.......................................................................GG
Ga0265743_102316/615-423      UGAAGACA...GAGAGGGAAACACAAGGCCU......................................CAGCGUA.......................................................................GG
Ga0210323_1072445/691-543     CGAAGGAUU..GUCGGUGAAACACCGACAGCC.....................................CUGGCUA......................................................................GGC
Ga0224712_10069322/1099-1357  GGAAAAUG...GUGAGGGAAACACUAGG.........................................AAACC...........................................................................
Ga0213850_1371453/186-298     GGAAGACA...CCUGGGGAAACACUCCU.GGCCAUAAGCUACCUG........................AGUCACA...........................................................CAGG.....AUCGCU
Ga0153880_1056505/290-430     UGAAGGCA...GUCAGGGAAACAUGCAAAAGCAUAU.................................UGUUAU...................................................................AUGUGCU
Ga0179940_1033563/296-410     AGAAAGCG...GUAAGGGAAACAUGCCA..GAAUG..................................AAAAA.....................................................................CAUUCC
Ga0265743_143797/241-325      GGAAGACA...GCUGGUGAAACACCGAC.........................................GGCGAA..........................................................................
Ga0242654_10006748/25-247     UGAAGCGCG..AAGAGGGAAACACACUCCUUC.....................................GAAUGUCG.....................................................................GGG
Ga0138278_1149859/65-166      AGAAGCUG...GUGGGCAAAACAUAUCCUG.UGUCUCGCGA............................UCA.................................................................UCGCGAU.CAUC
Ga0138278_1149142/191-91      AGAAGCUG...GUGGGCAAAACAUAUCCUG.UGUCUCGCGA............................UCA.................................................................UCGCGAU.CAUC
Ga0138277_1147417/463-362     AGAAGCUG...GUGGGCAAAACAUAUCCUG.UGCCUCGCGA............................UCA.................................................................UCGCGAU.CAUC
Ga0138278_1065026/314-214     AGAAGCUG...GUGGGCAAAACAUAUCCUG.UGCCUCGCGA............................UCA.................................................................UCGCGAU.CAUC
Ga0138277_1089473/408-304     AGAAGCUG...GUGGGCAAAACAUAUCAAUUGUGUCUCGCAA...........................UCA................................................................UUGCGAU.CAUCG
Ga0138277_1022592/399-305     AGAAGCUG...GUGGGCAAAACAUGUCAAUUAU....................................UCAUUAAUUU..................................................................GUUU
Ga0157559_1099797/360-209     GGAAAACCU..ACCAGGGAAACAUUCAAUGAAG....................................AAGCAC......................................................................CUCC
Ga0157572_1089974/360-209     GGAAAACCU..ACCAGGGAAACAUUCAAUGAAG....................................CAGCAC......................................................................CUCC
Ga0242646_1007472/516-317     AGAAGGAUU..CCCGGUGAAACAUGCGUACGC.....................................AGGCCA.......................................................................GCC
Ga0184583_104740/342-477      AGAAGGAA...GUCGGUGAAACAUGCUCGC.......................................CCGCCGAA.......................................................................G
Ga0242654_11312609/202-83     GGAAGUUU...GGCGGUGAAACACAUUGCAGAGCACUCAACAAUC........................AUUUCAA..........................................................GGUUG.UGAGGA...U
Ga0184568_109070/428-286      UGAAGAAU...CCAGGGGAAACACACCGUCC......................................AGCCGA........................................................................GG
Ga0224712_10002011/1564-1454  GGAAGACU...AGGAGGGAAACACAUCAGCGU.....................................AACG.........................................................................AC.
Ga0222728_1000095/4027-4133   AGAAGAAAU..GGAGGGGAAACAUAUCAG........................................CUUAGCGAC.......................................................................
Ga0307482_1068632/529-339     GGAAGAUU...GCAGGUGAAACAUCUCUAUGGUCUGAUCCC..GGGUCCUAGACCCGUGCU........GUACCGCAUUACUCGGCAUGUAUUACUACAUGACUUGU..........AACGAGGG..UGCACGAGAAAAGACAAGGGCA
Ga0157560_1170105/128-245     GGAAGCCG...GUGAGGGAAACCUCUAGAUAGUGGACACGUC...........................CGGAGU.............................................................GACGUAUCCUAA.
Ga0316031_107021/319-214      AGAAGAUAC..GGGAGGGAAACAUCUUGA........................................ACUAGCGAC.......................................................................
Ga0316047_100015/734-626      AGAAGAAAU..GAGAGGGAAACACAGUAGA.......................................AGUUGCGA.......................................................................U
Ga0247533_100162/1643-1535    AGAAGAAAU..GAGAGGGAAACACAGUAG........................................AAGUGGCGCA......................................................................
Ga0180108_1209503/1932-2029   GGAAGACC...GAAGGGGAAACACAACUG........................................AUUCGCGAC.......................................................................
Ga0265753_1009881/576-819     UGAAGAAU...CCAGGGGAAACACACCGUCC......................................AGCCGA........................................................................GG

                              ....>>>>...<<<<<.......<<<<<<<<<<<<<<<<<<<<<<<<<<<<<<<<<<<<<<<<<<<<<<........................................>>>>>>>>>>>>>>>>>>>>>>>>>>>>>>>>>>>>>>>>
                              ....1222...22222.......2222222..............................1..1100..........................................0011..1..............................2
                              RGAAG●●●---R●●RGRGAAACAY●●●●○○○--------------------------------------●●●●○-------------------------------------------------------------------------○○
```

```
Ga0265741_101522/997-754 UGAAGAAU...CCAGGGGAAACACACCGUCC......................................AGCCGA.........................................................................GG
Ga0184583_103998/587-740 AGAAGAAUA..GUCGGGGAAACAUGCUAGCC......................................AGCCAA.........................................................................GG
Ga0247521_125910/353-214 UGAAGUCA...GUGAGGGAAACACUGGA...GAAGG.................................UUAUCAC...................................................................CCUUCAA
Ga0184569_106253/218-80 UGAAGUCA...GUGAGGGAAACACUGGA..GAAGG..................................UUAUCAC....................................................................CCUUCA
Ga0316039_119781/347-457 UGAAGAGAC..GGGAGGGAAACAAGAUGA........................................ACUAGCGAC........................................................................
Ga0184572_112077/359-259 GGAAGAGA...ACUGGUGAAACAUCAUGAA.......................................GUAGCGA.........................................................................U
Ga0257141_1118781/153-31 AGAAGACA...CCAGAGGAAACACUCUCCAAGCUAGUAAAGAGUGAGCU....................GCGA.........................................................AGCUCACU.....AUAGCGA
Ga0257136_1059838/549-429 AGAAGACA...CCAGAGGAAACACUCUCCAAGCUAGUAAAGAGUGAGCU....................GCGA.........................................................AGCUCACU.....AUAGCGA
Ga0138283_1149501/173-59 UGAAGUCG...GCCGGUGAAACACGCGGCGCGCGC..................................AAACG......................................................................GCGCG.
Ga0184597_126105/545-362 AGAAGAAC...UCCAGGGAAACACUCUUAU.......................................GUCUCGGUG.......................................................................A
Ga0213850_1245299/581-409 AGAAGUCA...GUCAGGGAAACACGCGGG........................................AUACGAAACC.......................................................................
Ga0179948_1254202/198-296 GGAAGACA...GCUAGCAAAACACCGCGUUUAAUAUGUUUA............................UUUGGUU..............................................................UAAGC.......
Ga0180108_1093556/523-685 GGAAGCGAG..GAGAAGGAAACACACCUCGC......................................CCGCCGAA.......................................................................GC
Ga0180107_1321405/371-533 GGAAGCGAG..GAGAAGGAAACACGCCUCGC......................................CCGCCGAA.......................................................................GC
Ga0242641_1001197/544-746 AGAAGAAGA..ACCGGUGAAACACACCUCCCC.....................................CGCCGA........................................................................GGC
Ga0184597_115944/155-80 AGAAGAUG...GGUAGGGAAACAUAGCU.........................................GAUCA............................................................................
Ga0184595_100702/414-262 AGAAGAAA...GUCGGGGAAACAUGCUAGCC......................................AGCCGA.........................................................................GG
Ga0184601_118759/683-531 AGAAGAAA...GUCGGGGAAACAUGCUAGCC......................................AGCCAA.........................................................................GG
Ga0184592_125105/67-219 AGAAGAAA...GUCGGGGAAACAUGCUAGCC......................................AGCCAA.........................................................................GG
Ga0257141_1089020/433-224 UGAAGAUG...ACCAGGGAAACAUGACGUACA.....................................GUAA..........................................................................UGU
Ga0265743_103007/262-368 UGAGGCAU...CCCAGGGAAACAUGCAUCAUU.....................................GAC...........................................................................GAU
Ga0222728_1000095/4541-4435 UGAGGACA...UCCAGGGAAACAUGCAGG........................................GAUGACAU.........................................................................
Ga0247537_109082/42-148 UGAGGAUA...UCCAGGGAAACAUAUAGGGA......................................UGAC...........................................................................UU
Ga0247533_100162/1117-1223 UGAGGAUA...UCCAGGGAAACAUAUAGG........................................GAUGACAU.........................................................................
Ga0265757_100063/1489-1724 GGAAGAACU..UGAAGCGAAACACUGUUGGG......................................UUUAACUGU......................................................................CC
Ga0265747_118257/34-269 GGAAGAACU..UGAAGCGAAACACUGUUGGG......................................UUUAACUGU......................................................................CC
Ga0265745_1000046/1698-1933 GGAAGAACU..UGAAGCGAAACACUGUUGGGG.....................................UUAACUG.......................................................................UCC
Ga0115595_1153837/320-197 GGAAGACCA..GUCGGUGAAACAUCCAUAGACGGGAAAAGGCCUGUGC.....................ACA...........................................................GCACAUGUC....CCC..C
Ga0242657_1026087/460-322 AGAAGGAUU..GCUGGUGAAACAUGGCACGGU.....................................CGCUA.........................................................................ACC
Ga0265751_100417/1480-1588 GGAAGCCA...GGCGGUGAAACACCGGAUUGUU....................................GAC..........................................................................AGCA
Ga0265756_100485/1108-1000 GGAAGCCA...GGCGGUGAAACACCGGAUUGUU....................................GAC..........................................................................AGCA
Ga0265751_100393/1594-1698 AGAAGAAAU..GGAGAGGAAACAUACCAG........................................AUUAGCGAC........................................................................
Ga0307921_1011071/674-791 UGAAGUCA...GCGAGGGAAACAUGCAAAAGCAC...................................GUCCAU.......................................................................GUGCU
Ga0307919_1094420/160-285 UGAAGUCU...GUGAGGGAAACAUGCAAAAGCAC...................................GUCCAU.......................................................................GUGCU
Ga0316034_107719/314-205 GGAAGAUC...GGGAGUGAAACAUGUCAA........................................CUUAGCGAA........................................................................
Ga0265742_1065119/361-273 GGAAACAUA..GCUGCAGAAACAUGCCCGAAU.....................................CUA...........................................................................AUU
Ga0180107_1164033/560-431 AGAAGACU...UCUAGUGAAACACGCCU.........................................UAA..............................................................................
Ga0242641_1001148/1456-1256 AGAAGAUC...UCCGGUGAAACACGGCUG........................................ACAAUGGCA........................................................................
Ga0307482_1009758/562-669 AGAAGAUCU..GGGAGGGAAACAUGUCAG........................................CGUAGCGAA........................................................................
Ga0247527_109126/592-695 AGAAGCAAA..GUCAGUGAAACAUCCAGA.CGUCGUCGAAUCG..........................CUUG...............................................................CG...CGACCUCGU
Ga0247519_111615/148-45 AGAAGCAAA..GUCAGUGAAACAUCCAGA.CGUCGUCGAAUCG..........................CUUG...............................................................CG...CGACCUCGU
Ga0247513_116034/391-494 AGAAGCAAA..GUCAGUGAAACAUCCAGA.CGUCGUCGAAUCG..........................CUUG...............................................................CG...CGACCUAGU
Ga0213850_1290372/434-275 GGAAGUGU...GGUGGCGAAACACGC.CAAGG...UGUG.GUACCU.......................UUUU............................................................AGGGGCACAUAAAUCCU
Ga0138276_1121602/329-410 AGAAGCAG...GCAGGCAAAACAUGGCCG........................................AUUAAC.....................................................................
Ga0138278_1041650/183-102 AGAAGCAG...GCAGGCAAAACAUGGCC.........................................CGUUUAAA.........................................................................
Ga0138277_1137481/570-489 AGAAGCAG...GCAGGCAAAACAUGGCC.........................................CGUCGAAA.........................................................................
Ga0138278_1041788/293-378 AGAAGCAG...GCAGGCAAAACAUGGCCA........................................CCAAUAAAAA.......................................................................
Ga0138277_1142657/273-361 AGAAGCAG...GCAGGCAAAACAUGGCCA........................................CCAAUUUCAAAAA....................................................................
Ga0138275_1223952/371-283 AGAAGCAG...GCAGGCAAAACAUGGCCA........................................CCAAUCUCAAAAA....................................................................
Ga0213850_1450246/199-116 GGAAACCUAG.GAGAGAGAAACAUGACGCG.......................................GGCCGGG.........................................................................C
Ga0213850_1307525/101-186 GGAAGCCUAG.GAGAGAGAAACAUAACGUC.......................................GAGCCAGAAAA.....................................................................G
Ga0265756_118952/425-318 GGAAGCUC...GCCGGCAAAACACCAUGGUGUG....................................UAAGCAUAU...................................................................UACA
Ga0180107_1244437/408-507 AGAAGAUCU..GAGGGUGAAACAUGAUGA........................................AUUAACGAC........................................................................
Ga0265742_1051722/222-295 AGAAGCAC...GGAAGGGAAACACGGAU.........................................GAUCA............................................................................
Ga0242657_1051800/416-321 AGAAGUUG...CAUGGGGAAACAUACAGAAGU.....................................AGCG..........................................................................AC.
Ga0307484_102966/353-250 GGAAGUUA...UCUGGGGAAACACAUGGA........................................AUUAACGAC........................................................................
Ga0184568_108419/50-147 GGAAGACU...GAGAGGGAAACAUCUGUUA.......................................GUAGCGA.........................................................................U
Ga0184568_103677/484-581 UGAAGACC...GAGAGGGAAACAUCUGUUA.......................................GUAGCGA.........................................................................U
Ga0307484_102709/562-403 AGAAGAGA...GAUGGUGAAACAUGCCUGCGGCC...................................GGGC........................................................................GGCCG
Ga0127503_10678853/419-333 GGAAGGCA...GUGAGGGAAACACUGUUCGC......................................UUCAUAU........................................................................GC
Ga0307919_1065410/76-162 GGAAGGCA...GUGAGGGAAACACUGUUCGC......................................UUCAUAU........................................................................GC
Ga0242652_1038498/365-470 AGAAGAUUA..GAGAGGGAAACACUGCACUGG.....................................UUCGAAU.......................................................................CCA
Ga0307484_101264/723-941 AGAAGAAGU..GUUGGUGAAACACGCCUGC.......................................CCGCCGAA........................................................................G
Ga0247534_101560/228-122 AGAAAACC...GACGGUGAAACACAGUG..GC.....................................UUAGC.........................................................................GCA
Ga0307484_109931/110-215 UGAGGCAU...GCUAGGGAAACACUCUAC........................................CUUCAGAA.........................................................................
Ga0184595_116681/414-266 GGAAGAACG..GUCGGUGAAACAUGCUCGC.......................................CCGCCGAA........................................................................G
Ga0265749_100155/962-1187 AGAAGAAU...GUCGGUGAAACAUGCACUGC.C....................................UUUGCCU......................................................................GCGC
Ga0310115_109341/244-400 AGAAGAAU...AUCGGUGAAACAUGCACUGC.C....................................UUUGCCU......................................................................GCGC
Ga0310111_106996/679-905 AGAAGAAU...AUCGGUGAAACAUGCACUGC.C....................................UUUGCCU......................................................................GCGC
Ga0265756_101315/275-501 AGAAGAAU...AUCGGUGAAACAUGCACUGC.C....................................UUUGCCU......................................................................GCGC
Ga0265751_100874/820-594 AGAAGAAU...AUCGGUGAAACAUGCACUGC.C....................................UUUGCCU......................................................................GCGC
Ga0265744_120119/40-266 AGAAGAAU...AUCGGUGAAACAUGCACUGC.C....................................UUUGCCU......................................................................GCGC
Ga0310102_105010/226-452 AGAAGAAU...AUCGGUGAAACAUGCACUGC.C....................................UUUGCCU......................................................................GCGC
Ga0247534_103639/475-250 AGAAGAAU...AUCGGUGAAACAUGCACUGC.C....................................UUUGCCU......................................................................GCGC
Ga0265741_101833/434-208 AGAAGAAU...AUCGGUGAAACAUGCACUGC.C....................................UUUGCCU......................................................................GCGC
Ga0242647_1002114/801-1026 AGAAGAAU...GUCGGUGAAACAUGCACUGCCGU...................................UGCCU.......................................................................GC.GC
Ga0265752_104585/383-158 AGAAGAAU...GUCGGUGAAACAUGCACUGC.C....................................UUUGCCU......................................................................GCGC
Ga0310107_109330/1076-850 AGAAGAAU...AUCGGUGAAACAUGCACUGC.C....................................UUUGCCU......................................................................GCGC
Ga0115595_1051440/308-419 GGAAGUCA...GGAAGGGAAACAUACGGUGAUGCCCAUU..............................GGUGA..................................................................AAAAGACA.C
Ga0115594_1054328/389-276 GGAAGUCA...GGAAGGGAAACAUACGGUGAUGCUC.................................AAUGGUUGAAAAA.............................................................GA.CA.C
Ga0307482_1048753/383-528 AGAAGGACU..GACGGUGAAACAUGCUCGC.......................................CCGCCGAA........................................................................G
Ga0307480_1006257/684-599 GGAAGCCAU..GCUAGAGAAACACUGCAC........................................CCA..............................................................................
Ga0184595_116802/212-325 GGAAGCUA...GUCGGUGAAACACCAGUAACUCGCGU....GGGA........................GAGA............................................................UCCCUGAAAAGGCGAAA
Ga0307919_1071894/312-31 GGAAGAAG...GUGAGGGAAACACGUAG.........................................CGUG.............................................................................
Ga0307921_1036773/354-73 GGAAGAAG...GUGAGGGAAACACGUAG.........................................CGUG.............................................................................
Ga0307482_1023665/889-1191 GGAAGAUG...GUGGGGGAAACACGGCGUG.AACC..................................ACUA.......................................................................GGUGGC
Ga0180108_1022680/3902-3998 GGAAGACU...GUAGGUGAAACACAUCUG........................................CGUUGCGAA........................................................................
Ga0184599_137402/105-300 GGAAGACC...GUCGGUGAAACAUACCA.........................................CUU..............................................................................
Ga0157569_1080558/455-605 AGAAGUCGU..GGCAGUGAAACAUGGUACAAUAUUGU..GUUUGA........................GAUUCAAUUUCAA....................................................UCAAACAUACAAUA..

....>>>>...<<<<<.......<<<<<<<<<<<<<<<<<<<<<<<<<<<<<<<<<<<<<<<<<<<<<<.........................................>>>>>>>>>>>>>>>>>>>>>>>>>>>>>>>>>>>>>>>>
....1222...22222.......2222222..............................1..1100............................................0011..1..............................2
RGAAG●●●---R●●RGRGAAACAY●●●●○○○--------------------------------------●●●○○--------------------------------------------------------------------------○○
```

```
Ga0184601_113231/247-149      CGAAGACA...GCCGGUGAAACACACGAG........................................GGCGA............................................................................
Ga0242652_1002960/413-574     AGAAGAGU...CCUGGUGAAACACCGUGC.....UGGAGCU............................GUUAC................................................................GGCUCCGUGGGU
Ga0307484_101303/704-866      AGAAGAAU...CCUGGUGAAACACCGUGC.....UGGAGCU............................GUUAC................................................................GGCUCCAUGGGU
Ga0242641_1025149/251-89      AGAAGAAU...CCUGGUGAAACACCGUGC.....UGGAGCU............................GUUAC................................................................GGCUCCGUGGGU
Ga0307482_1023050/594-496     GGAAGAGC...GGUGGUGAAACAUCAUAGAG......................................UCGCGU.........................................................................C.
Ga0307484_100434/1330-1210    GGAAGACA...GUAGGGGAAACACUCACGA........CUCU...........................AUAAA...............................................................AGAGGACCGGAUU
Ga0153880_1432604/289-395     GGAAGACC...GCGGGGGAAACACAGACAUGA.UC..................................AUUGC......................................................................GACUCA
Ga0157559_1128929/201-87      AGAAGACU...GUAGGUGAAACACCGCGUGCUGAAAUGAUAAGCAUAAUU...................CUUA........................................................AAUUAAUUUUAA..UU..UGA
Ga0157560_1113202/155-269     AGAAGACU...GUAGGUGAAACACCGCGUGCUGAAAUGAUAAGCAUAAUU...................CUUA........................................................AAUUAAUUUUAA..UU..UGA
Ga0157566_1012736/497-384     AGAAGACU...GUAGGUGAAACACCGCGUGCUGAAAUGAUAAGCAUAAUU...................CUUA........................................................AAUUAAUUUUAA..UU..UGA
Ga0157579_1155702/323-436     AGAAGACU...GUAGGUGAAACACCGCGUGCUGAAAUGAUAAGCAUAAUU...................CUUA........................................................AAUUAAUUUUAA..UU..UGA
Ga0157562_1157806/189-74      AGAAGACU...GUAGGUGAAACACCGCGUGCUGAAAUGAUAAGCAUAAUU...................CUUA........................................................AAUUAGUAUUCUGAUU..UGA
Ga0180047_1032966/152-37      AGAAGACU...GUAGGUGAAACACCGCGUGAUGAAAUGAUAAGCAUAAUU...................CUUA........................................................AAUUAGUAUUCUGAUU..UGA
Ga0307484_166952/62-163       CGAAGACCU..GGGAGAGAAACACGCUGAU.......................................CUAGCGA.........................................................................G
Ga0005851_1178990/477-379     GGAAGACG...GUGAGGGAAACACAUCC.........................................UGAGU............................................................................
Ga0153880_1191383/455-340     GGAAGAUCA..GAGAGGGAAACAUGCUGUUAAAGAUA.................................CAACCU...................................................................UAACUUUU
Ga0213850_1282564/267-350     AGAGGCUC...GUCGGUGAAACACGUCGU........................................UGCG.............................................................................
Ga0315817_133437/498-413      AGAGGCUC...GUCGGUGAAACACGUCGUU.......................................UUGC............................................................................A
Ga0315826_123252/262-347      AGAGGCUC...GUCGGUGAAACACGUCGUU.......................................UUGC............................................................................A
Ga0242657_1008904/1267-1104   AGAAGAUG...AUCGGUGAAACACACCUGCUGAGUAACUAACG....ACGUUUGUG.............UAAACAUU..............................................CACAUCCGUAUCACGUUAGCCAAGGAG
Ga0222728_1009902/1182-968    GGAAGCUG...GUGAGGGAAACACGUUGACUA.....................................AACGC.........................................................................UAG
Ga0115594_1106220/441-334     GGAAGACC...GCCGAGGAAACAUGCUGAGAGACAAACGCU..............................AGGCA.................................................................AGCG.....UC
Ga0180107_1307934/226-315     GGAAGAAA...AUCGGGGAAACACACCUUGCA.....................................UCAUC.........................................................................UGC
Ga0184577_115871/202-369      GGAAGGAAU..CCGAAGGAAACAUUGCCGUC......................................CCGCCGA........................................................................GG
Ga0206356_11590518/224-68     GGAAGUCC...GCAAGGGAAACACUCCAU....CAUAGCCG..GUACCGC...AUGAAC..........CAUCCA.............................................GUUCAUAAAGCUAUACUGCGGCUAUGUUCU
Ga0222728_1062510/166-66      AGAAGCAG...GUGAGGGAAACAUCUCA.........................................UAA...............................................................................
Ga0316029_101202/440-551      AGAAGAUA...GCCAGGGAAACAUCGUG.........................................ACUAGCGAC........................................................................
Ga0307482_1017422/1387-1206   AGAAGACU...CCCAGGGAAACACGUGAU.GCU....................................AUG..........................................................................GGUA
Ga0307484_100889/412-594      AGAAGACU...CCCAGGGAAACACGUGAU.GCU....................................AUG..........................................................................GGUA
Ga0255062_11230321/200-110    UGAAGAUA...GCCAGGGAAACAUACCCGGAA.....................................CGAA..........................................................................UUC
Ga0255059_10691632/166-76     UGAAGAUA...GCCAGGGAAACAUACCCGGAA.....................................CGAA..........................................................................UUC
Ga0265744_100073/1235-1401    GGAAGACA...UCCAGGGAAACAUCCUGUUGC.....................................GUG...........................................................................GUC
Ga0265757_113703/332-416      GGAAGAAA...CCAAGGGAAACAUGUUG.CGAGA...................................GAGUUUCA....................................................................UCUCG
Ga0265756_158829/72-188       GGAAGUCA...UCCAGUAAAACACGUUUUAAUUCA..................................UACUCACU...................................................................UAGAUU
Ga0184597_100195/107-221      CGAAGUCA...GACAGGAAAACACACCAUG.......................................AGCUCG..........................................................................U
Ga0184594_121503/721-607      CGAAGUCA...GACAGGGAAACACACCAUG.......................................AGCUCG..........................................................................U
Ga0184582_121032/1005-1119    CGAAGUCA...GACAGGAAAACACACCAUG.......................................AGCUCG..........................................................................U
Ga0184602_104873/671-785      CGAAGUCA...GACAGGGAAACACACCAUG.......................................AGCUCG..........................................................................U
Ga0184589_132372/822-708      CGAAGUCA...GACAGGGAAACACACCAUG.......................................AGCUCG..........................................................................U
Ga0184580_125558/64-178       CGAAGUCA...GACAGGGAAACACACCAUG.......................................AGCUCG..........................................................................U
Ga0184596_101726/395-281      CGAAAUCA...GACAGGGAAACACACCAUG.......................................AGCUCG..........................................................................U
Ga0247541_109256/178-292      CGAAGUCA...GAUAGGGAAACACACCAUG.......................................AGCUCG..........................................................................U
Ga0184584_108444/253-366      CGAAGUCA...GACAGGAAAACACGGCUC........................................AAGCCCGA.........................................................................
Ga0184582_124913/742-629      CGAAGUCA...GACAGGAAAACACGGCUC........................................AAGCCCGA.........................................................................
Ga0184600_135218/318-205      CGAAGUCA...GACAGGAAAACACGGCUC........................................AAGCCCGA.........................................................................
Ga0247541_100074/611-498      CGAAGUCA...GACAGGAAAACACGGCUC........................................AAACCCGA.........................................................................
Ga0247551_101122/731-844      CGAAGUCA...GACAGGAAAACACGGCUC........................................AAACCCGA.........................................................................
Ga0247526_101949/462-575      CGAAGUCA...GGCAGGAAAACACGGCUC........................................AAACCCGA.........................................................................
Ga0247524_101842/357-244      CGAAGUCA...GACAGGAAAACACGGCUC........................................AAACCCGA.........................................................................
Ga0247519_108765/580-693      CGAAGUCA...GACAGGAAAACACGGCUC........................................AAGCCCGA.........................................................................
Ga0180107_1134582/382-542     GGAAGACA...CGCAUGGAAACACUGUGGG.......................................AUUUGACC........................................................................C
Ga0265747_125301/405-306      GGAAGACG...UCCAAGGAAACAUACAUACACUUC..................................AAUAGUCAUA.................................................................GAAGUA
Ga0157593_1101573/244-147     GGAAGUCA...GGUGGUGAAACAUCUCAGUGGUGCAUG...............................GCGGU...................................................................CGAGCACA.
Ga0184599_121568/40-155       GGAAGUCA...GGUGGUGAAACAUCACAUUUUAUGGAGUCAUAAGAAGG....................ACUU.........................................................CCUU....UGACUCUCACUC
Ga0316040_116783/379-494      GGAAGUCA...GGUGGUGAAACAUCACAAUUUGUG.GAGUUUAAAGAAGG...................ACUU........................................................CCUUC....AACUCACACUC.
Ga0157560_1091679/440-538     GGAAGUCA...GUGGGUGAAACAGGGGGCGUUGGUC..................................AAUGACAAA.................................................................GACCAAC
Ga0222728_1165579/392-259     UGAAGCCA...GUCAGGGAAACAACAAUCGUUUAGUUU................................UCAA....................................................................AAAC..GAC
Ga0224712_10601975/393-502    GGAAGACA...GGGAGGGAAACAUUUCAGU.......................................GUAGCGA.........................................................................A
Ga0224712_10010317/1973-2082  GGAAGACA...GGGAGGGAAACAUUUCAG........................................CCUAGCGAA........................................................................
Ga0307482_1048660/422-625     AGAGGAAG...GUCAGGGAAACAUGCUGGC.......................................CGGCCGAA........................................................................G
Ga0179934_1155244/114-26      GGAAAUCC...GUGAGGGAAACAUGC.CGUUAGAAUG.................................AAAAA....................................................................CAUUCUAU
Ga0307484_101172/602-495      GGAAACACC..GGAAGGGAAACACUUGAGGUCU....................................GACA.........................................................................AG.C
Ga0242654_10068518/113-218    GGAAACACC..GGAAGGGAAACACUUGAGGU......................................CGACAA.........................................................................GC
Ga0242647_1002406/915-1021    GGAAACACC..GGAAGGGAAACACUUGAGGUCU....................................GACA.........................................................................AG.C
Ga0222729_1010500/11-117      GGAAACACC..GGAAGGGAAACACUUGAGGUCU....................................GACA.........................................................................AG.C
Ga0307482_1202605/45-154      GGAAGAAAU..GGAAGGGAAACAUGGAGA........................................CUGCGAA..........................................................................
Ga0184603_139562/141-280      GGAAGAUC...GCGGAGGAAACAUGAUUUGUUCCUGCC.CCCCCGAACCUCAAUCCAAGCCUU......AUUCA..........................................AGGGCCCAGA..GAGG...GGAUUAGGUGUGAGC
Ga0242646_1001122/826-995     AGAAGAUUC..UCCGGGGAAACAUGCCCAC.......................................UGAGGU..........................................................................G
Ga0310107_144222/327-436      GGAAGAUA...UCCGGUGAAACACCGUCG........................................CUUAACGAC........................................................................
Ga0265751_124807/392-283      GGAAGAUA...UCCGGUGAAACACCGUCG........................................CUUAACGAC........................................................................
Ga0184572_105168/388-281      AGAAGAAC...GUUGGGGAAACACAUUGG........................................CCUUGCGCU........................................................................
Ga0316036_127178/124-231      AGAAGAAC...GUUGGGGAAACACAUUGG........................................UCUUGCGCU........................................................................
Ga0222728_1003482/1262-1107   AGAAGAACU..GUGAGUGAAACACGAUCGC.......................................CUUAACGGCA......................................................................G
Ga0265743_100639/33-191       AGAAGCAAA..UCCAGGGAAACAUACUGU...CUAUCACC...UGCCUUGUACGUGUUCGGG.......UGGUCU..........................................CCC..AC.CGUACUAGGCACAUGGAGCUAGGCA
Ga0307484_105374/518-723      AGAAGAGCU..UUCGGUGAAACACGCUGUG.....CUGUCA.AAU......AACGC..AUGG.......GAUU............................................CCAGAGGCGUUUACCUAGUUAUGACAGGUGGUC
Ga0265742_1015608/410-491     GGAAGAUC...GCGCAGGAAACAUGCAG..CC.....................................AUAGA.........................................................................GGA
Ga0316030_102139/629-482      CGAAGAAC...GGAAGGGAAACACAGAGUCCA....GGAAGCCAAGGCA....................GACUGGUAGU...................................................CGCUUGCG...AUUAGACGA
Ga0265749_106268/131-212      GGAAGCAC...GUGAGGGAAACACCGGCC........................................CGUC.............................................................................
Ga0184573_100752/140-46       GGAGGAUU...CCCAGGGAAACCGCAAUC........................................ACAU.............................................................................
Ga0157560_1010344/357-456     GGAAGUAA...AUCGGUGAAACACACACAAC..CUCUCAAAGC..........................UUAC...............................................................GC..UGAGAAUCGU
Ga0157593_1006201/239-338     GGAAGUAA...AUCGGUGAAACACACAUAAC..CUCUCAAAGC..........................UUAC...............................................................GC..UGAGAAUCGU
Ga0180047_1087048/281-180     GGAAGUAA...AUCGGUGAAACACGCACAAC..CUCUCAAUCUC.........................AAUU..............................................................GAGA..GAGAAUCGU
Ga0157565_1016893/233-334     GGAAGUAA...AUCGGUGAAACACGCACAAC..CUCUCGAUCUC.........................AAUU..............................................................GAGA..GAGAAUCGU
Ga0184568_100511/100-195      GGAAGACC...GGCAGGGAAACAUCG.CAGCGU....................................AGC..........................................................................GCAC
Ga0265759_114856/370-262      GGAAGACU...GUCAGGGAAACAUGAGAG...AGAGAUAUGGGCC........................GAGG.............................................................GGUCCAUAUCAAUGAC
Ga0222729_1080556/362-213     GGAAGAUU...ACGAGGGAAACACGACACGAGCCAAGGAAUCCUAUUAUUG..................GACCUUGAAUCUACUCACAAGCUAUG.................................CUGUGGUGAGAUG.AUGUGCUC
Ga0247541_102707/328-184      GGAAGAGG...GUCAGGGAAACAUAGCGUGUGC....................................CAAAAUCU.....................................................................GUGC
Ga0307921_1020694/406-483     GGAAAUGG...GUGAGGGAAACACUUCAU........................................UAUAU............................................................................
Ga0307919_1034281/527-451     GGAAAUGG...GUGAGGGAAACACUUCAU........................................UAUAU............................................................................

                              ....>>>>...<<<<<.......<<<<<<<<<<<<<<<<<<<<<<<<<<<<<<<<<<<<<<<<<<<<<<............................................>>>>>>>>>>>>>>>>>>>>>>>>>>>>>>>>>>>>>
                              ....1222...22222.......2222222..............................1..1100..............................................,..0011..1...........................2
                              RGAAG●●●---R●●RGRGAAACAY●●●●○○○--------------------------------------●●●●○--------------------------------------------------------------------------○○
```

```
Ga0184568_108869/934-1072      AGAAGAAGG..AUCGGUGAAACACGCUCUUCC.....................................CCGGUAA.......................................................................GGA
Ga0206356_10036221/150-306     GGAAGACA...GUCGGUGAAACACAUACACCUUAUG.................GUGGGUGG........AAAAGACAUUUUAUCUUAAUCU...........................CCCCCCGUGACCUAAAUCACGGAGAAACCCGG
Ga0247554_104968/233-375       UGAAGACG...GUGAGGGAAACAUGUGGAUCAUUGAUCACGAC..........................GUAU...............................................................GUCGGAGUUAAUGC
Ga0265753_1030448/124-358      AGAAGAAGG..AUCGGUGAAACAUGCUUGGUC.....................................AGCCAA........................................................................GGC
Ga0032354_1086862/128-19       GGAAGGGG...CUCAGGGAAACACAGUGGUGCC....................................CGAUUAC......................................................................GGUG
Ga0213850_1119088/23-236       GGAAGACU...GUGAGGGAAACAUGCUACAUAA....................................GUUGAGAA.....................................................................UUAU
Ga0222728_1000079/1837-1731    UGAGGCAU...CUCAGGGAAACAUAGGGA........................................UUUGACAC.........................................................................
Ga0247535_100068/719-828       AGAAGAAU...GGAGGGAAACACAUUGCCGU.....................................AGC...........................................................................GCC
Ga0265744_100861/398-567       AGAAGACU...UCCAGGGAAACACGUGGUG.CU....................................AUU..........................................................................GGUC
Ga0316038_100042/1676-1569     GGAAGAGU...AGGAGGGAAACACCUAUG........................................AUUAGCGAC........................................................................
Ga0184578_101068/480-310       AGAAGAAGC..GUCAGGGAAACACGUAGG........................................CUAUUUGGCA.......................................................................
Ga0265759_100473/818-988       AGAAGAAGC..GUCAGGGAAACACGUAGG........................................CUAUUUGGCA.......................................................................
Ga0265756_100592/1458-1288     AGAAGAAGC..GUCAGGGAAACACGUAGG........................................CUAUUUGGCA.......................................................................
Ga0265746_1003835/1049-1219    AGAAGAAGC..GUCAGGGAAACACGUAGG........................................CUAUUUGGCA.......................................................................
Ga0247536_100773/637-807       AGAAGAAGC..GUCAGGGAAACACGUAGG........................................CUAUUUGGCA.......................................................................
Ga0184573_112336/323-153       AGAAGAAGC..GUCAGGGAAACACGUAGG........................................CUAUUUGGCA.......................................................................
Ga0247530_109035/489-319       AGAAGAAGC..GUCAGGGAAACACGUAGG........................................CUAUUUGGCA.......................................................................
Ga0247513_128543/241-71        AGAAGAAGC..GUCAGGGAAACACGUAGG........................................CUAUUUGGCA.......................................................................
Ga0247541_101130/558-391       AGAAGAAGC..GUCAGGGAAACACGUGGA........................................CUAUUUGGCA.......................................................................
Ga0213850_1278242/600-506      GGAAGACG...GUGGGGGAAACACGCCGCGGCCUA..................................AGGC.......................................................................UAGGCU
Ga0213850_1524759/286-380      GGAAGACG...GUGGGGGAAACACGCCGCGGCCUA..................................AGGC.......................................................................UAGGCU
Ga0180107_1045142/740-541      AGAAGAAA...UCCAGGGAAACAUGGCGA........................................ACUUAUGGCC.......................................................................
Ga0307484_102002/698-552       AGAAGGAAU..UCCGGUGAAACAUGCUCGC.......................................CAUCGCCGAA......................................................................G
Ga0265759_115288/4-102         AGAGGUCA...ACCGGGGAAACACAUGGG........................................AUUAACGAC........................................................................
Ga0316029_136733/88-8          AGAAACAU...GCAAGGGAAACAUGUCCA........................................CAGUGCAA.........................................................................
Ga0242646_1008525/229-119      AGAAGCUG...GUUAGGGAAACAUGACAACCUGCUCUUCCAUAG.AUCUA...................UUUC........................................................UAGACCCUUAGGAAGUUUACA
Ga0115594_1180137/311-161      AGAAGAGC...GCCAGGGAAACAUCGCGGGGG.....................................GGGUUG........................................................................CCC
Ga0179947_1156513/270-495      AGAAGUUG...AUGAGGGAAACAUCUCAG........................................GUAU.............................................................................
Ga0179948_1263234/366-292      AGAAGUUG...AUGAGGGAAACAUCUCAG........................................GUAU.............................................................................
Ga0179947_1148619/378-303      AGAGGUUG...AUGAGGGAAACAUCACAG........................................GUAA.............................................................................
Ga0257141_1138773/402-200      AGAAGCAC...GCAGGUGAAACCACCGCUAUCUGUAAACAUCCAAUCCAACAGUGUGUU..........GAAA...............................................AACACGAUGU.GG.UUCGGUG.CCAAAGAU
Ga0242654_10401151/84-171      GGAAGAUG...GUCAGGGAAACAUUUCAAGGC.....................................AGUUAAUC......................................................................GCU
Ga0184603_114388/264-401       GGAAGUGA...GUGAGGGAAACCAUAUGUUAGG.GCGC...............................UAUAU...................................................................GCGUACUUA
Ga0316052_118887/313-177       GGAAGUGA...GUGAGGGAAACCAUAUGUUAGGGCGC................................UAUAC....................................................................GCGUACUA
Ga0079039_1671802/197-34       GGAAGACG...ACCAGGGAAACACUCUGUGAG.....................................AGUUAGGG......................................................................CUC
Ga0247542_100007/609-500       GGAAGAUUA..GCCAAAGAAACAAAGAG.........................................AUUUAACGCAC......................................................................
Ga0247552_100007/4311-4420     GGAAGAUCA..GCCAAAGAAACAAGGAGG........................................CUUAACGCA........................................................................
Ga0184586_103737/25-134        GGAAGAUUA..GCCAAAGAAACACAGAGG........................................CUUAACGCA........................................................................
Ga0184594_103311/207-316       GGAAGAUUA..GCCAAAGAAACAAGGAGG........................................CUUAACGCA........................................................................
Ga0265756_101275/650-541       GGAAGAUUA..GCCAAAGAAACAAGGAGG........................................CUUGACGCA........................................................................
Ga0247541_100010/3268-3377     GGAAGAUUA..GCCAAAGAAACAAGGAGG........................................CUUAACGCA........................................................................
Ga0184599_154003/184-293       GGAAGAUUA..GCCAAAGAAACAAAGAGG........................................CUUAACGCA........................................................................
Ga0184580_121439/573-464       GGAAGAUUA..GCCAAAGAAACAAAGAGG........................................CUUAACGCA........................................................................
Ga0184582_108821/1050-1159     GGAAGAUUA..GCCAAAGAAACAAAGAGG........................................CUUAACGCA........................................................................
Ga0184575_101094/586-477       GGAAGAUUA..GCCAAAGAAACAAAGAGG........................................CUUGACGCA........................................................................
Ga0184598_129111/512-403       GGAAGAUUA..GCCAAAGAAACAAAGAGG........................................CUUAACGCA........................................................................
Ga0184596_107732/313-205       GGAAGAUUA..GCCAAAGAAACAAAGAGG........................................CUGACGCA.........................................................................
Ga0247554_100006/1592-1485     GGAAGAUUA..GCCAAAGAAACAAAGGGG........................................CAACGCA..........................................................................
Ga0247534_100047/1899-2008     GGAAGAUUA..GCCAAAGAAACAAAGAGG........................................CUUAACGCA........................................................................
Ga0184597_128933/573-464       GGAAGAUUA..GCCAAAGAAACAAAGGGG........................................CUUAACGCA........................................................................
Ga0213850_1302171/343-113      GGAAAACG...GUGAGGGAAACACAAGG.........................................AAACC............................................................................
Ga0213850_1513954/279-196      CGAAGUCC...GUCGGUGAAACACGCAUGGGC.....................................GAAA..........................................................................GCC
Ga0153880_1231581/116-241      GGAAGCUG...GUGAGGGAAACACAGUGAGU.CGAAGUGGACGGGGCC.....................AAA...........................................................GGUCCUGAUUAC.UCGUAU
Ga0247541_104364/333-188       UGAAGAUC...GUAAGGGAAACAUACUACGUCACU..................................AAUU.......................................................................AGUU.C
Ga0138277_1028338/433-516      GGAAGAAG...GUGAGGGAAACAUACUA.........................................GAAU.............................................................................
Ga0265752_101001/521-687       GGAAGAAU...GUAGGGGAAACAUGCUAGCU......................................AAGCCGA........................................................................AG
Ga0307482_1449537/408-223      AGAAGAAUU..GUCAGUGAAACAUGCGGGC.......................................CUUAUGGCA.......................................................................G
Ga0257136_1044348/475-684      UGAAGAUG...ACCAGGGAAACAUGACGUACA.....................................GUAA..........................................................................UGU
Ga0257138_1055324/266-476      UGAAGAUG...ACCAGGGAAACAUGACGUACA.....................................GUAA..........................................................................UGU
Ga0257140_1130477/77-287       UGAAGAUG...ACCAGGGAAACAUGACGUACA.....................................GUAA..........................................................................UGU
Ga0127502_10711632/191-294     GGAAGAUA...GAAGGGGAAACAUCUGUAAUU.....................................AAC...........................................................................GAU
Ga0184568_105547/129-239       AGAAGGUA...CCGAGGGAAACAUGCG..........................................UUUAAC...........................................................................
Ga0206353_11976089/292-380     GGAAGAAC...GUCAGGGAAACACG...UGU.UGUC.................................CUAG......................................................................GACAUGU
Ga0184577_108791/182-295       CGAAGACA...ACCAGGGAAACACCCAGCA..UCGCUUG..............................GCUUG..................................................................CAAGCGCGCU
Ga0242649_1050441/340-170      GGAAGCCUAGAGGGAGGAAAACACGUCAGC.C.....................................UAGC..........................................................................GAG
Ga0179934_1197833/207-115      GGAAGACU...GGAAGGGAAACACUCU..........................................ACCA.............................................................................
Ga0180107_1174136/218-102      AGAAGCUA...GCCAGGGAAACCUUC...........................................UUCGU............................................................................
Ga0224712_10002011/1106-1211   UGAGGCUU...GCCAGGGAAACACUCUAGAGU.....................................AGCG..........................................................................AC.
Ga0224712_10010317/2429-2325   UGAGGCAU...GCCAGGGAAACACUGUAGAGU.....................................AGCG..........................................................................AC.
Ga0206355_1311803/144-334      AGAAGACA...GCCAGGGAAACACGUAUUGUGUGCUGGGUGAUAAGAUGUCU.UUUCGAA.........CAUUUUCUCUCUCCCCCCACAU.............................G.UGUGAGGGUCA..AUAUCACCC......
Ga0307482_1417159/374-296      GGAAGUCA...GGUGGGGAAACACAUCCUC.......................................UCAC............................................................................G
Ga0213850_1396191/936-678      GGAAAACG...GUGAGGGAAACACUCGG.........................................AAGCC............................................................................
Ga0222728_1287830/12-211       UGAAGAUG...GCCAGAGAAACACGCCACCACU...AGU..............................GAAA...................................................................GCUGAAAGUG
Ga0242652_1169169/12-211       UGAAGAUG...GCCAGAGAAACACGCCACCACU...AGU..............................GAAG...................................................................ACUGAAAGUG
Ga0242654_11312699/30-229      UGAAGAUG...GCCAGAGAAACACGCCACCACU...CGU..............................GAAG...................................................................GCGGAAAGUG
Ga0265759_104327/339-243       GGAAGAGU...GUGAGGGAAACACAGGUGUCGUGAAAUGAGC...........................UACG................................................................GCUC.UUGAACGA
Ga0265756_100717/910-1050      UGAAGAAU...CCAGGGGAAACAUACUGUCC......................................GCCCGA.........................................................................GG
Ga0265757_113513/277-362       GGAAGAAC...GUGAGGGAAACACACCA.CCAG....................................GACA.........................................................................CUGG
Ga0242654_10024002/836-915     GGAAGAGU...GUGAGGGAAACACACGU.........................................AAAUCACA.........................................................................
Ga0184573_101763/767-477       AGAAGAAGG..AUCGGUGAAACAUGCUUGCCU.....................................GCCG..........................................................................AGG
Ga0242654_10701525/53-296      AGAAGAUUC..UCCGGGGAAACACUAUGGC.......................................UAUAUGGC........................................................................G
Ga0247529_117158/253-331       GGAAGACU...GAUGGCAAAACACGGGAAAA......................................UAAA...........................................................................UU
Ga0326631_117973/181-293       GGAAGAGG...GCUCGGGAAACACGCACGAGCGGCU.................................CAGAUUA...................................................................AG.CGC.
Ga0316038_107116/33-174        GGAAGAUA...GCUAGGGAAACACCCGUCGCGCAAGUAACAUCGAAGGGAUCUU...............AAUAAA..................................................AAGAUU..UUCGGUUCUGCGA.CGU
Ga0316037_108094/19-160        GGAAGAUA...GCUAGGGAAACACCCGUCGCGCAAGUAACAUCGAAGG.....................GAGCGUAAUAAAAAGA..............................................UUUUCGGUUCUGCGA.CGU
Ga0247527_116942/161-88        CGAAGCUU...GCCAGGGAAACAUCGUUUU.......................................UCUU............................................................................A
Ga0138278_1022898/219-127      GGAAGACA...GUGGAGGAAACACGCGUAAGGUU...................................GCU.........................................................................AACCU
Ga0127503_11028958/51-130      AGAAGACU...GUGAGGGAAACACGUAC.........................................UAA..............................................................................
Ga0307482_1623775/241-347      GGAAGACU...AGGAGGGAAACACGUGCG........................................CUUAGCGAA........................................................................

                               ....>>>>...<<<<<.......<<<<<<<<<<<<<<<<<<<<<<<<<<<<<<<<<<<<<<<<<<<<<<.........................................>>>>>>>>>>>>>>>>>>>>>>>>>>>>>>>>>>>>>>>>
                               ....1222...22222.......2222222..............................1..1100...........................................0011..1..............................2
                               RGAAG●●●---R●●RGRGAAACAY●●●●●○○--------------------------------------●●●●○--------------------------------------------------------------------------○○
```

```
Ga0307484_111921/444-550    GGAAGACU...AGGAGGGAAACACGUGCG........................................CUUAGCGAA........................................................................
Ga0307484_101582/1018-1124    GGAAGACU...AGGAGGGAAACACGUGCG........................................CUUAGCGAA........................................................................
Ga0265742_1021317/385-459    GGAGGACG...GUGGGGGAAACACGUG..........................................GAAA.............................................................................
Ga0157569_1089063/252-138    AGAAGACU...GUAGGUGAAACACCGCGUGCUGAAAUGAUAAG..........................CAUAAUUCUUAAUUUAAU.................................................UUUA..AUUUGA..
Ga0153880_1457361/236-121    GGAAGACU...GUAGGUGAAACACCGCGUGCUGAAAUGAUAGGCAUAAUU...................CUUA..........................................................AAUUA.GUA.AUGAUUUGA
Ga0256352_1087084/23-118    AGAAGACC...GAAGGUGAAACACCG..CCA.......GCAAU..........................UUA................................................................AUUGCUUUAAUAUG
Ga0307482_1000325/24-131    GGAAGAUC...GCUGGUGAAACAUUAUCG........................................AUUAACGAC........................................................................
Ga0157560_1147189/148-60    GGAAGUAC...GUCAGGGAAACAACAUCGGUC......................................AAG...........................................................................GAC
Ga0247519_105128/410-263    GGAAGACUUG.AAGAGAGAAACACCUGUG........................................AUUAACGAC........................................................................
Ga0184573_113193/903-756    GGAAGACUUG.AAGAGAGAAACACCUGUG........................................AUUAACGAC........................................................................
Ga0157593_1013623/299-215    GGAAGACG...GUGAGGGAAACAUACUCU........................................UACA.............................................................................
Ga0184593_126886/349-213    GGAAGAGG...GCUGUGGAAACAUGCACGAGCGGCU.................................AGUAUAA...................................................................AG.CGCU
Ga0184568_117306/561-697    GGAAGAGG...GCUGUGGAAACAUGCACGAGCGGCU.................................AGUAUAA...................................................................AG.CGCU
Ga0184597_120210/233-147    GGAAGACU...GUGAGGGAAACACGCACGCAG.....................................UUG...........................................................................CUG
Ga0184570_100371/69-155    GGAAGACU...GUGAGGGAAACACGCACGCAG.....................................UUG...........................................................................CUG
Ga0157562_1160399/232-315    GGAAGACC...CCAAGGGAAACACAGUAGUU......................................AUC............................................................................AA
Ga0265744_114151/132-24    GGAAGCGAUG.AUCAGGGAAACCACACUUAUGCCGU.................................AUUGC.....................................................................AC.GCGU
Ga0265743_119695/104-184    GGAAGAGC...GGUCGGGAAACAUGCUGAA.......................................UAAGU.............................................................................U
Ga0265743_101198/448-619    AGAAGACU...UCCAGGGAAACACAUGAUG.CU....................................AUU..........................................................................GGUC
Ga0316046_121611/330-214    UGAAGACU...GCAAGGGAAACACCGC.........UACCUC...........................AAAUAACUAC..........................................................GAGGUGUAAUUUA
Ga0265757_120140/89-204    UGAAGACU...GCCAGGGAAACACCGC.........UGCCU.C..........................UUCCUUU............................................................GAAGGCGUAAUUUU
Ga0138279_1083071/419-326    GGAAGCAG...GUCGGUGAAACACCC...........................................AUCAA............................................................................
Ga0310106_100998/1608-1690    GGAAGCAU...AGCAGGGAAACACUGUUAUUC.....................................GAAA..........................................................................GAA
Ga0265756_102054/286-395    GGAAGAAC...AGCAGGGAAACAUGCAUCU.C.....................................UAAC..........................................................................GAA
Ga0180108_1209503/2438-2343    GGAGGUUA...UCCAGGGAAACACAUAGUCGU.....................................AACG..........................................................................AC.
Ga0180107_1057591/210-107    GGAAGCUA...GGCAGGGAAACAUCAC..........................................UAUA.............................................................................
Ga0307482_1074296/478-354    GGAAGUGUA..GUCAGCGAAACACCG.UUAAGGCGC..................................AUCA......................................................................GUGCCUU
Ga0180108_1022680/4248-4153    GGAAGUUC...ACCAGGGAAACAUAUGGUCGU.....................................AGCG..........................................................................AC.
Ga0247519_103291/1381-1460    GGAAGCAG...UGUGGGGAAACACCAUC.........................................GAUG.............................................................................
Ga0184590_126350/300-380    GGAAGCAG...UGUGGGGAAACACCAUC.........................................AAUA.............................................................................
Ga0247551_100996/477-556    GGAAGCAG...UGUGGGGAAACACCAUC.........................................AAUG.............................................................................
Ga0316035_144545/195-301    GGAAGAGG...GUGAGCGAAACACCACGCAGAUU........UGGC.......................CUUUAA..........................................................GCCUUUCCUGACAAUCU
Ga0316049_154968/227-319    GGAAGUAA...GCCAGGGAAACAUACC..........................................AUGUAA...........................................................................
Ga0184593_115334/333-240    GGAAGUAA...GCCAGGGAAACAUACCUU........................................UGU..............................................................................
Ga0307921_1005950/551-460    GGAAGACU...GCGAGGGAAACACGACAA........................................UGAUA............................................................................
Ga0242647_1014694/423-336    GGAAAAUC...GCAAGGGAAACACAUCU.........................................AUUACGUC.........................................................................
Ga0184580_102899/442-582    GGAAGAGG...GCCAAGGAAACACGUUAGUGCCA....................................GGGA........................................................................UGGCC
Ga0265743_108998/150-253    AGAAGAAAU..GAGAGGGAAACAUGUCAC........................................CCUAGCGAA........................................................................
Ga0316050_103193/566-460    AGAAAAUA...GAGAGGGAAACAUAUCAC........................................CGUAACGAA........................................................................
Ga0316030_100208/805-700    AGAAGAUAU..GAGAGGGAAACAUGAUGA........................................CUUAGCGAC........................................................................
Ga0307482_1000262/4800-4699    GGAAGAGA...GCCGGGGAAACACGUCAGCGU.....................................AGC...........................................................................GCA
Ga0307482_1018929/1182-1081    GGAAGAGG...GCCGGGGAAACAUGUCAGCGU.....................................AGC...........................................................................GCA
Ga0153880_1260556/441-589    GGAAGAGC...GCAGAGGAAACAUGCAUACAGUAGUAUAACCUACAAAUCAAUAU..............CAACUAAA...............................................AUAUUGUAA.GUAGGAC..ACU.CAG
Ga0180044_1087599/197-316    GGAAGAAA...GUGGAGGAAACACGCGCGGUUGUA..GAGAC............................AAAGC...............................................................GUCUCAAUACGGC
Ga0127502_11371108/362-252    AGAAGAGAC..GAGAGGGAAACAUGCCAG........................................AUUCACGAC........................................................................
Ga0184588_106948/865-640    CGAAGGAA...GUAGGGGAAACAUGCGUACGACACCUGCUGGUUGACCACACGGUU.............AACAACGCAGAGCUCGCCCACAGAAGGGAAUC......................ACCCCGAUGUGUGG.....ACUCGGUG
Ga0247533_100438/1040-888    AGAAGAAU...CUCAGGGAAACAUGCACUGG......................................UUUGCUGU.......................................................................CC
Ga0127502_10189886/163-55    GGAAGACA...GCUAGGGAAACACCACG...CGA.CAACCAAGCACU......................UAUUC..........................................................AGUGCGA.GUUGAUCGGC
Ga0079074_1318576/412-571    AGAAGACA...GCCAGGGAAACACGUAAGGGG.....................................GGGUGAG.......................................................................CCC
Ga0115594_1212468/8-118    AGAAGACC...GUGAGGGAAACAUACAUAGAG.AUAC................................ACUUGGG...................................................................GUAUGCUC
Ga0115594_1134035/652-542    AGAAGACC...GUGAGGGAAACAUGCAUAGAG.AUAC................................ACUAGGG...................................................................GUAAGCUC
Ga0316030_105840/623-504    AGAAGCUU...GCAAGGGAAACACACACA........................................AAACAAAAA........................................................................
Ga0316033_110175/528-409    AGAAGCUU...GCAAGGGAAACACACACA........................................AAACAAAAA........................................................................
Ga0228708_1128185/228-344    AGAAGUCA...GCAAGGGAAACACUUAUGCACACCUA.................................GAUA.....................................................................UAGGU.UG
Ga0126318_10199518/109-204    GGAAGCUC...GUGAGGGAAACACCCCGCUC......................................AGAU...........................................................................GG
Ga0265745_1000697/596-785    AGAAGAUUC..ACCAGGGAAACACUCGUGU.......................................UUAAGGC.........................................................................A
Ga0265747_105373/294-105    AGAAGAUUC..ACCAGGGAAACACUCGUAC.......................................UUGAGGC.........................................................................G
Ga0265745_1001084/890-701    AGAAGAUUC..ACCAGGGAAACACUCGUAC.......................................UUAAGGC.........................................................................G
Ga0265745_1000512/1043-854    AGAAGAUUC..ACCAGGGAAACACUCGUAC.......................................UUGAGGC.........................................................................G
Ga0310117_104105/1058-869    AGAAGAUUC..ACCAGGGAAACACUUGCAC.......................................UUAAGGC.........................................................................G
Ga0184587_113157/56-168    AGAAGCAUA..ACCGGUGAAACACGCGGCAU.CC...................................CAGG........................................................................GGCAU
Ga0206356_10600373/215-21    AGAAGACA...GGCAGGGAAACACUCACGUUUUAUAAGGUAGACGCGAUAACUUUUCGAUU........AUCAACUCUCCCCCAUGGUCAAGAUA.......................AAUUGACA..UGAUCUUG..GAGCACGUCUAA
Ga0307920_126542/349-261    GGAAGAAA...UCUAGGGAAACACGCCA.........................................CACU.............................................................................
Ga0265742_1001154/131-376    GGAAGACG...GUGAGGGAAACACGGA..GUUGGCCUACGCCC............................UUCG...............................................................GGGCGGA.GCGAGC
Ga0307482_1127544/505-398    GGAAGACC...GCCAGGGAAACACCGUCAGCUGUGU.................................AUUU......................................................................ACCCAGC
Ga0127503_11058765/362-444    GGAAGCUA...GGCAGGGAAACACCGU..........................................AAUGC............................................................................
Ga0210323_1050507/418-532    GGAAGAAC...GCCAGGGAAACACGCGAUGUAAC..AGCCUUUCAAGAACA....................AAG........................................................UGUUCUAAU.AGGUCAAGCUGC
Ga0242647_1002063/315-62    AGAAGACC...GGUGGUGAAACAUGCUUGC.......................................CUGCCGAA........................................................................G
Ga0307484_100992/499-246    AGAAGACC...GGUGGUGAAACAUGCUUGC.......................................CUGCCGAA........................................................................G
Ga0247534_117055/74-212    GGAAGAGG...UCCAGGGAAACAUAGUUAGUACCAGUGACCGGGCC.......................AGCG.............................................................AGCCC...GCGAGGGC
Ga0129331_1494381/292-368    GGAAGACG...GCAAGGGAAACAUCGGUC........................................GGA...............................................................................
Ga0242646_1000880/695-856    AGAAGAAU...UCCAGGGAAACACUCUAUG.C....GUGGC............................CAUUACA..............................................................GCCACUUGGGUC
Ga0180107_1174136/376-571    GGAAGACG...CUAAGUGAAACACACGCG...CAGCU.....AACGCUGUU.CUGGACCUGGAAU....CUAUCCAGGUCUGUCUGCAAUUU......................AUGCUGG...CAGCAACCUCUUUGAGGGAGCCGGCA
Ga0307921_1050271/251-412    GGAAGAUG...GAGAGGGAAACACGCCUUA.......................................CCUAGU............................................................................U
Ga0247524_126530/125-198    GGAAGUAA...CUCAGGGAAACACACAUU........................................GGCAA............................................................................
Ga0242646_1001007/790-609    AGAAGUAUA..CUCAGGGAAACACUCUGUCA.....GGGCU............................AUUAC...............................................................GGCCCUUUGGUG
Ga0213850_1280121/544-351    GGAAGACA...GCUGGGGAAACACGUGUGUGUGA...................................GCGA........................................................................UCGCA
Ga0307919_1109364/247-158    GGAAGACA...GUUAGGGAAACACAGAAGAACGG...................................GCU.........................................................................CCGUU
Ga0184600_147964/253-339    GGAAGACG...GUGAGGGAAACAUAAAUGUACA....................................GAAA.........................................................................UGUA
Ga0316036_107710/408-322    GGAAGACG...GUGAGGGAAACAUAAAUGUGCA....................................GAAA.........................................................................UGUA
Ga0184598_138497/440-355    GGAAGACA...GUGAGGGAAACACAAAUGUGCA....................................GAAA.........................................................................UGUA
Ga0184589_121067/502-587    GGAAGACA...GUGAGGGAAACACAAAUGUGCA....................................GAAA.........................................................................UGUA
Ga0184601_135946/406-321    GGAAGACA...GUGAGGGAAACACAAAUGUGCA....................................GAAA.........................................................................UGUA
Ga0115595_1093158/7-137    GGAAGCCG...GCCAGGGAAACAACAAAAGAAG.....GAGCUAUACCUUCC...............UCACC.......................................................GGAAGGC...GCUCUUAAACUUC
Ga0005851_1006483/274-172    GGAAGACC...GUGAGGGAAACAUGCUUU........................................UGC...............................................................................
Ga0247522_108409/684-554    AGAAGGAUU..GCCGGUGAAACAUGCUCGC.......................................CCGCCGAA........................................................................G
Ga0310119_105137/743-673    GGAAGCAG...GCUGGGGAAACAUACUC.........................................GAAA.............................................................................
Ga0247529_116306/255-362    GGAAGCAC...GGUAGGGAAACAUGCGUCGCAG....................................CCGU.........................................................................CUGC
Ga0316046_116376/278-384    GGAAGCAC...GGUAGGGAAACAUGCGUCGCAG....................................CCGU.........................................................................CUGC

    ....>>>>...<<<<<.......<<<<<<<<<<<<<<<<<<<<<<<<<<<<<<<<<<<<<<<<<<<<<<.........................................>>>>>>>>>>>>>>>>>>>>>>>>>>>>>>>>>>>>>>>>
    ....1222...22222.......2222222..............................1..1100............................................0011..1..............................2
    RGAAG●●●---R●●RGRGAAACAY●●●●○○---------------------------------------●●●○○--------------------------------------------------------------------------○○
```

```
Ga0247534_102735/124-18          GGAAGCAC...GGUAGGGAAACAUGCGUCGCAG....................................CCGU.........................................................................CUGC
Ga0247540_102569/210-104         GGAAGCAC...GGUAGGGAAACAUGCGUCGCAG....................................CCGU.........................................................................CUGC
Ga0316037_112281/325-219         GGAAGCAC...GGUAGGGAAACAUGCGUCGCAG....................................CCGU.........................................................................CUGC
Ga0184576_110594/6-113           GGAAGCAC...GGUAGGGAAACAUGCGUCGCAG....................................CCGU.........................................................................CUGC
Ga0316049_114344/181-288         GGAAGCAC...GGUAGGGAAACAUGCGUCGCAG....................................CCGU.........................................................................CUGC
Ga0115594_1200134/154-31         GGAAGACCA..GUCGGUGAAACAUCUGUAGACGGGAAAAGGCCUGUGC.....................ACA............................................................GCACAUGUC...CCC..C
Ga0307480_1000549/913-1064       AGAAGUAAC..GACAGGGAAACAUACCGGUG..AGAGG.C.CGAAUUGAC.CAAACGC...........AAC.................................................GCGUUAGCGUCGGUUGGAGUCCUAAGGCA
Ga0242647_1000589/378-557        GGAAGUCC...ACCAGGGAAACACUGGCAUCUAACUUGGAACUUAGGUCGAAGGAUC............UUCG.................................................GGUCUGUC..CCUUUGUUCGUGUCGAGA
Ga0222729_1000922/1729-1550      GGAAGUCC...ACCAGGGAAACACUGGCAUCUAACUUGGAACUUAGGUCGAAGGAUC............UUCG.................................................GGUCUGUC..CCUUCGUUCGUGUCGAGA
Ga0182091_1104347/98-194         AGAAGACA...GCAAGGGAAACAUGCAGCGCGUUU..................................GCU........................................................................AAACGC
Ga0316037_128286/253-347         GGAAGACC...GUCAGGGAAACAUGCGCGAAGUCU..................................GCU........................................................................AGGC.U
Ga0180107_1040049/1100-1010      GGAAGAAA...GCAAGGGAAACACGUCGGCGG.....................................UCGUC.........................................................................CUG
Ga0242641_1014724/551-384        AGAAGAACA..UCCAGGGAAACAUCGGC...CCGGAC................................CAUUACAGUCC..............................................................GUUUGGGU
Ga0242641_1002366/499-331        AGAAGAACA..UCCAGGGAAACAUCGGC...CCGGAC................................CAUCACAGUCC..............................................................GUUUGGGU
Ga0213850_1508869/496-402        GGAAGACC...GCAAGGGAAACAUGCACCAGGUU...................................GCU.........................................................................AAUCU
JGI12272J11983_1072695/40-150    AGAAGAGAU..GGGAGGGAAACAUGCUGGAGU.....................................AGCG..........................................................................AC.
Ga0307482_1020622/600-404        AGAAGAUC...UCCAGGGAAACACACUGUGGGCU...................................AAGU........................................................................GGUCC
Ga0242657_1009512/1576-1466      AGAAGAGUU..GGAAGGGAAACACACCAG........................................CCUAACGAU........................................................................
Ga0184577_116723/415-311         AGAAGAUCC..CGCAGUGAAACACGUGU...CCUAU.................................CUA.......................................................................AUAGGUA
Ga0184576_109717/432-536         AGAAGAUCC..CGCAGUGAAACACGUGU...CCUAU.................................CUA.......................................................................AUAGGUA
Ga0184572_113323/230-300         AGAGGACC...UAGGGUGAAACACGUGU.........................................AUA..............................................................................
Ga0307354_155926/276-116         AGAAGACA...GCCAGGGAAACACACAAGAGA.....................................AGUAAGG.......................................................................UCU
Ga0265756_100433/485-286         AGAAGCAAA..UCCAGGGAAACAUACUGUC.UAUCACCUGCCUUGUACGUGUUCGGGUGGUUUC.....CCACCGUACUAGGCACAUGGAGCUCGCUAUCAUGAAGGGGU.....GAAACCCUUC.....UGUACGAGAUA.ACUAGGCA
Ga0265751_100477/874-675         AGAAGCAAA..UCCAGGGAAACAUACUGUC.UAUCACCUGCCUUGUACGUGUUCGGGUGGUUUC.....CCACCGUACUAGGCACAUGGAGCUCGCUAUCGUGAAGGGGU.....GAAACCCUUC.....CGUACGAGAUA.ACUAGGCA
Ga0180045_119797/166-305         GGAAGAAC...GCCAAGGAAACACUCUAUGGA.....................................AGCGG.........................................................................UCC
Ga0222728_1380294/134-238        GGAAGCAC...GACGGUGAAACAUCUAUC........................................UGUUGGAU.........................................................................
Ga0265758_104499/242-367         GGAAGAGG...GCCAGGGAAACAUAGCGUGUGC....................................CAGAA.......................................................................GUGC
Ga0079039_1551950/237-315        GGAAGACU...GCAAGGGAAACAUGCU..........................................AUGAGC...........................................................................
Ga0157570_1034241/151-229        GGAAGACA...GACGGUGAAACACGUGCAGC......................................UAG............................................................................GC
Ga0157571_1064089/212-134        GGAAGACA...GACGGUGAAACACGUGCAGC......................................AAG............................................................................GC
Ga0179951_1211246/478-633        GGAAGACG...GGCAGGGAAACAUAAUUGUGACAUUACAUUUCCUACAGCA.CUGUAAAACAUUGGGGCACGU.....................................GCCCCGCCGUC.UACAGAUGC.............GUGUAA
Ga0179950_1197742/286-441        GGAAGACG...GGCAGGGAAACAUAAUUGUGACAUUACAUUUCCUACAGCA.CUGUAAGACAUUGGGGCACGU.....................................GCCCCGCCGUC.UACAGAUGC.............GUGUAA
Ga0184578_120646/150-246         GGAAGCUG...GUGAGGGAAACCACAGAGUUCUU...................................UUA.........................................................................AAGAU
Ga0247526_105246/689-508         AGAAGAUCU..UCCAGGGAAACACGCUUAGG......................................AAUUGG.........................................................................UC
Ga0247538_100442/1363-1181       AGAAGAUCU..UCCAGGGAAACACGCUUAGG......................................AAUUGG.........................................................................UC
Ga0247515_107373/548-731         AGAAGAUCU..UCCAGGGAAACACACUUAGG......................................AAUUGG.........................................................................CU
Ga0247538_100551/721-542         AGAAGACU...UCCAGGGAAACACACUUAGG......................................AAUUGG.........................................................................UC
Ga0184588_131258/156-253         CGAAGCUC...CAUAAGGAAACACACUAU........................................UAA..............................................................................
Ga0316047_100860/1040-888        AGAAGAAU...CUCAGGGAAACAUGCACUGG......................................UUUGCUGU.......................................................................CC
Ga0242657_1476020/160-244        GGAAGAUA...GCCAGGGAAACAUACUAAAUGUU...................................AGUU........................................................................AACAA
Ga0184602_130623/1321-1100       AGAAGAAC...UCCAGGGAAACACUCUUAUGAC....................................UCG..........................................................................GUGA
Ga0153880_1410217/288-397        GGAAGAUA...GGAAGGGAAACAUGCUCAC.......................................AAAU............................................................................G
Ga0316049_129960/371-262         GGAAGACA...GUGAGGGAAACAUGCAGA..CG.AGUUG.GUGACU.......................GUGG............................................................AGUCACUCAACAAUGAC
Ga0265747_125875/320-395         GGAAGAAC...GUGAGGGAAACAUGCGC.........................................GGG..............................................................................
Ga0242647_1002473/759-521        AGAAGAUUC..ACCAGGGAAACACGUUGGG.......................................UAUCUGGC........................................................................C
Ga0242641_1004288/32-112         GGAAGUAC...GCUGGGGAAACACUCCA.........................................UACC.............................................................................
Ga0247553_101094/709-629         GGAAGUAU...GCUGGGGAAACACCAC..........................................UCCGUC...........................................................................
Ga0127503_10814644/496-352       GGAAGAGA...GUCAGGGAAACAUAAAUUC.AGAGAGU...............................GAGCCGA.................................................................ACUCUUUCG
Ga0179939_1199779/67-205         GGAAGACC...ACCAGGGAAACAUUCU.CAUGUCUCGACCUGCCCGCC.........UC..........UCAAAUCCAAGUCGCUUGUCAC.............................GACCCAGUGAGGGCAG..AAGUCAA..CAU
Ga0180107_1021361/468-373        GGAAGAAC...ACCAGGGAAACACUCAAG........................................CGUAGCGAA........................................................................
Ga0180107_1218358/314-219        GGAAGUAA...UCCAGGGAAACACUCAAC........................................CAUAGCGCA........................................................................
Ga0265758_100138/1527-1633       UGAGGACA...CCCAGGGAAACAUAUGCAG.......................................GUGGCA..........................................................................C
Ga0265752_100416/442-337         UGAAGACA...CCCAGGGAAACAUGUAUAU.......................................GUGGCG..........................................................................A
Ga0247534_117029/65-195          GGAAGCGU...ACCGGUGAAACACACCAGCC...CCUCG..............................UGAAAGAU...............................................................CGAGGAGAGG
Ga0187793_1322799/72-151         GGAAGACU...GCAGGGGAAACACAGGG.........................................CAAGGAGAAU.......................................................................
Ga0307921_1005950/791-877        GGAAGACC...GCAAGGGAAACACAGGUAUCAU....................................AAA..........................................................................AUGA
Ga0180047_1128081/151-64         GGAAGACU...GCCGGUGAAACACAGCACA.......................................CAAG............................................................................U
Ga0157593_1125711/213-128        GGAAGACU...GCCGGUGAAACACAGCACA.......................................CAAG............................................................................U
Ga0157569_1032488/120-205        GGAAGACU...GCCGGUGAAACACAGCC.........................................AAAACAAA.........................................................................
Ga0157565_1077069/156-67         GGAAGACU...GCCGGUGAAACACGUUGUGC......................................GCAA...........................................................................GC
Ga0157593_1023702/238-314        GGAAGACA...GUUGGUGAAACACGCU..........................................ACAACAAUAC.......................................................................
Ga0255267_1268184/91-184         GGAAGACA...GUCGGUGAAACACGC...........................................CUACGA...........................................................................
Ga0307919_1081825/191-74         AGAAGAAA...GUGGAGGAAACACACCAAA.CGUCCUGA..............................CUAUACGC...............................................................UCAGUUCGAU
Ga0307921_1095896/178-61         AGAAGAAA...GUGGAGGAAACACACCAAA.CGUCCUG.GC............................UAUAC................................................................GCUCAGUUCGAU
Ga0316040_172648/178-66          AGAAGAUA...GUGGAGGAAACACACCAAA.CGUCCUG.GC............................UAAC.................................................................GCUCAGUUCGAU
Ga0184577_110850/246-358         AGAAGAUA...GUGGAGGAAACACACCAAA.CGUCCUG.GC............................UAAC.................................................................GCUCAGUUCGAU
Ga0307920_113994/130-9           AGAAGAAA...GUGGAGGAAACACACCAAA.CGUCCUAGC.............................UUUUCAAAUUAU..........................................................GCUAGUUCGAU
Ga0307920_113994/654-539         AGAAGAAA...GUGGAGGAAACACACCAAA.CGUCCAGC..............................UUUUCAAAUUAU...........................................................GCUGUUCGAU
Ga0307921_1037919/342-457        AGAAGAAA...GUGGAGGAAACACACCAAA.CGUCCAGC..............................UUUUCAAAUUAU...........................................................GCUGUUCGAU
Ga0242646_1001980/23-280         GGAAGACA...GCCAGGGAAACACUCGGAUGG.....................................AAUAGAAA......................................................................CCA
Ga0179935_1139075/165-78         GGAAGUGG...GCCAGUGAAACAUGCGUAAGUAGU..................................AACU.......................................................................GCUACU
Ga0179944_1246019/249-162        GGAAGUGA...GCAAGUGAAACAUGCAGAAGUAG...................................UGUA........................................................................CUACU
Ga0180044_1069332/269-187        GGAAGUCU...GCUAGUGAAACAUGCAUUG.......................................AAAAAAA.........................................................................C
Ga0157564_1065218/271-353        GGAAGUCU...GCUAGUGAAACAUGCAUUG.......................................AAAAAAA.........................................................................C
Ga0180044_1047678/444-368        AGAAGUCU...GCUGGUGAAACAUGCUUUC.......................................AAA.............................................................................G
Ga0180047_1061805/548-622        AGAAGUCCU..GCCAGUGAAACAUCCUC.........................................AAA..............................................................................
Ga0157569_1073490/184-258        AGAAGUCCU..GCCAGUGAAACAUCCUC.........................................AAC..............................................................................
Ga0213850_1394829/253-105        AGAAGACC...GCCAGGGAAACAUCAUCGUG......................................UAGAAAUUGCGGAU.................................................................CA
Ga0255293_1138691/440-213        GGAAACUC...GCCAGGGAAACACGUC..........................................UAAA.............................................................................
Ga0222728_1000079/1192-1299      AGAAGAAGA..GGGAGGGAAACAUAUCAAAGU.....................................AACG..........................................................................AC.
Ga0081246_1201717/153-259        AGAAGAAAU..GGGAGGGAAACACGAUGUCGU.....................................AGCG..........................................................................AC.
Ga0222728_1216887/51-157         AGAAGAAGC..UGGAGGGAAACACACUGAAGU.....................................AGCG..........................................................................AC.
Ga0265754_1141843/290-185        GGAAGACU...GGGAGGGAAACAUGUCAGCGU.....................................AGC...........................................................................GCA
JGI12272J11983_1345183/364-258   CGAAGAAAU..GGGAGGGAAACACAUCAGCGU.....................................AGCG..........................................................................AC.
Ga0242647_1005934/451-617        AGAAGAUUC..UCCGGGGAAACACAUCGG........................................CUUUACGGCA.......................................................................
Ga0115594_1187657/870-960        GGAAGACU...GGGAGGGAAACACCGUAUAAUUUA..................................UCUC.......................................................................UAAAUU
Ga0115594_1174394/642-729        GGAAGACU...GGGAGGGAAACACCGUACUU......................................AUUCUAAAC......................................................................AA
Ga0184569_112764/943-836         AGAAAACU...GGGAGGGAAACAUGCGUUA.......................................GUAGCGA.........................................................................U
Ga0265750_1013321/389-496        AGAAGAUA...GGGAGGGAAACACAUUGGU.......................................GUAGCGA.........................................................................A

                                 ....>>>>...<<<<<.......<<<<<<<<<<<<<<<<<<<<<<<<<<<<<<<<<<<<<<<<<<<<<<.........................................>>>>>>>>>>>>>>>>>>>>>>>>>>>>>>>>>>>>>>>>
                                 ....1222...22222.......2222222..............................1..1100..........................................0011..1..............................2
                                 RGAAG●●●---R●●RGRGAAACAY●●●●●○○○-------------------------------------●●●●○------------------------------------------------------------------------○○
```

```
Ga0265757_139522/177-70            AGAAGAUA...GGGAGGGAAACACAUUGG.........................................CAUAGCGAA.........................................................................
Ga0316029_116217/368-261           AGAAAACU...GGGAGGGAAACAUGCGUUA........................................GUAACGA..........................................................................U
Ga0316047_100016/2681-2574         AGAAAACU...GGGAGGGAAACAUGCGUUA........................................GUAGCGA..........................................................................U
Ga0256352_1101982/155-277          GGAAGACA...GUCAGGGAAACACGAU...UAGCGG.CAAAUCCC.........................ACCCA.............................................................GGAGUUUGACUGCUGC
Ga0256350_1100736/283-405          GGAAGACA...GUCAGGGAAACACGAUUAUCGGCAAAUC....CC.........................ACCCA.............................................................GGAGUUGAGCUGCCGC
Ga0079039_1443516/199-38           GGAAGACG...ACCAGGGAAACACUCUGUGAA......................................GAUAUGG........................................................................UUC
Ga0115595_1072788/334-441          GGAAGACC...GCCGAGGAAACAUGCUGAGA.GACAAAC...............................GACA....................................................................GGCAGUCGUC
Ga0115594_1022316/346-239          GGAAGACC...GCCGAGGAAACAUGUUGAGA.GACAAAC...............................GACA....................................................................GGCAGUCGUC
Ga0129331_1058147/189-99           GGAAGAAC...GUGAGGGAAACACCGCG..CAU.....................................AAUAUUCGA.....................................................................AUGA
Ga0265751_100127/176-103           GGAAGUAU...GCCAGGGAAACAACCAC..........................................UCA...............................................................................
Ga0307921_1043977/91-164           GGAAGAUA...GAUAGGGAAACACGAG...........................................GCAAA.............................................................................
Ga0307919_1048575/242-90           GGAAGAUG...GAGAGGGAAACACGCCUUA........................................CCUAGU...........................................................................U
Ga0242646_1001086/737-571          AGAAGAUUC..UCCAGGGAAACAUGCAUGU........................................UUAGGU...........................................................................A
Ga0265752_111922/418-343           GGAAGAAA...GGCAGGGAAACAUGCAC..........................................GAAA..............................................................................
Ga0157562_1134247/69-160           GGAAGAAC...GCCAGGGAAACAUGCUGUGC.......................................GCAA............................................................................GC
Ga0157559_1047848/128-41           GGAAGAAC...GCCAGGGAAACACACUGU.........................................AAAC..............................................................................
Ga0179944_1313268/129-232          GGAAGAAC...GCCAGGGAAACAUGCAUCGGCGCUC..................................ACGUAG.....................................................................GAGCGUC
Ga0213850_1259428/445-342          GGAAGAAC...GCCAGGGAAACAUGCAUCAGAGUUC..................................ACGUAG.....................................................................GAACUCU
Ga0153880_1167367/224-121          GGAAGAAC...GCCAGGGAAACAUGCAUCGGCGCUCA.................................AGUA......................................................................UGAGCGUC
Ga0153880_1058095/344-241          GGAAGAAC...GCCAGGGAAACAUGCAUCGGCGCUCA.................................AGUA......................................................................UGAGCGUC
Ga0179943_1178349/195-298          GGAAGAAC...GCCAGGGAAACAUGCAUCGGCUUUC..................................ACGUAG.....................................................................GAGAGUC
Ga0307921_1075885/188-36           GGAAGAUG...GAGAGGGAAACACGCUCUA........................................CCUGGC...........................................................................U
Ga0307921_1062541/180-338          GGAAGAUG...GAGAGGGAAACACGCUCUA........................................CCUGGU...........................................................................U
Ga0242641_1014474/336-257          GGAAGUGG...GUCGGUGAAACAUGCUCUA........................................GUGA.............................................................................U
Ga0307919_1064641/225-73           GGAAGAUG...GAGAGGGAAACACGCUCUA........................................CCUGGC...........................................................................U
Ga0265759_108120/277-351           GGAAGACC...GCCAGGGAAACACCGC...........................................UUAAUUC...........................................................................
Ga0265753_1005277/856-1057         AGAAGAUUC..UCCAGGGAAACAUCUACC.........................................GUUGAAGCA.........................................................................
Ga0247523_120120/325-403           GGAAGCUA...GCCAGGGAAACAUCAACC.........................................UUCG..............................................................................
Ga0228707_1100139/344-194          GGAAGUGU...GGUGGCGAAACACAAUCAAG.C..GUG.GUACCU..........................UUU...............................................................AGGGGCACAUAAGUCU
Ga0316049_154526/155-74            GGAAGAGA...GUGGAGGAAACAUGCA...........................................UAAC..............................................................................
Ga0316035_177575/224-142           GGAAGAGA...GUGGAGGAAACAUGCA...........................................UAAC..............................................................................
Ga0307920_126403/177-486           GGAAGAAG...GCGAGGGAAACACGCGGCGGAGC.....................................GAU..........................................................................GCUCA
Ga0138280_1140856/341-479          GGAAGACC...GCCAGGGAAACACGCCGCUC.......................................AGCG............................................................................GA
Ga0247539_101233/414-526           UGAAGCGA...GUGAGGGAAACAUGUAAUGGUGAGUGGUUG...............................AUUU..................................................................CAACUGCU.ACC
Ga0184569_108885/200-88            UGAAGCGA...GUGAGGGAAACAUGUAAAGGUGAGUGGUUG...............................AUUU..................................................................CAACUGCU.ACC
Ga0247521_112559/317-205           UGAAGCGA...GUGAGGGAAACAUGUAAUUGUGAGUGGUUG...............................AUUU..................................................................CAACUGCU.ACC
Ga0184599_154888/232-155           GGAAGCAG...ACCAGGGAAACAUCCUAU.........................................AAC...............................................................................
Ga0180108_1143860/199-116          AGAAACAG...GGCAGGGAAACACAUCC..........................................CAAG..............................................................................
Ga0247524_121352/374-303           GGAAGCUA...GGUGGGGAAACAUCAUG..........................................AUUAC.............................................................................
Ga0206356_11002907/329-148         AGAAGACA...GCCAGGGAAACACAUGAAAAUUAUGGUGGUGAAGAAAGAAAACUUUAUU..........CUAAACUUCCCCCCGUAUAGACUCUAAUUUUGGA.................AAUUUAGUCU...CUCUACGGAGCUCCACUU
Ga0265743_103007/946-842           UGAAGAAUA..GGAAGGGAAACACAUGAUCGU......................................AGC............................................................................GCC
Ga0316033_101029/151-49            GGAAGCCA...GACAGGGAAACAUCAGCGC........................................GUAGCGA..........................................................................G
Ga0179937_1126086/216-325          GGAAGACC...GGAAGGGAAACAUGCUACAGUUGCACAGAGUCU..........................GCGCUG.............................................................AGAC.CC..GUGACU
Ga0307482_1050048/176-95           GGAAACAU...GCCAGGGAAACAUCAUCU.........................................GAC...............................................................................
JGI12272J11983_1319142/230-159     GGAAGCAC...GGCAGGGAAACAUCAUAC.........................................GUU...............................................................................
Ga0180107_1285918/13-113           GGAAGCUA...GGUAGGGAAACACCGC...........................................UUG...............................................................................
Ga0265756_108694/382-277           GGAAGAAG...GCAAGGGAAACAUGCUGGUCAACACACU...............................AUUUCG..................................................................AGUGUGU...
Ga0265746_1140777/214-319          GGAAGAAG...GCAAGGGAAACAUGCUGGUCAACACACUC..............................UUUC...................................................................GAGUGUGC...
Ga0184574_101359/409-493           GGAAGCCGCC.GGGAGCGAAACAUGCUAAGCCC.....................................AUGCA.........................................................................GGGU
Ga0184568_108556/391-307           GGAAGCCGCC.GGGAGCGAAACAUGCUAGGCCCU....................................UGC..........................................................................GGGGC
Ga0316030_107573/391-475           GGAAGCCGCC.GGGAGCGAAACAUGCUAGGCCC.....................................UAGCC.........................................................................GGGC
Ga0180107_1031754/139-57           GGAAGAUG...GCCAGGGAAACAUGCACUG........................................UUAUUUU..........................................................................C
Ga0307919_1029784/253-144          AGAAAUGG...GUGAGGGAAACACUUUGCGAGGAAACCAACGA...........................AUCAAA..............................................................UCGUUGGGUGCCUU
Ga0307921_1042549/343-452          AGAAAUGG...GUGAGGGAAACACUUUGCGAGGAAACCAACGA...........................AUCAAA..............................................................UCGUUGGAUGCCUU
Ga0307482_1021658/484-563          GGAAGCUU...GCCAGGGAAACAUCUGCU.........................................UUU...............................................................................
Ga0179943_1102267/215-90           GGAAGACC...GCCAGGGAAACACGAGUCUCAG.CAUCCUACUCC..........................UUUC..............................................................GG...GGGAUGAUUGC
Ga0079074_1279626/114-274          AGAAGACA...GCCAGGGAAACACACAAGAGA......................................AGUAUGG........................................................................UCU
Ga0307921_1019702/90-184           GGAAGAUU...GCAAGGGAAACAUACGACGAGAGG...................................GAGA........................................................................UCUCUC
Ga0307920_133961/233-140           GGAAGAUU...GCAAGGGAAACAUACGACGAGAGG...................................GAGA........................................................................UCUC..
Ga0316035_179807/270-184           AGAAGCUG...GCCAGGGAAACAUGCACCUCCU.....................................GUCA..........................................................................AGGA
Ga0256338_1343513/152-228          GGAAGAGG...CCCAGGGAAACACACCAGA........................................UAAA.............................................................................U
Ga0255268_1432700/127-51           GGAAGAGG...CCCAGGGAAACACACCAGA........................................UAAA.............................................................................U
Ga0153880_1067318/492-566          CGAAGAGU...CCGAAGGAAACAUACAUA.........................................CUUAUC............................................................................
Ga0247533_103075/417-305           GGAAGAGG...CCCAGGGAAACACAUAUGCUACGCC..................................AGUGC......................................................................GGUGUAC
Ga0247541_102964/56-168            GGAAGAGG...CCCAGGGAAACACAUAUGCUACGCC..................................AGUGC......................................................................GGUGUAC
Ga0184589_110648/1041-881          AGAAGAAU...CCCAGGGAAACACCGUAGC.....CCGGCU.............................AUCAC.................................................................GGCCGGUGGGUG
Ga0184580_124121/239-79            AGAAGAAU...CCCAGGGAAACACCGUAGC.....CCGGCU.............................AUCAC.................................................................GGCCGGUGGGUG
Ga0184598_101324/530-690           AGAAGAAU...CCCAGGGAAACACCGUAGC.....CCGGCU.............................AUCAC.................................................................GGCCGGUGGGUG
Ga0184588_120624/266-426           AGAAGAAU...CCCAGGGAAACACUGUAGC.....CUGGCU.............................AUUAC.................................................................GGCCAGUGGGUG
Ga0184579_106194/996-1156          AGAAGAAU...CCCAGGGAAACACUGUAGC.....CUGGCU.............................AUUAC.................................................................GGCCAGUGGGUG
Ga0265758_100254/356-516           AGAAGAAU...CCCAGGGAAACACCGUAGC.....CUGGCU.............................AUUAC.................................................................GGCCAGUGGGUG
Ga0184597_106861/196-36            AGAAGAAU...CCCAGGGAAACACCGUAGC.....CUGGCU.............................AUUAC.................................................................GGCCAGUGGGUG
Ga0184600_132066/568-408           AGAAGAAU...CCCAGGGAAACACCGUAGC.....CUGGCU.............................AUUAC.................................................................GGCCAGUGGGUG
Ga0316031_108320/216-376           AGAAUAAU...CCCAGGGAAACACCGUAGC.....CUGGCU.............................AUUAC.................................................................GGCCAGUGGGUG
Ga0184596_109261/979-1139          AGAAGAAU...CCCAGGGAAACACCGUAGC.....CCGGCU.............................AUUAC.................................................................GGCCGGUGGGUG
Ga0316035_107544/354-514           AGAAGAAU...CCCAGGGAAACACCGUAGC.....CUGGCU.............................AUUAC.................................................................GGCCAGUGGGUG
Ga0247527_115808/280-440           AGAAGAAU...CCCAGGGAAACACCGUAGC.....CUGGCU.............................AUUAC.................................................................GGCCAGUGGGUG
Ga0247519_107918/217-58            AGAAGAAU...UCCAGGGAAACACUCCAUC....UGGGUU..............................AUUAU..................................................................AGCCCAGGGUG
Ga0184589_112135/40-200            AGAAGAAU...GCCAGGGAAACACUCUGUC...UUGGGUU..............................AUUAC..................................................................AGCCCAGGGUG
Ga0247551_100195/490-329           AGAAGAAU...UCCAGGGAAACACUCUGUC....UUGGGUU.............................AUUAC.................................................................AGCCCAAGGGUG
Ga0247526_117235/152-313           AGAAGAAU...UCCAGGGAAACACUCUGUC....UUGGGUU.............................AUUAC.................................................................AGCCCAAGGGUG
Ga0326631_101563/544-705           AGAAGAAU...UCCAGGGAAACACUCCAUC.CU...GGGUU.............................AUUAC.................................................................AGCCCAAGGGUG
Ga0184596_111007/525-686           AGAAGAAU...UCCAGGGAAACACUCCAUC.CU...GGGUUA............................UUA..................................................................UAGCCCAAGGGUG
Ga0184573_110190/115-276           AGAAGAAU...UCCAGGGAAACACUCCAUC....UUGGAUU.............................AUUAC.................................................................AGUCCAAGGGUG
Ga0316033_144939/167-328           AGAAGAAU...UCCAGGGAAACACUCCAUC....UUGGGUU.............................AUUAC.................................................................AGCCCAAGGGUG
Ga0247552_101742/612-452           AGAAGAAU...UCCAGGGAAACACUCCAUC....UUGGGUUA............................UUA..................................................................UAGCCCAAGGGUG
Ga0247545_112960/183-343           AGAAGAAU...UCCAGGGAAACACUCUGUC....UUGGGUU.............................AUUAC.................................................................AGCCCAAGGGUG
Ga0247541_103784/123-283           AGAAGAAU...UCCAGGGAAACACUCUGUC....UUGGGUU.............................AUUAC.................................................................AGCCCAAGGGUG
Ga0247524_111427/50-210            AGAAGAAU...UCCAGGGAAACACUCCAU....CUUGGGCU.............................AUUAC.................................................................AGCCCAAGGGUG

                                   ....>>>>...<<<<<.......<<<<<<<<<<<<<<<<<<<<<<<<<<<<<<<<<<<<<<<<<<<<<<<.........................................>>>>>>>>>>>>>>>>>>>>>>>>>>>>>>>>>>>>>>>>>
                                   ....1222...22222.......2222222...............................1..1100............................................0011..1...............................2
                                   RGAAG●●●---R●●RGRGAAACAY●●●●◐○○---------------------------------------●●●◐○---------------------------------------------------------------------------○○
```

```
Ga0247545_104341/406-246         AGAAGAAU...UCCAGGGAAACACUCCAUC....UUGGGUU.............................AUUAC.................................................................AGCCCAAGGGUG
Ga0184602_114416/11-171          AGAAGAAU...UCCAGGGAAACACUCCAUC....UUGGGUU.............................AUUAC.................................................................AGCCCAAGGGUG
Ga0316038_111670/11-171          AGAAGAAU...UCCAGGGAAACACUCCAUC....UUGGGCU.............................AUUAC.................................................................AGCCCAAGGGUG
Ga0184586_133850/368-528         AGAAGAAU...UCCAGGGAAACACUCCAUC....UUGGGCU.............................AUUAC.................................................................AGCCCAUGGGUG
Ga0184600_123882/188-28          AGAAGAAU...UCCAGGGAAACACUCCAUC....UUGGGUU.............................AUUAC.................................................................AGCCCAAGGGUG
Ga0184574_121290/269-109         AGAAGAAU...UCCAGGGAAACACUCCAUC....UUGGGUU.............................AUUAC.................................................................AGCCCAAGGGUG
Ga0316032_107575/215-55          AGAAGAAU...UCCAGGGAAACACUCCAUC....UUGGGUU.............................AUUAC.................................................................AGCCCAAGGGUG
Ga0247519_110701/607-447         AGAAGAAU...UCCAGGGAAACACUCCAUC....UUGGGUU.............................AUUAC.................................................................AGCCCAAGGGUG
Ga0247526_110221/603-443         AGAAGAAU...UCCAGGGAAACACUCCAUCAU....GGGUU.............................AUUAC.................................................................AGCCCAAGGGUG
Ga0247529_134913/224-64          AGAAGAAU...UCCAGGGAAACACUCCAUC....UUGGAUU.............................AUUAC.................................................................AGUCCAAGGGUG
Ga0247529_103231/270-430         AGAAGAAU...UCCAGGGAAACACUCCAUCGU...GGGUU..............................AUUAU..................................................................AGCCCAGGGUG
Ga0184580_114961/306-146         AGAAGAAU...UCCAGGGAAACACUCCAUC...UUGGGUUA.............................UUA...................................................................UAGCCCAGGGUG
Ga0184603_126764/80-240          AGAAGAAU...UCCAGGGAAACACUCCAUC...UUGGGUUA.............................UUA...................................................................UAGCCCAGGGUG
Ga0247550_114736/167-7           AGAAGAAU...UCCAGGGAAACACUCCAUC...UAGGGUUA.............................UUA...................................................................UAGCCCAGGGUG
Ga0184603_111918/241-82          AGAAGAAU...UCCAGGGAAACACUCCAUC....UGGGUUA.............................UUA...................................................................UAGCCCAGGGUG
Ga0247538_100424/120-281         AGAAGAAU...UCCAGGGAAACACUCCAUC...UAGGGUU..............................UUUUAU.................................................................AGCCCAGGGUG
Ga0247547_102464/189-29          AGAAGAAU...UCCAGGGAAACACUCCAUCA....UGGGUU.............................AUUAC.................................................................AGCCCAAGGGUG
Ga0247551_100828/546-387         AGAAGAAU...UCCAGGGAAACACUUCAUC.....UGGGCU.............................AUCAC.................................................................GGCCCAUGGGUG
Ga0247538_100480/788-947         AGAAGAAU...UCCAGGGAAACACUUCAUCA.....GGGCU.............................AUUAC.................................................................GGCCCAUGGGUG
Ga0247550_100850/456-297         AGAAGAAU...UCCAGGGAAACACUUCAUCA.....GGGCU.............................AUCAC.................................................................GGCCCAUGGGUG
Ga0247536_101914/360-201         AGAAGAAU...UCCAGGGAAACACUUCAUCA.....GGGCU.............................AUUAC.................................................................GGCCCAUGGGUG
Ga0247553_110698/526-367         AGAAGAAU...UCCAGGGAAACACUUCAUCA.....GGGCU.............................AUCAC.................................................................GGCCCAUGGGUG
Ga0153880_1114976/150-383        GGAAGAUC...GUCAGGGAAACAUGCAUCAUU......................................GCGA...........................................................................AAU
Ga0180052_189627/240-346         GGAAGACC...GCGAGGGAAACACGAUGGU........................................GGCGA............................................................................A
Ga0184598_136114/144-251         UGAGGCAU...CCUCGGGAAACAUGCAGG.........................................CUUUACUU..........................................................................
Ga0222728_1011988/841-947        UGAGGCAU...AUUGGGGAAACACGGGCA.........................................CAUGACUC..........................................................................
Ga0265751_160438/108-215         GGAAGACA...GCAAGGGAAACACGCGCGCCA......................................GAC............................................................................UGG
Ga0247514_107497/1071-1140       AGAGGCUC...GCCAGGGAAACACGCAUC.........................................UCCA..............................................................................
Ga0180107_1129325/244-60         AGAAGAACA..GUCAGGGAAACACUCAGU.........................................UCGAGC............................................................................
Ga0242646_1002014/11-270         GGAAGACC...GCCAGGGAAACACCACG....GUUC..................................GAGAA......................................................................GAGCACA
Ga0222728_1008526/24-283         GGAAGACC...GCCAGGGAAACACCAGG....GUUC..................................GAGAA......................................................................GAGCACA
Ga0222728_1008391/1281-1023      GGAAGACC...GCCAGGGAAACACCACG....GUUC..................................GAGAA......................................................................GAGCACA
Ga0265759_100017/4211-4288       AGAGGCGU...GCCAGGGAAACACUAUC..........................................GUAU..............................................................................
Ga0247512_104440/911-1082        AGAGGAAGAG.ACCAGGGAAACAUGCUGUAGUCUAGUCUGGGUUUACGGGUAUGUA..............GCCAU..................................................UACAG..CCAUAGACCUA.ACCAGAUU
Ga0265756_131580/159-58          GGAAGAAG...GCGAGGGAAACAUACCACGCAAGCGU.................................CGAA......................................................................ACGCUUGU
Ga0310119_169621/253-177         GGAAGUUC...GCCAGGGAAACACAGAC..........................................AAUU..............................................................................
Ga0247516_124175/202-272         GGAAGCUA...GCGAGGGAAACAUGCAUA.........................................CGU...............................................................................
Ga0247534_100772/309-234         GGAAGUUC...GCCAGGGAAACACAUCU..........................................AUCU..............................................................................
Ga0222728_1054736/119-193        GGAAGCUA...GCCAGGGAAACAUGGA...........................................GAAA..............................................................................
Ga0180106_1297567/444-515        GGAAGCUG...GCCAGGGAAACACC.............................................GUAAA.............................................................................
Ga0242647_1000066/2638-2719      GGAAGUUU...GCCAGGGAAACACCAC...........................................AUAU..............................................................................
Ga0307484_104189/90-171          GGAAGUAA...GGCAGGGAAACACACCU..........................................CACA..............................................................................
Ga0316048_109289/45-149          UGAGGCAU...ACCAGGGAAACACAUCAU.........................................AUUCGGAC..........................................................................
Ga0265750_1038125/531-427        UGAGGCAU...ACCAGGGAAACACAUCAU.........................................AUUCGGAC..........................................................................
Ga0247537_109516/130-26          UGAGGCUU...GCCAGAGAAACACAUCAG.........................................AUUAGGAC..........................................................................
Ga0310115_103434/744-848         UGAGGCUU...GCCAGAGAAACACAUCAG.........................................UUUAGGAC..........................................................................
Ga0307482_1009758/1126-1021      UGAGGCAC...UCCAGGGAAACACGCGGU.........................................CUUGACCA..........................................................................
JGI12272J11983_1356304/1548-1654 UGAGGCAU...ACCAGGGAAACACUUGGG.........................................ACUAGCAA..........................................................................
JGI12272J11983_1072695/645-539   UGAGGUCU...UCCAGGGAAACACAGUGG.........................................CUUAGCAA..........................................................................
Ga0242657_1009512/820-926        UGAGGUCU...UCCAGGGAAACACUCUGUU........................................AUCGCA...........................................................................G
Ga0316037_172473/89-196          UGAGGCAU...ACCAGGGAAACAUGUAGAGG.......................................UCGU............................................................................UC
Ga0184592_121084/378-271         UGAGGCAU...ACCAGGGAAACAUGUGGGGU.......................................UAGC............................................................................AU
Ga0184569_113518/504-609         UGAGGCAU...ACCAGGGAAACAUGUGGGGU.......................................UAGC............................................................................AU
Ga0184602_114837/392-286         UGAGGCAU...ACCAGGGAAACAUGCAGGGG.......................................UCGCU...........................................................................CC
Ga0316030_102131/386-280         UGAGGCAU...ACCAGGGAAACAUGCAGGGG.......................................UCACU...........................................................................CC
Ga0310118_104334/336-443         UGAGGCAU...CCAGGGGAAACAUGCGGUCG.......................................UAGCGU..........................................................................C.
Ga0180108_1146973/462-263        GGAAGCUU...GCUAGUGAAACACGGCAGU........................................GAAGAAAAG........................................................................A
Ga0184580_111164/678-749         GGAAGCUG...GCCAGGGAAACAACAUG..........................................UUCG..............................................................................
Ga0242647_1006852/526-598        GGAAGCUA...GCCAGGGAAACAACCU...........................................AUCU..............................................................................
Ga0179939_1059820/107-216        GGAAGACC...GCCAGGGAAACAUGCUACCAC...GGAUGCAG...........................AGCUUAGA............................................................CCGCACGGGAAGUG
Ga0115594_1156252/268-370        GGAAGACA...GCCAGGGAAACACCGCAGAGG.......................................GGACCAAA.......................................................................CCU
Ga0115594_1006122/120-29         GGAAGACA...GCCAGGGAAACACCGUCAGCU......................................GACCA..........................................................................AGC
Ga0115595_1180708/226-317        GGAAGACA...GCCAGGGAAACACCGUCAGCU......................................GACCA..........................................................................GGC
Ga0115594_1211214/299-389        GGAAGACG...GCCAGGGAAACACCGUUGGCU......................................GACCA..........................................................................AGC
Ga0115594_1007070/120-30         GGAAGACA...GCCAGGGAAACACCGUGAGCU......................................GACCA..........................................................................AGC
Ga0115595_1181384/176-86         GGAAGACA...GCCAGGGAAACACCGUGAGCU......................................GACCA..........................................................................GGC
Ga0115594_1166236/119-19         GGAAGACA...GCCAGGGAAACACCGUCAGCU......................................GACCA..........................................................................AGC
Ga0115594_1157594/155-254        GGAAGACA...GCCAGGGAAACACCGUCAGC.......................................CGACCAA.........................................................................GC
Ga0256352_1182139/180-81         GGAAGACA...GCCAGGGAAACACCGUCAGCU......................................GACCA..........................................................................AGC
Ga0115594_1182529/183-84         GGAAGACA...GCCAGGGAAACACCGUCAGCU......................................GACCA..........................................................................GGC
Ga0265746_1064626/389-458        GGAAGACA...GCCAGGGAAACACGCUC..........................................CAC...............................................................................
Ga0115594_1016490/326-410        GGAAGACA...GCCAGGGAAACACCGCAGCU.......................................GACCA...........................................................................AG
Ga0115595_1007417/328-412        GGAAGACA...GCCAGGGAAACACCGCAGCU.......................................GACCA...........................................................................AG
Ga0228708_1103570/188-285        GGAAGACC...GCCAGGGAAACACGCGGACUAGG.CU.................................GACA......................................................................AGACCUAG
Ga0265751_121301/189-266         AGAGGACG...GCCAGGGAAACACAUGG..........................................AAAUAA............................................................................
Ga0179944_1005311/261-382        GGAAGACC...GCGAGGGAAACACGGC.UAAGCAUCCUACUCC...........................UUUC................................................................GG...GGGAUGACA
Ga0179944_1191552/284-411        GGAAGACA...GCCAGGGAAACACGCAGUC.UCAGCAUCCUACUCC.........................UUUC.............................................................GG...GGGAUGAUCGCG
Ga0179943_1150468/216-89         GGAAGACC...GCCAGGGAAACACGUAGUC.UCAGCAUCCUGCC...........................UUUC...............................................................GG.GGGAUGAUCGCG
Ga0179944_1204933/216-89         GGAAGACC...GCCAGGGAAACACGUAGUC.UCAGCAUCCUGCC...........................UUUC...............................................................GG.GGGAUGAUCGCG
Ga0307482_1020425/440-614        AGAAGAUU...CCCAGGGAAACACGUGAUG.CU......................................AUU...........................................................................GGUC
Ga0242646_1001395/255-509        GGAAGACA...GCCAGGGAAACACUGAGA...UC.....................................UCAAU........................................................................GAUCC
Ga0184578_112031/156-44          UGAAGCUG...GUGAGGGAAACAUAGUGUUGUGGCUAGA...............................UAAUU...................................................................UCUAGUUACC
Ga0184577_120131/220-108         UGAAGCUG...GUGAGGGAAACAUAGUGUUGUGGCUAGAU..............................AGU....................................................................AUCUAGUUACC
Ga0247517_121471/387-499         UGAAGCUG...GUGAGGGAAACAUAGUGUUGUGGCUAGAU..............................AGU....................................................................AUCUAGUUACC
Ga0247539_107237/153-229         GGAAGUAG...ACCAGGGAAACACGAU...........................................UUCG..............................................................................
Ga0265758_109576/83-157          AGAAACCU...CCCAGGGAAACACGC............................................GAAAA.............................................................................
Ga0184577_107529/254-327         GGAAGCAC...GGCAGGGAAACAACACU..........................................ACU...............................................................................
Ga0213850_1246156/171-47         GGAAGAGG...GUCAGGGAAACAUGCGAUGUGCC.....................................AGU..........................................................................GGUGC
Ga0326631_172366/284-207         GGAAGAAG...UCCAGGGAAACAUGCCG..........................................GCACUG............................................................................

                                 ....>>>>...<<<<<.......<<<<<<<<<<<<<<<<<<<<<<<<<<<<<<<<<<<<<<<<<<<<<<<.........................................>>>>>>>>>>>>>>>>>>>>>>>>>>>>>>>>>>>>>>>>>
                                 ....1222...22222.......2222222...............................1..1100.............................................0011..1...............................2
                                 RGAAG●●●---R●●RGRGAAACAY●●●●●○○---------------------------------------●●●●○---------------------------------------------------------------------------○○
```

```
Ga0310114_149639/68-153        GGAAGACA...UCCAGGGAAACACUCGAUCAGC....................................AUC..........................................................................GCUG
Ga0184599_128731/188-273       AGAAGACA...UCCAGGGAAACACCCGAUCAGC....................................AUC..........................................................................GCUG
Ga0310119_131358/533-447       GGAAGACA...UCCAGGGAAACACUCGA.UCAGC...................................AUC.........................................................................GCUGC
Ga0247526_116548/279-354       GGAAGCAU...GACAGGGAAACACAUCU.........................................AUAUC............................................................................
Ga0247519_113845/334-409       GGAAGCAU...GGCAGGGAAACACAUCUG........................................UAU..............................................................................
Ga0247529_111070/303-228       GGAAGCAU...GACAGGGAAACACAUCUG........................................UAU..............................................................................
Ga0184580_116530/249-177       GGAAGCAU...GACAGGGAAACAUCGCU.........................................UAUG.............................................................................
Ga0247524_105844/561-637       GGAAACUA...GGUAGGGAAACAUCAUC.........................................UUUG.............................................................................
Ga0184593_119849/313-238       GGAAGCUA...GAUGGGGAAACAUCAC..........................................UCA..............................................................................
Ga0184599_140898/774-849       GGAAGCUA...GAUGGGGAAACAUCAC..........................................UCA..............................................................................
Ga0316049_111814/256-181       GGAAGCUA...GAUGGGGAAACAUCAC..........................................UCA..............................................................................
Ga0316034_100493/254-174       GGAAGCUA...GCCAGGGAAACACCACU.........................................GUU..............................................................................
Ga0184600_118565/177-97        GGAAGCUA...GCCAGGGAAACACCACU.........................................GUU..............................................................................
Ga0184602_122864/595-515       GGAAGCUA...GCCAGGGAAACACCACU.........................................GUU..............................................................................
Ga0184596_126546/83-162        GGAAGCUA...GCCAGGGAAACACCACU.........................................GUU..............................................................................
Ga0184597_129399/366-446       GGAAGCUA...GCCAGGGAAACACCACU.........................................GUU..............................................................................
Ga0184576_117703/380-460       GGAAGCUA...GCCAGGGAAACACCACU.........................................GUU..............................................................................
Ga0184590_111002/143-223       GGAAGCUA...GCCAGGGAAACAUCACU.........................................GUU..............................................................................
Ga0247551_103383/322-242       AGAAGCUA...GCCAGGGAAACACCACU.........................................GUU..............................................................................
Ga0184597_111165/262-182       GGAAGCUA...GCCAGGGAAACACCACU.........................................GUU..............................................................................
Ga0184570_116436/278-198       GGAAGCUA...GCCAGGGAAACACCACU.........................................GUU..............................................................................
Ga0316037_169124/279-199       GGAAGCUA...GCCAGGGAAACACCACU.........................................GUU..............................................................................
Ga0247543_103170/244-164       GGAAGCUA...GUCAGGGAAACACCACU.........................................AUU..............................................................................
Ga0184589_120451/249-329       GGAAGCUA...GUCAGGGAAACACCACU.........................................GUU..............................................................................
Ga0247519_108375/286-206       GGAAGCUA...GCCAGGGAAACACCACU.........................................GUU..............................................................................
Ga0184587_105985/71-151        GGAAGCUA...GCCAGGGAAACACCACU.........................................GUU..............................................................................
Ga0184593_101086/682-762       GGAAGCUA...GCCAGGGAAACACCACU.........................................GUU..............................................................................
Ga0184602_121948/324-245       GGAAGCUG...GACAGGGAAACACCAUC.........................................AGUA.............................................................................
Ga0184597_110829/1090-1011     GGAAGCUG...GACAGGGAAACACCAUC.........................................AGUA.............................................................................
Ga0184578_129447/314-235       GGAAGCUG...GACAGGGAAACACCAUC.........................................AGUA.............................................................................
Ga0184588_113246/331-252       GGAAGCUG...GACAGGGAAACACCAUC.........................................AGUA.............................................................................
Ga0316051_1003245/138-56       GGAAGCUG...GACAGGGAAACACCAUC.........................................AGUAGUA..........................................................................
Ga0316046_122965/221-142       GGAAGCUG...GACAGGGAAACACCAUC.........................................AGUA.............................................................................
Ga0184600_105920/81-160        GGAAGCUG...GACAGGGAAACACCAUC.........................................AGUA.............................................................................
Ga0184603_125382/9-88          GGAAGCUG...GACAGGGAAACACCAUC.........................................UGUA.............................................................................
Ga0247550_100138/208-129       GGAAGCUG...GACAGGGAAACACCAUC.........................................AGUA.............................................................................
Ga0316049_122425/209-288       GGAAGCUG...GACAGGGAAACACCAUC.........................................AGUA.............................................................................
Ga0032354_1062690/2659-2586    GGAAGCAU...GCCAGGGAAACACCC...........................................UUUUU............................................................................
Ga0179955_1048341/123-51       GGAAGACC...GCCAGGGAAACACACG..........................................UAUUUGGAAC.......................................................................
Ga0138277_1023917/182-18       GGAAGACC...GCCAGGGAAACACACCGCUCAACGAUCCUAUC..........................UAUAAUUU...........................................................GACUGGCUCG..GA
Ga0138282_1020546/737-573      GGAAGAUC...GCCAGGGAAACAUGCUGCUCAACGAUCCUAUC..........................UAUAAUUU...........................................................GACUGGCUCG..GA
Ga0242641_1105635/265-351      GGAAGACG...GUGAGGGAAACACACCCGAGUC....................................ACUCG........................................................................GACU
Ga0242654_10795375/263-349     GGAAGACG...GUGAGGGAAACACACCUGAGUC....................................ACUCG........................................................................GACU
Ga0247554_113506/254-161       GGAAGUUA...UCCGGGGAAACACAUGAACGU.....................................AGCG..........................................................................AC.
Ga0265745_1000618/793-552      AGAAGAUUC..UCCAGGGAAACACAUCGGG.......................................UAUAUGGC........................................................................C
Ga0265742_1001052/322-80       AGAAGAUUC..UCCAGGGAAACACAUCGGG.......................................UUUAUGGC........................................................................C
Ga0310113_106868/972-730       AGAAGAUUC..UCCAGGGAAACACAUCGGG.......................................UAUAUGGC........................................................................C
Ga0265750_1009219/272-30       AGAAGAUUC..UCCAGGGAAACACAUCGGG.......................................UUUAUGGC........................................................................C
Ga0265740_1001378/1084-842     AGAAGAUUC..UCCAGGGAAACACAUCGGG.......................................UUUAUGGC........................................................................C
Ga0265752_100504/930-688       AGAAGAUUC..UCCAGGGAAACACAUCGGG.......................................UAUAUGGC........................................................................C
Ga0265747_100653/495-253       AGAAGAUUC..UCCAGGGAAACACAUCGGG.......................................UUUAUGGC........................................................................C
Ga0265757_104669/135-376       AGAAGAUUC..UCCAGGGAAACACAUCGGG.......................................UAUAUGGC........................................................................C
Ga0265742_1088739/51-292       AGAAGAUUC..UCCAGGGAAACACAUCGGG.......................................UAUAUGGC........................................................................C
Ga0265747_100509/1277-1036     AGAAGAUUC..UCCAGGGAAACACAUCGGG.......................................UAUAUGGC........................................................................C
Ga0265752_100314/1466-1225     AGAAGAUUC..UCCAGGGAAACACAUCGGG.......................................UAUAUGGC........................................................................C
Ga0213850_1450246/323-606      GGAAGAUG...GCCAGGGAAACACAUCGCAGGAGGC.........AGUGUAGGAUCCCCCCC.......GCCAG..........................................GGGGGGGG..CCAAAGCUCGUAAGAGCCACUCCU
Ga0157593_1101573/285-478      GGAAACUA...GCCAGGGAAACACAUGUAUAAAGGCUC.UUAGCCGCCUGCCCCGUGAGG.........GGGGGCUUGCCCAACACUC...............................UUUAGUGAGAAAAGCGCGAAAGGAACCUUGA
Ga0222728_1000125/1315-1238    AGAGGCGA...GCCAGGGAAACACUGUU.........................................UUA..............................................................................
Ga0242654_10000586/1273-1196   AGAGGCGA...GCCAGGGAAACACUGUU.........................................UAC..............................................................................
Ga0265756_100130/1310-1239     CGAAGCUA...GCCAGGGAAACACAA...........................................CUAGA............................................................................
Ga0307482_1008742/1212-1288    AGAGGCGA...GCCAGGGAAACACUAUC.........................................AUA..............................................................................
Ga0242654_10040657/1018-942    AGAGGCGU...GCCAGGGAAACACCAUC.........................................AAU..............................................................................
Ga0307484_100842/1194-1271     AGAGGCGA...GCCAGGGAAACACUAUC.........................................UAUU.............................................................................
Ga0307482_1009223/670-508      AGAAGUUG...GUCAGGGAAACACCUCUG.CC.....................................AUU...........................................................................GGC
Ga0307484_108094/186-350       AGAAGUUG...GUCAGGGAAACACCUCC.GC......................................AUAUUG.........................................................................GC
Ga0247514_114593/280-211       AGAAGCCA...GCCAGGGAAACACAUUC.........................................UUAAU............................................................................
Ga0184583_106441/296-227       AGAGGCUA...GCCAGGGAAACACAUCC.........................................GUAA.............................................................................
Ga0242654_10672177/244-165     GGAAGCAU...GCCAGGGAAACACAUAAC........................................CAAG.............................................................................
Ga0126318_10229384/609-688     GGAAGCAG...GCCAGGGAAACACUUCAC........................................GUA..............................................................................
Ga0247551_122551/29-115        GGAAGCAG...GCCAGGGAAACACUUC..........................................AUAU.............................................................................
Ga0316048_107802/239-324       GGAAGCAG...GCCAGGGAAACACUUC..........................................AUAU.............................................................................
Ga0242641_1002064/1203-1116    GGAAGCAC...GCCAGGGAAACACAUCC.........................................UCGUC............................................................................
Ga0307482_1026216/884-813      GGAAGCAU...GCCAGGGAAACACAUC..........................................UUCG.............................................................................
Ga0307482_1636410/270-341      GGAAGCAU...GCCAGUGAAACACAUC..........................................UUCG.............................................................................
Ga0307482_1016877/168-97       GGAAGCAU...GCCAGGGAAACACAUC..........................................GAAA.............................................................................
Ga0180107_1106188/424-495      GGAAGCAU...GCCAGGGAAACACAUC..........................................GAAA.............................................................................
Ga0307482_1000354/2063-1987    GGAAGCAU...GCCAGGGAAACACUUCA.........................................GUUA.............................................................................
Ga0184587_107783/167-95        AGAAGCAU...GCCAGGGAAACACAUCA.........................................UCA..............................................................................
Ga0184585_128240/467-390       GGAAGCAC...GCCAGGGAAACACGAGA.........................................UUUG.............................................................................
Ga0184596_102741/625-701       GGAAGUAU...GCCAGGGAAACACGAUC.........................................UCU..............................................................................
Ga0247539_100086/1763-1850     GGAAGACU...UCCAGGGAAACAUUGGU.........................................UUUAU............................................................................
Ga0184584_115572/267-354       GGAAGACC...UCCAGGGAAACAUUGGU.........................................UUUAU............................................................................
Ga0184579_105257/1735-1648     GGAAGACU...UCCAGGGAAACAUUGGU.........................................UUUAU............................................................................
Ga0307482_1023270/273-197      GGAAGCAU...GCCAGGGAAACACGAUC.........................................UCU..............................................................................
Ga0307482_1219330/19-95        GGAAGCAA...GCCAGGGAAACACUGU..........................................UUUAU............................................................................
Ga0242657_1207391/462-383      GGAAGCAC...GCCAGGGAAACACAUUAC........................................UUU..............................................................................
Ga0247520_104849/891-821       GGAAGUAC...GCCAGGGAAACACAAUC.........................................UUUAU............................................................................
Ga0247531_104861/1082-1012     GGAAGCAC...GCCAGGGAAACACAAUC.........................................UUUAU............................................................................
Ga0247518_137824/301-231       GGAAGCAC...GCCAGGGAAACACAAUC.........................................UUUAU............................................................................

                               ....>>>>...<<<<<.......<<<<<<<<<<<<<<<<<<<<<<<<<<<<<<<<<<<<<<<<<<<<<<.........................................>>>>>>>>>>>>>>>>>>>>>>>>>>>>>>>>>>>>>>>>
                               ....1222...22222.......2222222..............................1..1100...........................................0011..1..............................2
                               RGAAG●●●---R●●RGRGAAACAY●●●●○○○--------------------------------------●●●○○--------------------------------------------------------------------------○○
```

```
Ga0307482_1202605/470-395     GGAAGACU...UCCAGGGAAACACUGUG.........................................UAUC.............................................................................
Ga0307921_1021783/160-261     GGAAGAUU...GCCAGGGAAACAUGCGUCAGGAGUUU................................GGA......................................................................GAACUCC.
Ga0242657_1054283/541-620     AGAAGCAC...GCCAGGGAAACACAAUGUGU......................................GCAA...........................................................................AC
Ga0242647_1005188/349-360     AGAAGCAC...GCCAGGGAAACACAAUGUGU......................................GCAA...........................................................................AC
Ga0153880_1391790/62-140      GGAAGAAC...GGGGUGGAAACAUGCUUGG.......................................AAAG............................................................................C
Ga0153880_1379927/66-144      GGAAGAAC...GGGGUGGAAACAUGCUUGG.......................................AAAG............................................................................C
Ga0307482_1023665/853-778     GGAAGACG...ACCAGGGAAACAUGCCG.........................................ACUCC............................................................................
Ga0242646_1047905/92-182      GGAAGACC...GUGAGGGAAACAUGCCCC........................................ACGCC............................................................................
Ga0307921_1024041/406-334     GGAAGAAG...ACGAGGGAAACAUGCCGC........................................AAUA.............................................................................
Ga0307919_1039507/513-440     GGAAGAAG...ACGAGGGAAACAUGCCGC........................................AAUA.............................................................................
Ga0307919_1084008/308-378     GGAAGAAG...GCGAGGGAAACAUGCCCC........................................ACA..............................................................................
Ga0247525_114510/199-318      GGAAGACA...ACCAGGGAAACACACCGG........................................CAAG.............................................................................
Ga0184603_107262/477-596      GGAAGACA...ACCAGGGAAACACACCGG........................................CAAG.............................................................................
Ga0184585_151549/495-376      GGAAGACA...ACCAGGGAAACACACCGG........................................CAAG.............................................................................
Ga0310119_113172/381-306      GGAAGACU...GCCAGGGAAACACACCC.........................................UCUU.............................................................................
Ga0310114_133004/56-132       GGGAGACU...GCCAGGGAAACACACCC.........................................CUCUC............................................................................
Ga0184603_148109/996-920      GGAAGACU...GCCAGGGAAACACACCC.........................................CUUUC............................................................................
Ga0184599_110049/275-349      GGAAGACU...GCCAGGGAAACACACC..........................................CUUGC............................................................................
Ga0184587_132427/565-485      GGAAGACU...GCCAGGGAAACACACCCCC.......................................CAGAA...........................................................................G
Ga0265756_135986/152-290      GGAAGAAC...GCCAGGGAAACACACCAC..CAUUUAGGUUGCCUG.CU....................AAUAUUUCA....................................................AGACAGG..ACAUAAAUACA
Ga0247519_104852/800-730      GGAAGCAC...GCCAGGGAAACACAAC..........................................GAAA.............................................................................
Ga0213850_1274705/160-417     GGAAGAUG...GUGGGGGAAACACACAACAACG.GC.................................GCACUCAAA.................................................................GUACG.U
Ga0265747_100454/1395-1324    GGAAGCUA...ACCAGGGAAACAUAUCC.........................................AACA.............................................................................
Ga0247519_118271/334-405      GGAAGCUA...ACCAGGGAAACAUAGACU........................................GACAA............................................................................
Ga0242654_10135580/213-336    GGAAGCUG...GCUAGGGAAACACACC..........................................UAAA.............................................................................
Ga0184576_110410/277-348      GGAAGCAG...GGCAGGGAAACACAGU..........................................UUCU.............................................................................
Ga0247535_105396/277-348      GGAAGCAG...GGCAGGGAAACACAGU..........................................UUCU.............................................................................
Ga0265758_104558/436-365      GGAAGCUA...GCCAGGGAAACACACUA.........................................AUA..............................................................................
Ga0247526_117664/434-499      GGAAGCUG...GCGAGGGAAACACACU..........................................AUAU.............................................................................
Ga0316037_123461/289-368      GGAAGCUU...GCCAGGGAAACACAUGAU........................................AACA.............................................................................
Ga0247543_100863/642-721      GGAAGCUU...GCCAGGGAAACACGAUAC........................................CUUU.............................................................................
Ga0247521_124040/388-309      GGAAGCUU...GCCAGGGAAACACAAUAC........................................CUUU.............................................................................
Ga0184589_120335/11-90        GGAAGCUU...GCCAGGGAAACACAAUAC........................................CUUU.............................................................................
Ga0247518_108423/758-837      GGAAGCUU...GCCAGGGAAACACAAUAC........................................CUUU.............................................................................
Ga0247531_104608/1256-1177    GGAAGCUU...GCCAGGGAAACACAAUAC........................................CUUU.............................................................................
Ga0265750_1365815/134-55      GGAAGCUU...GCCAGGGAAACACAAUAC........................................CUUU.............................................................................
Ga0184597_121517/262-183      GGAAGCUU...GCCAGGGAAACACAAUAC........................................CUUU.............................................................................
Ga0265747_102820/448-376      GGAAGCUU...GCCAGGGAAACACAUGAU........................................CACG.............................................................................
Ga0247554_105388/388-316      GGAAGCUU...GCCAGGGAAACACAUGAU........................................UACG.............................................................................
Ga0307482_1015330/1021-1093   GGAAGCUA...GCCAGGGAAACACACC..........................................AAA..............................................................................
Ga0184600_102725/60-135       UGAAGCGA...GCCAGGGAAACAUCACA.........................................GCAA.............................................................................
Ga0184602_122974/190-265      UGAAGCGA...GCCAGGGAAACAUCACA.........................................GCAA.............................................................................
Ga0316038_112263/442-356      GGAAGCAA...GCCAGGGAAACACCA.UAC.......................................AUAUGCGC........................................................................G
Ga0307484_161007/136-39       GGAAGCUA...GGCAGGGAAACAUCGUC.........................................UAUA.............................................................................
Ga0307482_1033688/516-422     GGAAGCUA...GGCAGGGAAACAUCGUC.........................................UAUA.............................................................................
Ga0316031_132099/178-294      GGAAGAGU...CCCAGGGAAACACCGCA.........................................GAA..............................................................................
Ga0213850_1220553/385-463     GGAAGUCA...GGCAGGGAAACACUCC..........................................GCGAAG...........................................................................
Ga0180107_1307618/257-174     GGAAGACU...GUGAGGGAAACAUGCCUUCUUC....................................UUG..........................................................................GAGG
Ga0265747_119090/8-84         GGAAGACU...GCAAGGGAAACAUGCC..........................................AUAC.............................................................................
Ga0242646_1115622/191-109     GGAAGCUG...GGCAGGGAAACAUCGCA.........................................CCUAU............................................................................
Ga0184570_114412/209-338      GGAAGACC...GCAAGGGAAACAUUAGA..GAUUUGAACUGGAGAUUCGAG..................AUCA.......................................................UUCCAA..UCCAG...GGAUCA
Ga0265757_143567/145-70       GGAAGACG...GCAAGGGAAACAUCGAGGAC......................................GAA............................................................................GU
Ga0184568_109812/391-283      GGAAGACU...GUGAGGGAAACAUACGACGAG.AGAAGAGCUAGUG.......................GUAG............................................................CACGA.CUCUAUGAC..
Ga0184568_104528/922-1000     GGAAGACU...AUGAGGGAAACAUGGAGUU.......................................CUAACAA.........................................................................A
                              ....>>>>...<<<<<.......<<<<<<<<<<<<<<<<<<<<<<<<<<<<<<<<<<<<<<<<<<<<<<........................................>>>>>>>>>>>>>>>>>>>>>>>>>>>>>>>>>>>>>>>>>
                              ....1222...22222.......2222222..............................1..1100..........................................0011..1...............................2
                              RGAAG●●●---R●●RGRGAAACAY●●●●●○○--------------------------------------●●●●○--------------------------------------------------------------------------○○
```

alignment positions 601 · · · 620

```
Ga0247541_102964/376-269      UGAUGAGCACAUUACUCUCU
Ga0182091_1018330/100-226     GCGAAGGCACAUUACCCAGU
Ga0316035_118720/195-42       CAUAAAGAAAAUUAACUCAA
Ga0179944_1246019/41-131      UUAUGGGUAAAUUAACUUAG
Ga0265747_125480/96-202       AUAAAAGUACAUUAACUUAU
Ga0265757_115889/161-280      UUGGCAGCACAUUACCAUGU
Ga0180107_1031754/4443-4551   GUAUGAGAAAAUUAGCCCAU
Ga0180107_1229893/426-318     CUUCGAGCACAUUAACUCUC
Ga0222728_1038816/522-635     AUGCCGGCACAUUACUGUCU
Ga0179955_1195671/481-563     UCUGAGGCACAUUACUUACC
Ga0265751_100560/274-382      UGGUAUGCACAUUACCCAGA
Ga0265751_124015/160-14       UACAAAGCACAUUAACCACC
Ga0316029_122532/20-172       GCUCAGGCAAAUUACUCAUC
Ga0316031_106349/204-348      ACUCAGGCAAAUUACUCAUC
Ga0265759_136872/100-7        ..GCAGGGAAAUUAACUGAC
Ga0138291_1027114/229-62      GCGAAAGCACAUUAACUGGU
Ga0138291_1177333/356-213     UACAAAGCACAUUAACUGUU
Ga0079074_1152161/13-203      AUAAUAGCAUAUUAACUGAC
Ga0242657_1353977/135-311     ACUAUAGCACAUUAUCCUCA
Ga0255293_1180932/373-295     ..GAGAGCAUAUUACCGCGC
Ga0307482_1147486/107-4       AUUACGGUACCUUACCCGUG
Ga0242647_1001226/1011-1115   ACAGAAGCACAUUACCCAGG
Ga0316032_128094/136-241      AUUGUGGCACAUUAACCAUG
Ga0184598_118707/299-404      UCCAUGGCACAUUAACCAAG
Ga0213850_1470830/321-202     UCUUUGGCACAUUAACUGAC
Ga0307482_1003156/2075-2177   .CAUGAGCACAUUACCCGAC

                              >>>>>>.........>>>>>
                              222222.........22222
                              ●●●●●RGYAYAUUA●CY●●Y
```

```
Ga0307482__1000291/592-698          GUAUGAGCACAUUACCCGAC
Ga0247532__117056/43-239            CAUGUGGCACAUUACCCUAG
Ga0184582__120088/374-570           CAUGUGGCACAUUACCCUAG
Ga0265742__1013613/579-453          CGCGUAGCACAUUACUCGUC
Ga0265748__110851/279-405           CGCGUAGCACAUUACUCGUC
Ga0307482__1000262/4292-4390        UUCCUGGCACAUUAACUCCC
Ga0307484__100453/182-280           UUCCUGGCACAUUAACUCCC
Ga0247538__120078/87-186            UCUGUGGUACAUUAACUUCC
Ga0307484__109934/368-484           CUUGUAGCAUAUUAACUUAG
Ga0184572__111224/187-319           UUGAUAGCACAUUAACUGAC
Ga0265744__102237/225-365           CAGUUGGCACAUUACCCGAU
Ga0265758__100384/785-1040          CCGGUGGUAUAUUACCCACU
Ga0265753__1005391/776-521          CCGGUGGUAUAUUACCCACU
Ga0265759__100623/780-1036          CCGGUGGUAUAUUACCCACU
Ga0247535__113123/229-119           CGUGAGGCACAUUACUCAGG
Ga0316036__112404/482-376           CGUGAGGCACAUUACUCAGG
Ga0247515__120448/91-178            CAAAAGGCAAAUUAUCCAAG
Ga0247518__120275/202-289           CAAAAGGCAAAUUAUCCAAG
Ga0307482__1000311/1469-1363        CACAUGGCAUAUUACCCCUC
Ga0184603__150346/629-518           CUAACGGUACAUUACCUCUC
Ga0307484__102855/507-659           GCUGAGGUACAUUACCUUCG
Ga0316029__101396/632-732           CCUAAGGCACAUUACCCGUC
Ga0184582__100659/424-334           GGAGCAGCACAUUAACCAUC
Ga0265754__1040230/51-148           ..UGCAGCACAUUAACCUUC
Ga0316039__120091/354-252           CGUGAGAGAAAUUAACUCAU
Ga0307921__1050750/452-358          .ACUAAGCACAUUAACCCAC
Ga0265759__141590/154-258           GCAUCGGCACAUUACCCAAC
Ga0184602__130775/1275-1431         UACGUAGCACAUUAACCUGG
Ga0213850__1052387/326-212          AGUCUGGCACAUUACCCAGG
Ga0213850__1393213/656-542          AGUCUGGCACAUUACCCAGG
Ga0184578__112042/249-140           UCUAGAGCACAUUAACUCCU
Ga0316033__100036/207-98            UCUAGAGCACAUUAACUCCU
Ga0247534__100007/116-225           UCUAGAGCACAUUAACUCCU
Ga0184569__109084/220-111           UCUAGAGCACAUUAACUCCU
Ga0206355__1134705/241-332          UUGUCGGCAUAUUAACUCAC
Ga0265754__1007706/448-552          UUAUAAGCACAUUACCCGAC
Ga0213850__1056422/14-139           CCGCGGGCACAUUACUGUCC
Ga0180107__1033011/786-679          UGUGGAGAAGAUUACCCGGA
Ga0307483__1000720/1178-1064        GUUGAGGUACAUUAACUUCC
Ga0265755__100088/1451-1216         CAGCAGGUAUAUUACCUUCU
Ga0310119__125223/193-319           AUUUCGGCACAUUACCUCAC
Ga0265754__1037910/322-179          GUUGUAGCACAUUAACCAGG
Ga0316053__162144/16-125            CGAGAAGCACAUUACCUAUC
Ga0242654__10007932/343-135         CUCGUAGUAUAUUACCUCGA
Ga0153880__1315932/142-46           ..GGCAGCACAUUAACCGCU
Ga0265750__1009277/794-905          UGAAAAGCACAUUAACUGGA
Ga0265756__100928/447-557           UGAAAAGCACAUUAACUGGA
Ga0265745__1004075/525-659          UGAAAAGCACAUUAACUGGA
Ga0138291__1147840/370-201          GCGAAAGCACAUUAACUGGU
Ga0138291__1027295/231-62           GCGAAAGCACAUUAACUGGU
Ga0184602__109114/416-317           A.AGUAGAACAUUAACUGAC
Ga0153880__1354682/355-454          A.AGUAGAACAUUAACUGAC
Ga0242649__1016743/464-237          UGAGAGGCACAUUAACUGGC
Ga0179943__1118543/65-150           UUUGUAGUAAAUUACCCAAG
Ga0184568__108419/492-395           UCUAAGGUACAUUACCCGUU
Ga0265756__117960/316-418           UUGUCGGCACAUUACCUCCU
Ga0265759__100202/139-258           CUGGUGGCAUAUUAACCUGG
Ga0265748__107452/280-387           CAUAGGGCACAUUAACUCCU
Ga0307484__149222/136-234           CUUGUAGUACAUUAACCAGU
Ga0316038__107364/55-195            CACUGAGCAUAUUACCUGAC
Ga0184593__123391/157-297           CACUGAGCAUAUUACCUGAC
Ga0242652__1159268/284-150          CAUUCAGCACAUUACCCAAC
Ga0310117__113093/724-511           GACCAGGUACAUUAACGUCU
Ga0179944__1119724/70-159           GACGUAGCACAUUAACUGGC
Ga0316036__104840/706-578           CUGUUGGCACAUUAACUGAG
Ga0247538__106858/350-156           G..UGGGCACAUUACCCAGU
Ga0180107__1215312/308-184          CAGGUGGCACAUUAACCUAG
Ga0247534__100003/3147-3245         CUAUGAGCACAUUACCCAUC
Ga0247519__101377/1717-1907         UGAAGAGCACAUUACCUGCU
Ga0184588__143621/642-452           UGAAGAGCACAUUACCUGCU
Ga0265758__100017/4028-4134         CCAAAAGGAAAUUAUCCCAU
Ga0265751__102572/231-125           CCAAAAGGAAAUUAUCCCAU
Ga0310106__100226/1503-1738         CAACAGGCAUAUUACCUUCG
Ga0184596__112078/314-191           .UGGUGGCACAUUACUUGGA
Ga0247528__108647/438-562           UUGGUGGCACAUUACUUGGA
Ga0184602__108471/49-173            .UGGUGGCACAUUACUUGGA
Ga0316031__112736/216-74            UUUUCGGUACAUUACUCUCC
Ga0184575__106581/174-318           UCCUCGGUACAUUACUCUCC
Ga0184582__129189/472-619           C.CGUAGCACAUUACCCUAG
Ga0180052__116799/419-501           UCUAUGGCACAUUACCCGUC
Ga0179955__1013331/439-311          CCGCGGGCACAUUACUGUCC
Ga0157569__1058012/474-586          GGGCGGGCACCUUACCCGAG
Ga0157562__1164332/672-560          GGGCGGGCACCUUACCCGAG
Ga0265746__1084801/20-158           CUUGUUGCAUAUUACCCGAC
Ga0265752__102479/223-81            CUUGUUGCAUAUUACCCGAC
Ga0265756__107066/520-660           CUUGUUGCAUAUUACCCGAC
Ga0247541__102884/37-209            ..GCUGGCACAUUACCCGGG
Ga0247516__125012/316-428           AGACUGGGAUAUUACCCGGA
Ga0213850__1071966/331-222          UGCAUAGCACAUUAACUUGC

                                    >>>>>>.........>>>>>
                                    222222.........22222
                                    ○●●●●RGYAYAUUA●CY●●Y
```

```
Ga0257141__1100114/291-412          AAGGAGGAAAAUUAACCUGG
Ga0265740__1020297/298-150          AAAGCAGCACAUUAACCGUU
Ga0307482__1064439/425-316          CUAAAGGAAAAUUAACCAUC
Ga0138292__1031932/14-100           GAUACGGCACAUUACCGCUU
Ga0138291__1022124/340-254          CAUACGGCACAUUACCGCUU
Ga0184582__127657/238-384           CUCAUGGGACAUUACCUAGA
Ga0184570__113141/244-341           AUAAUAGCACAUUACCUAGC
Ga0310119__152175/242-389           CACGUAGCACAUUAACUUGC
Ga0184576__119948/531-664           CGAAUGGCACAUUACCCGAA
Ga0316034__129624/151-284           CGAAUGGCACAUUACCCGAA
Ga0307482__1134687/287-181          UCGGCGGCACAUUAGCUUCC
Ga0307484__104189/399-292           UCGGCGGCACAUUAGCUUCU
Ga0138282__1242808/325-475          GCGGCGGUACAUUAACUGCC
Ga0180047__1081041/176-42           AGGUCGGCACAUUACCCCUC
Ga0265752__100023/1618-1523         CUACGAGCACAUUACCCAUC
Ga0242649__1051170/34-143           UAUAAAGAAAAUUAACCUCA
Ga0242654__10030584/1049-1243       AGUGUAGCAUAUUACCCGGA
Ga0307484__102966/727-825           AGCAAAGCACAUUAACUCCU
Ga0307921__1020571/133-221          .GUACAGCACAUUAACCCAC
Ga0184573__113483/125-229           UUGGCGGAAAAUUACCCAUU
Ga0184576__116834/47-151            UUGGCGGAAAAUUACCCAUU
Ga0184595__109216/770-611           AGUGUAGCACAUUACCCGUU
Ga0316040__154359/245-85            AGUGUAGCACAUUACCCGUU
Ga0184595__116949/555-395           AGUGUAGCACAUUACCCGUU
Ga0247553__104889/634-563           ..UGCAGCACAUUACCCAUA
Ga0316046__107255/463-562           .GUGGAGCACAUUACCCAGU
Ga0307484__102693/441-627           UGAGCGGCACAUUAACCCGG
Ga0184568__114257/261-413           CUGGUAGCACAUUACCCAUC
Ga0316047__106478/13-108            GCCCAAGUACAUUAACCUAC
Ga0242654__10024002/775-526         .CGGUAGCACAUUAACUCGC
Ga0316039__101269/650-841           GUCUUGGCACAUUAACUCUC
Ga0265743__102316/615-423           GUCUUGGCACAUUAACUCUC
Ga0210323__1072445/691-543          UGUCGGGCACAUUACCCGAC
Ga0224712__10069322/1099-1357       .CCUAGGUACAUUAACUCAC
Ga0213850__1371453/186-298          AAGGAGGUACAUUAUCCAGG
Ga0153880__1056505/290-430          UUUGUAGCACAUUAACUGAC
Ga0179940__1033563/296-410          UUGGUAGCACAUUAACUUAC
Ga0265743__143797/241-325           .GUCGGGCACAUUACCCAGC
Ga0242654__10006748/25-247          GGAGUGGUACAUUAACUCUU
Ga0138278__1149859/65-166           AG.AUAGCAUAUUACCCCAC
Ga0138278__1149142/191-91           AG.AUAGAACAUUACCCCAC
Ga0138277__1147417/463-362          AG.AUAGCAUAUUACCCCAC
Ga0138278__1065026/314-214          AG.AUAGCAUAUUACCCCAC
Ga0138277__1089473/408-304          UCGAUAGAACAUUACCCCAC
Ga0138277__1022592/399-305          UUGACAGAACAUUACCCCAC
Ga0157559__1099797/360-209          AUUGAAGCACAUUAACUGGU
Ga0157572__1089974/360-209          AUUGAAGCACAUUAACUGGU
Ga0242646__1007472/516-317          UACGCAGCACAUUACCCGGG
Ga0184583__104740/342-477           CGAGUAGCACAUUACCCGAA
Ga0242654__11312609/202-83          GCAAUGGCACAUUACCCGUC
Ga0184568__109070/428-286           CCGGUGGUACAUUAACCUGG
Ga0224712__10002011/1564-1454       CUGGUGGAAUAUUAACUCCU
Ga0222728__1000095/4027-4133        CUGAUAGUAAAUUAACCUCC
Ga0307482__1068632/529-339          UAGAGAGUAAAUUACCCUGC
Ga0157560__1170105/128-245          .CUAGAGAAAAUUAACUCAC
Ga0316031__107021/319-214           UCAAGAGUAAAUUAACUCCC
Ga0316047__100015/734-626           CUACUGGAAAAUUAACUCUC
Ga0247533__100162/1643-1535         CUACUGGAAAAUUAACUCUC
Ga0180108__1209503/1932-2029        CAGUUGGCACAUUAACCUUC
Ga0265753__1009881/576-819          CCGGUGGCACAUUAACCUGG
Ga0265741__101522/997-754           CCGGUGGCACAUUAACCUGG
Ga0184583__103998/587-740           CUAGUAGCAUAUUAACCGAC
Ga0247521__125910/353-214           AUCCAGGCACAUUAACUCAC
Ga0184569__106253/218-80            AUCCAGGCACAUUAACUCAC
Ga0316039__119781/347-457           UCAUCUGCACAUUAACUCCC
Ga0184572__112077/359-259           UCAUGAGUACAUUACCCAGU
Ga0257141__1118781/153-31           GGAGGGGAAAAUUAACCUGG
Ga0257136__1059838/549-429          GGAGGGGAAAAUUAACCUGG
Ga0138283__1149501/173-59           GCCGCGGCACAUUACCCGGC
Ga0184597__126105/545-362           UAAGGGGUACAUUAACUGGA
Ga0213850__1245299/581-409          CUCGCGGCACAUUAACUGAC
Ga0179948__1254202/198-296          ACGCGGGCAAAUUACCUAGC
Ga0180108__1093556/523-685          GGGGUGGCAUAUUAACUCUC
Ga0180107__1321405/371-533          GAGGUGGCAUAUUAACUCUC
Ga0242641__1001197/544-746          GAGGUGGCACAUUACCCGGU
Ga0184597__115944/155-80            .AGCUAGUAAAUUAACUACC
Ga0184595__100702/414-262           CUAGUAGCACAUUAACCGAC
Ga0184601__118759/683-531           CUAGUAGCACAUUAACCGAC
Ga0184592__125105/67-219            CUAGUAGCACAUUAACCGAC
Ga0257141__1089020/433-224          GCGUUAGCACAUUAACUGGU
Ga0265743__103007/262-368           .AUGCAGUACAUUAACUGGG
Ga0222728__1000095/4541-4435        CCUGUAGCACAUUAACUGGA
Ga0247537__109082/42-148            CCUAUAGCACAUUAACUGGA
Ga0247533__100162/1117-1223         CCUAUAGCACAUUAACUGGA
Ga0265757__100063/1489-1724         CAACAGGCACAUUACCUUCG
Ga0265747__118257/34-269            CAACAGGCACAUUACCUUCG
Ga0265745__1000046/1698-1933        CAACAGGCACAUUACCUUCG
Ga0115595__1153837/320-197          UACAGAGCACAUUACCCGAC
Ga0242657__1026087/460-322          GUGCUAGCACAUUACCCAGU

                                    >>>>>>.........>>>>>
                                    222222.........22222
                                    ●●●●●RGYAYAUUA●CY●●Y
```

```
Ga0265751__100417/1480-1588      GUUCGGGCAAAUUAUCCGUC
Ga0265756__100485/1108-1000      GUUCGGGCAAAUUAUCCGUC
Ga0265751__100393/1594-1698      CUGGUAGCAUAUUAACCUCC
Ga0307921__1011071/674-791       CUUGUAGCACAUUAACUCGC
Ga0307919__1094420/160-285       CUUGUAGCACAUUAACUCAC
Ga0316034__107719/314-205        UUGACAGCAUAUUACCUCCC
Ga0265742__1065119/361-273       CCGGCAGCACAUUACGUAGC
Ga0180107__1164033/560-431       .AGGCGGCACAUUACCUAGA
Ga0242641__1001148/1456-1256     CGGCUGGUAUAUUACCCGGA
Ga0307482__1009758/562-669       CUGACAGCAUAUUAACUCCC
Ga0247527__109126/592-695        UCUGGAGCACCUUACCUGAC
Ga0247519__111615/148-45         UCUGGAGCACCUUACCUGAC
Ga0247513__116034/391-494        UCUGGAGCACCUUACCUGAC
Ga0213850__1290372/434-275       UGUGUGGCACAUUACCCACC
Ga0138276__1121602/329-410       UGGCCAGCAUAUUACCCUGC
Ga0138278__1041650/183-102       .GGCCAGCAUAUUACCCUGC
Ga0138277__1137481/570-489       .GGCCAGCAUAUUACCCUGC
Ga0138278__1041788/293-378       UGGCCAGCAUAUUACCCUGC
Ga0138277__1142657/273-361       UGGCCAGCAUAUUACCCUGC
Ga0138275__1223952/371-283       UGGCCAGCAUAUUACCCUGC
Ga0213850__1450246/199-116       GCGUUAGCACAUUACCUCUC
Ga0213850__1307525/101-186       ACGUUAGCAUAUUACCUCUC
Ga0265756__118952/425-318        CCACGGGAAAAUUACCCGGC
Ga0180107__1244437/408-507       UCAUCAGCACAUUACCCCUC
Ga0265742__1051722/222-295       .GUCCGGAAAACUAACUUCC
Ga0242657__1051800/416-321       UCUGUAGAAAAUUAACCAUG
Ga0307484__102966/353-250        UCCAUGGGACAUUAACCAGA
Ga0184568__108419/50-147         AACAGAGCACAUUAACUCUC
Ga0184568__103677/484-581        AACAGAGCACAUUAACUCUC
Ga0307484__102709/562-403        UCGGUAGCACAUUACCCAUC
Ga0127503__10678853/419-333      .AGCAGGCACAUUAACUCAC
Ga0307919__1065410/76-162        .AGCAGGCACAUUAACUCAC
Ga0242652__1038498/365-470       GU.CAGGCACAUUAACUCUC
Ga0307484__101264/723-941        CAGGUGGCACAUUACCCAAC
Ga0247534__101560/228-122        UCACUGGCACAUUACCCGUC
Ga0307484__109931/110-215        GUAGAGGCACAUUAACUAGC
Ga0184595__116681/414-266        CGAGUAGCACAUUACCCGAU
Ga0265749__100155/962-1187       AGUGUAGCACAUUACCCGAC
Ga0310115__109341/244-400        AGUGUAGCACAUUACCCGAU
Ga0310111__106996/679-905        AGUGUAGCACAUUACCCGAU
Ga0265756__101315/275-501        AGUGUAGCACAUUACCCGAU
Ga0265751__100874/820-594        AGUGUAGCACAUUACCCGAU
Ga0265744__120119/40-266         AGUGUAGCACAUUACCCGAU
Ga0310102__105010/226-452        AGUGUAGCACAUUACCCGAU
Ga0247534__103639/475-250        AGUGUAGCACAUUACCCGAU
Ga0265741__101833/434-208        AGUGUAGCACAUUACCCGAU
Ga0242647__1002114/801-1026      AGUGUAGCACAUUACCCGAC
Ga0265752__104585/383-158        AGUGUAGCACAUUACCCGAC
Ga0310107__109330/1076-850       AGUGUAGCACAUUACCCGAU
Ga0115595__1051440/308-419       AUCGUAGUACAUUAACUUCC
Ga0115594__1054328/389-276       AUCGUAGUACAUUAACUUCC
Ga0307482__1048753/383-528       CGAGUAGCACAUUACCCGUC
Ga0307480__1006257/684-599       GUGCAGGCACAUUACCUAGC
Ga0184595__116802/212-325        ...UGGGCAUAUUACCCGAC
Ga0307919__1071894/312-31        .CUACGGUAUAUUAACUCAC
Ga0307921__1036773/354-73        .CUACGGUAUAUUAACUCAC
Ga0307482__1023665/889-1191      ACGACGGUACAUUAACCCAC
Ga0180108__1022680/3902-3998     CAGAUGGUACAUUACCCUAC
Ga0184599__137402/105-300        .UGGUAGUACAUUACCCGAC
Ga0157569__1080558/455-605       .UACCAGCACAUUACCUGCC
Ga0184601__113231/247-149        CUCGUGGUACAUUACCCGGC
Ga0242652__1002960/413-574       GCACGGGCACAUUACCCAGG
Ga0307484__101303/704-866        GCACGGGCACAUUACCCAGG
Ga0242641__1025149/251-89        GCACGGGCACAUUACCCAGG
Ga0307482__1023050/594-496       CUAUGAGUACAUUACCCACC
Ga0307484__100434/1330-1210      CGUGAGGUACAUUAACCUAC
Ga0153880__1432604/289-395       U.UCUGGCACAUUAACCCGC
Ga0157559__1128929/201-87        ACGCGGGCACAUUACCCUAC
Ga0157560__1113202/155-269       GCGCGGGCACAUUACCCUAC
Ga0157566__1012736/497-384       GCGCGGGCACAUUACCCUAC
Ga0157579__1155702/323-436       GCGCGGGCACAUUACCCUAC
Ga0157562__1157806/189-74        GCGCGGGCACAUUACCCUAC
Ga0180047__1032966/152-37        GCGCGGGCACAUUACCCUAC
Ga0307484__166952/62-163         UCAGUGGCACAUUACCUCCC
Ga0005851__1178990/477-379       .GGAUGGUACAUUAACUCAC
Ga0153880__1191383/455-340       UCAGUAGUAAAUUAACUUUC
Ga0213850__1282564/267-350       ACGGCGGGACAUUACCCGAC
Ga0315817__133437/498-413        ACGGCGGGACAUUACCCGAC
Ga0315826__123252/262-347        ACGGCGGGACAUUACCCGAC
Ga0242657__1008904/1267-1104     CAUGUGGUACAUUACCCGAU
Ga0222728__1009902/1182-968      .CAACGGCACAUUAACUCAC
Ga0115594__1106220/441-334       UCAGCAGCACAUUAGUCGGC
Ga0180107__1307934/226-315       AUCGUGGUACAUUAACCGAU
Ga0184577__115871/202-369        CGGUAAGGAUAUUAACUCGG
Ga0206356__11590518/224-68       AUGGAGGUACAUUAACUUGC
Ga0222728__1062510/166-66        .UGAGAGAAUAUUAACUCAC
Ga0316029__101202/440-551        .CACGAGCACAUUAACUGGC
Ga0307482__1017422/1387-1206     AUCACGGCACAUUAACUGGG
Ga0307484__100889/412-594        AUCACGGCACAUUAACUGGG

                                 >>>>>>.........>>>>>
                                 222222.........22222
                                 ○●●●●RGYAYAUUA●CY●●Y
```

```
Ga0255062_11230321/200-110       CGAGUAGCACCUUAACUGGC
Ga0255059_10691632/166-76        CGAGUAGCACCUUAACUGGC
Ga0265744_100073/1235-1401       ACAGGAGCACAUUAACUGGA
Ga0265757_113703/332-416         UUAACAGCACAUUAACUUGG
Ga0265756_158829/72-188          AAAACGGUACAUUACCUGGA
Ga0184597_100195/107-221         AUGGUGGCACAUUAACUGUC
Ga0184594_121503/721-607         AUGGUGGCACAUUAACUGUC
Ga0184582_121032/1005-1119       AUGGUGGCACAUUAACUGUC
Ga0184602_104873/671-785         AUGGUGGUAUAUUAACUGUC
Ga0184589_132372/822-708         AUGGUGGCAUAUUAACUGUC
Ga0184580_125558/64-178          AUGGUGGUAUAUUAACUGUC
Ga0184596_101726/395-281         AUGGUGGCAUAUUAACUGUC
Ga0247541_109256/178-292         AUGGUGGCAUAUUAACUAUC
Ga0184584_108444/253-366         GAGUCGGUAAAUUAACUGUC
Ga0184582_124913/742-629         GAGUCGGCACAUUAACUGUC
Ga0184600_135218/318-205         GAGUCGGCAUAUUAACUGUC
Ga0247541_100074/611-498         GAGUCGGCACAUUAACUGUC
Ga0247551_101122/731-844         GAGUCGGCACAUUAACUGUC
Ga0247526_101949/462-575         GAGUCGGCACAUUAACUGUC
Ga0247524_101842/357-244         GAGUCGGCACAUUAACUGUC
Ga0247519_108765/580-693         GAGUCGGCAUAUUAACUGUC
Ga0180107_1134582/382-542        CCACAGGCACAUUAACUGCG
Ga0265747_125301/405-306         AUUGUAGUACAUUAACUGGA
Ga0157593_1101573/244-147        .UGAGAGAAAAUUACCCACC
Ga0184599_121568/40-155          .UGUGAGAAAAUUACCCACC
Ga0316040_116783/379-494         .UGUGAGAAAAUUACCCACC
Ga0157560_1091679/440-538        .CCUCCGAAAAUUACCCCAC
Ga0222728_1165579/392-259        GAGUGUGCAUAUUAACUGAC
Ga0224712_10601975/393-502       CUGAAAGGAAAUUAACUCUC
Ga0224712_10010317/1973-2082     CUGAAAGGAAAUUAACUCUC
Ga0307482_1048660/422-625        CCAGUAGCACAUUAACUGAC
Ga0179934_1155244/114-26         CGAGCAGCACAUUAACUCAC
Ga0307484_101172/602-495         CUUGAGGCACAUUAACUUCC
Ga0242654_10068518/113-218       CUUGAGGCACAUUAACUUCC
Ga0242647_1002406/915-1021       CUUGAGGCACAUUAACUUCC
Ga0222729_1010500/11-117         CUUGAGGCACAUUAACUUCC
Ga0307482_1202605/45-154         UCUCUAGCACAUUACCUUCC
Ga0184603_139562/141-280         AAAUCAGAAAAUUAACCCGC
Ga0242646_1001122/826-995        UGGGUAGCACAUUAACCGGA
Ga0310107_144222/327-436         CGACGUGCACCUUACCCGGA
Ga0265751_124807/392-283         CGACGUGCACCUUACCCGGA
Ga0184572_105168/388-281         CCAAUGGUACAUUAACCAAC
Ga0316036_127178/124-231         CCAAUGGUACAUUAACCAAC
Ga0222728_1003482/1262-1107      CGGUUGGCACAUUACCUCAC
Ga0265743_100639/33-191          ACAGUAGCACAUUAACUGGA
Ga0307484_105374/518-723         ACAGUGGCACAUUACCCGAU
Ga0265742_1015608/410-491        UUUGCAGCACAUUAACGCGC
Ga0316030_102139/629-482         GCUCUGGCAUAUUAACUUCC
Ga0265749_106268/131-212         GGCCGGGGAGACUAACUCAC
Ga0184573_100752/140-46          GAUUGUGCACAUUAACUGGG
Ga0157560_1010344/357-456        UGUGUGGAAAAUUACCCGAU
Ga0157593_1006201/239-338        UGUGUGGAAAAUUACCCGAU
Ga0180047_1087048/281-180        UGUGUGGAAAAUUACCCGAU
Ga0157565_1016893/233-334        UGUGUGGAAAAUUACCCGAU
Ga0184568_100511/100-195         UGUCGAGCAUAUUAACUGCC
Ga0265759_114856/370-262         CUCUCAGCACAUUAACUGAC
Ga0222729_1080556/362-213        UUGUUGGCACAUUAACUCGU
Ga0247541_102707/328-184         ACGUUAGCACAUUAACUGGC
Ga0307921_1020694/406-483        AUGAAGGUACAUUAACUCAC
Ga0307919_1034281/527-451        AUGAAGGUACAUUAACUCAC
Ga0184568_108869/934-1072        GGAGUGGCACAUUACCCGAU
Ga0206356_10036221/150-306       UGUAUGGCACAUUACCCGAC
Ga0247554_104968/233-375         UUCAUAGCAUAUUAACUCAC
Ga0265753_1030448/124-358        CGAGUAGCACAUUACCCGAU
Ga0032354_1086862/128-19         CCAUUGGCACAUUAACUGGG
Ga0213850_1119088/23-236         GUAGUAGCACAUUAACUCAC
Ga0222728_1000079/1837-1731      UCCCUAGUACAUUAACUGAG
Ga0247535_100068/719-828         GCAGUGGUAUAUUAACUCCC
Ga0265744_100861/398-567         AUCACGGCAUAUUAACUGGA
Ga0316038_100042/1676-1569       CAUAGGGCACAUUAACUCCU
Ga0184578_101068/480-310         CCUACGGCACAUUAACUGAC
Ga0265759_100473/818-988         CCUACGGCACAUUAACUGAC
Ga0265756_100592/1458-1288       CCUACGGCACAUUAACUGAC
Ga0265746_1003835/1049-1219      CCUACGGCACAUUAACUGAC
Ga0247536_100773/637-807         CCUACGGCACAUUAACUGAC
Ga0184573_112336/323-153         CCUACGGCACAUUAACUGAC
Ga0247530_109035/489-319         CCUACGGCACAUUAACUGAC
Ga0247513_128543/241-71          CCUACGGCACAUUAACUGAC
Ga0247541_101130/558-391         UCCACGGCACAUUAACUGAC
Ga0213850_1278242/600-506        GCCGCGGUACAUUAACCCAC
Ga0213850_1524759/286-380        GCCGCGGUACAUUAACCCAC
Ga0180107_1045142/740-541        UCGCUAGCAUAUUAACUGGA
Ga0307484_102002/698-552         CGAGUAGCACAUUACCCGGU
Ga0265759_115288/4-102           CCUAUGGUAUAUUAACCGGU
Ga0316029_136733/88-8            UGGUCAGUAUAUUAACUUGC
Ga0242646_1008525/229-119        A.GUCAGCAUAUUAACUAAC
Ga0115594_1180137/311-161        CCGCGAGCACAUUAACUGGC
Ga0179947_1156513/270-495        CUGAGAGCAUAUUAACUCAU
Ga0179948_1263234/366-292        CUGAGAGCAUAUUAACUCAU

                                 >>>>>>.........>>>>>
                                 222222.........22222
                                 ○●●●●RGYAYAUUA●CY●●Y
```

```
Ga0179947__1148619/378-303         CUGUGAGUAUAUUAACUCAU
Ga0257141__1138773/402-200         AG.GGUGCAUAUUACCCUGC
Ga0242654__10401151/84-171         UUGAAAGCACAUUAACUGAC
Ga0184603__114388/264-401          ACGUACGUAUAUUAACUCAC
Ga0316052__118887/313-177          ACGUACGUAUAUUAACUCAC
Ga0079039__1671802/197-34          ACAGAGGUACAUGGACUGGU
Ga0247542__100007/609-500          .CUCUUGCACAUUACUUGGC
Ga0247552__100007/4311-4420        CCUCUUGCACAUUACUUGGC
Ga0184586__103737/25-134           CCUCUUGCACAUUACUUGGC
Ga0184594__103311/207-316          CCUCUUGCACAUUACUUGGC
Ga0265756__101275/650-541          CCUCUUGCACAUUACUUGGC
Ga0247541__100010/3268-3377        CCUCUUGCACAUUACUUGGC
Ga0184599__154003/184-293          CCUCUUGCACAUUACUUGGC
Ga0184580__121439/573-464          CCUCUUGCACAUUACUUGGC
Ga0184582__108821/1050-1159        CCUCUUGCACAUUACUUGGC
Ga0184575__101094/586-477          CCUCUUGCACAUUACUUGGC
Ga0184598__129111/512-403          CCUCUUGCACAUUACUUGGC
Ga0184596__107732/313-205          CCUCUUGCACAUUACUUGGC
Ga0247554__100006/1592-1485        CCUCUUGCACAUUACUUGGC
Ga0247534__100047/1899-2008        CCUCUUGCACAUUACUUGGC
Ga0184597__128933/573-464          CCUCUUGCACAUUACUUGGC
Ga0213850__1302171/343-113         .CCUUGGUACAUUAACUCAC
Ga0213850__1513954/279-196         CGUUCGGUACAUUACCCGAC
Ga0153880__1231581/116-241         UCACGGGUAAAUUAACUCAC
Ga0247541__104364/333-188          GUAGUAGCACAUUAACUUAC
Ga0138277__1028338/433-516         .UAGUAGCACAUUAACUCAC
Ga0265752__101001/521-687          CUAGUAGCACAUUAACCUAC
Ga0307482__1449537/408-223         CUCGUAGCACAUUACCUGAC
Ga0257136__1044348/475-684         GCGUUAGCACAUUAACUGGU
Ga0257138__1055324/266-476         GCGUUAGCACAUUAACUGGU
Ga0257140__1130477/77-287          GCGUUAGCACAUUAACUGGU
Ga0127502__10711632/191-294        UACAGAGGAAAUUAACCUUC
Ga0184568__105547/129-239          ..CGCAGCACAUUAACUCGG
Ga0206353__11976089/292-380        AAAUCGGCACAUUAACUGAC
Ga0184577__108791/182-295          GCUGGGGUACCUUAACUGGU
Ga0242649__1050441/340-170         CUGAUGGUACAUUACCUCCC
Ga0179934__1197833/207-115         ..AGAGGUACAUUAACUUCC
Ga0180107__1174136/218-102         ...GAAGAAAAUUAACUGGC
Ga0224712__10002011/1106-1211      CUAGAGGCACAUUAACUGGC
Ga0224712__10010317/2429-2325      CUACAGGCACAUUAACUGGC
Ga0206355__1311803/144-334         AAUACGGUAAAUUAACUGGC
Ga0307482__1417159/374-296         AGGGUGGCAUAUUAACCACC
Ga0213850__1396191/936-678         .CCGAGGUACAUUAACUCAC
Ga0222728__1287830/12-211          GA.GCGGUACAUUAACUGGC
Ga0242652__1169169/12-211          GA.GCGGUACAUUAACUGGC
Ga0242654__11312699/30-229         GA.GCGGUACAUUAACUGGC
Ga0265759__104327/339-243          .GCCUGGUACAUUAACUCAC
Ga0265756__100717/910-1050         GCAGUAGUACAUUAACCUGG
Ga0265757__113513/277-362          CUGGUGGCACAUUAACUCAC
Ga0242654__10024002/836-915        .ACGUGGUACAUUAACUCAC
Ga0184573__101763/767-477          CAAGUAGCACAUUACCCGAU
Ga0242654__10701525/53-296         CCAUAGGCACAUUAACCGGA
Ga0247529__117158/253-331          CCCCCGGCACAUUACCCAUC
Ga0326631__117973/181-293          CUUGCGGCACAUUAACGAGC
Ga0316038__107116/33-174           G.CGGGGCAUAUUAACUAGC
Ga0316037__108094/19-160           G.CGGGGCAUAUUAACUAGC
Ga0247527__116942/161-88           AAACGAGCAUAUUAACUGGC
Ga0138278__1022898/219-127         UGCGUGGUAAAUUAACCCAC
Ga0127503__11028958/51-130         .GUACGGCACAUUAACUCAC
Ga0307482__1623775/241-347         CGCAUGGCAUAUUAACUCCU
Ga0307484__111921/444-550          CGCAUGGCAUAUUAACUCCU
Ga0307484__101582/1018-1124        CGCAUGGCAUAUUAACUCCU
Ga0265742__1021317/385-459         ..CACGGCACAUUAACCCAC
Ga0157569__1089063/252-138         GCGCGGGCACAUUACCCUAC
Ga0153880__1457361/236-121         GCGCGGGCACAUUACCCUAC
Ga0256352__1087084/23-118          GCGCGGGCACAUUACCCUUC
Ga0307482__1000325/24-131          CGAUAAGUAAAUUACCCAGC
Ga0157560__1147189/148-60          CG.UGUGAAAAUUAACUGAC
Ga0247519__105128/410-263          CACAGGGUAUAUUACCUCUU
Ga0184573__113193/903-756          CACAGGGUAUAUUACCUCUU
Ga0157593__1013623/299-215         AGAGUAGCACAUUAACUCAC
Ga0184593__126886/349-213          U.UGCAGCACAUUAACCAGC
Ga0184568__117306/561-697          U.UGCAGCACAUUAACCAGC
Ga0184597__120210/233-147          CCUGCGGCACAUUAACUCAC
Ga0184570__100371/69-155           CCUGCGGCACAUUAACUCAC
Ga0157562__1160399/232-315         CUACUGGUACAUUAACUUGG
Ga0265744__114151/132-24           AAGCGUGUACAUUAACUGAC
Ga0265743__119695/104-184          UCAGCAGCACAUUAACGACC
Ga0265743__101198/448-619          AUCAUGGCAUAUUAACUGGA
Ga0316046__121611/330-214          AUGCGGGCAAAUUAACUUGC
Ga0265757__120140/89-204           AUGCGGGCAAAUUAACUGGC
Ga0138279__1083071/419-326         ...GGGGUACAUUACCCGAC
Ga0310106__100998/1608-1690        UAACAGGUAAAUUAACUGCU
Ga0265756__102054/286-395          GAUGUAGUAAAUUAACUGCU
Ga0180108__1209503/2438-2343       ACUAUGGUACAUUAACUGGA
Ga0180107__1057591/210-107         ..GUGAGCAAAUUAACUGCC
Ga0307482__1074296/478-354         CAUCGGGCACAUUAACUGAC
Ga0180108__1022680/4248-4153       ACCAUAGCACAUUAACUGGU
Ga0247519__103291/1381-1460        .GAUGGGCACAUUAACCACA

                                   >>>>>>.........>>>>>
                                   222222.........22222
                                   ●●●●●RGYAYAUUA●CY●●Y
```

```
Ga0184590__126350/300-380          .GAUGGGCACAUUAACCACA
Ga0247551__100996/477-556          .GAUGGGCACAUUAACCACA
Ga0316035__144545/195-301          .CGUGGGUACAUUACCUCAC
Ga0316049__154968/227-319          ..GGUAGAAAAUUAACUGGC
Ga0184593__115334/333-240          AAGGUAGAAAAUUAACUGGC
Ga0307921__1005950/551-460         UCAUCGGUACAUUAACUCGC
Ga0242647__1014694/423-336         .AGAUGGUAAAUUAACUUGC
Ga0184580__102899/442-582          UUAACGGCACAUUAACUGGC
Ga0265743__108998/150-253          GUGAUAGUAAAUUAACUCUC
Ga0316050__103193/566-460          GUGAUAGCAUAUUAACUCUC
Ga0316030__100208/805-700          UCAUCAGAAUAUUAACUCUC
Ga0307482__1000262/4800-4699       CUGAUGGUAUAUUAACCGGC
Ga0307482__1018929/1182-1081       CUGAUAGUAUAUUAACCGGC
Ga0153880__1260556/441-589         UAUGUAGCACAUUAACCUGC
Ga0180044__1087599/197-316         CCCGCGGCACAUUAACCCAC
Ga0127502__11371108/362-252        CUGGUAGCACAUUAACUCUC
Ga0184588__106948/865-640          UACGCAGCACAUUAACCUAC
Ga0247533__100438/1040-888         AGUGUAGCACAUUAACUGAG
Ga0127502__10189886/163-55         ACGUGGGCACAUUAACUAGC
Ga0079074__1318576/412-571         CUUAUGGUACAUUAACUGGU
Ga0115594__1212468/8-118           UAUGUAGUACAUUAACUCAC
Ga0115594__1134035/652-542         UUUGUAGCACAUUAACUCAC
Ga0316030__105840/623-504          UGUGUGUUAUAUUAACUUGC
Ga0316033__110175/528-409          UGUGUGUUAUAUUAACUUGC
Ga0228708__1128185/228-344         C.UAAGGUAAAUUAACUUGC
Ga0126318__10199518/109-204        GCUGGGGCACAUUAACUCAC
Ga0265745__1000697/596-785         CACGAGGCACAUUAACUGGU
Ga0265747__105373/294-105          UACGAGGCACAUUAACUGGU
Ga0265745__1001084/890-701         UACGAGGCACAUUAACUGGU
Ga0265745__1000512/1043-854        UACGAGGCACAUUAACUGGU
Ga0310117__104105/1058-869         UGCAAGGCACAUUAACUGGU
Ga0184587__113157/56-168           GCCGUGGCACAUUACCCGGU
Ga0206356__10600373/215-21         CGUGAGGCACAUUAACUGCC
Ga0307920__126542/349-261          .UGGCGGCAUAUUAACUAGA
Ga0265742__1001154/131-376         CUUCCGGUACAUUAACUCAC
Ga0307482__1127544/505-398         UGGAGGGCACAUUAACUGGC
Ga0127503__11058765/362-444        ..ACGGUUAUAUUAACUGCC
Ga0210323__1050507/418-532         ..CGCGGCACAUUAACUGGC
Ga0242647__1002063/315-62          CAAGUAGCACAUUACCCACC
Ga0307484__100992/499-246          CAAGUAGCACAUUACCCACC
Ga0247534__117055/74-212           UUGCUAGCACAUUAACUGGA
Ga0129331__1494381/292-368         GACCGAGCACAUUAACUUGC
Ga0242646__1000880/695-856         AUAGAGGCACAUUAACUGGA
Ga0180107__1174136/376-571         UGCGUGGUACAUUACCUUAG
Ga0307921__1050271/251-412         AGGGCGGCACAUUAACUCUC
Ga0247524__126530/125-198          AAUGUGGGAAAUUAACUGAG
Ga0242646__1001007/790-609         ACAGAGGCAUAUUAACUGAG
Ga0213850__1280121/544-351         CACUCGGCACAUUAACCAGC
Ga0307919__1109364/247-158         CUUCUGGUACAUUAACUAAC
Ga0184600__147964/253-339          CAUUUAGUACAUUAACUCAC
Ga0316036__107710/408-322          CAUUUAGUACAUUAACUCAC
Ga0184598__138497/440-355          CUUUUGGUACAUUAACUCAU
Ga0184589__121067/502-587          CAUUUGGUACAUUAACUCAU
Ga0184601__135946/406-321          CAUUUGGUACAUUAACUCAU
Ga0115595__1093158/7-137           ..UUGUGCAAAUUAACUGGC
Ga0005851__1006483/274-172         AAAGCAGCACAUUAACUCAC
Ga0247522__108409/684-554          CGAGUAGCACAUUACCCGGU
Ga0310119__105137/743-673          .GAGUAGUAAAUUAACCAGC
Ga0247529__116306/255-362          UACGCAGCACAUUAACUACC
Ga0316046__116376/278-384          UACGCAGCACAUUAACUACC
Ga0247534__102735/124-18           UACGCAGCACAUUAACUACC
Ga0247540__102569/210-104          UACGCAGCACAUUAACUACC
Ga0316037__112281/325-219          UACGCAGCACAUUAACUACC
Ga0184576__110594/6-113            UACGCAGCACAUUAACUACC
Ga0316049__114344/181-288          UACGCAGCACAUUAACUACC
Ga0115594__1200134/154-31          UACAGAGCACAUUACCCGAC
Ga0307480__1000549/913-1064        UCGGUAGCAUAUUAACUGUC
Ga0242647__1000589/378-557         UGUUGGGCACAUUAACUGGU
Ga0222729__1000922/1729-1550       UGUUGGGCACAUUAACUGGU
Ga0182091__1104347/98-194          GCUGUAGCACAUUAACUUGC
Ga0316037__128286/253-347          CGCGUAGCAUAUUAACUGAC
Ga0180107__1040049/1100-1010       UCGAUGGUACAUUAACUUGC
Ga0242641__1014724/551-384         AGCCGAGCACAUUAACUGGA
Ga0242641__1002366/499-331         AGCCGAGCACAUUAACUGGA
Ga0213850__1508869/496-402         GGUGUAGCAUAUUAACUUGC
JGI12272J11983__1072695/40-150     CCAGUAGCACAUUAACUCCC
Ga0307482__1020622/600-404         .CGGUGGCACAUUAACUGGA
Ga0242657__1009512/1576-1466       CUGGUGGUACAUUAACUUCC
Ga0184577__116723/415-311          AACACGGCACAUUAACUGCC
Ga0184576__109717/432-536          AACACGGCACAUUAACUGCC
Ga0184572__113323/230-300          .ACACGGCACAUUACCCCUA
Ga0307354__155926/276-116          CUUGUGGCACAUUAACUGGC
Ga0265756__100433/485-286          ACAGUAGCACAUUAACUGGA
Ga0265751__100477/874-675          ACAGUAGCACAUUAACUGGA
Ga0180045__119797/166-305          AUAGAGGCACAUUAACUGGC
Ga0222728__1380294/134-238         GAUAGAGCACAUUACCCGUC
Ga0265758__104499/242-367          ACGUUAGCACAUUAACUGGC
Ga0079039__1551950/237-315         ..AGCAGCACAUUAACUUGC
Ga0157570__1034241/151-229         UGCACGGUACAUUACCCGUC

                                   >>>>>>.........>>>>>
                                   222222.........22222
                                   ∘●●●●RGYAYAUUA●CY●●Y
```

```
Ga0157571__1064089/212-134        UGCACGGUACAUUACCCGUC
Ga0179951__1211246/478-633        CAAUUAGUAAAUUAACUGCC
Ga0179950__1197742/286-441        CAAUUAGUAAAUUAACUGCC
Ga0184578__120646/150-246         UUCUGUGAAAAUUAACUCAC
Ga0247526__105246/689-508         UAAGUGGCACAUUAACUGGA
Ga0247538__100442/1363-1181       UAAGUGGCACAUUAACUGGA
Ga0247515__107373/548-731         UAAGUGGCACAUUAACUGGA
Ga0247538__100551/721-542         UAAGUGGCACAUUAACUGGA
Ga0184588__131258/156-253         AUAGUGGUACAUUACUUAUG
Ga0316047__100860/1040-888        AGUGUAGCACAUUAACUGAG
Ga0242657__1476020/160-244        GAAGUAGCACAUUAACUGGC
Ga0184602__130623/1321-1100       UAAGGGGUACAUUAACUGGA
Ga0153880__1410217/288-397        UGAGUAGUACAUUAACUUCC
Ga0316049__129960/371-262         UCUGUAGCACAUUAACUCAC
Ga0265747__125875/320-395         .GCGCAGCACAUUAACUCAC
Ga0242647__1002473/759-521        CCAACGGCACAUUAACUGGU
Ga0242641__1004288/32-112         .UGGGGGUACAUUAACCAGC
Ga0247553__101094/709-629         ..GUGGGCACAUUAACCAGC
Ga0127503__10814644/496-352       AGUUUAGCACAUUAACUGAC
Ga0179939__1199779/67-205         GCUGAAGCACAUUAACUGGU
Ga0180107__1021361/468-373        CUUGGGGCACAUUAACUGGU
Ga0180107__1218358/314-219        GUUGAGGUACAUUAACUGGA
Ga0265758__100138/1527-1633       UGUAUAGCACAUUAACUGGG
Ga0265752__100416/442-337         UAUACAGCAUAUUAACUGGG
Ga0247534__117029/65-195          CGGGUGGAACAUUACCCGGC
Ga0187793__1322799/72-151         .CCCUGGUACAUUAACCUGC
Ga0307921__1005950/791-877        CACCUGGCACAUUAACUUGC
Ga0180047__1128081/151-64         GUGCUGGCACAUUACCCGGC
Ga0157593__1125711/213-128        GUGCUGGCACAUUACCCGGC
Ga0157569__1032488/120-205        .GGCUGGUACAUUACCCGGC
Ga0157565__1077069/156-67         ACAACGGUACAUUACCCGGC
Ga0157593__1023702/238-314        ..AGCGGCACAUUACCCAAC
Ga0255267__1268184/91-184         ...GCGGCACAUUACCCGAC
Ga0307919__1081825/191-74         UUAGUGGCACAUUAACCCAC
Ga0307921__1095896/178-61         UUAGUGGCACAUUAACCCAC
Ga0316040__172648/178-66          UUAGUGGCACAUUAACCCAC
Ga0184577__110850/246-358         UUAGUGGCACAUUAACCCAC
Ga0307920__113994/130-9           UUAGUGGCACAUUAACCCAC
Ga0307920__113994/654-539         UUAGUGGCACAUUAACCCAC
Ga0307921__1037919/342-457        UUAGUGGCACAUUAACCCAC
Ga0242646__1001980/23-280         U.CGAGGCACAUUAACUGGC
Ga0179935__1139075/165-78         UAUGUAGCACAUUACCUGGC
Ga0179944__1246019/249-162        UCUGUAGCACAUUACCUUGC
Ga0180044__1069332/269-187        AAUGUAGCACAUUACCUAGC
Ga0157564__1065218/271-353        AAUGUAGCACAUUACCUAGC
Ga0180044__1047678/444-368        AAAGUAGCACAUUACCCAGU
Ga0180047__1061805/548-622        .GAGGAGCACAUUACCUGGC
Ga0157569__1073490/184-258        .GAGGAGCACAUUACCUGGC
Ga0213850__1394829/253-105        CGAUGAGCACAUUAACUGGC
Ga0255293__1138691/440-213        ..GACGGCACAUUAACUGGC
Ga0222728__1000079/1192-1299      UUGAUAGCACAUUAACUCCC
Ga0081246__1201717/153-259        ACAUCGGAAAAUUAACUCCC
Ga0222728__1216887/51-157         UCAGUGGUACAUUAACUCCU
Ga0265754__1141843/290-185        CUGAUAGUAAAUUAACUCCC
JGI12272J11983__1345183/364-258   CUGAUGGAAAAUUAACUCCC
Ga0242647__1005934/451-617        CCGAUGGCACAUUAACCGGA
Ga0115594__1187657/870-960        AUAAGGGCACAUUAACUCCC
Ga0115594__1174394/642-729        GUAAGGGCACAUUAACUCCC
Ga0184569__112764/943-836         AGCGCAGUACAUUAACUCCC
Ga0265750__1013321/389-496        CCAAUGGCACAUUAACUCCC
Ga0265757__139522/177-70          CCAAUGGCACAUUAACUCCC
Ga0316029__116217/368-261         AGCGCAGUACAUUAACUCCC
Ga0316047__100016/2681-2574       AGCGCAGUACAUUAACUCCC
Ga0256352__1101982/155-277        CAAUCGGUACAUUAACUGAC
Ga0256350__1100736/283-405        CAAUCGGUACAUUAACUGAC
Ga0079039__1443516/199-38         ACAGAGGCACAUUAACUGGU
Ga0115595__1072788/334-441        UCAGCAGCACAUUAGCCGGC
Ga0115594__1022316/346-239        UCAGCAGCACAUUAGCCGGC
Ga0129331__1058147/189-99         ACGCGGGCACCUUAACUCAC
Ga0265751__100127/176-103         .GUGGUGCACAUUAACUGGC
Ga0307921__1043977/91-164         ..CUCGGCACAUUAACUAUC
Ga0307919__1048575/242-90         AGGGCGGCACAUUAACUCUC
Ga0242646__1001086/737-571        CAUGUAGCAUAUUAACUGGA
Ga0265752__111922/418-343         .GUGUAGCACAUUAACUGCC
Ga0157562__1134247/69-160         GCAGUAGCACAUUAACUGGC
Ga0157559__1047848/128-41         ACAGUGGUACAUUAACUGGC
Ga0179944__1313268/129-232        GUUGUAGCACAUUAACUGGC
Ga0213850__1259428/445-342        GUUGUAGCACAUUAACUGGC
Ga0153880__1167367/224-121        GUUGUAGCACAUUAACUGGC
Ga0153880__1058095/344-241        GUUGUAGCACAUUAUCUGGC
Ga0179943__1178349/195-298        GUUGUAGCACAUUAACUGGC
Ga0307921__1075885/188-36         AGGGCGGCACAUUAACUCUC
Ga0307921__1062541/180-338        AGGGCGGCACAUUAACUCUC
Ga0242641__1014474/336-257        AGAGUAGCACAUUACCCGAC
Ga0307919__1064641/225-73         AGGGCGGCACAUUAACUCUC
Ga0265759__108120/277-351         ..GCGGGCACAUUAACUGGC
Ga0265753__1005277/856-1057       GGUAGAGCACAUUAACUGGA
Ga0247523__120120/325-403         GGUUGAGCACAUUAACUGGU
Ga0228707__1100139/344-194        UGAUUGGUACAUUACCCACC

                                  >>>>>>.........>>>>>
                                  222222.........22222
                                  ∘••••RGYAYAUUA•CY••Y
```

```
Ga0316049_154526/155-74              ..UGCAGCACAUUAACCCAC
Ga0316035_177575/224-142             ..UGCAGCACAUUAACCCAC
Ga0307920_126403/177-486             G.CGCGGCACAUUAACUCGC
Ga0138280_1140856/341-479            GCGGCGGCACAUUAACUGGC
Ga0247539_101233/414-526             AUUGCAGUAAAUUAACUCAC
Ga0184569_108885/200-88              AUUGCAGUAAAUUAACUCAC
Ga0247521_112559/317-205             AUUGCAGUAAAUUAACUCAC
Ga0184599_154888/232-155             AUAGGAGCAAAUUAACUGGU
Ga0180108_1143860/199-116            .GGAUGGAAAAUUAACUGCC
Ga0247524_121352/374-303             .CAUGAGGAAAUUAACCACC
Ga0206356_11002907/329-148           UUCAUGGCACAUUAACUGGC
Ga0265743_103007/946-842             AUCAUGGUAUAUUAACUUCC
Ga0316033_101029/151-49              C.CUGAGCACAUUAACUGUC
Ga0179937_1126086/216-325            GUAGUAGCACAUUAACUUCC
Ga0307482_1050048/176-95             GGAUGAGCACAUUAACUGGC
JGI12272J11983_1319142/230-159       GUAUGAGCACAUUAACUGCC
Ga0180107_1285918/13-113             ..GCGGGCACAUUAACUACC
Ga0265756_108694/382-277             CCAGCAGCACAUUAACUUGC
Ga0265746_1140777/214-319            CCAGCAGCACAUUAACUUGC
Ga0184574_101359/409-493             UUAGUAGCACAUUACCUCCU
Ga0184568_108556/391-307             UUAGUAGCACAUUACCUCCU
Ga0316030_107573/391-475             UUAGUAGCACAUUACCUCCU
Ga0180107_1031754/139-57             AGUGUAGCAUAUUAACUGGC
Ga0307919_1029784/253-144            GUAAAGGCACAUUAACUCAC
Ga0307921_1042549/343-452            GUAAAGGCAUAUUAACUCAC
Ga0307482_1021658/484-563            AGCAGAGCAUAUUAACUGGC
Ga0179943_1102267/215-90             GACUCGGUAAAUUAACUGGC
Ga0079074_1279626/114-274            CUUGUGGCACAUUAACUGGC
Ga0307921_1019702/90-184             ...GUAGCACAUUAACUUGC
Ga0307920_133961/233-140             .UCGUAGCACAUUAACUUGC
Ga0316035_179807/270-184             GUUGUAGUACAUUAACUGGC
Ga0256338_1343513/152-228            CUGGUGGUACAUUAACUGGG
Ga0255268_1432700/127-51             CUGGUGGUACAUUAACUGGG
Ga0153880_1067318/492-566            UAUGUAGUACAUUAACUCGG
Ga0247533_103075/417-305             CAUGUGGUACAUUAACUGGG
Ga0247541_102964/56-168              CAUGUGGUACAUUAACUGGG
Ga0184589_110648/1041-881            CUACGGGCACAUUAACUGGG
Ga0184580_124121/239-79              CUACGGGCACAUUAACUGGG
Ga0184598_101324/530-690             CUACGGGCACAUUAACUGGG
Ga0184588_120624/266-426             CUACAGGCACAUUAACUGGG
Ga0184579_106194/996-1156            CUACAGGCACAUUAACUGGG
Ga0265758_100254/356-516             CUACGGGCACAUUAACUGGG
Ga0184597_106861/196-36              CUACGGGCACAUUAACUGGG
Ga0184600_132066/568-408             CUACGGGCACAUUAACUGGG
Ga0316031_108320/216-376             CUACGGGCACAUUAACUGGG
Ga0184596_109261/979-1139            CUACGGGCACAUUAACUGGG
Ga0316035_107544/354-514             CUACGGGCACAUUAACUGGG
Ga0247527_115808/280-440             CUACGGGCACAUUAACUGGG
Ga0247519_107918/217-58              AUGGAGGCACAUUAACUGGA
Ga0184589_112135/40-200              ACAGAGGCACAUUAACUGGC
Ga0247551_100195/490-329             ACAGAGGCACAUUAACUGGA
Ga0247526_117235/152-313             ACAGAGGCACAUUAACUGGA
Ga0326631_101563/544-705             AUGGAGGCACAUUAACUGGA
Ga0184596_111007/525-686             AUGGAGGCACAUUAACUGGA
Ga0184573_110190/115-276             AUGGAGGCACAUUAACUGGA
Ga0316033_144939/167-328             AUGGAGGCACAUUAACUGGA
Ga0247552_101742/612-452             AUGGAGGCACAUUAACUGGA
Ga0247545_112960/183-343             ACAGAGGCACAUUAACUGGA
Ga0247541_103784/123-283             AUGGAGGCACAUUAACUGGA
Ga0247524_111427/50-210              AUGGAGGCACAUUAACUGGA
Ga0247545_104341/406-246             AUGGAGGCACAUUAACUGGA
Ga0184602_114416/11-171              AUGGAGGCACAUUAACUGGA
Ga0316038_111670/11-171              AUGGAGGCACAUUAACUGGA
Ga0184586_133850/368-528             AUGGAGGCACAUUAACUGGA
Ga0184600_123882/188-28              AUGGAGGCACAUUAACUGGA
Ga0184574_121290/269-109             AUGGAGGCACAUUAACUGGA
Ga0316032_107575/215-55              AUGGAGGCACAUUAACUGGA
Ga0247519_110701/607-447             AUGGAGGCACAUUAACUGGA
Ga0247526_110221/603-443             AUGGAGGCACAUUAACUGGA
Ga0247529_134913/224-64              AUGGAGGCACAUUAACUGGA
Ga0247529_103231/270-430             AUGGAGGCACAUUAACUGGA
Ga0184580_114961/306-146             AUGGAGGCACAUUAACUGGA
Ga0184603_126764/80-240              AUGGAGGCACAUUAACUGGA
Ga0247550_114736/167-7               AUGGAGGCACAUUAACUGGA
Ga0184603_111918/241-82              AUGGAGGCACAUUAACUGGA
Ga0247538_100424/120-281             AUGGAGGCACAUUAACUGGA
Ga0247547_102464/189-29              AUGGAGGCACAUUAACUGGA
Ga0247551_100828/546-387             AUGAAGGCACAUUAACUGGA
Ga0247538_100480/788-947             AUGAAGGCACAUUAACUGGA
Ga0247550_100850/456-297             AUGAAGGCACAUUAACUGGA
Ga0247536_101914/360-201             AUGAAGGCACAUUAACUGGA
Ga0247553_110698/526-367             AUGAAGGCACAUUAACUGGA
Ga0153880_1114976/150-383            GAUGUAGCAUAUUAACUGAC
Ga0180052_189627/240-346             GCAUCGGUACAUUAACUCGC
Ga0184598_136114/144-251             CCUGUAGCACAUUAACGAGG
Ga0222728_1011988/841-947            UGCCUGGCACAUUAACCAAU
Ga0265751_160438/108-215             GGCGCGGCACAUUAACUUGC
Ga0247514_107497/1071-1140           GAUGUGGCAUAUUAACUGGC
Ga0180107_1129325/244-60             GCUGAGGCACAUUAACUGAC

                                     >>>>>>.........>>>>>
                                     222222.........22222
                                     ●●●●●RGYAYAUUA●CY●●Y
```

```
Ga0242646__1002014/11-270              ACGUGGGCACAUUAACUGGC
Ga0222728__1008526/24-283              ACCUGGGCACAUUAACUGGC
Ga0222728__1008391/1281-1023           ACGUGGGCACAUUAACUGGC
Ga0265759__100017/4211-4288            .GAUAGGAAAAUUAACUGGC
Ga0247512__104440/911-1082             UCGGUAGCACAUUAACUGGC
Ga0265756__131580/159-58               .ACGUAGCACAUUAACUCGC
Ga0310119__169621/253-177              .GUCUGGUACAUUAACUGGC
Ga0247516__124175/202-272              UAUGCAGCAUAUUAACUCGC
Ga0247534__100772/309-234              .AGAUGGCAAAUUAACUGGC
Ga0222728__1054736/119-193             ..UCCAGAAAAUUAACUGGC
Ga0180106__1297567/444-515             ....GGGCACAUUAACUGGC
Ga0242647__1000066/2638-2719           ..GUGGGCACAUUAACUGGC
Ga0307484__104189/90-171               .AGGUGGAAAAUUAACUGCC
Ga0316048__109289/45-149               AUGAUGGUACAUUAACUGGU
Ga0265750__1038125/531-427             AUGAUGGUAUAUUAACUGGU
Ga0247537__109516/130-26               CUGAUGGCACAUUAACUGGC
Ga0310115__103434/744-848              CUGAUGGCACAUUAACUGGC
Ga0307482__1009758/1126-1021           ACUGCGGCACAUUAACUGGA
JGI12272J11983__1356304/1548-1654      UCCAAGGCACAUUAACUGGU
JGI12272J11983__1072695/645-539        CCACUGGCAUAUUAACUGGA
Ga0242657__1009512/820-926             GCAGAGGCACAUUAACUGGA
Ga0316037__172473/89-196               UCUAUAGCACAUUAACUGGU
Ga0184592__121084/378-271              CCCAUAGCACAUUAACUGGU
Ga0184569__113518/504-609              CCCAUAGCACAUUAACUGGU
Ga0184602__114837/392-286              CUU.CAGCACAUUAACUGGU
Ga0316030__102131/386-280              CUU.CAGCACAUUAACUGGU
Ga0310118__104334/336-443              ACCGUAGCAUAUUAACCUGG
Ga0180108__1146973/462-263             CUGCUGGUACAUUACCUAGC
Ga0184580__111164/678-749              .CAUGUGUACAUUAACUGGC
Ga0242647__1006852/526-598             ..AGGUGCACAUUAACUGGC
Ga0179939__1059820/107-216             GUAGUAGCAUAUUAACUGGC
Ga0115594__1156252/268-370             CUGAGGGCACAUUAACUGGC
Ga0115594__1006122/120-29              UGAAGGGCACAUUAACUGGC
Ga0115595__1180708/226-317             UGAAGGGCACAUUAACUGGC
Ga0115594__1211214/299-389             CAAAGGGCACAUUAACUGGC
Ga0115594__1007070/120-30              UCAAGGGCACAUUAACUGGC
Ga0115595__1181384/176-86              UCAAGGGCACAUUAACUGGC
Ga0115594__1166236/119-19              UGAAGGGCACAUUAACUGGC
Ga0115594__1157594/155-254             UGAAGGGCACAUUAACUGGC
Ga0256352__1182139/180-81              UGAAGGGCACAUUAACUGGC
Ga0115594__1182529/183-84              UGAAGGGCACAUUAACUGGC
Ga0265746__1064626/389-458             .GAGCGGAAUAUUAACUGGC
Ga0115594__1016490/326-410             CUGAGGGCACAUUAACUGGC
Ga0115595__1007417/328-412             CUGAGGGCACAUUAACUGGC
Ga0228708__1103570/188-285             A.CGUGGCACAUUAACUGGC
Ga0265751__121301/189-266              .CCAUGGUACAUUAACUGGC
Ga0179944__1005311/261-382             GCGCCGGUACAUUAACUCGC
Ga0179944__1191552/284-411             ACUACGGUACAUUAACUGGC
Ga0179943__1150468/216-89              ACUGCGGUAAAUUAACUGGC
Ga0179944__1204933/216-89              ACUGCGGUAAAUUAACUGGC
Ga0307482__1020425/440-614             AUCAUGGCACAUUAACUGGG
Ga0242646__1001395/255-509             UCUCAGGCAUAUUAACUGGC
Ga0184578__112031/156-44               AUACUAGGAUAUUAACUCAC
Ga0184577__120131/220-108              AUACUAGGAUAUUAACUCAC
Ga0247517__121471/387-499              AUACUAGAAUAUUAACUCAC
Ga0247539__107237/153-229              ..AUCGGCAUAUUAACUGGU
Ga0265758__109576/83-157               ...GCGGAAAAUUAACUGGG
Ga0184577__107529/254-327              .AGUGUGCACAUUAACUGCC
Ga0213850__1246156/171-47              AUCGUAGCACAUUAACUGGC
Ga0326631__172366/284-207              .CGGCAGCAUAUUAACUGGA
Ga0310114__149639/68-153               CUUGGGGUACAUUAACUGGA
Ga0184599__128731/188-273              CUUGGGGUACAUUAACCGGA
Ga0310119__131358/533-447              UUUGAGGUACAUUAACUGGA
Ga0247526__116548/279-354              .AGAUGGCAAAUUAACUGUC
Ga0247519__113845/334-409              CAGAUGGCAAAUUAACUGUC
Ga0247529__111070/303-228              CAGAUGGCAAAUUAACUGUC
Ga0184580__116530/249-177              .AGCGAGUAUAUUAACUGUC
Ga0247524__105844/561-637              .GAUGAGUAAAUUAACUACC
Ga0184593__119849/313-238              ..GUGAGAACAUUAACCAUC
Ga0184599__140898/774-849              ..GUGAGAAUAUUAACCAUC
Ga0316049__111814/256-181              ..GUGAGAACAUUAACCAUC
Ga0316034__100493/254-174              .AGUGGGAAAAUUAACUGGC
Ga0184600__118565/177-97               .AGUGGGAAGAUUAACUGGC
Ga0184602__122864/595-515              .AGUGGGAAGAUUAACUGGC
Ga0184596__126546/83-162               .AGUGGGAAGAUUAACUGGC
Ga0184597__129399/366-446              .AGUGGGCAAAUUAACUGGC
Ga0184576__117703/380-460              .AGUGGGAAAAUUAACUGGC
Ga0184590__111002/143-223              .AGUGAGUAAAUUAACUGGC
Ga0247551__103383/322-242              .AGUGGGAAAAUUAACUGGC
Ga0184597__111165/262-182              .AGUGGGAAAAUUAACUGGC
Ga0184570__116436/278-198              .AGUGGGAAAAUUAACUGGC
Ga0316037__169124/279-199              .AGUGGGAAGAUUAACUGGC
Ga0247543__103170/244-164              .AGUGGGAAAAUUAACUGGC
Ga0184589__120451/249-329              .AGUGGGAAUAUUAACUGGC
Ga0247519__108375/286-206              .AGUGGGAAGAUUAACUGGC
Ga0184587__105985/71-151               .AGUGGGAAGAUUAACUGGC
Ga0184593__101086/682-762              .AGUGGGAAGAUUAACUGGC
Ga0184602__121948/324-245              .GAUGGGCACAUUAACUGUC
Ga0184597__110829/1090-1011            .GAUGGGCACAUUAACUGUC

                                       >>>>>>.........>>>>>
                                       222222.........22222
                                       ●●●●●RGYAYAUUA●CY●●Y
```

```
Ga0184578_129447/314-235          .GAUGGGCACAUUAACUGUC
Ga0184588_113246/331-252          .GAUGGGUACAUUAACUGUC
Ga0316051_1003245/138-56          .GAUGGGUAUAUUAACUGUC
Ga0316046_122965/221-142          .GAUGGGUACAUUAACUGUC
Ga0184600_105920/81-160           .GAUGGGUACAUUAACUGUC
Ga0184603_125382/9-88             .GAUGGGUAUAUUAACUGUC
Ga0247550_100138/208-129          .GAUGGGUAUAUUAACUGUC
Ga0316049_122425/209-288          .GAUGGGUACAUUAACUGUC
Ga0032354_1062690/2659-2586       ...GGGGCACAUUAACUGGU
Ga0179955_1048341/123-51          ..CGUGGUACAUUAACUGGC
Ga0138277_1023917/182-18          GCGGUGGUACAUUAACUGGC
Ga0138282_1020546/737-573         GCAGUAGUACAUUAACUGGC
Ga0242641_1105635/265-351         CGGGUGGCACAUUAACUCAC
Ga0242654_10795375/263-349        CGGGUGGCACAUUAACUCAC
Ga0247554_113506/254-161          UUCAUGGAAAAUUAACCGGA
Ga0265745_1000618/793-552         CCGAUGGCACAUUAACUGGA
Ga0265742_1001052/322-80          CCGAUGGCACAUUAACUGGA
Ga0310113_106868/972-730          CCGAUGGCACAUUAACUGGA
Ga0265750_1009219/272-30          CCGAUGGCACAUUAACUGGA
Ga0265740_1001378/1084-842        CCGAUGGCACAUUAACUGGA
Ga0265752_100504/930-688          CCGAUGGCACAUUAACUGGA
Ga0265747_100653/495-253          CCGAUGGCACAUUAACUGGA
Ga0265757_104669/135-376          CCGAUGGCACAUUAACUGGA
Ga0265742_1088739/51-292          CCGAUGGCACUUUAACUGGA
Ga0265747_100509/1277-1036        CCGAUGGCACAUUAACUGGA
Ga0265752_100314/1466-1225        CCGAUGGCACAUUAACUGGA
Ga0213850_1450246/323-606         GCGAUGGUACAUUAACUGGU
Ga0157593_1101573/285-478         UACAUGGUACAUUAACUGGC
Ga0222728_1000125/1315-1238       .AACAGGUACAUUAACUGGC
Ga0242654_10000586/1273-1196      .AACAGGCACAUUAACUGGC
Ga0265756_100130/1310-1239        ...UUGGCACAUUAACUGGC
Ga0307482_1008742/1212-1288       .GAUAGGCACAUUAACUGGC
Ga0242654_10040657/1018-942       .GAUGGGUACAUUAACUGGC
Ga0307484_100842/1194-1271        .GAUAGGCACAUUAACUGGC
Ga0307482_1009223/670-508         CAGAGGGCAUAUUAACUGAC
Ga0307484_108094/186-350          AGGAGGGCAUAUUAACUGAC
Ga0247514_114593/280-211          .GGAUGGAAAAUUAACUGGC
Ga0184583_106441/296-227          .GGAUGGGACAUUAACUGGC
Ga0242654_10672177/244-165        GUUAUGGCACAUUAACUGGC
Ga0126318_10229384/609-688        GUGAAGGUACAUUAACUGGC
Ga0247551_122551/29-115           ..GAAGGCAAAUUAACUGGC
Ga0316048_107802/239-324          ..GAAGGCAAAUUAACUGGC
Ga0242641_1002064/1203-1116       .GGAUGGCACAUUAACUGGC
Ga0307482_1026216/884-813         ..GAUGGAAAAUUAACUGGC
Ga0307482_1636410/270-341         ..GAUGGAAAAUUAACUGGC
Ga0307482_1016877/168-97          ..GAUGGAAAAUUAACUGGC
Ga0180107_1106188/424-495         ..GAUGGAAAAUUAACUGGC
Ga0307482_1000354/2063-1987       .UGAAGGAAAAUUAACUGGC
Ga0184587_107783/167-95           .UGAUGGCACAUUAACUGGC
Ga0184585_128240/467-390          .UCUCGGAAAAUUAACUGGC
Ga0184596_102741/625-701          .GAUCGGUACAUUAACUGGU
Ga0247539_100086/1763-1850        .ACCAGGUACAUUAACUGGA
Ga0184584_115572/267-354          .ACCAAGUACAUUAACUGGA
Ga0184579_105257/1735-1648        .ACCAAGUACAUUAACUGGA
Ga0307482_1023270/273-197         .GAUCGGUACAUUAACUGGC
Ga0307482_1219330/19-95           ..ACAGGCACAUUAACUGGC
Ga0242657_1207391/462-383         GUAAUGGCAUAUUAACUGGC
Ga0247520_104849/891-821          .GAUUGGCACAUUAACUGGC
Ga0247531_104861/1082-1012        .GAUUGGCACAUUAACUGGC
Ga0247518_137824/301-231          .GAUUGGCACAUUAACUGGC
Ga0307482_1202605/470-395         .CACAGGUACAUUAACUGGA
Ga0307921_1021783/160-261         ..CGCAGCACAUUAACUGGC
Ga0242657_1054283/541-620         AUAUUGGAAAAUUAACUGGC
Ga0242647_1005188/439-360         AUAUUGGAAAAUUAACUGGC
Ga0153880_1391790/62-140          CAAGUAGCACAUUAAACCCC
Ga0153880_1379927/66-144          CAGGCAGCACAUUAAACCCC
Ga0307482_1023665/853-778         .CGGCAGCACAUUAACUGGU
Ga0242646_1047905/92-182          GGGGUAGCACAUUAACUCAC
Ga0307921_1024041/406-334         GCGGCAGCACAUUAACUCGU
Ga0307919_1039507/513-440         GCGGCAGCACAUUAACUCGU
Ga0307919_1084008/308-378         GGGGCAGCACAUUAACUCGC
Ga0247525_114510/199-318          CCGGUGGCACAUUAACUGGU
Ga0184603_107262/477-596          CCGGUGGCACAUUAACUGGU
Ga0184585_151549/495-376          CCGGUGGCAUAUUAACUGGU
Ga0310119_113172/381-306          .GGGUGGCACAUUAACUGGC
Ga0310114_133004/56-132           .GGGUGGCACAUUAACUGGC
Ga0184603_148109/996-920          .GGGUGGCACAUUAACUGGC
Ga0184599_110049/275-349          ..GGUGGCACAUUAACUGGC
Ga0184587_132427/565-485          GGGGUGGCACAUUAACUGGC
Ga0265756_135986/152-290          GUGGUGGCACAUUAACUGGC
Ga0247519_104852/800-730          ..GUUGGCAUAUUAACUGGC
Ga0213850_1274705/160-417         GUUGUGGUACAUUAACCCAC
Ga0265747_100454/1395-1324        .GGAUAGAAAAUUAACUGGU
Ga0247519_118271/334-405          AGUCUAGUAAAUUAACUGGU
Ga0242654_10135580/213-336        ..GGUGGUAAAUUAACUAGC
Ga0184576_110410/277-348          ..ACUGGAAAAUUAACUGCC
Ga0247535_105396/277-348          ..ACUGGAAAAUUAACUGCC
Ga0265758_104558/436-365          .UGGUGGUAAAUUAACUGGC
Ga0247526_117664/434-499          ..AGUGGGAAAUUAACUCGC

                                  >>>>>>.........>>>>>
                                  222222.........22222
                                  ●●●●●RGYAYAUUA●CY●●Y
```

```
Ga0316037__123461/289-368          AUCAUGGAAAAUUAACUGGU
Ga0247543__100863/642-721          GUAUUGGGAAAUUAACUGGC
Ga0247521__124040/388-309          GUAUUGGGACAUUAACUGGC
Ga0184589__120335/11-90            GUAUUGGGAAAUUAACUGGC
Ga0247518__108423/758-837          GUAUUGGGAAAUUAACUGGC
Ga0247531__104608/1256-1177        GUAUUGGGACAUUAACUGGC
Ga0265750__1365815/134-55          GUAUUGGGAAAUUAACUGGC
Ga0184597__121517/262-183          GUAUUGGGAAAUUAACUGGC
Ga0265747__102820/448-376          AUCAUGGAAAAUUAACUGGU
Ga0247554__105388/388-316          GUCAUGGAAAAUUAACUGGU
Ga0307482__1015330/1021-1093       ..GGUGGCACAUUAACUGGC
Ga0184600__102725/60-135           .UGUGAGCACAUUAACUGGU
Ga0184602__122974/190-265          .UGUGAGCACAUUAACUGGU
Ga0316038__112263/442-356          UAUUGGGCACAUUAACUGGC
Ga0307484__161007/136-39           .GGCGAGAAAAUUAACUGCC
Ga0307482__1033688/516-422         .GGCGAGAAAAUUAACUGCC
Ga0316031__132099/178-294          .UGCGGGUACAUUAACUGGG
Ga0213850__1220553/385-463         ..GGAGGUAAAUUAACUGCC
Ga0180107__1307618/257-174         AAGGCAGCAUAUUAACUCAC
Ga0265747__119090/8-84             ..GGCAGCACAUUAACUUGC
Ga0242646__1115622/191-109         .UGCGAGCAAAUUAACUGCC
Ga0184570__114412/209-338          UUCUAAGUAAAUUAACUUGC
Ga0265757__143567/145-70           UCACGAGAAGAUUAACUUGC
Ga0184568__109812/391-283          .UCGUAGCACAUUAACUCAC
Ga0184568__104528/922-1000         AUUCCAGUACAUUAACUCAU
                                   >>>>>>.........>>>>>
                                   222222.........22222
                                   ●●●●●RGYAYAUUA●CY●●Y
```

4 hairpin-stringent

For a description of this alignment, see Supplementary Table 2

4.1 Multiple-sequence alignment

Each ribozyme in this alignment is identified by its genomic location in the form SEQID/START-END. SEQID (the sequence accession) is derived from sources such as IMG/M or GenBank. START is the coordinate of the $5'$ nucleotide of the hairpin ribozyme, and END corresponds to the $3'$ nucleotide. If START>END, then the ribozyme is on the reverse complement strand.

Nucleotides proposed to basepair as part of the consensus structure are shaded in color when they comprise Watson-Crick or G-U pairs. Otherwise they are shaded gray. Conserved stems are also indicated at the bottom of the alignment by angle brackets, where matching $<$ and $>$ denote base-paired columns. Below these angle brackets, the symbol "2" denotes base pairs exhibiting covariation according to the statistically well-founded R-scape method. "1" denotes base pairs exhibiting covariation according to R2R's simplistic method. "0" denotes base pairs that are not observed to mutate and "?" denotes base pairs that have a significant frequency of non-canonical nucleotides for Watson-Crick or G-U pairs ($> 5\%$). Below these base pair annotation is the consensus sequence: "R" = "A" or "G", "Y" = "C" or "U", red nucleotides: nucleotide identity conserved more than 97% of the time, black nucleotides: 90%, gray nucleotides: 75%, red circle (●): nucleotide is present 97% of the time, black circle (●): 90%, gray circle (●): 75%, white circle (○): 50%. All percentages of sequences just described (e.g. 97% conserved) assume that sequences have been weighted by the GSC algorithm implemented by the Infernal software package.

The alignment begins on the next page.

alignment positions 1 · · · 145

```
Ga0179934_1155244/114-26        GGAUGGUCCG...........................................................................AAGGCAGAAGC.................................................
Ga0247515_120448/91-178         CAGCUGUCCU...........................................................................UAACU.......................................................
Ga0247518_120275/202-289        CAGCUGUCCU...........................................................................UAACU.......................................................
Ga0247522_108409/684-554        GUCCUGUCCUCUAGG...AUGAA........................GCACUAGGCGAGGUUG.CGGA.................AAGAC...........................UCCGCUGGCUUCAG....GGUGUGUG..
Ga0179955_1195671/481-563       AUGCGGUAUGCAUC.......................................................................AUAAU.......................................................
Ga0247529_117158/253-331        AGUCUGUCCCAG.........................................................................GCAAUCAAA...................................................
Ga0179948_1254202/198-296       AGUCGGUCCACAC........................................................................ACGUACUAAUA.................................................
Ga0247528_108647/438-562        AUACUGUCUGAUU.....UC...........................GUAUGGU...............................UUGU..........................................AUUCCAUACAU...
Ga0256798_1305521/221-305       CCUCUGUCCGGUUG.........................GAAA....G.....................................CCAA..................................................CAAGUA
JGI12272J11983_1345183/364-258  UUUCUGUCCCACAGUCC.G....UGC..GUAAA...GCAAU......GCC...................................AAGC................................................GGCUA...
JGI12272J11983_1072695/40-150   UCUUUGUCCUCGC..................................AUCCGU......GUGCGU....................AUA................................GCGCAAUCCCAAG..CGGGUAGGA.
Ga0179934_1197833/207-115       AGUCGGUCCACAG.....AAUCU........................UUGGCCAUC.............................AUCA........................................GAUGUUGUCAA.....
Ga0255059_10691632/166-76       UAUCAGUGUGUGG.....A............................AGAG..................................AUGAG..............................................CUCUCAA..
Ga0255062_11230321/200-110      UAUCAGUGUGUGG.....AA...........................GAGA..................................UGAGC..............................................UCUCGA...
Ga0247527_116942/161-88         AAGCGGGGCGGCA........................................................................UGCA........................................................
Ga0247521_112559/317-205        UCGCUGUGGUUUAUG...GUAAA........................GCC...................................UUAAUU..............................................GGCA....
Ga0247529_116306/255-362        GCGCUGUCCUGGCA....CUUU.........................UGACCUUCGAU...........................ACGG......................................AUCGAA..GGUUG.....
Ga0179944_1246019/249-162       UCAUUGUCCUAAG..................................ACU...................................UGGA................................................AGU.....
Ga0247517_121471/387-499        CAGCUGUGGUUGGAUG..GUAAA........................GCC...................................UUUAGU..............................................GGCA....
Ga0247524_126530/125-198        UUACGGUCCAUCC........................................................................GAUGA.......................................................
Ga0247519_107918/217-58         AAUCUGUCCCUACUUCGG.....GGGU.GCCAA..ACCCCAAGCACAAGCUGAA........CUGCGAU................AUGAG..........................AUCGCAGAACACUUCCU..CAGCUA....
Ga0247529_103231/270-430        AAUCUGUCCCUACUUCGG.....GGGU.GCCAA..ACCCCAAGCACAAGCUGAA........CUGCGAU................AUGAG..........................AUCGCAGAACACUUCCU..CAGCUA....
Ga0247526_117235/152-313        UUUCUGUCCCUACUUCGG.....GGGU.GCCAA..ACCCCAAGCACAAGCUGAA........CCGCGAU................AUGCG..........................AUCGCGGCACACUUCCU..CAGCUA....
Ga0247526_110221/603-443        AAUCUGUCCCUACUUCGG.....GGGU.GCCAA..ACCCCAAGCACAAGCUGA.........CCGCCAU................AUGUG..........................AUGACGGAACACUUCCU..CAGCUA....
Ga0247519_110701/607-447        AAUCUGUCCCUACUUCGG.....GGGU.GCCAA..ACCCCAAGCACAAGCUGA.........CCGUCAU................AUGUG..........................AUGGCGGACCACUUCCU..CAGCUA....
Ga0247524_111427/50-210         AAUCUGUCCCUACUUCGG.....GGGU.GCCAA..ACCCCAAGCACAAGCUGA.........CCGUCAU................AUGUG..........................AUGGCGGACCACUUCCU..CAGCUA....
Ga0247529_134913/224-64         AAUCUGUCCCUACUUCGG.....GGGU.GCCAA..ACCCCAAGCACAAGCUGA.........CCGUCAU................AUGUG..........................AUGGCGGACCACUUCCU..CAGCUA....
Ga0247527_115808/280-440        CGUCUGUCCCUACUUCGG.....GGGU.GCCAA..ACCCCAAGCACAAGCUGC.........CAU.CCAU...............AUGUG.........................AUGGAAUGGGUGAUCCCU..CAGCUA....
Ga0247513_128543/241-71         CUUCUGUCUCGGUCAA.......GGGUUCCCA..AGCCCAUGGAACAGG.UAUCUC.GGUUAAUGGUCAUCACACGGC.......CUCGU.................GCAAUGUGUUUGUCCUUAGCCUCAGA..UCUCCAGC..
Ga0247530_109035/489-319        CUUCUGUCUCAGUCAA.......GGGUUCCCA..AGCCCAUGGAACAGG.UAUCUC.GGUUAAUGGUCAUCACACGGC.......CUCGU.................GCAAUGUGUUUGUCCUUAGCCUCAGA..UCUCCAGC..
Ga0247519_103291/1381-1460      CUGCGGUCGGGA......UGU..........................UGAC..................................UUAU...............................................GUCAC....
JGI12272J11983_1319142/230-159  GUGCGGUCGGGGG........................................................................ACCCC.......................................................
Ga0247519_118271/334-405        UAGCGGUCCGG..........................................................................GACAUUA.....................................................
Ga0247526_116548/279-354        AUGCGGUCGGAGG..................................AU....................................CCUAG................................................AU.....
Ga0247529_111070/303-228        AUGCGGUCGGGAG..................................AUC...................................UUA.................................................GAU.....
Ga0247519_113845/334-409        AUGCGGUCGGGAG..................................AUC...................................UUA.................................................GAU.....
Ga0247525_114510/199-318        UGUCUGUCCCGAAC....UA...CAU..CAGGGU..AUG........CAACAGGACCUUCC........................UUCAC..................................GGAAGGUCC..UGUUGACU..
Ga0179944_1313268/129-232       GUUCGGUCCUC.......GAUGA........................AACCC.................................CUGGCCA...........................................GGGUUGA...
Ga0247519_108375/286-206        UAGCGGUCCAGG......G............................GUAUAG................................UAGA.............................................CUAUACA....
Ga0247526_105246/689-508        UCUCUGUCUCUGAUUCG......GGUUUUGCUA.AAACCCAAGGCAUUGGUUCAGUGUGU...CGUUGCUCGGGCCUUUCUUACCUAAAC..........GGUUGUUAGA.CCUGAGUGCU..UUGCAC.UUG..AUCCACC...
Ga0247515_107373/548-731        UCUCUGUCUCUGAUUCG......GGAUUUGCUA.AAUCCCAAGGCAUUGGUUCAGUGUGU...CGUUGCUCAGGCCUUUC.UACCUUAGU..........GGUGUUUGAUACCUGAGUGCUUCUUGCAC.UUG..AUCCACC...
Ga0179955_1048341/123-51        GGUCGGUCCGGU.........................................................................UACAU.......................................................
Ga0179944_1191552/284-411       CGUCGGUCCAUCG.....G............................GGCAUCACAGCG..........................CUUGAC...................................UG.UGUG..AGGCU.....
Ga0179944_1204933/216-89        GGUCAGUCCUUCA.....G............................GGCAUCAU..UGCU........................UACAC..................................AGCGAUGUG..AGGCU.....
Ga0179944_1005311/261-382       GGUCGGUCCUUCC.....G............................GGCAUCACAGCG..........................CAUGAC...................................UG.UGUG..AGGCC.....
Ga0247514_107497/1071-1140      GAGCAGUCCCUC.........................................................................UACG........................................................
Ga0247523_120120/325-403        UAGCGGUCCGCAU..................................GUG...................................UAAUC...............................................CAC.....
Ga0247524_121352/374-303        UAGCGGUCCGGAUC.......................................................................AUCU........................................................
Ga0247524_105844/561-637        UAGUGGUCCAGA......CCA..........................GU....................................GAAU.................................................ACGA...
Ga0247514_114593/280-211        AGGCAGUCCCUC.........................................................................UUUUU.......................................................
Ga0247519_104852/800-730        GUGCGGUCCGG..........................................CCC.............................AUCC........................................GGGU............
Ga0247531_104861/1082-1012      GUGCGGUCCCAUC........................................................................UCCG........................................................
Ga0247518_137824/301-231        GUGCGGUCCCAUC........................................................................UUCG........................................................
Ga0247520_104849/891-821        GUACGGUCCCAUC........................................................................UCCG........................................................
Ga0247526_117664/434-499        CAGCGGUCCAUG.........................................................................UUAU........................................................
Ga0247518_108423/758-837        AAGCGGUCCGU.......U............................AUGUC.................................CAUCA.............................................GACAU.....
Ga0247521_124040/388-309        AAGCUGUCCGU.......U............................AUGUC.................................CAUCA.............................................GACAU.....
Ga0247531_104608/1256-1177      AAGCGGUCCGU.......U............................AUGUC.................................CAUCA.............................................GACAU.....
Ga0247516_124175/202-272        UAGCGGUCUACAC........................................................................UUAU........................................................
                                <<<<....<<<<<<<<<<.....<<<<<......>>>>>........<<<<<<<<<<<<<<<<<<<<<<<<<<<<<<<<<<<<<<...............>>>>>>>>>>>>>>>>>>>>>>>>>>>>>>>>>..>>>>>.....
                                2221....2222211110.....111.0......0.111........222.1..........1.1.1.1.0.11..11....000...............000...11..11.0.1.1.1.1............1.222.....
                                ●●●C●GUCY●●●○----------------------------------○○○-----------------------------------●●●●○-----------------------------------------------○○○-----
```

alignment positions 146 · · · 246

```
Ga0179934_1155244/114-26        ........CGGGAAAUCC.GUGAGGGAAACAUGC.CGUUAGAAUG.........AAAAA..............CAUUCUAUCGAGCAGCACAUUAACUCAC
Ga0247515_120448/91-178         ........AGGGAAGCUG.CUUGGUGAAACACUUUUGUGUUGGUAUCC......UUUAA...........GGA..CCCCUACAAAAGGCAAAUUAUCCAAG
Ga0247518_120275/202-289        ........AGGGAAGCUG.CUUGGUGAAACACUUUUGUGUUC.UC.GUC.....CUU............GAAAGACGGCUACAAAAGGCAAAUUAUCCAAG
Ga0247522_108409/684-554        ...CCUAGAGAGAAGGAUUGCCGGUGAAACAUGCUCGC................CCGCCGAA...................GCGAGUAGCACAUUACCGGU
Ga0179955_1195671/481-563       ....GGUGCAUGAAGCAU.GGUAACGAAACACUCAGACUU..............UAUUUC..................AAGUCUGAGGCACAUUACUUACC
Ga0247529_117158/253-331        ......CUGGGGAAGACU.GAUGGCAAAACACGGGAAAA...............UAAA.....................UUCCCCCGGCACAUUACCCAUC
Ga0179948_1254202/198-296       .....GUGUGGGAAGACA.GCUAGCAAAACACCGCGUUUAAUAUGUUUA.....UUUGGUU........UAAGC.......ACGCGGGCAAAUUACCUAGC
Ga0247528_108647/438-562        .....AGUCAAGAAGUAU.UCCAAGGAAACACACCACUUUGAUCAUCGUGCUAUAGCUA.....AUAGCACUCACAUUA..UUGGUGGCACAUUACUUGGA
Ga0256798_1305521/221-305       ....CAACCGGGAAGAGG.CGUGGGGAAACAUGCUG..................GCACAG......................CAGCAGCACAUUAACCACG
JGI12272J11983_1345183/364-258  .GGA.UGUGGCGAAGAAAUGGGAGGGAAACACAUCAGCGU..............AGCG....................AC.CUGAUGGAAAAUUAACUCCC
JGI12272J11983_1072695/40-150   .....GCGAGAGAAGAGAUGGGAGGGAAACAUGCUGGAGU..............AGCG....................AC.CCAGUAGCACAUUAACUCCC
Ga0179934_1197833/207-115       .....CUGUGGGAAGACU.GGAAGGGAAACACUCU...................ACCA.........................AGAGGUACAUUAACUUCC
Ga0255059_10691632/166-76       .....CCACAUGAAGAUA.GCCAGGGAAACAUACCCGGAA..............CGAA....................UUCCGAGUAGCACCUUAACUGGC
Ga0255062_11230321/200-110      .....CCACAUGAAGAUA.GCCAGGGAAACAUACCCGGAA..............CGAA....................UUCCGAGUAGCACCUUAACUGGC
Ga0247527_116942/161-88         .....UGUCGCGAAGCUU.GCCAGGGAAACAUCGUUUU................UCUU......................AAAACGAGCAUAUUAACUGGC
Ga0247521_112559/317-205        ...CAUAGGCUGAAGCGA.GUGAGGGAAACAUGUAAUUGUGAGUGGUUG.....AUUU...........CAACUGC.UACCAUUGCAGUAAAUUAACUCAC
Ga0247529_116306/255-362        ....UGUCAGGGAAGCAC.GGUAGGGAAACAUGCGUCGCAG.............CCGU...................CUGCUACGCAGCACAUUAACUACC
Ga0179944_1246019/249-162       .....CUUAGGGAAGUGA.GCAAGUGAAACAUGCAGAAGUAG............UGUA..................CUACUUCUGUAGCACAUUACCUUGC
Ga0247517_121471/387-499        ..CAUUCGGCUGAAGCUG.GUGAGGGAAACAUAGUGUUGUGGCUAGAU......AGU.............AUCUAGUUACCAUACUAGAAUAUUAACUCAC

                                >>>>>>>>>>....>>>>.<<<<<.......<<<<<<<<<<<<<<<<<<<<<<<..........>>>>>>>>>>>>>>>>>>>>>>>.........>>>>>
                                0111122222....1222.22221.......222222..........11...11..........11...11..........222222.........12222
                                -----○○●●RRGAAG●●●-R●YAGGGAAACAY●●●●○○---------------●●●●○-----------------------○●●●●RGYA●AUUAACUR●Y
```

```
Ga0247524_126530/125-198          .....GGAUGGGAAGUAA.CUCAGGGAAACACACAUU.................GGCAA..................................AAUGUGGGAAAUUAACUGAG
Ga0247519_107918/217-58           CCGGAGUAGGAGAAGAAU.UCCAGGGAAACACUCCAUC....UGGGUUA.....UUA............UAGCCCAGGGUGAUGGAGGCACAUUAACUGGA
Ga0247529_103231/270-430          CCGGAGUAGGAGAAGAAU.UCCAGGGAAACACUCCAUCGU...GGGUUA.....UUA............UAGCCCAGGGUGAUGGAGGCACAUUAACUGGA
Ga0247526_117235/152-313          CCGGAGUAGGAGAAGAAU.UCCAGGGAAACACUCUGU....CUUGGGUU.....AUUAC..........AGCCCAAGGGUGACAGAGGCACAUUAACUGGA
Ga0247526_110221/603-443          CCGGAGUAGGAGAAGAAU.UCCAGGGAAACACUCCAUCAU....GGGUU.....AUUAC..........AGCCCAAGGGUGAUGGAGGCACAUUAACUGGA
Ga0247519_110701/607-447          CCGGAGUAGGAGAAGAAU.UCCAGGGAAACACUCCAU....CUUGGGUU.....AUUAC..........AGCCCAAGGGUGAUGGAGGCACAUUAACUGGA
Ga0247524_111427/50-210           CCGGAGUAGGAGAAGAAU.UCCAGGGAAACACUCCAU....CUUGGGCU.....AUUAC..........AGCCCAAGGGUGAUGGAGGCACAUUAACUGGA
Ga0247529_134913/224-64           CCGAAGUAGGAGAAGAAU.UCCAGGGAAACACUCCAU....CUUGGAUU.....AUUAC..........AGUCCAAGGGUGAUGGAGGCACAUUAACUGGA
Ga0247527_115808/280-440          CCGAGGUAGGAGAAGAAU.CCCAGGGAAACACCGUAGC.....CUGGCU.....AUUAC..........GGCCAGUGGGUGCUACGGGCACAUUAACUGGG
Ga0247513_128543/241-71           ..UUGGCUGAAGAAGAAGCGUCAGGGAAACACGUAGG.................CUAUUUGGCA.................CCUACGGCACAUUAACUGAC
Ga0247530_109035/489-319          ..UUGGCUGAAGAAGAAGCGUCAGGGAAACACGUAGG.................CUAUUUGGCA.................CCUACGGCACAUUAACUGAC
Ga0247519_103291/1381-1460        ......UCCUGGAAGCAG.UGUGGGGAAACACCAUC..................GAUG........................GAUGGGCACAUUAACCACA
JGI12272J11983_1319142/230-159    .....CCCCUGGAAGCAC.GGCAGGGAAACAUCAUAC.................GUU.......................GUAUGAGCACAUUAACUGCC
Ga0247519_118271/334-405          .......CCGGGAAGCUA.ACCAGGGAAACAUAGACU.................GACAA.....................AGUCUAGUAAAUUAACUGGU
Ga0247526_116548/279-354          .....CCUCGGGAAGCAU.GACAGGGAAACACAUCU..................AUAUC.......................AGAUGGCAAAUUAACUGUC
Ga0247529_111070/303-228          .....CUUCGGGAAGCAU.GACAGGGAAACACAUCUG.................UAU.......................CAGAUGGCAAAUUAACUGUC
Ga0247519_113845/334-409          .....CUUCGGGAAGCAU.GGCAGGGAAACACAUCUG.................UAU.......................CAGAUGGCAAAUUAACUGUC
Ga0247525_114510/199-318          ....GUUCGGGGAAGACA.ACCAGGGAAACACACCGG.................CAAG......................CCGGUGGCACAUUAACUGGU
Ga0179944_1313268/129-232         .......GGGGGAAGAAC.GCCAGGGAAACAUGCAUCGGCGCUC..........ACGUAG..............GAGCGUCGUUGUAGCACAUUAACUGGC
Ga0247519_108375/286-206          ......UCUGGGAAGCUA.GCCAGGGAAACACCACU..................GUU........................AGUGGGAAGAUUAACUGGC
Ga0247526_105246/689-508          .CGGAUCAGAAGAAGAUCUUCCAGGGAAACACGCUUAGG...............AAUUGG...................UCUAAGUGGCACAUUAACUGGA
Ga0247515_107373/548-731          .CGGAUCAGAAGAAGAUCUUCCAGGGAAACACACUUAGG...............AAUUGG...................CUUAAGUGGCACAUUAACUGGA
Ga0179955_1048341/123-51          ......GCCGGGAAGACC.GCCAGGGAAACACACG...................UAUUUGGAAC...................CGUGGUACAUUAACUGGC
Ga0179944_1191552/284-411         .....CGAUGGGAAGACA.GCCAGGGAAACACGCAGUC.UCAGCAUCCUACUCCUUUC......GG...GGGAUGAUCGCGACUACGGUACAUUAACUGGC
Ga0179944_1204933/216-89          .....UGAAGGGAAGACC.GCCAGGGAAACACGUAGUC.UCAGCAUCCUGCC..UUUC........GG.GGGAUGAUCGCGACUGCGGUAAAUUAACUGGC
Ga0179944_1005311/261-382         .....GGGAGGGAAGACC.GCGAGGGAAACACGGC.UAAGCAUCCUACUCC...UUUC.........GG...GGGAUGACAGCGCCGGUACAUUAACUCGC
Ga0247514_107497/1071-1140        ......GAGGAGAGGCUC.GCCAGGGAAACACGCAUC.................UCCA......................GAUGUGGCAUAUUAACUGGC
Ga0247523_120120/325-403          .....AUGUAGGAAGCUA.GCCAGGGAAACAUCAACC.................UUCG......................GGUUGAGCACAUUAACUGGU
Ga0247524_121352/374-303          ....GA.UCGGGAAGCUA.GGUGGGGAAACAUCAUG..................AUUAC.......................CAUGAGGAAAUUAACCACC
Ga0247524_105844/561-637          ......UCUGGGAAACUA.GGUAGGGAAACAUCAUC..................UUUG........................GAUGAGUAAAUUAACUACC
Ga0247514_114593/280-211          ......GAGGAGAAGCCA.GCCAGGGAAACACAUUC..................UUAAU.......................GGAUGGAAAAUUAACUGGC
Ga0247519_104852/800-730          .......CUGGGAAGCAC.GCCAGGGAAACACAAC...................GAAA.........................GUUGGCAUAUUAACUGGC
Ga0247531_104861/1082-1012        .....GAUGAGGAAGCAC.GCCAGGGAAACACAAUC..................UUUAU.......................GAUUGGCACAUUAACUGGC
Ga0247518_137824/301-231          .....GAUGAGGAAGCAC.GCCAGGGAAACACAAUC..................UUUAU.......................GAUUGGCACAUUAACUGGC
Ga0247520_104849/891-821          .....GAUGAGGAAGUAC.GCCAGGGAAACACAAUC..................UUUAU.......................GAUUGGCACAUUAACUGGC
Ga0247526_117664/434-499          ......CAUGGGAAGCUG.GCGAGGGAAACACACU...................AUAU.........................AGUGGGAAAUUAACUCGC
Ga0247518_108423/758-837          .......GCGGGAAGCUU.GCCAGGGAAACACAAUAC.................CUUU......................GUAUUGGGAAAUUAACUGGC
Ga0247521_124040/388-309          .......GCGGGAAGCUU.GCCAGGGAAACACAAUAC.................CUUU......................GUAUUGGGACAUUAACUGGC
Ga0247531_104608/1256-1177        .......GCGGGAAGCUU.GCCAGGGAAACACAAUAC.................CUUU......................GUAUUGGGACAUUAACUGGC
Ga0247516_124175/202-272          .....GUGUGGGAAGCUA.GCGAGGGAAACAUGCAUA.................CGU.......................UAUGCAGCAUAUUAACUCGC
                                  >>>>>>>>>>....>>>>.<<<<<.......<<<<<<<<<<<<<<<<<<<<<<<..........>>>>>>>>>>>>>>>>>>>>>>>.........>>>>>
                                  0111122222....1222.22221.......222222..........11...11..........11...11..........222222.........12222
                                  -----○○●●RRGAAG●●●-R●YAGGGAAACAY●●●●○○----------------●●●○-------------------○●●●●RGYA●AUUAACUR●Y
```

5 hairpin-extra-spruce-stringent

For a description of this alignment, see Supplementary Table 2

5.1 Multiple-sequence alignment

Each ribozyme in this alignment is identified by its genomic location in the form SEQID/START-END. SEQID (the sequence accession) is derived from sources such as IMG/M or GenBank. START is the coordinate of the 5′ nucleotide of the hairpin ribozyme, and END corresponds to the 3′ nucleotide. If START>END, then the ribozyme is on the reverse complement strand.

Nucleotides proposed to basepair as part of the consensus structure are shaded in color when they comprise Watson-Crick or G-U pairs. Otherwise they are shaded gray. Conserved stems are also indicated at the bottom of the alignment by angle brackets, where matching < and > denote base-paired columns. Below these angle brackets, the symbol "2" denotes base pairs exhibiting covariation according to the statistically well-founded R-scape method. "1" denotes base pairs exhibiting covariation according to R2R's simplistic method. "0" denotes base pairs that are not observed to mutate and "?" denotes base pairs that have a significant frequency of non-canonical nucleotides for Watson-Crick or G-U pairs (> 5%). Below these base pair annotation is the consensus sequence: "R" = "A" or "G", "Y" = "C" or "U", red nucleotides: nucleotide identity conserved more than 97% of the time, black nucleotides: 90%, gray nucleotides: 75%, red circle (●): nucleotide is present 97% of the time, black circle (●): 90%, gray circle (●): 75%, white circle (○): 50%. All percentages of sequences just described (e.g. 97% conserved) assume that sequences have been weighted by the GSC algorithm implemented by the Infernal software package.

The alignment begins on the next page.

1828 duplicate sequences are not shown in the alignment

alignment positions 1 · · · 150

```
Ga0316035__118720/195-42        UACUUGUCUUUU.GUAC.AU.C................................................................................................................................
Ga0265747__125480/96-202        CCUUUGAGUUAC.A.......AU...............................................................................................................................
Ga0242657__1353977/135-311      CUUUUGUCCUGA.AAG..................CAUAAUGAUAAGAAGAGUUACGCUUGCGAUGAUGCAAGCAGCUACGAAUUGAUUUAUAGUGGUAGUCCAACAUACAAUAUGUUAUGUAGAU.........................
Ga0242649__1051170/34-143       CAUUUGUCCUGU.UUGA.U..........GUGCAUCUU................................................................................................................
Ga0265758__100138/1527-1633     UGUUUGAGUCGG.G.......AU...UA....CAGUGGAU..............................................................................................................
Ga0184593__103988/348-257       AAUCUGUCCUCG.ACA.............C........................................................................................................................
Ga0316047__106478/13-108        CCACUGUCCUGG.G...............GUUCUCUAUG...............................................................................................................
Ga0213850__1056422/14-139       UGUCUGUCUCGA.C.......AU......GUG......................................................................................................................
Ga0316032__128094/136-241       GUGUUGAGUCGG.GU...................AACAGU..............................................................................................................
Ga0307482__1000311/1469-1363    UCUCGGUCCGGU.AUCC.................UCCGAU..............................................................................................................
Ga0242657__1051800/416-321      CAAUUGUCCCAG.GUCU.....A...............................................................................................................................
Ga0307921__1050750/452-358      UUGUUGUCCCAG.AACU.C...............ACUCUU..............................................................................................................
Ga0265756__117960/316-418       GUUCAGACCACA.UUGU.U...................................................................................................................................
Ga0242654__10352202/387-307     UGACGGUCAGGG.UA.......................................................................................................................................
Ga0242652__1159268/284-150      UCUCUGUCCCUU.GG.......A...A.......U...................................................................................................................
Ga0265744__102237/225-365       CUUCUGUCUCAU.C.......GG...AGAAGGCUCCAGGCAUGGCCUGGGUGAGAAGUAAUAGCUUACCCUC..............................................................................
Ga0316029__101396/632-732       UGACGGUCCUAU.GAC......................................................................................................................................
Ga0265759__100202/139-258       ACACUGUCCAUA.UAGA.UG........AGGCU..GUA................................................................................................................
Ga0179943__1118543/65-150       ACGUUGUCCUUA.AGAU.................GG..................................................................................................................
Ga0182091__1018330/100-226      AUGCAGUAUUUC.G.......AA...AC..........................................................................................................................
Ga0127503__10119710/546-465     GGACAGUGUCUA.GUC..................UU..................................................................................................................
Ga0138277__1022592/399-305      CGGCUGUCCGUU.GUCA.................AUUC................................................................................................................
Ga0138278__1026470/183-287      CGGCUGUCCGUU.GCG..................AAUU................................................................................................................
Ga0138277__1089473/408-304      CGGCUGUCCGUU.GCGA.................AUU.................................................................................................................
Ga0247538__105452/166-82        UGUCAGUGUGGU.................GGGU.UACAAG..............................................................................................................
Ga0247554__102783/498-580       UGUCAGUGUGGU.GGG..................UUAC................................................................................................................
Ga0138291__1177333/295-213      AGUUUGGCUGUG.U....................UAA.................................................................................................................
Ga0210324__1290306/191-291      CCCCUGAGUUGG.GUGG.UG..................................................................................................................................
Ga0242657__1026087/460-322      GUCCUGUCCAUG.GGG.....AU...GA..........................................................................................................................
Ga0307482__1147486/107-4        UAACGGUCCCAA.G.......AC...ACGUGGU.....................................................................................................................
Ga0184573__113483/125-229       CAUCUGUCCUGC.AUAG.CU.GG......CGAGUUGCUA...............................................................................................................
Ga0184576__116834/47-151        CAUCUGUCCUGC.AUAG.CU.GG......CGAGUUGCUA...............................................................................................................
Ga0184568__108419/492-395       UGACAGUCUCAU.AAGU.....................................................................................................................................
Ga0307482__1023050/594-496      GCUCGGUCCUGG.CAUU.....................................................................................................................................
Ga0179955__1013331/439-311      CGUCUGUCUCGA.CAA..................CGUCUCACUCA.........................................................................................................
Ga0184596__112078/314-191       AUACUGUCUGAU.U.......UC.......GU......................................................................................................................
Ga0184602__108471/49-173        AUACUGUCUGAU.U.......UC.......GU......................................................................................................................
Ga0247528__108647/438-562       AUACUGUCUGAU.U.......UC...............................................................................................................................
Ga0316039__120091/354-252       CUACUGUCCCA.......................UGAAAGCAACCCGCAA....................................................................................................
Ga0307484__166952/62-163        UGUCUGUGUUAC.UAUG.....C...............................................................................................................................
Ga0180107__1244437/408-507      GAUCUGUCCAAA.GCC......................................................................................................................................
Ga0265742__1065119/361-273      AUGCUGUCCGCA.CAA..................AUUUGA..............................................................................................................
Ga0316035__113756/173-88        AGUCUGAGGUAC.CAC..................UCGC................................................................................................................
Ga0206355__1134705/241-332      AGUCUGAGUUGU.UUAC.GA.A................................................................................................................................
Ga0180107__1031754/4443-4551    CGUUUGUCCCGU.UACC.G............CCUAUGACAGGCUAG........................................................................................................
Ga0138292__1031932/14-100       CAUCUGUCCUGA.GUU..................AU..................................................................................................................
Ga0316029__100807/1303-1197     CUGCGGUCCUGG.G.......GA...A...........................................................................................................................
Ga0184568__114257/261-413       CUUCUGUCCUUG.AGUG.................AGAAGCCCAGGCCGGGGAAGGCCGAAUGGCGCGACCUUU.............................................................................
Ga0242647__1005188/73-152       CUGUGGUCCGA.......................AGAA................................................................................................................
Ga0153880__1340646/556-457      UGUUUGAAAUUU.UAAG.....................................................................................................................................
Ga0307484__101303/704-866       GUUCUGUCCCUU.CUUC............GGGUUCCCA................................................................................................................
Ga0242641__1025149/251-89       GUUCUGUCCCUU.CUUC............GGGUUCCCA................................................................................................................
Ga0242652__1002960/413-574      GUUCUGUCCCUU.CUUC............GGGUUCCCA................................................................................................................
Ga0265754__1007706/448-552      UGCUUGUCCUAC.AGG.....GU......GGUUC....................................................................................................................
Ga0157562__1164332/672-560      GGUCUGACCACC.U.......AU...AGCAUUGUUUACA...............................................................................................................
Ga0157569__1058012/474-586      GGUCUGACCACC.U.......AG...AGCAUUGUUUACA...............................................................................................................
Ga0307921__1027128/160-73       UGACUGUCCGAG.AAC..................AAGC................................................................................................................
Ga0138279__1083071/419-326      CUGCGGUCCGUA.G........................................................................................................................................
Ga0127502__10704012/113-7       AUCUUGAGUCGG.G.......AU...UAC.........................................................................................................................
Ga0222728__1000095/4541-4435    UGUUUGAGCUGG.G.......UU...AAC.........................................................................................................................
Ga0265750__1166784/261-415      GUCCUGUCCACA.A..U.GG.UU...GAA.........................................................................................................................
Ga0310115__109341/244-400       GUCCUGUCCACA.A..U.GG.UU...GAA.........................................................................................................................
Ga0265756__158829/72-188        UGACGGUCCGGA.U........................................................................................................................................
Ga0184583__104740/342-477       GUCCUGUCCUUA.CAG..................AUGAAGCGCUAGGCGACCCCGUAUCAG.........................................................................................
Ga0180107__1215312/308-184      GUCCUGUCCAUA.GG......UU...GAACGCUU....................................................................................................................
Ga0265742__1013613/579-453      AGUCAGUACCAG.G.......UG...G..CGCACUACAC...............................................................................................................
Ga0265748__110851/279-405       AGUCAGUACCAG.G.......UG...G..CGCACUACAU...............................................................................................................
Ga0265752__120463/277-403       AGUCAGUACCAG.G...............UGGUGCACUA...............................................................................................................
Ga0265759__115288/4-102         AGACAGUCCACA.GGU..........C.......AU..................................................................................................................
Ga0242646__1001122/826-995      GAUCUGUCCGCG.CACA.C..........GGAUUCCUGA...............................................................................................................
Ga0157593__1101573/244-147      UGACUGUCCCAU.GAG..................GAGCUC..............................................................................................................
Ga0184597__102941/191-111       GAACGGUCCAGU.GUAU.................CCU.................................................................................................................
Ga0184597__115944/155-80        UAUCUGUAUCUG.GUUU.................GU..................................................................................................................
Ga0265742__1051722/222-295      UUGCUGUCUCGG.CUU..................GU..................................................................................................................
Ga0180107__1229893/426-318      AGUCUGAGUUGA.AAUC....U.......CCGCCUGCG................................................................................................................
Ga0242654__11312609/202-83      AAACGGUCCGAG.AG.......................................................................................................................................
Ga0187793__1464320/616-535      UCACUGUCCUGG.U....................GG..................................................................................................................
Ga0187794__1206392/88-6         UCACUGUCCUGG.U....................GAC.................................................................................................................
Ga0316034__129624/151-284       UUCCUGUCCCAG.AG.......U...GAU.........................................................................................................................
Ga0184576__119948/531-664       UUCCUGUCCCAG.AG.......U...GAU.........................................................................................................................
Ga0184568__109070/428-286       GUUCUGUCCUUU.GCA..................GUCGAGCUCUAGGCAGUAGAAUACCACCUCCCAUA.................................................................................
Ga0265756__100717/910-1050      GUUCUGUCCAAC.GUGG.UG.AC...A...........................................................................................................................
Ga0179955__1195671/481-563      AUGCGGUAUGCA.UC.......................................................................................................................................

                                <<<<....<<<<.<<<<.<<.........<<<<<....................................................................................................................
                                2222....2222.22.......................................................................................................................................
                                ●●●C●GUCC●●●−◦◦−−−−−−−−−−−−−−−−−−◦◦◦−−−−−−−−−−−−−−−−−−−−−−−−−−−−−−−−−−−−−−−−−−−−−−−−−−−−−−−−−−−−−−−−−−−−−−−−−−−−−−−−−−−−−−−−−−−−−−−−−−−−−−−−−−−−−−−−−−
```

```
Ga0184579__120620/502-584      CUAUGGUCCGGA.ACC..................GCU.................................................................................................................
Ga0127502__10711632/191-294    UAUCGGUCCAGA.UACU............GGC..GAAA................................................................................................................
Ga0310119__125223/193-319      UCUCUGUCUGCC.UUCG.GU.AA................................................................................................................................
Ga0307482__1064439/425-316     AAUCAGUCCUAU.CAU.............UGAUGGUUUC...............................................................................................................
Ga0307484__102002/698-552      UUCCUGUCCUAC.AAG.....AU...GAA.....GCGCUAGGCGGAAGCCCGUAGAACUAGU........................................................................................
Ga0265741__107029/232-320      CAGCUGUCCU........................UUA.................................................................................................................
Ga0247515__120448/91-178       CAGCUGUCCU............................................................................................................................................
Ga0265759__103410/556-638      CAGCUGUCCU............................................................................................................................................
Ga0247534__101509/435-523      CAGCUGUCCU........................UUA.................................................................................................................
Ga0310102__119240/132-46       CAGCUGUCCUA...........................................................................................................................................
Ga0265742__1084621/112-29      CAGCUGUCCU............................................................................................................................................
Ga0310114__135156/99-185       CAGCUGUCCU........................UAA.................................................................................................................
Ga0265752__102595/694-607      CAGCUGUCCU........................UAA.................................................................................................................
Ga0265756__105276/677-589      CAGCUGUCCU........................UUA.................................................................................................................
Ga0153880__1339022/280-371     CAGCUGUCCU........................UUA.................................................................................................................
Ga0247518__120275/202-289      CAGCUGUCCU............................................................................................................................................
Ga0265752__100023/1618-1523    CAUCGGUCCCAU.................AGUCCAAGA................................................................................................................
Ga0307484__102966/353-250      UAACGGUCCCAU.G.......AC...AC..........................................................................................................................
Ga0265752__101001/521-687      AUUCUGUCCCUU.GAGA.G...................................................................................................................................
Ga0184593__121910/718-622      AUGCAGUACUAG.A........................................................................................................................................
Ga0184602__103319/96-192       AUGCAGUACUAG.A................UUUCUCCUA...............................................................................................................
Ga0247528__108722/131-33       AUGCAGUACUAG.A................UUUCUCCUA...............................................................................................................
Ga0265757__143567/145-70       CGUUUGUCCUGC.UUU......................................................................................................................................
Ga0184592__121084/378-271      CUGUUGAGCCGG.G.......UA...A........ACUG...............................................................................................................
Ga0316037__172473/89-196       UCGCUGAGCCGG.G.......UA...A........ACUG...............................................................................................................
Ga0307921__1011071/674-791     CGACUGAGGUUG.ACUC.....................................................................................................................................
Ga0265744__124024/357-288      CUACGGUACGGU.C....................CA..................................................................................................................
Ga0307482__1000291/592-698     UGUCAGUCUCCC.U..A.GG..........ACUCUUCGAGUUGCC.........................................................................................................
Ga0184568__108869/934-1072     CUUCUGUCCAUG.UU......G...AGAAGACUCCACGCCAUGACGUGGUUGACUUGAUCAGCCUU...................................................................................
Ga0138276__1121602/329-410     CUGCUGUCCAGC.GAG..................UUU.................................................................................................................
Ga0138278__1041650/183-102     CUGCUGUCCAGC.GAAU.................UUC.................................................................................................................
Ga0138277__1137481/570-489     CUGCUGUCUAGC.AAA..................CUUC................................................................................................................
Ga0138278__1041788/293-378     CUGCUGUCCAGC.AAA..................CUUC................................................................................................................
Ga0138275__1223952/371-283     CUGCUGUCCAGC.AAA..................CUUC................................................................................................................
Ga0138277__1142657/273-361     CUGCUGUCCAGC.AAA..................CUUC................................................................................................................
Ga0307482__1202605/45-154      UUUCGGUCC.UA.UGAC.C..........CUCUCUCUG................................................................................................................
Ga0180107__1017343/1163-1243   CUGCAGUACACU.G....................AA..................................................................................................................
Ga0157560__1170105/128-245     GGGCGGUCCUAC.AGU.....UA...............................................................................................................................
Ga0316030__137115/325-257      GAUCAGUCCGU...........................................................................................................................................
Ga0316046__103474/576-502      UUACGGUCCUCC.U........................................................................................................................................
Ga0265754__1141843/290-185     AGUUUGUCCCAG.AGUC.C...................................................................................................................................
Ga0222729__1080556/362-213     AAUCUGUCCUU................UA.........................................................................................................................
Ga0265740__1020297/298-150     UGUUUGUCUUGU.AAAC.AC..................................................................................................................................
Ga0157569__1080558/455-605     CGACUGUCCCGC.GU..............CAGUCCGAUU...............................................................................................................
Ga0307484__101264/723-941      CUUCUGUCCCUU.GA..............GAG..AGAGCUCAGGCCGAGCAAGAGCGUCAAGUCCCGUGUCAAUUCACGGGCCAGUCCGUAUUCCAGACUGGGUAGGAAUGAUGGUACCUACCGUCUCGACGACAUCCUGUUGUCAGACG
Ga0213850__1513954/279-196     GGACGGUCCCGG.GG...................UAUAC...............................................................................................................
Ga0316053__117700/109-179      CGAUGGUCUUCA.U....................UUA.................................................................................................................
Ga0184582__101997/109-1        GAGCGGUGUGAU.UC.......................................................................................................................................
Ga0184602__109114/416-317      UCACUGUGUGUG.CGUA.C...............CUACUAG.............................................................................................................
Ga0153880__1354682/355-454     UCACUGUGUGUG.CGUA.C...............CUACUAG.............................................................................................................
Ga0184602__110257/415-316      UCACUGUGUGUG.CGCA.C...............CUAC................................................................................................................
Ga0307484__149222/136-234      UCACGGUCCAUG.GUGU.....................................................................................................................................
Ga0184572__111224/187-319      AACCUGUCUAUC.UGGA.U..........CGGUGC...................................................................................................................
Ga0138278__1022898/219-127     CGUCGGUCCUCA.A........................................................................................................................................
Ga0184577__108791/182-295      CGUCGGUGCGGC.ACUC....AA...GAA.........................................................................................................................
Ga0265748__107452/280-387      AGUCUGUCCACA.UUGU.CG.AC...CCG.........................................................................................................................
Ga0180107__1164033/560-431     AGUCCGUCCUUC.GCGU....C.....AU.........................................................................................................................
Ga0032354__1086862/688-579     ACCCUGUCCUGA.UCCG.AA.AC......UGACACGUGA...............................................................................................................
Ga0265751__100560/274-382      CAUCGGUCCUAU.CG......U....GCUCCGUCCU..................................................................................................................
Ga0180107__1045142/740-541     CUUCUGUCUUCA.CUUC.C..........GGACUCCCAA...............................................................................................................
Ga0213850__1296465/305-234     AAGCUGUCAGGU.CGAA.....................................................................................................................................
Ga0265757__111747/356-286      AAGCUGUCAGGU.UGAA.....................................................................................................................................
Ga0247553__104889/634-563      AAGCUGUCAGGU.UGAA.....................................................................................................................................
Ga0265751__102572/231-125      UAUCGGUCCCUA.U.GA.UG.GC...AA.UGG......................................................................................................................
Ga0265758__100017/4028-4134    UAUCGGUCCCUA.U.GA.UG.GC...AA.UGG......................................................................................................................
Ga0180107__1307934/226-315     UUUCUGUCCGGG......................CAUCUACCAG..........................................................................................................
Ga0184572__112077/359-259      UCUCGGUCCCAC.CAU..................GACACGCCCAAG........................................................................................................
Ga0213850__1371453/186-298     UGUCGGUCCAAA.CAGA.................UUGUG...............................................................................................................
Ga0213850__1469439/119-231     UGUCGGUCCAAA.GAGA.A...............UGCG................................................................................................................
Ga0247535__113123/229-119      CAGCUGUCCUCU.CCUC.GU..............A...................................................................................................................
Ga0316036__112404/482-376      CAGCUGUCCUUC.CUCG.....................................................................................................................................
Ga0179943__1118543/263-176     CCGUUGUCUCAC.CC.......................................................................................................................................
Ga0180108__1207316/715-601     UCUUUGUCCGU.......................UCCAGUCACCCCUCGG....................................................................................................
Ga0184578__120646/150-246      CAGCUGUCCAAG.A.......CU...............................................................................................................................
Ga0127503__10678853/419-333    UGCUUGUCCUGU.CUC..................GAUUUA..............................................................................................................
Ga0307919__1065410/76-162      UGCUUGUCCUGU.CUC..................GAU.................................................................................................................
Ga0180108__1209503/1932-2029   GGUCUGUCCUAG.CACU....GU...GAG.........................................................................................................................
Ga0180108__1022680/3902-3998   AGUCAGUCCUAA.AGAU....GU...AAG.........................................................................................................................
Ga0316031__107021/319-214      UUUCUGUCCACC.AAUC.C...............GUGCGUCGGCAGAACCAAGCGGU.............................................................................................
Ga0247527__116942/161-88       AAGCGGGGCGGC.A........................................................................................................................................
Ga0307484__100434/1330-1210    UGUUUGUCCGGU.GACC....A............AUCGGGAAGGUC........................................................................................................
Ga0307482__1160392/324-400     GUUCAGUCCCCU......................AGAC................................................................................................................
Ga0316033__105623/339-253      CGCUGGUCCUGC.UG...................ACG.................................................................................................................
Ga0265754__1037910/322-179     UCUCUGUCCUUA.GAGA....UG...AAGCGCU.....................................................................................................................
Ga0307482__1048753/383-528     GUCCUGUCCACC.A.......U.............UGAAU..............................................................................................................
Ga0265752__102479/223-81       GGUCUGUCUUGC.U....................CCCC................................................................................................................
Ga0265746__1084801/20-158      GGUCUGUCUUGC.U....................CCCC................................................................................................................
Ga0265756__107066/520-660      GGUCUGUCUUGC.U....................CCCC................................................................................................................
Ga0265742__1022009/340-200     GGUCUGUCUUGC.U....................CCCC................................................................................................................

                               <<<<....<<<<.<<<<.<<.........<<<<<....................................................................................................................
                               2222....2222.22.......................................................................................................................................
                               ●●●C●GUCC●●●-○○-------------------○○○-----------------------------------------------------------------------------------------------------------------
```

```
Ga0265748_106902/213-353              GGUCUGUCAUGC.U....................CCCC................................................................................................................
Ga0180052_116799/419-501              AGUCUGUCUCGC.A....................CUGGUAA.............................................................................................................
Ga0115594_1106220/441-334             GGUCGGUCCUAG.U........C.............AUA...............................................................................................................
Ga0247534_117029/65-195               UCGCUGUCCUGC.U.......AA...........CCUAAC..............................................................................................................
JGI12272J11983_1072695/40-150         UCUUUGUCCUCG.C........................................................................................................................................
Ga0247552_100007/4311-4420            UAUCGGUCCUAC.ACCU.CU.......CU.........................................................................................................................
Ga0184596_107732/313-205              UAUCGGUCCUAC.ACCU.CU.CU...............................................................................................................................
Ga0247542_100007/609-500              UUUCGGUCCUAC.ACCU.CU.CU...............................................................................................................................
Ga0247534_100047/1899-2008            UAUCGGUCCUAC.ACCU.CU.CU...............................................................................................................................
Ga0184598_129111/512-403              UAUCGGUCCUAC.ACCU.CU.CU...............................................................................................................................
Ga0184597_128933/573-464              UAUCGGUCCUAC.ACCU.CU.CU...............................................................................................................................
Ga0184582_108821/1050-1159            UAUCGGUCCUAC.ACCU.CU.CU...............................................................................................................................
Ga0247541_100010/3268-3377            UUUCGGUCCUAC.ACCU.CU.CU...............................................................................................................................
Ga0184580_121439/573-464              UUUCGGUCCUAC.ACCU.CU.CU...............................................................................................................................
Ga0265756_101275/650-541              UAUCGGUCCUAC.ACCU.CU.CU...............................................................................................................................
Ga0184575_101094/586-477              UAUCGGUCCUAC.ACCU.CU.CU...............................................................................................................................
Ga0184594_103311/207-316              UAUCGGUCCUAC.ACCU.CU.CU...............................................................................................................................
Ga0247554_100006/1592-1485            UAUCGGUCCUAC.ACCU.CU.CU...............................................................................................................................
Ga0247522_108409/684-554              GUCCUGUCCUCU.AGG.....AU...GAA.........................................................................................................................
Ga0247534_100003/3147-3245            GUUCGGUCCCAU.AAC................U.....................................................................................................................
Ga0265751_100393/1594-1698            UUUCUGUCCUAC.ACCC............G.UGUGGCA................................................................................................................
Ga0265751_124807/392-283              UAUCGGUCCCAU.UAC..........GC.UGCAUCUUCG...............................................................................................................
Ga0310107_144222/327-436              UAUCGGUCCCAU.UAC.....GC......UGCAUCUCCG...............................................................................................................
Ga0265743_103007/946-842              AUUCAGUCCUCU.AUUC....GU...GCA.........................................................................................................................
Ga0316038_107364/55-195               CCGCUGUCCUUU.UG......AA...............................................................................................................................
Ga0184593_123391/157-297              CCGCUGUCCUUU.UG......AA...............................................................................................................................
Ga0222728_1003482/1262-1107           GUUCUGUCCUGC.GU..............CGGACUCUAA...............................................................................................................
Ga0242654_10701525/53-296             UCUCUGUCCCUG.CAUU.C..........GGACUCCCAA...............................................................................................................
Ga0242654_11172691/285-48             UCUCUGUCCCUG.CAUU.C..........GGACUCCCUA...............................................................................................................
Ga0316038_100042/1676-1569            UCUCUGUCCUGA.G...............UCUCGUCCAUU..............................................................................................................
Ga0307921_1043977/91-164              UAUUUGUCCAAC.A........................................................................................................................................
Ga0079039_1293848/95-22               CUGCGGUCCGUG.C....................UAUUA...............................................................................................................
Ga0247534_101560/228-122              GGUCUGUCCCCU.GACC.G............CUCUAAAG...............................................................................................................
Ga0153880_1379927/66-144              GUUCUGUCCCGG.UU...................AGAU................................................................................................................
Ga0153880_1391790/62-140              GUUCUGUCCCGG.UU...................AGA.................................................................................................................
Ga0307482_1048660/422-625             CUUCUGUCCCGU.UGG..................AGAGAGCCUAGGCAGGGUCUGGUAGACGUGAGUAUUACUCUCACAGGGCCGUGAGCCGUA........................................................
Ga0180108_1022680/4248-4153           GAAUUGUCCGUA.GCCA....U............UAU.................................................................................................................
Ga0247516_125012/316-428              CUUCUGUCCGUC.AAA.....AU......GAAGCACCUAG..............................................................................................................
Ga0242652_1038498/365-470             UAUCUGUCCUGU.GGU......................................................................................................................................
Ga0210323_1050281/92-187              CUUGAGUCCUCU.G...............UGG......................................................................................................................
Ga0224712_10542545/154-227            UAAUUGUCCACU..........................................................................................................................................
Ga0184602_130775/1275-1431            UUACUGUCCCUC.UGAG....AU...G.AAGCCUUAAA................................................................................................................
Ga0206355_1311803/144-334             UGUCUGUCGUCA.UAUC.CG.AC...GGU....UGGAUG...............................................................................................................
Ga0310119_152175/242-389              GCUCUGUCCUUU.CUAG....AU...............................................................................................................................
Ga0255059_10691632/166-76             UAUCAGUGUGUG.G.......A................................................................................................................................
Ga0255062_11230321/200-110            UAUCAGUGUGUG.G.......AA...............................................................................................................................
Ga0265743_143797/241-325              UGUCGGUCCCUG.A...............AGAGUAGCGA...............................................................................................................
Ga0247534_117055/74-212               CCUCUGUCCUCU.GGAG.AGCUC...AAUCGACCACACAA..............................................................................................................
Ga0213850_1282564/267-350             GAGCAGUCCCCC.................GGCCCCCGAAA..............................................................................................................
Ga0315826_123252/262-347              GAGCAGUCCCCC.................GGCCCCCGAAA..............................................................................................................
Ga0315817_133437/498-413              GAGCAGUCCCCC.................GACCCCCGAAA..............................................................................................................
Ga0184577_116723/415-311              GAUCAGUCCCAU.UCCC.................UUCGGCUA............................................................................................................
Ga0184576_109717/432-536              GAUCAGUCCCAU.UCC..................CUUCGGCUACCAA.......................................................................................................
Ga0184572_113323/230-300              GGUCAGUCCCUU.UC...................UU..................................................................................................................
Ga0179935_1139075/165-78              CCAUUGUCCUCG.ACU..................UGGA................................................................................................................
Ga0179944_1246019/249-162             UCAUUGUCCUAA.G........................................................................................................................................
Ga0213850_1307525/101-186             AGGCUGUCCGCG.GU...................GUA.................................................................................................................
Ga0222728_1062510/166-66              CUGCGGUCUUCC.CGCG.CU.AU...UAUAGAGUAU..................................................................................................................
Ga0265743_101198/448-619              CGUCUGUCUUCA.GUCC.G..........GAAUUUGACUA..............................................................................................................
Ga0180108_1209503/2438-2343           AGACAGUCCGUG.ACUG.................AAGACA..............................................................................................................
Ga0184583_103998/587-740              AUUCUGUCCAUC.G.......GC...G............AU.............................................................................................................
Ga0184595_100702/414-262              AUUCUGUCCAUC.G.......GU...GAU.........................................................................................................................
Ga0184592_125105/67-219               AUUCUGUCCAUC.G.......GU...GAU.........................................................................................................................
Ga0224712_10002011/1564-1454          UGUCUGUCCUUA.UAUC.GC.........GGAUCUCCG................................................................................................................
Ga0316049_114344/181-288              GCGCUGUCCUGG.CAC..................UUUUGACCUUCGAUA.....................................................................................................
Ga0184576_110594/6-113                GCGCUGUCCUGG.CACU.....U...UU..........................................................................................................................
Ga0316037_112281/325-219              GCGCUGUCCUGG.CAC.....UU...UU..........................................................................................................................
Ga0316046_116376/278-384              GCGCUGUCCCGG.CAC.....UU...G...........................................................................................................................
Ga0247534_102735/124-18               GCGCUGUCCUGG.CAC.....UU...G...........................................................................................................................
Ga0247540_102569/210-104              GCGCUGUCCUGG.CAC.....UU...G...........................................................................................................................
Ga0247529_116306/255-362              GCGCUGUCCUGG.CA......CU...UU..........................................................................................................................
Ga0184568_104528/922-1000             UGUCUGUCCUAC.G....................UG..................................................................................................................
Ga0184578_112031/156-44               CAGCUGUGGUUG.GAUG....GU...AAA.........................................................................................................................
Ga0247517_121471/387-499              CAGCUGUGGUUG.GAUG....GU...AAA.........................................................................................................................
Ga0184569_108885/200-88               UCGCUGUGGUUU.AUG.....GU...AAA.........................................................................................................................
Ga0247521_112559/317-205              UCGCUGUGGUUU.AUG.....GU...AAA.........................................................................................................................
Ga0247539_101233/414-526              UCGCUGUGGUUU.AUG.....GU...AAA.........................................................................................................................
Ga0265756_100485/1108-1000            UGGCCGUCCGCA.G........................................................................................................................................
Ga0265751_100417/1480-1588            UGGCUGUCCGCA.G........................................................................................................................................
Ga0213850_1295854/263-370             CCACUGUCUACG.ACGU.................ACAG................................................................................................................
Ga0307919_1029784/253-144             CCACUGUCCAUG.ACGU.A...............CUAGG...............................................................................................................
Ga0307921_1042549/343-452             CCACUGUCCAUG.ACGU.A...............CUAGG...............................................................................................................
Ga0307484_102966/727-825              AGUCUGUCCUAU.UG.............U.....GCCAAU..............................................................................................................
Ga0184588_131258/156-253              GAGCGGUCCAUG.C.......AA...A..........U................................................................................................................
Ga0224712_10010317/1973-2082          CGUCUGUCCCUA.AAGU.C..GC......GGGAUUU..................................................................................................................
Ga0224712_10601975/393-502            CGUCUGUCCCUA.AAGU.C..GC......GGG..AGAAAU..............................................................................................................
Ga0222728_1000118/2272-2196           GGUCGGUCCCUA.CCU......................................................................................................................................
Ga0115594_1200134/154-31              GGUCGGUCCCAA.CCG......................................................................................................................................
Ga0115595_1153837/320-197             GGUCGGUCCUAG.CCGC.................AUGCCCGUGCC.........................................................................................................

                                      <<<<....<<<<.<<<<.<<.........<<<<<....................................................................................................................
                                      2222....2222.22.......................................................................................................................................
                                      ●●●C●GUCC●●●-∘∘-------------------∘∘∘-----------------------------------------------------------------------------------------------------------------
```

```
Ga0255282_1067513/33-105      CGGCGGUCCAGC.C....................GAG.......................................................................................................
Ga0242647_1005934/451-617     UCUCUGUCUCCA.UCGC.GG.UU...ACC...............................................................................................................
Ga0307482_1449537/408-223     GUUCUGUCCCUG.CAAG.U.........GGCCUUUUACA.....................................................................................................
Ga0115594_1022316/346-239     GGUCGGUCCUAG.G.......AU...UA................................................................................................................
Ga0115595_1072788/334-441     GGUCGGUCCUAG.G.......AU...UA................................................................................................................
Ga0184568_108419/50-147       AGUCUGUCCUUU.UUGU.CG.AU.....................................................................................................................
Ga0247534_100007/116-225      AAUCUGUCCAUC.UUUU.C...............GCCUCCUUCGGGGGAA..........................................................................................
Ga0184569_109084/220-111      AAUCUGUCCAUC.UUUU.C...............GCCUCCUUCGGGG.............................................................................................
Ga0316033_100036/207-98       AAUCUGUCCAUC.UUUU.C...........GCCUCCUUU.....................................................................................................
Ga0184578_112042/249-140      AAUCUGUCCAUC.UUUU.C...........GCCUCCUUU.....................................................................................................
Ga0184594_123034/256-94       CGUUUGAGUCUU.CG...................AUUG......................................................................................................
Ga0256798_1305521/221-305     CCUCUGUCCGGU.UG.............................................................................................................................
Ga0184595_116681/414-266      GUUCUGUCCCCA.GAGA....UG...AAG.....UGCUAG....................................................................................................
Ga0184568_105547/129-239      UACUUGUCCCUU.CUG..................CUGACUUGAUCUCUAUG.........................................................................................
Ga0157559_1099797/360-209     GGUUGGUCCGAG.CG....G.CU......UAUGUAAUAU.....................................................................................................
Ga0157572_1089974/360-209     GGUUGGUCCGAG.CG....G.CU......UAUGUAAUAU.....................................................................................................
Ga0316037_108094/19-160       UAUCGGUCCUUC.AUCU.AA.C............UGAG......................................................................................................
Ga0316038_107116/33-174       UAUCGGUCCUUC.AUCU.AA.C............UGAG......................................................................................................
Ga0210323_1072445/691-543     GUCCUGUCCACC.A.......UA...GUA...............................................................................................................
Ga0307482_1000707/704-773     GUGCGGUCUGGC.G....................GA........................................................................................................
Ga0079039_1671802/197-34      CGUCGGUCCUUU.CCG..GG.CU...CCACCCCUUCCAA.....................................................................................................
Ga0247533_103342/137-220      UUUCUGUCCUCA......................AACU......................................................................................................
Ga0157565_1016893/233-334     UUACGGUCCUAG.CUU..................CA........................................................................................................
Ga0180047_1087048/281-180     UUACGGUCCUAG.CUU..................CA........................................................................................................
Ga0157593_1006201/239-338     UUACGGUCCUAG.CUU............................................................................................................................
Ga0157560_1010344/357-456     UUACGGUCCUAG.CUU............................................................................................................................
Ga0316049_154526/155-74       UCUCUGUCCCGG.UA...................CUUA......................................................................................................
Ga0316035_177575/224-142      UCUCUGUCCCGG.UA...................CU........................................................................................................
Ga0316033_101029/151-49       GGGCGGUCCG....GAU.GG.G......................................................................................................................
Ga0206356_10600373/215-21     CGUCUGUCAUCA.GG......A............ACCUAAGCCGGAUGGAUCAUAAAGUCCUGGCCGGUA......................................................................
Ga0307482_1009758/562-669     CAUCUGUCCCAA.CGUC.GA.........GCCAU..........................................................................................................
Ga0213850_1245299/581-409     AGAUUGUCCGUA.GUCA.AC.AACAAAUAUAAUUCACAAAAGUUGCGAA...........................................................................................
Ga0180107_1134582/382-542     UGUCUGUCCUGACGCCA.AG.A....AUGCUACUUGGUC.....................................................................................................
Ga0307484_100970/951-1026     CAGCGGUCCGGC.GC...................AU........................................................................................................
Ga0307919_1034281/527-451     CCACUGUCCUAA.GAC..................UAA.......................................................................................................
Ga0307921_1020694/406-483     CCACUGUCCUAA.GAC............................................................................................................................
Ga0316033_105623/3-98         AGUCUGUCCACA.CG...................GACCGCGCAACCGC............................................................................................
Ga0265743_111644/592-512      UGUCUGUACGGU.G....................AAU.......................................................................................................
Ga0265755_108708/373-453      UGUCUGUACGGU.G....................AAU.......................................................................................................
Ga0316038_121313/184-264      UGUCUGUACGGU......................GAA.......................................................................................................
Ga0265756_109326/116-196      UGUCUGUACGGU.G....................AA........................................................................................................
Ga0265759_104327/339-243      CCUCUGUCCUCC.C..............................................................................................................................
Ga0316035_144545/195-301      CCUCGGUCCAUU.GAC..................UUUUU.....................................................................................................
Ga0265743_108998/150-253      UUUCUGUCCCAC.AGUC............CG.............................................................................................................
Ga0153880_1067318/492-566     ACUCUGUCCCGU.A....................AAA.......................................................................................................
Ga0180044_1047678/444-368     CGACUGUCCCAG.GUU..................CGU.......................................................................................................
Ga0157564_1065218/271-353     CGACUGUCCGUG.UGCA.................CG........................................................................................................
Ga0180044_1069332/269-187     CGACUGUCCGUG.UGCA.................CGU.......................................................................................................
Ga0242647_1014694/423-336     GAUUGGUCCAGC.U.............A................................................................................................................
Ga0180108_1143860/199-116     CUGCUGUCAGG.................................................................................................................................
Ga0265744_100073/1235-1401    UAUCUGUCUCUG.UUAU............GGAAUCCCAA.....................................................................................................
Ga0307482_1054608/326-205     CAGCUGUCCGCU.UA.............................................................................................................................
Ga0242646_1008525/229-119     CAGCUGUCCGCU.UA.............................................................................................................................
Ga0153880_1260556/441-589     GCUCGGUCCAAG.C............ACA...............................................................................................................
Ga0127503_10814644/496-352    CCUCUGUCCAUG.AUAG.GU.GA...AAACGGCAUAGAAA....................................................................................................
Ga0326631_105052/171-77       UCACCGCGU.CC.UUGC.AA.........UGGGCUAUUU.....................................................................................................
Ga0307920_126542/349-261      UUUCGGUCCCAA.CUCG.C.........................................................................................................................
Ga0247533_100162/1643-1535    UUUCUGUCUCCA.GAUC.........CGUGGGCGUAU.......................................................................................................
Ga0316047_100015/734-626      UUUCUGUCUCCA.G.......AU...CCGUGAGCGGUU......................................................................................................
Ga0265743_101537/1064-984     AUGUGGUCCAUG.A....................GAGUCAAGGUC...............................................................................................
Ga0316030_100208/805-700      UAUCUGUCCAUC.AGUC............CGU............................................................................................................
Ga0316050_103193/566-460      UAUCUGUCCCUC.AGUC.CG.........UGC............................................................................................................
Ga0184601_113231/247-149      UGUCAGUACCAU.GC.......U...GAGGGGCGUGCGAG....................................................................................................
Ga0307350_148584/139-224      ACUCUGUCCAU.......................GUACCU....................................................................................................
Ga0307482_1018929/1182-1081   CCUCUGUCCCAU.ACG.....A............UCAUGCACAAGUC.............................................................................................
Ga0307482_1000262/4800-4699   UCUCUGUCCCAU.ACG.....A............UCAUGCACAAGU..............................................................................................
Ga0316047_100860/1040-888     UGUCUGUCCCGG.ACUC....AU...CA......UGCACUAGGCAAGUGACAGGUAGCAACGCUGGCACAGGAUGCUGGAACCUAGUUACAAAAGGU.........................................
Ga0242654_10068518/113-218    GUGUUGUCCGAU.UU..............CGU............................................................................................................
Ga0222729_1010500/11-117      GUGUUGUCCGAU.UU..............CGUCAUGGGG.....................................................................................................
Ga0307484_101172/602-495      GUGUUGUCCGAU.UU.......C...GUC.....AUGGGGCAGUCG..............................................................................................
Ga0242647_1002406/915-1021    GUGUUGUCCGAU.UU.......C...GU......CAUGGGGGUGUC..............................................................................................
Ga0307483_1000720/1178-1064   CCUCUGGACUGA.UUUC.GU.G............GGACCACCAAAGCGUGG.........................................................................................
Ga0307921_1022947/450-369     GGUCUGUCCAC.......................CUA.......................................................................................................
Ga0153880_1432604/289-395     GGUCGGUCUUGG.........U......................................................................................................................
Ga0247533_101442/511-432      UGUUUGACCAGU.GA...................UU........................................................................................................
Ga0242647_1002473/759-521     UCUCUGUCCCUG.UUAC............GGACUCCCGA.....................................................................................................
Ga0256352_1087084/23-118      GGUCUGUCCUCC.A....................CUAAAU....................................................................................................
Ga0157562_1157806/189-74      GGUCUGUCCUCC.A....................UAAAU.....................................................................................................
Ga0153880_1457361/236-121     GGUCUGUCCUCC.AUA............................................................................................................................
Ga0157559_1128929/201-87      GGUCUGUCCACC.A....................UAAC......................................................................................................
Ga0157566_1012736/497-384     GGUCUGUCCUCC.A....................UAA.......................................................................................................
Ga0157579_1155702/323-436     GGUCUGUCCUCC.A....................UAAGU.....................................................................................................
Ga0316036_116159/257-326      ACUUGGUCCGAC......................AGA.......................................................................................................
Ga0180047_1032966/152-37      GGUCUGUCCUCC.A....................UAAAU.....................................................................................................
Ga0184587_125934/78-152       AUGCAGUCCCAU.C....................UA........................................................................................................
Ga0179934_1155244/114-26      GGAUGGUCCG..................................................................................................................................
Ga0307482_1623775/241-347     CGUCUGUCCUUG.AAAC.U...........G.UCUUAAAU....................................................................................................
Ga0307484_101582/1018-1124    CGUCUGUCCUUG.AAA.............CUGUCUUAGAU....................................................................................................
Ga0307484_111921/444-550      UGUCUGUCCUUG.AAA.............CUGUCUUAGAU....................................................................................................

                              <<<<....<<<<.<<<<.<<.........<<<<<..........................................................................................................
                              2222....2222.22..............................................................................................................................
                              •••C•GUCC•••-∘∘-------------------∘∘∘-------------------------------------------------------------------------------------------------------
```

```
Ga0184587_113157/56-168        GCGCUGUCCCUC.UGUA.GA........U.........................................................................................................................
Ga0255228_1272153/272-192      AAGCUGUCCAUC......................AUUU................................................................................................................
Ga0153880_1325448/483-575      CGUCGGUCUCAC......................AUGG................................................................................................................
Ga0157565_1130498/91-183       CGUCGGUCUCAC......................AUGG................................................................................................................
Ga0265758_129208/41-120        AGUCGGUCCGAA.A....................AG..................................................................................................................
Ga0265756_146329/172-94        AGUCGGUCCGAA.AU...................GA..................................................................................................................
Ga0115594_1180137/311-161      GCUCGGUUCCGC.GA......CU......GCACUGGAA................................................................................................................
Ga0265744_100861/398-567       UGUCUGUCUCCA.AUUC.G..........GAAUUGACCA...............................................................................................................
Ga0180107_1285918/13-113       UAGCGGUCCGGG.UAGG.GG.UC...UCG.........................................................................................................................
Ga0247519_103291/1381-1460     CUGCGGUCGGGA.U.......GU...............................................................................................................................
Ga0184603_106561/141-62        UUGCGGUCGGAA.U....................GUUGA...............................................................................................................
Ga0247551_100996/477-556       CUGCGGUCGGGA.U....................GUUGACU.............................................................................................................
Ga0184590_126350/300-380       CUGCGGUCGGGA.UG...................UU..................................................................................................................
Ga0265756_131580/159-58        CUCCUGUCCCAG.AACU.A...................................................................................................................................
Ga0307482_1000325/24-131       GAUCGGUCCUCU.GUC.....GC......UGUCUUCC.................................................................................................................
Ga0242646_1001086/737-571      AAUCUGUCCACG.UUUG.C..........GGAUUCCUGA...............................................................................................................
Ga0265747_132694/181-66        CUUCUGUCUCCC......................AG..................................................................................................................
Ga0157593_1023702/238-314      AGUCUGUCCCAG......................GCA.................................................................................................................
Ga0316053_103654/814-736       AGUCUGUCCCAG......................GCA.................................................................................................................
Ga0247529_117158/253-331       AGUCUGUCCCAG..........................................................................................................................................
Ga0180107_1129325/244-60       GUUCUGUCCCUU.GUGG............AGUCUGCUCA...............................................................................................................
Ga0307482_1162833/232-148      GGGCGGUCCGCA.G........................................................................................................................................
Ga0255267_1268184/91-184       AGUCUGUCCCCU.CCGC.C...................................................................................................................................
Ga0307919_1064641/225-73       CAUUUGUCCGCU.CCUU.AA.A.......CGG.CCUUGC...............................................................................................................
Ga0307921_1075885/188-36       CAUUUGUCCGCU.CCUU.AA.A.......CGG.CCUUGC...............................................................................................................
Ga0265742_1021317/385-459      CGUCAGUCCUGG.ACA..................GAAU................................................................................................................
Ga0307483_1002304/791-713      GUGCGGUCCAUG.U....................CGAGUCAAC...........................................................................................................
Ga0307919_1165008/203-116      AGUUUGUCCCCC......................GUA.................................................................................................................
Ga0210323_1093898/306-192      GUUCGGUACUCA.G....................CUA.................................................................................................................
Ga0265743_119695/104-184       GCUCGGUCCGUG.CAC..................GC..................................................................................................................
Ga0310118_104706/1022-1118     GUACGGUCAG........................GACUGAA.............................................................................................................
Ga0307921_1005950/551-460      UGUCUGUCCAGG.G.......AU...........UC..................................................................................................................
Ga0242641_1014724/551-384      GUUCUGUCUCAU.CUUC.C..........GAGGUCUAAA...............................................................................................................
Ga0242641_1002366/499-331      GUUCUGUCUCAU.CUUC.C..........GAGGUCUAAA...............................................................................................................
Ga0242657_1007384/290-215      UCGCUGUCCCAC.U........................................................................................................................................
Ga0265757_113513/277-362       GUUCUGUCUCCC.ACUA.................AA..................................................................................................................
Ga0242646_1001007/790-609      UCACUGUCCCCA.CUUC.G..........GAAUAGCUCG...............................................................................................................
Ga0316034_107719/314-205       GUUCGGUCCUCA.U.......AU...UCGUGCGCGGUUA...............................................................................................................
Ga0316039_119781/347-457       UCUCAGUCCCUA.U.......GA...UCGUGUGCGUUUA...............................................................................................................
Ga0255295_1078742/236-408      CAGCGGUCCAGG.AA.......................................................................................................................................
Ga0184603_150346/629-518       GUGUUGUCCUGA.A.......GU...CCGUGCGUGUGUUA..............................................................................................................
Ga0180106_1187148/166-89       CGACGGUCCACC.AGUA.....................................................................................................................................
Ga0307482_1417159/374-296      AGACGGUCCU.A.U....................CAUUUCA.............................................................................................................
Ga0222728_1000095/4027-4133    UUUCUGUCCCUA.ACUC....G............CGCACGUCCGUGCAAUCC..................................................................................................
Ga0316030_105840/623-504       AAGCUGUCCCUA.AA.......................................................................................................................................
Ga0316033_110175/528-409       AAGCUGUCCCUA.AAGG.GG.GG...GUU.........................................................................................................................
Ga0326631_124620/105-175       AAGCUGUCCCU...........................................................................................................................................
Ga0206356_11590518/224-68      UGACGGUCGCGC.G........................................................................................................................................
Ga0213850_1246156/171-47       CCUCUGUCCCUG.ACA......................................................................................................................................
Ga0184599_140450/516-588       UUGCAGUCGGAU.UGA......................................................................................................................................
Ga0206356_11002907/329-148     UGUCUGUCAUAG.G.CU.GG.A................................................................................................................................
Ga0307482_1001469/2898-2828    AUGCGGUCCACC.C....................UC..................................................................................................................
Ga0157570_1089798/68-159       GUGUGGUCCCCC.CUC..................GAUGGA..............................................................................................................
Ga0127503_11028958/51-130      CGUCUGUCAUUU.UGUC.AU..................................................................................................................................
Ga0157560_1126722/83-169       UUGCAGUGUCUU.AAGU.UU..................................................................................................................................
Ga0138277_1028338/433-516      CUUCUGUCCGA.......................AGAACUAU............................................................................................................
Ga0265742_1001214/55-127       GGUUUGUCCUG.......................AUG.................................................................................................................
Ga0115594_1134035/652-542      GGUCGGUACUGU.GUUC....UU...AA..........................................................................................................................
Ga0115594_1212468/8-118        GGUCGGUACUGU.GUUC....UU...AA..........................................................................................................................
Ga0247533_100438/1040-888      UGUCUGUCCCGG.ACUC....AU...CAU.....GCACUAGGCAAGUGACAGGUAGCAACGCUGACACAGGGUGUUAGAACCUAGUUACAAAAGG.......................................................
Ga0326631_117973/181-293       CCUCGGUCCU...........GA...GUA.........................................................................................................................
Ga0184600_117644/32-108        UAGUGGUCCAGA.CC...................AAUG................................................................................................................
Ga0184584_133603/317-393       UAGUGGUCCAGA.CC...................AGU.................................................................................................................
Ga0316052_100527/264-188       UAGUGGUCCAGA.CC...................AGU.................................................................................................................
Ga0184580_125947/153-229       UAGUGGUCCAGA.CC...................AGU.................................................................................................................
Ga0247524_105844/561-637       UAGUGGUCCAGA.C.......CA...............................................................................................................................
Ga0184580_109821/480-551       UAGCGGUCCAG.......................AUCAUCU.............................................................................................................
Ga0184598_107135/96-25         UAGCGGUCCAG.......................AUCA................................................................................................................
Ga0316051_1015080/488-559      UAGCGGUCC.........................AGGUCA..............................................................................................................
Ga0247524_121352/374-303       UAGCGGUCCGGA.UC.......................................................................................................................................
Ga0179934_1197833/207-115      AGUCGGUCCACA.G.......AA...UCU.........................................................................................................................
Ga0316049_129960/371-262       CGUCUGUCCCUU.GGGU.................UGCAA...............................................................................................................
Ga0265747_125301/405-306       CGUCAGUCCUUU.C....UG..............GUAG................................................................................................................
Ga0157570_1034241/151-229      UGUCGGUCCUGC.G....................AUGUG...............................................................................................................
Ga0157571_1064089/212-134      UGUCGGUCCUGC.G....................AUGUGU..............................................................................................................
Ga0242652_1041630/308-377      UAACGGUCCAUU......................GC..................................................................................................................
Ga0307480_1006257/684-599      CGGCGGUCCACC.CA.......................................................................................................................................
Ga0310119_142698/346-430       CGUUGGUCCCAG......................AGGA................................................................................................................
Ga0307482_1074296/478-354      CCACUGUCUCGA.G.......CU...GCGUCAUUCAUU................................................................................................................
Ga0307483_1005049/26-150       CCACUGUCUCGA.G.......CC...GCGUCAUUCAUU................................................................................................................
Ga0265757_127696/205-308       CUCCUGUCCAAG.GAAC.CG.A............AUC.................................................................................................................
Ga0316029_101202/440-551       UAUUUGUCUCUG.GGG.....AA...G..CGUG.....................................................................................................................
Ga0307482_1134687/287-181      AUGCGGUCCCAG.GU...................AACAGU..............................................................................................................
Ga0307484_104189/399-292       AUGCGGUCCCAG.GU.......A...........AACAG...............................................................................................................
Ga0307921_1050271/251-412      CAUUUGUCCGCU.CCUC............AAACUACAUAA..............................................................................................................
Ga0184580_102899/442-582       CCUCGGUCCUUG.AUGG.AA.GC...........CGCCCCAUUGUCAGGGGGAU................................................................................................
Ga0316029_136733/88-8          AUGUUGUCCC........................GAGGCUCAC...........................................................................................................
Ga0180107_1174136/218-102      UAGCGGUCCUAC.GAGA....UU...AAG.........................................................................................................................
Ga0179948_1254202/198-296      AGUCGGUCCACA.C........................................................................................................................................

                               <<<<....<<<<.<<<<.<<.........<<<<<....................................................................................................................
                               2222....2222.22.......................................................................................................................................
                               ●●●C●GUCC●●●-●○-------------------○○○-----------------------------------------------------------------------------------------------------------------
```

```
Ga0265746_1140777/214-319       CUCCUGUCCAAG.GAGC.GU.A............AUCUA................................................................................................................
Ga0265756_108694/382-277        CUCCUGUCCAAG.GAGC.GU.A............AUCUA................................................................................................................
Ga0265758_129717/201-94         CUCCUGUCCAAG.GA........................................................................................................................................
Ga0213850_1394829/253-105       AGUCUGUCCUUC.AG......UU......GUAUUAUAUAC...............................................................................................................
Ga0184603_139562/141-280        GAUCUGUCCCUU.GG...................AGGAA................................................................................................................
Ga0157560_1100546/299-385       GGACGGUCCCAC.AU...................AAA..................................................................................................................
Ga0129331_1494381/292-368       CGUCGGUCC.........................GGAGCCC..............................................................................................................
Ga0265759_100473/818-988        CUUCUGUCUCAG.UCAA............GGGUUCCCA.................................................................................................................
Ga0265746_1003835/1049-1219     CUUCUGUCUCAG.UCAA............GGGUUCCCA.................................................................................................................
Ga0265756_100592/1458-1288      CUUCUGUCUCAG.UCAA............GGGUUCCCA.................................................................................................................
Ga0247536_100773/637-807        CUUCUGUCUCAG.UCAA............GGGUUCCCA.................................................................................................................
Ga0184578_101068/480-310        CUUCUGUCUCAG.UCAA............GGGUUCCCA.................................................................................................................
Ga0247530_109035/489-319        CUUCUGUCUCAG.UCAA............GGGUUCCCA.................................................................................................................
Ga0247513_128543/241-71         CUUCUGUCUCGG.UCAA............GGGUUCCCA.................................................................................................................
Ga0184573_112336/323-153        CUUCUGUCUUGG.UCAA............GGGUUCCCA.................................................................................................................
Ga0127502_10575752/515-449      CUGCGGUCCCCU.U.........................................................................................................................................
Ga0180107_1033011/786-679       GGCUUGUCCCAC.UAC.............GGUUCACUCGU...............................................................................................................
Ga0222728_1380294/134-238       GAGCGGUCCUGG.G.......AC...........UACAG................................................................................................................
Ga0307484_107311/306-374        AAGCGGUCCCCU......................GGUUC................................................................................................................
Ga0316046_107255/463-562        ACGCUGUCCCCU.UUCU.................GAUACCUGAAAG.........................................................................................................
Ga0184570_114412/209-338        GGUUGGUCCUUC.AAUC.AG.A.................................................................................................................................
Ga0242646_1000880/695-856       GUUCUGUCCCUA.CUCC.GG.........GGGUUGCCA.................................................................................................................
Ga0316031_123456/415-346        CGUCUGUCCGG.......................AGG..................................................................................................................
Ga0157630_1203478/203-99        UGUCUGUCCGGU......................AGC..................................................................................................................
Ga0247541_102707/328-184        CCUCUGUCCUCU.GAAG.U..UG...AUCUAGUUCGUGA................................................................................................................
Ga0115594_1174394/642-729       UGUCUGUCCUAC.C.......AA...........UAA..................................................................................................................
Ga0115594_1187657/870-960       UGUCUGUCCUAC.C.......AA...........UAAGUA...............................................................................................................
Ga0242646_1047905/92-182        GGUCUGUCUCUC.U.......GU...AUA.....UCAGCCA..............................................................................................................
Ga0247541_101130/558-391        CUUCUGUCUCCU.CCUG............GGGCUCCCA.................................................................................................................
Ga0307921_1062541/180-338       CAUUUGUCCGCU.CCCC.AU.AA...CAAAGCGUCAAA.................................................................................................................
Ga0184572_105168/388-281        UUUCGGUCCUCC.A.......GU...UCGUGCGUAUG..................................................................................................................
Ga0316036_127178/124-231        UUUCGGUCCUCC.A.......GU...UCGUGCGUAUG..................................................................................................................
JGI12272J11983_1345183/364-258  UUUCUGUCCCAC.AGUC.C.........GUGC..GUAAA................................................................................................................
Ga0247535_100068/719-828        GUUCGGUCCUCC.A.......GU...UCGUGCGCGGAUA................................................................................................................
Ga0222728_1000079/1192-1299     CUUUUGUCCUCC.AGUC.C.........GUGC..GUUGUU...............................................................................................................
Ga0081246_1201717/153-259       UUUCUGUCCCAC.AGUC..........CGUGC..GUAAA................................................................................................................
Ga0222728_1216887/51-157        CUUCGGUCCUCC.AGUC..........CGUGC..GUAAA................................................................................................................
Ga0157569_1073490/184-258       GGACUGUCCCAG.AGUU......................................................................................................................................
Ga0180047_1061805/548-622       GGACUGUCCCAG.AGUU......................................................................................................................................
Ga0206353_11976089/292-380      GUUCGGUCCGCC.A....................ACGGU................................................................................................................
Ga0247553_101094/709-629        GUACGGUCCACG.U....................GGAUUCAAUGA.........................................................................................................
Ga0242641_1004288/32-112        GUACGGUCCGCG.U....................CGAGUCAA.............................................................................................................
Ga0242641_1014474/336-257       CCACGGUCCCAU.C.........................................................................................................................................
Ga0126318_10199518/109-204      UAGCGGUCGCGC.GCUA......................................................................................................................................
Ga0307919_1109364/247-158       UGUCGGUCCUCU.CUC..................AUUA.................................................................................................................
Ga0180107_1307618/257-174       CGUCUGUCCGCU.CUU..................GCU..................................................................................................................
Ga0179937_1126086/216-325       GGUCGGUCCGUA.GCGC.G...............AGC..................................................................................................................
Ga0265744_134092/134-214        UUGCAGUCCCAG.ACU.......................................................................................................................................
Ga0307482_1015399/786-719       UUGCAGUCCCAU.C....................GA...................................................................................................................
Ga0265751_100477/874-675        UCGCUGUCCCAG.UAGG....AG...UAA.....GCCCGA...............................................................................................................
Ga0265756_100433/485-286        UCGCUGUCCCAG.UAGG....AG...UAA.....GCCCGA...............................................................................................................
Ga0265743_100639/33-191         UCGCUGUCCCAG.UAGG....AG...UAA.....GCCCGA...............................................................................................................
Ga0184602_130623/1321-1100      AUUCUGUCCCCG.UUUCCGG.........UGCCUAAAAGCAACCGC.........................................................................................................
Ga0184597_126105/585-362        AUUCUGUCCCCG.UUUCCGG.........UGCCUAAAAGC...............................................................................................................
Ga0187793_1322799/72-151        CGUCGGUCCUGA.GCU.......................................................................................................................................
Ga0316037_128286/253-347        GGUUUGUCCCUC.AGAG.C...............ACUCAA...............................................................................................................
Ga0265758_109576/83-157         AGGCUGUCCCAC.CAU..................GCCUUG...............................................................................................................
Ga0265751_100127/176-103        AUACGGUCCUGA......................UGUUUAA..............................................................................................................
Ga0265758_108618/113-22         GGAUGGUCCGGU.GCUC.................AGC..................................................................................................................
Ga0228708_1128185/228-344       UGACGGUCUCUC.GUUU....UU...AAU..........................................................................................................................
Ga0256309_1199458/363-444       CAACGGUCCAUU.C....................AU...................................................................................................................
Ga0256302_1090383/76-157        CAACGGUCCAUU.C....................AUG..................................................................................................................
Ga0255224_1021381/13-113        CAACGGUCCAUU.C....................AUG..................................................................................................................
Ga0255223_1087001/28-107        CAACGGUCCAUU.C....................AU...................................................................................................................
Ga0127502_11371108/362-252      UCUUUGUCCUAC.A.......GU...CCGUCCUUGUCCA................................................................................................................
Ga0222728_1000125/1315-1238     UCGCAGUCCUCU.AC...................CAUCACA..............................................................................................................
Ga0242654_10000586/1273-1196    UCGCAGUCCUCA.UG...................CACAUAU..............................................................................................................
Ga0180107_1218358/314-219       UUACGGUCCCAU.UC...................GGAGUCUAAGCAGA.......................................................................................................
Ga0307482_1020425/440-614       GAUCUGUCCCUA.GUUU.GG.........GUAUUUGUCA................................................................................................................
Ga0179951_1211246/478-633       CGUCGGUCCAUA.ACUU.................AGAAUUAGG............................................................................................................
Ga0179950_1197742/286-441       CGUCGGUCCAUA.ACUU.................AGAAGUAGG............................................................................................................
Ga0129331_1058147/189-99        GUUCUGUCCCCA.U.......A............UAAG.................................................................................................................
Ga0265756_102054/286-395        GUUCGGUCCCAG.A.......AU...CCGUGUACGUUGA................................................................................................................
Ga0222728_1000128/4501-4432     CUGUCGUCCCAU.CUA.......................................................................................................................................
Ga0265759_114856/370-262        CGUCUGUCCUAA.G.......A............AUCGAA...............................................................................................................
Ga0079039_1551950/237-315       AGUUUGUCCAGC.GC...................GCUUUC...............................................................................................................
Ga0179943_1170285/153-222       AGUUUGUCCAAA.C.........................................................................................................................................
Ga0213850_1119088/23-236        AGUCGGUCCUUA.CACA.UA.GC...........GUGGACAAUGAAAUCAACACCGCUCACCUGCUGGCAUCACCUUCUAGCUUGAUAGCACUGGUA......................................................
Ga0247533_103075/417-305        CCUCGGUCCCGU.CGG...G.AA...CUC..........................................................................................................................
Ga0247541_102964/56-168         CCUCGGUCCCGU.CGGG.................AA...................................................................................................................
Ga0265755_105988/310-386        CGUCAGUCUCUU.CG........................................................................................................................................
Ga0157560_1026167/317-382       UGUCGGUCUGUG......................CG...................................................................................................................
Ga0115595_1093158/7-137         AGGCGGUCUUCA.CUC.......................................................................................................................................
Ga0256352_1101982/155-277       UGUCUGUCCCUC.UCG..............AG..AGCAAUCUCCG..........................................................................................................
Ga0256350_1100736/283-405       UGUCUGUCCCUC.UCG..............AG..AGCAAUCUCCG..........................................................................................................
Ga0213850_1360620/298-221       CAGCGGUCCGUC......................GU...................................................................................................................
Ga0081247_1167831/113-35        AGUCUGUCCUC.......................AACC.................................................................................................................
Ga0127503_10676578/136-67       CGUCGGUCCCCC......................AG...................................................................................................................
Ga0138283_1149501/173-59        CGACGGUCCCCC.CC........................................................................................................................................

                                <<<<....<<<<.<<<<.<<.........<<<<<.....................................................................................................................
                                2222....2222.22........................................................................................................................................
                                ●●●C●GUCC●●●-○○-------------------○○○------------------------------------------------------------------------------------------------------------------
```

```
Ga0265757_139522/177-70         UAUUUGUCCCAG.AGUC..........CGUGUG..UUUG................................................................................................................
Ga0265750_1013321/389-496       UAUUUGUCCCAG.AGUC..........CGUGUG..UUUG................................................................................................................
Ga0316029_116217/368-261        AGUUGGUCCCUC.AGUC..........CGUGUG..UUUG................................................................................................................
Ga0316047_100016/2681-2574      AGUUGGUCCCUC.AGUC..........CGUGUG..UUUG................................................................................................................
Ga0184569_112764/943-836        AGUUGGUCCCUC.AGUC..........CGUGUG..UUUG................................................................................................................
Ga0242652_1131915/134-203       GGUCGGUCCCAC.U....................CAAG.................................................................................................................
Ga0307482_1020622/600-404       CUUCUGUCCCCU.GUUC............AGGUUACCUAA...............................................................................................................
Ga0265756_143657/133-55         UGUUGGUCCG........................AAGGCCG..............................................................................................................
Ga0180107_1262812/3136-3066     GGUCGGUCCUGG......................GUGA.................................................................................................................
Ga0265756_118952/425-318        GGGCUGUCCCAG.U.......UU...GGUGAUUCAUUAUU...............................................................................................................
Ga0265757_113703/332-416        UUUCUGUCCGCA......................AA...................................................................................................................
Ga0256332_1243244/48-162        CGGCGGUCCAGU.AG...................ACAUG................................................................................................................
Ga0307919_1048575/242-90        CAUUUGUCCGCU.CCUC.AA.A.......CACGCGAGAU................................................................................................................
Ga0265754_1081367/214-290       CGUCGGUCCACC......................GCG..................................................................................................................
Ga0184597_114231/79-11          CUGCGGUCCAG.......................CU...................................................................................................................
Ga0184582_129853/106-35         CUGCGGUCCAGU.G....................GA...................................................................................................................
Ga0265744_136679/344-276        CUGCGGUCCGAC.U.........................................................................................................................................
Ga0326631_172366/284-207        CUUCUGUCCAUG.U....................UACGA................................................................................................................
Ga0307921_1021783/160-261       AUUCUGUCCCGU.AC......................AA................................................................................................................
Ga0247554_113506/254-161        UAACGGUCCCAU.AUG..................UCCAAU...............................................................................................................
Ga0179939_1229388/322-392       AGUCUGUCCGCU.G....................UG...................................................................................................................
Ga0184568_100511/100-195        AGUCUGUCCGCU.GAG..................AUGAAGCCUAG..........................................................................................................
Ga0247525_114510/199-318        UGUCUGUCCCGA.AC......UA......CAU..CAGGGU...............................................................................................................
Ga0222728_1008526/24-283        GGUCUGUCCUUA.UCCU.AU..............ACUCCCUCACCCCGAACAGAAAGAGGGGAGCUGCCCACCCAUUAAAGUGGAACAGACACAUUCGUGCUACGUGCUU........................................
Ga0242654_10278372/461-362      AUGCAGUACUGG.A...............UUUCUCCAAA................................................................................................................
Ga0184570_100371/69-155         AGUCAGUCCCGC.G....................GCACAGAC.............................................................................................................
Ga0184597_120210/233-147        AGUCGGUCCCGC.G.........................................................................................................................................
Ga0213850_1220553/385-463       GGACGGUCCCAC.CAU..................AAGU.................................................................................................................
Ga0265745_1120044/54-129        GCCCUGUCCCAC.U....................GUA..................................................................................................................
Ga0213850_1278242/600-506       CGUCGGUCUCGG.A....................GCCCGCGAC............................................................................................................
Ga0213850_1524759/286-380       CGUCGGUCUCGG.A....................GCCCGCGGA............................................................................................................
Ga0242657_1009512/1576-1466     CCUCGGUCCCAC.A.......GU...CCGUGCGCGUUUA................................................................................................................
Ga0307482_1430195/259-189       GAGCGGUCCAUC......................UA...................................................................................................................
Ga0184590_129043/450-518        GUGCGGUCCAUC.U.........................................................................................................................................
Ga0184578_132646/9-94           GGUCUGUCCCCA.A.........................................................................................................................................
Ga0180107_1040049/1100-1010     UUUCUGUCCGG.......................CGUCGCAUUA...........................................................................................................
Ga0307484_103912/620-553        UGGCAGUCCGC.......................CUUU.................................................................................................................
Ga0307921_1037919/342-457       AUUCUGUCCCAG.GAUA.................CGGCUU...............................................................................................................
Ga0307920_113994/654-539        AUUCUGUCCCAG.GAUA.................C....................................................................................................................
Ga0316040_172648/178-66         CAUCUGUCCCAG.GAUA.C....................................................................................................................................
Ga0184577_110850/246-358        CAUCUGUCCCAG.GAUA.C....................................................................................................................................
Ga0307920_113994/130-9          AUUCUGUCCCAG.GAUA............CGGCGGGUUGACG.............................................................................................................
Ga0307919_1081825/191-74        AUUCUGUCCCAA.GAUA.................CCUCGGCCUAGG.........................................................................................................
Ga0307921_1095896/178-61        AUUCUGUCCCAA.GAUA.................CCUCGGCCUAGG.........................................................................................................
Ga0316031_113250/505-406        ACUCGGUCCGCU......................GA...................................................................................................................
Ga0265744_118928/137-218        GUGCUGUCCCUA.C....................AACGGA...............................................................................................................
Ga0265749_106268/131-212        GUGCUGUCCCAA.C....................AACGGAGG.............................................................................................................
Ga0157562_1160399/232-315       GGUCGGUCCCAU.AGAU.U...............GAU..................................................................................................................
Ga0153880_1231581/116-241       CAGCUGUCCCAA.U.........................................................................................................................................
Ga0153880_1191383/455-340       GAUCUGUCCUAU.UUC.......................................................................................................................................
Ga0138282_1242808/325-475       GAUCGGUCCGGA.GGCG.CG.CU...GCA..........................................................................................................................
Ga0242654_10024002/836-915      ACUCUGUCUCAA.G....................CAGGU................................................................................................................
Ga0242646_1103331/248-318       GGGCAGUCCUUC.UU........................................................................................................................................
Ga0081243_1208438/380-456       CGUCUGUCCACC......................AUCC.................................................................................................................
Ga0184600_147964/253-339        AGUCGGUCCCAU.CGUA.C....................................................................................................................................
Ga0316036_107710/408-322        AGUCGGUCCCAU.CGUA.C....................................................................................................................................
Ga0184601_135946/406-321        UGUCGGUCCCAU.CGUA.C....................................................................................................................................
Ga0184589_121067/502-587        UGUCGGUCCCAU.CGUA.C....................................................................................................................................
Ga0184598_138497/440-355        UGUCGGUCCCAU.CGUA.C....................................................................................................................................
Ga0184580_113725/262-335        UGUCGGUCCC........................GA...................................................................................................................
Ga0184584_115572/267-354        GGUCGGUCCCCU.UAUC.AU.G.................................................................................................................................
Ga0247539_100086/1763-1850      GGUCGGUCCCCU.UAUC.AU.G.................................................................................................................................
Ga0184579_105257/1735-1648      GGUCGGUCCCCU.UAUC.AU.G.................................................................................................................................
Ga0126318_10229384/609-688      CUGCGGUCCAGC.AACU.U...............UCAA.................................................................................................................
Ga0307482_1000291/1027-950      CAGCGGUCCU........................UA...................................................................................................................
Ga0316031_119808/254-182        UCUCAGUCCCCG.UC........................................................................................................................................
Ga0307482_1023665/853-778       CGUCUGUCCCAC.AGU..................GG...................................................................................................................
Ga0180106_1297567/444-515       CAGCGGUCCCUA.GGAC.................CU...................................................................................................................
Ga0307484_115660/213-145        GAGCGGUCCCUA.CA...................CUA..................................................................................................................
Ga0307484_161007/136-39         UAGCGGUCCAUG.G.......AG......AGGGGUAAAC................................................................................................................
Ga0307484_177982/136-39         UGUCGGUCCAUG.........AA...G..AGGGGUAAAC................................................................................................................
Ga0307482_1033688/516-422       UAGCGGUCCAUG.A...............GGGGGGUUAA................................................................................................................
Ga0265752_111922/418-343        UUUCUGUCCGCG.UUU..................GUG..................................................................................................................
Ga0179939_1199779/67-205        GGUCGGUCCCCG.AUUG......................................................................................................................................
Ga0307482_1023050/176-255       UGACGGUCCAC.......................CUA..................................................................................................................
Ga0307482_1202605/470-395       CGUCGGUCCCCU.U.G..................CUG..................................................................................................................
Ga0242657_1476020/160-244       GAUCUGUCCCAG.UC...................AU...................................................................................................................
Ga0247523_120120/325-403        UAGCGGUCCGCA.U.........................................................................................................................................
Ga0316035_179807/270-184        CAGCGGUCCUCG.CCGU.GA...................................................................................................................................
Ga0210323_1050507/418-532       GUUCGGUCCUCA.G....................CUA..................................................................................................................
Ga0184571_112742/47-116         GUGCAGUCCCUA.CA...................UUC..................................................................................................................
Ga0247538_100770/928-997        GUGCAGUCCCUA.CA...................UUCG.................................................................................................................
Ga0157560_1032840/250-177       AAGCGGUCCGGA.U....................GGCGU................................................................................................................
Ga0247514_107497/1071-1140      GAGCAGUCCCUC...........................................................................................................................................
Ga0242646_1002014/11-270        GGUCUGUCCUUA.UCCU.AU..............ACUCCCUCACCCCGAACAGAAAGAGGGGAGCUGCCCACCCAUUAAAGUGGAACAGACACAUUCGUGC..................................................
Ga0222728_1008391/1281-1023     GGUCUGUCCUUA.UCCU.AU..............ACUCCCUCACCCCGAACAGAAAGAGGGGAGCUGCCCACCCAUUAAAGUGGAACAGACACAUUCGUGCUACGUGCUUGACUACGAGCGCAAGCAAGCAUCCUUCCACAUAAAAACCA
Ga0242646_1001395/255-509       CGUCUGUCCCCA.CCAU.CU.G............UGCUACCGAUGUUAACGUUAAGGUAGACACACGCACCGCCCAACCCGACCUCAAGGGAAUUCGUAGAGGACGCAUUUUG.....................................
Ga0307482_1000256/3341-3276     CGGUUGUCCGC............................................................................................................................................
Ga0242647_1006852/526-598       UAGCGGUCCCCA.G....................UGUAACA..............................................................................................................

                                <<<<....<<<<.<<<<.<<.........<<<<<.....................................................................................................................
                                2222....2222.22
                                ●●●C●GUCC●●●-○○-------------------○○○----------------------------------------------------------------------------------------------------------------------
```

```
Ga0184580_111164/678-749       CAGCGGUCCCU.C.....................CUU.................................................................................................................
Ga0184600_102725/60-135        UCGCGGUCCCCA.ACA..................CUU.................................................................................................................
Ga0184602_122974/190-265       UCGCGGUCCCCA.AC...................ACU.................................................................................................................
Ga0256338_1343513/152-228      CCUCGGUCCUCA.CU...................UA..................................................................................................................
Ga0255268_1432700/127-51       CCUCGGUCCUCA.CU...................UG..................................................................................................................
Ga0157560_1147189/148-60       GUACGGUCCAAG.CGCG.G...................................................................................................................................
Ga0242654_10672177/244-165     AUGCGGUCCCAC.U.......A................................................................................................................................
Ga0242654_10135580/213-336     CAGUAGUCCAGC.AA......U............GACU................................................................................................................
Ga0265742_1000025/2819-2888    CAGCGGUCCGCU.C....................AUC.................................................................................................................
Ga0184599_154888/232-155       CUGCGGUCCGG.......................AUGGGUGAC...........................................................................................................
Ga0228707_1100139/344-194      UCACUGUCUCAU.C.......UU...UAG.....AG..................................................................................................................
Ga0307919_1084008/308-378      AUUCUGUCCCG.......................UAC.................................................................................................................
Ga0307919_1039507/513-440      UUUCUGUCCCGU.A....................CUA.................................................................................................................
Ga0307921_1024041/406-334      UUUCUGUCCCGU.A....................CUA.................................................................................................................
Ga0242657_1015112/947-881      GAGUGGUCCCU...........................................................................................................................................
Ga0307482_1021658/484-563      AAGCGGUCC.UA.CACU.....................................................................................................................................
Ga0307920_133961/233-140       UAUCUGUCCCGG.AC...................ACCUGCUG............................................................................................................
Ga0307921_1019702/90-184       UAUCUGUCCCGG.AC...................AAACUGCUGGAGAGC.....................................................................................................
Ga0182092_1031527/199-109      GGUCGGUCCUGA.C....................AUGG................................................................................................................
Ga0184575_104772/255-338       CAGCGGUCCUCG.C....................AUUGGUU.............................................................................................................
Ga0179940_1033563/296-410      CGCCUGUCCUCG.AACA.UC.CU...UUCGGCGUAGUAAU..............................................................................................................
Ga0184596_102602/271-200       UUGCGGUCAGUG.AUC......................................................................................................................................
Ga0184596_125844/4802-4873     UUGCGGUCAGUG.AUC......................................................................................................................................
Ga0153880_1058095/344-241      GUUCUGUCCUCU.A.......UG...............................................................................................................................
Ga0153880_1167367/224-121      GUUCUGUCCUCU.A.......UG...............................................................................................................................
Ga0179943_1178349/195-298      GUUCGGUCCUCG.A....................UGA.................................................................................................................
Ga0179944_1313268/129-232      GUUCGGUCCUCG.A.......UG...A...........................................................................................................................
Ga0213850_1259428/445-342      GUUCUGUCCUCU.A.......UG...............................................................................................................................
Ga0115595_1051440/308-419      UGACGGUCCUUU.UAC..................AUAUGACGCA..........................................................................................................
Ga0310106_100998/1608-1690     AUGCGGUCCUAG.AAG..................UGA.................................................................................................................
Ga0307482_1017422/1387-1206    UGUCUGUCCCUA.GUUC.GG.........GAAUUAACCA...............................................................................................................
Ga0307482_1050048/176-95       AUGUGGUCCGGG.GCCC.CC.UU...............................................................................................................................
Ga0179939_1078463/91-178       CGUCUGUCCACC......................AU..................................................................................................................
Ga0247539_107237/153-229       CUACGGUCCCUA.AUAU.CA..................................................................................................................................
Ga0127502_10189886/163-55      UGUCGGUCCGUU.CUC..................AA..................................................................................................................
Ga0265746_1085300/256-329      CACCGGUCCCCU.CUC..................GU..................................................................................................................
Ga0242641_1002064/1203-1116    AUGCUGUCCAGC.UUCU.CA..................................................................................................................................
Ga0242657_1090937/459-526      GUGCAGUCCCCG.G....................AU..................................................................................................................
Ga0180107_1021361/468-373      GUUCGGUCCCAC.AC..............GGA......................................................................................................................
Ga0184599_136526/36-116        AUGCGGUCCGGG.AG.......................................................................................................................................
Ga0316032_127868/210-130       AUGCGGUCCGGG.AG.......................................................................................................................................
Ga0032354_1062690/2659-2586    AUGCGGUCC.CC.CUGG.AC..................................................................................................................................
Ga0242657_1027062/255-322      AUGCGGUCCCCG.C........................................................................................................................................
Ga0265747_105373/294-105       UCUCUGUCCCUG.UUAC............GGACUCCUGA...............................................................................................................
Ga0265745_1000512/1043-854     UCUCUGUCCCUG.UUAC............GGACUCCUGA...............................................................................................................
Ga0265745_1001084/890-701      UCUCUGUCCCUG.UUAC............GGACUCCUGA...............................................................................................................
Ga0310117_104105/1058-869      UCUCUGUCCCUG.UUAC............GGACUCCUGA...............................................................................................................
Ga0265745_1000697/596-785      UCUCUGUCCCUG.UUAC............GGACUCCUGA...............................................................................................................
Ga0247551_106718/112-179       GUGCGGUCCUGU......................AA..................................................................................................................
Ga0184603_137775/245-174       CUACGGUCCGG.......................CAUCU...............................................................................................................
Ga0242641_1105635/265-351      CGUCGGUCCAGU.GUC..................AU..................................................................................................................
Ga0242654_10795375/263-349     CGUCGGUCCAGU.GUC..................AU..................................................................................................................
Ga0157576_1168696/131-64       CGUCGGUCCAC.......................AUGGU...............................................................................................................
Ga0255292_1094640/555-357      CGUCUGUCCUCA.GA...................UCGCCACAA...........................................................................................................
Ga0256349_1073199/279-477      CGUCUGUCCUCA.GAUC.................GCCACAG.............................................................................................................
Ga0256358_1096062/218-22       CGUCUGUCCUCA.GAUC.................GCCCC...............................................................................................................
Ga0256350_1079106/235-433      CGUCUGUCCUCA.GAUC.G...............CCACAGCUAAG.........................................................................................................
Ga0256352_1080168/251-449      CGUCUGUCCUCA.GAUC.G...............CCACAGCUAAG.........................................................................................................
Ga0255293_1084149/567-369      CGUCUGUCCUCA.GAUC.G...............CCAC................................................................................................................
Ga0256348_1071395/353-551      CGUCUGUCCUCA.GAUC.....................................................................................................................................
Ga0265758_100178/821-753       GUGCGGUCCUC...........................................................................................................................................
Ga0157560_1090855/105-186      CGACGGUCCUGA.CA...................AU..................................................................................................................
Ga0005851_1006483/274-172      GGUCGGUCCUCG.A..................UCUCAGAA..............................................................................................................
Ga0184603_107262/477-596       UGUCUGUCCCGA.AC...................UACAU...............................................................................................................
Ga0184585_151549/495-376       UGUCUGUCCCGA.AC...................UACAU...............................................................................................................
Ga0242647_1022053/339-428      UUGCGGUCGGGA.C....................CUU.................................................................................................................
Ga0184599_128731/188-273       CGUCGGUCCCAU.CACC.U...............AC..................................................................................................................
Ga0310114_149639/68-153        CGUCGGUCCCAU.CACC.U...............AC..................................................................................................................
Ga0310119_131358/533-447       CGUCGGUCCCAU.CACC.U...............AC..................................................................................................................
Ga0079074_1318576/412-571      UGUUUGUCCCUU.CGG..................CACUGCUGCAAAACGCACAGCUGCCUGAGGCCCGCUGCCGGAAC........................................................................
Ga0307484_102285/276-209       GUGCAGUCAGGA......................CCAAA...............................................................................................................
Ga0247534_109145/204-136       GUGCAGUCGGG.......................CCC.................................................................................................................
Ga0242657_1013457/154-227      GAGUGGUCCCCA.AC...................AUG.................................................................................................................
Ga0242654_10000487/4104-4168   AGACGGUCUCCC.U........................................................................................................................................
Ga0242647_1038796/266-195      GAGCGGUCUACA.GG...................UGA.................................................................................................................
Ga0115594_1156252/268-370      UGUCUGUCCGUA.C.......AA...AUA.....AGGGCGUAUC..........................................................................................................
Ga0115594_1016490/326-410      UGUCUGUCCGCA.C....................ACCGACU.............................................................................................................
Ga0115595_1007417/328-412      UGUCUGUCCGCA.C....................GCCGACU.............................................................................................................
Ga0265742_1038020/174-90       CGACGGUCCCCC.AA...................CCAA................................................................................................................
Ga0184568_114501/165-82        CGGCGGUCCCC.......................ACAC................................................................................................................
Ga0265755_113253/366-449       CGGCGGUCCCC.......................ACAC................................................................................................................
Ga0184600_115185/364-447       CGGCGGUCCC........................CACACCAA............................................................................................................
Ga0157593_1013623/299-215      CGUUUGUCCC...........AA......GACUUCGAAAU..............................................................................................................
Ga0153880_1374410/101-33       AAUCUGUCCCA.......................UUUAC...............................................................................................................
Ga0316038_101969/827-896       CAGCGGUCCGCA.U........................................................................................................................................
Ga0316046_130251/204-135       CAGCGGUCCGCA.U........................................................................................................................................
Ga0265758_104499/242-367       CCUCGGUCUCCC.GGAA.GA.GC...CCACCUCUCGUGU...............................................................................................................
Ga0307484_108094/186-350       UGACUGUCCCCA.CUUC.G..........GGUUUGCUCA...............................................................................................................
Ga0307482_1009223/670-508      UGACUGUCCCCA.CUUC............GGGUUGCUCA...............................................................................................................

                               <<<<....<<<<.<<<<.<<.........<<<<<...................................................................................................................
                               2222....2222.22.......................................................................................................................................
                               ●●●C●GUCC●●●-∘∘-------------------∘∘∘-----------------------------------------------------------------------------------------------------------------
```

```
Ga0242647_1067563/210-289      GUGCAGUCCACA.CCAU.AU..................................................................................................................................
Ga0242657_1207391/462-383      GUGCGGUCCACA.CCAU.AU..............CA..................................................................................................................
Ga0265742_1001052/322-80       CAUCUGUCCCUG.UUAC............GGACUUUCGA...............................................................................................................
Ga0265750_1004028/1262-1504    CAUCUGUCCCUG.UUAC............GGACUUUCGA...............................................................................................................
Ga0310113_106868/972-730       CAUCUGUCCCUG.UUAC............GGACUUUCGA...............................................................................................................
Ga0265747_100653/495-253       CAUCCGUCCCUG.UUAC............GGACUUUCGA...............................................................................................................
Ga0265752_100504/930-688       CAUCUGUCCCUG.UUAC............GGACUUUCGA...............................................................................................................
Ga0265740_1001378/1084-842     CAUCUGUCCCUG.UUAC............GGACUUUCGA...............................................................................................................
Ga0265750_1009219/272-30       CAUCUGUCCCUG.UUAC............GGACUUUCGA...............................................................................................................
Ga0265757_104669/135-376       CAUCUGUCCCUG.UUAC............GGACUUCCA................................................................................................................
Ga0265745_1000618/793-552      CAUCUGUCCCUG.UUAC............GGACUUCGA................................................................................................................
Ga0265742_1088739/51-292       CAUCUGUCCCUG.UUAC............GGACUUCCA................................................................................................................
Ga0265747_100509/1277-1036     CAUCUGUCCCUG.UUAC............GGACUUCCA................................................................................................................
Ga0265752_100314/1466-1225     CAUCUGUCCCUG.UUAC............GGACCUCCA................................................................................................................
Ga0265743_104537/484-553       UAGCGGUCCGGG.C....................GCA.................................................................................................................
Ga0265744_160011/83-18         UAGUGGUCCCCC......................AU..................................................................................................................
Ga0153880_1410217/288-397      UAUUUGUCCUCC.CACA.GA.........AGUGCGGUUAU..............................................................................................................
Ga0115594_1054328/389-276      UGACGGUCCUUU.UAC.....U.............UAA................................................................................................................
Ga0316029_112968/271-337       UGUCGGUCCUC.......................AG..................................................................................................................
Ga0157560_1034839/242-321      GAUCGGUCCUCU......................GAA.................................................................................................................
Ga0157566_1117351/242-321      GAUCGGUCCUCU......................GAU.................................................................................................................
Ga0157593_1112297/86-12        GAACGGUCCAUU.C....................UGAU................................................................................................................
Ga0157565_1020724/148-72       GAACGGUCCAUU.CUA..................AU..................................................................................................................
Ga0247519_104852/800-730       GUGCGGUCCGG...........................................................................................................................................
Ga0307480_1000549/913-1064     GUACUGUCCCUG.CGGG.AG.A...............AAG..............................................................................................................
Ga0316031_132099/178-294       UCUCGGUCCCUU.GACC.....................................................................................................................................
Ga0247526_116548/279-354       AUGCGGUCGGAG.G........................................................................................................................................
Ga0247519_113845/334-409       AUGCGGUCGGGA.G........................................................................................................................................
Ga0247529_111070/303-228       AUGCGGUCGGGA.G........................................................................................................................................
Ga0184602_101759/1045-970      AUGCGGUCGGAG.GGUC.....................................................................................................................................
Ga0184600_139292/252-327       AUGCGGUCGGAG.GAUC.................CU..................................................................................................................
Ga0184580_108231/296-221       AUGCGGUCGGAG.GAUC.................CU..................................................................................................................
Ga0247534_105038/288-211       AUGCGGUCGGAG.GAUC.................CU..................................................................................................................
Ga0247552_109074/256-333       AUGCGGUCGGGG.GAUC.................CU..................................................................................................................
Ga0247542_100296/1022-1097     AUGCGGUCGGGG.GAUC.................CU..................................................................................................................
Ga0247545_113720/232-157       AUGCGGUCGGAG.GAUC.................CU..................................................................................................................
Ga0184597_110829/1090-1011     CAGCGGUCCGUG.G....................CUGUAGAAUAU.........................................................................................................
Ga0184600_105920/81-160        CAGCGGUCCG...........U................................................................................................................................
Ga0184578_129447/314-235       CAGCGGUCCGUG......................GCUGUAGAAU..........................................................................................................
Ga0184602_121948/324-245       CAGCGGUCCGUG.GCU......................................................................................................................................
Ga0184603_125382/9-88          CAGCGGUCCAGG..........................................................................................................................................
Ga0247550_100138/208-129       CAGCGGUCCAGG..........................................................................................................................................
Ga0316046_122965/221-142       CAGCGGUCCAGG.GCGU.................UGGAAU..............................................................................................................
Ga0316051_1003245/138-56       CAGCGGUCCAGG.GCGU.................UGGAAU..............................................................................................................
Ga0316049_122425/209-288       CAGCGGUCCAGG......................GCGUAAG.............................................................................................................
Ga0184588_113246/331-252       CAGCGGUCCG........................UGGC................................................................................................................
Ga0184582_111448/113-237       UUGCGGUCCAGC.U....................UU..................................................................................................................
Ga0184597_112736/543-466       UUGCGGUCCAGC.U....................UU..................................................................................................................
Ga0184577_100528/550-473       UUGCGGUCCAGC.U....................UU..................................................................................................................
Ga0127502_10397722/141-209     GUGCAGUCGGGG......................ACCCA...............................................................................................................
Ga0184593_119512/675-608       UUGCAGUCGGGG.A....................UC..................................................................................................................
Ga0307482_1597388/86-18        UUGCAGUCGAGU.C....................GU..................................................................................................................
Ga0307482_1001973/47-115       UUGCAGUCGAGU.C....................GU..................................................................................................................
Ga0184595_132469/902-822       AUGCGGUCCGG.......................UAGGAC..............................................................................................................
Ga0079039_1443516/199-38       CGUCGGUCCUUU.CCAG.G..CU...CCACCCCUUCCAA...............................................................................................................
Ga0265756_100130/1310-1239     UAGCGGUCCCAG.CAUC.A...................................................................................................................................
Ga0265747_125875/320-395       GUUCGGUCCCCG.CGU..................AGGC................................................................................................................
Ga0180052_189627/240-346       GGUCGGUCCCAU.CAUC....CA...CCGCGGCUAGUUA...............................................................................................................
Ga0180044_1074338/112-33       UGACGGUCCUAA.U....................GA..................................................................................................................
Ga0157559_1047848/128-41       GUUCGGUCCCUC.U.......AU...........GAAGCCCCG...........................................................................................................
Ga0157562_1134247/69-160       GUUCUGUCCCUC.U.......AU...........GAAGCCCCG...........................................................................................................
Ga0247548_101188/825-888       GAUCGGUCCCA.......................GA..................................................................................................................
Ga0228707_1093045/293-207      CGUCUGUCCUC.......................AUC.................................................................................................................
Ga0184597_119383/488-559       UAGCGGUCCGG.......................AACAU...............................................................................................................
Ga0184602_121040/35-106        UAGCGGUCCGGU.AA.......................................................................................................................................
Ga0326631_107633/416-489       UAGCGGUCCGGU.AA...................AA..................................................................................................................
Ga0316046_149266/91-162        UAGCGGUCCGGU.AA.......................................................................................................................................
Ga0247538_100917/537-466       UAGCGGUCCGGU.AA.......................................................................................................................................
Ga0247519_118271/334-405       UAGCGGUCCGGG.A........................................................................................................................................
Ga0265747_100454/1395-1324     UAGCGGUCCGUU.A....................ACUA................................................................................................................
Ga0316046_100502/370-442       UAGCGGUCCAUC.AA...................UU..................................................................................................................
Ga0247551_101484/617-543       UAGCGGUCUGUC.AAUU.....................................................................................................................................
Ga0213850_1280121/544-351      UGUCUGUCCCCC.UGGA.CA..............ACCAAUUCCACUCUGCAUCGCAUGAAGUAUUAG...................................................................................
Ga0265759_100017/4211-4288     ACGCAGUCCCAU.C....................AUCAAUGC............................................................................................................
Ga0307482_1127544/505-398      GGUCUGUCCGCG.G.......GG...............................................................................................................................
Ga0180107_1057591/210-107      UAGUUGUCCAGA.UAUA.U...................................................................................................................................
Ga0157569_1061476/44-119       CGUCUGUCCC........................GA..................................................................................................................
Ga0307484_100889/412-594       UGUCUGUCCCUA.GUUC.GG.........GAAUUAACCA...............................................................................................................
Ga0310106_100022/563-489       AUACGGUCCC........................UUA.................................................................................................................
Ga0310115_100154/4483-4559     AUACGGUCCC........................UUA.................................................................................................................
Ga0180107_1262812/2765-2832    UGACGGUCCCU.......................UU..................................................................................................................
Ga0184597_110025/865-793       CUACGGUCCAUC.C....................GAU.................................................................................................................
Ga0247550_100495/487-560       CUACGGUCCAUC.C....................GAU.................................................................................................................
Ga0184582_135749/62-135        UUACGGUCCAUC.C....................GAU.................................................................................................................
Ga0184603_122688/908-835       UUACGGUCCAUC.C....................GAU.................................................................................................................
Ga0247524_126530/125-198       UUACGGUCCAUC.C........................................................................................................................................
Ga0316030_115438/368-435       CAUCAGUCCCUC......................GAA.................................................................................................................
Ga0307354_155926/276-116       UGUUUGUCCCUU.UGG..................CACUGCUGCAACUUUGCACAGCUGUUUGAGGCCCGCUGCCGGA.........................................................................
Ga0079074_1279626/114-274      UGUUUGUCCCUU.UGG..................CACUGCUGCAACUUUGCACAUCUGUUUGAGGCCCGCU...............................................................................

                               <<<<....<<<<.<<<<.<<.........<<<<<....................................................................................................................
                               2222....2222.22.......................................................................................................................................
                               ●●●C●GUCC●●●-○○-------------------○○○-----------------------------------------------------------------------------------------------------------------
```

```
Ga0184602_132007/314-246        UGUCGGUCCCUC.U......................................................................................................................................
Ga0307921_1005950/791-877       GGUCGGUCCCUC.UC...................AGAAC..............................................................................................................
Ga0242654_10000487/4544-4475    GGUCGGUCCCUC.U......................................................................................................................................
Ga0157569_1032488/120-205       AGUCGGUCCAGC.A....................AAAUCCU............................................................................................................
Ga0157593_1125711/213-128       AGUCGGUCCAGC.A....................AAAUCCU............................................................................................................
Ga0157565_1077069/156-67        AGUCGGUCCAAG.C.......AA...A.........................................................................................................................
Ga0180047_1128081/151-64        AGUCGGUCCAAG.C.......AA...A.........................................................................................................................
Ga0265756_135986/152-290        GUUUUGUCCCGC.GCC.....A............AACAACCCGCU........................................................................................................
Ga0180045_119797/166-305        GUUCGGUCCAUA.AC.....................................................................................................................................
Ga0115595_1089740/408-494       GGUCGGUCUAAC.U....................AAAA...............................................................................................................
Ga0179955_1048341/123-51        GGUCGGUCCGGU........................................................................................................................................
Ga0138280_1140856/341-479       GGUCGGUCCG.G.AGGC.GC.........GGUGCAUUUCA.............................................................................................................
Ga0180106_1302226/203-273       GGUCGGUCCUGC.A....................GCC................................................................................................................
Ga0310119_169621/253-177        GGACGGUCCCCC.A....................UCCUUUU............................................................................................................
Ga0138277_1023917/182-18        GGUCGGUCCA.G.AGGC.GC.........GCAUCACGUCU.............................................................................................................
Ga0138282_1020546/737-573       GGUCGGUCCA.G.AGGC.GC.........GCAUCACGUCU.............................................................................................................
Ga0179939_1059820/107-216       GGUCGGUCCAAC.AGCU.................UA.................................................................................................................
Ga0179950_1098211/345-278       GGUCGGUCCAAC.A....................AG.................................................................................................................
Ga0115594_1068468/127-56        GGUCGGUCCGGA.U....................UGUCU..............................................................................................................
Ga0228708_1103570/188-285       GGUCGGUCCGAC.UGUG.UC.G............AUUU...............................................................................................................
Ga0265751_160438/108-215        UGUCAGUCCACA.G...............UGAGCACAAAC.............................................................................................................
Ga0242647_1000066/2638-2719     AAACGGUCCCCU.AC................U....................................................................................................................
Ga0242646_1001227/1243-1308     CAUCGGUCCCU.........................................................................................................................................
Ga0182091_1104347/98-194        UGUUUGUCCCUC.AAGA.AG.AA...U.......CAA................................................................................................................
Ga0115594_1178636/111-46        AGUCGGUCCC........................GA.................................................................................................................
Ga0213850_1508869/496-402       CGUCUGUCCUUC.AACU.GC.GA...U.......CA.................................................................................................................
Ga0307482_1152465/114-38        CAGCGGUCCU..........................................................................................................................................
Ga0180107_1031754/139-57        CAUCUGUCCCUA.ACAC...................................................................................................................................
Ga0316037_121702/176-240        CAUCGGUCCC........................GU.................................................................................................................
Ga0265759_107962/103-39         CAUCGGUCCC..........................................................................................................................................
Ga0242649_1051170/368-302       CAUCGGUCCC........................GA.................................................................................................................
Ga0307482_1064439/84-153        CAUCAGUCCC........................GAA................................................................................................................
Ga0115594_1006122/120-29        UGUCUGUCCUAC.C.......AA...........CUUAAUA............................................................................................................
Ga0115595_1180708/226-317       UGUCUGUCCUAC.C.......AA...........CUUAAUA............................................................................................................
Ga0265746_1064626/389-458       CGUCUGUCCUAC.C....................AU.................................................................................................................
Ga0265759_108120/277-351        GGUCUGUCUCAC.UU...................UCA................................................................................................................
Ga0307482_1000450/2015-2077     GGUCUGUCCC........................GAG................................................................................................................
Ga0247527_115808/280-440        CGUCUGUCCCUA.CUUC.GG.........GGGU.GCCAA..............................................................................................................
Ga0184588_120624/266-426        AAUCUGUCCCUA.UUUC.GG.........GGGU.GCCAA..............................................................................................................
Ga0184579_106194/996-1156       AGUCUGUCCCUA.UUUC.GG.........GGGU.GCCAA..............................................................................................................
Ga0265758_100254/356-516        AGUCUGUCCCUA.UUUC.GG.........GGGU.GCCAA..............................................................................................................
Ga0316035_107544/354-514        CGUCUGUCCCUA.CUUC.GG.........GGGU.GCCAA..............................................................................................................
Ga0184596_109261/979-1139       CGUCUGUCCCUA.CUUC.GG.........GGGU.GCCAA..............................................................................................................
Ga0184600_132066/568-408        AGUCUGUCCCUA.UUUC.GG.........GGGU.GCCAA..............................................................................................................
Ga0184597_106861/196-36         AAUCUGUCCCUA.UUUC.GG.........GGGU.GCCAA..............................................................................................................
Ga0316031_108320/216-376        AAUCUGUCCCUA.UUUC.GG.........GGGU.GCCAA..............................................................................................................
Ga0184589_110648/1041-881       UUUCUGUCCCUC.CUUC.GG.........GGGU.GCCAA..............................................................................................................
Ga0184580_124121/239-79         UUUCUGUCCCUA.CUCC.GG.........GGGU.GCCAA..............................................................................................................
Ga0184598_101324/530-690        UUUCUGACCCUA.CUCC.GG.........GGGU.GCCAA..............................................................................................................
Ga0247547_102464/189-29         AGUCUGUCCCUA.CUUC.GG.........GGGU.GCCAA..............................................................................................................
Ga0247551_100828/546-387        AGUCUGUCCCUA.CUUC.GG.........GGGU.GCCAA..............................................................................................................
Ga0247536_101914/360-201        AGUCUGUCCCUA.CUUC.GG.........GGGU.GCCAA..............................................................................................................
Ga0247538_100480/788-947        AAUCUGUCCCUA.CUUC.GG.........GGGU.GCCAA..............................................................................................................
Ga0247550_100850/456-297        AAUCUGUCCCUA.CUUC.GG.........GGGU.GCCAA..............................................................................................................
Ga0247553_110698/526-367        AGUCUGUCCCUA.CUUC.GG.........GGGU.GCCAA..............................................................................................................
Ga0184589_112135/40-200         UAUCUGUCCCUA.CUUC.GG.........GGGU.GCCAA..............................................................................................................
Ga0247526_110221/603-443        AAUCUGUCCCUA.CUUC.GG.........GGGU.GCCAA..............................................................................................................
Ga0247541_103784/123-283        AAUCUGUCCCUA.CUUC.GG.........GGGU.GCCAA..............................................................................................................
Ga0247545_112960/183-343        AAUCUGUCCCUA.CUUC.GG.........GGGU.GCCAA..............................................................................................................
Ga0184586_133850/368-528        AAUCUGUCCCUA.CUUC.GG.........GGGU.GCCAA..............................................................................................................
Ga0247552_101742/612-452        AAUCUGUCCCUA.CUUC.GG.........GGGU.GCCAA..............................................................................................................
Ga0247545_104341/406-246        AAUCUGUCCCUA.CUUC.GG.........GGGU.GCCAA..............................................................................................................
Ga0184602_114416/11-171         AAUCUGUCCCUA.CUUC.GG.........GGGU.GCCAA..............................................................................................................
Ga0316038_111670/11-171         AAUCUGUCCCUA.CUUC.GG.........GGGU.GCCAA..............................................................................................................
Ga0247529_134913/224-64         AAUCUGUCCCUA.CUUC.GG.........GGGU.GCCAA..............................................................................................................
Ga0184603_143085/253-93         AAUCUGUCCCUA.CUUC.GG.........GGGU.GCCAA..............................................................................................................
Ga0316032_107575/215-55         AAUCUGUCCCUA.CUUC.GG.........GGGU.GCCAA..............................................................................................................
Ga0247519_110701/607-447        AAUCUGUCCCUA.CUUC.GG.........GGGU.GCCAA..............................................................................................................
Ga0247524_111427/50-210         AAUCUGUCCCUA.CUUC.GG.........GGGU.GCCAA..............................................................................................................
Ga0184574_121290/269-109        AAUCUGUCCCUA.CUUC.GG.........GGGU.GCCAA..............................................................................................................
Ga0184595_111222/3-163          AAUCUGUCCCUA.CUUC.GG.........GGGU.GCCAA..............................................................................................................
Ga0184600_123882/188-28         AAUCUGUCCCUA.CUUC.GG.........GGGU.GCCAA..............................................................................................................
Ga0326631_101563/544-705        AGUCUGUCCCUA.CUUC.GG.........GGGU.GCCAA..............................................................................................................
Ga0184573_110190/115-276        AAUCUGUCCCUA.CUUC.GG.........GGGU.GCCAA..............................................................................................................
Ga0184596_111007/525-686        AGUCUGUCCCUA.CUUC.GG.........GGGU.GCCAA..............................................................................................................
Ga0316033_144939/167-328        AGUCUGUCCCUA.CUUC.GG.........GGGU.GCCAA..............................................................................................................
Ga0247526_117235/152-313        UUUCUGUCCCUA.CUUC.GG.........GGGU.GCCAA..............................................................................................................
Ga0247551_100195/490-329        UUUCUGUCCCUA.CUUC.GG.........GGGU.GCCAA..............................................................................................................
Ga0247538_100424/120-281        AAUCUGUCCCUA.CUUC.GG.........GGGU.GCCAA..............................................................................................................
Ga0247550_114736/167-7          AAUCUGUCCCUA.CUUC.GG.........GGGU.GCCAA..............................................................................................................
Ga0247529_103231/270-430        AAUCUGUCCCUA.CUUC.GG.........GGGU.GCCAA..............................................................................................................
Ga0184603_111918/241-82         AAUCUGUCCCUA.CUUC.GG.........GGGU.GCCAA..............................................................................................................
Ga0184603_126764/80-240         AAUCUGUCCCUA.CUUC.GG.........GGGU.GCCAA..............................................................................................................
Ga0247519_107918/217-58         AAUCUGUCCCUA.CUUC.GG.........GGGU.GCCAA..............................................................................................................
Ga0184580_114961/306-146        AAUCUGUCCCUA.CUUC.GG.........GGGU.GCCAA..............................................................................................................
Ga0213850_1042834/351-428       AGACGGUCCCGC.CC...................AA.................................................................................................................
Ga0265756_103656/97-163         CGACGGUCCCC.......................AUCC...............................................................................................................
Ga0265756_101189/896-833        CGACGGUCCCC.......................AU.................................................................................................................
Ga0180107_1033011/414-478       AGACGGUCCCC.......................UU.................................................................................................................

                                <<<<....<<<<.<<<<.<<.........<<<<<...................................................................................................................
                                2222....2222.22......................................................................................................................................
                                •••C•GUCC•••-∘∘-------------------∘∘∘---------------------------------------------------------------------------------------------------------------
```

```
Ga0115594_1157594/155-254          UGUCUGUCCGUA.C.......AA...AUA..........................................................................................................................
Ga0115594_1166236/119-19           UGUCUGUCCGUA.C.......AA...AUA..........................................................................................................................
Ga0115594_1007070/120-30           UGUCUGUCCGUA.C.......AU...UAA..........................................................................................................................
Ga0115595_1181384/176-86           UGUCUGUCCGUA.C.......AU...UAA..........................................................................................................................
Ga0115594_1211214/299-389          CGUCUGUCCGCA.C.......AA...AU......AAAG.................................................................................................................
Ga0115594_1182529/183-84           UGUCUGUCCGUA.C.......AA...AUA.....AAGGCGUAC............................................................................................................
Ga0256352_1182139/180-81           UGUCUGUCCGUA.C.......AA...A.......UAAGGGCGU............................................................................................................
Ga0179944_1005311/261-382          GGUCGGUCCUUC.C.......G.................................................................................................................................
Ga0213850_1508120/373-299          CGUCAGUCCCGA.G.........................................................................................................................................
Ga0307484_162891/325-258           CGUCGGUCCCU............................................................................................................................................
Ga0184596_111635/86-148            CGUCGGUCCCC.......................AAC..................................................................................................................
Ga0316051_1005111/447-509          CGUCGGUCCCC.......................AA...................................................................................................................
Ga0265753_1379003/85-148           CGUCGGUCCCC.......................AA...................................................................................................................
Ga0184603_116647/206-269           CGUCGGUCCCC.......................AA...................................................................................................................
Ga0184587_132427/565-485           CGUCGGUCCCUU.UCCC.................UCU..................................................................................................................
Ga0184599_110049/275-349           CGUCGGUCCCAU.UCCC.................UC...................................................................................................................
Ga0184603_148109/996-920           CGUCGGUCCCAU.UCCC.................UC...................................................................................................................
Ga0310114_133004/56-132            CGUCGGUCCCAU.UCCC.................UC...................................................................................................................
Ga0310119_113172/381-306           CGUCGGUCCCAU.UCCC.................UC...................................................................................................................
Ga0265747_119090/8-84              UGUCGGUCCGCG.CU...................UUAGGUG..............................................................................................................
Ga0247551_122551/29-115            CUGCGGUCCAAU.AU.......A...AUAG.........................................................................................................................
Ga0316048_107802/239-324           CUGCGGUCCAAU.AU.......A...AUA..........................................................................................................................
Ga0242657_1208314/452-382          GUGCGGUCCAGU.AU........................................................................................................................................
Ga0184596_129213/83-168            GUGCGGUCCAUU......................CAAAUG...............................................................................................................
Ga0184603_146917/441-510           GUGCGGUCCAUU.CC...................UU...................................................................................................................
Ga0247550_102601/160-89            GUGCGGUCCAUU......................CGUAA................................................................................................................
Ga0184602_110024/263-177           GUGCGGUCCAUU......................CAUAAU...............................................................................................................
Ga0184597_116115/700-787           GUGCGGUCCAUU......................CGUAAA...............................................................................................................
Ga0184582_128596/700-786           GUGCGGUCCAUU......................CGUAAU...............................................................................................................
Ga0184597_102019/330-402           GUGCGGUCCAUU......................CGUAAU...............................................................................................................
Ga0242649_1000320/700-631          AUGCGGUCCUC.......................CUUA.................................................................................................................
Ga0179943_1102267/215-90           GGUCGGUCCGUC.G.......G.................................................................................................................................
Ga0179944_1191552/284-411          CGUCGGUCCAUC.G.......G.................................................................................................................................
Ga0179943_1150468/216-89           GGUCGGUCCUUC.A.......G.................................................................................................................................
Ga0179944_1204933/216-89           GGUCAGUCCUUC.A.......G.................................................................................................................................
Ga0265758_104558/436-365           UAGCGGUCCUCC.C....................UCAACA...............................................................................................................
Ga0180107_1040315/471-547          AUGCGGUCCGGC.UC...................UA...................................................................................................................
Ga0180106_1332058/4386-4449        GUGCGGUCCC........................GAG..................................................................................................................
Ga0307484_104189/90-171            UUACGGUCCGAC.GAAC.................CUUGCU...............................................................................................................
Ga0265752_100023/1212-1290         CAACGGUCCAUC......................AU...................................................................................................................
Ga0157593_1101573/285-478          UAGCUGUCCCCC.ACA.............CGUCCCACA.................................................................................................................
Ga0307482_1008742/1212-1288        UCGCAGUCCCCG.CAUU.................UUU..................................................................................................................
Ga0247551_104770/284-362           CUUCUGUCCGGC...........................................................................................................................................
Ga0184593_112903/290-212           CUUCUGUCCGGC...........................................................................................................................................
Ga0307482_1128390/271-339          UUGCGGUCCGGC.C....................CA...................................................................................................................
Ga0184580_126177/800-732           CUGCGGUCCGGG.G....................AA...................................................................................................................
Ga0242646_1115622/191-109          CAGCGGUCCACG.GAUU.GU.G............AAU..................................................................................................................
JGI12272J11983_1319142/230-159     GUGCGGUCCGGG.G.........................................................................................................................................
Ga0184596_105062/163-229           GCGCGGUCGGGG.G....................AC...................................................................................................................
Ga0307482_1011891/77-10            GCGCGGUCGGGG.G....................AC...................................................................................................................
Ga0184588_125064/416-488           GUGCGGUCCAGG.U....................UAUUA................................................................................................................
Ga0184577_107529/254-327           GUGCGGUCCAGG.U....................UAUUUU...............................................................................................................
Ga0180106_1182034/403-337          GUGCGGUCGGGG......................AG...................................................................................................................
Ga0180107_1241189/1554-1620        GUGCGGUCGGGG......................AA...................................................................................................................
Ga0222728_1000110/4754-4821        AUGCGGUACGGG.A....................CC...................................................................................................................
Ga0307484_100154/562-629           AUGCGGUACGGG.A....................CC...................................................................................................................
Ga0180107_1285439/94-155           CUGCGGUCC.........................GUCA.................................................................................................................
Ga0184580_116530/249-177           CUGCGGUCCAGA.C....................AUA..................................................................................................................
Ga0247526_117664/434-499           CAGCGGUCCAUG...........................................................................................................................................
Ga0184580_120219/79-4              AUGCGGUCCAAC.GUCU......................................................................................................................................
Ga0247539_100482/682-612           UAGCGGUCUACG.C....................GU...................................................................................................................
Ga0247516_124175/202-272           UAGCGGUCUACA.C.........................................................................................................................................
Ga0184580_121247/966-1036          UAGCGGUCUACA.C....................UU...................................................................................................................
Ga0265743_100411/1096-1162         CAGCGGUCCAUA.C.........................................................................................................................................
Ga0242657_1070636/108-176          CAGCAGUCCCUC.U.........................................................................................................................................
Ga0242657_1122750/95-162           CAGCAGUCCCUC.UG........................................................................................................................................
Ga0307482_1405960/253-322          UAGCGGUCCACC.C....................CA...................................................................................................................
Ga0316049_154968/227-319           UUACGGUCCUAU.G.........................................................................................................................................
Ga0127502_10852756/278-345         UCGCGGUCCAUC......................CU...................................................................................................................
Ga0247534_100003/3657-3582         UGACGGUCCAUC......................AUC..................................................................................................................
Ga0180106_1204848/284-221          GUGCGGUCCACC.A.........................................................................................................................................
Ga0184585_128240/467-390           GUGCGGUCCUCC.ACAA.................UCCAU................................................................................................................
Ga0180106_1180558/1549-1486        GUGCGGUCCAC.......................CAG..................................................................................................................
Ga0265755_100134/298-372           GAACGGUCCGGG.G....................CCU..................................................................................................................
Ga0247534_100772/309-234           GAACGGUCCGUA......................CAACCGU..............................................................................................................
Ga0307482_1202063/402-466          GAGCGGUCCCC.......................UU...................................................................................................................
Ga0247514_114593/280-211           AGGCAGUCCCUC...........................................................................................................................................
Ga0310101_157764/124-55            GGGCAGUCCCUC...........................................................................................................................................
Ga0265745_1000136/129-60           AGGCAGUCCCUC...........................................................................................................................................
Ga0265745_1013560/201-270          AGGCAGUCCCUC...........................................................................................................................................
Ga0307482_1779657/200-270          UUGCGGUCCCUU.C....................UC...................................................................................................................
Ga0307482_1044183/658-591          UUGCGGUCCCU.......................CAA..................................................................................................................
Ga0265758_100017/4376-4309         GAGCGGUCCCU............................................................................................................................................
Ga0242654_10617983/312-237         CUGCGGUCCAAU.GAU.......................................................................................................................................
Ga0222728_1054736/119-193          UAGCGGUCCCUA.CAA..................UUGAA................................................................................................................
Ga0184583_106441/296-227           UAGCUGUCCCCA.C....................UU...................................................................................................................
Ga0247520_104849/891-821           GUACGGUCCCAU.C.........................................................................................................................................
Ga0247518_137824/301-231           GUGCGGUCCCAU.C.........................................................................................................................................
Ga0247531_104861/1082-1012         GUGCGGUCCCAU.C.........................................................................................................................................

                                   <<<<....<<<<.<<<<.<<.........<<<<<.....................................................................................................................
                                   2222....2222.22........................................................................................................................................
                                   ●●●C●GUCC●●●–∘∘-------------------∘∘∘--------------------------------------------------------------------------------------------------------------------
```

```
Ga0265746_1040401/190-123        GUGCGGUCCCUA.A........................................................................................................................................
Ga0184603_139760/199-128         GAGCGGUCCCUU.C....................UU..................................................................................................................
Ga0184603_138085/381-311         GAGCGGUCCCUU.C....................UU..................................................................................................................
Ga0265747_102820/448-376         GAGCGGUCCCUU.CUU......................................................................................................................................
Ga0247554_105388/388-316         GAGCGGUCCCUU.CUU......................................................................................................................................
Ga0316037_123461/289-368         AAGCGGUCCGUC.AUG..................UCCAUC..............................................................................................................
Ga0184597_121517/262-183         AAGCGGUCCGUC.AUG..................UCCAUC..............................................................................................................
Ga0247551_100118/653-723         GAGCGGUCCCCU.C........................................................................................................................................
Ga0265750_1365815/134-55         AAGCGGUCCGUC..........................................................................................................................................
Ga0184589_120335/11-90           AAGCUGUCCGUU..........................................................................................................................................
Ga0247521_124040/388-309         AAGCUGUCCGU..........U................................................................................................................................
Ga0247518_108423/758-837         AAGCGGUCCGU..........U................................................................................................................................
Ga0247531_104608/1256-1177       AAGCGGUCCGU..........U................................................................................................................................
Ga0247543_100863/642-721         AAGCGGUCCGUU..........................................................................................................................................
Ga0307482_1026216/884-813        AUGCGGUCCCUA.UCC..................UA..................................................................................................................
Ga0307482_1636410/270-341        AUGCGGUCCCUA.UCC......................................................................................................................................
Ga0310119_128165/432-365         GUGCGGUCCCCU......................CA..................................................................................................................
Ga0307482_1016877/168-97         AUGCGGUCCCCA.U....................CCU.................................................................................................................
Ga0126318_10746885/3334-3265     UAGCGGUCCCCU.C........................................................................................................................................
Ga0206353_11117566/971-1032      UUGCGGUCCCU...........................................................................................................................................
Ga0180107_1106188/424-495        AUGCGGUCCG........................CCGAUU..............................................................................................................
Ga0307482_1000354/2063-1987      AUGCGGUCCG........................CAGCAUGUA...........................................................................................................
Ga0180107_1132096/355-287        UUGCGGUCCUCU.U....................CA..................................................................................................................
Ga0180108_1238667/303-371        UUGCGGUCCUCU.U........................................................................................................................................
Ga0265751_100981/715-788         CUGCGGUCCACA.AC.......................................................................................................................................
Ga0265756_100898/263-336         CUGCGGUCCACA.AC.......................................................................................................................................
Ga0307484_102094/153-80          ACGCGGUCCCAC.U....................UC..................................................................................................................
Ga0307482_1023270/273-197        UUGCGGUCCGCC.CC...................ACUAAA..............................................................................................................
Ga0184596_102741/625-701         AUACGGUCCCCU.AUGU.C...................................................................................................................................
Ga0316033_103637/453-391         AUGCGGUCCC............................................................................................................................................
Ga0180107_1069127/170-106        CUACGGUCCAUA......................CU..................................................................................................................
Ga0247535_105396/277-348         CUGCGGUCCAGG.GG...................UU..................................................................................................................
Ga0184576_110410/277-348         CUGCGGUCCAGG.GGU..................UC..................................................................................................................
Ga0307482_1219330/19-95          GUGCGGUCCCCA.UACC.A...............GU..................................................................................................................
Ga0247534_116177/42-105          AUACGGUCCUC.......................UU..................................................................................................................
Ga0316033_123149/165-228         AUACGGUCCUC.......................UU..................................................................................................................
Ga0265751_100560/642-572         GUACGGUCCCCU......................UG..................................................................................................................
Ga0307482_1011142/698-628        GUGCGGUCCCG.......................CAAGA...............................................................................................................
Ga0307482_1465843/259-190        GUGCGGUCCCG.......................CAAGA...............................................................................................................
Ga0184593_115334/333-240         UUACGGUCCCAU.G.......U............A...................................................................................................................
Ga0265751_100089/3005-3069       GUACGGUCCC........................GAA.................................................................................................................
Ga0307482_1000325/380-315        GUACGGUCCCA.......................CAG.................................................................................................................
Ga0222729_1014021/463-392        AGGCGGUCCCU...........................................................................................................................................
Ga0307482_1033651/424-494        AUGCGGUCCAUA.UA.......................................................................................................................................
Ga0307482_1015330/1021-1093      UAGCGGUCCGGA.A....................AGCAAGA.............................................................................................................
Ga0184570_111551/35-97           UAGCGGUCCAG.......................AC..................................................................................................................
Ga0184576_117861/362-299         UAGCGGUCCAG.......................AC..................................................................................................................
Ga0307482_1589279/351-280        UUGCGGUCCAAG.C....................AACUU...............................................................................................................
Ga0184586_104419/174-104         UUGCGGUCCGGG.U....................AA..................................................................................................................
Ga0222728_1148620/185-250        UUGCGGUCCAGA......................CG..................................................................................................................
Ga0316049_103217/889-823         UUGCGGUCCGGA.U........................................................................................................................................
Ga0316034_104024/504-439         UUGCUGUCCGGA.U....................AA..................................................................................................................
Ga0184595_117496/296-230         UUGCGGUCCGGA......................CA..................................................................................................................
Ga0184597_109849/897-963         UUGCGGUCCGGA......................GA..................................................................................................................
Ga0184588_134146/1219-1285       UUGCAGUCCGGA......................GA..................................................................................................................
Ga0184584_115247/186-121         UUGCGGUCCGGA......................CAA.................................................................................................................
Ga0184596_126546/83-162          UAGCGGUCCAGA.GGAA.U...............GUAG................................................................................................................
Ga0184570_116436/278-198         CAGCGGUCCAGG.GGUA.UA.G................................................................................................................................
Ga0184597_111165/262-182         UAGCGGUCCAGG.GGUA.UA.G................................................................................................................................
Ga0316037_169124/279-199         CAGCGGUCCAGG.GGUA.UA.G................................................................................................................................
Ga0247551_103383/322-242         UAGCGGUCCAGA.GGUA.UA.G................................................................................................................................
Ga0247543_103170/244-164         UAGCGGUCCAGA.GAUA.UA..............G...................................................................................................................
Ga0184589_120451/249-329         UAGCGGUCCAGA.GAUA.UA..............G...................................................................................................................
Ga0184600_118565/177-97          UAGCGGUCCAGG.UGUA.UA..............GUA.................................................................................................................
Ga0316034_100493/254-174         UAGCGGUCCAGG.UGUA.UA..............GUA.................................................................................................................
Ga0184576_117703/380-460         UAGCGGUCCAGG.GGUG.UA.GU...........AGA.................................................................................................................
Ga0184597_129399/366-446         UAGCGGUCCAGG.GGUG.UA.GU...........AGA.................................................................................................................
Ga0184590_111002/143-223         UAGCGGUCCAGA.GAUA.UA..................................................................................................................................
Ga0184587_105985/71-151          UAGCGGUCCAGG.GGU..................CAUGU...............................................................................................................
Ga0184602_122864/595-515         UAGCAGUCCAGG.GGU..................CAU.................................................................................................................
Ga0184593_101086/682-762         UAGCGGUCCAGA.GGU..................CAUGUA..............................................................................................................
Ga0247519_108375/286-206         UAGCGGUCCAGG.G........................................................................................................................................
Ga0127502_10351452/615-550       GAGCGGUCCCCC......................UAUC................................................................................................................
Ga0316038_112263/442-356         UUGCGGUCCUCC.A........................................................................................................................................
Ga0242657_1073895/573-672        GUGCGGUCCCGA.UC.......................................................................................................................................
Ga0184587_107783/167-95          AUGCGGUCCUGG.UG...................GAC.................................................................................................................
Ga0265758_101918/310-243         AUGCGGUCCUUC......................GG..................................................................................................................
Ga0307482_1029599/655-579        CUGCGGUCCGGG.GG...................UA..................................................................................................................
Ga0265746_1009702/779-848        CUGCGGUCUGGG.G....................CCAAU...............................................................................................................
Ga0184583_113025/102-26          UAGCGGUCCAG.......................GGUGACAG............................................................................................................
Ga0184580_109961/270-194         UAGCGGUCCAGG......................GGUACAGA............................................................................................................
Ga0316049_111814/256-181         UAGCGGUCCAGG......................GUGCAGA.............................................................................................................
Ga0247553_120631/200-275         UAGCGGUCCAGG......................GCGCAG..............................................................................................................
Ga0184593_119849/313-238         UAGCGGUCCAGG..........................................................................................................................................
Ga0184599_140898/774-849         UAGCGGUCCAGG..........................................................................................................................................
Ga0242647_1005188/439-360        GUGCUGUCCGG.......................CAGUA...............................................................................................................
Ga0242657_1054283/541-620        GUGCUGUCCGG.......................CAGUAA..............................................................................................................
Ga0184597_102100/600-669         GUGCGGUCCAG.......................AAU.................................................................................................................
Ga0213850_1460186/227-149        UGACGGUCCAAC.CC...................AA..................................................................................................................

                                 &lt;&lt;&lt;&lt;....&lt;&lt;&lt;&lt;.&lt;&lt;&lt;&lt;.&lt;&lt;.........&lt;&lt;&lt;&lt;&lt;...................................................................................................................
                                 2222....2222.22.......................................................................................................................................
                                 ●●●C●GUCC●●●–∘∘–––––––––––––––––––∘∘∘–––––––––––––––––––––––––––––––––––––––––––––––––––––––––––––––––––––––––––––––––––––––––––––––––––––––––––––––––
```

```
Ga0242654_10040657/1018-942    CCGCAGUCCCUA.CGCA.A...............GC..................................................................................................................
Ga0307484_100842/1194-1271     ACGCAGUCCCUA.UGCA.................CU..................................................................................................................
Ga0242654_10401151/84-171      CAUCUGUCCGAU.CUCC.................GC..................................................................................................................
Ga0310119_105137/743-673       CUGCGGUCCUCU.AA...................UU..................................................................................................................
Ga0247541_104364/333-188       GAUUUGUACUUA.GUUG.AU.CC...AAAAG...AGAAACCUUAAGAGGUAUUCU...............................................................................................
Ga0184582_129125/749-675       GUGCUGUACUGA.CUC..................AG..................................................................................................................
Ga0265753_1022489/457-531      GUGCUGUACUGA.CUC..................AG..................................................................................................................
Ga0265751_121301/189-266       CGUCAGUCCGGU.GUUU.................UGA.................................................................................................................
Ga0247526_105246/689-508       UCUCUGUCUCUG.AUUC.G..........GGUUUUGCUA...............................................................................................................
Ga0247515_107373/548-731       UCUCUGUCUCUG.AUUC.G..........GGAUUUGCUA...............................................................................................................
Ga0247538_100442/1363-1181     UCUCUGUCUCUG.AUUC.G..........GGAUUUGCUA...............................................................................................................
Ga0247538_100551/721-542       UCUCUGUCUCUG.AUUC.G..........GGAUUUGCUA...............................................................................................................
                               <<<<....<<<<.<<<<.<<.........<<<<<....................................................................................................................
                               2222....2222.22.......................................................................................................................................
                               ●●●C●GUCC●●●-○○-------------------○○○-----------------------------------------------------------------------------------------------------------------
```

alignment positions 151 · · · 300

```
Ga0316035_118720/195-42        ......................................CUUGG.AAU........GU....GUGUCU.............................AACUACUCGUAAAGGUUCUAGAAGCCUUACGAG.....................
Ga0265747_125480/96-202        ......................................CAAGC.CGA.................................................CAAUAGUCAAAGCAG.......................................
Ga0242657_1353977/135-311      ......................................................................................................................................................
Ga0242649_1051170/34-143       ..............................UGC...AC.................GCC.C....................................AAGU..................................................
Ga0265758_100138/1527-1633     ..............................UGU............................................................ACCAAUGCCG...............................................
Ga0184593_103988/348-257       .......................................................GUA.U.C..................................CUAA..................................................
Ga0316047_106478/13-108        ..............................AGA...ACC.........................................................AAU...................................................
Ga0213850_1056422/14-139       ................................................................................................GACUCUUUCACCACCUUA....................................
Ga0316032_128094/136-241       .......................................................GGA.U.U.............................ACAUCUAUGUC................................................
Ga0307482_1000311/1469-1363    ......................................UA...............GGU.G.A...............................CCCAAGU..................................................
Ga0242657_1051800/416-321      .............................................UA..U.....GCG.U....................................UAGC..................................................
Ga0307921_1050750/452-358      ................................................................................................CUACA.................................................
Ga0265756_117960/316-418       .........................................................................................U.CUUCGAUGGU.................................................
Ga0242654_10352202/387-307     ................................................................................................UAAGU.................................................
Ga0242652_1159268/284-150      ......................................UAAGC.GAU..A.....GGC.U....................................UGCGCCCCGUCGAAAGACGUGCUCGUAAAAUUCGUG..................
Ga0265744_102237/225-365       ..............................AGC...CUAAAGG.A...................................................GUG...................................................
Ga0316029_101396/632-732       .......................................................ACG.U.G..................................GUUACGCCGAGCGGCUA.....................................
Ga0265759_100202/139-258       ...............................GG...CCGAAGC.CCA.................................................AAUACCGU..............................................
Ga0179943_1118543/65-150       ................................................................................................GA....................................................
Ga0182091_1018330/100-226      .................................................U.....AGU.C.GAGCGC.............................GCAAU.................................................
Ga0127503_10119710/546-465     ................................................................................................UC....................................................
Ga0138277_1022592/399-305      ................................................................................................ACG...................................................
Ga0138278_1026470/183-287      ................................................................................................CAUGU.................................................
Ga0138277_1089473/408-304      ................................................................................................CAUG..................................................
Ga0247538_105452/166-82        ...............................AC...CC................................................................................................................
Ga0247554_102783/498-580       ................................................................................................UCGAC.................................................
Ga0138291_1177333/295-213      ................................................................................................AGU...................................................
Ga0210324_1290306/191-291      .......................................................AUG.G.G..................................CAAAA.................................................
Ga0242657_1026087/460-322      .......................................................AGC.G.C..........UAGGCUUUGACACCU.UA.UACCCCAAC..................................................
Ga0307482_1147486/107-4        ...............................AC...CAC........................................................CAAGCGGUGGGAG..........................................
Ga0184573_113483/125-229       ..............................ACU...CG..........................................................CUAC..................................................
Ga0184576_116834/47-151        ..............................ACU...CG..........................................................CUAC..................................................
Ga0184568_108419/492-395       ......................................CAAGA.CAG........GCC......................................AAGU..................................................
Ga0307482_1023050/594-496      ......................................CAAAA.ACG.................................................AGACUAGCAGUCC.........................................
Ga0179955_1013331/439-311      ................................................................................................AAUAGAGACCACCAAU......................................
Ga0184596_112078/314-191       ................................A...UG.................GUU.U.G..................................UAUUC.................................................
Ga0184602_108471/49-173        ................................A...UG.................GUU.U.G..................................UAUUC.................................................
Ga0247528_108647/438-562       .......................................................GUA.U.GGU................................UUGU..................................................
Ga0316039_120091/354-252       ................................................................................................GGGCCGAGUUGAC.........................................
Ga0307484_166952/62-163        ......................................GAAGA.AAC........GGG.U.C..................................AGC...................................................
Ga0180107_1244437/408-507      .......................................CAGA.....................................................AGGAUGUCAGUGAC........................................
Ga0265742_1065119/361-273      ................................................................................................UGGAA.................................................
Ga0316035_113756/173-88        ................................................................................................CU....................................................
Ga0206355_1134705/241-332      ................................................................................................UG....................................................
Ga0180107_1031754/4443-4551    ................................................................................................CCAAGUGGUGA...........................................
Ga0138292_1031932/14-100       ................................................................................................A.....................................................
Ga0316029_100807/1303-1197     ............................................ACA..G.....UGG.A.UCUAGA.............................AAGC..................................................
Ga0184568_114257/261-413       ................................................................................................GGUCCCUGUCAUCGACCCAGUCCUCAGAGGGAG.....................
Ga0242647_1005188/73-152       ................................................................................................GA....................................................
Ga0153880_1340646/556-457      .......................................................GUU.C.G..................................UAUUAGCAAG.A..........................................
Ga0307484_101303/704-866       ..............................AAC...CCCAGGU.ACA..C.....AGU.G.G..............CACGGAGGUGG.UG.CUCUC...U..................................................
Ga0242641_1025149/251-89       ..............................AAC...CCCAGGU.ACA..C.....AGU.G.GCAC.................GGAGG.AG.GUGCUCUCCG.................................................
Ga0242652_1002960/413-574      ..............................AAC...CCCAGGU.ACA..C.....AGU.G.GCAAU..............................GGAGAGGUGCUUCCAUGUACCAACCU............................
Ga0265754_1007706/448-552      ..............................GAU...CC..........................................................UAUAUCAAGU.GAU.GA.....................................
Ga0157562_1164332/672-560      ..............................ACA...AUAUA.......................................................AUACCCUAGCGACAACUU....................................
Ga0157569_1058012/474-586      ..............................ACA...AUAU........................................................AAUACCCUAGCGACAACUU...................................
Ga0307921_1027128/160-73       ................................................................................................GCC...................................................
Ga0138279_1083071/419-326      .......................................................GGU.C.C........................G.AC.GCGCACUAAAUGCG.............................................
Ga0127502_10704012/113-7       ......................................AGUGG.AUU..G.....CAU.C.A..................................AUGUCGA...............................................
Ga0222728_1000095/4541-4435    ......................................AGUGG.AUU..G.....CAU.C.A..................................ACGUCGA...............................................
Ga0265750_1166784/261-415      .......................................................GCG.U.U..................A...............GGCCGGAGCCUGGAUCAGAACUGCCGAUUACUCGGAGGUUCU............
Ga0310115_109341/244-400       .......................................................GCG.U.U..................................AGGCCGGAGCCUGGAUCAGAACUGCCCCGAUUACUCGGAGGUUCUCCAGCUUCA
Ga0265756_158829/72-188        ......................................CAAAC.AAA..U.....GUC......................................UUUCGUGAAA.AAG.AC.....................................
Ga0184583_104740/342-477       ................................................................................................CUCAGUUAGAGCUACGGCUUCAAGGCGUGUG.......................
Ga0180107_1215312/308-184      ..............................AGG.......................C.......................................UGCAUGUGACCCAAGUCUCACAUCAAAGA.........................
Ga0265742_1013613/579-453      ..............................GCG...CGCAAAU.UAA........GAU.G.UCGAGUGG...........................GC....................................................
Ga0265748_110851/279-405       ..............................GCG...CGCAAAU.UAA........GAU.G.UCGAGUGG...........................GC....................................................
Ga0265752_120463/277-403       ..............................CACGCACGCAAAU.UAA........GAU.G.ACGAGUGG...........................GC....................................................

                               ..............................>>>...>>.................<<<.<.<<<<<<<<<<<<<<<<<<<<<<<<<<.<<.<<<<<......................................................
                               ......................................................................................................................................................
                               ------------------------------------------------------------------------------------------------●○○○--------------------------------------------------
```

```
Ga0265759_115288/4-102        ......................................CAAGC.A..........AGA.G.C..................................AGU...................................................
Ga0242646_1001122/826-995     ..............................AAU...CCUAAGG.UAU........GGC.C.GGGAAAGGCUACAG.CUCAUGUAGUG.A.......CACAC.................................................
Ga0157593_1101573/244-147     ................................................................................................AAA...................................................
Ga0184597_102941/191-111      ................................................................................................CC....................................................
Ga0184597_115944/155-80       ................................................................................................A.....................................................
Ga0265742_1051722/222-295     ................................................................................................A.....................................................
Ga0180107_1229893/426-318     ..............................GGU...GGUUAGA.UCA.................................................AGUG..................................................
Ga0242654_11312609/202-83     .......................................................AGU.A.GU.................................UUGAAAUA..............................................
Ga0187793_1464320/616-535     ................................................................................................UAA...................................................
Ga0187794_1206392/88-6        ................................................................................................UAA...................................................
Ga0316034_129624/151-284      ......................................UAAGC.ACU..A.....GGC......................................CGAAGCCUGUCUUCACAGACUGUGCUUCAAAGGUGUGC................
Ga0184576_119948/531-664      ......................................UAAGC.ACU..A.....GGC...C.....................GAAG.CC..UGUCUUCAUAGACUGUGCUUCAAAGGUGUGC...........................
Ga0184568_109070/428-286      ................................................................................................AAGGGUUGUUGGGGCUACGAGGAGACAUGA........................
Ga0265756_100717/910-1050     .......................................................GUC.C.U..................................AGGGAACUCUUAAAACAAGAGCAGAGGGUGAUACCUCACUGUCA..........
Ga0179955_1195671/481-563     ................................................................................................AUAAU.................................................
Ga0184579_120620/502-584      ................................................................................................UCUGAG................................................
Ga0127502_10711632/191-294    ................................G...CC..........................................................UGGGGAAGUCCCG.........................................
Ga0310119_125223/193-319      .......................................................GCG.A.U..................................UGACG.................................................
Ga0307482_1064439/425-316     ..............................CAU...CG..................GA.C.C..................................AAGU..................................................
Ga0307484_102002/698-552      ................................................................................................GGGCGAACCACUUUUCCGGCUUCGAGGCGUGUG.....................
Ga0265741_107029/232-320      ................................................................................................ACU...................................................
Ga0247515_120448/91-178       ................................................................................................UAACU.................................................
Ga0265759_103410/556-638      ......................................................................................................................................................
Ga0247534_101509/435-523      ................................................................................................ACU...................................................
Ga0310102_119240/132-46       ................................................................................................AC....................................................
Ga0265742_1084621/112-29      ................................................................................................U.....................................................
Ga0310114_135156/99-185       ................................................................................................U.....................................................
Ga0265752_102595/694-607      ................................................................................................CU....................................................
Ga0265756_105276/677-589      ................................................................................................ACU...................................................
Ga0153880_1339022/280-371     ................................................................................................ACU...................................................
Ga0247518_120275/202-289      ................................................................................................UAACU.................................................
Ga0265752_100023/1618-1523    ..............................GGG...CU.................AGC.A.G..................................U.....................................................
Ga0307484_102966/353-250      .......................................................GUG.G.U...........................U.UCGCCUAGU..................................................
Ga0265752_101001/521-687      ......................................AAAGC.UCU..A.....GGC.A.UGGCCAGA...AGUGGGACCGUAAAC.CC.AAGCCCACGU.................................................
Ga0184593_121910/718-622      ........................................................U.......................................UUCUCCUAAA.AAG.AG.....................................
Ga0184602_103319/96-192       ..............................GAA...AG..........................................................AGAU..................................................
Ga0247528_108722/131-33       ..............................GAA...AG..........................................................AGAU..................................................
Ga0265757_143567/145-70       ................................................................................................G.....................................................
Ga0184592_121084/378-271      .......................................................UGG.A.UUACACG............................AAUAU.................................................
Ga0316037_172473/89-196       .......................................................UGG.A.UUACACG............................AAUAU.................................................
Ga0307921_1011071/674-791     .......................................................GUC.U.C..................................ACAACACAUGAUCUAUGUG...................................
Ga0265744_124024/357-288      ................................................................................................AU....................................................
Ga0307482_1000291/592-698     ................................................................................................AAGUGGCUUAAA..........................................
Ga0184568_108869/934-1072     ..............................A.................................................................GUCUAAA.............................................
Ga0138276_1121602/329-410     ................................................................................................CAACC.................................................
Ga0138278_1041650/183-102     ................................................................................................GAC...................................................
Ga0138277_1137481/570-489     ................................................................................................AAGA..................................................
Ga0138278_1041788/293-378     ................................................................................................AACA..................................................
Ga0138275_1223952/371-283     ................................................................................................AACA..................................................
Ga0138277_1142657/273-361     ................................................................................................AACA..................................................
Ga0307482_1202605/45-154      ..............................GAG...AG..........................................................UAGCCUAAGC.AGG.UG.....................................
Ga0180107_1017343/1163-1243   ................................................................................................UUAUA.................................................
Ga0157560_1170105/128-245     .......................................................GUC.U.U..................................UCGAUAGU..............................................
Ga0316030_137115/325-257      ................................................................................................UUU...................................................
Ga0316046_103474/576-502      ................................................................................................AA....................................................
Ga0265754_1141843/290-185     .......................................GUGC.GUA..A.....AGC.A.G..................................UGCCAA................................................
Ga0222729_1080556/362-213     .......................................................GCC.U.A..................................CUCAUG................................................
Ga0265740_1020297/298-150     ............................................GCA..U.....ACU.U.GAA...........AGCUCGAGAUUU.UU.UACUCAAAAGCUGAA.UAGAUG.....................................
Ga0157569_1080558/455-605     ..............................GAU...UG.................AGA.U.U..................................CAAUUUCAAUCAAA.CA.....................................
Ga0307484_101264/723-941      ACCGU...........................C...UC.................GCU.C.A..................................AAGAG.................................................
Ga0213850_1513954/279-196     ................................................................................................AAU...................................................
Ga0316053_117700/109-179      ................................................................................................CC....................................................
Ga0184582_101997/109-1        ........................................AGA.ACA..UG...CGGU.U.C................................CCCUAUCCUUUC.A..........................................
Ga0184602_109114/416-317      ................................................................................................A.....................................................
Ga0153880_1354682/355-454     ................................................................................................A.....................................................
Ga0184602_110257/415-316      ................................................................................................UAGA..................................................
Ga0307484_149222/136-234      ......................................CAAGA.CAU........GGU......................................UAGCA.................................................
Ga0184572_111224/187-319      ..............................CAC...CGCGUGC.AAA.................................................UGCGGGAGUAC...........................................
Ga0138278_1022898/219-127     .......................................................GGU.C....................................AUUCAAAUA.............................................
Ga0184577_108791/182-295      .......................................................CCU.C.A..................................GGCAAG................................................
Ga0265748_107452/280-387      ......................................AAAGG.GAG..U..............................................GUCAAGCGACUA..........................................
Ga0180107_1164033/560-431     .......................................................UGC...............GUUCACACCUUGGG.GU.GGAUCUUAC..................................................
Ga0032354_1086862/688-579     ..............................UGU.....................................................................................................................
Ga0265751_100560/274-382      ..............................GAC...GG..........................................................AUAUACCAAG.UGG.UG.....................................
Ga0180107_1045142/740-541     ..............................AGU...CCAUGGG.UAU........GC.......................................AACCAGAUGCUGCAAGUGUCCUGUCUUUGUUCCCGUAGCCCUUUAUACAGGGUC
Ga0213850_1296465/305-234     ................................................................................................UU....................................................
Ga0265757_111747/356-286      ................................................................................................UU....................................................
Ga0247553_104889/634-563      ................................................................................................UU....................................................
Ga0265751_102572/231-125      ................................C...CA.................GAU.C.C..................................AAGU..................................................
Ga0265758_100017/4028-4134    ................................C...CA.................GAU.C.C..................................AAGU..................................................
Ga0180107_1307934/226-315     ................................................................................................AGUCAGU...............................................
Ga0184572_112077/359-259      ................................................................................................CUUUCGGGGAUG..........................................
Ga0213850_1371453/186-298     ................................................................................................UCCUUG................................................
Ga0213850_1469439/119-231     ................................................................................................UCCUU.................................................
Ga0247535_113123/229-119      ......................................................................................................................................................
Ga0316036_112404/482-376      ................................................................................................UA....................................................
Ga0179943_1118543/263-176     ................................................................................................GGAA..................................................
Ga0180108_1207316/715-601     ................................................................................................GUUUGACCUAGUAGUGU.....................................
Ga0184578_120646/150-246      .......................................................AUU.C.U..................................AUGGA.................................................

                              ..............................>>>...>>.................<<<.<.<<<<<<<<<<<<<<<<<<<<<<<<<<.<<.<<<<<......................................................
                              ......................................................................................................................................................
                              ------------------------------------------------------------------------------------------------●○○○--------------------------------------------------
```

```
Ga0127503_10678853/419-333      ................................................................................................CAA...................................................
Ga0307919_1065410/76-162        ................................................................................................UAACGA................................................
Ga0180108_1209503/1932-2029     .......................................................CAG.A.C..................................AAGC..................................................
Ga0180108_1022680/3902-3998     .......................................................CGG.U.U..................................AGC...................................................
Ga0316031_107021/319-214        ......................................................................................................................................................
Ga0247527_116942/161-88         ................................................................................................UGCA..................................................
Ga0307484_100434/1330-1210      ................................................................................................CACGCGCCUGGAA.........................................
Ga0307482_1160392/324-400       ................................................................................................G.....................................................
Ga0316033_105623/339-253        ................................................................................................U.....................................................
Ga0265754_1037910/322-179       ...............................AG...C...........................................................GUGAUGGCUUAGCGGUGGAAUCACUUCCGCGCUGGCUUCAAAGGCG........
Ga0307482_1048753/383-528       ......................................GAA.......................................................GCGCUAGGCAGUUGCCCGUUUCUGUGGGGUUUUACGCCACAACGGCUUCGAUGC
Ga0265752_102479/223-81         .......................................................AAC.A.U..................................CAAUUGAGGA............................................
Ga0265746_1084801/20-158        .......................................................AAC.A.U..................................CAAUUGAGGA............................................
Ga0265756_107066/520-660        .......................................................AAC.A.U..................................CAAUUGAGGA............................................
Ga0265742_1022009/340-200       .......................................................AAC.A.U..................................CAAUUGAGGA............................................
Ga0265748_106902/213-353        .......................................................AAC.A.U..................................CAAUUGAGGA............................................
Ga0180052_116799/419-501        ................................................................................................UAAUA.................................................
Ga0115594_1106220/441-334       .............................................CA..C.....GAC.G.C..................................CAAUC.................................................
Ga0247534_117029/65-195         .......................................................CGA.......ACCU...........................CUCGAUAAAA.AGA.GA.....................................
JGI12272J11983_1072695/40-150   .......................................................AUC.C.GU......GUGCGU.....................AUA...................................................
Ga0247552_100007/4311-4420      ......................................CAUGG.AGA..U.....AGC.C.C..................................AAGU..................................................
Ga0184596_107732/313-205        ......................................CAUGG.AGA..U.....AGC.C.C..................................AAGU..................................................
Ga0247542_100007/609-500        ......................................CAUGG.AGA..U.....AGC.C.C..................................AAGU..................................................
Ga0247534_100047/1899-2008      ......................................CAUGG.AGA..U.....AAC.C.C..................................AAGU..................................................
Ga0184598_129111/512-403        ......................................CAUGG.AGA..U.....AGC.C.C..................................AAGU..................................................
Ga0184597_128933/573-464        ......................................CAUGG.AGA..U.....AGC.C.C..................................AAGU..................................................
Ga0184582_108821/1050-1159      ......................................CAUGG.AGA..U.....AGC.C.C..................................AAGU..................................................
Ga0247541_100010/3268-3377      ......................................CAUGG.AGA..U.....AGC.C.C..................................AAGU..................................................
Ga0184580_121439/573-464        ......................................CAUGG.AGA..U.....AGC.C.C..................................AAGU..................................................
Ga0265756_101275/650-541        ......................................CAUGG.AGA..U.....AGC.C.C..................................AAGU..................................................
Ga0184575_101094/586-477        ......................................CAUGG.AGA..U.....AGC.C.C..................................AAGU..................................................
Ga0184594_103311/207-316        ......................................CAUGG.AGA..U.....AGC.C.C..................................AAGU..................................................
Ga0247554_100006/1592-1485      ......................................CAUGG.AGA..U.....AGC.C.C..................................AAGU..................................................
Ga0247522_108409/684-554        .......................................................GCA.C.UAGGCGAGGUUG.CGGA..................AAGAC.................................................
Ga0247534_100003/3147-3245      ......................................CAAGA.U.A........GAG.U.U...................................AGC..................................................
Ga0265751_100393/1594-1698      ..............................ACA...CA.................AUC......................................CAAGC.......GG.U......................................
Ga0265751_124807/392-283        ..............................AUG...CA...........................................................UAUGCCAAG.UGG.UG.....................................
Ga0310107_144222/327-436        ..............................AUG...CAUA.............................................................UGCCAAG.UGG.UG.....................................
Ga0265743_103007/946-842        ......................................UUAGC.AAU.................................................CCCAAGCGGGU...........................................
Ga0316038_107364/55-195         .........................................................U.U.UAUUCGAUUCGAAUGAAC.................AUUU..................................................
Ga0184593_123391/157-297        .........................................................U.U.UAUUCGAUUCGAAUGAAC.................AUUU..................................................
Ga0222728_1003482/1262-1107     ..............................GCC...CGACAGA.GGU........AG..................UGUAGUGGCCUU.CC.UG...AUUGUGUAAC.AAC.UA.....................................
Ga0242654_10701525/53-296       ..............................AGU...CCAUGGG.UAU........AGC.G.GUGUU..............................AUGUGCCACGUCAAUCCACUUGACUGUGUGUUGUCCUAUUGUCACUUUGUGACU
Ga0242654_11172691/285-48       ..............................AGU...CCAUGGG.UAU........AGC.G.G..................................UGUUCUGUGCCACGUCAAUCCACUUGACUGUAUGGUUCCUAUCGUCACUACGUG
Ga0316038_100042/1676-1569      ..............................C.................................................................GUGGAAUCCCAAGCGGGU....................................
Ga0307921_1043977/91-164        ...................................................................................................UUUAAAUAA..........................................
Ga0079039_1293848/95-22         ................................................................................................CA....................................................
Ga0247534_101560/228-122        ..............................GAG...C...........................................................UAGCCAAGCGGUG.........................................
Ga0153880_1379927/66-144        ................................................................................................GAAG..................................................
Ga0153880_1391790/62-140        ................................................................................................UCAAG.................................................
Ga0307482_1048660/422-625       ................................................................................................GCUAGAACCGAGUGAUUUCGGACUGGUCCGCGCAGGUCUGUCACCUACCAUGAC
Ga0180108_1022680/4248-4153     .......................................................GGA.G....................................CAGCG.................................................
Ga0247516_125012/316-428        ..............................GGU...UCAAAG......................................................GUGUG.................................................
Ga0242652_1038498/365-470       ...............................AC...CGUUAGG.CUU..U.....GG.......................................GAGACCAAA.............................................
Ga0210323_1050281/92-187        ................................................................................................AUACCAAGCGGUAAAUG.....................................
Ga0224712_10542545/154-227      ................................................................................................UC....................................................
Ga0184602_130775/1275-1431      ..............................AGC...CUCAAGG.UC..................................................CU....................................................
Ga0206355_1311803/144-334       ..............................GAC...U...........................................................ACAAUUGGAACGUUAGUCCUG.................................
Ga0310119_152175/242-389        ......................................GAAGC.CUA..A.....AG..A.C..................................CUCGG.................................................
Ga0255059_10691632/166-76       .......................................................AGA.G....................................AUGAG.................................................
Ga0255062_11230321/200-110      .......................................................GAG.A....................................UGAGC.................................................
Ga0265743_143797/241-325        ..............................ACU...CU..........................................................CUGAC.................................................
Ga0247534_117055/74-212         ..............................GGU...CG..........................................................AGAG..................................................
Ga0213850_1282564/267-350       ..............................GGG...UC................................................................................................................
Ga0315826_123252/262-347        ..............................GGG...UC................................................................................................................
Ga0315817_133437/498-413        ..............................GGG...UC................................................................................................................
Ga0184577_116723/415-311        ................................................................................................UCAACCGAUGCCA.........................................
Ga0184576_109717/432-536        ................................................................................................CCGAUUCCA.............................................
Ga0184572_113323/230-300        ................................................................................................UU....................................................
Ga0179935_1139075/165-78        ................................................................................................A.....................................................
Ga0179944_1246019/249-162       .......................................................ACU......................................UGGA..................................................
Ga0213850_1307525/101-186       ................................................................................................CA....................................................
Ga0222728_1062510/166-66        ................................................................................................UCAACUCAAA.UAA.U......................................
Ga0265743_101198/448-619        ..............................AAU...UCCAAGG.UCA........GU..G.GGUCA.GAAAUAUAGCCAGUGGCAAU.A.......CUCCGUGUUCC...........................................
Ga0180108_1209503/2438-2343     .......................................................GGG......................................UAGUA.................................................
Ga0184583_103998/587-740        ......................................GAAGC.GCU..A.....GG.....CAGAUGC.......CCGUUUCAACC.CA.UCUCCCCAU..................................................
Ga0184595_100702/414-262        ......................................GAAGC.GCU..A.....GGC.G.AA...UGC.......CCGUUUCAACC.CG.UCUCCCCAU..................................................
Ga0184592_125105/67-219         ......................................GAAGC.GCU..A.....GGC.G.A...AUGC.......CCGUUUCAACC.CG.UCUCCUCAU..................................................
Ga0224712_10002011/1564-1454    ..............................GAU...CU.................AGU.C.C..................................CAAGU.................................................
Ga0316049_114344/181-288        ................................................................................................CGGAUCCAAGGCU.........................................
Ga0184576_110594/6-113          .......................................................GC.....CCUUCGA..........................UACGGA.................................................
Ga0316037_112281/325-219        .......................................................GAC.C.UUCGA..............................UCGGA.................................................
Ga0316046_116376/278-384        .......................................................AAC.C.UUCGAU.............................ACGG..................................................
Ga0247534_102735/124-18         .......................................................AGC.C.UUCGAU.............................ACGG..................................................
Ga0247540_102569/210-104        .......................................................AGC.C.UUCGAU.............................ACGG..................................................
Ga0247529_116306/255-362        .......................................................UGA.C.CUUCGAU............................ACGG..................................................
Ga0184568_104528/922-1000       ................................................................................................CAAA..................................................
Ga0184578_112031/156-44         .......................................................GCC......................................UUUAGU................................................
Ga0247517_121471/387-499        .......................................................GCC......................................UUUAGU................................................

                                ..............................>>>...>>.................<<<.<.<<<<<<<<<<<<<<<<<<<<<<<<<<.<<.<<<<<......................................................
                                ......................................................................................................................................................
```

```
Ga0184569_108885/200-88      .......................................................GCC......................................UUAAUU................................................
Ga0247521_112559/317-205     .......................................................GCC......................................UUAAUU................................................
Ga0247539_101233/414-526     .......................................................GCC......................................UUAAUU................................................
Ga0265756_100485/1108-1000   ......................................CUGGU.CAU........AUC.C.U..................................CAGUCGUUAAAG..........................................
Ga0265751_100417/1480-1588   ......................................CUGGU.CAU........AUC.C.U..................................CAGUCGUUAAAG..........................................
Ga0213850_1295854/263-370    ................................................................................................GGU...................................................
Ga0307919_1029784/253-144    ................................................................................................U.....................................................
Ga0307921_1042549/343-452    ................................................................................................U.....................................................
Ga0307484_102966/727-825     .......................................................GCU.U.C..................................AAGCGAA...............................................
Ga0184588_131258/156-253     ..............................................C..U.....UUG.G.CCAUC..............................AUCA..................................................
Ga0224712_10010317/1973-2082 ..............................AUC...CC..........................................................AAUCUCAAGC.GAG.UA.....................................
Ga0224712_10601975/393-502   ................................C...CC..........................................................AAUCUCAAGC.GAG.UA.....................................
Ga0222728_1000118/2272-2196  ......................................................................................................................................................
Ga0115594_1200134/154-31     ......................................CAUGC.CCU..U.....GCC.U....................................UUCA..................................................
Ga0115595_1153837/320-197    ................................................................................................UUUCAGG...............................................
Ga0255282_1067513/33-105     ................................................................................................GG....................................................
Ga0242647_1005934/451-617    ......................................ACAGA.CCU..UGGUAAGGC.C.GGG....UAAGG........UGUCAC..U.UGUACUCCAC.................................................
Ga0307482_1449537/408-223    ..............................AGA...CC.AGGA.AUG..A.....GGG....UCUU.UAGGUGAACCGUGCGACCUG.CUUAGGUCCCAGU.................................................
Ga0115594_1022316/346-239    .......................................................GUG.C....................................GACGCCAAAC............................................
Ga0115595_1072788/334-441    .......................................................GUG.C....................................GACGCCAAAC............................................
Ga0184568_108419/50-147      .......................................................ACC.A.U..................................AAGC..................................................
Ga0247534_100007/116-225     ................................................................................................UUUCAAGCGAAUA.........................................
Ga0184569_109084/220-111     ................................................................................................GAAUUUCAAGCGAAUA......................................
Ga0316033_100036/207-98      ..............................AGG...AG..........................................................AAUUUCAAGCGAAU........................................
Ga0184578_112042/249-140     ..............................AGG...AG..........................................................AAUUUCAAGCGAAU........................................
Ga0184594_123034/256-94      ................................................................................................GCAC..................................................
Ga0256798_1305521/221-305    ................................................................................................GAAAGCCAAC.AAG.UA.....................................
Ga0184595_116681/414-266     .......................................................GCC.G.UAGCCCGUGUCAUC.............UA.GCCGCUUGCG.................................................
Ga0184568_105547/129-239     ................................................................................................GUGACAUAGAAUGAAGUUGAUGCCU.............................
Ga0157559_1099797/360-209    ..............................ACA...UG...........A.....GGC.C.U..............GCGCUGGACGG.CU.CCGUACCAGU.................................................
Ga0157572_1089974/360-209    ..............................ACA...UG...........A.....GGC.C.U..............GCGCUGGACGG.CU.CCGUACCAGU.................................................
Ga0316037_108094/19-160      ................................................................................................UUUACUCA..............................................
Ga0316038_107116/33-174      ................................................................................................UUUACUCA..............................................
Ga0210323_1072445/691-543    .............................................GU..A.....GCG.C.UAGCCUUG...........................CCAAGACUGCAUUCACCUCGGUGAAGAGGUUGCGGCUAAA..............
Ga0307482_1000707/704-773    ................................................................................................UG....................................................
Ga0079039_1671802/197-34     ..............................AGG...UGUA...............GU.......................................UUUAAGGCCCGUCCCGGGGACCAAACAGACCAGAUCUGUACUCCGAGG......
Ga0247533_103342/137-220     ................................................................................................AU....................................................
Ga0157565_1016893/233-334    ................................................................................................CA....................................................
Ga0180047_1087048/281-180    ................................................................................................CA....................................................
Ga0157593_1006201/239-338    ................................................................................................CAAA..................................................
Ga0157560_1010344/357-456    ................................................................................................CAAA..................................................
Ga0316049_154526/155-74      ................................................................................................UUCUUUG...............................................
Ga0316035_177575/224-142     .......................................................AAU.......................................UCUUUGAAUU...........................................
Ga0316033_101029/151-49      ......................................ACAGA.AGA........GAU.C.G..................................AAGC..................................................
Ga0206356_10600373/215-21    ......................................................................................................................................................
Ga0307482_1009758/562-669    ..............................AUG...GC......A..........AUC.C....................................CAAGCG................................................
Ga0213850_1245299/581-409    ................................................................................................UCAUGUCUGGCGUCCGUACAGUGCAAAUGAUGCACCUUGUUAAUGGUACUAAGA
Ga0180107_1134582/382-542    ..............................AGU...AG.CAGU.AUG..C.....UUC.C.ACU.....G...............CU.GU.AUGCCCCCCCU................................................
Ga0307484_100970/951-1026    ................................................................................................AC....................................................
Ga0307919_1034281/527-451    ................................................................................................U.....................................................
Ga0307921_1020694/406-483    ................................................................................................UUUAU.................................................
Ga0316033_105623/3-98        ......................................................................................................................................................
Ga0265743_111644/592-512     ................................................................................................GA....................................................
Ga0265755_108708/373-453     ................................................................................................AA....................................................
Ga0316038_121313/184-264     ................................................................................................UGAA..................................................
Ga0265756_109326/116-196     ................................................................................................UGA...................................................
Ga0265759_104327/339-243     ................................................................................................GUUGG.................................................
Ga0316035_144545/195-301     ................................................................................................GUC...................................................
Ga0265743_108998/150-253     ................................................................................................UGCGAGAGCAAUCCAAGCGGU.................................
Ga0153880_1067318/492-566    ................................................................................................UC....................................................
Ga0180044_1047678/444-368    ................................................................................................C.....................................................
Ga0157564_1065218/271-353    ................................................................................................UC....................................................
Ga0180044_1069332/269-187    ................................................................................................C.....................................................
Ga0242647_1014694/423-336    .......................................................GCA.G.C...................................AUU..................................................
Ga0180108_1143860/199-116    .......................................................AGA.U.U..................................AGGCACUGUCA...........................................
Ga0265744_100073/1235-1401   ..............................AUU...CCACGGG.UAU........GG.......................................ACCUACAGCCAAUGGCCCGAGGGGCGACCAGUCCCCCGAUUUCCGCCAGUGCUC
Ga0307482_1054608/326-205    ................................................................................................C.....................................................
Ga0242646_1008525/229-119    ................................................................................................C.....................................................
Ga0153880_1260556/441-589    .......................................................GCA.C.UAC...............................CAUCGUGU...............................................
Ga0127503_10814644/496-352   ................................C...CGUU..U.UCU......................................UG.GA.CCACCCUCG..................................................
Ga0326631_105052/171-77      ..............................ACC...CA
Ga0307920_126542/349-261     ................................................................................................AAGGUGAAUA.AAU.UC.....................................
Ga0247533_100162/1643-1535   ..............................GCC...UA.................AUC......................................CAAGC.................................................
Ga0316047_100015/734-626     ..............................GCU...CA.................AUC......................................CAAGC.................................................
Ga0265743_101537/1064-984    ................................................................................................GCUC..................................................
Ga0316030_100208/805-700     ................................G...CGAAAGC.AAU........GUC......................................AAGC..................................................
Ga0316050_103193/566-460     ................................G...UAAAAGC.AAU........GUC......................................AAGU..................................................
Ga0184601_113231/247-149     ..............................ACC...CC..........................................................CCAAUG................................................
Ga0307350_148584/139-224     ................................................................................................A.....................................................
Ga0307482_1018929/1182-1081  ................................................................................................GCUGUGUUCG............................................
Ga0307482_1000262/4800-4699  ................................................................................................CGCUGUGUUCG...........................................
Ga0316047_100860/1040-888    ......................................................................................................................................................
Ga0242654_10068518/113-218   ................................................................................................CAUGGGGUGUCGGUAAAGCCCUU...............................
Ga0222729_1010500/11-117     ..............................UGU...CG..........................................................AUAAAGCCCUU...........................................
Ga0307484_101172/602-495     ................................................................................................UAAAAGCCCUU...........................................
Ga0242647_1002406/915-1021   ................................................................................................AGUAAAGCCCUU..........................................
Ga0307483_1000720/1178-1064  ................................................................................................AGACUGUAACCC..........................................
Ga0307921_1022947/450-369    ................................................................................................CCAUA.................................................
Ga0153880_1432604/289-395    .......................................................AGA.A.CCACGC.............................UAGGUA................................................

                             ..............................>>>...>>.................<<<.<.<<<<<<<<<<<<<<<<<<<<<<<<<<.<<.<<<<<......................................................
                             ......................................................................................................................................................
                             ------------------------------------------------------------------------------------------------●○○○----------------------------------------------
```

```
Ga0247533__101442/511-432     ................................................................................................AAU...................................................
Ga0242647__1002473/759-521    ..............................AGU...CCAUGGG.UAU..A.....GGC.U.G..................................GUAAUGUGUCACGUCCAAAGCCAAUUGAACGUAUGUCUCCAGUGGUCCGCGAGG
Ga0256352__1087084/23-118     ................................................................................................UAAU..................................................
Ga0157562__1157806/189-74     ................................................................................................AAU...................................................
Ga0153880__1457361/236-121    ................................................................................................AAAUAA................................................
Ga0157559__1128929/201-87     ................................................................................................UUAAUA................................................
Ga0157566__1012736/497-384    ................................................................................................AUAAU.................................................
Ga0157579__1155702/323-436    ................................................................................................AAU...................................................
Ga0316036__116159/257-326     ................................................................................................CG....................................................
Ga0180047__1032966/152-37     ................................................................................................AAU...................................................
Ga0184587__125934/78-152      ................................................................................................UAU...................................................
Ga0179934__1155244/114-26     ................................................................................................AAGGCAGAAG.C..........................................
Ga0307482__1623775/241-347    ..............................GA....CA...........U.....GGG.U....................................UAGC..................................................
Ga0307484__101582/1018-1124   ..............................GAC...AG.................AGG.U.C..................................AGC...................................................
Ga0307484__111921/444-550     ..............................GGC...AG.................AGG.U.C..................................AGC...................................................
Ga0184587__113157/56-168      ......................................GAAGC.CUA........AAG.A.C..................................CUCAG.................................................
Ga0255228__1272153/272-192    ................................................................................................A.....................................................
Ga0153880__1325448/483-575    ................................................................................................CUA...................................................
Ga0157565__1130498/91-183     ................................................................................................CUA...................................................
Ga0265758__129208/41-120      ................................................................................................ACG...................................................
Ga0265756__146329/172-94      ................................................................................................C.....................................................
Ga0115594__1180137/311-161    ..............................AGU...GC.................GGU.GUGAGGCCGCUCGCGGUGA..................GCUUCGCCAGAAGCGU......................................
Ga0265744__100861/398-567     ..............................AAU...UCCAAGG.UCA..U.....GGG.U.CAGAAU.............................AUCGCCGAGUGGCAAUACCCUGUGUAUCCCUCGAGCUCU...............
Ga0180107__1285918/13-113     .......................................................GU.......................................AACACGUAAG.UGA.UA.....................................
Ga0247519__103291/1381-1460   .......................................................UGA.C....................................UUAU..................................................
Ga0184603__106561/141-62      ................................................................................................CUUAUGUCA.............................................
Ga0247551__100996/477-556     ................................................................................................UAUGUCA...............................................
Ga0184590__126350/300-380     .......................................................GAC......................................UUUAUG................................................
Ga0265756__131580/159-58      ................................................................................................UAAUUCAAAU.U..........................................
Ga0307482__1000325/24-131     ..............................AGA...CA...................................................U.AUACCAAGU..................................................
Ga0242646__1001086/737-571    ..............................AAU...CCUAAGG.UAU........GGC.......CAGG..UAGUAUGCUUGGAGAG.CC.AUUGAUUCU..................................................
Ga0265747__132694/181-66      ................................................................................................ACG...................................................
Ga0157593__1023702/238-314    ................................................................................................AUCAAA................................................
Ga0316053__103654/814-736     ................................................................................................AUCAAA................................................
Ga0247529__117158/253-331     ................................................................................................GCAAUCAAA.............................................
Ga0180107__1129325/244-60     ..............................AGA...CUCAGGC.AGC........GUU.U.UCAGUU.............................AUGACUCGUUUGAUUUGGUCAUCGAUCAAAUCUUGCGAUUACAGUGCCAUUGAG
Ga0307482__1162833/232-148    ................................................................................................AAA...................................................
Ga0255267__1268184/91-184     ........................................UGU.AAU..U.....C....................................CACCACAAA.................................................
Ga0307919__1064641/225-73     ..............................GCC...GG.................CCA.C.AACU............CUAAGCAACA.CG.UUCCAUAAU..................................................
Ga0307921__1075885/188-36     ..............................GCC...GG.................CCA.C.AACU............CUAAGCAACA.CG.UUCCAUAGU..................................................
Ga0265742__1021317/385-459    ................................................................................................CCA...................................................
Ga0307483__1002304/791-713    ................................................................................................GUCGCUC...............................................
Ga0307919__1165008/203-116    ................................................................................................GG....................................................
Ga0210323__1093898/306-192    ................................................................................................AUGAAA................................................
Ga0265743__119695/104-184     ................................................................................................GGUA..................................................
Ga0310118__104706/1022-1118   ................................................................................................AGAUCAGA..............................................
Ga0307921__1005950/551-460    .......................................................GCA.U.U..................................UGCGUCUAUU............................................
Ga0242641__1014724/551-384    ..............................ACC...CCGAGGC.CAA........GUG.C.................UGCGAGUCCU.GU.GUGCGAACUG.................................................
Ga0242641__1002366/499-331    ..............................ACC...CCGAGGC.CAA........GUGCU.GCGAGUCC..UGUGUGC.................GGAACUG................................................
Ga0242657__1007384/290-215    ................................................................................................UC....................................................
Ga0265757__113513/277-362     ................................................................................................CC....................................................
Ga0242646__1001007/790-609    ..............................AAU...UCCAAGA.GCU..C.....GGU.C.GAGGCCAUAUA.........................UGAUGUUUACCCGGGUGAUUCCGGACCUC.........................
Ga0316034__107719/314-205     ..............................GCG...UA.................AUC.C....................................CAAGC.................................................
Ga0316039__119781/347-457     ..............................GCA...UA.................AUC.C....................................CAAGC.................................................
Ga0255295__1078742/236-408    ......................................GAAGA.CUU........UGU.A.G........................................................................................
Ga0184603__150346/629-518     ..............................ACG...UA.................AUC.C....................................CAAGC.................................................
Ga0180106__1187148/166-89     ................................................................................................CA....................................................
Ga0307482__1417159/374-296    ................................................................................................UUG...................................................
Ga0222728__1000095/4027-4133  ................................................................................................AAGCGGUA..............................................
Ga0316030__105840/623-504     ..............................................................GGGGGGUUUGCAGAACAUUGA.............CGUG..................................................
Ga0316033__110175/528-409     ......................................GCAGA.ACA.................................................UUGACGUGUCAAAUG.......................................
Ga0326631__124620/105-175     ................................................................................................AA....................................................
Ga0206356__11590518/224-68    ......................................CUGGA.UAA..A.....UCG.U.G..................................UAAAG..ACG.UAC.UA.....................................
Ga0213850__1246156/171-47     .......................................................GGG.A.G..................................UUGCUGAUUAGGCAGACACCCAUGCCUGGCAGCCUC..................
Ga0184599__140450/516-588     ................................................................................................UUU...................................................
Ga0206356__11002907/329-148   .......................................................CCC.U.C..................................GUGUA.................................................
Ga0307482__1001469/2898-2828  ................................................................................................UAAG..................................................
Ga0157570__1089798/68-159     ................................................................................................ACGA..................................................
Ga0127503__11028958/51-130    ................................................................................................AAUCU.................................................
Ga0157560__1126722/83-169     ................................................................................................UGAUGA................................................
Ga0138277__1028338/433-516    ................................................................................................AGAAAUAUAGCAAG........................................
Ga0265742__1001214/55-127     ................................................................................................AGCA..................................................
Ga0115594__1134035/652-542    .......................................................GUG.C.U..................................CCCCA.................................................
Ga0115594__1212468/8-118      .......................................................GUG.C.U..................................CCCCA.................................................
Ga0247533__100438/1040-888    ......................................................................................................................................................
Ga0326631__117973/181-293     .......................................................GCA.U.C..................................CUCUCACUGUUCGCAGU.....................................
Ga0184600__117644/32-108      ................................................................................................AAUAC.................................................
Ga0184584__133603/317-393     ................................................................................................GAAUAC................................................
Ga0316052__100527/264-188     ................................................................................................GAAUAC................................................
Ga0184580__125947/153-229     ................................................................................................GAAUAC................................................
Ga0247524__105844/561-637     .......................................................GU.......................................GAAU..................................................
Ga0184580__109821/480-551     ................................................................................................GAU...................................................
Ga0184598__107135/96-25       ................................................................................................UCUGAU................................................
Ga0316051__1015080/488-559    ................................................................................................UCUGAUC...............................................
Ga0247524__121352/374-303     ................................................................................................AUCU..................................................
Ga0179934__1197833/207-115    .......................................................UUG.G.CCAUC..............................AUCA..................................................
Ga0316049__129960/371-262     ................................................................................................A.....................................................
Ga0265747__125301/405-306     ................................................................................................UAAAAC................................................
Ga0157570__1034241/151-229    ................................................................................................UAC...................................................

                              ..............................>>>...>>.................<<<.<.<<<<<<<<<<<<<<<<<<<<<<<<<<.<<.<<<<<......................................................
                              ......................................................................................................................................................
                              ------------------------------------------------------------------------------------------------●○○○--------------------------------------------------
```

```
Ga0157571_1064089/212-134      ................................................................................................AC....................................................
Ga0242652_1041630/308-377      ................................................................................................AA....................................................
Ga0307480_1006257/684-599      .......................................................GGC.A.C..................................CGUUUU................................................
Ga0310119_142698/346-430       ................................................................................................GCUG..................................................
Ga0307482_1074296/478-354      ..............................UAU...GA.................GUG......................................AACAG.AAAC.AAA.AA.....................................
Ga0307483_1005049/26-150       ..............................UAU...GA.................GUG......................................AACAG.AAAC.AAA.AA.....................................
Ga0265757_127696/205-308       ................................................................................................UAUUU.................................................
Ga0316029_101202/440-551       ...............................UA...CGGUGGU.ACG..A.....AGG.U.C..................................AAGC..................................................
Ga0307482_1134687/287-181      .......................................................GGA.U.UCACCU............................UAAGU..................................................
Ga0307484_104189/399-292       .......................................................UGG...........................AU.UC.AUCUCAAGU..................................................
Ga0307921_1050271/251-412      ..............................AGU...AG.................GUA.U.A..................................CAAAGCAACACGUGCACACGGAACCAAGACUUUCGUCUAAUCACGGCACGGCCU
Ga0184580_102899/442-582       ................................................................................................GUCGCAAGACAUAUCUCGAAAGAGAUC...........................
Ga0316029_136733/88-8          ................................................................................................UGCCGA................................................
Ga0180107_1174136/218-102      .......................................................ACC.A.GAAGA..............................GACAAAGGACGUAAGUCACGUGC...............................
Ga0179948_1254202/198-296      ................................................................................................ACGUACUAAU.A..........................................
Ga0265746_1140777/214-319      ................................................................................................AUU...................................................
Ga0265756_108694/382-277       ................................................................................................AUU...................................................
Ga0265758_129717/201-94        .......................................................AGC.C.GAA................................UCUAAU................................................
Ga0213850_1394829/253-105      ..............................AAU...AC.................GGC.C.G..........CCUGAAGCAGUACUA.C.......GCCA..................................................
Ga0184603_139562/141-280       ................................................................................................AA....................................................
Ga0157560_1100546/299-385      ................................................................................................AUAAUG................................................
Ga0129331_1494381/292-368      ................................................................................................AGUAUGGGAAA...........................................
Ga0265759_100473/818-988       ..............................AGC...CCAUGGA.ACA........GG..U.AUCUC.GGUUAAGUGUCAUCACACGG.C.......CUCGU.................................................
Ga0265746_1003835/1049-1219    ..............................AGC...CCAUGGA.ACA........GG..U.AUCUC.GGUUAAGUGUCAUCACACGG.C.......CUCGU.................................................
Ga0265756_100592/1458-1288     ..............................AGC...CCAUGGA.ACA........GG..U.AUCUC.GGUUAAGUGUCAUCACACAG.C.......CUCGU.................................................
Ga0247536_100773/637-807       ..............................AGC...CCAUGGA.ACA........GG..U.AUCUC.GGUUAAUGGUCAUCACACGG.C.......CUCGU.................................................
Ga0184578_101068/480-310       ..............................AGC...CCAUGGA.ACA........GG..U.AUCUC.GGUUAAUGGUCAUCACACGG.C.......CUCGU.................................................
Ga0247530_109035/489-319       ..............................AGC...CCAUGGA.ACA........GG..U.AUCUC.GGUUAAUGGUCAUCACACGG.C.......CUCGU.................................................
Ga0247513_128543/241-71        ..............................AGC...CCAUGGA.ACA........GG..U.AUCUC.GGUUAAUGGUCAUCACACGG.C.......CUCGU.................................................
Ga0184573_112336/323-153       ..............................AGC...CCAUGGA.ACA........GG..U.AUCUC.GGUUAAUGGUCAUCACACGG.C.......CUCGU.................................................
Ga0127502_10575752/515-449     ................................................................................................CA....................................................
Ga0180107_1033011/786-679      ..............................GAA...CU..................CU.U.C..................................CAAGC.................................................
Ga0222728_1380294/134-238      .......................................................UGG.A.UUGCGCG............................AACU..................................................
Ga0307484_107311/306-374       ................................................................................................UCCA..................................................
Ga0316046_107255/463-562       ................................................................................................UGCACUCAGAUUAGUG......................................
Ga0184570_114412/209-338       .......................................................GAU......................................UCGAA.................................................
Ga0242646_1000880/695-856      ..............................AAC...CCCAAGC.AAC..A.....AGG.U.GGCGAUG............................UUGUGACAAC.GCU.CA.....................................
Ga0316031_123456/415-346       ................................................................................................AAG...................................................
Ga0157630_1203478/203-99       ................................................................................................UA....................................................
Ga0247541_102707/328-184       ..............................AAC...UAGAC..............GC.........................CUGGU.AC.UCCUCUCAC..................................................
Ga0115594_1174394/642-729      ................................................................................................GUAAUG................................................
Ga0115594_1187657/870-960      ................................................................................................AUG...................................................
Ga0242646_1047905/92-182       ................................................................................................UCCGGCC...............................................
Ga0247541_101130/558-391       ..............................AGC...CCAAAGA.GCA........GGA.A.UC..UCGGUUAAGCGCC........A.GC.CUACGGCCU..................................................
Ga0307921_1062541/180-338      ..............................ACG...CUUUAGC.GCC..........A......................................CAACUCUAAGCAACACCUCCUAUCGGAGGGCCUAGCAGAUUUAAG.........
Ga0184572_105168/388-281       ..............................ACG...CA.................AUC.C....................................CAAGC.................................................
Ga0316036_127178/124-231       ..............................ACG...CA.................AUC.C....................................CAAGC.................................................
JGI12272J11983_1345183/364-258 ................................G...CAAU...............GCC......................................AAGC..................................................
Ga0247535_100068/719-828       ..............................GCG...UA.................AUC.C....................................CAAGC.................................................
Ga0222728_1000079/1192-1299    ................................G...CA.................AUC.C....................................CAAGU.................................................
Ga0081246_1201717/153-259      ................................G...CA.................AUC.C....................................CAAG..................................................
Ga0222728_1216887/51-157       ................................G...CA.................AUC.C....................................CAAG..................................................
Ga0157569_1073490/184-258      ................................................................................................GCA...................................................
Ga0180047_1061805/548-622      ................................................................................................GCA...................................................
Ga0206353_11976089/292-380     ................................................................................................GAUUCG................................................
Ga0247553_101094/709-629       ................................................................................................CGAUC.................................................
Ga0242641_1004288/32-112       ................................................................................................GUUCGCU...............................................
Ga0242641_1014474/336-257      ...........................................................C.G..................................UUCUU.................................................
Ga0126318_10199518/109-204     .......................................................CCA.U.C..................................UACG..................................................
Ga0307919_1109364/247-158      ................................................................................................AUCAA.................................................
Ga0180107_1307618/257-174      ................................................................................................UA....................................................
Ga0179937_1126086/216-325      ................................................................................................GC....................................................
Ga0265744_134092/134-214       ................................................................................................UC....................................................
Ga0307482_1015399/786-719      ................................................................................................AA....................................................
Ga0265751_100477/874-675       ................................................................................................AGGCCUUGGG............................................
Ga0265756_100433/485-286       ................................................................................................AGGCCUUGGG............................................
Ga0265743_100639/33-191        ................................................................................................AGGCCUUGGG............................................
Ga0184602_130623/1321-1100     ..............................AGG...CG......ACC..U.....GUC.U.UUGUACGU...........................UCAGCCCUUAGUUUGGGACGAGGAACUCGUCCUCCGAGACGCUCGAGGAGAACU
Ga0184597_126105/585-362       ..............................AAC...CGCAGGC.GAU..C....UGUC.U.UUGUACGU...........................UCAGCCCUUAGUUUGGGAAAGAGGAACUCUCCCCCGAGACGCUCGAGAAAAGAA
Ga0187793_1322799/72-151       ................................................................................................GAUG..................................................
Ga0316037_128286/253-347       ................................................................................................AUA...................................................
Ga0265758_109576/83-157        ................................................................................................UU....................................................
Ga0265751_100127/176-103       ................................................................................................CGAC..................................................
Ga0265758_108618/113-22        ................................................................................................C.....................................................
Ga0228708_1128185/228-344      .......................................................GGG.U.U........G.........................UUGAUCAAA.............................................
Ga0256309_1199458/363-444      ................................................................................................GA....................................................
Ga0256302_1090383/76-157       ................................................................................................A.....................................................
Ga0255224_1021381/13-113       ................................................................................................A.....................................................
Ga0255223_1087001/28-107       ................................................................................................GA....................................................
Ga0127502_11371108/362-252     ..............................GAG...GA.................AUC.C....................................CAAGU.................................................
Ga0222728_1000125/1315-1238    ................................................................................................UGAU..................................................
Ga0242654_10000586/1273-1196   ................................................................................................GUGU..................................................
Ga0180107_1218358/314-219      ................................................................................................CGAUGAA...............................................
Ga0307482_1020425/440-614      ..............................AAU...ACCAAGA.CAU..C.....AGC.U..CACUCAUAUGCG......................CCAGGACUCAAUACUCUGUGUUCUUCCUGCCC......................
Ga0179951_1211246/478-633      ................................................................................................AUGAAAGA..............................................
Ga0179950_1197742/286-441      ................................................................................................AUGAAAGA..............................................
Ga0129331_1058147/189-99       ................................................................................................UCGAAUG...............................................
Ga0265756_102054/286-395       ..............................GUA...CA.................AUU.C.G..................................AAGU..................................................
Ga0222728_1000128/4501-4432    ................................................................................................CA....................................................

                               ..............................>>>...>>.................<<<.<.<<<<<<<<<<<<<<<<<<<<<<<<<<.<<.<<<<<......................................................
                               ......................................................................................................................................................
                               ------------------------------------------------------------------------------------------------●○○○----------------------------------------------
```

```
Ga0265759_114856/370-262    ................................................................................................AGAAA.................................................
Ga0079039_1551950/237-315   ................................................................................................GAGAG.................................................
Ga0179943_1170285/153-222   ................................................................................................AUGG..................................................
Ga0213850_1119088/23-236    ................................................................................................UCUCUAAACCAGGAGGGUGGUAUAAUUACAACCAGCAAAGGUUGGUCACUCGUU
Ga0247533_103075/417-305    .......................................................GCC.C.U..................................UUAUAA................................................
Ga0247541_102964/56-168     .........................................................................................C.UCGCCCUUUAUCA..............................................
Ga0265755_105988/310-386    ................................................................................................GAU...................................................
Ga0157560_1026167/317-382   ................................................................................................GAA...................................................
Ga0115595_1093158/7-137     .....................................................................................GG.UC.CCAGCUAUC..................................................
Ga0256352_1101982/155-277   ................................................................................................GCUCGAGGA.............................................
Ga0256350_1100736/283-405   ................................................................................................GCUCGAGGA.............................................
Ga0213850_1360620/298-221   ................................................................................................AUA...................................................
Ga0081247_1167831/113-35    ................................................................................................A.....................................................
Ga0127503_10676578/136-67   ................................................................................................ACG...................................................
Ga0138283_1149501/173-59    .......................................................GUC.U.C.............................AAGCCGAGAAUUUAA.UCU.CA.....................................
Ga0265757_139522/177-70     ..............................ACA...CA.................AUC.C....................................CAAGC.................................................
Ga0265750_1013321/389-496   ..............................ACA...CA.................AUC.C....................................CAAGC.................................................
Ga0316029_116217/368-261    ..............................ACA...CA.................AUC.C....................................CAAGU.................................................
Ga0316047_100016/2681-2574  ..............................ACA...CA.................AUC.C....................................CAAGU.................................................
Ga0184569_112764/943-836    ..............................ACA...CA.................AUC.C....................................CAAGC.................................................
Ga0242652_1131915/134-203   ................................................................................................CU....................................................
Ga0307482_1020622/600-404   ..............................AUU...CCACGGG.UUA..U.....GG..C.CGUUAACAUUGUGUAAGACCAGUACC.AC.CGAUGCUCACGACAU.GUG.AA.....................................
Ga0265756_143657/133-55     ................................................................................................UCAGC.................................................
Ga0180107_1262812/3136-3066 ................................................................................................CA....................................................
Ga0265756_118952/425-318    ..............................GAA...CC..........................................................AUU...................................................
Ga0265757_113703/332-416    ................................................................................................CUAU..................................................
Ga0256332_1243244/48-162    ................................................................................................CAUUGUC...............................................
Ga0307919_1048575/242-90    ..............................GCG...UG.................GCC.A.CAACUC............UAAGCAAC.AC.GUUCCUAAU..................................................
Ga0265754_1081367/214-290   ................................................................................................GUG...................................................
Ga0184597_114231/79-11      ................................................................................................AAUCA.................................................
Ga0184582_129853/106-35     ................................................................................................CA....................................................
Ga0265744_136679/344-276    ................................................................................................GAU...................................................
Ga0326631_172366/284-207    ................................................................................................GCUAC.................................................
Ga0307921_1021783/160-261   .......................................................GCG.C.U..................................UAGAGGGAGA............................................
Ga0247554_113506/254-161    .......................................................GG.......................................UAGCA.................................................
Ga0179939_1229388/322-392   ................................................................................................CC....................................................
Ga0184568_100511/100-195    ................................................................................................CGGUGUG...............................................
Ga0247525_114510/199-318    ................................A...UG.................CAA.C.AGGACCUUCC.........................UUCAC.................................................
Ga0222728_1008526/24-283    ................................................................................................GACUUCGAGCGCAAGCAAGCAUCAUUCCACAUAAAAACCAUAAAAAAAGAUAGG
Ga0242654_10278372/461-362  ..............................AGA...GA................................................................................................................
Ga0184570_100371/69-155     ................................................................................................GGUGCA................................................
Ga0184597_120210/233-147    .......................................................GCA...C..................................AAACGG................................................
Ga0213850_1220553/385-463   ................................................................................................GUUAA.................................................
Ga0265745_1120044/54-129    ................................................................................................ACA...................................................
Ga0213850_1278242/600-506   ................................................................................................GGGGCG................................................
Ga0213850_1524759/286-380   ................................................................................................GGGGCG................................................
Ga0242657_1009512/1576-1466 ..............................GCG...UA.................AUU.C....................................CAAGU.................................................
Ga0307482_1430195/259-189   ................................................................................................AC....................................................
Ga0184590_129043/450-518    ................................................................................................A.....................................................
Ga0184578_132646/9-94       ................................................................................................C.....................................................
Ga0180107_1040049/1100-1010 ................................................................................................GCGAAUCAGU............................................
Ga0307484_103912/620-553    ................................................................................................UGA...................................................
Ga0307921_1037919/342-457   ................................................................................................GCGCCAUAC.............................................
Ga0307920_113994/654-539    .......................................................GGC......................................UUACGC................................................
Ga0316040_172648/178-66     .......................................................GGC.C....................................UUAC..................................................
Ga0184577_110850/246-358    .......................................................GGC.C....................................UUGCG.................................................
Ga0307920_113994/130-9      ................................C...C...........................................................AUAC..................................................
Ga0307919_1081825/191-74    ................................................................................................CUGUGAUAC.............................................
Ga0307921_1095896/178-61    ................................................................................................CUGUGAUAC.............................................
Ga0316031_113250/505-406    ................................................................................................AA....................................................
Ga0265744_118928/137-218    ................................................................................................GGGUGGAC..............................................
Ga0265749_106268/131-212    ................................................................................................GUGGAC................................................
Ga0157562_1160399/232-315   ................................................................................................GA....................................................
Ga0153880_1231581/116-241   .....................................................................................UG.GC.CCAUCCACGU.................................................
Ga0153880_1191383/455-340   ......................................GAGGU.AAA..................................................CGGUUGU..............................................
Ga0138282_1242808/325-475   ..............................................................CGUCUGC...AGU.....................AUUUCCAAAUGCGAGCAACUGUACAAU...........................
Ga0242654_10024002/836-915  ................................................................................................UAAGC.................................................
Ga0242646_1103331/248-318   ................................................................................................U.....................................................
Ga0081243_1208438/380-456   ................................................................................................AC....................................................
Ga0184600_147964/253-339    ................................................................................................AU....................................................
Ga0316036_107710/408-322    ................................................................................................AU....................................................
Ga0184601_135946/406-321    ................................................................................................UU....................................................
Ga0184589_121067/502-587    ................................................................................................UU....................................................
Ga0184598_138497/440-355    ................................................................................................UU....................................................
Ga0184580_113725/262-335    ................................................................................................AA....................................................
Ga0184584_115572/267-354    ...........................................................G.A..................................UUUAU.................................................
Ga0247539_100086/1763-1850  ...........................................................G.A..................................UUUAU.................................................
Ga0184579_105257/1735-1648  ...........................................................G.A..................................UUUAU.................................................
Ga0126318_10229384/609-688  ................................................................................................C.....................................................
Ga0307482_1000291/1027-950  ................................................................................................AC....................................................
Ga0316031_119808/254-182    ................................................................................................UGGC..................................................
Ga0307482_1023665/853-778   ................................................................................................UGG...................................................
Ga0180106_1297567/444-515   ................................................................................................CGU...................................................
Ga0307484_115660/213-145    ................................................................................................GC....................................................
Ga0307484_161007/136-39     ..............................CCC...CUCA...............GUG.G.A..................................GUUA..................................................
Ga0307484_177982/136-39     ..............................CCC...CUCA...............GUG.G.A..................................GUUA..................................................
Ga0307482_1033688/516-422   ..............................CCC...CCA................GUG.G.A..................................GUUA..................................................
Ga0265752_111922/418-343    ................................................................................................UGG...................................................
Ga0179939_1199779/67-205    ................................................................................................A.....................................................

                            ..............................>>>...>>.................<<<.<.<<<<<<<<<<<<<<<<<<<<<<<<<<.<<.<<<<<......................................................
                            ..............................xxx...xx.................yyy.y.yyyyyyyyyyyyyyyyyyyyyyyyyy.yy.yyyyy......................................................
                            ------------------------------------------------------------------------------------------------●○○○----------------------------------------------
```

```
Ga0307482_1023050/176-255 ................................................................................................U.....................................................
Ga0307482_1202605/470-395 ................................................................................................UGCG..................................................
Ga0242657_1476020/160-244 ................................................................................................UGA...................................................
Ga0247523_120120/325-403 .......................................................GUG......................................UAAUC.................................................
Ga0316035_179807/270-184 ................................................................................................U.....................................................
Ga0210323_1050507/418-532 ................................................................................................AUGAAA................................................
Ga0184571_112742/47-116 ................................................................................................GU....................................................
Ga0247538_100770/928-997 ................................................................................................U.....................................................
Ga0157560_1032840/250-177 ................................................................................................G.....................................................
Ga0247514_107497/1071-1140 ................................................................................................UACG..................................................
Ga0242646_1002014/11-270 ................................................................................................UACGUGCUUGACUUCGAGCGCAAGCAAGCAUCAUUCCACAUAAAAACCAUAAAA
Ga0222728_1008391/1281-1023 UAAAACAGAUAGGUAAAAUAUGUGUUAUCC..................................................................UUGAGAGACUUGGAUAGCAUG.................................
Ga0242646_1001395/255-509 ................................................................................................ACAACGACCCGCAAGGAGCUCUAGUCAGUGGUGACCAAGAAAUCACACAUGCGG
Ga0307482_1000256/3341-3276 ................................................................................................GUUU..................................................
Ga0242647_1006852/526-598 ................................................................................................U.....................................................
Ga0184580_111164/678-749 ................................................................................................CA....................................................
Ga0184600_102725/60-135 ................................................................................................UGU...................................................
Ga0184602_122974/190-265 ................................................................................................UUGUG.................................................
Ga0256338_1343513/152-228 ................................................................................................UUC...................................................
Ga0255268_1432700/127-51 ................................................................................................UUC...................................................
Ga0157560_1147189/148-60 .........................................................C.A.A..................................CAAG..................................................
Ga0242654_10672177/244-165 .......................................................GCC......................................UUUAC.................................................
Ga0242654_10135580/213-336 ..............................................A..U.....GUG.C.G..................................CCUAUCGUUCACGAUAGGAAAUUAACAGUUUCUAGAACUGA.............
Ga0265742_1000025/2819-2888 ................................................................................................CGA...................................................
Ga0184599_154888/232-155 ................................................................................................AACCCAU...............................................
Ga0228707_1100139/344-194 .......................................................ACG.A.C..................................ACCGCUAAGUUAUAGCGAUUGGUCCUGCCAAGGACUG.................
Ga0307919_1084008/308-378 ................................................................................................AUUAU.................................................
Ga0307919_1039507/513-440 ................................................................................................AAA...................................................
Ga0307921_1024041/406-334 ................................................................................................AA....................................................
Ga0242657_1015112/947-881 ................................................................................................UC....................................................
Ga0307482_1021658/484-563 ...........................................................U.C..................................UUAC..................................................
Ga0307920_133961/233-140 ................................................................................................GAGAGCA...............................................
Ga0307921_1019702/90-184 ......................................................................................................................................................
Ga0182092_1031527/199-109 ................................................................................................UG....................................................
Ga0184575_104772/255-338 ................................................................................................UACAA.................................................
Ga0179940_1033563/296-410 ..............................ACG...CCA.........................................................GG..................................................
Ga0184596_102602/271-200 ................................................................................................CAC...................................................
Ga0184596_125844/4802-4873 ................................................................................................CAC...................................................
Ga0153880_1058095/344-241 .......................................A...............AGC.C.C..................................CUGGCCA...............................................
Ga0153880_1167367/224-121 .......................................A...............AGC.C.C..................................CUGGCCA...............................................
Ga0179943_1178349/195-298 .......................................................AAC.C.C..................................CUGGCCA...............................................
Ga0179944_1313268/129-232 .......................................................AAC.C.C..................................CUGGCCA...............................................
Ga0213850_1259428/445-342 .......................................A...............AGC.C.C..................................CUGGCCA...............................................
Ga0115595_1051440/308-419 ................................................................................................CCACUACGUC............................................
Ga0310106_100998/1608-1690 ................................................................................................UC....................................................
Ga0307482_1017422/1387-1206 ..............................AAU...UCCCAGGUUAG..U.....AGC.U.AC......AAGUUGUG.....GCCGGUAU.CCAUACUCUG.................................................
Ga0307482_1050048/176-95 ................................................................................................UCG...................................................
Ga0179939_1078463/91-178 ................................................................................................CCAC..................................................
Ga0247539_107237/153-229 ................................................................................................AA....................................................
Ga0127502_10189886/163-55 ................................................................................................GAG...................................................
Ga0265746_1085300/256-329 ................................................................................................ACG...................................................
Ga0242641_1002064/1203-1116 .......................................................GC....G..................................AUUUA.................................................
Ga0242657_1090937/459-526 ................................................................................................CC....................................................
Ga0180107_1021361/468-373 ................................U...CCUGAG......................................................CAGGGCGUGAU...........................................
Ga0184599_136526/36-116 ................................................................................................AUC...................................................
Ga0316032_127868/210-130 ................................................................................................AUC...................................................
Ga0032354_1062690/2659-2586 ................................................................................................UC....................................................
Ga0242657_1027062/255-322 ................................................................................................GG....................................................
Ga0265747_105373/294-105 ..............................AGU...CCUAAGG.UAU........AGC.C.AGGUAGUG...........................UGUCAGCACGCGCCACGCGUGAUGUGUCCAGGUUGGGUGAUACCAACCGGCUCC
Ga0265745_1000512/1043-854 ..............................AGU...CCUUAGG.UAU........GGC.C.AGGUAGUG...........................UGUCAGCACGCGCCACGCGUGAUGUGUCCAGGUUGGGUGAUACCAACCGGCUCC
Ga0265745_1001084/890-701 ..............................AGU...CCUAAGG.UAU........AGC.C.AGGUAGUG...........................UGUCAGCACGCGCCACGCGUGAUGUGUUCAGGUUGGGUGAUACCAACCGACUCC
Ga0310117_104105/1058-869 ..............................AGU...CCUUAGG.UAU........AGC.C.AGGUAGUG...........................UGUCAGCACGCACCACGCGUGAUGUGUCCAGGUUGGGUGAUACCAACCGGCUCC
Ga0265745_1000697/596-785 ..............................AGU...CCUAAGG.UAU........AGC.C.AGGUAGUG...........................UGUCAGCACGCGCCACGCGUGAUGUGUCCAGGUUGGGUGAUACCAACCGGCUCC
Ga0247551_106718/112-179 ................................................................................................AC....................................................
Ga0184603_137775/245-174 ................................................................................................UA....................................................
Ga0242641_1105635/265-351 ................................................................................................CAGA..................................................
Ga0242654_10795375/263-349 ................................................................................................CAGA..................................................
Ga0157576_1168696/131-64 ................................................................................................UA....................................................
Ga0255292_1094640/555-357 ................................................................................................CUAAUUGG..............................................
Ga0256349_1073199/279-477 ................................................................................................CUGAGU................................................
Ga0256358_1096062/218-22 ................................................................................................AUCGA.................................................
Ga0256350_1079106/235-433 ......................................................................................................................................................
Ga0256352_1080168/251-449 ......................................................................................................................................................
Ga0255293_1084149/567-369 ................................................................................................AGUCAAG...............................................
Ga0256348_1071395/353-551 .......................................................GCC.A.C..................................AACUAA................................................
Ga0265758_100178/821-753 ................................................................................................UUCG..................................................
Ga0157560_1090855/105-186 ................................................................................................A.....................................................
Ga0005851_1006483/274-172 ..............................GGU...UG.................UUC......................................UUCUU.................................................
Ga0184603_107262/477-596 ......................................CAGGG.UAU..G.....CAA.C.A.......................GG.AC.CUUCCUUUAC.................................................
Ga0184585_151549/495-376 ......................................CAGGG.UAU..G.....CAA.C.A.......................GG.AC.CUUCCUUCAC.................................................
Ga0242647_1022053/339-428 ................................................................................................GU....................................................
Ga0184599_128731/188-273 ................................................................................................C.....................................................
Ga0310114_149639/68-153 ................................................................................................C.....................................................
Ga0310119_131358/533-447 ................................................................................................C.....................................................
Ga0079074_1318576/412-571 ................................................................................................AAACCAACCAGUCAUAUGGUUGCCCAGCGCCCCCU...................
Ga0307484_102285/276-209 ................................................................................................UA....................................................
Ga0247534_109145/204-136 ................................................................................................AAAAAC................................................
Ga0242657_1013457/154-227 ................................................................................................ACG...................................................
Ga0242654_10000487/4104-4168 ................................................................................................UC....................................................

                              >>>...>>.................<<<.<.<<<<<<<<<<<<<<<<<<<<<<<<<<.<<.<<<<<......................................................
                              ......................................................................................................................................................
                              ------------------------------------------------------------------------------------------------●●○○--------------------------------------------------
```

```
Ga0242647_1038796/266-195     ................................................................................................AAC...................................................
Ga0115594_1156252/268-370     ................................................................................................GCCACAA...............................................
Ga0115594_1016490/326-410     ................................................................................................AAACG.................................................
Ga0115595_1007417/328-412     ................................................................................................AAACG.................................................
Ga0265742_1038020/174-90      ................................................................................................G.....................................................
Ga0184568_114501/165-82       ................................................................................................CAAGUG................................................
Ga0265755_113253/366-449      ................................................................................................CAAGUG................................................
Ga0184600_115185/364-447      ................................................................................................GUGGC.................................................
Ga0157593_1013623/299-215     ..............................AAG...AC..........................................................UUCAA.................................................
Ga0153880_1374410/101-33      ................................................................................................CGC...................................................
Ga0316038_101969/827-896      ......................................................................................................................................................
Ga0316046_130251/204-135      ......................................................................................................................................................
Ga0265758_104499/242-367      ..............................AGA...GG....U.GUA..U.....GAC......................................AUGGA.................................................
Ga0307484_108094/186-350      ..............................AAG...CCCAAGA.GCA..C.....GGU.U.GAGGCCA............................UAUAUGAUGUUUAUCCGGGUAAUUCCGGGACCUCAU..................
Ga0307482_1009223/670-508     ..............................AAG...CCCAAGA.GCA..C.....GGU.U.GAGGCCA............................UAUAUGAUGUUUAUCCGGGUAAUUCCGGGACCUCAU..................
Ga0242647_1067563/210-289     ................................................................................................CAC...................................................
Ga0242657_1207391/462-383     ................................................................................................C.....................................................
Ga0265742_1001052/322-80      ..............................AGU...CCAUGGA.AAA..A.....GGC.A.G..................................UUGGCAGUGCUAUCACAGCGGCCACGCUGGAUGAUGUUUCCUGUUGUCUUACGA
Ga0265750_1004028/1262-1504   ..............................AGU...CCAUGGA.AAA..A.....GGC.A.G..................................UUGGCAGUGCUAUCACAGCGGCCACGCUGGAUGAUGUUUCCUGUUGUCUUACGA
Ga0310113_106868/972-730      ..............................AGU...CCAUUGA.AAA..A.....GGC.A.G..................................UUGGCAGUGCUAUCACAGCGGCCACGCUGGAUGAUGUUUCCUGUUGUCUUACGA
Ga0265747_100653/495-253      ..............................AGU...CCAUUGA.AAA..A.....GGC.A.G..................................UUGGCAGUGCUAUCACAGCGGCCACGCUGGAUGAUGUUUCCUUUCGUCUUAUGA
Ga0265752_100504/930-688      ..............................AGU...CCAUUGA.AAA..A.....GGC.A.G..................................UUGGCAGUGCUAUCACAGCGGCCACGCUGGAUGAUGUUUCCUUUCGUCUUAUGA
Ga0265740_1001378/1084-842    ..............................AGU...CCAUUGA.AAA..A.....GGC.A.G..................................UUGGCAGUGCUAUCACAGCGGCCACGCUGGAUGAUGUUUCCUUUCGUCUUAUGA
Ga0265750_1009219/272-30      ..............................AGU...CCAUUGA.AAA..A.....GGC.A.GU.U...............................]..GGCAGUGCUAUCACAGCGGCCACGCUGGAUGAUGUUUCCUUUCGUCUUAUGA
Ga0265757_104669/135-376      ..............................AGU...CCAUAGA.UGA..A.....GGC.A.G..................................UUGGCAGUGCUAUCACAGCGGCCAUGCUGGAUGAUGUUUCCUAUUGUCUUAUGA
Ga0265745_1000618/793-552     ..............................AGU...CCAUAGA.UGA..A.....GGC.A.GU.U.................................GGCAGUGCUAUCACAGCGGCCAUGCUGGAUGAUGUUUCCUAUUGUCUUAUGA
Ga0265742_1088739/51-292      ..............................AGU...CCAUAGA.UGA..A.....GGC.A.GU.U.................................GGCAGUGCUAUCACAGCGGCCAUGCUGGAUGAUGUUUCCUAUUGUCUUAUGA
Ga0265747_100509/1277-1036    ..............................AGU...CCAUAGA.UGA..A.....GGC.A.GU.U.................................GGCAGUGCUAUCACAGCGGCCAUGCUGGAUGAUGUUUCCUAUUGUCUUAUGA
Ga0265752_100314/1466-1225    ..............................AGU...CCAUAGA.UGA..A.....GGC.A.GU..................................UGGCAGUGCUAUCACAGCGGCCAUGCUGGAUGAUGUUUCCUAUUGUCUUAUGA
Ga0265743_104537/484-553      ................................................................................................UU....................................................
Ga0265744_160011/83-18        ................................................................................................CC....................................................
Ga0153880_1410217/288-397     ..............................ACA...CU.AUGC.ACA.................................................UCA...................................................
Ga0115594_1054328/389-276     .......................................................UGA.C....................................GCACCACUAC............................................
Ga0316029_112968/271-337      ................................................................................................UCG...................................................
Ga0157560_1034839/242-321     ................................................................................................GA....................................................
Ga0157566_1117351/242-321     ................................................................................................GA....................................................
Ga0157593_1112297/86-12       ................................................................................................U.....................................................
Ga0157565_1020724/148-72      ................................................................................................U.....................................................
Ga0247519_104852/800-730      ...............................................................CCC..............................AUCC..................................................
Ga0307480_1000549/913-1064    .......................................................CUC.G.A..................................AAGCCUUGAGU...........................................
Ga0316031_132099/178-294      ...........................................................A.UCGCCGCCGGGCUGGCCC.................GCGUUU................................................
Ga0247526_116548/279-354      .......................................................AU.......................................CCUAG.................................................
Ga0247519_113845/334-409      .......................................................AUC......................................UUA...................................................
Ga0247529_111070/303-228      .......................................................AUC......................................UUA...................................................
Ga0184602_101759/1045-970     ................................................................................................CUA...................................................
Ga0184600_139292/252-327      ................................................................................................A.....................................................
Ga0184580_108231/296-221      ................................................................................................A.....................................................
Ga0247534_105038/288-211      ................................................................................................A.....................................................
Ga0247552_109074/256-333      ................................................................................................A.....................................................
Ga0247542_100296/1022-1097    ................................................................................................A.....................................................
Ga0247545_113720/232-157      ................................................................................................A.....................................................
Ga0184597_110829/1090-1011    ................................................................................................ACAGAC................................................
Ga0184600_105920/81-160       .......................................................GGC.U.G..................................UAGAAUAAA.............................................
Ga0184578_129447/314-235      ................................................................................................AUGCAG................................................
Ga0184602_121948/324-245      .........................................................G......................................UAGAAUAUA.............................................
Ga0184603_125382/9-88         .......................................................GUA.U.A..................................AGAA..................................................
Ga0247550_100138/208-129      .......................................................GUA.U.A..................................AGAA..................................................
Ga0316046_122965/221-142      ................................................................................................AU....................................................
Ga0316051_1003245/138-56      ................................................................................................AU....................................................
Ga0316049_122425/209-288      ................................................................................................AAUAUACAA.............................................
Ga0184588_113246/331-252      ................................................................................................UGUAGAAUAGACAGAC......................................
Ga0184582_111448/113-237      ................................................................................................UC....................................................
Ga0184597_112736/543-466      ................................................................................................UC....................................................
Ga0184577_100528/550-473      ................................................................................................UC....................................................
Ga0127502_10397722/141-209    ................................................................................................C.....................................................
Ga0184593_119512/675-608      ................................................................................................CG....................................................
Ga0307482_1597388/86-18       ................................................................................................CA....................................................
Ga0307482_1001973/47-115      ................................................................................................CA....................................................
Ga0184595_132469/902-822      ................................................................................................ACUAC.................................................
Ga0079039_1443516/199-38      ..............................AGG...UGUA...............GUU.U.U..................................GUGGCCCGCCCUGGUGACCGUCAGACCAGUUCUGCGUUCCU.............
Ga0265756_100130/1310-1239    ................................................................................................C.....................................................
Ga0265747_125875/320-395      ................................................................................................GAG...................................................
Ga0180052_189627/240-346      ..............................GGC...CG..........................................................CGCAA.................................................
Ga0180044_1074338/112-33      ................................................................................................AA....................................................
Ga0157559_1047848/128-41      ................................................................................................GUCAGGCUG.............................................
Ga0157562_1134247/69-160      ................................................................................................GUCAGGCUG.............................................
Ga0247548_101188/825-888      ................................................................................................GA....................................................
Ga0228707_1093045/293-207     ................................................................................................CA....................................................
Ga0184597_119383/488-559      ................................................................................................UA....................................................
Ga0184602_121040/35-106       ................................................................................................A.....................................................
Ga0326631_107633/416-489      ................................................................................................A.....................................................
Ga0316046_149266/91-162       ................................................................................................A.....................................................
Ga0247538_100917/537-466      ................................................................................................A.....................................................
Ga0247519_118271/334-405      ................................................................................................CAU...................................................
Ga0265747_100454/1395-1324    ................................................................................................UG....................................................
Ga0316046_100502/370-442      ................................................................................................AU....................................................
Ga0247551_101484/617-543      ................................................................................................AU....................................................
Ga0213850_1280121/544-351     ................................................................................................CACACGUUGUGCUUAGACUGCAUGCGGGGUGGGCUUGUGACCACCACUGCUGCA
Ga0265759_100017/4211-4288    ................................................................................................GAUGU.................................................

                              ..............................>>>...>>.................<<<.<.<<<<<<<<<<<<<<<<<<<<<<<<<<.<<.<<<<<......................................................
                              ......................................................................................................................................................
                              ------------------------------------------------------------------------------------------------●○○○--------------------------------------------------
```

```
Ga0307482_1127544/505-398     ......................................CCAGC.UUU..U.....CGU.U.G..................................GUCUC.................................................
Ga0180107_1057591/210-107     .........................................................A.U.C..................................CUGAAACCCUCGAGAGGGAUAACAGAA...........................
Ga0157569_1061476/44-119      ................................................................................................AA....................................................
Ga0307484_100889/412-594      ..............................AAU...UCCCAGG.UUA..G....CAGU.U.A..................................CCAAGUUGUGUCAGGUAUCCAUACUCUGUGUGCGUCCUGCUCAAUCACAACUGA
Ga0310106_100022/563-489      ................................................................................................AC....................................................
Ga0310115_100154/4483-4559    ................................................................................................AC....................................................
Ga0180107_1262812/2765-2832   ................................................................................................CG....................................................
Ga0184597_110025/865-793      ................................................................................................GA....................................................
Ga0247550_100495/487-560      ................................................................................................GA....................................................
Ga0184582_135749/62-135       ................................................................................................GA....................................................
Ga0184603_122688/908-835      ................................................................................................GA....................................................
Ga0247524_126530/125-198      ................................................................................................GAUGA.................................................
Ga0316030_115438/368-435      ................................................................................................A.....................................................
Ga0307354_155926/276-116      ................................................................................................ACAAACCAGCCAGUCAUAUGGCUUCCGCGGCACUCCU.................
Ga0079074_1279626/114-274     ................................................................................................GCCGGAACAAACCAGCCAGUCAUAUGGCUUCCGCGGCACUCCU...........
Ga0184602_132007/314-246      ................................................................................................CA....................................................
Ga0307921_1005950/791-877     ................................................................................................AUCAG.................................................
Ga0242654_10000487/4544-4475  ................................................................................................CAA...................................................
Ga0157569_1032488/120-205     ................................................................................................CGCGAAGGA.............................................
Ga0157593_1125711/213-128     ................................................................................................CGCGAAGGA.............................................
Ga0157565_1077069/156-67      .......................................................AUC.C.U..................................CUCGA.................................................
Ga0180047_1128081/151-64      .......................................................AUC.C.U..................................CUCGA.................................................
Ga0265756_135986/152-290      ................................................................................................CUUGCAGCAGGAAAC.......................................
Ga0180045_119797/166-305      ......................................CACGC.CUU..U.....AGG.U.GUGCGUGAG..........................CCCGCGAACCGUGAACAAGCCAG...............................
Ga0115595_1089740/408-494     ................................................................................................CACU..................................................
Ga0179955_1048341/123-51      ................................................................................................UACAU.................................................
Ga0138280_1140856/341-479     ..............................GCA...CU.................AUU.U.CCAAGUG............................CGAGCGAAAG.AAU.GA......................................
Ga0180106_1302226/203-273     ................................................................................................G.....................................................
Ga0310119_169621/253-177      ................................................................................................GUAUG.................................................
Ga0138277_1023917/182-18      ..............................GAU...............................................................GUAUUUCCAAAUGCAGCGAUUCUUAAGAAAGAACGGCAGAU.............
Ga0138282_1020546/737-573     ..............................GAU...............................................................GUAUUUCCAAAUGCAGCGAUUCUUAAGAAAGAACGGCAGAU.............
Ga0179939_1059820/107-216     ................................................................................................GCUCA.................................................
Ga0179950_1098211/345-278     ................................................................................................C.....................................................
Ga0115594_1068468/127-56      ................................................................................................G.....................................................
Ga0228708_1103570/188-285     ................................................................................................C.....................................................
Ga0265751_160438/108-215      ..............................GCU...CACA...............ACC.G.C.................................GAGAAG.................................................
Ga0242647_1000066/2638-2719   ................................................................................................CUCUCGUUAGAGUUU.......................................
Ga0242646_1001227/1243-1308   ................................................................................................UC....................................................
Ga0182091_1104347/98-194      ................................................................................................AU....................................................
Ga0115594_1178636/111-46      ................................................................................................AA....................................................
Ga0213850_1508869/496-402     ................................................................................................AAUA..................................................
Ga0307482_1152465/114-38      ................................................................................................UCAC..................................................
Ga0180107_1031754/139-57      ................................................................................................UAU...................................................
Ga0316037_121702/176-240      ................................................................................................AGA...................................................
Ga0265759_107962/103-39       ................................................................................................AUAGA.................................................
Ga0242649_1051170/368-302     ................................................................................................AA....................................................
Ga0307482_1064439/84-153      ................................................................................................A.....................................................
Ga0115594_1006122/120-29      ................................................................................................ACGAA.................................................
Ga0115595_1180708/226-317     ................................................................................................ACGAA.................................................
Ga0265746_1064626/389-458     ................................................................................................CCG...................................................
Ga0265759_108120/277-351      ................................................................................................UAU...................................................
Ga0307482_1000450/2015-2077   ................................................................................................A.....................................................
Ga0247527_115808/280-440      ...............................AC...CCAAGC.ACA.........AGC.U.GC.........CAU.CCAU................AUGUG.................................................
Ga0184588_120624/266-426      ...............................AC...CCAAGC.ACA.........AGC.U.GC.........CAUCCAU.................AUGCGA................................................
Ga0184579_106194/996-1156     ...............................AC...CCAAGC.ACA.........AGC.U.GC.........CAUCCAU.................AUGCGA................................................
Ga0265758_100254/356-516      ...............................AC...CCAAGC.ACA.........AGC.U.GC.........CAUCCAU.................AUGCGA................................................
Ga0316035_107544/354-514      ...............................AC...CCAAGC.ACA.........AGC.U.GC.........CAUCCAU.................AUGUG.................................................
Ga0184596_109261/979-1139     ...............................AC...CCAAGC.ACA.........AGC.U.GC.........CAUCCAU.................AUGUG.................................................
Ga0184600_132066/568-408      ...............................AC...CCAAGC.ACA.........AGC.U.GC.........CAUCCAU.................AUGUG.................................................
Ga0184597_106861/196-36       ...............................AC...CCAAGC.ACA.........AGC.U.GC.........CAUCCAU.................AUGUG.................................................
Ga0316031_108320/216-376      ...............................AC...CCAAGC.ACU.........AGC.U.GC.........CAUCCAU.................AUGUG.................................................
Ga0184589_110648/1041-881     ...............................AC...CCAAGC.ACA.........AGC.U.GC.........CAUCCAU.................AUGUG.................................................
Ga0184580_124121/239-79       ...............................AC...CCAAGC.ACA.........AGC.U.GC.........CAUCCAU.................AUGUG.................................................
Ga0184598_101324/530-690      ...............................AC...CCAAGC.ACA.........AGC.U.GC.........CAUCCAU.................AUGUG.................................................
Ga0247547_102464/189-29       ...............................AC...CCAAGC.ACA.........AGC.U.GA........CUGC.CGU.................AUGAG.................................................
Ga0247551_100828/546-387      ...............................AC...CCAAGC.ACA.........AGC.U.GA........CUGC.CGU.................AUGAG.................................................
Ga0247536_101914/360-201      ...............................AC...CCAAGC.ACA.........AGC.U.GA........CUGC.CGU.................AUGAG.................................................
Ga0247538_100480/788-947      ...............................AC...CCAAGC.ACA.........AGC.U.GA........CUGC.CGU.................AUGAG.................................................
Ga0247550_100850/456-297      ...............................AC...CCAAGC.ACA.........AGC.U.GA........CUGC.CGU.................AUGAG.................................................
Ga0247553_110698/526-367      ...............................AC...CCAAGC.ACA.........AGC.U.GA........CUGC.CGU.................AUGAG.................................................
Ga0184589_112135/40-200       ...............................AC...CCAAGC.ACA.........AGC.U.GAA........CCGCGAU.................AUGCG.................................................
Ga0247526_110221/603-443      ...............................AC...CCAAGC.ACA.........AGC.U.GA.........CCGCCAU.................AUGUG.................................................
Ga0247541_103784/123-283      ...............................AC...CCAAGC.ACA.........AGC.U.GA.........CCGCAAU.................AUGUG.................................................
Ga0247545_112960/183-343      ...............................AC...CCAAGC.ACA.........AGC.U.GA.........CCGCAAU.................AUGUG.................................................
Ga0184586_133850/368-528      ...............................AC...CCAAGC.ACA.........AGC.U.GA.........CCGCCAU.................AUGUG.................................................
Ga0247552_101742/612-452      ...............................AC...CCAAGC.ACA.........AGC.U.GA.........CCGCCAU.................AUGUG.................................................
Ga0247545_104341/406-246      ...............................AC...CCAAGC.ACA.........AGC.U.GA.........CCGCCAU.................AUGUG.................................................
Ga0184602_114416/11-171       ...............................AC...CCAAGC.ACA.........AGC.U.GA.........CCGCCAU.................AUGUG.................................................
Ga0316038_111670/11-171       ...............................AC...CCAAGC.ACA.........AGC.U.GA.........CCGCCAU.................AUGUG.................................................
Ga0247529_134913/224-64       ...............................AC...CCAAGC.ACA.........AGC.U.GA.........CCGUCAU.................AUGUG.................................................
Ga0184603_143085/253-93       ...............................AC...CCAAGC.ACA.........AGC.U.GA.........CCGUCAU.................AUGUG.................................................
Ga0316032_107575/215-55       ...............................AC...CCAAGC.ACA.........AGC.U.GA.........CCGUCAU.................AUGUG.................................................
Ga0247519_110701/607-447      ...............................AC...CCAAGC.ACA.........AGC.U.GA.........CCGUCAU.................AUGUG.................................................
Ga0247524_111427/50-210       ...............................AC...CCAAGC.ACA.........AGC.U.GA.........CCGUCAU.................AUGUG.................................................
Ga0184574_121290/269-109      ...............................AC...CCAAGC.ACA.........AGC.U.GA.........CCGCCAU.................AUGUG.................................................
Ga0184595_111222/3-163        ...............................AC...CCAAGC.ACA.........AGC.U.GA.........CCGCAAU.................AUGUG.................................................
Ga0184600_123882/188-28       ...............................AC...CCAAGC.ACA.........AGC.U.GA.........CCGUCAU.................AUGCG.................................................
Ga0326631_101563/544-705      ...............................AC...CCAAGC.ACA.........AGC.U.GAA........CUGCGAU.................AUGUA.................................................
Ga0184573_110190/115-276      ...............................AC...CCAAGC.ACA.........AGC.U.GAA........CUGCGAU.................AUGAG.................................................

                              ..............................>>>...>>.................<<<.<.<<<<<<<<<<<<<<<<<<<<<<<<<<.<<.<<<<<......................................................
                              ......................................................................................................................................................
```

```
Ga0184596_111007/525-686       ..............................AC...CCCAAGC.ACA.......AGC.U.GAA.......CUGCGAU...................AUGAG................................................
Ga0316033_144939/167-328       ..............................AC...CCCAAGC.ACA.......AGC.U.GAA.......CUGCGAU...................AUGAG................................................
Ga0247526_117235/152-313       ..............................AC...CCCAAGC.ACA.......AGC.U.GAA.......CCGCGAU...................AUGCG................................................
Ga0247551_100195/490-329       ..............................AC...CCCAAGC.ACA.......AGC.U.GAA.......CCGCGAU...................AUGCG................................................
Ga0247538_100424/120-281       ..............................AC...CCCAAGC.ACA.......AGC.U.GAA.......CUGCGAU...................AUGAG................................................
Ga0247550_114736/167-7         ..............................AC...CCCAAGC.ACA.......AGC.U.GAA.......CUGCGAU...................AUGAG................................................
Ga0247529_103231/270-430       ..............................AC...CCCAAGC.ACA.......AGC.U.GAA.......CUGCGAU...................AUGAG................................................
Ga0184603_111918/241-82        ..............................AC...CCCGAGC.ACA.......AGC.U.GAA.......CUGCGAU...................AUGAG................................................
Ga0184603_126764/80-240        ..............................AC...CCCAAGC.ACA.......AGC.U.GCA.......CUGCGAU...................AUGAG................................................
Ga0247519_107918/217-58        ..............................AC...CCCAAGC.ACA.......AGC.U.GAA.......CUGCGAU...................AUGAG................................................
Ga0184580_114961/306-146       ..............................AC...CCCAAGC.ACA.......AGC.U.GAA.......CUGCGAU...................AUGAG................................................
Ga0213850_1042834/351-428      ...............................................................................................GAG..................................................
Ga0265756_103656/97-163        ...............................................................................................AG...................................................
Ga0265756_101189/896-833       ...............................................................................................CC...................................................
Ga0180107_1033011/414-478      ...............................................................................................CG...................................................
Ga0115594_1157594/155-254      .....................................AAGG......................................................CAUACCGCCACAA........................................
Ga0115594_1166236/119-19       .....................................AAG.......................................................GCAGUAUCGCCACAA......................................
Ga0115594_1007070/120-30       ...............................................................................................UAAACGA..............................................
Ga0115595_1181384/176-86       ...............................................................................................UAAACGA..............................................
Ga0115594_1211214/299-389      ...............................................................................................CUCAA................................................
Ga0115594_1182529/183-84       ...............................................................................................CGCCACAAA............................................
Ga0256352_1182139/180-81       ...............................................................................................ACGCCACAAGU..........................................
Ga0179944_1005311/261-382      .....................................................GGC.A.UCACAG..............................CGCAUGA..............................................
Ga0213850_1508120/373-299      ...............................................................................................CAAAG................................................
Ga0307484_162891/325-258       ...............................................................................................UC...................................................
Ga0184596_111635/86-148        ...............................................................................................C....................................................
Ga0316051_1005111/447-509      ...............................................................................................CC...................................................
Ga0265753_1379003/85-148       ...............................................................................................CC...................................................
Ga0184603_116647/206-269       ...............................................................................................CC...................................................
Ga0184587_132427/565-485       ...............................................................................................C....................................................
Ga0184599_110049/275-349       ...............................................................................................UC...................................................
Ga0184603_148109/996-920       ...............................................................................................UC...................................................
Ga0310114_133004/56-132        ...............................................................................................UC...................................................
Ga0310119_113172/381-306       ...............................................................................................UC...................................................
Ga0265747_119090/8-84          ...............................................................................................UAAC.................................................
Ga0247551_122551/29-115        .....................................................GGG.G.G...................................UUUA.................................................
Ga0316048_107802/239-324       .....................................................GGG.G.G...................................GUUAC................................................
Ga0242657_1208314/452-382      ...............................................................................................UCAU.................................................
Ga0184596_129213/83-168        ...............................................................................................AU...................................................
Ga0184603_146917/441-510       ...............................................................................................A....................................................
Ga0247550_102601/160-89        ...............................................................................................UGA..................................................
Ga0184602_110024/263-177       ...............................................................................................GAGU.................................................
Ga0184597_116115/700-787       ...............................................................................................GAGU.................................................
Ga0184582_128596/700-786       ...............................................................................................GAGU.................................................
Ga0184597_102019/330-402       ...............................................................................................GAGU.................................................
Ga0242649_1000320/700-631      ...............................................................................................ACCG.................................................
Ga0179943_1102267/215-90       .....................................................GGC.A.UCACAG..............................CGCUUGA..............................................
Ga0179944_1191552/284-411      .....................................................GGC.A.UCACAG..............................CGCUUGA..............................................
Ga0179943_1150468/216-89       .....................................................GGC.A.UCAUUGCU............................UACAC................................................
Ga0179944_1204933/216-89       .....................................................GGC.A.UCAUUGCU............................UACAC................................................
Ga0265758_104558/436-365       ...............................................................................................G....................................................
Ga0180107_1040315/471-547      ...............................................................................................CC...................................................
Ga0180106_1332058/4386-4449    ...............................................................................................A....................................................
Ga0307484_104189/90-171        ...............................................................................................AAAA.................................................
Ga0265752_100023/1212-1290     ...............................................................................................CCG..................................................
Ga0157593_1101573/285-478      .............................GGA...UG................GGC.G.U.......................AGU.UC.AUAGGGUUG.................................................
Ga0307482_1008742/1212-1288    ...............................................................................................CAU..................................................
Ga0247551_104770/284-362       ...............................................................................................CGUC.................................................
Ga0184593_112903/290-212       ...............................................................................................CGUC.................................................
Ga0307482_1128390/271-339      ...............................................................................................G....................................................
Ga0184580_126177/800-732       ...............................................................................................CC...................................................
Ga0242646_1115622/191-109      ...............................................................................................UC...................................................
JGI12272J11983_1319142/230-159 ...............................................................................................ACCCC................................................
Ga0184596_105062/163-229       ...............................................................................................CC...................................................
Ga0307482_1011891/77-10        ...............................................................................................CC...................................................
Ga0184588_125064/416-488       ...............................................................................................GAUGA................................................
Ga0184577_107529/254-327       ...............................................................................................GAUGA................................................
Ga0180106_1182034/403-337      ...............................................................................................GAC..................................................
Ga0180107_1241189/1554-1620    ...............................................................................................GAC..................................................
Ga0222728_1000110/4754-4821    ...............................................................................................CA...................................................
Ga0307484_100154/562-629       ...............................................................................................CA...................................................
Ga0180107_1285439/94-155       ...............................................................................................AA...................................................
Ga0184580_116530/249-177       ...............................................................................................GAUA.................................................
Ga0247526_117664/434-499       ...............................................................................................UUAU.................................................
Ga0184580_120219/79-4          ...............................................................................................A....................................................
Ga0247539_100482/682-612       ...............................................................................................AU...................................................
Ga0247516_124175/202-272       ...............................................................................................UUAU.................................................
Ga0184580_121247/966-1036      ...............................................................................................AU...................................................
Ga0265743_100411/1096-1162     ...............................................................................................UUCAU................................................
Ga0242657_1070636/108-176      ...............................................................................................AC...................................................
Ga0242657_1122750/95-162       .....................................................................................................................................................
Ga0307482_1405960/253-322      ...............................................................................................AC...................................................
Ga0316049_154968/227-319       ......................................................CA....ACAUUGGAU.........................UUGU.................................................
Ga0127502_10852756/278-345     ...............................................................................................UGU..................................................
Ga0247534_100003/3657-3582     ...............................................................................................UG...................................................
Ga0180106_1204848/284-221      ...............................................................................................AG...................................................
Ga0184585_128240/467-390       ...............................................................................................U....................................................
Ga0180106_1180558/1549-1486    ...............................................................................................U....................................................
Ga0265755_100134/298-372       ...............................................................................................UAGU.................................................

                               .............................>>>...>>................<<<.<.<<<<<<<<<<<<<<<<<<<<<<<<<<.<<.<<<<<.....................................................
                               .....................................................................................................................................................
                               -----------------------------------------------------------------------------------------------●○○○-------------------------------------------------
```

```
Ga0247534_100772/309-234     ................................................................................................GCGUUG................................................
Ga0307482_1202063/402-466    ................................................................................................CG....................................................
Ga0247514_114593/280-211     ................................................................................................UUUUU.................................................
Ga0310101_157764/124-55      ................................................................................................UAAUU.................................................
Ga0265745_1000136/129-60     ................................................................................................UGAUU.................................................
Ga0265745_1013560/201-270    ................................................................................................UAAUU.................................................
Ga0307482_1779657/200-270    ................................................................................................A.....................................................
Ga0307482_1044183/658-591    ................................................................................................A.....................................................
Ga0265758_100017/4376-4309   ................................................................................................UC....................................................
Ga0242654_10617983/312-237   ................................................................................................U.....................................................
Ga0222728_1054736/119-193    ................................................................................................UU....................................................
Ga0184583_106441/296-227     ................................................................................................UU....................................................
Ga0247520_104849/891-821     ................................................................................................UCCG..................................................
Ga0247518_137824/301-231     ................................................................................................UUCG..................................................
Ga0247531_104861/1082-1012   ................................................................................................UCCG..................................................
Ga0265746_1040401/190-123    ......................................................................................................................................................
Ga0184603_139760/199-128     ................................................................................................C.....................................................
Ga0184603_138085/381-311     ................................................................................................C.....................................................
Ga0265747_102820/448-376     ................................................................................................C.....................................................
Ga0247554_105388/388-316     ................................................................................................C.....................................................
Ga0316037_123461/289-368     ................................................................................................AGA...................................................
Ga0184597_121517/262-183     ................................................................................................AGA...................................................
Ga0247551_100118/653-723     ................................................................................................UUC...................................................
Ga0265750_1365815/134-55     .......................................................AUG.U.C..................................CAUUA.................................................
Ga0184589_120335/11-90       .......................................................AUG.U.C..................................CAUCA.................................................
Ga0247521_124040/388-309     .......................................................AUG.U.C..................................CAUCA.................................................
Ga0247518_108423/758-837     .......................................................AUG.U.C..................................CAUCA.................................................
Ga0247531_104608/1256-1177   .......................................................AUG.U.C..................................CAUCA.................................................
Ga0247543_100863/642-721     .......................................................AUG.U.C..................................CAUCA.................................................
Ga0307482_1026216/884-813    ................................................................................................A.....................................................
Ga0307482_1636410/270-341    ................................................................................................UUA...................................................
Ga0310119_128165/432-365     ................................................................................................AUA...................................................
Ga0307482_1016877/168-97     ................................................................................................UGAGA.................................................
Ga0126318_10746885/3334-3265 ................................................................................................UC....................................................
Ga0206353_11117566/971-1032  ................................................................................................UC....................................................
Ga0180107_1106188/424-495    ................................................................................................UUAUCGGU..............................................
Ga0307482_1000354/2063-1987  ................................................................................................UUUGCUGU..............................................
Ga0180107_1132096/355-287    ................................................................................................A.....................................................
Ga0180108_1238667/303-371    ................................................................................................CAA...................................................
Ga0265751_100981/715-788     ................................................................................................AGA...................................................
Ga0265756_100898/263-336     ................................................................................................AUA...................................................
Ga0307484_102094/153-80      ................................................................................................CUUC..................................................
Ga0307482_1023270/273-197    ................................................................................................CCUG..................................................
Ga0184596_102741/625-701     ................................................................................................CUA...................................................
Ga0316033_103637/453-391     ................................................................................................UCAC..................................................
Ga0180107_1069127/170-106    ................................................................................................A.....................................................
Ga0247535_105396/277-348     ................................................................................................CACA..................................................
Ga0184576_110410/277-348     ................................................................................................AC....................................................
Ga0307482_1219330/19-95      ................................................................................................A.....................................................
Ga0247534_116177/42-105      ................................................................................................CG....................................................
Ga0316033_123149/165-228     ................................................................................................CG....................................................
Ga0265751_100560/642-572     ................................................................................................AG....................................................
Ga0307482_1011142/698-628    ................................................................................................U.....................................................
Ga0307482_1465843/259-190    ................................................................................................U.....................................................
Ga0184593_115334/333-240     .......................................................ACA.U.C..................................GAAUGUGUAUCC..........................................
Ga0265751_100089/3005-3069   ................................................................................................A.....................................................
Ga0307482_1000325/380-315    ................................................................................................CU....................................................
Ga0222729_1014021/463-392    ................................................................................................UC....................................................
Ga0307482_1033651/424-494    ................................................................................................GA....................................................
Ga0307482_1015330/1021-1093  ................................................................................................UAU...................................................
Ga0184570_111551/35-97       ................................................................................................CAC...................................................
Ga0184576_117861/362-299     ................................................................................................CAC...................................................
Ga0307482_1589279/351-280    ................................................................................................A.....................................................
Ga0184586_104419/174-104     ................................................................................................AUA...................................................
Ga0222728_1148620/185-250    ................................................................................................AAAA..................................................
Ga0316049_103217/889-823     ................................................................................................AAAA..................................................
Ga0316034_104024/504-439     ................................................................................................G.....................................................
Ga0184595_117496/296-230     ................................................................................................AAGA..................................................
Ga0184597_109849/897-963     ................................................................................................UAGA..................................................
Ga0184588_134146/1219-1285   ................................................................................................UAGA..................................................
Ga0184584_115247/186-121     ................................................................................................GA....................................................
Ga0184596_126546/83-162      ................................................................................................ACUA..................................................
Ga0184570_116436/278-198     ................................................................................................UAGAC.................................................
Ga0184597_111165/262-182     ................................................................................................UAGAC.................................................
Ga0316037_169124/279-199     ................................................................................................UAGAC.................................................
Ga0247551_103383/322-242     ................................................................................................UAGAC.................................................
Ga0247543_103170/244-164     ................................................................................................UAGAC.................................................
Ga0184589_120451/249-329     ................................................................................................UAGAC.................................................
Ga0184600_118565/177-97      ................................................................................................GAGU..................................................
Ga0316034_100493/254-174     ................................................................................................GAGU..................................................
Ga0184576_117703/380-460     ................................................................................................A.....................................................
Ga0184597_129399/366-446     ................................................................................................A.....................................................
Ga0184590_111002/143-223     ................................................................................................UUAGAA................................................
Ga0184587_105985/71-151      ................................................................................................AGACUAU...............................................
Ga0184602_122864/595-515     ................................................................................................GUAGACUAU.............................................
Ga0184593_101086/682-762     ................................................................................................GACUAU................................................
Ga0247519_108375/286-206     .......................................................GUA.U.AG.................................UAGA..................................................
Ga0127502_10351452/615-550   ................................................................................................U.....................................................
Ga0316038_112263/442-356     .......................................................GG..C.C..................................AUGGC.................................................

                             ..............................>>>...>>.................<<<.<.<<<<<<<<<<<<<<<<<<<<<<<<<<.<<.<<<<<......................................................
                             ......................................................................................................................................................
                             ------------------------------------------------------------------------------------------------●○○○----------------------------------------------
```

```
Ga0242657_1073895/573-672    ...AUA...
Ga0184587_107783/167-95      ...AAU...
Ga0265758_101918/310-243     ...GAU...
Ga0307482_1029599/655-579    ...CAU...
Ga0265746_1009702/779-848    ...C...
Ga0184583_113025/102-26      ...AGUACACUAA...
Ga0184580_109961/270-194     ...GUACACUA...
Ga0316049_111814/256-181     ...AUACACUA...
Ga0247553_120631/200-275     ...AGUAUGCAA...
Ga0184593_119849/313-238     ...GUA...CAGAAUG...
Ga0184599_140898/774-849     ...GUA...CAGAAUG...
Ga0242647_1005188/439-360    ...ACUUAAA...
Ga0242657_1054283/541-620    ...CUUGAA...
Ga0184597_102100/600-669     ...UCUA...
Ga0213850_1460186/227-149    ...GAG...
Ga0242654_10040657/1018-942  ...AA...
Ga0307484_100842/1194-1271   ...UUCA...
Ga0242654_10401151/84-171    ...AA...
Ga0310119_105137/743-673     ...C...
Ga0247541_104364/333-188     ...UU.CA.UUAUCCGCGUUAAUC.A...
Ga0184582_129125/749-675     ...A...
Ga0265753_1022489/457-531    ...A...
Ga0265751_121301/189-266     ...A...
Ga0247526_105246/689-508     ...AAA..CCCAAGG.CAU..U....GGU.U.CAGUGUGU..CGUUGCUCGGGCCUU.UC.UUACCUAAAC...
Ga0247515_107373/548-731     ...AAU..CCCAAGG.CAU..U....GGU.U.CAGUGUGU..CGUUGCUCAGGCCUU.UC..UACCUUAGU...
Ga0247538_100442/1363-1181   ...AAU..CCCAAGG.CAU..U....GGU.U.CAGUGUGU..CGUUGCUCAGGCCUU.UC..UACCUAAAC...
Ga0247538_100551/721-542     ...AAU..UCCAAGG.CAU..U....GGU.U.CAGUGUGU..CGUUGCUCAGGCCUU.U..CAACCUAAAU...
                             ...>>>..>>...<<<.<.<<<<<<<<<<<<<<<<<<<<<<<<<<.<<.<<<<<...
                             ---●◦○○---
```

alignment positions 301 · · · 450

```
Ga0316035_118720/195-42      ...ACACA...U.CACCUAC...GUG.U..CAA.A.A.AGAAAGUA..U
Ga0265747_125480/96-202      ...AUUA........AGU.A.A.UGAAGAGG..A
Ga0242657_1353977/135-311    ...UAUCAAU........UUUC.A.G.GGAAGAAG..U
Ga0242649_1051170/34-143     ...GGGUAUA...U.C..AGC.A.G.GGAAGAUG..A
Ga0265758_100138/1527-1633   ...GUAUU...G...GCCA.......CCC.G.A.UGAGGACA..C
Ga0184593_103988/348-257     ...GAUACGAC........UAG.A.G.AGAAGAUUA.G
Ga0316047_106478/13-108      ...AGU........ACC.A.G.AGAAGUGG..G
Ga0213850_1056422/14-139     ...ACC........GUC.G.A.GGAAGACA..G
Ga0316032_128094/136-241     ...GAUGU...UGGCC...........ACCC.G.A.UGAGGCAU..C
Ga0307482_1000311/1469-1363  ...GGGUGAUC...G.G.AUUC.C.G.GGAAGAGU..G
Ga0242657_1051800/416-321    ...AACGC......A.G.AUCU.G.G.AGAAGUUG..C
Ga0307921_1050750/452-358    ...GA.G.UUCA.G.G.GGAAGCAA..G
Ga0265756_117960/316-418     ...CGAUGGU...GAC......AA.C.AACG.U.G.UGAAGGACA.G
Ga0242654_10352202/387-307   ...UACU.C.U.AGAAGUCU..C
Ga0242652_1159268/284-150    ...UGCC..........CAA.G.G.AGAAGAUU..G
Ga0265744_102237/225-365     ...ACCA........GAU.G.A.AGAAGAAGG.A
Ga0316029_101396/632-732     ...CACG..........UAU.A.G.GGAAGUCU..G
Ga0265759_100202/139-258     ...GGGUUCAAA..CG..C.UAUA.U.G.AGAAGUGA..C
Ga0179943_1118543/65-150     ...A.U.CUUA.A.G.GGAAGCGG..C
Ga0182091_1018330/100-226    ...G...CGCA...UCGACUAGA........AGA.A.A.CGAGGCAU..A
Ga0127503_10119710/546-465   ...G.ACAA.G.A.UGAAGUCC..G
Ga0138277_1022592/399-305    ...U.G.GCAA.C.G.AGAAGCUG..G
Ga0138278_1026470/183-287    ...C.ACAA.C.G.AGAAGCUG..G
Ga0138277_1089473/408-304    ...U.C.GCAA.C.G.AGAAGCUG..G
Ga0247538_105452/166-82      ...G..........AC.C.A.UGAAGACA..G
Ga0247554_102783/498-580     ...C.CCAC.C.A.UGAAGACA..G
Ga0138291_1177333/295-213    ...ACA.C.A.UGAAGACG..A
Ga0210324_1290306/191-291    ...CCCAU....CAC.U.ACUC.A.A.UGAAGAGGA.G
Ga0242657_1026087/460-322    ...GGGUAGU.UGUCUUCA.A.GGCGU...GUGC..........CCCA.U.G.AGAAGGAUU.G
Ga0307482_1147486/107-4      ...GCUA........CUU.G.G.GGAAGUUA..C
Ga0184573_113483/125-229     ...AUU..GGC.U.AUGC.G.G.AGAAGAUG..A
Ga0184576_116834/47-151      ...AUU..GAC.U.AUGC.G.G.AGAAGAUG..A
Ga0184568_108419/492-395     ...GGCU......G.C.UUAU.G.A.AGAGGUCG..G
Ga0307482_1023050/594-496    ...A.UGUC.A.G.GGAAGAGC..G
Ga0179955_1013331/439-311    ...CGUC.G.A.AGAAGACG..G
Ga0184596_112078/314-191     ...CAUACAU..........AGU.C.A.AGAAGUAU..U
Ga0184602_108471/49-173      ...CAUACAU..........AGU.C.A.AGAAGUAU..U
Ga0247528_108647/438-562     ...AU...UCCAUACAU..........AGU.C.A.AGAAGUAU..U
Ga0316039_120091/354-252     ...U.G.G.GGAAGUAG..A
Ga0307484_166952/62-163      ...GACCUA........A.UAGU.A.A.CGAAGACCU.G
Ga0180107_1244437/408-507    ...AUGA....G.GUUU.U.G.AGAAGAUCU.G
Ga0265742_1065119/361-273    ...U.UGUG.C.G.GGAAACAUA.G
Ga0316035_113756/173-88      ...G.UGGU.A.C.UGAAGACC..G
Ga0206355_1134705/241-332    ...UCG.U.AUAC.A.A.UGAAAACU..G
Ga0180107_1031754/4443-4551  ...UG.G.UAAC.G.G.AGAAGACU..G
Ga0138292_1031932/14-100     ...A.AUUC.A.G.AGAAGAUGCAA
Ga0316029_100807/1303-1197   ...UCUAG...AAGGCCA..........CCC.A.G.GGAAGCAAU.U
Ga0184568_114257/261-413     ...C.G.CUCA.A.G.AGAAGAAGC.G
Ga0242647_1005188/73-152     ...U.C.G.GGAAACAG..G
Ga0153880_1340646/556-457    ...CGAACUCUA...U.U.UAAG.A.U.UGAAGACA..A
Ga0307484_101303/704-866     ...GAGUGCU.UAUCCUU...CCACUACC...G.A.AGUA.G.G.AGAAGAAU..C
Ga0242641_1025149/251-89     ...AGUACC..CAUCCUU...CCACUACC...G.A.AGUA.G.G.AGAAGAAU..C
Ga0242652_1002960/413-574    ...A.......UCCACUACC...G.A.AGUA.G.G.AGAAGAGU..C

                             ...>>>>>>>.>>>>>>>>.>.>>>>>>>>>>>>>>>>....>>>>>....>>>.>.>>>>.>.>.....>>>>..<
                             ...2222.2.2.....2222..2
                             ---○◦◦●-●-G-RGAAG●●●--R
```

```
Ga0265754_1007706/448-552      ......A........C.CUGU.A.G.AGAGGGCA..G
Ga0157562_1164332/672-560      ......AACC........AGG.U.G.GGAAGACC..C
Ga0157569_1058012/474-586      ......AACC........AGG.U.G.GGAAGACC..C
Ga0307921_1027128/160-73       ......G.UUCU.C.G.GGAAGUCA..G
Ga0138279_1083071/419-326      ......GGAUCGGUG........CUA.C.G.GGAAGCAG..G
Ga0127502_10704012/113-7       ......UGUUGGCCA........CCC.G.A.UGAGGGAU..U
Ga0222728_1000095/4541-4435    ......UGUUGGCC.......ACCC.A.G.UGAGGACA..U
Ga0265750_1166784/261-415      ......CC.A.GCUUCAAA............GACGUAUG..CC.....UUG.U.G.AGAAGAAU..A
Ga0310115_109341/244-400       AA......GACGUAUG..CC.....UUG.U.G.AGAAGAAU..A
Ga0265756_158829/72-188        ......GAAAUA..........AUC.C.G.GGAAGUCA..U
Ga0184583_104740/342-477       ......C.UGUG.A.G.AGAAGGAA..G
Ga0180107_1215312/308-184      ......GCAGUA.......CCUA.U.G.AGAAGGAA..C
Ga0265742_1013613/579-453      ......UCACUCG.....ACGUC...........CCU.G.G.CGAAGACUC.G
Ga0265748_110851/279-405       ......UCACUCG.....ACGUC...........CCU.G.G.CGAAGACUC.G
Ga0265752_120463/277-403       ......UCACUCG.....UCGUC...........CCU.G.G.CGAAGACUC.G
Ga0265759_115288/4-102         ......GCUCU........A.CCUG.U.G.AGAGGUCA..A
Ga0242646_1001122/826-995      ......UGCUACAU.G.UCUCUGGAUCCUUCUC...AGCCGCC......GU.G..GCG.U.G.AGAAGAUUC.U
Ga0157593_1101573/244-147      ......C.UCAU.G.G.GGAAGUCA..G
Ga0184597_102941/191-111       ......A.U.ACAC.U.G.GGAAGUUC..G
Ga0184597_115944/155-80        ......A.A.ACCA.G.A.AGAAGAUG..G
Ga0265742_1051722/222-295      ......A.GGCC.G.A.AGAAGCAC..G
Ga0180107_1229893/426-318      ......AUC....G.A.UUUC.A.A.UGAAGACU..G
Ga0242654_11312609/202-83      ......CCACUAC..........U.C.C.G.GGAAGUUU..G
Ga0187793_1464320/616-535      ......GCC.A.G.GGAAGUGAC.C
Ga0187794_1206392/88-6         ......GCC.A.G.GGAAGUGAC.C
Ga0316034_129624/151-284       ......GCC...........UCU.G.G.AGAAGGAUA.U
Ga0184576_119948/531-664       ......GCC...........UCU.G.G.AGAAGGAUA.U
Ga0184568_109070/428-286       ......U.GCAA.A.G.UGAAGAAU..C
Ga0265756_100717/910-1050      ......GGGAC.....UAU.C.GCGU.U.G.UGAAGAAU..C
Ga0179955_1195671/481-563      ......GGUG.C.A.UGAAGCAU..G
Ga0184579_120620/502-584       ......G.GUUU.C.G.GGAAAUAG..U
Ga0127502_10711632/191-294     ......AUC.....G.UAUC.U.G.GGAAGAUA..G
Ga0310119_125223/193-319       ......AUCGC.....ACU.A.AUGG.C.A.AGAAGAGAUUG
Ga0307482_1064439/425-316      ......GGUCUU......G.U.UGAU.A.G.AGAGGAUUC.G
Ga0307484_102002/698-552       ......C.UUGU.A.G.AGAAGGAAU.U
Ga0265741_107029/232-320       ......A.G.GGAAGCUG..C
Ga0247515_120448/91-178        ......A.G.GGAAGCUG..C
Ga0265759_103410/556-638       ......A.G.GGAAGCUG..C
Ga0247534_101509/435-523       ......A.G.GGAAGCUG..C
Ga0310102_119240/132-46        ......U.A.G.GGAAGCUG..C
Ga0265742_1084621/112-29       ......A.G.GGAAGCUG..C
Ga0310114_135156/99-185        ......A.G.GGAAGCUG..C
Ga0265752_102595/694-607       ......A.G.GGAAGCUG..C
Ga0265756_105276/677-589       ......A.G.GGAAGCUG..C
Ga0153880_1339022/280-371      ......A.G.GGAAGCUG..C
Ga0247518_120275/202-289       ......A.G.GGAAGCUG..C
Ga0265752_100023/1618-1523     ......CUGCU............AU.G.G.GGAAGAUA..G
Ga0307484_102966/353-250       ......GGCGGGU......GGCUA............CAU.G.G.GGAAGUUA..U
Ga0265752_101001/521-687       ......GGCUCUG.GU.....G.U.CUCCCAUAACCUGGCU...UAAGGAGUGU....G.C.UCAA.G.C.GGAAGAAU..G
Ga0184593_121910/718-622       ......AUUU.........ACU.A.G.CGAAGCAUU.G
Ga0184602_103319/96-192        ......UU..........ACU.A.G.CGAAGCAUU.G
Ga0247528_108722/131-33        ......UU..........ACU.A.G.CGAAGCAUU.G
Ga0265757_143567/145-70        ......G.AAGC.A.G.GGAAGACG..G
Ga0184592_121084/378-271       ......CGCGUG...UUGGCCA...........CCC.G.G.UGAGGCAU..A
Ga0316037_172473/89-196        ......CGCGUG...UUGGCCA...........CCC.G.G.UGAGGCAU..A
Ga0307921_1011071/674-791      ......GAGACUAG....U.A.GUCA.A.C.UGAAGUCA..G
Ga0265744_124024/357-288       ......GAC.C.G.AGAAGUAG..G
Ga0307482_1000291/592-698      ......UCU.A..GGG.U.U.GGAGGACA..G
Ga0184568_108869/934-1072      ......G.....GAGUGACC.......AACU.U.G.AGAAGAAGG.A
Ga0138276_1121602/329-410      ......C.UCGC.U.G.AGAAGCAG..G
Ga0138278_1041650/183-102      ......A.U.UCGC.U.G.AGAAGCAG..G
Ga0138277_1137481/570-489      ......U.UUGC.U.A.AGAAGCAG..G
Ga0138278_1041788/293-378      ......U.UUGC.U.G.AGAAGCAG..G
Ga0138275_1223952/371-283      ......U.UUGC.U.G.AGAAGCAG..G
Ga0138277_1142657/273-361      ......U.UUGC.U.G.AGAAGCAG..G
Ga0307482_1202605/45-154       ......AC....GG.U.CAUA.U.G.GGAAGAAAU.G
Ga0180107_1017343/1163-1243    ......CAG.U.G.AGAGGCAG..G
Ga0157560_1170105/128-245      ......AAGAC.........G.CUGU.A.G.GGAAGCCG..G
Ga0316030_137115/325-257       ......A.C.G.GGAAGAUC..G
Ga0316046_103474/576-502       ......GGG.A.G.GGAAGUAA..G
Ga0265754_1141843/290-185      ......GCGGCUA......G.G.AUCU.G.G.GGAAGACU..G
Ga0222729_1080556/362-213      ......UAGGUAACU...........A.G.GGAGGAUU..A
Ga0265740_1020297/298-150      ......GGAUGAA.AUAUAUUC.A.UCCA..........UU...CUCGUGUA.......UUGC.A.A.GGAAGACA..A
Ga0157569_1080558/455-605      ......AAUCGAAC........GCGC.G.G.AGAAGUCGU.G
Ga0307484_101264/723-941       ......UGUGC...........UCAA.G.G.AGAAGAAGU.G
Ga0213850_1513954/279-196      ......CCC.G.GACGAAGUCC..G
Ga0316053_117700/109-179       ......AUG.A.A.GGAAAUUA..G
Ga0184582_101997/109-1         ......GG.....GAACCACAA......GAAU.C.A.CGAAGCUC..G
Ga0184602_109114/416-317       ......GU.G.CGCA.C.A.UGAAGUGAA.G
Ga0153880_1354682/355-454      ......GU.G.CGCA.C.A.UGAAGUGAA.G
Ga0184602_110257/415-316       ......GU.G.CGCA.C.A.UGAAGUGAA.G
Ga0307484_149222/136-234       ......ACC.........A.C.ACUA.U.G.GGAAGUAC..A
Ga0184572_111224/187-319       ......A....GGU.U.UAGA.U.G.GGAAGGUA..G
Ga0138278_1022898/219-127      ......GACCA............G.A.G.GGAAGACA..G
Ga0184577_108791/182-295       ......UGAGG.......G.A.GCGU.C.G.CGAAGACA..A
Ga0265748_107452/280-387       ......UGA.C..AUG.U.G.GGAAGACU..A
Ga0180107_1164033/560-431      ......GGUCCUCAUUCAAGAG.U.UUGCA.................GCCAA.....G.C.GCUA.A.G.AGAAGACU..U
Ga0032354_1086862/688-579      ......CAGACC..UUC.C.GAUU.A.G.GGAAGGGG..C
Ga0265751_100560/274-382       ......AAG.......CGAU.A.G.GGAAGAUG..U

                               ......>>>>>>>.>>>>>>>>.>.>>>>>>>>>>>>>>>>.....>>>>>.....>>>.>.>>>>.>.>.....>>>>..<
                               ......2222.2.2.....2222..2
                               ------oooo•-•-G-RGAAG•••--R
```

```
Ga0180107_1045142/740-541          UCCUGGAUUCACAACGGGCCAGCAGCGCAAGGCAU.............................................................................GCGC....CG.G.AGUG.A.A.AGAAGAAA..U
Ga0213850_1296465/305-234          ...........................................................................................................................U.U.CGGC.U.U.GGAAGCUU..U
Ga0265757_111747/356-286           ...........................................................................................................................U.U.CAGC.U.U.GGAAGCUU..U
Ga0247553_104889/634-563           ...........................................................................................................................U.U.CAGC.U.U.GGAAGCUU..U
Ga0265751_102572/231-125           ................................................................................................................GGAUUAU...AU.C..AUA.GCG.GGAAGAUA..A
Ga0265758_100017/4028-4134         ................................................................................................................GGAUUAU...AU.C..AUA.GCG.GGAAGAUA..A
Ga0180107_1307934/226-315          .................................................................................................................................CC.C.G.GGAAGAAA..A
Ga0184572_112077/359-259           .............................................................................................................................G.UGGU.G.G.GGAAGAGA..A
Ga0213850_1371453/186-298          ...........................................................................................................................U.C.UGUU.U.G.GGAAGACA..C
Ga0213850_1469439/119-231          ..........................................................................................................................UU.C.UCUU.U.G.GGAAGACA..C
Ga0247535_113123/229-119           ..........................................................................................................................CG.A.GGGG.G.G.GGAAGCUG..C
Ga0316036_112404/482-376           ...........................................................................................................................C.G.AGGG.A.G.GGAAGCUG..C
Ga0179943_1118543/263-176          ...............................................................................................................................GGGU.G.A.GGAAACGA..U
Ga0180108_1207316/715-601          ..................................................................................................................................U.C.G.GGAAGAGA..G
Ga0184578_120646/150-246           ................................................................................................................AGAAUAGA........ACU.U.G.GGAAGCUG..G
Ga0127503_10678853/419-333         .............................................................................................................................G.AGUC.A.G.GGAAGGCA..G
Ga0307919_1065410/76-162           .............................................................................................................................G.AGUC.A.G.GGAAGGCA..G
Ga0180108_1209503/1932-2029        ................................................................................................................GUCUG......A.G.UGUU.A.G.GGAAGACC..G
Ga0180108_1022680/3902-3998        ................................................................................................................AACCG......A.U.CUUU.A.G.GGAAGACU..G
Ga0316031_107021/319-214           ....................................................................................................................UAG......G.AUGG.U.G.AGAAGAUAC.G
Ga0247527_116942/161-88            ................................................................................................................................UGU.C.G.CGAAGCUU..G
Ga0307484_100434/1330-1210         .......................................................................................................................AA..G.G.UCAU.C.G.GGAAGACA..G
Ga0307482_1160392/324-400          .................................................................................................................................AG.G.G.GGAAGAAC..G
Ga0316033_105623/339-253           ...............................................................................................................................AAGC.A.G.GGAAAGCG..G
Ga0265754_1037910/322-179          .....................................................................................................................UGUG....C.UCUA.A.C.GGAAGAGA..C
Ga0307482_1048753/383-528          GUAUGCU........................................................................................................................CUGG.U.G.AGAAGGACU.G
Ga0265752_102479/223-81            ................................................................................................................AUGUUAU.........AGC.A.A.GGAAGACU..G
Ga0265746_1084801/20-158           ................................................................................................................AUGUUAU.........AGC.A.A.GGAAGACU..G
Ga0265756_107066/520-660           ................................................................................................................AUGUUAU.........AGC.A.A.GGAAGACU..G
Ga0265742_1022009/340-200          ................................................................................................................AUGUUAU.........AGC.A.A.GGAAGACU..G
Ga0265748_106902/213-353           ................................................................................................................AUGUUAU.........AGC.A.U.GGAAGACU..G
Ga0180052_116799/419-501           ................................................................................................................................AGC.G.A.GGAAGACU..G
Ga0115594_1106220/441-334          ................................................................................................................GUGUC............CU.A.G.GGAAGACC..G
Ga0247534_117029/65-195            ...................................................................................................CUGGG.........AUCGAUG........AGC.A.G.GGAAGCGU..A
JGI12272J11983_1072695/40-150      ................................................................................................GCGCAAUCCCAAG...CGGGUAGGA.......GCG.A.G.AGAAGAGAU.G
Ga0247552_100007/4311-4420         ................................................................................................................GGGUGAC...GG.G.GUGU.A.G.GGAAGAUCA.G
Ga0184596_107732/313-205           ................................................................................................................GGGUGA...UGG.G.GUGU.A.G.GGAAGAUUA.G
Ga0247542_100007/609-500           ................................................................................................................GGGUGA...UGG.G.GUGU.A.G.GGAAGAUUA.G
Ga0247534_100047/1899-2008         ................................................................................................................GGGUGA...UGG.G.GUGU.A.G.GGAAGAUUA.G
Ga0184598_129111/512-403           ................................................................................................................GGGUGA...UGG.G.GUGU.G.G.GGAAGAUUA.G
Ga0184597_128933/573-464           ................................................................................................................GGGUGA...UGG.G.GUGU.A.G.GGAAGAUUA.G
Ga0184582_108821/1050-1159         ................................................................................................................GGGUGA...UGG.G.GUGU.A.G.GGAAGAUUA.G
Ga0247541_100010/3268-3377         ................................................................................................................GGGUGA...UGG.G.GUGU.A.G.GGAAGAUUA.G
Ga0184580_121439/573-464           ................................................................................................................GGGUGA...UGG.G.GUGU.A.G.GGAAGAUUA.G
Ga0265756_101275/650-541           ................................................................................................................GGGUGA...UGG.G.GUGU.A.G.GGAAGAUUA.G
Ga0184575_101094/586-477           ................................................................................................................GGGUGA...UGG.G.GUGU.A.G.GGAAGAUUA.G
Ga0184594_103311/207-316           ................................................................................................................GGGUGA...UGG.G.GUGU.A.G.GGAAGAUUA.G
Ga0247554_100006/1592-1485         ................................................................................................................GGGUGA...UGG.G.GUGU.A.G.GGAAGAUUA.G
Ga0247522_108409/684-554           .............................................................................................UCCGCUGGCUUCAG.....GGUGUGUG.....C.CUAG.A.G.AGAAGGAUU.G
Ga0247534_100003/3147-3245         ................................................................................................................AACUC........G.UUGU.G.G.GGAAGAUCC.G
Ga0265751_100393/1594-1698         .....................................................................................................................A.....G.G.GUGU.A.G.AGAAGAAAU.G
Ga0265751_124807/392-283           .....................................................................................................................AA......G.UAAU.G.G.GGAAGAUA..U
Ga0310107_144222/327-436           .....................................................................................................................AA......G.UAAU.G.G.GGAAGAUA..U
Ga0265743_103007/946-842           .....................................................................................................................A.....G.A.AUAG.A.G.UGAAGAAUA.G
Ga0316038_107364/55-195            ...........................................................................................G.UUUCUUCGUGUUGAUU...AG...ACA.......CAAG.G.G.GGAAGCGG..G
Ga0184593_123391/157-297           ...........................................................................................G.UUUCUUCGUGUUGAUU...AG...ACA.......CAAG.G.G.GGAAGCGG..G
Ga0222728_1003482/1262-1107        .............................................................................ACAGG.GAA.CC.C.U.ACG...............CCUACG.......GCGC.A.G.AGAAGAACU.G
Ga0242654_10701525/53-296          CCGGACCUCUCCAGCUCACUGGUGGUGGUACUUGCCUGACUGGAAAACCAACCAGUUGGGUAAC........................................AACA....CCCCUGCC..GA.A..GCA.G.G.AGAAGAUUC.U
Ga0242654_11172691/285-48          ACUCCGGACCUCUUCAGCUCACUGGUGGUGGUACUUGCCUGGCUGGGAAACCAGUUGGGUCCAUCA..............................................CCUCUGCC..GA.A..GCA.G.G.AGAAGAUUC.U
Ga0316038_100042/1676-1569         .....................................................................................................................AGAG.......CUC.A.GUGGAAGAGU..A
Ga0307921_1043977/91-164           ................................................................................................................................GUU.U.G.GGAAGAUA..G
Ga0079039_1293848/95-22            ................................................................................................................................GCA...G.GGAAGCAG..G
Ga0247534_101560/228-122           .....................................................................................................................A....UG.G.UCAG.G.G.AGAAAACC..G
Ga0153880_1379927/66-144           ................................................................................................................................ACC.G.G.GGAAGAAC..G
Ga0153880_1391790/62-140           ................................................................................................................................ACC.G.G.GGAAGAAC..G
Ga0307482_1048660/422-625          UCAAAGGGUGUG.................................................................................................................C.CAAC.G.G.AGAGGAAG..G
Ga0180108_1022680/4248-4153        .................................................................................................................CUCU......U.G.GUUA.U.G.GGAAGUUC..A
Ga0247516_125012/316-428           ......................................................................................................................GG.....U.UUGA.C.G.AGAAGAAUG.U
Ga0242652_1038498/365-470          ....................................................................................................................G........A.CCAC.A.G.AGAAGAUUA.G
Ga0210323_1050281/92-187           .....................................................................................................................AAC........CAG.A.G.AGAGCAUA..G
Ga0224712_10542545/154-227         .................................................................................................................................AG.U.G.GGAAGUUA..G
Ga0184602_130775/1275-1431         .....................................................................................................................AUG...C.U.CAGA.G.U.GGAAGUAU..C
Ga0206355_1311803/144-334          ....................................................................................................CCC.........AGUUG....UGG.A.UUAG.A.C.AGAAGACA..G
Ga0310119_152175/242-389           ................................................................................................................GUCCUAUG...C.U.AGAU.U.G.AGAAGAGCA.G
Ga0255059_10691632/166-76          .................................................................................................................CUCUCAA........CCA.C.A.UGAAGAUA..G
Ga0255062_11230321/200-110         .................................................................................................................UCUCGA.........CCA.C.A.UGAAGAUA..G
Ga0265743_143797/241-325           ....................................................................................................................................G.G.GGAAGACA..G
Ga0247534_117055/74-212            .....................................................................................................................ACAA.CUCUU.CCAA.A.G.GGAAGAGG..U
Ga0213850_1282564/267-350          .....................................................................................................................AG..........GU.G.G.AGAGGCUC..G
Ga0315826_123252/262-347           .....................................................................................................................AG..........GU.G.G.AGAGGCUC..G
Ga0315817_133437/498-413           .....................................................................................................................AG..........GU.G.G.AGAGGCUC..G
Ga0184577_116723/415-311           .............................................................................................................................G.GAAU.G.G.AGAAGAUCC.C
Ga0184576_109717/432-536           .............................................................................................................................G.GAAU.G.G.AGAAGAUCC.C
Ga0184572_113323/230-300           ...............................................................................................................................GAAA.G.G.AGAGGACC..U
Ga0179935_1139075/165-78           ...............................................................................................................................AUCG.A.G.GGAAGUGG..G
Ga0179944_1246019/249-162          ..................................................................................................................AGU...........CUU.A.G.GGAAGUGA..G
Ga0213850_1307525/101-186          ...............................................................................................................................AUCG.C.G.GGAAGCCUAGG
Ga0222728_1062510/166-66           .........................................................................................................................AGU.C.UGUG.A.A.AGAAGCAG..G
Ga0265743_101198/448-619           .......................................................................................CCC.UAGGCUCUAUUACCUGGC...CCUAC.....CG.G.GCUG.G.A.AGAAGACU..U

                                   ...........................................................................>>>>>>>.>>>>>>>>.>.>>>>>>>>>>>>>>>....>>>>>....>>>.>.>>>>.>.>.....>>>>..<
                                   ...................................................................................................................................2222.2.2.....2222..2
                                   -------------------------------------------------------------------------------------------------------------------------------○◦◦●-●-G-RGAAG●●●--R
```

```
Ga0180108_1209503/2438-2343   ..........................................................................................................................CCC.......U.A.GUCA.C.G.GGAGGUUA..U
Ga0184583_103998/587-740      ................................................................................GGGGAUC.GGUGGUUA.C.GGCUUCAAAGCGUGUG........CC............CGG.U.G.AGAAGAAUA.G
Ga0184595_100702/414-262      ................................................................................GGGGAUC.GGUGGUUA.C.GGCUUCAAGGCGU........GUGCC............CGG.U.G.AGAAGAAA..G
Ga0184592_125105/67-219       ................................................................................GGGGAUC.GGUGGUUA.C.GGCUUCAAGGCGU........GUGCC............CGG.U.G.AGAAGAAA..G
Ga0224712_10002011/1564-1454  ................................................................................G.......................................GGU.......AUG.G.UAUA.A.G.GGAAGACU..A
Ga0316049_114344/181-288      ......................................................................................................................................G.UGUC.A.G.GGAAGCAC..G
Ga0184576_110594/6-113        ............................................................................................................UCGAAGG..........U......U.G.UGUC.A.G.GGAAGCAC..G
Ga0316037_112281/325-219      ...............................................................................................................UCGA.....AGGUU.........G.UGUC.A.G.GGAAGCAC..G
Ga0316046_116376/278-384      ..............................................................................................................AUCGA.....AGGUU.........G.UGUC.G.G.GGAAGCAC..G
Ga0247534_102735/124-18       ..............................................................................................................AUCGA.....AGGCU.........G.UGUC.A.G.GGAAGCAC..G
Ga0247540_102569/210-104      ..............................................................................................................AUCGA.....AGGUU.........G.UGUC.A.G.GGAAGCAC..G
Ga0247529_116306/255-362      .............................................................................................................AUCGAA.....GGUUG...........UGUC.A.G.GGAAGCAC..G
Ga0184568_104528/922-1000     .........................................................................................................................................AGU.A.G.GGAAGACU..A
Ga0184578_112031/156-44       ..........................................................................................................................GGCA......C.A.UUCG.G.C.UGAAGCUG..G
Ga0247517_121471/387-499      ..........................................................................................................................GGCA......C.A.UUCG.G.C.UGAAGCUG..G
Ga0184569_108885/200-88       ..........................................................................................................................GGCA........C.AUAG.G.C.UGAAGCGA..G
Ga0247521_112559/317-205      ..........................................................................................................................GGCA........C.AUAG.G.C.UGAAGCGA..G
Ga0247539_101233/414-526      ..........................................................................................................................GGCA........C.AUAG.G.C.UGAAGCGA..G
Ga0265756_100485/1108-1000    ........................................................................................................................AGGAUAGUC........GUG.C.G.GGAAGCCA..G
Ga0265751_100417/1480-1588    ........................................................................................................................AGGAUAGUC........GUG.C.G.GGAAGCCA..G
Ga0213850_1295854/263-370     ....................................................................................................................................A.C.G.CG.U.A.AGAAAUGG..G
Ga0307919_1029784/253-144     ....................................................................................................................................A.C.G.CA.U.G.AGAAAUGG..G
Ga0307921_1042549/343-452     ....................................................................................................................................A.C.G.CA.U.G.AGAAAUGG..G
Ga0307484_102966/727-825      ........................................................................................................................GGGGC...........CUUU.A.G.GGAAGACU..A
Ga0184588_131258/156-253      ...............................................................................................................GAUG...UUGUCAAA...........GCA.U.G.CGAAGCUC..C
Ga0224712_10010317/1973-2082  ...................................................................................................................................GA.C.CUUA.G.G.GGAAGACA..G
Ga0224712_10601975/393-502    ...................................................................................................................................GA.C.CUUA.G.G.GGAAGACA..G
Ga0222728_1000118/2272-2196   ......................................................................................................................................G.GGUA.G.G.GGAAGAUC..G
Ga0115594_1200134/154-31      .........................................................................................................................GGGC...........GGUU.G.G.GGAAGACCA.G
Ga0115595_1153837/320-197     ....................................................................................................................................G.C.GGCU.A.G.GGAAGACCA.G
Ga0255282_1067513/33-105      .........................................................................................................................................GGC.U.G.GGAAGCCG..G
Ga0242647_1005934/451-617     ................................................................................GUACAAU.GUGUGUAA.U.CCCUGGAUCC....CU.....CAGCCA....CCG.U.GGUG.G.A.AGAAGAUUC.U
Ga0307482_1449537/408-223     ................................................................................GGCUCUG.CUACUCGC.A.CGAGUAACCGACAAGA...UC....CCC...UGC.U.UGCA.G.G.AGAAGAAUU.G
Ga0115594_1022316/346-239     .........................................................................................................................GCA.............CCU.A.G.GGAAGACC..G
Ga0115595_1072788/334-441     .........................................................................................................................GCA.............CCU.A.G.GGAAGACC..G
Ga0184568_108419/50-147       ........................................................................................................................AUGGU......GG.C.ACUA.A.G.GGAAGACU..G
Ga0247534_100007/116-225      ...................................................................................................................................GA.A.UCGA.U.G.GGAAGAUU..A
Ga0184569_109084/220-111      ...................................................................................................................................GA.A.UCGA.U.G.GGAAGAUU..A
Ga0316033_100036/207-98       ..................................................................................................................................AGA.A.UCGA.U.G.GGAAGAUU..A
Ga0184578_112042/249-140      ..................................................................................................................................AGA.A.UCGA.U.G.GGAAGAUU..A
Ga0184594_123034/256-94       ........................................................................................................................................CGAA.G.A.UGAAGACG..U
Ga0256798_1305521/221-305     ........................................................................................................................................CAAC.C.G.GGAAGAGG..C
Ga0184595_116681/414-266      .................................................................................................G.CGACUUGAUACGA.CU...UCGAGGCAUGU...G.C.UCUG.A.G.GGAAGAACG.G
Ga0184568_105547/129-239      ........................................................................................................................................GUAA.G.G.AGAAGGUA..C
Ga0157559_1099797/360-209     ................................................................................UACGGUG.ACCCCCAG.U.G....................AGGCUC........C.CGCG.A.G.GGAAAACCU.A
Ga0157572_1089974/360-209     ................................................................................UACGGUG.ACCCCCAG.U.G....................AGGCUC........C.CGCG.A.G.GGAAAACCU.A
Ga0316037_108094/19-160       .............................................................................................................................AGU..AUA.G.AUAU.A.G.GGAAGAUA..G
Ga0316038_107116/33-174       .............................................................................................................................AGU..AUA.G.AUAU.A.G.GGAAGAUA..G
Ga0210323_1072445/691-543     ............................................................................................................CA..........GCGUUUGC........GUGG.U.G.CGAAGGAUU.G
Ga0307482_1000707/704-773     .........................................................................................................................................CGC.C.A.GGAAGCAC..C
Ga0079039_1671802/197-34      ...........................................................................................................................ACACUC..CC.A.GGAA.A.G.GGAAGACG..A
Ga0247533_103342/137-220      ..........................................................................................................................................CG.A.G.GGAAGAAA..G
Ga0157565_1016893/233-334     ......................................................................................................................................A.AGCU.A.G.GGAAGUAA..A
Ga0180047_1087048/281-180     ......................................................................................................................................A.AGCU.A.G.GGAAGUAA..A
Ga0157593_1006201/239-338     ......................................................................................................................................A.AGCU.A.G.GGAAGUAA..A
Ga0157560_1010344/357-456     ......................................................................................................................................A.AGCU.A.G.GGAAGUAA..A
Ga0316049_154526/155-74       ..........................................................................................................................AAUAUU.........CCU.G.G.GGAAGAGA..G
Ga0316035_177575/224-142      .............................................................................................................................UU.........UCCU.G.G.GGAAGAGA..G
Ga0316033_101029/151-49       ........................................................................................................................CGAUCAUA..CCA.U.CU...C.G.GGAAGCCA..G
Ga0206356_10600373/215-21     ........................................................................................................................CGGUCA..........CUUG.A.U.AGAAGACA..G
Ga0307482_1009758/562-669     .........................................................................................................................GG.UA.....UG.G.CGUU.G.G.AGAAGAUCU.G
Ga0213850_1245299/581-409     CGU..........................................................................................................................ACGA.GUG.A.GCUA.C.G.AGAAGUCA..G
Ga0180107_1134582/382-542     ................................................................................GGCUUGC.AGA.................CCUUCAU.....UGGAAAGUC.CUA.G.GCUC.A.G.GGAAGACA..C
Ga0307484_100970/951-1026     ........................................................................................................................................GCGU.C.G.CGAAGCUG..U
Ga0307919_1034281/527-451     ......................................................................................................................................G.UCUU.A.G.GGAAAUGG..G
Ga0307921_1020694/406-483     ......................................................................................................................................G.UCUU.A.G.GGAAAUGG..G
Ga0316033_105623/3-98         ...........................................................................................................................GCAU.........CGUG.U.G.GGAAGACU..G
Ga0265743_111644/592-512      .........................................................................................................................................CGC.C.G.UAAAGACA..G
Ga0265755_108708/373-453      .........................................................................................................................................CGC.C.G.UGAAGACA..G
Ga0316038_121313/184-264      ..........................................................................................................................................GC.C.G.UGAAGACA..G
Ga0265756_109326/116-196      .........................................................................................................................................CGC.C.G.UGAAGACA..G
Ga0265759_104327/339-243      .........................................................................................................................................GGG.A.G.GGAAGAGU..G
Ga0316035_144545/195-301      ......................................................................................................................................G.UCAA.U.G.GGAAGAGG..G
Ga0265743_108998/150-253      .............................................................................................................................A......G.G.AUGU.G.G.AGAAGAAAU.G
Ga0153880_1067318/492-566     .........................................................................................................................................UAC.G.G.CGAAGAGU..C
Ga0180044_1047678/444-368     ......................................................................................................................................A.ACCU.G.G.AGAAGUCU..G
Ga0157564_1065218/271-353     ....................................................................................................................................U.G.CACA.A.G.GGAAGUCU..G
Ga0180044_1069332/269-187     ....................................................................................................................................U.G.CACA.C.G.GGAAGUCU..G
Ga0242647_1014694/423-336     ........................................................................................................................GCUGCAACC........AAC.U.G.GGAAAAUC..G
Ga0180108_1143860/199-116     ........................................................................................................................AAUCUA.............C.C.U.AGAAACAG..G
Ga0265744_100073/1235-1401    CACU.......................................................................................................................CCGCC....G.U.AGCA.G.A.GGAAGACA..U
Ga0307482_1054608/326-205     ........................................................................................................................................UAAG.C.G.GGAAGCUG..G
Ga0242646_1008525/229-119     ........................................................................................................................................UAAG.C.G.AGAAGCUG..G
Ga0153880_1260556/441-589     .................................................................................G................................U.....AGUGUAUAA........GCU.U.G.GGAAGAGC..G
Ga0127503_10814644/496-352    ................................................................................GGUGG........................................ACCA.UCC.A.AUCA.U.G.GGAAGAGA..G
Ga0326631_105052/171-77       .............................................................................................................................AAAG.UUG.C.AAGG.U.A.CGAAGUGA..U
Ga0307920_126542/349-261      ...................................................................................................................................GC.G.AGCG.G.G.GGAAGAAA..U
Ga0247533_100162/1643-1535    ..........................................................................................................................GGUAGGA........CUG.G.A.AGAAGAAAU.G
Ga0316047_100015/734-626      ..........................................................................................................................GGUAGGA........CUG.G.A.AGAAGAAAU.G

                              ................................................................................>>>>>>>.>>>>>>>>.>.>>>>>>>>>>>>>>>>.....>>>>>.....>>>.>.>>>>.>.>.....>>>>..<
                              ........................................................................................................................................2222.2.2.....2222..2
                              ----------------------------------------------------------------------------------------------------------------------------------------○○○●-●-G-RGAAG●●●--R
```

```
Ga0265743_101537/1064-984      ....................................................................................................................................CG.U.G.GGAAACAU..G
Ga0316030_100208/805-700       ....................................................................................................................GACUA.....G.G.AUGA.U.G.AGAAGAUAU.G
Ga0316050_103193/566-460       ....................................................................................................................GAC.....UAG.G.AUGA.G.G.AGAAAAUA..G
Ga0184601_113231/247-149       ........................................................................................................................AU........GCAU.G.G.CGAAGACA..G
Ga0307350_148584/139-224       .....................................................................................................................................A.U.G.GGAAGAGU..G
Ga0307482_1018929/1182-1081    ................................................................................................................................C.GUAU.GCG.GGAAGAGG..G
Ga0307482_1000262/4800-4699    ................................................................................................................................C.GUAU.GCG.GGAAGAGA..G
Ga0316047_100860/1040-888      ....................................................................................................................GUUA......G.G.AGCC.G.G.AGAAGAAU..C
Ga0242654_10068518/113-218     .......................................................................................................................AGU........GAGU.C.G.GGAAACACC.G
Ga0222729_1010500/11-117       .......................................................................................................................AGU........GAGU.C.G.GGAAACACC.G
Ga0307484_101172/602-495       .......................................................................................................................AGU........GAGU.C.G.GGAAACACC.G
Ga0242647_1002406/915-1021     .......................................................................................................................AGU........GAGU.C.G.GGAAACACC.G
Ga0307483_1000720/1178-1064    .......................................................................................................................AAU..ACG.A..GUC.A.G.CGAAGAGA..G
Ga0307921_1022947/450-369      .....................................................................................................................................G.U.G.GGAAGACC..G
Ga0153880_1432604/289-395      .......................................................................................................GGCGUG...UAGGGCU.............CC.A.A.GGAAGACC..G
Ga0247533_101442/511-432       ..................................................................................................................................UUAC.U.G.GGAAGACA..A
Ga0242647_1002473/759-521      ACUCUGGUAGGUCUUCACUCAAUCGGUGGCGGACUUGCCUGACUGUUAAAUCAGUUGGGUCACCAACCGC............................................CUGCC.......G.U.AGCA.G.G.AGAAGAUUC.A
Ga0256352_1087084/23-118       ...................................................................................................................................AGG.A.G.AGAAGACC..G
Ga0157562_1157806/189-74       ...................................................................................................................................AGG.A.G.AGAAGACU..G
Ga0153880_1457361/236-121      ................................................................................................................................U.AAGG.A.G.GGAAGACU..G
Ga0157559_1128929/201-87       ....................................................................................................................................GG.U.G.AGAAGACU..G
Ga0157566_1012736/497-384      ...................................................................................................................................AGG.A.G.AGAAGACU..G
Ga0157579_1155702/323-436      ...................................................................................................................................AGG.A.G.AGAAGACU..G
Ga0316036_116159/257-326       ....................................................................................................................................GU.C.G.GGAAAAGG..G
Ga0180047_1032966/152-37       ...................................................................................................................................AGG.A.G.AGAAGACU..G
Ga0184587_125934/78-152        ...................................................................................................................................GGU.G.G.AGAGGCAU..G
Ga0179934_1155244/114-26       .......................................................................................................................................C.G.GGAAAUCC..G
Ga0307482_1623775/241-347      ..................................................................................................................GACCU.......G.U.UUCU.U.G.GGAAGACU..A
Ga0307484_101582/1018-1124     ..................................................................................................................GACCUG........U.UUCU.U.G.GGAAGACU..A
Ga0307484_111921/444-550       ..................................................................................................................GACCUG........U.UUCU.U.G.GGAAGACU..A
Ga0184587_113157/56-168        ..................................................................................................................GUCCUAUGC.U.....CAGA.G.G.AGAAGCAUA.A
Ga0255228_1272153/272-192      ....................................................................................................................................GA.U.G.GGAAGCUA..G
Ga0153880_1325448/483-575      ....................................................................................................................................GU.G.A.GGAAGACG..G
Ga0157565_1130498/91-183       ....................................................................................................................................GU.G.A.GGAAGACG..G
Ga0265758_129208/41-120        ...................................................................................................................................UUU.C.G.GGAAGACG..G
Ga0265756_146329/172-94        ..................................................................................................................................GUUU.C.G.GGAAGACA..A
Ga0115594_1180137/311-161      .............................................................................................GCGCCCCGGGCCCC.......CCUCC...........UCGC.G.G.AGAAGAGC..G
Ga0265744_100861/398-567       ........................................................................................................AUUAC...UCGACCCUAC...CG.G.GUUG.G.A.AGAAGACU..U
Ga0180107_1285918/13-113       .....................................................................................................................ACAAGA.GCC.C.UUAU.U.G.GGAAGCUA..G
Ga0247519_103291/1381-1460     ...................................................................................................................GUCAC............UC.C.U.GGAAGCAG..U
Ga0184603_106561/141-62        ...................................................................................................................................CUU.C.U.GGAAGCAG..U
Ga0247551_100996/477-556       ...................................................................................................................................CUC.C.U.GGAAGCAG..U
Ga0184590_126350/300-380       .....................................................................................................................UCA...........CUC.C.U.GGAAGCAG..U
Ga0265756_131580/159-58        .............................................................................................................................UA.G.CUCU.G.G.GGAAGAAG..G
Ga0307482_1000325/24-131       ..........................................................................GGUG.........................................AA.......G.AUAG.A.G.GGAAGAUC..G
Ga0242646_1001086/737-571      ..........................................................................UCAA.UG.UUUUCCGA.G.GAUCCUGCC.C..........CCGCC......GC.G..GCG.U.G.AGAAGAUUC.U
Ga0265747_132694/181-66        ....................................................................................................................................GG.G.A.GGAAGAAG..G
Ga0157593_1023702/238-314      ....................................................................................................................................CU.G.G.GGAAGACA..G
Ga0316053_103654/814-736       ....................................................................................................................................CU.G.G.GGAAGACU..G
Ga0247529_117158/253-331       ....................................................................................................................................CU.G.G.GGAAGACU..G
Ga0180107_1129325/244-60       UGCAUUGUCU..............................................................................................GACUG.....GAACCU......C.C.AUAA.G.G.AGAAGAACA.G
Ga0307482_1162833/232-148      ...................................................................................................................................CUG.C.G.GGAAGCCG..G
Ga0255267_1268184/91-184       ..........................................................................GGUG.........................................AU.....G.A.GGAG.G.G.GGAAGACA..G
Ga0307919_1064641/225-73       ..........................................................................UGGAACG.....GCCU.A.GC.........AGAUU...UAUGUGGA....CUA.A.GGAC.U.G.GGAAGAUG..G
Ga0307921_1075885/188-36       ..........................................................................UGGAACG.....GCCU.A.GC.........AGAUU...UAUGUGGA....CUA.A.GGAC.U.G.GGAAGAUG..G
Ga0265742_1021317/385-459      ................................................................................................................................U.GUCC.A.G.GGAGGACG..G
Ga0307483_1002304/791-713      ....................................................................................................................................CA.U.G.GGAAGCAC..G
Ga0307919_1165008/203-116      ....................................................................................................................................GG.G.G.GGAAGACU..G
Ga0210323_1093898/306-192      ...................................................................................................................................CUG.A.G.AGAAGAAC..G
Ga0265743_119695/104-184       ................................................................................................................................G.UGUU.C.G.GGAAGAGC..G
Ga0310118_104706/1022-1118     .......................................................................................................................................C.U.GGAAGUAC..A
Ga0307921_1005950/551-460      ..................................................................................................................AAUG.............CCC.U.G.GGAAGACU..G
Ga0242641_1014724/551-384      ..........................................................................CGCAUGU.UCGACUCU.C.......................UCACAAA....G.G.AGAU.G.A.AGAAGAACA.U
Ga0242641_1002366/499-331      ..............................................................................................CGCAUGUUCGACUCU...C..UCACAAA....G.G.AGAU.G.A.AGAAGAACA.U
Ga0242657_1007384/290-215      ...................................................................................................................................GGU.G.G.AGAGGCGAA.G
Ga0265757_113513/277-362       ..............................................................................................................................U.A.GUGG.GUA.GGAAGAAC..G
Ga0242646_1001007/790-609      ...................................................................................................UGUAUGGCUU.....CAUCCACC...CG...UGUG.G.G.AGAAGUAUA.C
Ga0316034_107719/314-205       ...................................................................................................................GGGUAGAU........AUG.A.G.GGAAGAUC..G
Ga0316039_119781/347-457       ...................................................................................................................GGGUAGAU........AUA.G.G.UGAAGAGAC.G
Ga0255295_1078742/236-408      ..................................................................................................................CUUCAA..........UUCC.U.G.GGAAGCUG..G
Ga0184603_150346/629-518       ...................................................................................................................GGGUAGGA........UUC.A.G.GGAAGCACU.G
Ga0180106_1187148/166-89       ..............................................................................................................................U.A.CUGG.U.G.GGAAGUCG..G
Ga0307482_1417159/374-296      ...................................................................................................................................AUA.U.G.GGAAGUCA..G
Ga0222728_1000095/4027-4133    ..............................................................................................................................G.A.GUUA.G.G.AGAAGAAAU.G
Ga0316030_105840/623-504       ......................................................................................UCAA.A.UGUCUACAAACCCCCC..........A............AA.G.G.AGAAGCUU..G
Ga0316033_110175/528-409       ......................................................................................................UCUACAA...........AC..CCC.C.CAAA.G.G.AGAAGCUU..G
Ga0326631_124620/105-175       .....................................................................................................................................A.G.G.AGAAGCUU..A
Ga0206356_11590518/224-68      ..................................................................................................................CACGAGCAC........CGC.G.C.GGAAGUCC..G
Ga0213850_1246156/171-47       ..................................................................................................................UUCCC............AAG.G.G.GGAAGAGG..G
Ga0184599_140450/516-588       ................................................................................................................................U.CAAU.C.C.AGAGGCAA..A
Ga0206356_11002907/329-148     ..................................................................................................................GAGGG.....CCG.G.UCUU.A.U.AGAAGACA..G
Ga0307482_1001469/2898-2828    ...................................................................................................................................GGU.U.G.GGAAGCAUU.G
Ga0157570_1089798/68-159       ................................................................................................................................G.AGGG.G.G.GGAAACACA.G
Ga0127503_11028958/51-130      ............................................................................................................................AUG.A.UAAA.A.U.AGAAGACU..G
Ga0157560_1126722/83-169       ............................................................................................................................AAA.C.UUAA.G.A.UGAGGCUA..G
Ga0138277_1028338/433-516      .....................................................................................................................................U.A.G.GGAAGAAG..G
Ga0265742_1001214/55-127       .....................................................................................................................................C.A.G.GGAAAACC..G
Ga0115594_1134035/652-542      ..................................................................................................................AGCAC.......G.A.ACAC.A.G.AGAAGACC..G
Ga0115594_1212468/8-118        ..................................................................................................................AGCAC.......G.A.ACAC.A.G.AGAAGACC..G

                               ..........................................................................>>>>>>>.>>>>>>>>.>.>>>>>>>>>>>>>>>>.....>>>>>.....>>>.>.>>>>.>.>.....>>>>..<
                               ..................................................................................................................................2222.2.2.....2222..2
                               ----------------------------------------------------------------------------------------------------------------------------------○●●●-●-G-RGAAG●●●--R
```

```
Ga0247533_100438/1040-888      ..........UGUUA......G.G.AGCC.G.G.AGAAGAAU..C
Ga0326631_117973/181-293       ..........GAUGCGAAA...........A.G.GGAAGAGG..G
Ga0184600_117644/32-108        ..........GAUC.U.G.GGAAACUA..G
Ga0184584_133603/317-393       ..........GAUC.U.G.GGAAACUA..G
Ga0316052_100527/264-188       ..........GAUC.U.G.GGAAACUA..G
Ga0184580_125947/153-229       ..........GAUC.U.G.GGAAACUA..G
Ga0247524_105844/561-637       ..........ACG...........AUC.U.G.GGAAACUA..G
Ga0184580_109821/480-551       ..........C...G.GGAAGCUA..G
Ga0184598_107135/96-25         ..........C...G.GGAAGCUA..G
Ga0316051_1015080/488-559      ..........G.GGAAGCUA..G
Ga0247524_121352/374-303       ..........GA.U.C.G.GGAAGCUA..G
Ga0179934_1197833/207-115      ..........GAUG...UUGUCAA...........CUG.U.G.GGAAGACU..G
Ga0316049_129960/371-262       ..........G.C.CUAG.G.G.GGAAGACA..G
Ga0265747_125301/405-306       ..........AG....AAG.A.G.GGAAGACG..U
Ga0157570_1034241/151-229      ..........UGC.A.G.GGAAGACA..G
Ga0157571_1064089/212-134      ..........UGC.A.G.GGAAGACA..G
Ga0242652_1041630/308-377      ..........AA.U.G.GGAAGUUA..G
Ga0307480_1006257/684-599      ..........GGGCC..........CGGG.C.G.GGAAGCCAU.G
Ga0310119_142698/346-430       ..........CU.G.G.GGAAAACG..U
Ga0307482_1074296/478-354      ..........CACAAUA........CUC.G.A.GGAAGUGUA.G
Ga0307483_1005049/26-150       ..........CACAAUA........CUC.G.A.GGAAGUGUA.G
Ga0265757_127696/205-308       ..........UGG.C.UCCA.A.G.GGAAGAAG..G
Ga0316029_101202/440-551       ..........GACCAAGCU.......CCCA.G.A.AGAAGAUA..G
Ga0307482_1134687/287-181      .....G..........AGGUG....UGGCC..........ACCU.G.G.AGAAGCAU..G
Ga0307484_104189/399-292       .....GAGGUGU.GG..........CC..........ACCU.G.G.AGAAGCAU..G
Ga0307921_1050271/251-412      AGC..........UAUACA.....G.A.GGAC.U.G.GGAAGAUG..G
Ga0184580_102899/442-582       ..........GCUCUUC.C.CUCA.C.G.GGAAGAGG..G
Ga0316029_136733/88-8          ..........G.G.AGAAACAU..G
Ga0180107_1174136/218-102      ..........UCAU...AACUGGU.......U.C.UCGU.A.G.AGAAGCUA..G
Ga0179948_1254202/198-296      ..........GUG.U.G.GGAAGACA..G
Ga0265746_1140777/214-319      ..........UCG.C.UCCA.A.G.GGAAGAAG..G
Ga0265756_108694/382-277       ..........UCG.C.UCCA.A.G.GGAAGAAG..G
Ga0265758_129717/201-94        ..........U....UGGCUA..........CCA.A.G.GGAAGAAG..G
Ga0213850_1394829/253-105      ..........G.UAGUGUUG.U.UUCCGA..........UAUCCU.........CUGA.A.G.AGAAGACC..G
Ga0184603_139562/141-280       ..........CCAA.G.G.GGAAGAUC..G
Ga0157560_1100546/299-385      ..........AGU.G.G.GGAAGUCA..G
Ga0129331_1494381/292-368      ..........G.GGAAGACG..G
Ga0265759_100473/818-988       ..........G.CAAUGUGU.U.UGUCCUUAGCCUCGGA....UCUCCAGC...U.U.GGCU.G.A.AGAAGAAGC.G
Ga0265746_1003835/1049-1219    ..........G.CAAUGUGU.U.UGUCCUUAGCCUCAGA....UCUCCAGC...U.U.GGCU.G.A.AGAAGAAGC.G
Ga0265756_100592/1458-1288     ..........G.CAAUGUGU.U.UGUCCUUAGCCUCAGA....UCUCCAGC...U.U.GGCU.G.A.AGAAGAAGC.G
Ga0247536_100773/637-807       ..........G.CAAUGUGU.U.UGUCCGUAGCCUCAGA....UCUCCAGC...U.U.GGCU.G.A.AGAAGAAGC.G
Ga0184578_101068/480-310       ..........G.CAAUGUGU.U.UGUCCUUAGCCUCGGA....UCUCCAGC...U.U.GGCU.G.A.AGAAGAAGC.G
Ga0247530_109035/489-319       ..........G.CAAUGUGU.U.UGUCCUUAGCCUCAGA....UCUCCAGC...U.U.GGCU.G.A.AGAAGAAGC.G
Ga0247513_128543/241-71        ..........G.CAAUGUGU.U.UGUCCUUAGCCUCAGA....UCUCCAGC...U.U.GGCU.G.A.AGAAGAAGC.G
Ga0184573_112336/323-153       ..........G.CAAUGUGU.U.UGUCCUUAGCCUCAGA....UCUCCAGC...U.U.GGCU.G.A.AGAAGAAGC.G
Ga0127502_10575752/515-449     ..........AGG.G.G.GGAAGCAG..G
Ga0180107_1033011/786-679      ..........GGAGAA........G.UAGU.G.G.GGAAGGCG..U
Ga0222728_1380294/134-238      ..........CGCG...UUGGCCA............CCC.A.G.GGAAGCAC..G
Ga0307484_107311/306-374       ..........GG.U.G.GGAAGCUU..G
Ga0316046_107255/463-562       ..........A........G.CGAG.G.G.AGAAGCGU..G
Ga0184570_114412/209-338       ..........AUCAUU..CUG.A.AAGA.A.G.GGAAGACC..G
Ga0242646_1000880/695-856      ..........CAUUC...CCCCACUA....CCG.A.AGUA.G.G.AGAAGAAU..U
Ga0316031_123456/415-346       ..........C.C.G.GGAAGACC..G
Ga0157630_1203478/203-99       ..........AC.C.G.GGAAGACAG.G
Ga0247541_102707/328-184       .....GAGGGGG.AAC..........GGCACCU...C.U.UCGA.A.G.GGAAGAGG..G
Ga0115594_1174394/642-729      ..........GGU.A.G.GGAAGACU..G
Ga0115594_1187657/870-960      ..........GGU.A.G.GGAAGACU..G
Ga0242646_1047905/92-182       ..........GCA........GGA.G.A.GGAAGACC..G
Ga0247541_101130/558-391       .....CGUAGGU.CUA.........UGUGUCUGAUCGAUCG.....ACUCCAAC...C.A.GGUU.G.A.AGAAGAAGC.G
Ga0307921_1062541/180-338      ..........UGGA....CUG.G.GGAC.U.G.GGAAGAUG..G
Ga0184572_105168/388-281       ..........GGGUAGAA........UGG.A.G.AGAAGAAC..G
Ga0316036_127178/124-231       ..........GGGUAGAA........UGG.A.G.AGAAGAAC..G
JGI12272J11983_1345183/364-258 ..........GGCUA....GG.A..UGU.G.G.CGAAGAAAU.G
Ga0247535_100068/719-828       ..........GGGUAGAA........UGG.A.G.AGAAGAAU..G
Ga0222728_1000079/1192-1299    ..........GGGUAGGA........UGG.A.G.AGAAGAAGA.G
Ga0081246_1201717/153-259      ..........CGGGUAGGA........UGU.G.G.AGAAGAAAU.G
Ga0222728_1216887/51-157       ..........CGGGUAGGA........UGG.A.G.AGAAGAAGC.U
Ga0157569_1073490/184-258      ..........A.A.UUCU.G.G.AGAAGUCCU.G
Ga0180047_1061805/548-622      ..........A.A.UUCU.G.G.AGAAGUCCU.G
Ga0206353_11976089/292-380     ..........UUG.C.G.GGAAGAAC..G
Ga0247553_101094/709-629       ..........CG.U.G.GGAAGUAU..G
Ga0242641_1004288/32-112       ..........CCG.U.G.GGAAGUAC..G
Ga0242641_1014474/336-257      ..........CG..............GAU.GCG.GGAAGUGG..G
Ga0126318_10199518/109-204     ..........GAUGGAGUA...U.G.GUGC.G.C.GGAAGCUC..G
Ga0307919_1109364/247-158      ..........G.AGAG.A.G.GGAAGACA..G
Ga0180107_1307618/257-174      ..........A.GGAG.C.G.GGAAGACU..G
Ga0179937_1126086/216-325      ..........CA.C.GCUA.C.G.GGAAGACC..G
Ga0265744_134092/134-214       ..........G.GUCU.G.G.AGAGGCAA..G
Ga0307482_1015399/786-719      ..........GGU.G.G.AGAGGCAA..G
Ga0265751_100477/874-675       ..........UGUG...C.C.UGCU.G.G.AGAAGCAAA.U
Ga0265756_100433/485-286       ..........UGUG...C.C.UGCU.G.G.AGAAGCAAA.U
Ga0265743_100639/33-191        ..........UGUG...C.C.UGCU.G.G.AGAAGCAAA.U
Ga0184602_130623/1321-1100     CUUGGCUGACACAAAUCUCAUGUGUAACC..........ACGUACA....AGGAUUAC..CCG.G.UACG.G.G.AGAAGAAC..U
Ga0184597_126105/585-362       CUCUUGGCUGACACAAAUCUCAUGUGUAACC..........ACGUACA....AGGAUUAC..CCG.G.UACG.G.G.AGAAGAAC..U
Ga0187793_1322799/72-151       ..........A.GCUC.A.G.GGAAGACU..G
Ga0316037_128286/253-347       ..........GC.U.CAGA.G.G.GGAAGACC..G
Ga0265758_109576/83-157        ..........G.UGGU.G.G.AGAAACCU..C
Ga0265751_100127/176-103       ..........UC.A.G.GGAAGUAU..G
Ga0265758_108618/113-22        ..........G.A.GCACUC.G.GGAAAUCC..G

                               .....>>>>>>>>.>>>>>>>>>.>.>>>>>>>>>>>>>>>>>>....>>>>>>....>>>.>.>>>>.>.>.....>>>>..<
                               ..........2222.2.2.....2222..2
                               ----------○○○●-●-G-RGAAG●●●--R
```

```
Ga0228708_1128185/228-344        ......................................C.............AUCCCAUU....G.A.GCGU.G.A.AGAAGUCA..G
Ga0256309_1199458/363-444        ......................................................................GAA.U.G.GGAGGUUG..A
Ga0256302_1090383/76-157         ......................................................................GAA.U.G.GGAAGUUG..A
Ga0255224_1021381/13-113         ......................................................................GAA.U.G.GGAAGUUG..A
Ga0255223_1087001/28-107         ......................................................................GAA.U.G.GGAAGUUG..A
Ga0127502_11371108/362-252       ..............................................GGGUAGGA........UGU.A.G.AGAAGAGAC.G
Ga0222728_1000125/1315-1238      .....................................................................GUAG.A.G.AGAGGCGA..G
Ga0242654_10000586/1273-1196     ..................................................U..........AUG.A.G.AGAGGCGA..G
Ga0180107_1218358/314-219        .....................................................................GUAU.G.G.GGAAGUAA..U
Ga0307482_1020425/440-614        .................................CGCAUGUUAGGG...U..AGCUAA...CCA.A.GCUA.G.G.AGAAGAUU..C
Ga0179951_1211246/478-633        .................................................................A.G.GUUA.U.G.GGAAGACG..G
Ga0179950_1197742/286-441        .................................................................A.G.GUUA.U.G.GGAAGACG..G
Ga0129331_1058147/189-99         ......................................................................GUG.G.G.GGAAGAAC..G
Ga0265756_102054/286-395         .............................................C.GAUAGGA........UCU.G.G.GGAAGAAC..A
Ga0222728_1000128/4501-4432      ...................................................................U.AGGU.G.G.UGAAGCAG..G
Ga0265759_114856/370-262         ......................................................................CUU.A.G.GGAAGACU..G
Ga0079039_1551950/237-315        .....................................................................GCGC.U.G.GGAAGACU..G
Ga0179943_1170285/153-222        ......................................................................GUU.U.G.GGAAGACU..G
Ga0213850_1119088/23-236         UGACG...............................................................CAU.G.UGUA.A.G.GGAAGACU..G
Ga0247533_103075/417-305         ......GGCG..............................AUA..CC..U.CUUC.G.G.GGAAGAGG..C
Ga0247541_102964/56-168          ......GGCG..............................AUA..C.C.U.CUUC.G.G.GGAAGAGG..C
Ga0265755_105988/310-386         .....................................................................CGAA.G.A.GGAAGACG..G
Ga0157560_1026167/317-382        .......................................................................CA.C.A.GGAAGACA..G
Ga0115595_1093158/7-137          ......GCUGGGU.CU..........................................G.CGUG.A.G.GGAAGCCG..G
Ga0256352_1101982/155-277        ..................................................................U.GAGA.C.G.GGAAGACA..G
Ga0256350_1100736/283-405        ..................................................................U.GAGA.C.G.GGAAGACA..G
Ga0213850_1360620/298-221        .......................................................................GA.C.G.GGAAGCUG..G
Ga0081247_1167831/113-35         ........................................................................G.A.G.GGAAGACU..G
Ga0127503_10676578/136-67        .......................................................................GG.G.G.GGAAGACG..G
Ga0138283_1149501/173-59         ......GGC...........................GAGUCA..........GGG.G.G.UGAAGUCG..G
Ga0265757_139522/177-70          ..............................................GGGUAGGA........UCU.G.G.AGAAGAUA..G
Ga0265750_1013321/389-496        ..............................................GGGUAGGA........UCU.G.G.AGAAGAUA..G
Ga0316029_116217/368-261         ..............................................GGGUAGGA........UGA.G.G.AGAAAACU..G
Ga0316047_100016/2681-2574       ..............................................GGGUAGGA........UGA.G.G.AGAAAACU..G
Ga0184569_112764/943-836         ..............................................GGGUAGGA........UGA.G.G.AGAAAACU..G
Ga0242652_1131915/134-203        ......................................................................GGU.G.G.GGAAGACU..G
Ga0307482_1020622/600-404        ......CCUCGUG.UGUAUUGCCU.AUCACACGCUG...AC.....AGUCUAAC...U..G.ACAG.G.G.AGAAGAUC..U
Ga0265756_143657/133-55          ..........................................................................C.G.GGAAAACA..G
Ga0180107_1262812/3136-3066      .......................................................................CC.G.G.GGAAGACU..G
Ga0265756_118952/425-318         ..............................................AUU.........CUU.G.G.GGAAGCUC..G
Ga0265757_113703/332-416         .......................................................................CG.C.G.GGAAGAAA..C
Ga0256332_1243244/48-162         .......................................................................AC.U.G.GGAAGCCG..G
Ga0307919_1048575/242-90         ......GGAACG....GCCUA.G.C..........AGAUU..UAUGUGGA....CUG.A.GGAC.U.G.GGAAGAUG..G
Ga0265754_1081367/214-290        .......................................................................GG.U.G.GGAAGACA..G
Ga0184597_114231/79-11           ........................................................................C.U.G.GGAAGCAG..C
Ga0184582_129853/106-35          ......................................................................CAC.U.G.GGAAGCAG..U
Ga0265744_136679/344-276         ......................................................................GGU.C.G.GGAAGCAG..C
Ga0326631_172366/284-207         ......................................................................ACA.U.G.GGAAGAAG..U
Ga0307921_1021783/160-261        .............................................AGCG.............CAC.G.G.GGAAGAUU..G
Ga0247554_113506/254-161         ................................................CCAU.......C.AUAU.G.G.GGAAGUUA..U
Ga0179939_1229388/322-392        ......................................................................CAG.C.G.GGAAGACU..G
Ga0184568_100511/100-195         ..................................................................C.UCAG.C.G.GGAAGACC..G
Ga0247525_114510/199-318         ..............................GGAAGGUCC.....UGUUGACU........GUUC.G.G.GGAAGACA..A
Ga0222728_1008526/24-283         UAAAAUAUGUGUUAUCCUUGGGAGACUUGGAUAGCAUGA.......................AC...AAG.G.GAUA.A.G.GGAAGACC..G
Ga0242654_10278372/461-362       .................................................UUU...........ACC.A.G.CGAAGCAUA.G
Ga0184570_100371/69-155          ......................................................................GGC.G.G.GGAAGACU..G
Ga0184597_120210/233-147         ...............................................UGCA............GGC.G.G.GGAAGACU..G
Ga0213850_1220553/385-463        ..................................................................G.UGGU.G.G.GGAAGUCA..G
Ga0265745_1120044/54-129         ......................................................................GGU.G.G.GGAAGAGC..G
Ga0213850_1278242/600-506        ......................................................................GCC.G.A.GGAAGACG..G
Ga0213850_1524759/286-380        ......................................................................GCC.G.A.GGAAGACG..G
Ga0242657_1009512/1576-1466      ..............................................GGAUAGGA........UGU.G.G.AGAAGAGUU.G
Ga0307482_1430195/259-189        .......................................................................GA.U.G.GGAAGCUC..G
Ga0184590_129043/450-518         ......................................................................GGA.U.G.GGAAGCAC..G
Ga0184578_132646/9-94            ......................................................................UUG.G.G.GGAAGACU..G
Ga0180107_1040049/1100-1010      ........................................................................C.C.G.GGAAGAAA..G
Ga0307484_103912/620-553         ........................................................................G.C.G.GGAAGCCU..G
Ga0307921_1037919/342-457        ................................................................U.G.UCCU.G.G.AGAAGAAA..G
Ga0307920_113994/654-539         ..................................................CAUAC...U.G.UCCU.G.G.AGAAGAAA..G
Ga0316040_172648/178-66          ..............................................GGCCAUAC...U.G.UCCU.G.G.AGAAGAUA..G
Ga0184577_110850/246-358         ...............................................GCCAUAC...U.G.UCCU.G.G.AGAAGAUA..G
Ga0307920_113994/130-9           ................................................................U.G.UCCU.G.G.AGAAGAAA..G
Ga0307919_1081825/191-74         ................................................................U.G.UCUU.G.G.AGAAGAAA..G
Ga0307921_1095896/178-61         ................................................................U.G.UCUU.G.G.AGAAGAAA..G
Ga0316031_113250/505-406         .......................................................................AG.C.G.GGAAGAGA..G
Ga0265744_118928/137-218         ......................................................................GUU.G.G.GGAAGCAC..G
Ga0265749_106268/131-212         ......................................................................GUU.G.G.GGAAGCAC..G
Ga0157562_1160399/232-315        .................................................................AA.U.CUAU.G.G.GGAAGACC..C
Ga0153880_1231581/116-241        ......GGUGGUC.UG..........................................GUC.G.G.GGAAGCUG..G
Ga0153880_1191383/455-340        ..............................................UACCAAU..GAA.G.AAUU.A.G.GGAAGAUCA.G
Ga0138282_1242808/325-475        ..............................GCUCAAGCAGAUG..........A....CGC.G.CUACAA.G.GGAAGAUC..G
Ga0242654_10024002/836-915       ......................................................................CUU.G.A.GGAAGAGU..G
Ga0242646_1103331/248-318        .....................................................................AGGA.A.G.GGAGGCUC..G
Ga0081243_1208438/380-456        .......................................................................GG.U.G.GGAAGACG..G
Ga0184600_147964/253-339         .................................................................GU.A.CGAU.GUG.GGAAGACG..G
Ga0316036_107710/408-322         .................................................................GU.A.CGAU.GUG.GGAAGACG..G
Ga0184601_135946/406-321         .................................................................GU.A.CGGU.G.G.GGAAGACA..G
Ga0184589_121067/502-587         .................................................................GU.A.CGAU.G.G.GGAAGACA..G
Ga0184598_138497/440-355         .................................................................GU.A.CGAU.G.G.GGAAGACA..G

                                 ......>>>>>>>>.>>>>>>>>.>.>>>>>>>>>>>>>>>>....>>>>>.....>>>.>.>>>>.>.>.....>>>>..<
                                 ..........................................................2222.2.2.....2222..2
                                 ------------------------------------------------------------○○○●-●-G-RGAAG●●●--R
```

```
Ga0184580_113725/262-335    .............................................................................................................................................G.G.GGAAGACA..G
Ga0184584_115572/267-354    ..................................................................................................................UC.....AUG.G.UAAG.GUG.GGAAGACC..U
Ga0247539_100086/1763-1850  ..................................................................................................................UC.....AUG.G.UAAG.GUG.GGAAGACU..U
Ga0184579_105257/1735-1648  ..................................................................................................................UC.....AUG.G.UAAG.GUG.GGAAGACU..U
Ga0126318_10229384/609-688  ..........................................................................................................................AA.G.UUGC.U.A.GGAAGCAG..G
Ga0307482_1000291/1027-950  .............................................................................................................................................A.G.GGAAGCUG..G
Ga0316031_119808/254-182    ...........................................................................................................................GACG.G.G.AGAAGAGU..A
Ga0307482_1023665/853-778   .............................................................................................................................A.CUGU.G.G.GGAAGACG..A
Ga0180106_1297567/444-515   ...........................................................................................................................G.U.UCUA.G.A.GGAAGCUG..G
Ga0307484_115660/213-145    ...........................................................................................................................UGUA.G.A.GGAAGCUC..G
Ga0307484_161007/136-39     .................................................................................................................UCCA...........CCA.U.G.GGAAGCUA..G
Ga0307484_177982/136-39     ...............................................................................................................UCCAC.............CA.U.G.GGAAGCUA..G
Ga0307482_1033688/516-422   ...............................................................................................................UCCA.............CCA.U.G.GGAAGCUA..G
Ga0265752_111922/418-343    .............................................................................................................................A.GACG.C.G.GGAAGAAA..G
Ga0179939_1199779/67-205    ...........................................................................................................................C.A.AUCG.G.G.GGAAGACC..A
Ga0307482_1023050/176-255   ..............................................................................................................................G.U.G.GGAAGUCG..A
Ga0307482_1202605/470-395   .............................................................................................................................C.AAGG.C.G.GGAAGACU..U
Ga0242657_1476020/160-244   ...........................................................................................................................GAUU.G.G.GGAAGAUA..G
Ga0247523_120120/325-403    .................................................................................................................CAC............AUG.U.A.GGAAGCUA..G
Ga0316035_179807/270-184    .........................................................................................................................UCA.C.GGAC.G.A.AGAAGCUG..G
Ga0210323_1050507/418-532   ............................................................................................................................CUG.A.G.GGAAGAAC..G
Ga0184571_112742/47-116     ...........................................................................................................................UGUA.G.G.AGAGGCAC..G
Ga0247538_100770/928-997    ...........................................................................................................................UGUA.G.G.AGAGGCAC..G
Ga0157560_1032840/250-177   ............................................................................................................................AUC.C.G.GGAAGCUU..G
Ga0247514_107497/1071-1140  .............................................................................................................................GA.G.G.AGAGGCUC..G
Ga0242646_1002014/11-270    AAAGAUAGGUAAAAUAUGUGUUAUCCUUGGGAGACUUGGAUAGCAUGA....................................................................AC...AAG.G.GAUA.A.G.GGAAGACC..G
Ga0222728_1008391/1281-1023 ....................................................................................................................AAC..AAA.G.GAUA.A.G.GGAAGACC..G
Ga0242646_1001395/255-509   GAAAGCGAGACCACGCCCAUAGGAAAUAUCCUUAG......................................................................................GGA.U.GGUG.G.G.GGAAGACA..G
Ga0307482_1000256/3341-3276 ..............................................................................................................................G.C.G.GGAAGCCA..G
Ga0242647_1006852/526-598   ............................................................................................................................CUG.G.GCGGAAGCUA..G
Ga0184580_111164/678-749    ...........................................................................................................................GAGG.G.U.GGAAGCUG..G
Ga0184600_102725/60-135     .............................................................................................................................U.GUUG.G.G.UGAAGCGA..G
Ga0184602_122974/190-265    ...........................................................................................................................GUUG.G.G.UGAAGCGA..G
Ga0256338_1343513/152-228   ...........................................................................................................................AGUG.A.G.GGAAGAGG..C
Ga0255268_1432700/127-51    ...........................................................................................................................AGUG.A.G.GGAAGAGG..C
Ga0157560_1147189/148-60    ...............................................................................................................UUG..........CC.G..UCU.U.G.GGAAGUAC..G
Ga0242654_10672177/244-165  .................................................................................................................GGC............GGU.G.A.GGAAGCAU..G
Ga0242654_10135580/213-336  ...............................................................................................................CGCAC...........UUGC.U.G.GGAAGCUG..G
Ga0265742_1000025/2819-2888 ............................................................................................................................GUG.U.G.CGAAGCUG..G
Ga0184599_154888/232-155    ................................................................................................................................A.G.GGAAGCAG..A
Ga0228707_1100139/344-194   .............................................................................................................GUGUCGU............GAU.G.A.GGAAGUGU..G
Ga0307919_1084008/308-378   ..............................................................................................................................C.G.G.GGAAGAAG..G
Ga0307919_1039507/513-440   ............................................................................................................................UUC.G.G.GGAAGAAG..A
Ga0307921_1024041/406-334   ............................................................................................................................UUC.G.G.GGAAGAAG..A
Ga0242657_1015112/947-881   ..............................................................................................................................G.G.G.GGAAACUC..G
Ga0307482_1021658/484-563   ...............................................................................................................GA..........A.G.UGUA.U.G.GGAAGCUU..G
Ga0307920_133961/233-140    ...........................................................................................................................GUCC.G.G.GGAAGAUU..G
Ga0307921_1019702/90-184    ..................................................................................................................A...........GUCC.G.G.GGAAGAUU..G
Ga0182092_1031527/199-109   ............................................................................................................................GUC.A.G.GGAAGACC..G
Ga0184575_104772/255-338    ............................................................................................................................GCG.A...AGAAGCUG..G
Ga0179940_1033563/296-410   .................................................................................................................G.......GUA.G.CUCG.A.G.AGAAAGCG..G
Ga0184596_102602/271-200    .............................................................................................................................G.AUCA.C.U.GGAAGCAA..G
Ga0184596_125844/4802-4873  .............................................................................................................................G.AUCA.C.U.GGAAGCAA..G
Ga0153880_1058095/344-241   ...............................................................................................................GGGCU............GAG.A.G.GGAAGAAC..G
Ga0153880_1167367/224-121   ...............................................................................................................GGGCU............GAG.A.G.GGAAGAAC..G
Ga0179943_1178349/195-298   ...............................................................................................................GGGUU............GAG.G.G.GGAAGAAC..G
Ga0179944_1313268/129-232   ...............................................................................................................GGGUU............GAG.G.G.GGAAGAAC..G
Ga0213850_1259428/445-342   ...............................................................................................................GGGCU............GAG.A.G.GGAAGAAC..G
Ga0115595_1051440/308-419   .............................................................................................................................G.UAAA.A.G.GGAAGUCA..G
Ga0310106_100998/1608-1690  .............................................................................................................................C.UUCU.G.G.GGAAGCAU..A
Ga0307482_1017422/1387-1206 .......................................................................UGUGCAU.CCUGCUCA.A.UCACAACUGAUUUACG...AGUAGCUUA...CCG.A.GCUA.G.G.AGAAGACU..C
Ga0307482_1050048/176-95    .........................................................................................................................GGG.G.GUCC.C.A.GGAAACAU..G
Ga0179939_1078463/91-178    .............................................................................................................................GG.U.G.GGAAGACG..U
Ga0247539_107237/153-229    .........................................................................................................................UGA.U.AUUA.GUG.GGAAGUAG..A
Ga0127502_10189886/163-55   .............................................................................................................................G.AAAA.C.G.GGAAGACA..G
Ga0265746_1085300/256-329   .............................................................................................................................G.GGAG.G.G.GGAAGGUG..G
Ga0242641_1002064/1203-1116 .................................................................................................................CGC.....UGG.G.AUUU.U.G.GGAAGCAC..G
Ga0242657_1090937/459-526   ............................................................................................................................CCG.G.G.AGAGGCAC..G
Ga0180107_1021361/468-373   ...........................................................................................................................GUGU.G.G.GGAAGAAC..A
Ga0184599_136526/36-116     ...........................................................................................................................CUCC.U.G.GGAAGCAU..U
Ga0316032_127868/210-130    ...........................................................................................................................CUCC.U.G.GGAAGCAU..U
Ga0032354_1062690/2659-2586 .........................................................................................................................GUG.C.AGGG.U.G.GGAAGCAU..G
Ga0242657_1027062/255-322   ............................................................................................................................GUG.G.G.GGAAGCAU..A
Ga0265747_105373/294-105    CUGAUCU............................................................................................CACU.CC.....UGCCUGCC....G.U.AGCA.G.G.AGAAGAUUC.A
Ga0265745_1000512/1043-854  CUGAUCU............................................................................................CACU.UC.....UGCCCGCC....G.U.AGCA.G.G.AGAAGAUUC.A
Ga0265745_1001084/890-701   CUGAUCU............................................................................................CACU.CC.....UCGCUGCC....G.U.AGCA.G.G.AGAAGAUUC.A
Ga0310117_104105/1058-869   CUGAUCU............................................................................................CACU.CC.....UGCCUGCC....G.U.AGCA.G.G.AGAAGAUUC.A
Ga0265745_1000697/596-785   CUGAUCU............................................................................................CACU.CC.....UGCCUGCC....G.U.AGCA.G.G.AGAAGAUUC.A
Ga0247551_106718/112-179    .............................................................................................................................AC.A.G.GGAAGCAC..G
Ga0184603_137775/245-174    ..............................................................................................................................C.C.G.GGAAGUAG..G
Ga0242641_1105635/265-351   .............................................................................................................................G.ACAC.U.G.GGAAGACG..G
Ga0242654_10795375/263-349  .............................................................................................................................G.ACAC.U.G.GGAAGACG..G
Ga0157576_1168696/131-64    ..............................................................................................................................G.U.G.GGAAGACG..G
Ga0255292_1094640/555-357   ............................................................................................................................CUG.A.G.GGAAGACG..G
Ga0256349_1073199/279-477   ...........................................................................................................................G.G..CUG.A.G.GGAAGACG..G
Ga0256358_1096062/218-22    ...........................................................................................................................G.G.GCUG.A.G.GGAAGACG..G
Ga0256350_1079106/235-433   ................................................................................................................U..........G.G..CUG.A.G.GGAAGACG..G
Ga0256352_1080168/251-449   ................................................................................................................U..........G.G..CUG.A.G.GGAAGACG..G
Ga0255293_1084149/567-369   ..........................................................................................................................UG.G..CUG.A.G.GGAAGACG..G

                            .......................................................................>>>>>>>.>>>>>>>>.>.>>>>>>>>>>>>>>>>.....>>>>>.....>>>.>.>>>>.>.>.....>>>>..<
                            ...............................................................................................................................2222.2.2.....2222..2
                            -------------------------------------------------------------------------------------------------------------------------------○○○●-●-G-RGAAG●●●--R
```

```
Ga0256348_1071395/353-551      ...GUGG...CUG.A.G.GGAAGACG..G
Ga0265758_100178/821-753       ...G.G.G.GGAAGCAC..G
Ga0157560_1090855/105-186      ...UGUC.A.G.GGAAGUCA..G
Ga0005851_1006483/274-172      ...AACAGCUU...UGUGAAU...GAG.A.G.GGAAGACC..G
Ga0184603_107262/477-596       ...GGAAGGU.CC...UGUUGACU...GUUC.G.G.GGAAGACA..A
Ga0184585_151549/495-376       ...GGAAGGU.CC...UGUUGACU...GUUC.G.G.GGAAGACA..A
Ga0242647_1022053/339-428      ...GUC.C.U.GGAAGCAAA.G
Ga0184599_128731/188-273       ...AG.G.UGGC.G.G.AGAAGACA..U
Ga0310114_149639/68-153        ...AG.G.UGGC.G.G.GGAAGACA..U
Ga0310119_131358/533-447       ...AG.G.UGCC.G.G.GGAAGACA..U
Ga0079074_1318576/412-571      ...C.CGAA.G.G.AGAAGACA..G
Ga0307484_102285/276-209       ...UC.C.U.AGAGGCAC..G
Ga0247534_109145/204-136       ...C.C.C.AGAGGCAC..G
Ga0242657_1013457/154-227      ...UUG.GUG.GGAAACUC..G
Ga0242654_10000487/4104-4168   ...GGG.G.A.GGAAGUCA..G
Ga0242647_1038796/266-195      ...CCUG.U.G.GGAAGCUC..G
Ga0115594_1156252/268-370      ...AUG...GUA.C.G.GGAAGACA..G
Ga0115594_1016490/326-410      ...GUG.C.G.GGAAGACA..G
Ga0115595_1007417/328-412      ...GUG.C.G.GGAAGACA..G
Ga0265742_1038020/174-90       ...UCGG.G.G.GGAAGUCG..G
Ga0184568_114501/165-82        ...G.A.G.GGAAGCCG..G
Ga0265755_113253/366-449       ...G.A.G.GGAAGCCG..G
Ga0184600_115185/364-447       ...G.GGAAGCCG..G
Ga0157593_1013623/299-215      ...G.G.GGAAGACG..G
Ga0153880_1374410/101-33       ...U.G.G.GGAAGAUU..G
Ga0316038_101969/827-896       ...AUG.C.G.GGAAGCUG..G
Ga0316046_130251/204-135       ...AUG.C.G.GGAAGCUG..G
Ga0265758_104499/242-367       ...GUCAAC..UCU.U.CCGC.G.A.GGAAGAGG..G
Ga0307484_108094/186-350       ...CGGCUU...CAUCCACC..CG.U..GUG.G.G.AGAAGUUG..G
Ga0307482_1009223/670-508      ...CGGCUU...CAUCCACC...C.G.UGUG.G.G.AGAAGUUG..G
Ga0242647_1067563/210-289      ...AUA.U.GUUG.U.G.GGAAGCAC..G
Ga0242657_1207391/462-383      ...AUA.U.GUUG.U.G.GGAAGCAC..G
Ga0265742_1001052/322-80       CUCUGGACCUCUCCUCGUGAUCGGAUGGCACACUUUCCUCAGUUUGGGUAAACCGAAUUGGGCGACGCCUCCGC...CUGCC...G.U.AGCA.G.G.AGAAGAUUC.U
Ga0265750_1004028/1262-1504    CUCUGGACCUCUCCUCGUGAUCGGAUGGCACACUUUCCUCAGUUUGGGUAAACCGAAUUGGGCGACGCCUCCGC...CUGCC...G.U.AGCA.G.G.AGAAGAUUC.U
Ga0310113_106868/972-730       CUCUGGACCUCUCCUCGUGAUCGGAUGGCACACUUUCCUCAGUUUGGGUAAACCGAAUUGGGCGACGCCUCCGC...CUGCC...G.U.AGCA.G.G.AGAAGAUUC.U
Ga0265747_100653/495-253       CUCUGGACCUCUCCUCGUGAUCGGAUGGCACACUUUCCUCAGUUUGGGUAAACCGAAUUGGGCGACGCCUACGC...CUGCC...G.U.AGCA.G.G.AGAAGAUUC.U
Ga0265752_100504/930-688       CUCUGGACCUCUCCUCGUGAUCGGAUGGCACACUUUCCUCAGUUUGGGUAAACCGAAUUGGGCGACGCCUCCGC...CUGCC...G.U.AGCA.G.G.AGAAGAUUC.U
Ga0265740_1001378/1084-842     CUCUGGACCUCUCCUCGUGAUCGGAUGGCACACUUUCCUCAGUUUGGGUAAACCGAAUUGGGCGACGCCUCCGC...CUGCC...G.U.AGCA.G.G.AGAAGAUUC.U
Ga0265750_1009219/272-30       CUCUGGACCUCUCCUCGUGAUCGGAUGGCACACUUUCCUCAGUUUGGGUAAACCGAAUUGGGCGUCGCCACCG...CCUGCC...G.U.AGCA.G.G.AGAAGAUUC.U
Ga0265757_104669/135-376       CUCUGGAUCUCUCCACUCGCCCGGAUGGCACACUUUCCUCAGUUUGGGUAAACCGAAUUGGGCGACGCCUCCGC...CUGCC...G.U.AGCA.G.G.AGAAGAUUC.U
Ga0265745_1000618/793-552      CUCUGGAUCUCUCCACUCGCCCGGAUGGCACACUUUCCUCAGUUUGGGUAAACCGAAUUGGGCGUCGCCACCG...CCUGCC...G.U.AGCA.G.G.AGAAGAUUC.U
Ga0265742_1088739/51-292       CUCUGGAUCUCUCCACUCGCCCGGAUGGCACACUUUCCUCAGUUUGGGUAAACCGAAUUGGGCGUCGCCACCG...CCUGCC...G.U.AGCA.G.G.AGAAGAUUC.U
Ga0265747_100509/1277-1036     CUCUGGAUCUCUCCACUCGCCCGGAUGGCACACUUUCCUCAGUUUGGGUAAACCGAAUUGGGCGUCGCCACCG...CCUGCC...G.U.AGCA.G.G.AGAAGAUUC.U
Ga0265752_100314/1466-1225     CUCUGGAUCUCUCCACUCGCCCGGAUGGCACACUUUCCUCAGUUUGGGUAAACCGAAUUGGGCGUCGCCACCG...CCUGCC...G.U.AGCA.G.G.AGAAGAUUC.U
Ga0265743_104537/484-553       ...GCC.U.A.GGAAGCUA..G
Ga0265744_160011/83-18         ...GG.G.G.UGAAACUA..G
Ga0153880_1410217/288-397      ...ACG..UCU.GAUGCG.A.G.GGAAGAUA..G
Ga0115594_1054328/389-276      ...GUC...G.UAAA.A.G.GGAAGUCA..G
Ga0316029_112968/271-337       ...G.A.G.GGAAGACA..G
Ga0157560_1034839/242-321      ...AG.A.G.GGAAGAUC..G
Ga0157566_1117351/242-321      ...AG.A.G.GGAAGAUC..G
Ga0157593_1112297/86-12        ...GAA.U.G.GGAAGUUC..G
Ga0157565_1020724/148-72       ...UGAA.U.G.GGAAGUUC..G
Ga0247519_104852/800-730       ...GGGU...C.U.G.GGAAGCAC..G
Ga0307480_1000549/913-1064     ...GUG..C.U.CGCA.G.G.AGAAGUAAC.G
Ga0316031_132099/178-294       ...G.GGUUGGCUCAUUGGUG..UUUU...G.G.UCAG.G.G.GGAAGAGU..C
Ga0247526_116548/279-354       ...AU...CCU.C.G.GGAAGCAU..G
Ga0247519_113845/334-409       ...GAU...CUU.C.G.GGAAGCAU..G
Ga0247529_111070/303-228       ...GAU...CUU.C.G.GGAAGCAU..G
Ga0184602_101759/1045-970      ...G.A.UCCU.C.G.GGAAGCAU..G
Ga0184600_139292/252-327       ...G.A.UCCU.C.G.GGAAGCAU..G
Ga0184580_108231/296-221       ...G.A.UCCU.C.G.GGAAGCAU..G
Ga0247534_105038/288-211       ...G.A.UCCU.C.G.GGAAGCAU..G
Ga0247552_109074/256-333       ...G.A.UCCU.C.G.GGAAGCAU..G
Ga0247542_100296/1022-1097     ...G.A.UCCU.C.G.GGAAGCAU..G
Ga0247545_113720/232-157       ...G.A.UCCU.C.G.GGAAGCAU..G
Ga0184597_110829/1090-1011     ...U.G.GGAAGCUG..G
Ga0184600_105920/81-160        ...CAGUC...U.G.GGAAGCUG..G
Ga0184578_129447/314-235       ...AA.C.G.GGAAGCUG..G
Ga0184602_121948/324-245       ...C...A.G.AA.C.G.GGAAGCUG..G
Ga0184603_125382/9-88          ...UAUACGA...UC.U.G.GGAAGCUG..G
Ga0247550_100138/208-129       ...UAUACAA...AC.U.G.GGAAGCUG..G
Ga0316046_122965/221-142       ...A.C.GAAC.U.G.GGAAGCUG..G
Ga0316051_1003245/138-56       ...A.C.GAAC.U.G.GGAAGCUG..G
Ga0316049_122425/209-288       ...AC.U.G.GGAAGCUG..G
Ga0184588_113246/331-252       ...U.G.GGAAGCUG..G
Ga0184582_111448/113-237       ...AGC.U.G.GGAAGCAA..G
Ga0184597_112736/543-466       ...AGC.U.G.GGAAGCAA..G
Ga0184577_100528/550-473       ...AGC.U.G.GGAAGCAA..G
Ga0127502_10397722/141-209     ...CC.C.C.AGAGGCAC..G
Ga0184593_119512/675-608       ...UCC.C.C.AGAGGCAA..G
Ga0307482_1597388/86-18        ...GAC.U.C.AGAGGCAA..G
Ga0307482_1001973/47-115       ...GAC.U.C.AGAGGCAA..G
Ga0184595_132469/902-822       ...C.C.G.GGAAGCAUA.G
Ga0079039_1443516/199-38       ...AGGACACUC..CC.C.GGAA.A.G.GGAAGACG..A
Ga0265756_100130/1310-1239     ...UG.A.UGUU.G.G.CGAAGCUA..G
Ga0265747_125875/320-395       ...A.CGCG.G.G.GGAAGAAC..G
Ga0180052_189627/240-346       ...GUAGGAGG...GGU.G.G.GGAAGACC..G
Ga0180044_1074338/112-33       ...AUU.A.G.GGAAGUCG..G

                               ...>>>>>>>.>>>>>>>>.>.>>>>>>>>>>>>>>>>...>>>>>...>>>.>.>>>>.>.>.....>>>>..<
                               ...2222.2.2.....2222..2
                               ---○○○●-●-G-RGAAG●●●--R
```

```
Ga0157559_1047848/128-41      ...AGG.G.G.GGAAGAAC..G
Ga0157562_1134247/69-160      ...AGG.G.G.GGAAGAAC..G
Ga0247548_101188/825-888      ...U.G.G.GGAAGAUC..G
Ga0228707_1093045/293-207     ...G.A.G.GGAAGACU..U
Ga0184597_119383/488-559      ...C.C.G.GGAAGCUA..A
Ga0184602_121040/35-106       ...UUAC.C.G.GGAAGCUA..A
Ga0326631_107633/416-489      ...UUAC.C.G.GGAAGCUA..A
Ga0316046_149266/91-162       ...UUAC.C.G.GGAAGCUA..A
Ga0247538_100917/537-466      ...UUAC.C.G.GGAAGCUA..G
Ga0247519_118271/334-405      ...UAC.C.G.GGAAGCUA..A
Ga0265747_100454/1395-1324    ...UGA.C.G.GGAAGCUA..A
Ga0316046_100502/370-442      ...UUGA.U.G.GGAAGCUA..A
Ga0247551_101484/617-543      ...G.A.UUGA.C.G.GGAAGCUA..A
Ga0213850_1280121/544-351     UUAAAAUUGCGGCUUUGG...A....UGU.U.UAGG.G.G.GGAAGACA..G
Ga0265759_100017/4211-4288    ...AU.G.G.AGAGGCGU..G
Ga0307482_1127544/505-398     ...UAAUG............CCG.C.G.GGAAGACC..G
Ga0180107_1057591/210-107     ...GGUGU.........A.UA.U.U.G.GGAAGCUA..G
Ga0157569_1061476/44-119      ...G.G.GGAAGACA..G
Ga0307484_100889/412-594      UUUACGAG...UAGCUUA...CCG.A.GCUA.G.G.AGAAGACU..C
Ga0310106_100022/563-489      ...G.G.GGAAGUAU..A
Ga0310115_100154/4483-4559    ...G.G.GGAAGUAU..A
Ga0180107_1262812/2765-2832   ...A.G.G.GGAAGUCU..A
Ga0184597_110025/865-793      ...GGA.U.G.GGAAGUAU..C
Ga0247550_100495/487-560      ...GGA.U.G.GGAAGUAU..C
Ga0184582_135749/62-135       ...GGA.U.G.GGAAGUAA..C
Ga0184603_122688/908-835      ...GGA.U.G.GGAAGUAA..C
Ga0247524_126530/125-198      ...GGA.U.G.GGAAGUAA..C
Ga0316030_115438/368-435      ...GA.G.G.AGAGGAUG..G
Ga0307354_155926/276-116      ...C.CAAA.G.G.AGAAGACA..G
Ga0079074_1279626/114-274     ...C.CAAA.G.G.AGAAGACA..G
Ga0184602_132007/314-246      ...GGA.G.G.AGAAGACA..G
Ga0307921_1005950/791-877     ...GAGA.G.G.GGAAGACC..G
Ga0242654_10000487/4544-4475  ...GGA.G.G.GGAAGACC..G
Ga0157569_1032488/120-205     ...AGC.U.G.GGAAGACU..G
Ga0157593_1125711/213-128     ...AGC.U.G.GGAAGACU..G
Ga0157565_1077069/156-67      ...AGGA.A...........GCU.U.G.GGAAGACU..G
Ga0180047_1128081/151-64      ...AGGA.A...........GCU.U.G.GGAAGACU..G
Ga0265756_135986/152-290      ...G.GUUC.G.G.GGAAGAAC..G
Ga0180045_119797/166-305      ...UUCACACU...AGCACUCUCC.......GUUU.U.G.GGAAGAAC..G
Ga0115595_1089740/408-494     ...UGU.U.A.GGAAGACC..C
Ga0179955_1048341/123-51      ...GC.C.G.GGAAGACC..G
Ga0138280_1140856/341-479     ...CGCUCA...AGCAAAUGAC..GCG.C.UACA.A.G.GGAAGACC..G
Ga0180106_1302226/203-273     ...UGC.A.G.GGAAGACC..G
Ga0310119_169621/253-177      ...GG.A.G.GGAAGUUC..G
Ga0138277_1023917/182-18      ...GGC..GCG.C.UACA.A.G.GGAAGACC..G
Ga0138282_1020546/737-573     ...GGC..GCG.C.UACA.A.G.GGAAGAUC..G
Ga0179939_1059820/107-216     ...G.G.CUGU.U.G.GGAAGACC..G
Ga0179950_1098211/345-278     ...UGU.U.G.GGAAGACC..G
Ga0115594_1068468/127-56      ...GUC.C.G.GGAAGACC..G
Ga0228708_1103570/188-285     ...GAU.G.CAUU.C.G.GGAAGACC..G
Ga0265751_160438/108-215      ...C...GCGGU............AGG.U.G.GGAAGACA..G
Ga0242647_1000066/2638-2719   ...GUAG.G.G.GGAAGUUU..G
Ga0242646_1001227/1243-1308   ...G.G.G.GGAAGAUG..G
Ga0182091_1104347/98-194      ...A.CUU.C..AGA.G.G.AGAAGACA..G
Ga0115594_1178636/111-46      ...G.G.GGAAGACU..G
Ga0213850_1508869/496-402     ...GCA.G..AGA.A.G.GGAAGACC..G
Ga0307482_1152465/114-38      ...A.G.GGAAGCUG..G
Ga0180107_1031754/139-57      ...G.U.GUUA.GUG.GGAAGAUG..G
Ga0316037_121702/176-240      ...G.G.GGAAGAUG..G
Ga0265759_107962/103-39       ...G.G.GGAAGAUG..G
Ga0242649_1051170/368-302     ...G.G.GGAAGAUG..G
Ga0307482_1064439/84-153      ...G.G.GGAGGAUG..G
Ga0115594_1006122/120-29      ...AUC.........GGU.A.G.GGAAGACA..G
Ga0115595_1180708/226-317     ...AUC.........GGU.A.G.GGAAGACA..G
Ga0265746_1064626/389-458     ...GGU.A.G.GGAAGACA..G
Ga0265759_108120/277-351      ...AGGU.G.A.GGAAGACC..G
Ga0307482_1000450/2015-2077   ...G.G.GGAAGAUC..G
Ga0247527_115808/280-440      ...A.U.GGAAUGGGUGAUCCCU....CAGCUA...CCG.A.GGUA.G.G.AGAAGAAU..C
Ga0184588_120624/266-426      ...U.GGAAUGGGUGAUCCCU....CAGCUA...CCG.A.AGUA.G.G.AGAAGAAU..C
Ga0184579_106194/996-1156     ...U.GGAAUGGGUGAUCCCU....CAGCUA...CCG.A.AGUA.G.G.AGAAGAAU..C
Ga0265758_100254/356-516      ...U.GGAAUGGGUGAUCCCU....CAGCUA...CCG.A.AGUA.G.G.AGAAGAAU..C
Ga0316035_107544/354-514      ...A.U.GGAAUGGGUGAUCCCU....CAGCUA...CCG.A.AGUA.G.G.AGAAGAAU..C
Ga0184596_109261/979-1139     ...A.U.GGAAUGGGUGAUCCCU....CAGCUA...CCG.A.GGUA.G.G.AGAAGAAU..C
Ga0184600_132066/568-408      ...A.U.GGAAUGGGUGAUUCCU....CAGCUA...CCG.A.AGUA.G.G.AGAAGAAU..C
Ga0184597_106861/196-36       ...A.U.GGAAUGGGUGAUUCCU....CAGCUA...CCG.A.AGUA.G.G.AGAAGAAU..C
Ga0316031_108320/216-376      ...A.U.GGAAUGGGUGAUUCCU....CAGCUA...CCG.A.AGUA.G.G.AGAAUAAU..C
Ga0184589_110648/1041-881     ...A.U.GGAAUGGGUGAUCCCU....CAGCUA...CCG.A.AGUA.G.G.AGAAGAAU..C
Ga0184580_124121/239-79       ...A.U.GGAAUGGGUGAUCCCU....CAGCUA...CCG.A.AGUA.G.G.AGAAGAAU..C
Ga0184598_101324/530-690      ...A.U.GGAAUGGGUGAUCCCU....CAGCUA...CCG.A.AGUA.G.G.AGAAGAAU..C
Ga0247547_102464/189-29       ...A.CGAGCAGGCACUUCCU....CAGCUA...CCG.G.AGUA.G.G.AGAAGAAU..U
Ga0247551_100828/546-387      ...A.CGAGCAGGCACUUCCU....CAGCUA...CCG.G.AGUA.G.G.AGAAGAAU..U
Ga0247536_101914/360-201      ...A.CGAGCAGGCACUUCCU....CAGCUA...CCG.G.AGUA.G.G.AGAAGAAU..U
Ga0247538_100480/788-947      ...A.CGAGCAGGCACUUCCU....CAGCUA...CCG.G.AGUA.G.G.AGAAGAAU..U
Ga0247550_100850/456-297      ...A.CGAGCAGGCACUUCCU....CAGCUA...CCG.G.AGUA.G.G.AGAAGAAU..U
Ga0247553_110698/526-367      ...A.CGAGCAGGCACUUCCU....CAGCUA...CCG.G.AGUA.G.G.AGAAGAAU..U
Ga0184589_112135/40-200       ...A.UCGCGGCACACUUCCU....CAGCUA...CCG.G.AGUA.G.G.AGAAGAAU..G
Ga0247526_110221/603-443      ...A.UGACGGAACACUUCCU....CAGCUA...CCG.G.AGUA.G.G.AGAAGAAU..U
Ga0247541_103784/123-283      ...A.UAGCGGACCACUUCCU....CAGCUA...CCG.G.AGUA.G.G.AGAAGAAU..U
Ga0247545_112960/183-343      ...A.UAGCGGACCACUUCCU....CAGCUA...CCG.G.AGUA.G.G.AGAAGAAU..U

                              ...>>>>>>>.>>>>>>>>.>.>>>>>>>>>>>>>>>>>.....>>>>>.....>>>.>.>>>>.>.>.....>>>>..<
                              ...........................................2222.2.2.....2222..2
                              -------------------------------------------○●●●-●-G-RGAAG●●●--R
```

```
Ga0184586__133850/368-528          ...........................................................................................A.UGACGGACCACUUCCU.....CAGCUA....CCG.G.AGUA.G.G.AGAAGAAU..U
Ga0247552__101742/612-452          ...........................................................................................A.UGACGGAACACUUCCU.....CAGCUA....CCG.G.AGUA.G.G.AGAAGAAU..U
Ga0247545__104341/406-246          ...........................................................................................A.UGACGGAACACUUCCU.....CAGCUA....CCG.G.AGUA.G.G.AGAAGAAU..U
Ga0184602__114416/11-171           ...........................................................................................A.UGACGGACCACUUCCU.....CAGCUA....CCG.G.AGUA.G.G.AGAAGAAU..U
Ga0316038__111670/11-171           ...........................................................................................A.UGACGGACCACUUCCU.....CAGCUA....CCG.G.AGUA.G.G.AGAAGAAU..U
Ga0247529__134913/224-64           ...........................................................................................A.UGGCGGACCACUUCCU.....CAGCUA....CCG.A.AGUA.G.G.AGAAGAAU..U
Ga0184603__143085/253-93           ...........................................................................................A.UGGCGGACCACUUCCU.....CAGCUA....CCG.G.AGUA.G.G.AGAAGAAU..U
Ga0316032__107575/215-55           ...........................................................................................A.UGGCGGACCACUUCCU.....CAGCUA....CCG.A.AGUA.G.G.AGAAGAAU..U
Ga0247519__110701/607-447          ...........................................................................................A.UGGCGGACCACUUCCU.....CAGCUA....CCG.G.AGUA.G.G.AGAAGAAU..U
Ga0247524__111427/50-210           ...........................................................................................A.UGGCGGACCACUUCCU.....CAGCUA....CCG.G.AGUA.G.G.AGAAGAAU..U
Ga0184574__121290/269-109          ...........................................................................................A.UAGCGGACCACUUCCU.....CAGCUA....CCG.G.AGUA.G.G.AGAAGAAU..U
Ga0184595__111222/3-163            ...........................................................................................A.UAGCGGACCACUUCCU.....CAGCUA....CCG.G.AGUA.G.G.AGAAGAAU..U
Ga0184600__123882/188-28           ...........................................................................................A.UGGCGGGCCACUUCCU.....CAGCUA....CCG.G.AGUA.G.G.AGAAGAAU..U
Ga0326631__101563/544-705          ...........................................................................................A.UCGCAGCACACUUCCU.....CAGCUA....CCG.G.AGUA.G.G.AGAAGAAU..U
Ga0184573__110190/115-276          ...........................................................................................A.UCGCAGAACACUUCCU.....CAGCUA....CCG.G.AGUA.G.G.AGAAGAAU..U
Ga0184596__111007/525-686          ...........................................................................................A.UCACAGAACACUUCCU.....CAGCUA....CCG.G.AGUA.G.G.AGAAGAAU..U
Ga0316033__144939/167-328          ...........................................................................................A.UCACAGAACACUUCCU.....CAGCUA....CCG.G.AGUA.G.G.AGAAGAAU..U
Ga0247526__117235/152-313          ...........................................................................................A.UCGCGGCACACUUCCU.....CAGCUA....CCG.G.AGUA.G.G.AGAAGAAU..U
Ga0247551__100195/490-329          ...........................................................................................A.UCGUGGCACACUUCCU.....CAGCUA....CCG.G.AGUA.G.G.AGAAGAAU..U
Ga0247538__100424/120-281          ...........................................................................................A.UCGCAGAACACUUCCU.....CAGCUA....CCG.G.AGUA.G.G.AGAAGAAU..U
Ga0247550__114736/167-7            ...........................................................................................A.UCGCAGAACACUUCCU.....CAGCUA....CCG.G.AGUA.G.G.AGAAGAAU..U
Ga0247529__103231/270-430          ...........................................................................................A.UCGCAGAACACUUCCU.....CAGCUA....CCG.G.AGUA.G.G.AGAAGAAU..U
Ga0184603__111918/241-82           ...........................................................................................A.UCGCAGAACACUUCCU.....CAGCUA....CCG.G.AGUA.G.G.AGAAGAAU..U
Ga0184603__126764/80-240           ...........................................................................................A.UCGCAGAACACUUCCU.....CAGCUA....CCG.G.AGUA.G.G.AGAAGAAU..U
Ga0247519__107918/217-58           ...........................................................................................A.UCGCAGAACACUUCCU.....CAGCUA....CCG.G.AGUA.G.G.AGAAGAAU..U
Ga0184580__114961/306-146          ...........................................................................................A.UCGCAGAACACUUCCU.....CAGCUA....CCG.G.AGUA.G.G.AGAAGAAU..U
Ga0213850__1042834/351-428         ..................................................................................................................................GGGC.G.G.GGAAGUCA..G
Ga0265756__103656/97-163           .....................................................................................................................................G.G.G.GGAAGUCA..G
Ga0265756__101189/896-833          .....................................................................................................................................G.G.G.GGAAGUCA..G
Ga0180107__1033011/414-478         .....................................................................................................................................G.G.G.GGAAGUCA..G
Ga0115594__1157594/155-254         ........................................................................................................................AUC........GUA.C.G.GGAAGACA..G
Ga0115594__1166236/119-19          ........................................................................................................................AUC........GUA.C.G.GGAAGACA..G
Ga0115594__1007070/120-30          .......................................................................................................................AAUG........GUA.C.G.GGAAGACA..G
Ga0115595__1181384/176-86          .......................................................................................................................AAUG........GUA.C.G.GGAAGACA..G
Ga0115594__1211214/299-389         .......................................................................................................................AUC.........GUG.C.G.GGAAGACG..G
Ga0115594__1182529/183-84          ................................................................................................................................U.CGUA.C.G.GGAAGACA..G
Ga0256352__1182139/180-81          ..................................................................................................................................CGUA.C.G.GGAAGACA..G
Ga0179944__1005311/261-382         ........................................................................................................CUGUG...UGAGGCC............GGG.A.G.GGAAGACC..G
Ga0213850__1508120/373-299         ...................................................................................................................................CUC.GAG.GGAAGACG..G
Ga0307484__162891/325-258          .....................................................................................................................................G.G.G.GGAAGACA..G
Ga0184596__111635/86-148           .....................................................................................................................................G.G.G.GGAAGACG..G
Ga0316051__1005111/447-509         .....................................................................................................................................G.G.G.GGAAGACG..G
Ga0265753__1379003/85-148          .....................................................................................................................................G.G.G.GGAAGACG..G
Ga0184603__116647/206-269          .....................................................................................................................................G.G.G.GGAAGACG..G
Ga0184587__132427/565-485          ..............................................................................................................................G.G.GAUU.G.G.GGAAGACU..G
Ga0184599__110049/275-349          ..............................................................................................................................G.G.GUUU.G.G.GGAAGACU..G
Ga0184603__148109/996-920          ..............................................................................................................................G.G.GUUU.G.G.GGAAGACU..G
Ga0310114__133004/56-132           ..............................................................................................................................G.G.GUUU.G.G.GGGAGACU..G
Ga0310119__113172/381-306          ..............................................................................................................................G.G.GUUU.G.G.GGAAGACU..G
Ga0265747__119090/8-84             ..................................................................................................................................AGCA.C.G.GGAAGACU..G
Ga0247551__122551/29-115           ..................................................................................................................CCCCCUA.........AUAU.U.G.GGAAGCAG..G
Ga0316048__107802/239-324          ..................................................................................................................CCCCUA..........AUAU.U.G.GGAAGCAG..G
Ga0242657__1208314/452-382         ..................................................................................................................................AUAC.U.G.GGAAGCAC..G
Ga0184596__129213/83-168           ....................................................................................................................................GA.U.G.GGAAGCAC..G
Ga0184603__146917/441-510          ..................................................................................................................................GUAA.U.G.GGAAGCAC..G
Ga0247550__102601/160-89           ....................................................................................................................................AA.U.G.GGAAGCAC..G
Ga0184602__110024/263-177          ....................................................................................................................................AA.U.G.GGAAGCAC..G
Ga0184597__116115/700-787          ....................................................................................................................................AA.U.G.GGAAGCAC..G
Ga0184582__128596/700-786          ....................................................................................................................................AA.U.G.GGAAGCAC..G
Ga0184597__102019/330-402          ....................................................................................................................................AA.U.G.GGAAGCAC..G
Ga0242649__1000320/700-631         .....................................................................................................................................G.A.GCGGAAGCAU..G
Ga0179943__1102267/215-90          ........................................................................................................CUGUG...UGAGGCU............CGA.C.G.GGAAGACC..G
Ga0179944__1191552/284-411         ........................................................................................................CUGUG...UGAGGCU............CGA.U.G.GGAAGACA..G
Ga0179943__1150468/216-89          ......................................................................................................AGCGAUG...UGAGGCU............UGA.A.G.GGAAGACC..G
Ga0179944__1204933/216-89          ......................................................................................................AGCGAUG...UGAGGCU............UGA.A.G.GGAAGACC..G
Ga0265758__104558/436-365          ...................................................................................................................................GGA.U.G.GGAAGCUA..G
Ga0180107__1040315/471-547         ..................................................................................................................................GAGU.C.A.GGAAGCAU..G
Ga0180106__1332058/4386-4449       .......................................................................................................................................G.G.GGAAGCAC..G
Ga0307484__104189/90-171           ..............................................................................................................................G.U.UCGG.A.G.GGAAGUAA..G
Ga0265752__100023/1212-1290        ....................................................................................................................................GA.U.G.GGAAGUUG..A
Ga0157593__1101573/285-478         ..........................................................................CUUAUGA.A...............................CGGCU.........U.GUGG.G.G.GGAAACUA..G
Ga0307482__1008742/1212-1288       ..............................................................................................................................G.A.UGUG.G.G.AGAGGCGA..G
Ga0247551__104770/284-362          ....................................................................................................................................GC.C.G.GGAAGAAG..G
Ga0184593__112903/290-212          ....................................................................................................................................GC.C.G.GGAAGAAG..G
Ga0307482__1128390/271-339         ...................................................................................................................................AGC.C.G.GGAAGCAA..G
Ga0184580__126177/800-732          ...................................................................................................................................CCC.C.G.GGAAGCAG..G
Ga0242646__1115622/191-109         ............................................................................................................................ACA.A.UAAC.U.G.GGAAGCUG..G
JGI12272J11983__1319142/230-159    ...................................................................................................................................CCC.C.U.GGAAGCAC..G
Ga0184596__105062/163-229          ...................................................................................................................................CCC.C.U.GGAAGCGC..G
Ga0307482__1011891/77-10           ...................................................................................................................................CCC.C.U.GGAAGCGC..G
Ga0184588__125064/416-488          .....................................................................................................................................C.C.G.GGAAGCAC..G
Ga0184577__107529/254-327          .....................................................................................................................................C.C.G.GGAAGCAC..G
Ga0180106__1182034/403-337         ....................................................................................................................................CC.C.C.GGAAGCAC..G
Ga0180107__1241189/1554-1620       ....................................................................................................................................CC.C.C.GGAAGCAC..G
Ga0222728__1000110/4754-4821       ...................................................................................................................................UCC.C.G.UGAAGCAU..A
Ga0307484__100154/562-629          ...................................................................................................................................UCC.C.G.UGAAGCAU..A
Ga0180107__1285439/94-155          .........................................................................................................................................G.GGAAGCAG..G
Ga0184580__116530/249-177          ...................................................................................................................................GUC.U.G.GGAAGCAU..G
Ga0247526__117664/434-499          ....................................................................................................................................CA.U.G.GGAAGCUG..G

                                   ..........................................................................>>>>>>>.>>>>>>>>.>.>>>>>>>>>>>>>>>>.....>>>>>.....>>>.>.>>>>.>.>.....>>>>..<
                                   ..................................................................................................................................2222.2.2.....2222..2
                                   ----------------------------------------------------------------------------------------------------------------------------------○○○●-●-G-RGAAG●●●--R
```

```
Ga0184580__120219/79-4          .............................................................................................................................A.G.ACGG.A.G.GGAAGCAU..G
Ga0247539__100482/682-612       ..................................................................................................................................GUG.U.U.GGAAGCUA..G
Ga0247516__124175/202-272       ..................................................................................................................................GUG.U.G.GGAAGCUA..G
Ga0184580__121247/966-1036      ..................................................................................................................................GUG.U.G.GGAAGCUA..G
Ga0265743__100411/1096-1162     ..................................................................................................................................GUA.U.G.GGAAGCUG..G
Ga0242657__1070636/108-176      ..................................................................................................................................GGA.G.G.UGAAGCUG..U
Ga0242657__1122750/95-162       .................................................................................................................................CGGA.G.G.UGAAGCUG..U
Ga0307482__1405960/253-322      ..................................................................................................................................GGG.U.G.AGAAGCUA..G
Ga0316049__154968/227-319       ...................................................................................................AUCCAACGU.......CGUA............UU.A.G.GGAAGUAA..G
Ga0127502__10852756/278-345     ...................................................................................................................................GA.U.G.GGAAGCGG..G
Ga0247534__100003/3657-3582     ...................................................................................................................................GA.U.G.GGAAGUCA..G
Ga0180106__1204848/284-221      ..................................................................................................................................UGA.U.G.GGAAGCAC..G
Ga0184585__128240/467-390       ...............................................................................................................................U.GUGG.AUG.GGAAGCAC..G
Ga0180106__1180558/1549-1486    ....................................................................................................................................G.U.G.GGAAGCAU..G
Ga0265755__100134/298-372       ..................................................................................................................................CAC.C.G.GGAAGUUC..G
Ga0247534__100772/309-234       ....................................................................................................................................A.C.G.GGAAGUUC..G
Ga0307482__1202063/402-466      ....................................................................................................................................G.G.G.GGAAGCUC..G
Ga0247514__114593/280-211       ...................................................................................................................................GA.G.G.AGAAGCCA..G
Ga0310101__157764/124-55        ...................................................................................................................................GA.G.G.AGAAGCCA..G
Ga0265745__1000136/129-60       ...................................................................................................................................GA.G.G.AGAAGCCA..G
Ga0265745__1013560/201-270      ...................................................................................................................................GA.G.G.AGAAGCCA..G
Ga0307482__1779657/200-270      ..................................................................................................................................AAA.G.G.GGAAGCAC..U
Ga0307482__1044183/658-591      ....................................................................................................................................A.G.G.GGAAGCAU..U
Ga0265758__100017/4376-4309     ....................................................................................................................................G.G.G.GGAAGCUA..G
Ga0242654__10617983/312-237     ...............................................................................................................................A.UCAU.U.G.GGAAGCAG..G
Ga0222728__1054736/119-193      .................................................................................................................................UGUA.GCG.GGAAGCUA..G
Ga0184583__106441/296-227       ..................................................................................................................................GUG.G.G.AGAGGCUA..G
Ga0247520__104849/891-821       ..................................................................................................................................GAU.G.A.GGAAGUAC..G
Ga0247518__137824/301-231       ..................................................................................................................................GAU.G.A.GGAAGCAC..G
Ga0247531__104861/1082-1012     ..................................................................................................................................GAU.G.A.GGAAGCAC..G
Ga0265746__1040401/190-123      ..................................................................................................................................UUU.G.G.GGAAGCAC..G
Ga0184603__139760/199-128       ..................................................................................................................................GAA.G.G.GGAAGCUU..G
Ga0184603__138085/381-311       ..................................................................................................................................GAG.G.G.GGAAGCUU..G
Ga0265747__102820/448-376       ...............................................................................................................................G.AGAA.G.G.GGAAGCUU..G
Ga0247554__105388/388-316       ...............................................................................................................................G.AGAG.G.G.GGAAGCUU..G
Ga0316037__123461/289-368       ...............................................................................................................................C.A.UG.C.G.GGAAGCUU..G
Ga0184597__121517/262-183       ...............................................................................................................................C.A.UG.C.G.GGAAGCUU..G
Ga0247551__100118/653-723       ..................................................................................................................................GAG.G.G.GGAAGCUU..G
Ga0265750__1365815/134-55       .................................................................................................................GACAU..............G.C.G.GGAAGCUU..G
Ga0184589__120335/11-90         .................................................................................................................GACAU..............G.C.G.GGAAGCUU..G
Ga0247521__124040/388-309       .................................................................................................................GACAU..............G.C.G.GGAAGCUU..G
Ga0247518__108423/758-837       .................................................................................................................GACAU..............G.C.G.GGAAGCUU..G
Ga0247531__104608/1256-1177     .................................................................................................................GACAU..............G.C.G.GGAAGCUU..G
Ga0247543__100863/642-721       .................................................................................................................GACAU..............G.C.G.GGAAGCUU..G
Ga0307482__1026216/884-813      ...............................................................................................................................G.GAUA.GUG.GGAAGCAU..G
Ga0307482__1636410/270-341      ...............................................................................................................................G.GAUA.GUG.GGAAGCAU..G
Ga0310119__128165/432-365       ...................................................................................................................................GG.G.U.GGAAGCAC..G
Ga0307482__1016877/168-97       ...................................................................................................................................UG.GUG.GGAAGCAU..G
Ga0126318__10746885/3334-3265   ..................................................................................................................................GGG.G.G.GGAAGCUA..G
Ga0206353__11117566/971-1032    ....................................................................................................................................G.G.G.GGAAGCAA..G
Ga0180107__1106188/424-495      ......................................................................................................................................U.G.GGAAGCAU..G
Ga0307482__1000354/2063-1987    ......................................................................................................................................U.G.GGAAGCAU..G
Ga0180107__1132096/355-287      ..................................................................................................................................GAG.G.G.GGAAGCAA..G
Ga0180108__1238667/303-371      ..................................................................................................................................GAG.G.G.GGAAGCAA..G
Ga0265751__100981/715-788       .................................................................................................................................GUUG.G.G.GGAAGCAG..G
Ga0265756__100898/263-336       .................................................................................................................................GUUG.G.G.GGAAGCAG..G
Ga0307484__102094/153-80        ..................................................................................................................................AGU.G.G.GGAAGCGU..G
Ga0307482__1023270/273-197      .................................................................................................................................GGGU.U.G.GGAAGCAU..G
Ga0184596__102741/625-701       ............................................................................................................................GA.C.AUAG.GCG.GGAAGUAU..G
Ga0316033__103637/453-391       ......................................................................................................................................G.G.GGAAGCAU..G
Ga0180107__1069127/170-106      ...................................................................................................................................UA.U.G.GGAAGUAG..G
Ga0247535__105396/277-348       .................................................................................................................................CCUA.U.G.GGAAGCAG..G
Ga0184576__110410/277-348       ...............................................................................................................................A.CCCA.U.G.GGAAGCAG..G
Ga0307482__1219330/19-95        ............................................................................................................................UG.G.UGUG.GCG.GGAAGCAA..G
Ga0247534__116177/42-105        ....................................................................................................................................G.A.G.GGAAGUAU..G
Ga0316033__123149/165-228       ....................................................................................................................................G.A.G.GGAAGUAU..G
Ga0265751__100560/642-572       ...................................................................................................................................GG.G.G.GGAAGUAC..G
Ga0307482__1011142/698-628      ....................................................................................................................................U.G.G.GGAAGCAC..G
Ga0307482__1465843/259-190      ....................................................................................................................................U.G.G.GGAAGCAC..G
Ga0184593__115334/333-240       .................................................................................................................GAUGUCGU.........AUU.G.G.GGAAGUAA..G
Ga0265751__100089/3005-3069     ......................................................................................................................................G.G.GGAAGUAC..G
Ga0307482__1000325/380-315      ....................................................................................................................................U.G.G.GGAAGUAC..G
Ga0222729__1014021/463-392      ....................................................................................................................................G.G.G.GGAAGCCU..A
Ga0307482__1033651/424-494      .................................................................................................................................UAUA.U.G.GGAAGCAU..G
Ga0307482__1015330/1021-1093    ..................................................................................................................................AUC.C.G.GGAAGCUA..G
Ga0184570__111551/35-97         ....................................................................................................................................C.U.G.GGAAGCUA..G
Ga0184576__117861/362-299       ....................................................................................................................................C.U.G.GGAAGCUA..G
Ga0307482__1589279/351-280      ..................................................................................................................................GCU.U.G.GGAAGCAA..G
Ga0184586__104419/174-104       ..................................................................................................................................ACC.U.G.GGAAGCAA..G
Ga0222728__1148620/185-250      ...................................................................................................................................UC.U.G.GGAAGCAA..G
Ga0316049__103217/889-823       ..................................................................................................................................AUC.U.G.GGAAGCAA..G
Ga0316034__104024/504-439       ..................................................................................................................................AUC.U.G.GGAAGCAA..G
Ga0184595__117496/296-230       ...................................................................................................................................UC.U.G.GGAAGCAA..G
Ga0184597__109849/897-963       ...................................................................................................................................UC.C.G.GGAAGCAA..G
Ga0184588__134146/1219-1285     ...................................................................................................................................UC.C.G.GGAAGCAA..G
Ga0184584__115247/186-121       ...................................................................................................................................UC.U.G.GGAAGCAA..G
Ga0184596__126546/83-162        .............................................................................................................................U.A.CAUC.U.G.GGAAGCUA..G
Ga0184570__116436/278-198       ...........................................................................................................................UAU.A.CAUC.U.G.GGAAGCUA..G
Ga0184597__111165/262-182       ...........................................................................................................................UAU.A.CAUC.U.G.GGAAGCUA..G

                                .........................................................................>>>>>>>.>>>>>>>>.>.>>>>>>>>>>>>>>>>.....>>>>>.....>>>.>.>>>>.>.>.....>>>>..<
                                .................................................................................................................................2222.2.2.....2222..2
                                ---------------------------------------------------------------------------------------------------------------------------------○●●●-●-G-RGAAG●●●--R
```

```
Ga0316037_169124/279-199     ............................................................................................................................UAU.A.CAUC.U.G.GGAAGCUA..G
Ga0247551_103383/322-242     ............................................................................................................................UAU.A.CAUC.U.G.AGAAGCUA..G
Ga0247543_103170/244-164     ............................................................................................................................UAU.A.CAUC.U.G.GGAAGCUA..G
Ga0184589_120451/249-329     ............................................................................................................................UAU.A.CAUC.U.G.GGAAGCUA..G
Ga0184600_118565/177-97      .............................................................................................................................AC.A.CGUC.U.G.GGAAGCUA..G
Ga0316034_100493/254-174     .............................................................................................................................AC.A.CGUC.U.G.GGAAGCUA..G
Ga0184576_117703/380-460     ............................................................................................................................UAC.A.CGUC.U.G.GGAAGCUA..G
Ga0184597_129399/366-446     ............................................................................................................................UAC.A.CGUC.U.G.GGAAGCUA..G
Ga0184590_111002/143-223     ............................................................................................................................UAU.A.CAUC.U.G.GGAAGCUA..G
Ga0184587_105985/71-151      ................................................................................................................................A.CACC.U.G.GGAAGCUA..G
Ga0184602_122864/595-515     ................................................................................................................................A.CAUC.U.G.GGAAGCUA..G
Ga0184593_101086/682-762     ................................................................................................................................A.CAUC.U.G.GGAAGCUA..G
Ga0247519_108375/286-206     .................................................................................................................CUAUAC............AUC.U.G.GGAAGCUA..G
Ga0127502_10351452/615-550   ....................................................................................................................................GG.G.G.CGAAGCUC..G
Ga0316038_112263/442-356     ..................................................................................................................GGCCC............GGG.G.G.GGAAGCAA..G
Ga0242657_1073895/573-672    ..................................................................................................................................GAGC.G.G.GGAAGCAC..U
Ga0184587_107783/167-95      ..................................................................................................................................CACC.A.G.AGAAGCAU..G
Ga0265758_101918/310-243     ....................................................................................................................................GA.A.G.GGAAGCAU..G
Ga0307482_1029599/655-579    ..................................................................................................................................CCUC.C.A.GGAAGCAG..G
Ga0265746_1009702/779-848    ...................................................................................................................................CUC.C.A.GGAAGCAG..G
Ga0184583_113025/102-26      .....................................................................................................................................C.U.G.GGAAGCUA..G
Ga0184580_109961/270-194     ....................................................................................................................................AC.U.G.GGAAGCUA..G
Ga0316049_111814/256-181     ....................................................................................................................................AC.U.G.GGAAGCUA..G
Ga0247553_120631/200-275     ....................................................................................................................................AC.U.G.GGAAGCUA..G
Ga0184593_119849/313-238     ....................................................................................................................UACAG...........AC.U.G.GGAAGCUA..G
Ga0184599_140898/774-849     ....................................................................................................................UACAG...........AC.U.G.GGAAGCUA..G
Ga0242647_1005188/439-360    .....................................................................................................................................C.C.G.AGAAGCAC..G
Ga0242657_1054283/541-620    .....................................................................................................................................C.C.G.AGAAGCAC..G
Ga0184597_102100/600-669     .....................................................................................................................................C.U.G.GGAAGCAC..G
Ga0213850_1460186/227-149    ..................................................................................................................................GGGU.U.G.GGAAGUCG..G
Ga0242654_10040657/1018-942  .............................................................................................................................UU.G.CGUA.G.G.AGAGGCGU..G
Ga0307484_100842/1194-1271   ..............................................................................................................................U.G.UGUA.G.G.AGAGGCGA..G
Ga0242654_10401151/84-171    ..............................................................................................................................G.G.AGGA.A.G.GGAAGAUG..G
Ga0310119_105137/743-673     ..................................................................................................................................UUAG.A.G.GGAAGCAG..G
Ga0247541_104364/333-188     ...........................................................................GAUAAUG.AG.......................................GUC.A.ACUA.A.G.UGAAGAUC..G
Ga0184582_129125/749-675     ................................................................................................................................G.AGUC.A.G.AGAAGCAC..G
Ga0265753_1022489/457-531    ................................................................................................................................G.AGUC.A.G.AGAAGCAC..G
Ga0265751_121301/189-266     ..............................................................................................................................G.A.ACAU.C.G.AGAGGACG..G
Ga0247526_105246/689-508     ...........................................................................GGUUGUU.AGA.CCUG.A.GUGCU..UUGCAC.UU.....GAUCCACC..CG.G.AUCA.G.A.AGAAGAUCU.U
Ga0247515_107373/548-731     ...........................................................................GGUGUUU.GAUACCUG.A.GUGCUUCUUGCAC.UU.....GAUCCACC..CG.G.AUCA.G.A.AGAAGAUCU.U
Ga0247538_100442/1363-1181   ...........................................................................GGUGUUU.GAUACCUG.A.GUGCU.UCUGCAC.UU.....GAUCCACC..CG.G.AUCA.G.A.AGAAGAUCU.U
Ga0247538_100551/721-542     ...........................................................................GGCUGUU.ACC.CCUG.A.GUGCU..UUGCAC.UU.....GAUCCACC..CG.G.AUCA.G.A.AGAAGACU..U
                             ...........................................................................>>>>>>>.>>>>>>>>.>.>>>>>>>>>>>>>>>>.....>>>>>....>>>.>.>>>>.>.>.....>>>>..<
                             ..................................................................................................................................2222.2.2.....2222..2
                             ----------------------------------------------------------------------------------------------------------------------------------○●●●-●-G-RGAAG●●●--R
```

alignment positions 451 · · · 600

```
Ga0316035_118720/195-42      .UGAGGGAAACAUU.U.A..UGU..UCG...............AGUUC.CUGAA................................................................................................
Ga0265747_125480/96-202      .UAAGGGAAACAUU.U.U..AUA..AGU...............UGUUG.U....................................................................................................
Ga0242657_1353977/135-311    .GAGGUGAAACAUA.U.A..GUA..U..................CUCU.UUAA.................................................................................................
Ga0242649_1051170/34-143     .GAGGAGAAACAUU.U.A..UAG..A..................GUAA.CG...................................................................................................
Ga0265758_100138/1527-1633   .CCAGGGAAACAUA.U.G..CAG....................GUG...GCA..................................................................................................
Ga0184593_103988/348-257     .AACAAGAAACACG.C.G..ACA....................ACAUA......................................................................................................
Ga0316047_106478/13-108      .UAGGGGAAACCUU.G.G..GCA..U.................AGAC...UA..................................................................................................
Ga0213850_1056422/14-139     .GACAAGAAACACC.G.C..GU.........CGCU..GGUCAUCCAGC..GAGA................................................................................................
Ga0316032_128094/136-241     .AUGGGGAAACACA.C.A..G......................UCUUG.ACACA................................................................................................
Ga0307482_1000311/1469-1363  .AGGGCAAAACACA.U.G..UG.....................ACUAA.C.GAC................................................................................................
Ga0242657_1051800/416-321    .AUGGGGAAACAUA.C.A..GAA..GU................AGCG.......................................................................................................
Ga0307921_1050750/452-358    .UGGAGGAAACAUU.A.G..UA................GUUGCGCU........................................................................................................
Ga0265756_117960/316-418     .GGAGUGAAACACG.G.C..AAC....................GUCGC......................................................................................................
Ga0242654_10352202/387-307   .GUAGUGAAACAUU.C.U..GUU..CC................AAGA.......................................................................................................
Ga0242652_1159268/284-150    .UUGGUGAAACAUG.G.A..UGG..C.................CCUUA.GCCGA................................................................................................
Ga0265744_102237/225-365     .UCGGUGAAACACG.A.C..CGC.....................CCGC.CGAA.................................................................................................
Ga0316029_101396/632-732     .GCGGUGAAACACU.U.A..GG.....................ACUA..GCGAC................................................................................................
Ga0265759_100202/139-258     .CAGAGGAAACACA.C.C..AG.....................AGCAG.CGG..................................................................................................
Ga0179943_1118543/65-150     .UUGGUGAAACAUA.C.A..AAG..UAG...............UUUA.......................................................................................................
Ga0182091_1018330/100-226    .CUGGUGAAACACU.U.C..GCG..UCUGUGGACA..C.....AAUUA.A....................................................................................................
Ga0127503_10119710/546-465   .AGUGUGAAACACA.C.G..CUA..G.................UUGGC.G....................................................................................................
Ga0138277_1022592/399-305    .UGGGCAAAACAUG.U.C..AAU..UAUUCA............UUAA.......................................................................................................
Ga0138278_1026470/183-287    .UGGGCAAAACAUA.U.C..AAU..UGUGUCUCGC..A.....AUCAU......................................................................................................
Ga0138277_1089473/408-304    .UGGGCAAAACAUA.U.C..AAU..UGUGUCUCGC..A.....AUCAU......................................................................................................
Ga0247538_105452/166-82      .ACGAGGAAACACG.U.G..UG.....................GUGCA...ACA................................................................................................
Ga0247554_102783/498-580     .ACGAAGAAACACG.U.G..UG.....................GUGCA.A....................................................................................................
Ga0138291_1177333/295-213    .ACAGGGAAACAUU.U.G..UAG..UGC...............UUAGU......................................................................................................
Ga0210324_1290306/191-291    AUCAGGGAAACACU.A.U..GUA..CA................GUAA.......................................................................................................
Ga0242657_1026087/460-322    .CUGGUGAAACAUG.G.C..ACG..GU................CGCUA......................................................................................................
Ga0307482_1147486/107-4      .ACGGUGAAACACG.U.A..AUC....................GUAGC......................................................................................................
Ga0184573_113483/125-229     .AUGGUGAAACACG.C.U..AAU....................AAUAA......................................................................................................
Ga0184576_116834/47-151      .AUGGUGAAACACG.C.U..AAU....................AAUAA......................................................................................................
Ga0184568_108419/492-395     .ACGGUGAAACACU.U.A..GAA..GU................AACG.......................................................................................................
Ga0307482_1023050/594-496    .GUGGUGAAACAUC.A.U..AGA..GU.................CGC.......................................................................................................
Ga0179955_1013331/439-311    .GACAAGAAACACC.G.C..GUA..GUUUUUG...........AAACUAUAAUAGUAUGACUUUUU....................................................................................
Ga0184596_112078/314-191     .CCAAGGAAACACA.C.C..ACU..UUGAUCAUCG..UACUAUAGCU.......................................................................................................
Ga0184602_108471/49-173      .CCAAGGAAACACA.C.C..ACU..UUGAUCAUUG..UGCUAUAGCUA......................................................................................................
Ga0247528_108647/438-562     .CCAAGGAAACACA.C.C..ACU..UUGAUCAUCG..UGCUAUAGCUA......................................................................................................

                             .<<<<.......<<.<.<..<<<..<<<<<<<<<<..<<<<<<...........................................................................................................
                             .2222.......22.2.2..22................................................................................................................................
                             -●●RGGGAAACAY●-●-●--●○○--------------------●●●●○------------------------------------------------------------------------------------------------------
```

```
Ga0316039__120091/354-252      .UGAGGGAAACACU.U.A..CGG..UAG................UCU.CGG...................................................................................................
Ga0307484__166952/62-163       .GGAGAGAAACACG.C.U..GAU..CU................AGCG.......................................................................................................
Ga0180107__1244437/408-507     .AGGGUGAAACAUG.A.U..GAA.....................UUAA.C.G..................................................................................................
Ga0265742__1065119/361-273     .CUGCAGAAACAUG.C.C..CGA..A.................UCUA.......................................................................................................
Ga0316035__113756/173-88       .GUGGUGAAACACG.U.C..GGA..CC................UCCCC.G....................................................................................................
Ga0206355__1134705/241-332     .UGAGGGAAACACG.C.C..AAC..AAUG..............AUCG.......................................................................................................
Ga0180107__1031754/4443-4551   .UGGGGGAAACAUU.A.U..ACG..A..................GUAA.C....................................................................................................
Ga0138292__1031932/14-100      .AGCGGGAAACACG.U.A..UCG..A.................ACUCU.AGGCU................................................................................................
Ga0316029__100807/1303-1197    .CCAAGGAAACAUA.U.A..GGC..GU................AGCG.......................................................................................................
Ga0184568__114257/261-413      .AUGGAGAAACAUG.C.C..AGC.....................CUGC.CGAA.................................................................................................
Ga0242647__1005188/73-152      .UCGGUGAAACACG.A.G..UAU..U.................CGCAA.GCAAU................................................................................................
Ga0153880__1340646/556-457     .UGAGGGAAACAUG.C.C..AC.....................AUCUA......................................................................................................
Ga0307484__101303/704-866      .CUGGUGAAACACC.G.U..GC.......UGGAGC..U.....GUUAC......................................................................................................
Ga0242641__1025149/251-89      .CUGGUGAAACACC.G.U..GCU.......GGAGC..U.....GUUAC......................................................................................................
Ga0242652__1002960/413-574     .CUGGUGAAACACC.G.U..GCU.......GGAGC..U.....GUUAC......................................................................................................
Ga0265754__1007706/448-552     .UCGGUGAAACAUU.A.U..AAC....................UUAAC......................................................................................................
Ga0157562__1164332/672-560     .UCGGUGAAACACC.G.C..CC.....................UAGG.......................................................................................................
Ga0157569__1058012/474-586     .UCGGUGAAACACC.G.C..CC.....................UAGG.......................................................................................................
Ga0307921__1027128/160-73      .UGGGUGAAACACU.C.U..UGC..UGU...............GAAGG......................................................................................................
Ga0138279__1083071/419-326     .UCGGUGAAACACC.C...........................AUCA.......................................................................................................
Ga0127502__10704012/113-7      .CCAGGGAAACAUG.C.A..GA.....................AGUGA.CGA..................................................................................................
Ga0222728__1000095/4541-4435   .CCAGGGAAACAUG.C.A..GGG....................AUGAC.A....................................................................................................
Ga0265750__1166784/261-415     .UCGGUGAAACAUG.C.A..CUG..C.................CUUUG.CCUGC................................................................................................
Ga0310115__109341/244-400      .UCGGUGAAACAUG.C.A..CUG..C.................CUUUG.CCUGC................................................................................................
Ga0265756__158829/72-188       .CCAGUAAAACACG.U.U..UUA..AUUCAU............ACUCAC.....................................................................................................
Ga0184583__104740/342-477      .UCGGUGAAACAUG.C.U..CGC.....................CCGC.CGAA.................................................................................................
Ga0180107__1215312/308-184     .UAGGGGAAACACA.C.C..AGU..CU................GCCG.......................................................................................................
Ga0265742__1013613/579-453     .ACGAAGAAACAUG.C.G..CGG..GA................UGCC.......................................................................................................
Ga0265748__110851/279-405      .ACGAAGAAACAUG.C.G..CGG..GA................UGCC.......................................................................................................
Ga0265752__120463/277-403      .ACGAAGAAACAUG.C.G..CGG..GA................UGCC.......................................................................................................
Ga0265759__115288/4-102        .CCGGGGAAACACA.U.G..GGA.....................UUAA.C.G..................................................................................................
Ga0242646__1001122/826-995     .CCGGGGAAACAUG.C.C..CAC....................UGAGG......................................................................................................
Ga0157593__1101573/244-147     .GUGGUGAAACAUC.U.C..AGU..GGU...............GCAUGGCGGUCGAGC............................................................................................
Ga0184597__102941/191-111      .UCGGUGAAACCUA.G.C.........................AACCU.UAAAC................................................................................................
Ga0184597__115944/155-80       .GUAGGGAAACAUA.G.C..U......................GAUCA......................................................................................................
Ga0265742__1051722/222-295     .GAAGGGAAACACG.G.A..U......................GAUCA......................................................................................................
Ga0180107__1229893/426-318     .AGAGGGAAACAUC.G.A..AG.....................CCUAA..CGAA................................................................................................
Ga0242654__11312609/202-83     .GCGGUGAAACACA.U.U..GCA..GAGCACUCAA....CAAUCAUUU.C....................................................................................................
Ga0187793__1464320/616-535     .GGAAAGAAACAUA.C.U..ACC..GAG...............GAGCA......................................................................................................
Ga0187794__1206392/88-6        .GGAGAGAAACAUA.C.U..ACC..GAG...............GAGCA......................................................................................................
Ga0316034__129624/151-284      .UCGGUGAAACACA.U.U..CGA....................CCAGCCGA...................................................................................................
Ga0184576__119948/531-664      .UCGGUGAAACACA.U.U..CGA....................CCAGCCGA...................................................................................................
Ga0184568__109070/428-286      .CAGGGGAAACACA.C.C..GUC..C.................AGCCG.A....................................................................................................
Ga0265756__100717/910-1050     .CAGGGGAAACAUA.C.U..GUC..CG.................CCCG......................................................................................................
Ga0179955__1195671/481-563     .GUAACGAAACACU.C.A..GAC..UU................UAUUU.C....................................................................................................
Ga0184579__120620/502-584      .CCGGUGAAACACU.U.G..UC.....................GACUG.A....................................................................................................
Ga0127502__10711632/191-294    .AAGGGGAAACAUC.U.G..UAA..U.................UAACG......................................................................................................
Ga0310119__125223/193-319      .UGAGAGAAACACG.A.A..AU...CA...UCCGA..UGAUGGCUAG.......................................................................................................
Ga0307482__1064439/425-316     .AUGGGGAAACACU.U.U..GGG....................CGUAA.C.GA.................................................................................................
Ga0307484__102002/698-552      .CCGGUGAAACAUG.C.U..CGC....................CAUCG.CCGAA................................................................................................
Ga0265741__107029/232-320      .UUGGUGAAACACU.U.U..UGU..GUUCUCGUCC..U.....UGA......A.................................................................................................
Ga0247515__120448/91-178       .UUGGUGAAACACU.U.U..UGU..GUUGGUAUCC........UUUAA......................................................................................................
Ga0265759__103410/556-638      .UUGGUGAAACACU.U.U..UGU..GUUGGUAUCC..U.....UUA........................................................................................................
Ga0247534__101509/435-523      .UUGGUGAAACACU.U.U..UGU..GUUGGUAUCC..U.....UUA........................................................................................................
Ga0310102__119240/132-46       .UUGGUGAAACACU.U.U..UGU..GUUGGUAUCC..U.....UUA........................................................................................................
Ga0265742__1084621/112-29      .UUGGUGAAACACU.U.U..UGU..GUUGACGUCC..U.....UGA........................................................................................................
Ga0310114__135156/99-185       .UUGGUGAAACACU.U.U..UGU..GUUGACGUCC..U.....UGA........................................................................................................
Ga0265752__102595/694-607      .UUGGUGAAACACU.U.U..UGU..GUUGACGUCC..U.....UGA........................................................................................................
Ga0265756__105276/677-589      .UUGGUGAAACACU.U.U..UGU..GUUGACGUCC..U.....UGA........................................................................................................
Ga0153880__1339022/280-371     .UUGGUGAAACACU.U.U..UGU..GGUGUUGGUA..UCCU...UUA.......................................................................................................
Ga0247518__120275/202-289      .UUGGUGAAACACU.U.U..UGU..GUUC.UC.GU..C.....CUU........................................................................................................
Ga0265752__100023/1618-1523    .AUGGUGAAACAUC.G.U..AGA..GU................UGCG.......................................................................................................
Ga0307484__102966/353-250      .CUGGGGAAACACA.U.G..GAA.....................UUAA.C.G..................................................................................................
Ga0265752__101001/521-687      .UAGGGGAAACAUG.C.U..AGC..U.................AAGCC.G....................................................................................................
Ga0184593__121910/718-622      .AGAGUGAAACACU.U.C..UGU..UACCC..............UA........................................................................................................
Ga0184602__103319/96-192       .AGAGUGAAACACU.U.C..UGU..UACCC..............UA........................................................................................................
Ga0247528__108722/131-33       .AGAGUGAAACACU.U.C..UGU..UACCC.............UAUA.......................................................................................................
Ga0265757__143567/145-70       .CAAGGGAAACAUC.G.A..GGA..C.................GAA........................................................................................................
Ga0184592__121084/378-271      .CCAGGGAAACAUG.U.G..GGG..U.................UAGC.......................................................................................................
Ga0316037__172473/89-196       .CCAGGGAAACAUG.U.A..GAG..G.................UCGU.......................................................................................................
Ga0307921__1011071/674-791     .CGAGGGAAACAUG.C.A..AAA..GCAC..............GUCCA......................................................................................................
Ga0265744__124024/357-288      .CUGGGGAAACAUG.U.U..G......................CACG.......................................................................................................
Ga0307482__1000291/592-698     .UCGGUGAAACAUC.A.U..ACA..GU................AACG.......................................................................................................
Ga0184568__108869/934-1072     .UCGGUGAAACACG.C.U..CUU..CC................CCGGU...AA.................................................................................................
Ga0138276__1121602/329-410     .CAGGCAAAACAUG.G.C..CG.....................AUUAA.C....................................................................................................
Ga0138278__1041650/183-102     .CAGGCAAAACAUG.G.C..CC.....................GUUUA.A....................................................................................................
Ga0138277__1137481/570-489     .CAGGCAAAACAUG.G.C..C......................CGUCG...AA.................................................................................................
Ga0138278__1041788/293-378     .CAGGCAAAACAUG.G.C..CA.....................CCAAU.AAAAA................................................................................................
Ga0138275__1223952/371-283     .CAGGCAAAACAUG.G.C..CAC..CA................AUCUC.AAAAA................................................................................................
Ga0138277__1142657/273-361     .CAGGCAAAACAUG.G.C..CAC..CA................AUUUC.AAAAA................................................................................................
Ga0307482__1202605/45-154      .GAAGGGAAACAUG.G.A..GA.....................CUGCG...AA.................................................................................................
Ga0180107__1017343/1163-1243   .CCGGUGAAACACU.C.A..AAC.....................UAUC.ACA..................................................................................................
Ga0157560__1170105/128-245     .UGAGGGAAACCUC.U.A..GAU..AG....UGGA..CACGUCCGGAG.U....................................................................................................
Ga0316030__137115/325-257      .UGUGGGAAACAUU.C.U..CG.....................CUUAC.G....................................................................................................
Ga0316046__103474/576-502      .UUGGUGAAACACA.A.G..CUA..A.................UUCAU......................................................................................................
Ga0265754__1141843/290-185     .GGAGGGAAACAUG.U.C..AGC..G.................UAGC.......................................................................................................
Ga0222729__1080556/362-213     .CGAGGGAAACACG.A.C..ACG..AGC...............CAAGGAAUCCUAUUAUUGGACCUUGAAUCUACUCACAAGCUAUGCUGUGGUGAGAUGAUGU..............................................
Ga0265740__1020297/298-150     .ACGGGGAAACAUG.C.U..UU.....................UGA........................................................................................................
Ga0157569__1080558/455-605     .GCAGUGAAACAUG.G.U..ACA..AUAUUGUGUU..UGAGAUUCAA..UUUCA................................................................................................

                               .<<<<.......<<.<.<..<<<..<<<<<<<<<<..<<<<<<...........................................................................................................
                               .2222.......22.2.2..22................................................................................................................................
                               -●●RGGGAAACAY●-●-●--●○○--------------------●●●●○------------------------------------------------------------------------------------------------------
```

```
Ga0307484_101264/723-941        .UUGGUGAAACACG.C.C..UGC.....................CCGC.CGAA..........
Ga0213850_1513954/279-196       .UCGGUGAAACACG.C.A..UGG..GC................GAAA.................
Ga0316053_117700/109-179        .UGAGGGAAACAUG.U.G..U......................GCAA.................
Ga0184582_101997/109-1          .CCAGGGAAACAUU.C.U..UC.....................AUCUU................
Ga0184602_109114/416-317        .UCAGGGAAACAUA.C.U...........CUGU..UCCAGCUUGC.................
Ga0153880_1354682/355-454       .UCAGGGAAACAUA.C.U...........CUGU..UCCAGCUCUC.................
Ga0184602_110257/415-316        .UCAGGGAAACAUA.C.U...........CUGU..UCCAGCUCUC.................
Ga0307484_149222/136-234        .CUGGGGAAACAUG.C.A..AGG..G.................GUAGC.G..............
Ga0184572_111224/187-319        .UCAGGGAAACAUG.U.C..AUG.UCAGGGGAGGU..C.....AACC.................
Ga0138278_1022898/219-127       .UGGAGGAAACACG.C.G..UAA..GGU...............UGCUA................
Ga0184577_108791/182-295        .CCAGGGAAACACC.C.A..GCA..UC...........GCUUGGCUUG.................
Ga0265748_107452/280-387        .GGAGGGAAACACC.U.A..UGC..GUCG...................................
Ga0180107_1164033/560-431       .CUAGUGAAACACG.C.C..U......................UAA..................
Ga0032354_1086862/688-579       .UCAGGGAAACACA.G.U..GGU..GC.............CCGAUUA.................
Ga0265751_100560/274-382        .CUGGUGAAACAAU.A.C..CAU..CU................AACG.................
Ga0180107_1045142/740-541       .CCAGGGAAACAUG.G.C..GA.....................ACUUA.UGGCC..........
Ga0213850_1296465/305-234       .AUGGUGAAACAUG.C.A.........................UUAU.................
Ga0265757_111747/356-286        .AUGGUGAAACAUG.C.A.........................UUA..................
Ga0247553_104889/634-563        .AUGGUGAAACAUG.C.A.........................UUAU.................
Ga0265751_102572/231-125        .UGGGGGAAACAUU.U.U..GG.....................AUUC..ACGCA..........
Ga0265758_100017/4028-4134      .UGGGGGAAACAUU.U.U..GG.....................AUUU..ACGCA..........
Ga0180107_1307934/226-315       .UCGGGGAAACACA.C.C..UUG..CA................UCAUC................
Ga0184572_112077/359-259        .CUGGUGAAACAUC.A.U..GAA.....................GUA..GCG............
Ga0213850_1371453/186-298       .CUGGGGAAACACU.C.C...UG..GCCAUAAGCU..ACCUGAGUCAC................
Ga0213850_1469439/119-231       .CUGGUGAAACACU.C.C..UG...GCCAUCAGCU..AUC...CGAGUCGCACG..........
Ga0247535_113123/229-119        .CUGAAGAAACACU.U.A..UGG...UCACCUCUU..ACCGCUGAA..................
Ga0316036_112404/482-376        .CUGAAGAAACACU.U.A..UGG...UCACC.CCU..GUUGCCACUAA.A..............
Ga0179943_1118543/263-176       .CCAGGGAAACACC.A.C..UAC..CUGU..............UUAC.................
Ga0180108_1207316/715-601       .UCAGGGAAACACG.G.G..AG...........GA..UGCUAUCGCAU.UCACA..........
Ga0184578_120646/150-246        .UGAGGGAAACCAC.A.G..AGU..UCU...............UUUAA................
Ga0127503_10678853/419-333      .UGAGGGAAACACU.G.U..UC.....................GCUUC.AUAUG..........
Ga0307919_1065410/76-162        .UGAGGGAAACACU.G.U..UC.....................GCUUC.AUAUG..........
Ga0180108_1209503/1932-2029     .AAGGGGAAACACA.A.C..UG.....................AUUC..GCGAC..........
Ga0180108_1022680/3902-3998     .UAGGUGAAACACA.U.C..UG.....................CGUU..GCGAA..........
Ga0316031_107021/319-214        .GGAGGGAAACAUC.U.U..GA.....................ACUA..GCGAC..........
Ga0247527_116942/161-88         .CCAGGGAAACAUC.G.U..UUU....................UCUU.................
Ga0307484_100434/1330-1210      .UAGGGGAAACACU.C.A..CGA..........CU..C....UAUAAA................
Ga0307482_1160392/324-400       .UGGGGGAAACACA.U.C..UG.....................AUGGU.UGUGC..........
Ga0316033_105623/339-253        .UCGGUGAAACAUG.C.A..GAG..GUGGU.............CUUA...GA............
Ga0265754_1037910/322-179       .CUGGGGAAACAUG.C.A..UCG..CCU...............AACGC................
Ga0307482_1048753/383-528       .ACGGUGAAACAUG.C.U..CGC.....................CCGC.CGAA..........
Ga0265752_102479/223-81         .UCGGUGAAACAAA.C.A..AGG..AG................UAGAACCAUACAAGGAAUAUUUCAUCCAUCAGUAUGGCAUACUUUAC..........
Ga0265746_1084801/20-158        .UCGGUGAAACAAA.C.A..AGG..AG.UAGA......ACCAUAAAGGAGUUACCUCCAUCAU..........
Ga0265756_107066/520-660        .UCGGUGAAACAAA.C.A..AGG..AG.UAGA......ACCAUACAAGGAUUAUCAUCCAUCAGU..........
Ga0265742_1022009/340-200       .UCGGUGAAACAAA.C.A..AG.....................GAAUGGAACCAUACAAGGAUUUACAUCCAUCAGUACGGUAUACUUUACCCA..........
Ga0265748_106902/213-353        .UCGGUGAAACAAA.C.A..AG.....................GAAUGGAACCAUACAAGGAUUUACAUCCAUCAGUACGGUAUACUUUACCCA..........
Ga0180052_116799/419-501        .ACGGUGAAACACU.U.A..GAU..A.................AAA..................
Ga0115594_1106220/441-334       .CCGAGGAAACAUG.C.U..GAG..AGACAAACGC..U.....AGGCA................
Ga0247534_117029/65-195         .CCGGUGAAACACA.C.C..AGC..C.............CCUCGUGAA.AGAUC..........
JGI12272J11983_1072695/40-150   .GGAGGGAAACAUG.C.U..GGA..GU................AGCG.................
Ga0247552_100007/4311-4420      .CCAAAGAAACAAG.G.A..GG.....................CUUA..ACGCA..........
Ga0184596_107732/313-205        .CCAAAGAAACAAA.G.A..GG.....................CUGAC.G..CA..........
Ga0247542_100007/609-500        .CCAAAGAAACAAA.G.A..GA......................UUUA.ACGCA..........
Ga0247534_100047/1899-2008      .CCAAAGAAACAAA.G.A..GG.....................CUUA..ACGCA..........
Ga0184598_129111/512-403        .CCAAAGAAACAAA.G.A..GG.....................CUUA..ACGCA..........
Ga0184597_128933/573-464        .CCAAAGAAACAAA.G.G..GG.....................CUUA..ACGCA..........
Ga0184582_108821/1050-1159      .CCAAAGAAACAAA.G.A..GG.....................CUUA..ACGCA..........
Ga0247541_100010/3268-3377      .CCAAAGAAACAAG.G.A..GG.....................CUUA..ACGCA..........
Ga0184580_121439/573-464        .CCAAAGAAACAAA.G.A..GG.....................CUUA..ACGCA..........
Ga0265756_101275/650-541        .CCAAAGAAACAAG.G.A..GG.....................CUUG..ACGCA..........
Ga0184575_101094/586-477        .CCAAAGAAACAAA.G.A..GG.....................CUUG..ACGCA..........
Ga0184594_103311/207-316        .CCAAAGAAACAAG.G.A..GG.....................CUUA..ACGCA..........
Ga0247554_100006/1592-1485      .CCAAAGAAACAAA.G.G..GG.....................CAAC...GCA..........
Ga0247522_108409/684-554        .CCGGUGAAACAUG.C.U..CGC....................CCGCC.GAA...........
Ga0247534_100003/3147-3245      .AUGGUGAAACAUC.A.U..AG.....................CGUU..GCGAA..........
Ga0265751_100393/1594-1698      .GAGAGGAAACAUA.C.C..AG.....................AUUA..GCGAC..........
Ga0265751_124807/392-283        .CCGGUGAAACACC.G.U..CG.....................CUUAA.C.G............
Ga0310107_144222/327-436        .CCGGUGAAACACC.G.U..CG.....................CUUAA.C.G............
Ga0265743_103007/946-842        .GAAGGGAAACACA.U.G..AUC..G.................UAGC.................
Ga0316038_107364/55-195         .UCAGUGAAACAUU.A.G..UGU...CGUUCCGG.........AAAUC.AA.............
Ga0184593_123391/157-297        .UCAGUGAAACAUU.A.G..UGU...CGUUCCGG.........AAAUC...ACA..........
Ga0222728_1003482/1262-1107     .UGAGUGAAACACG.A.U..CGC....................CUUAA.CGGCA..........
Ga0242654_10701525/53-296       .CCGGGGAAACACU.A.U..GG.....................CUAUA.UGGCG..........
Ga0242654_11172691/285-48       .CCGGGGAAACACU.A.U..GG.....................CUAUA.UGGCG..........
Ga0316038_100042/1676-1569      .GGAGGGAAACACC.U.A..UG.....................AUUA..GCGAC..........
Ga0307921_1043977/91-164        .AUAGGGAAACACG.A.G.........................GCAAA................
Ga0079039_1293848/95-22         .CUGGUGAAACACG.C.A..GA.....................UGUC.................
Ga0247534_101560/228-122        .ACGGUGAAACACA.G.U..GG.....................CUUA..GCGCA..........
Ga0153880_1379927/66-144        .GGGUGGAAACAUG.C.U..UGG....................AAAG.................
Ga0153880_1391790/62-140        .GGGUGGAAACAUG.C.U..UGG....................AAAG.................
Ga0307482_1048660/422-625       .UCAGGGAAACAUG.C.U..GGC.....................CGGC.CGAA..........
Ga0180108_1022680/4248-4153     .CCAGGGAAACAUA.U.G..GUC..GU................AGCG.................
Ga0247516_125012/316-428        .CCGGUGAAACACA.G.C..CUG..GG................AUUAA.C.CU...........
Ga0242652_1038498/365-470       .AGAGGGAAACACU.GCA..CUG..G.................UUCGA.A..............
Ga0210323_1050281/92-187        .CCAGGGAAACACA.U.G..UG.....................UCUA..GCGC...........
Ga0224712_10542545/154-227      .AGAGGGAAACACA.G.U..AG.....................CUUAC.UAGGC..........
Ga0184602_130775/1275-1431      .CAGGGGAAACAUG.C.G..UAC......UGGCCU..CUUG..ACCUGCUCGGCUUAUGCUAUACAGCAGCGUG..........
Ga0206355_1311803/144-334       .CCAGGGAAACACG.U.A..UUG..UGUGCU............GGGUGAUAAGAUGUCUUUUCGAACAUUUUCUCUCUCCCCCACAUGUGUGAGGGUCAAU..........
Ga0310119_152175/242-389        .CAAGGGAAACAUG.C.G..UG......CAUGCAU..CGUUACCACAGUGGUGAAUCACCG..........

                                .<<<<.......<<.<.<..<<<..<<<<<<<<<<..<<<<<<.....................
                                .2222.......22.2.2..22..........................................
                                -●●RGGGAAACAY●-●-●--○○○-----------------------●●●●○-------------
```

```
Ga0255059_10691632/166-76       .CCAGGGAAACAUA.C.C..CGG..AA................CGAA........................................................................................................
Ga0255062_11230321/200-110      .CCAGGGAAACAUA.C.C..CGG..AA................CGAA........................................................................................................
Ga0265743_143797/241-325        .CUGGUGAAACACC.G.A..C......................GGCGA.A.....................................................................................................
Ga0247534_117055/74-212         .CCAGGGAAACAUA.G.U..UAG..UACCAGUGAC..CGGGCCAGCG........................................................................................................
Ga0213850_1282564/267-350       .UCGGUGAAACACG.U.C..GU.....................UGCG........................................................................................................
Ga0315826_123252/262-347        .UCGGUGAAACACG.U.C..GU.....................UUUGC.A.....................................................................................................
Ga0315817_133437/498-413        .UCGGUGAAACACG.U.C..GU.....................UUUGC.A.....................................................................................................
Ga0184577_116723/415-311        .GCAGUGAAACACG.U.G..U................CCUAUCUA..........................................................................................................
Ga0184576_109717/432-536        .GCAGUGAAACACG.U.G..U................CCUAUCUA..........................................................................................................
Ga0184572_113323/230-300        .AGGGUGAAACACG.U.G..U......................AUA.........................................................................................................
Ga0179935_1139075/165-78        .CCAGUGAAACAUG.C.G..UAA..GUAG..............UAACU.G.....................................................................................................
Ga0179944_1246019/249-162       .CAAGUGAAACAUG.C.A..GAA..GUAG..............UGUA........................................................................................................
Ga0213850_1307525/101-186       .AGAGAGAAACAUA.A.C..GUC..G.................AGCC..AGAAA.................................................................................................
Ga0222728_1062510/166-66        .UGAGGGAAACAUC.U.C..A......................UAA.........................................................................................................
Ga0265743_101198/448-619        .CCAGGGAAACACA.U.G..AUG....................CUAU.UG.....................................................................................................
Ga0180108_1209503/2438-2343     .CCAGGGAAACACA.U.A..GUC..GU................AACG.A......................................................................................................
Ga0184583_103998/587-740        .UCGGGGAAACAUG.C.U..AGC..C.................AGCCA.A.....................................................................................................
Ga0184595_100702/414-262        .UCGGGGAAACAUG.C.U..AGC..C.................AGCCG.A.....................................................................................................
Ga0184592_125105/67-219         .UCGGGGAAACAUG.C.U..AGC..C.................AGCCA.A.....................................................................................................
Ga0224712_10002011/1564-1454    .GGAGGGAAACACA.U.C..AGC..GU................AACG........................................................................................................
Ga0316049_114344/181-288        .GUAGGGAAACAUG.C.G..UCG..CAG...............CCGU........................................................................................................
Ga0184576_110594/6-113          .GUAGGGAAACAUG.C.G..UCG..CAG...............CCGU........................................................................................................
Ga0316037_112281/325-219        .GUAGGGAAACAUG.C.G..UCG..CAG...............CCGU........................................................................................................
Ga0316046_116376/278-384        .GUAGGGAAACAUG.C.G..UCG..CAG...............CCGU........................................................................................................
Ga0247534_102735/124-18         .GUAGGGAAACAUG.C.G..UCG..CAG...............CCGU........................................................................................................
Ga0247540_102569/210-104        .GUAGGGAAACAUG.C.G..UCG..CAG...............CCGU........................................................................................................
Ga0247529_116306/255-362        .GUAGGGAAACAUG.C.G..UCG..CAG...............CCGU........................................................................................................
Ga0184568_104528/922-1000       .UGAGGGAAACAUG.G.A..GUU..C..................UAAC...A...................................................................................................
Ga0184578_112031/156-44         .UGAGGGAAACAUA.G.U..GUU..GUGGCUAGAU........AAU.........................................................................................................
Ga0247517_121471/387-499        .UGAGGGAAACAUA.G.U..GUU..GUGGCUAGAU........AGU.........................................................................................................
Ga0184569_108885/200-88         .UGAGGGAAACAUG.U.A..AAG..GUGAGUGGUU..G.....AUUU........................................................................................................
Ga0247521_112559/317-205        .UGAGGGAAACAUG.U.A..AUU..GUGAGUGGUU..G.....AUUU........................................................................................................
Ga0247539_101233/414-526        .UGAGGGAAACAUG.U.A..AUG..GUGAGUGGUU..G.....AUUU........................................................................................................
Ga0265756_100485/1108-1000      .GCGGUGAAACACC.G.G..AUU..GU................UGACA.......................................................................................................
Ga0265751_100417/1480-1588      .GCGGUGAAACACC.G.G..AUU..GU................UGACA.......................................................................................................
Ga0213850_1295854/263-370       .UGAGGGAAACACU.U.U..GCG..AGUCAACCAA..CGA...ACUA........................................................................................................
Ga0307919_1029784/253-144       .UGAGGGAAACACU.U.U..GCG..AGGAAACCAA..C...GAAUCAA.A.....................................................................................................
Ga0307921_1042549/343-452       .UGAGGGAAACACU.U.U..GCG..AGGAAACCAA..C...GAAUCAA.A.....................................................................................................
Ga0307484_102966/727-825        .GGAGGGAAACAUU.U.G..CU.....................AUUA..GCGAA.................................................................................................
Ga0184588_131258/156-253        .AUAAGGAAACACA.C.U..AU.....................UAA.........................................................................................................
Ga0224712_10010317/1973-2082    .GGAGGGAAACAUU.U.C..AG.....................CCUA..GCGAA.................................................................................................
Ga0224712_10601975/393-502      .GGAGGGAAACAUU.U.C..AGU.....................GUA..GCGA..................................................................................................
Ga0222728_1000118/2272-2196     .UCGGUGAAACACG.U.U..UGA..G.................CACGC.......................................................................................................
Ga0115594_1200134/154-31        .UCGGUGAAACAUC.U.G..UAGACGGGAAAAGGC..CUGUGCACA.........................................................................................................
Ga0115595_1153837/320-197       .UCGGUGAAACAUC.C.A..UAGACGGGAAAAGGC..CUGUGCACA.........................................................................................................
Ga0255282_1067513/33-105        .GCGGUGAAACCUC.C.C..UA.....................CGCA........................................................................................................
Ga0242647_1005934/451-617       .CCGGGGAAACACA.U.C..GG.....................CUUUA.CGGCA.................................................................................................
Ga0307482_1449537/408-223       .UCAGUGAAACAUG.C.G..GGC....................CUUA..UGGCA.................................................................................................
Ga0115594_1022316/346-239       .CCGAGGAAACAUG.U.U..GAG..AGA...............CAAACGACAGGCAGU.............................................................................................
Ga0115595_1072788/334-441       .CCGAGGAAACAUG.C.U..GAG..AGA...............CAAACGACAGGCAGU.............................................................................................
Ga0184568_108419/50-147         .AGAGGGAAACAUC.U.G..UUA.....................GUA..GCG...................................................................................................
Ga0247534_100007/116-225        .GGAGGGAAACAUC.U.A..GAC..G.................UUGCG.......................................................................................................
Ga0184569_109084/220-111        .GGAGGGAAACAUC.U.A..GAC..GU.................CGC........................................................................................................
Ga0316033_100036/207-98         .GGAGGGAAACAUC.U.A..GAC..GU.................CGC........................................................................................................
Ga0184578_112042/249-140        .GGAGGGAAACAUC.U.A..GAC..G.................UUGCG.......................................................................................................
Ga0184594_123034/256-94         .CCAGGGAAACACG.C.U..GCG..A.................CUCGGAGUCACCUCCUCCAGUCCUCAAGAGAACCAUCGGACCUGUGGAAAUCCGGCAAUGAAGUCGGACGCCCCACAGUAACACC......................
Ga0256798_1305521/221-305       .GUGGGGAAACAUG.C.U..G......................GCACA.G.....................................................................................................
Ga0184595_116681/414-266        .UCGGUGAAACAUG.C.U..CGC.....................CCGC.CGAA..................................................................................................
Ga0184568_105547/129-239        .CGAGGGAAACAUG.C.G.........................UUUAA.......................................................................................................
Ga0157559_1099797/360-209       .CCAGGGAAACAUU.C.A..AUG..AAG...............AAGCA...C...................................................................................................
Ga0157572_1089974/360-209       .CCAGGGAAACAUU.C.A..AUG..AAG...............CAGCA.C.....................................................................................................
Ga0316037_108094/19-160         .CUAGGGAAACACC.C.G..UC.....................GCGCAAGUAACAUCGAAGGGAGCGUAAUAAAAAGAUUUUCGGUUCUGCGACG........................................................
Ga0316038_107116/33-174         .CUAGGGAAACACC.C.G..UC.....................GCGCAAGUAACAUCGAAGGGAUCUUAAUAAAAAGAUUUUCGGUUCUGCGACG........................................................
Ga0210323_1072445/691-543       .UCGGUGAAACACC.G.A..CAG..CC................CUGGC.U...A.................................................................................................
Ga0307482_1000707/704-773       .UCGGGGAAACACA.U.C..A......................GAGA........................................................................................................
Ga0079039_1671802/197-34        .CCAGGGAAACACU.C.U..GUG..AG................AGUUA.GGG...................................................................................................
Ga0247533_103342/137-220        .CGGAGGAAACAUG.U.U..G....GAAGAG............GAAA........................................................................................................
Ga0157565_1016893/233-334       .UCGGUGAAACACG.C.A..CAA..C.......CU..CUCGAUCUCA........................................................................................................
Ga0180047_1087048/281-180       .UCGGUGAAACACG.C.A..CAA..C.......CU..CUCAAUCUCA........................................................................................................
Ga0157593_1006201/239-338       .UCGGUGAAACACA.C.A..UAA..CCUCUCAAAG..C.....UUAC........................................................................................................
Ga0157560_1010344/357-456       .UCGGUGAAACACA.C.A..CAA..CCUCUCAAAG..C.....UUAC........................................................................................................
Ga0316049_154526/155-74         .UGGAGGAAACAUG.C.A.........................UAAC........................................................................................................
Ga0316035_177575/224-142        .UGGAGGAAACAUG.C.A.........................UAAC........................................................................................................
Ga0316033_101029/151-49         .ACAGGGAAACAUC.A.G...C.....................GCGUAGCGA...................................................................................................
Ga0206356_10600373/215-21       .GCAGGGAAACACU.C.A..CGU..UUUA..............UAAGGUAGACGCGAUAACUUUUCGAUUAUCAACUCUCCCCCAUGGUCAAGAUAAAUUGACAUGAUCUUGGAGCACGUC...............................
Ga0307482_1009758/562-669       .GGAGGGAAACAUG.U.C..AG.....................CGUA..GCGAA.................................................................................................
Ga0213850_1245299/581-409       .UCAGGGAAACACG.C.G..GG.....................AUACG.AAACC.................................................................................................
Ga0180107_1134582/382-542       .GCAUGGAAACACU.G.U..GGG.....................AUUU.GACC..................................................................................................
Ga0307484_100970/951-1026       .CUGGUGAAACACA.C.U..UCC....................AUUA........................................................................................................
Ga0307919_1034281/527-451       .UGAGGGAAACACU.U.C..AU.....................UAUAU.......................................................................................................
Ga0307921_1020694/406-483       .UGAGGGAAACACU.U.C..AU.....................UAUAU.......................................................................................................
Ga0316033_105623/3-98           .UGAGGGAAACACA.C.C.........................GCGAGAGGCAUCUCAA............................................................................................
Ga0265743_111644/592-512        .GCAGGGAAACAUGUC.C..UAG..GU................AGCAA.......................................................................................................
Ga0265755_108708/373-453        .GCAGGGAAACAUGUC.C..UAG..GU................AGCAA.......................................................................................................
Ga0316038_121313/184-264        .GCAGGGAAACAUGUC.C..UAG..GU................AGCAA.......................................................................................................
Ga0265756_109326/116-196        .GCAGGGAAACAUGUC.C..UAG..GU................AGCAA.......................................................................................................
Ga0265759_104327/339-243        .UGAGGGAAACACA.G.G..U...........GU..CG...UGAAAU.GAGCU..................................................................................................
Ga0316035_144545/195-301        .UGAGCGAAACACC.A.C..GCA..GAUU..............UGGCCUUUAAGCCUUUCCUGAC......................................................................................
Ga0265743_108998/150-253        .AGAGGGAAACAUG.U.C..AC.....................CCUA..GCGAA.................................................................................................

                                .<<<<.......<<.<.<..<<<..<<<<<<<<<<..<<<<<<............................................................................................................
                                .2222.......22.2.2..22.................................................................................................................................
                                -●●RGGAAACAY●-●-●--○○○------------------------●●●○○-----------------------------------------------------------------------------------------------------
```

```
Ga0153880_1067318/492-566       .CGAAGGAAACAUA.C.A..UA.....................CUUAU.C.....................................................................................................
Ga0180044_1047678/444-368       .CUGGUGAAACAUG.C.U..UU.....................CAAAG.......................................................................................................
Ga0157564_1065218/271-353       .CUAGUGAAACAUG.C.A..UUG....................AAAAA.A.....................................................................................................
Ga0180044_1069332/269-187       .CUAGUGAAACAUG.C.A..UUG....................AAAAA.A.....................................................................................................
Ga0242647_1014694/423-336       .CAAGGGAAACACA.U.C..U......................AUUA.CGUC..................................................................................................
Ga0180108_1143860/199-116       .GCAGGGAAACACA.U.C..C......................CAAG........................................................................................................
Ga0265744_100073/1235-1401      .CCAGGGAAACAUC.C.U..GUU..G.................CGUG........................................................................................................
Ga0307482_1054608/326-205       .UUAGGGAAACAUG.A.C.........................AACUCAUUCUUCCGUGAUCCUUCGGAUGAGGCCCUACUGGACCCUUAGGAAGUUUACAA.................................................
Ga0242646_1008525/229-119       .UUAGGGAAACAUG.A.C.........................AACCUGCUCUUCCAUAGAUCUAUUUCUAGACCCUUAGGAAGUUUACAA............................................................
Ga0153880_1260556/441-589       .CAGAGGAAACAUG.C.A..UAC..AG................UAGUAUAACCUACAAAUCAAUAUCAACUAAAAUAUUGUAAGUAGGACACU..........................................................
Ga0127503_10814644/496-352      .UCAGGGAAACAUA.A.A..UUC...AGAGAGUG.........AGCCG.......................................................................................................
Ga0326631_105052/171-77         .CCAGGGAAACAUG.C.U..C......................GUAAU.......................................................................................................
Ga0307920_126542/349-261        .CUAGGGAAACACG.C.C..A......................CACU........................................................................................................
Ga0247533_100162/1643-1535      .AGAGGGAAACACA.G.U..AG.....................AAGUG.GCGCA.................................................................................................
Ga0316047_100015/734-626        .AGAGGGAAACACA.G.U..AG.....................AAGUU.GCGAU.................................................................................................
Ga0265743_101537/1064-984       .CUGGGGAAACACC.A.C..U......................UCAA.......................................................................................................
Ga0316030_100208/805-700        .AGAGGGAAACAUG.A.U..GA.....................CUUA..GCGAC.................................................................................................
Ga0316050_103193/566-460        .AGAGGGAAACAUA.U.C..AC.....................CGUAA..CGAA.................................................................................................
Ga0184601_113231/247-149        .CCGGUGAAACACA.C.G..AG.....................GGCGA.......................................................................................................
Ga0307350_148584/139-224        .UGAGGGAAACAUU.C.C..GCU..CACUCA............UGAAU.......................................................................................................
Ga0307482_1018929/1182-1081     .CCGGGGAAACAUG.U.C..AGC..G.................UAGC........................................................................................................
Ga0307482_1000262/4800-4699     .CCGGGGAAACACG.U.C..AGC..G.................UAGC........................................................................................................
Ga0316047_100860/1040-888       .UCAGGGAAACAUG.C.A..CUG..GU................UUGC.UG....................................................................................................
Ga0242654_10068518/113-218      .GAAGGGAAACACU.U.G..AGG..U.................CGACA.A.....................................................................................................
Ga0222729_1010500/11-117        .GAAGGGAAACACU.U.G..AGG..UC................UGAC.A.....................................................................................................
Ga0307484_101172/602-495        .GAAGGGAAACACU.U.G..AGG..UC................UGAC.A.....................................................................................................
Ga0242647_1002406/915-1021      .GAAGGGAAACACU.U.G..AGG..UC................UGAC.A.....................................................................................................
Ga0307483_1000720/1178-1064     .GGAGGGAAACACU.C.A..ACA..GU................GGUC........................................................................................................
Ga0307921_1022947/450-369       .UGAGGGAAACAUG.C.C..AAC..CG...............CCACCG.U.....................................................................................................
Ga0153880_1432604/289-395       .CGGGGGAAACACA.G.A..CAU..GA................UCAUU.GCGAC.................................................................................................
Ga0247533_101442/511-432        .UGAGGGAAACAUG.C.U..GUA..U.................CGCCU.......................................................................................................
Ga0242647_1002473/759-521       .CCAGGGAAACACG.U.U..GG.....................GUAUC.UGGCC.................................................................................................
Ga0256352_1087084/23-118        .AAGGUGAAACACC.G.C..C................AGCAAUUUA.........................................................................................................
Ga0157562_1157806/189-74        .UAGGUGAAACACC.G.C..GUG..CUGAAAUGA.........UAAGCAUAAUUCUUAAAUUAGUAU....................................................................................
Ga0153880_1457361/236-121       .UAGGUGAAACACC.G.C..GUG..CUGAAAUGAU..AGGCAUAAUUC.UUAAA.................................................................................................
Ga0157559_1128929/201-87        .UAGGUGAAACACC.G.C..GUG..CUGAAAUGAU..AAGCAUAAUUC.UUAAA.................................................................................................
Ga0157566_1012736/497-384       .UAGGUGAAACACC.G.C..GUG..CUGAAAUGAU..AAGCAUAAUUC.UUAAA.................................................................................................
Ga0157579_1155702/323-436       .UAGGUGAAACACC.G.C..GUG..CUGAAAUGAU..AAGCAUAAUUC.UUAAA.................................................................................................
Ga0316036_116159/257-326        .UGAGGGAAACACC.A.U..UC.....................AAG.........................................................................................................
Ga0180047_1032966/152-37        .UAGGUGAAACACC.G.C..GU.....................GAUGAAAUGAUAAGCAUAAUUCUUAAAUUAGUAUUCUGAUUUGA................................................................
Ga0184587_125934/78-152         .CUGGGGAAACAUG.G.U..GG.....................UGAAA.......................................................................................................
Ga0179934_1155244/114-26        .UGAGGGAAACAUG.C....CGU..UAGAAU............GAAAA.A.....................................................................................................
Ga0307482_1623775/241-347       .GGAGGGAAACACG.U.G..CG.....................CUUA..GCGAA.................................................................................................
Ga0307484_101582/1018-1124      .GGAGGGAAACACG.U.G..CG.....................CUUA..GCGAA.................................................................................................
Ga0307484_111921/444-550        .GGAGGGAAACACG.U.G..CG.....................CUUA..GCGAA.................................................................................................
Ga0184587_113157/56-168         .CCGGUGAAACACG.C.G..GCA..UC................CCAG.G.....................................................................................................
Ga0255228_1272153/272-192       .UUAGGGAAACACA.A.G..UA................GCGUUCUA.........................................................................................................
Ga0153880_1325448/483-575       .UUAGCGAAACAUG.C.U..UAC..CCAGA..........UUCUGAGUGAAU...................................................................................................
Ga0157565_1130498/91-183        .CUAGUGAAACAUG.C.U..UAC..CCAGA..........UUCUGAGUGAAU...................................................................................................
Ga0265758_129208/41-120         .UCAGGGAAACACG.U.U..GA.....................GUCGCGAGUGA.................................................................................................
Ga0265756_146329/172-94         .UCAGGGAAACACG.U.A..AA.....................GUUGG.AGCGA.................................................................................................
Ga0115594_1180137/311-161       .CCAGGGAAACAUC.G.C..GGG..GG................GGGUU.G.....................................................................................................
Ga0265744_100861/398-567        .CCAGGGAAACACG.U.G..GUG....................CUAU.UG....................................................................................................
Ga0180107_1285918/13-113        .GUAGGGAAACACC.G.C.........................UUG.........................................................................................................
Ga0247519_103291/1381-1460      .GUGGGGAAACACC.A.U..C......................GAUG........................................................................................................
Ga0184603_106561/141-62         .GUGGGGAAACACC.A.U..C......................AAUG........................................................................................................
Ga0247551_100996/477-556        .GUGGGGAAACACC.A.U..C......................AAUG........................................................................................................
Ga0184590_126350/300-380        .GUGGGGAAACACC.A.U..C......................AAUA........................................................................................................
Ga0265756_131580/159-58         .CGAGGGAAACAUA.C.C..A...........CGC..AAGCGUCGAA........................................................................................................
Ga0307482_1000325/24-131        .CUGGUGAAACAUU.A.U..CGA....................UUAA.C.G...................................................................................................
Ga0242646_1001086/737-571       .CCAGGGAAACAUG.C.A..UG.....................UUUAG.G.....................................................................................................
Ga0265747_132694/181-66         .UCAGGGAAACAUG.A.C..UGA..CUUUGU............GCACUAAUCUCUUUUUCUUCGAGAUGAUAGUGACUG........................................................................
Ga0157593_1023702/238-314       .UUGGUGAAACACG.C.U.........................ACAAC.AAUA..................................................................................................
Ga0316053_103654/814-736        .AUGGCAAAACACG.G.G..AAA..A.................UAAA........................................................................................................
Ga0247529_117158/253-331        .AUGGCAAAACACG.G.G..AAA..A.................UAAA........................................................................................................
Ga0180107_1129325/244-60        .UCAGGGAAACACU.C.A..GU.....................UCGAG.C.....................................................................................................
Ga0307482_1162833/232-148       .UCAGGGAAACACG.A.C..GGC..UCACAG............AAAA........................................................................................................
Ga0255267_1268184/91-184        .UCGGUGAAACACG.C.C.........................UACG........................................................................................................
Ga0307919_1064641/225-73        .AGAGGGAAACACG.C.U..CUA......................CCU.GGC...................................................................................................
Ga0307921_1075885/188-36        .AGAGGGAAACACG.C.U..CUA......................CCU.GGC...................................................................................................
Ga0265742_1021317/385-459       .UGGGGGAAACACG.U.G.........................GAAA........................................................................................................
Ga0307483_1002304/791-713       .CUGGUGAAACACG.G.U.........................UGAC........................................................................................................
Ga0307919_1165008/203-116       .UGAGGGAAACACA.C.A........GCCUA...........ACCA.C.AGAAA.................................................................................................
Ga0210323_1093898/306-192       .CCAGGGAAACACG.C.GAUGUA..GCAACCAUCU..AAGAACAAAGUGUUCUAAUAUGUCCA........................................................................................
Ga0265743_119695/104-184        .GUCGGGAAACAUG.C.U..GAA....................UAAGU.......................................................................................................
Ga0310118_104706/1022-1118      .GCAGGGAAACACC.A.U..UGU..UUAUUAGACU..C.....UUA.........................................................................................................
Ga0307921_1005950/551-460       .CGAGGGAAACACG.A.C..AA.....................UGAUA.......................................................................................................
Ga0242641_1014724/551-384       .CCAGGGAAACAUC.G.G..CC.........CGGA..C.....CAUUA....CA.................................................................................................
Ga0242641_1002366/499-331       .CCAGGGAAACAUC.G.G..CC.........CGGA..C.....CAUCA....CA.................................................................................................
Ga0242657_1007384/290-215       ACCAGGGAAACAAC.U.A..UGA..C.................UUAU........................................................................................................
Ga0265757_113513/277-362        .UGAGGGAAACACA.C.C..A..................CCAGGACA........................................................................................................
Ga0242646_1001007/790-609       .UCAGGGAAACACU.C.U..GUC.......AGGGC..U.....AUUAC.......................................................................................................
Ga0316034_107719/314-205        .GGAGUGAAACAUG.U.C..AA.....................CUUA..GCGAA.................................................................................................
Ga0316039_119781/347-457        .GGAGGGAAACAAG.A.U..GA.....................ACUA..GCGAC.................................................................................................
Ga0255295_1078742/236-408       .CCAGGGAAACAAC.A.U..CUG...AUC..............AACCUGCAAAUCAUUUGUGAAAAUAAUCGCGUUGAUCAAAAUACUAGCGGCGGUACUCGCACGCCAGCAAAUGACCAUU.............................
Ga0184603_150346/629-518        .AGGGAGAAACACG.U.U..AG.....................AUUA..GCGAC.................................................................................................
Ga0180106_1187148/166-89        .GUGGGGAAACACA.U.C..CU.....................CUUAG.G.....................................................................................................
Ga0307482_1417159/374-296       .GUGGGGAAACACA.U.C..CU.....................CUCAC.G.....................................................................................................
Ga0222728_1000095/4027-4133     .GAGGGGAAACAUA.U.C..AG.....................CUUA..GCGAC.................................................................................................

                                .<<<<.......<<.<.<..<<<..<<<<<<<<<<..<<<<<<............................................................................................................
                                .2222.......22.2.2..22.................................................................................................................................
                                -●●RGGGAAACAY●-●-●--●○○--------------------●●●●○-------------------------------------------------------------------------------------------------------
```

```
Ga0316030_105840/623-504          .CAAGGGAAACACA.C.A..CA....................AAAC..AAAAA................................................................................................
Ga0316033_110175/528-409          .CAAGGGAAACACA.C.A..CA....................AAAC..AAAAA................................................................................................
Ga0326631_124620/105-175          .CAAGGGAAACACA.C.A..CA....................AAAC..AAAAA................................................................................................
Ga0206356_11590518/224-68         .CAAGGGAAACACU.C.C..AU......CAUAGCC..G....GUACCGCAUGAACCAUCCAGUUCAUAAAGCUAUACUG......................................................................
Ga0213850_1246156/171-47          .UCAGGGAAACAUG.C.G..AUG..UGCC.............AGU........................................................................................................
Ga0184599_140450/516-588          .GCAGGGAAACACC.G.A..U.....................CUAU.......................................................................................................
Ga0206356_11002907/329-148        .CCAGGGAAACACA.U.G..AAA..AUU..............AUGGUGGUGAAGAAAGAAAACUUUAUUCUAAACUUCCCCCGUAUAGACUCUAAUUUUGGAAAUUUAGUCUCUCUACGGAGCUCC.......................
Ga0307482_1001469/2898-2828       .UUGGGGAAACACA.C.C........................GAAA.......................................................................................................
Ga0157570_1089798/68-159          .GAGAGGAAACACC.U.C..UC...............CCCCCGCU........................................................................................................
Ga0127503_11028958/51-130         .UGAGGGAAACACG.U.A..C.....................UAA........................................................................................................
Ga0157560_1126722/83-169          .CCAGGGAAACACA.A.G..UGU..G................ACU........................................................................................................
Ga0138277_1028338/433-516         .UGAGGGAAACAUA.C.U..A.....................GAAU.......................................................................................................
Ga0265742_1001214/55-127          .UGAGGGAAACAUG.C.A..AA....................GUUAA.U....................................................................................................
Ga0115594_1134035/652-542         .UGAGGGAAACAUG.C.A..UAG..AGAUAC...........ACUAG.GGGU.................................................................................................
Ga0115594_1212468/8-118           .UGAGGGAAACAUA.C.A..UAG..AG.AUAC..........ACUUG.G....................................................................................................
Ga0247533_100438/1040-888         .UCAGGGAAACAUG.C.A..CUG..GU................UUGC.UG...................................................................................................
Ga0326631_117973/181-293          .CUCGGGAAACACG.C.A..CGA..GCGG.............CUCAGAUUAA.................................................................................................
Ga0184600_117644/32-108           .GUAGGGAAACAUC.A.U..C.....................UUCG.......................................................................................................
Ga0184584_133603/317-393          .GUAGGGAAACAUC.A.U..C.....................CUUG.......................................................................................................
Ga0316052_100527/264-188          .GUAGGGAAACAUC.G.U..C.....................UUCG.......................................................................................................
Ga0184580_125947/153-229          .GUAGGGAAACAUC.A.U..C.....................UUCG.......................................................................................................
Ga0247524_105844/561-637          .GUAGGGAAACAUC.A.U..C.....................UUUG.......................................................................................................
Ga0184580_109821/480-551          .GUGGGGAAACAUC.A.U..GA.....................UUA.......................................................................................................
Ga0184598_107135/96-25            .GUGGGGAAACAUC.A.U..G.....................AUUAC......................................................................................................
Ga0316051_1015080/488-559         .GUGGGGAAACAUC.A.U..G.....................AUUAC......................................................................................................
Ga0247524_121352/374-303          .GUGGGGAAACAUC.A.U..G.....................AUUAC......................................................................................................
Ga0179934_1197833/207-115         .GAAGGGAAACACU.C.U........................ACCA.......................................................................................................
Ga0316049_129960/371-262          .UGAGGGAAACAUG.C.A..GAC..GA.GUUG.....GUGACUGUGG.A....................................................................................................
Ga0265747_125301/405-306          .CCAAGGAAACAUA.C.A..UAC..............ACUUCAAUAG.UCAUA................................................................................................
Ga0157570_1034241/151-229         .ACGGUGAAACACG.U.G..CAG..C................UAG........................................................................................................
Ga0157571_1064089/212-134         .ACGGUGAAACACG.U.G..CAG..C................AAG........................................................................................................
Ga0242652_1041630/308-377         .GCAGGGAAACACU.A.C..CA.....................GACA......................................................................................................
Ga0307480_1006257/684-599         .CUAGAGAAACACU.G.C..AC....................CCA........................................................................................................
Ga0310119_142698/346-430          .CCAGGGAAACACU.U.G..AUC..GACGCCG.....................................................................................................................
Ga0307482_1074296/478-354         .UCAGCGAAACACC.G.U..UA..............AGGCGCAUCA.......................................................................................................
Ga0307483_1005049/26-150          .UCAGCGAAACACC.G.U..UA..............AGGCGCAUCA.......................................................................................................
Ga0265757_127696/205-308          .CAAGGGAAACAUG.C.G..UUG........AAA..CCACUCUUUC.......................................................................................................
Ga0316029_101202/440-551          .CCAGGGAAACAUC.G.U..GA.....................CUA..GCGA.................................................................................................
Ga0307482_1134687/287-181         .GAAGUGAAACACG.U.C..GAA.....................UUAA.C.G..................................................................................................
Ga0307484_104189/399-292          .GAAGUGAAACACG.U.C..GAA.....................UUAA.C.G..................................................................................................
Ga0307921_1050271/251-412         .AGAGGGAAACACG.C.C..UUA...................CCUAG......................................................................................................
Ga0184580_102899/442-582          .CCAAGGAAACACG.U.U..AGU..GCCA.............GGGA.......................................................................................................
Ga0316029_136733/88-8             .CAAGGGAAACAUG.U.C..CA.....................CAGU.GCAA.................................................................................................
Ga0180107_1174136/218-102         .CCAGGGAAACCUU.C.U........................UCG........................................................................................................
Ga0179948_1254202/198-296         .CUAGCAAAACACC.G.C..GUU..UAAUAUGUUU..A....UUUGG.UU...................................................................................................
Ga0265746_1140777/214-319         .CAAGGGAAACAUG.C.U..GGU..CAACACACUC.......UUUC.......................................................................................................
Ga0265756_108694/382-277          .CAAGGGAAACAUG.C.U..GGU..CA......A..CACACUAUUUC.G....................................................................................................
Ga0265758_129717/201-94           .CAAGGGAAACAUG.C.U..GGA..GAACAAACUC.......UUUC.......................................................................................................
Ga0213850_1394829/253-105         .CCAGGGAAACAUC.A.U..CGU..GU...............AGAAAUUGCGGA...............................................................................................
Ga0184603_139562/141-280          .CGGAGGAAACAUG.A.U..UUG..UUCCUGCC.....CCCCCGAACCUCAAUCCAAGCCUUAUUCAAGGGCCCAGAGA......................................................................
Ga0157560_1100546/299-385         .AUAGGGAAACACG.U.G..UAA..A.................AAAG.UAAGA................................................................................................
Ga0129331_1494381/292-368         .CAAGGGAAACAUC.G.G..UC....................GGA........................................................................................................
Ga0265759_100473/818-988          .UCAGGGAAACACG.U.A..GG....................CUAUU.UGGCA................................................................................................
Ga0265746_1003835/1049-1219       .UCAGGGAAACACG.U.A..GG....................CUAUU.UGGCA................................................................................................
Ga0265756_100592/1458-1288        .UCAGGGAAACACG.U.A..GG....................CUAUU.UGGCA................................................................................................
Ga0247536_100773/637-807          .UCAGGGAAACACG.U.A..GG....................CUAUU.UGGCA................................................................................................
Ga0184578_101068/480-310          .UCAGGGAAACACG.U.A..GG....................CUAUU.UGGCA................................................................................................
Ga0247530_109035/489-319          .UCAGGGAAACACG.U.A..GG....................CUAUU.UGGCA................................................................................................
Ga0247513_128543/241-71           .UCAGGGAAACACG.U.A..GG....................CUAUU.UGGCA................................................................................................
Ga0184573_112336/323-153          .UCAGGGAAACACG.U.A..GG....................CUAUU.UGGCA................................................................................................
Ga0127502_10575752/515-449        .CCGGUGAAACAUC.U.A..C.....................CAU........................................................................................................
Ga0180107_1033011/786-679         .CCGGUGAAACAUC.C.A..UAU...................CUUGC...GA.................................................................................................
Ga0222728_1380294/134-238         .ACGGUGAAACAUC.U.A..UC....................UGUU..GGAU.................................................................................................
Ga0307484_107311/306-374          .UUGGGGAAACACC.C..........................CUAU.......................................................................................................
Ga0316046_107255/463-562          .CUGGUGAAACAUC.C.A..C.....................UUAU.......................................................................................................
Ga0184570_114412/209-338          .CAAGGGAAACAUU.A.G..AGA.....UUUGAA..CUGGAGAUUCGAGAUCA................................................................................................
Ga0242646_1000880/695-856         .CCAGGGAAACACU.C.U..AUG......CGUGG..C.....CAUUA...CA.................................................................................................
Ga0316031_123456/415-346          .CUGGGGAAACAUG.C.A..GC....................UAGG.......................................................................................................
Ga0157630_1203478/203-99          .CGGGGGAAACAUG.C.A..AGC..GUUACUU....CUGAUCAAUUU.GACCA................................................................................................
Ga0247541_102707/328-184          .UCAGGGAAACAUA.G.C..GUG..UGC..............CAA.A.AUCU.................................................................................................
Ga0115594_1174394/642-729         .GGAGGGAAACACC.G.U..ACU..U................AUUCU.AAAC.................................................................................................
Ga0115594_1187657/870-960         .GGAGGGAAACACC.G.U..AUA..AUU...............UAUC.UCUA.................................................................................................
Ga0242646_1047905/92-182          .UGAGGGAAACAUG.C.C..CC....................ACGCC......................................................................................................
Ga0247541_101130/558-391          .UCAGGGAAACACG.U.G..GA....................CUAUU.UGGCA................................................................................................
Ga0307921_1062541/180-338         .AGAGGGAAACACG.C.U..CUA....................CCU.GGU...................................................................................................
Ga0184572_105168/388-281          .UUGGGGAAACACA.U.U..GG....................CCUU..GCGCU................................................................................................
Ga0316036_127178/124-231          .UUGGGGAAACACA.U.U..GG....................UCUU..GCGCU................................................................................................
JGI12272J11983_1345183/364-258    .GGAGGGAAACACA.U.C..AGC..GU...............AGCG.......................................................................................................
Ga0247535_100068/719-828          .GGAGGGAAACACA.U.U..GCC..G................UAGC.......................................................................................................
Ga0222728_1000079/1192-1299       .GGAGGGAAACAUA.U.C..AAA..GU...............AACG.......................................................................................................
Ga0081246_1201717/153-259         .GGAGGGAAACACG.A.U..GUC..GU...............AGCG.......................................................................................................
Ga0222728_1216887/51-157          .GGAGGGAAACACA.C.U..GAA..GU...............AGCG.......................................................................................................
Ga0157569_1073490/184-258         .CCAGUGAAACAUC.C.U..C.....................AAC........................................................................................................
Ga0180047_1061805/548-622         .CCAGUGAAACAUC.C.U..C.....................AAA........................................................................................................
Ga0206353_11976089/292-380        .UCAGGGAAACACG.U.G..UUG..U................CCUAG.GACAU................................................................................................
Ga0247553_101094/709-629          .CUGGGGAAACACC.A.C........................UCCGU......................................................................................................
Ga0242641_1004288/32-112          .CUGGGGAAACACU.C.C..A.....................UACC.......................................................................................................
Ga0242641_1014474/336-257         .UCGGUGAAACAUG.C.U..CU....................AGUGA.U....................................................................................................
Ga0126318_10199518/109-204        .UGAGGGAAACACC.C.C..GCU..C................AGAU.......................................................................................................

                                  .<<<<.......<<.<.<..<<<..<<<<<<<<<<..<<<<<<.........................................................................................................
                                  .2222.......22.2.2..22..................................................................................................................................
                                  -●●RGGGAAACAY●-●-●--○○○---------------------○●●○○-------------------------------------------------------------------------------------------------------
```

```
Ga0307919_1109364/247-158      .UUAGGGAAACACA.G.A..AGA..ACGG..............GCU........................................................................................................
Ga0180107_1307618/257-174      .UGAGGGAAACAUG.C.C..UUC..UU................CUUGG......................................................................................................
Ga0179937_1126086/216-325      .GAAGGGAAACAUG.C.U..ACA..GUUGCAC...........AGAGUCUGCGCUGAGAC..........................................................................................
Ga0265744_134092/134-214       .CAAGGGAAACACU.C.U..UUU..AU................CUCCG......................................................................................................
Ga0307482_1015399/786-719      .CCAGGGAAACAUC.C.U.........................CUUG.......................................................................................................
Ga0265751_100477/874-675       .CCAGGGAAACAUA.C.U..GUC..UAU...............CACCUGCCUUGUACGUGUUCGGGUGGUUUCCCACCGUACUAGGCACAUGGAGCUCGCUAUCGUGAAGGGGUGAAACCCUUCCGUACGAGAUAACUA...........
Ga0265756_100433/485-286       .CCAGGGAAACAUA.C.U..GUC..UAU...............CACCUGCCUUGUACGUGUUCGGGUGGUUUCCCACCGUACUAGGCACAUGGAGCUCGCUAUCAUGAAGGGGUGAAACCCUUCUGUACGAGAUAACUA...........
Ga0265743_100639/33-191        .CCAGGGAAACAUA.C.U..GUC..UAUC..............ACCUGCCUUGUACGUGUUCGGGUGGUCUCCCACCGUACUAGGCACAUGGAGCU......................................................
Ga0184602_130623/1321-1100     .CCAGGGAAACACU.C.U..UAU..GA................CUCG.......................................................................................................
Ga0184597_126105/585-362       .CCAGGGAAACACU.C.U..UAU....................GUCUC.GGU..................................................................................................
Ga0187793_1322799/72-151       .CAGGGGAAACACA.G.G..G......................CAAGG.AGAAU................................................................................................
Ga0316037_128286/253-347       .UCAGGGAAACAUG.C.G..CGA..AGUC..............UGCU.......................................................................................................
Ga0265758_109576/83-157        .CCAGGGAAACACG.C...........................GAAA.......................................................................................................
Ga0265751_100127/176-103       .CCAGGGAAACAAC.C.A..C......................UCA........................................................................................................
Ga0265758_108618/113-22        .GCAGGGAAACAUG.A.C..GCU..GUC...............AUUAG.GAGAC................................................................................................
Ga0228708_1128185/228-344      .CAAGGGAAACACU.U.A..UGC..A...........CACCUAGAUA.......................................................................................................
Ga0256309_1199458/363-444      .AGAGGGAAACACA.U.U..GAC..UGAC..............UUGU.......................................................................................................
Ga0256302_1090383/76-157       .AGAGGGAAACACA.U.U..GAC..UGAC..............UUGU.......................................................................................................
Ga0255224_1021381/13-113       .AGAGGGAAACACA.U.U..GAC..UGACUUAAAC..C.GCACUUGU.......................................................................................................
Ga0255223_1087001/28-107       .AGAGGGAAACACA.U.U..GAC..UGA...............CUUG.......................................................................................................
Ga0127502_11371108/362-252     .AGAGGGAAACAUG.C.C..AGA....................UUCA.C.G..................................................................................................
Ga0222728_1000125/1315-1238    .CCAGGGAAACACU.G.U..U......................UUA........................................................................................................
Ga0242654_10000586/1273-1196   .CCAGGGAAACACU.G.U..U......................UAC........................................................................................................
Ga0180107_1218358/314-219      .CCAGGGAAACACU.C.A..AC.....................CAUA..GCGCA................................................................................................
Ga0307482_1020425/440-614      .CCAGGGAAACACG.U.G..AUG.....................CUAU.UG...................................................................................................
Ga0179951_1211246/478-633      .GCAGGGAAACAUA.A.U..UGU..GACAU.............UACAUUUCCUACAGCACUGUAAAACAUUGGGGCACGUGCCCCGCCGUCUACAGAUGC..................................................
Ga0179950_1197742/286-441      .GCAGGGAAACAUA.A.U..UGU..GACAU.............UACAUUUCCUACAGCACUGUAAGACAUUGGGGCACGUGCCCCGCCGUCUACAGAUGC..................................................
Ga0129331_1058147/189-99       .UGAGGGAAACACC.G.C..GCA..UAA................UAU..UCGAA................................................................................................
Ga0265756_102054/286-395       .GCAGGGAAACAUG.C.A..UCU....................CUAAC.G....................................................................................................
Ga0222728_1000128/4501-4432    .CCAGGGAAACACA.C.U.........................UAAA.......................................................................................................
Ga0265759_114856/370-262       .UCAGGGAAACAUG.A.G..AGA..GA....GAUA..UGGGCCGAGG.......................................................................................................
Ga0079039_1551950/237-315      .CAAGGGAAACAUG.C.U..A......................UGAG.......................................................................................................
Ga0179943_1170285/153-222      .UCAGGGAAACAUG.C.U..C......................AAAG.......................................................................................................
Ga0213850_1119088/23-236       .UGAGGGAAACAUG.C.U..ACA..UA................AGUUG.AGAAU................................................................................................
Ga0247533_103075/417-305       .CCAGGGAAACACA.U.A..UGC..UACGCC............AGUGC......................................................................................................
Ga0247541_102964/56-168        .CCAGGGAAACACA.U.A..UGC..UACGCC............AGUGC......................................................................................................
Ga0265755_105988/310-386       .CGAAGGAAACAUA.C.A..GU.....................ACG.G..AACA................................................................................................
Ga0157560_1026167/317-382      .UGAGGGAAACACG.G.C.........................GGA........................................................................................................
Ga0115595_1093158/7-137        .CCAGGGAAACAAC.A.A..AAG..AAGGAGCUAU..ACCUUCCUCAC.CG...................................................................................................
Ga0256352_1101982/155-277      .UCAGGGAAACACG.A.U..UAG..CGGCA.......AAUCCCACCCA......................................................................................................
Ga0256350_1100736/283-405      .UCAGGGAAACACG.A.U..UAU..CGGCAAAUCC........CACCCAGGAGU................................................................................................
Ga0213850_1360620/298-221      .CCAGGGAAACAAU.G.C..UUG..AA.................AUU..U.UA.................................................................................................
Ga0081247_1167831/113-35       .CCGGUGAAACAUA.C.G..ACU..C.................UCGUC.UCGAC................................................................................................
Ga0127503_10676578/136-67      .CCGGUGAAACAUG.C.G..CC.....................GCG........................................................................................................
Ga0138283_1149501/173-59       .CCGGUGAAACACG.C.G..GC...............GCGCGCAAACG......................................................................................................
Ga0265757_139522/177-70        .GGAGGGAAACACA.U.U..GG.....................CAUA..GCGAA................................................................................................
Ga0265750_1013321/389-496      .GGAGGGAAACACA.U.U..GGU.....................GUA..GCGA.................................................................................................
Ga0316029_116217/368-261       .GGAGGGAAACAUG.C.G..UUA..G..................UAAC.G....................................................................................................
Ga0316047_100016/2681-2574     .GGAGGGAAACAUG.C.G..UUA.....................GUA..GCG..................................................................................................
Ga0184569_112764/943-836       .GGAGGGAAACAUG.C.G..UUA.....................GUA..GCG..................................................................................................
Ga0242652_1131915/134-203      .GAGGUGAAACACA.C.C.........................GAAA.......................................................................................................
Ga0307482_1020622/600-404      .CCAGGGAAACACA.C.U..GUG..GG..............C.UAAGU.G....................................................................................................
Ga0265756_143657/133-55        .CAAGGGAAACACA.C.G..ACC..U.................UUUUC......................................................................................................
Ga0180107_1262812/3136-3066    .GAAGGGAAACACA.U.U..AC.....................AAU........................................................................................................
Ga0265756_118952/425-318       .CCGGCAAAACACC.A.U..GGU..GUGUA...............AGCAUA...................................................................................................
Ga0265757_113703/332-416       .CAAGGGAAACAUG.U.U..G.C..GAGAG...............AGU.UU...................................................................................................
Ga0256332_1243244/48-162       .UCGGUGAAACACC.C.C..AUA....CCGUUGUU..CGCAAGCCUAA.UCGGC................................................................................................
Ga0307919_1048575/242-90       .AGAGGGAAACACG.C.C..UUA....................CCUAG......................................................................................................
Ga0265754_1081367/214-290      .UAAGGGAAACACA.G.C..AGA..G.................GUAGA.G....................................................................................................
Ga0184597_114231/79-11         .CUGGGGAAACACU.C.U..A......................GAAA.......................................................................................................
Ga0184582_129853/106-35        .CUGGGGAAACACA.C.U..AU.....................GAAA.......................................................................................................
Ga0265744_136679/344-276       .CUGGGGAAACACU.C.U..A......................GAGA.......................................................................................................
Ga0326631_172366/284-207       .CCAGGGAAACAUG.C.C..G......................GCACU.G....................................................................................................
Ga0307921_1021783/160-261      .CCAGGGAAACAUG.C.G..UCA..GG................AGUUU.GGAGA................................................................................................
Ga0247554_113506/254-161       .CCGGGGAAACACA.U.G..AAC..GU................AGCG.......................................................................................................
Ga0179939_1229388/322-392      .ACAGGGAAACACU.G.C..A......................CCUCA......................................................................................................
Ga0184568_100511/100-195       .GCAGGGAAACAUC.G.C..AGC..G...................UA..GCG..................................................................................................
Ga0247525_114510/199-318       .CCAGGGAAACACA.C.C..GG.....................CAAG.......................................................................................................
Ga0222728_1008526/24-283       .CCAGGGAAACACC.A.G..GGU..UC................GAGAA.GAGCA................................................................................................
Ga0242654_10278372/461-362     .GAGGGGAAACACC.A.C..AAU..U...........AACUAUCAAA.......................................................................................................
Ga0184570_100371/69-155        .UGAGGGAAACACG.C.A..CGC..A.................GUUG.......................................................................................................
Ga0184597_120210/233-147       .UGAGGGAAACACG.C.A..CGC..A.................GUUG.......................................................................................................
Ga0213850_1220553/385-463      .GCAGGGAAACACU.C.C.........................GCGAA.G....................................................................................................
Ga0265745_1120044/54-129       .GGAGGGAAACACG.C.G...C.....................GCGAGGA....................................................................................................
Ga0213850_1278242/600-506      .UGGGGGAAACACG.C.C..GCG..GCCU.............AAGGC.......................................................................................................
Ga0213850_1524759/286-380      .UGGGGGAAACACG.C.C..GCG..GCCU.............AAGGC.......................................................................................................
Ga0242657_1009512/1576-1466    .GAAGGGAAACACA.C.C..AG.....................CCUAA.C.GAU................................................................................................
Ga0307482_1430195/259-189      .CUGGGGAAACACA.C.A..UU.....................AAUUC......................................................................................................
Ga0184590_129043/450-518       .GUGGGGAAACACG.U.C..UU.....................GCCU.......................................................................................................
Ga0184578_132646/9-94          .GCAGGGAAACACA.C.U..AAC...........C..CCCCCCCUCCC.A....................................................................................................
Ga0180107_1040049/1100-1010    .CAAGGGAAACACG.U.C..GGC..GG................UCGUC......................................................................................................
Ga0307484_103912/620-553       .UCAGGGAAACAUC.C.U.........................CUUGU......................................................................................................
Ga0307921_1037919/342-457      .UGGAGGAAACACA.C.C..AAA...CGUCCAGCU..U......UUCA.AAUU.................................................................................................
Ga0307920_113994/654-539       .UGGAGGAAACACA.C.C..AAA...CGUCCAGCU..U......UUCA.AAUU.................................................................................................
Ga0316040_172648/178-66        .UGGAGGAAACACA.C.C..AAA...CGUCCUGGC........UAAC.......................................................................................................
Ga0184577_110850/246-358       .UGGAGGAAACACA.C.C..AAA...CGUCCUGGC........UAAC.......................................................................................................
Ga0307920_113994/130-9         .UGGAGGAAACACA.C.C..AAA...CGUCCUAGC..U.....UUUCA.AAUUA................................................................................................
Ga0307919_1081825/191-74       .UGGAGGAAACACA.C.C..AAA...CGUCCUGAC..U.....AUAC.......................................................................................................
Ga0307921_1095896/178-61       .UGGAGGAAACACA.C.C..AAA...CGUCCUGGC..U.....AUAC.......................................................................................................

                               .<<<<.......<<.<.<..<<<..<<<<<<<<<<..<<<<<<...........................................................................................................
                               .2222.......22.2.2..22....................................................................................................................................
                               -●●RGGGAAACAY●-●-●--●○○--------------------●●●●○------------------------------------------------------------------------------------------------------
```

```
Ga0316031_113250/505-406        .UGAGGGAAACACG.U.G..AUA..G..A..GCG...AAUGACUGUGG.A...................................................................................................
Ga0265744_118928/137-218        .UGAGGGAAACACC.G.G..CC.....................CGUC.......................................................................................................
Ga0265749_106268/131-212        .UGAGGGAAACACC.G.G..CC.....................CGUC.......................................................................................................
Ga0157562_1160399/232-315       .CAAGGGAAACACA.G.U..AGU..U.................AUC........................................................................................................
Ga0153880_1231581/116-241       .UGAGGGAAACACA.G.U..GAG..UCGAAGUGGA..CGGGGCCAAAG......................................................................................................
Ga0153880_1191383/455-340       .AGAGGGAAACAUG.C.U..GUU..AAAGAUA........CAACCU........................................................................................................
Ga0138282_1242808/325-475       .GCAGGGAAACACG.C.C..GCU..C...........AACAUCUCAUC.G....................................................................................................
Ga0242654_10024002/836-915      .UGAGGGAAACACA.C.G..U......................AAAUC...ACA................................................................................................
Ga0242646_1103331/248-318       .CCAAGGAAACACA.U.U..CU.....................UACU.......................................................................................................
Ga0081243_1208438/380-456       .CCAGGGAAACAUA.C.U..A......................CCGGGUUGCCC................................................................................................
Ga0184600_147964/253-339        .UGAGGGAAACAUA.A.A..UGU..AC................AGAAA.U....................................................................................................
Ga0316036_107710/408-322        .UGAGGGAAACAUA.A.A..UGU..GC................AGAAA.U....................................................................................................
Ga0184601_135946/406-321        .UGAGGGAAACACA.A.A..UGU..GC................AGAAA.U....................................................................................................
Ga0184589_121067/502-587        .UGAGGGAAACACA.A.A..UGU..GC................AGAAA.U....................................................................................................
Ga0184598_138497/440-355        .UGAGGGAAACACA.A.A..UGU..GC................AGAAA.U....................................................................................................
Ga0184580_113725/262-335        .UGAGGGAAACACA.A.A..UGU..GC................AGAAA.U....................................................................................................
Ga0184584_115572/267-354        .CCAGGGAAACAUU.G.G..U......................UUUAU......................................................................................................
Ga0247539_100086/1763-1850      .CCAGGGAAACAUU.G.G..U......................UUUAU......................................................................................................
Ga0184579_105257/1735-1648      .CCAGGGAAACAUU.G.G..U......................UUUAU......................................................................................................
Ga0126318_10229384/609-688      .CCAGGGAAACACU.U.C..AC.....................GUA........................................................................................................
Ga0307482_1000291/1027-950      .CCAGGGAAACAUU.C.A..UGG..UUUUAC................A.U....................................................................................................
Ga0316031_119808/254-182        .CCGGUGAAACACU.C.C..AC.....................UUA........................................................................................................
Ga0307482_1023665/853-778       .CCAGGGAAACAUG.C.C..G......................ACUCC......................................................................................................
Ga0180106_1297567/444-515       .CCAGGGAAACACC.............................GUAAA......................................................................................................
Ga0307484_115660/213-145        .CCAGGGAAACAUC.C.U.........................UC.........................................................................................................
Ga0307484_161007/136-39         .GCAGGGAAACAUC.G.U..C......................UAUA.......................................................................................................
Ga0307484_177982/136-39         .GCAGGGAAACAUC.G.U..C......................UAUA.......................................................................................................
Ga0307482_1033688/516-422       .GCAGGGAAACAUC.G.U..C......................UAUA.......................................................................................................
Ga0265752_111922/418-343        .GCAGGGAAACAUG.C.A..C......................GAAA.......................................................................................................
Ga0179939_1199779/67-205        .CCAGGGAAACAUU.C.U..CAU..GU................CUCGACCUGCCCGCCUCUCAAAUCCAAGUCGCUUGUCACGACCCAGUGAGGGCAGAAGUCA..............................................
Ga0307482_1023050/176-255       .CCAGGGAAACACA.U.C..UGU..UAUCUU............AAU........................................................................................................
Ga0307482_1202605/470-395       .CCAGGGAAACACU.G.U..G......................UAUC.......................................................................................................
Ga0242657_1476020/160-244       .CCAGGGAAACAUA.C.U..AAA..UGUU..............AGUU.......................................................................................................
Ga0247523_120120/325-403        .CCAGGGAAACAUC.A.A..CC.....................UUCG.......................................................................................................
Ga0316035_179807/270-184        .CCAGGGAAACAUG.C.A..CCU..CC...............UGUCA..A....................................................................................................
Ga0210323_1050507/418-532       .CCAGGGAAACACG.C.G..A......................UGUAACAGCCUUUCAAGAACAAAGUGUUCUAAUAGGUCAAGCUG...............................................................
Ga0184571_112742/47-116         .CCAGGGAAACACG.C.U..........................UCA.......................................................................................................
Ga0247538_100770/928-997        .CCAGGGAAACACG.C...........................UUCA.......................................................................................................
Ga0157560_1032840/250-177       .GGAGGGAAACACC.U.U..U......................AAAGA.A....................................................................................................
Ga0247514_107497/1071-1140      .CCAGGGAAACACG.C.A..UC.....................UCCA.......................................................................................................
Ga0242646_1002014/11-270        .CCAGGGAAACACC.A.C..GGU..UC................GAGAAGAGCA.................................................................................................
Ga0222728_1008391/1281-1023     .CCAGGGAAACACC.A.C..GGU..UC................GAGAAGAGCA.................................................................................................
Ga0242646_1001395/255-509       .CCAGGGAAACACU.G.A..GAU....................CUCAA.UGAUC................................................................................................
Ga0307482_1000256/3341-3276     .ACAGGGAAACACU.A.U..C......................UUAC.......................................................................................................
Ga0242647_1006852/526-598       .CCAGGGAAACAAC.C.U.........................AUCU.......................................................................................................
Ga0184580_111164/678-749        .CCAGGGAAACAAC.A.U..G......................UUCG.......................................................................................................
Ga0184600_102725/60-135         .CCAGGGAAACAUC.A.C..A......................GCAA.......................................................................................................
Ga0184602_122974/190-265        .CCAGGGAAACAUC.A.C..A......................GCAA.......................................................................................................
Ga0256338_1343513/152-228       .CCAGGGAAACACA.C.C..AG.....................AUAAA.U....................................................................................................
Ga0255268_1432700/127-51        .CCAGGGAAACACA.C.C..AG.....................AUAAA.U....................................................................................................
Ga0157560_1147189/148-60        .UCAGGGAAACAAC.A.U..CGG..U.................CAAG.......................................................................................................
Ga0242654_10672177/244-165      .CCAGGGAAACACA.U.A..AC.....................CAAG.......................................................................................................
Ga0242654_10135580/213-336      .CUAGGGAAACACA.C.C.........................UAAA.......................................................................................................
Ga0265742_1000025/2819-2888     .CCAGGGAAACACU.G.C.........................GAAU.......................................................................................................
Ga0184599_154888/232-155        .CCAGGGAAACAUC.C.U..AU.....................AAC........................................................................................................
Ga0228707_1100139/344-194       .GUGGCGAAACACA.A.U..CAA..GC.....GUG..GUACCUUUUA.......................................................................................................
Ga0307919_1084008/308-378       .CGAGGGAAACAUG.C.C..CC.....................ACA........................................................................................................
Ga0307919_1039507/513-440       .CGAGGGAAACAUG.C.C..GC.....................AAUA.......................................................................................................
Ga0307921_1024041/406-334       .CGAGGGAAACAUG.C.C..GC.....................AAUA.......................................................................................................
Ga0242657_1015112/947-881       .CCAGGGAAACAUG.A.U..CUG.....................GUU.......................................................................................................
Ga0307482_1021658/484-563       .CCAGGGAAACAUC.U.G..CU.....................UUU........................................................................................................
Ga0307920_133961/233-140        .CAAGGGAAACAUA.C.G..ACG..A.................GAGGGAGAU..................................................................................................
Ga0307921_1019702/90-184        .CAAGGGAAACAUA.C.G..ACG..A.................GAGGGAGAU..................................................................................................
Ga0182092_1031527/199-109       .UGGGGGAAACAUA.C.C..ACA.....GAAGA..........AACAU.G....................................................................................................
Ga0184575_104772/255-338        .CCAGGGAAACAUG.C.A..CCU..U.................GCUUA......................................................................................................
Ga0179940_1033563/296-410       .UAAGGGAAACAUG.C.C..AGA.................AUGAAAAA......................................................................................................
Ga0184596_102602/271-200        .GCAGGGAAACACC.G.A..U......................CUU........................................................................................................
Ga0184596_125844/4802-4873      .GCAGGGAAACACC.G.A..U......................CUU........................................................................................................
Ga0153880_1058095/344-241       .CCAGGGAAACAUG.C.A..UCG..GCGCUC...........AAGUA.......................................................................................................
Ga0153880_1167367/224-121       .CCAGGGAAACAUG.C.A..UCG..GCGCUC...........AAGUA.......................................................................................................
Ga0179943_1178349/195-298       .CCAGGGAAACAUG.C.A..UCG..GCUUUC............ACGUA.G....................................................................................................
Ga0179944_1313268/129-232       .CCAGGGAAACAUG.C.A..UCG..GCGCUC............ACGUA.G....................................................................................................
Ga0213850_1259428/445-342       .CCAGGGAAACAUG.C.A..UCA..GAGUUC............ACGUA.G....................................................................................................
Ga0115595_1051440/308-419       .GAAGGGAAACAUA.C.G..GUG..AUG...............CCCAUUGGUGAAAAAG...........................................................................................
Ga0310106_100998/1608-1690      .GCAGGGAAACACU.G.U..UAU..UC................GAAA.......................................................................................................
Ga0307482_1017422/1387-1206     .CCAGGGAAACACG.U.G..AU.....................GCUAU.GGGUA................................................................................................
Ga0307482_1050048/176-95        .CCAGGGAAACAUC.A.U..C......................UGAC.......................................................................................................
Ga0179939_1078463/91-178        .CGAGGGAAACAUA.U.U..CCG..CACG..............AUCAG.AGAGA................................................................................................
Ga0247539_107237/153-229        .CCAGGGAAACACG.A.U.........................UUCG.......................................................................................................
Ga0127502_10189886/163-55       .CUAGGGAAACACC.A.C..GCG..ACAACCAA....GCACUUAUUC.......................................................................................................
Ga0265746_1085300/256-329       .GCAGGGAAACACC.U.C..G......................UCU........................................................................................................
Ga0242641_1002064/1203-1116     .CCAGGGAAACACA.U.C..C......................UCGUC......................................................................................................
Ga0242657_1090937/459-526       .GCAGGGAAACACC.G.A.........................UCAA.......................................................................................................
Ga0180107_1021361/468-373       .CCAGGGAAACACU.C.A..AG.....................CGUA..GCGAA................................................................................................
Ga0184599_136526/36-116         .GCAGGGAAACACU.A.U..AUC..AU................CUCGU.G....................................................................................................
Ga0316032_127868/210-130        .GCAGGGAAACACU.A.U..AUC..AU................CUCGU.G....................................................................................................
Ga0032354_1062690/2659-2586     .CCAGGGAAACACC.C...........................UUUU.......................................................................................................
Ga0242657_1027062/255-322       .CCAGGGAAACACU.C.G..U......................GUAA.......................................................................................................
Ga0265747_105373/294-105        .CCAGGGAAACACU.C.G..UAC....................UUGA.......................................................................................................

                                .<<<<.......<<.<.<..<<<..<<<<<<<<<<..<<<<<<...........................................................................................................
                                .2222.......22.2.2..22................................................................................................................................
                                -●●RGGGAAACAY●-●-●--○○○--------------------●●●○○-----------------------------------------------------------------------------------------------------
```

```
Ga0265745__1000512/1043-854     .CCAGGGAAACACU.C.G..UAC....................UUGA.......................................................................................................
Ga0265745__1001084/890-701      .CCAGGGAAACACU.C.G..UAC....................UUAA.......................................................................................................
Ga0310117__104105/1058-869      .CCAGGGAAACACU.U.G..CAC....................UUAA.......................................................................................................
Ga0265745__1000697/596-785      .CCAGGGAAACACU.C.G..UG.....................UUUA..AGGCA................................................................................................
Ga0247551__106718/112-179       .ACAGGGAAACACG.U.A..C......................AACA.......................................................................................................
Ga0184603__137775/245-174       .CCAGGGAAACAUU.G.G..AU.....................GAUUU......................................................................................................
Ga0242641__1105635/265-351      .UGAGGGAAACACA.C.C..CGA..GUC...............ACUCG......................................................................................................
Ga0242654__10795375/263-349     .UGAGGGAAACACA.C.C..UGA..GUC...............ACUCG......................................................................................................
Ga0157576__1168696/131-64       .UGAGGGAAACACA.U.A..C......................UAG........................................................................................................
Ga0255292__1094640/555-357      .UGAGGGAAACACA.U.A..C......................UACCAAGAGCACUAGGGCCAUCCCCCGGAUGUACCGGGAUUGGCCCGCCCCUUAUGGUGGAAAUGGCUUGCUCAGCCCCCAGGCAGGAACCGAGACAUGCGUCUUGU
Ga0256349__1073199/279-477      .UGAGGGAAACACA.U.A..C......................UACCAAGAGCACUAGGGCCAUCCCCCGGAUGGACCGGGAUUGGCCCGCCCCUUAUGGUGGAAAUGGCUUGCUCAGCCCCCAGGCAGGAACCGAGACAUGCGUCUUGU
Ga0256358__1096062/218-22       .UGAGGGAAACACA.U.A..C......................UACCAAGAGCACUAGGGCCAUCCCCCGGAUGGACCGGGAUUGGCCCGCCCCUUAUGGUGGAAAUGGCUUGCUCAGCCCCCAGGCGGAAACCGAGACAUGCGUCUUGU
Ga0256350__1079106/235-433      .UGAGGGAAACACA.U.A..C......................UACCAAGAGCACCAGGGCCAUCCCCCGGAUGGACCGGGAUUGGCCCGCCCCUUAUGGUGGAAAUGGCUUGCUCAGCCCCCAGGCAGGAACCGAGACAUGCGUCUUGU
Ga0256352__1080168/251-449      .UGAGGGAAACACA.U.A..C......................UACCAAGAGCACUAGGGCCAUCCCCCGGAUGGACCGGGAUUGGCCCGCCCCUUAUGGUGGAAAUGGCUUGCUCAGCCCCCAGGCAGGAACCGAGACAUGCGUCUUGU
Ga0255293__1084149/567-369      .UGAGGGAAACACA.U.A..C......................UACCAAGAGCACUAGGGCCAUCCCCCGGAUGGACCGGGAUUGGCCCGCCCCUUAUGGUGGAAAUGGCUUGCUCAGCCCCCAGGCAGGAACCGAGACAUGCGUCUUGU
Ga0256348__1071395/353-551      .UGAGGGAAACACA.U.A..C......................UACCAAGAGCACUAGGGCCAUCCCCCGGAUGGACCGGGAUUGGCCCGCCCCUUAUGGUGGAAAUGGCUUGCUCAGCCCCCAGGCAGGAACCGAGACAUGCGUCUUGU
Ga0265758__100178/821-753       .GCAGGGAAACAUG.G.U..CU.....................CACGC......................................................................................................
Ga0157560__1090855/105-186      .UGAGGGAAACACG.C.U..UAG..UUGC..............AUG........................................................................................................
Ga0005851__1006483/274-172      .UGAGGGAAACAUG.C.U..UU.....................UGC........................................................................................................
Ga0184603__107262/477-596       .CCAGGGAAACACA.C.C..GG.....................CAAG.......................................................................................................
Ga0184585__151549/495-376       .CCAGGGAAACACA.C.C..GG.....................CAAG.......................................................................................................
Ga0242647__1022053/339-428      AGCAGGGAAACAUC.A.G..UGA..AGCA..............CAAUU.GUA..................................................................................................
Ga0184599__128731/188-273       .CCAGGGAAACACC.C.G..AUC..AG................CAUCG......................................................................................................
Ga0310114__149639/68-153        .CCAGGGAAACACU.C.G..AUC..AG................CAUCG......................................................................................................
Ga0310119__131358/533-447       .CCAGGGAAACACU.C.G..AU..................CAGCAUC........................................................................................................
Ga0079074__1318576/412-571      .CCAGGGAAACACG.U.A..AGG..GG................GGGUGA.....................................................................................................
Ga0307484__102285/276-209       .ACAGGGAAACACC.G.U.........................AUA........................................................................................................
Ga0247534__109145/204-136       .GCAGGGAAACACC.G.U.........................AGUU.......................................................................................................
Ga0242657__1013457/154-227      .CCAGGGAAACAUA.G.A..C......................AAUA.......................................................................................................
Ga0242654__10000487/4104-4168   .CCAGGGAAACACG.C.U..........................UAA.......................................................................................................
Ga0242647__1038796/266-195      .GCAGGGAAACACU.A.C..C.......................UC........................................................................................................
Ga0115594__1156252/268-370      .CCAGGGAAACACC.G.C..AGA..GG................GGA.C.CAAA.................................................................................................
Ga0115594__1016490/326-410      .CCAGGGAAACACC.G.C..AGC..U.................GACCA......................................................................................................
Ga0115595__1007417/328-412      .CCAGGGAAACACC.G.C..AGC..U.................GACCA......................................................................................................
Ga0265742__1038020/174-90       .CCAGGGAAACACU.C.U..UGC..U..................ACUC.AAUU.................................................................................................
Ga0184568__114501/165-82        .CCAGGGAAACACU.C.U..UGU..UACU..............CAAUU......................................................................................................
Ga0265755__113253/366-449       .CCAGGGAAACACU.C.U..UGU..UAC..............UUAAU.......................................................................................................
Ga0184600__115185/364-447       .CCAGGGAAACACU.C.U..UGU..UACU..............CAAUU......................................................................................................
Ga0157593__1013623/299-215      .UGAGGGAAACAUA.C.U..CU.....................UACA.......................................................................................................
Ga0153880__1374410/101-33       .UCAGGGAAACACU.C.C.........................UACAU......................................................................................................
Ga0316038__101969/827-896       .GCAGGGAAACACG.U.U..CA.....................AUAUA......................................................................................................
Ga0316046__130251/204-135       .GCAGGGAAACACG.U.U..CA.....................AUAUA......................................................................................................
Ga0265758__104499/242-367       .CCAGGGAAACAUA.G.C..GUG..UGC...............CAGAA......................................................................................................
Ga0307484__108094/186-350       .UCAGGGAAACACC.U.C..CG....................CAUAUU..GGCA................................................................................................
Ga0307482__1009223/670-508      .UCAGGGAAACACC.U.C..UGC..C.................AUUG.......................................................................................................
Ga0242647__1067563/210-289      .CCAGGGAAACACA.U.C..C......................ACGUC......................................................................................................
Ga0242657__1207391/462-383      .CCAGGGAAACACA.U.U..AC.....................UUU........................................................................................................
Ga0265742__1001052/322-80       .CCAGGGAAACACA.U.C..GG.....................GUUUA.UGGCC................................................................................................
Ga0265750__1004028/1262-1504    .CCAGGGAAACACA.U.C..GG.....................GUAUA.UGGCC................................................................................................
Ga0310113__106868/972-730       .CCAGGGAAACACA.U.C..GG.....................GUAUA.UGGCC................................................................................................
Ga0265747__100653/495-253       .CCAGGGAAACACA.U.C..GG.....................GUUUA.UGGCC................................................................................................
Ga0265752__100504/930-688       .CCAGGGAAACACA.U.C..GG.....................GUAUA.UGGCC................................................................................................
Ga0265740__1001378/1084-842     .CCAGGGAAACACA.U.C..GG.....................GUUUA.UGGCC................................................................................................
Ga0265750__1009219/272-30       .CCAGGGAAACACA.U.C..GG.....................GUUUA.UGGCC................................................................................................
Ga0265757__104669/135-376       .CCAGGGAAACACA.U.C..GG.....................GUAUA.UGGCC................................................................................................
Ga0265745__1000618/793-552      .CCAGGGAAACACA.U.C..GG.....................GUAUA.UGGCC................................................................................................
Ga0265742__1088739/51-292       .CCAGGGAAACACA.U.C..GG.....................GUAUA.UGGCC................................................................................................
Ga0265747__100509/1277-1036     .CCAGGGAAACACA.U.C..GG.....................GUAUA.UGGCC................................................................................................
Ga0265752__100314/1466-1225     .CCAGGGAAACACA.U.C..GG.....................GUAUA.UGGCC................................................................................................
Ga0265743__104537/484-553       .CCAGGGAAACACC.U.C.........................ACCCA......................................................................................................
Ga0265744__160011/83-18         .CCAGGGAAACACG.U.A.........................GCAA.......................................................................................................
Ga0153880__1410217/288-397      .GAAGGGAAACAUG.C.U..CAC....................AAAU.......................................................................................................
Ga0115594__1054328/389-276      .GAAGGGAAACAUA.C.G..GUG..AUGCUC............AAUGGUUGAAA................................................................................................
Ga0316029__112968/271-337       .CAAGGGAAACAUA.C.C.........................AAGGA.G....................................................................................................
Ga0157560__1034839/242-321      .CAAGGGAAACAUA.C.U..UUG..A..................AGAA.GAGAG................................................................................................
Ga0157566__1117351/242-321      .CAAGGGAAACAUA.C.U..UUG..A..................AGAA.GAGA.................................................................................................
Ga0157593__1112297/86-12        .CAAGGGAAACAUA.C.U..GU.....................AUUUA.U....................................................................................................
Ga0157565__1020724/148-72       .CAAGGGAAACAUA.C.U..GUU.....................UUUC.A....................................................................................................
Ga0247519__104852/800-730       .CCAGGGAAACACA.A.C.........................GAAA.......................................................................................................
Ga0307480__1000549/913-1064     .ACAGGGAAACAUA.C.C..GGU..G.................AGAGGCCGAAUUGACCAAACGCAACGCGUUAGCGUCGGUUGGAGUCCUAAGG.......................................................
Ga0316031__132099/178-294       .CCAGGGAAACACC.G.C..A......................GAA........................................................................................................
Ga0247526__116548/279-354       .ACAGGGAAACACA.U.C..U......................AUAUC......................................................................................................
Ga0247519__113845/334-409       .GCAGGGAAACACA.U.C..UG.....................UAU........................................................................................................
Ga0247529__111070/303-228       .ACAGGGAAACACA.U.C..UG.....................UAU........................................................................................................
Ga0184602__101759/1045-970      .ACAGGGAAACACA.U.C..UG.....................UAU........................................................................................................
Ga0184600__139292/252-327       .GCAGGGAAACACA.U.C..UG.....................UAU........................................................................................................
Ga0184580__108231/296-221       .ACAGGGAAACACA.U.C..UG.....................UAU........................................................................................................
Ga0247534__105038/288-211       .ACAGGGAAACACA.U.C..UU.....................UUUAU......................................................................................................
Ga0247552__109074/256-333       .ACAGGGAAACACA.U.C..UU.....................UUUAU......................................................................................................
Ga0247542__100296/1022-1097     .ACAGGGAAACACA.U.C..U......................UUAUC......................................................................................................
Ga0247545__113720/232-157       .ACAGGGAAACACA.U.C..U......................AUAUC......................................................................................................
Ga0184597__110829/1090-1011     .ACAGGGAAACACC.A.U..C......................AGUA.......................................................................................................
Ga0184600__105920/81-160        .ACAGGGAAACACC.A.U..C......................AGUA.......................................................................................................
Ga0184578__129447/314-235       .ACAGGGAAACACC.A.U..C......................AGUA.......................................................................................................
Ga0184602__121948/324-245       .ACAGGGAAACACC.A.U..C......................AGUA.......................................................................................................
Ga0184603__125382/9-88          .ACAGGGAAACACC.A.U..C......................UGUA.......................................................................................................
Ga0247550__100138/208-129       .ACAGGGAAACACC.A.U..C......................AGUA.......................................................................................................
Ga0316046__122965/221-142       .ACAGGGAAACACC.A.U..C......................AGUA.......................................................................................................
Ga0316051__1003245/138-56       .ACAGGGAAACACC.A.U..C......................AGUAG.U...A................................................................................................

                                .<<<<.......<<.<.<..<<<..<<<<<<<<<<..<<<<<<...........................................................................................................
                                .2222.......22.2.2..22.
                                -••RGGGAAACAY•-•-•--∘∘∘--------------------•••∘∘------------------------------------------------------------------------------------------------------
```

```
Ga0316049_122425/209-288       .ACAGGGAAACACC.A.U..C......................AGUA.........................................................................................................
Ga0184588_113246/331-252       .ACAGGGAAACACC.A.U..C......................AGUA.........................................................................................................
Ga0184582_111448/113-237       .CCAGGGAAACACC.C.U..GGA..UGCGAGAACC........CAAGUCCUCCAACUGUAUUUUCAGGGAGUAGCAAU..........................................................................
Ga0184597_112736/543-466       .CCAGGGAAACACC.C.U..UG.....................AUGCA.UAGAA..................................................................................................
Ga0184577_100528/550-473       .CCAGGGAAACACC.C.U..UG.....................AUGCA.AAGAA..................................................................................................
Ga0127502_10397722/141-209     .GCAGGGAAACACC.C.A..U......................GUA..........................................................................................................
Ga0184593_119512/675-608       .ACAGGGAAACACC.C.A.........................AUAA.........................................................................................................
Ga0307482_1597388/86-18        .ACAGGGAAACACC.A.A..C......................AAC..........................................................................................................
Ga0307482_1001973/47-115       .ACAGGGAAACACC.A.A..C......................AAU..........................................................................................................
Ga0184595_132469/902-822       AGCAGGGAAACACA.U.A..CAU....................GUCAU.A......................................................................................................
Ga0079039_1443516/199-38       .CCAGGGAAACACU.C.U..GUG..AA................GAUAU.GG.....................................................................................................
Ga0265756_100130/1310-1239     .CCAGGGAAACACA.A.C.........................UAG..........................................................................................................
Ga0265747_125875/320-395       .UGAGGGAAACAUG.C.G..C......................GGG..........................................................................................................
Ga0180052_189627/240-346       .CGAGGGAAACACG.A.U..GG......................UGGCGA......................................................................................................
Ga0180044_1074338/112-33       .UGAGGGAAACACA.U.U..UGA..UAG...............UGCA.........................................................................................................
Ga0157559_1047848/128-41       .CCAGGGAAACACA.C.U..GU.....................AAAC.........................................................................................................
Ga0157562_1134247/69-160       .CCAGGGAAACAUG.C.U..GUG..C.................GCAA.........................................................................................................
Ga0247548_101188/825-888       .UGAGGGAAACACG.C.U.........................UGAC.........................................................................................................
Ga0228707_1093045/293-207      .CCAGGGAAACAUG.C.C..GCU..GUUCGAU...........UUGAA........................................................................................................
Ga0184597_119383/488-559       .CCAGGGAAACAUA.G.A..CU.....................GACAA........................................................................................................
Ga0184602_121040/35-106        .CCAGGGAAACAUA.G.A..CU.....................GACAA........................................................................................................
Ga0326631_107633/416-489       .CCAGGGAAACAUA.G.A..CU.....................GACAA........................................................................................................
Ga0316046_149266/91-162        .CCAGGGAAACAUG.G.A..CU.....................GACAA........................................................................................................
Ga0247538_100917/537-466       .CCAGGGAAACAUG.G.A..CU.....................GACAA........................................................................................................
Ga0247519_118271/334-405       .CCAGGGAAACAUA.G.A..CU.....................GACAA........................................................................................................
Ga0265747_100454/1395-1324     .CCAGGGAAACAUA.U.C..C......................AACA.........................................................................................................
Ga0316046_100502/370-442       .CCAGGGAAACAUA.U.C..C......................UAAUC........................................................................................................
Ga0247551_101484/617-543       .CCAGGGAAACAUA.U.C..C......................UAAUC........................................................................................................
Ga0213850_1280121/544-351      .CUGGGGAAACACG.U.G..UGU..GUGAGC..............................................................................................................................
Ga0265759_100017/4211-4288     .CCAGGGAAACACU.A.U..C......................GUAU.........................................................................................................
Ga0307482_1127544/505-398      .CCAGGGAAACACC.G.U..CAG..CUGUGU............AUUU.........................................................................................................
Ga0180107_1057591/210-107      .GCAGGGAAACAUC.A.C..U.......................AU..........................................................................................................
Ga0157569_1061476/44-119       .GUAGGGAAACAUG.U.U..AAG..GCAC..............UUUU.........................................................................................................
Ga0307484_100889/412-594       .CCAGGGAAACACG.U.G..AU.....................GCUAU.GGGUA..................................................................................................
Ga0310106_100022/563-489       .CCAGGGAAACACG.C.A..UAU..ACU...............AAAA.........................................................................................................
Ga0310115_100154/4483-4559     .CCAGGGAAACACG.C.A..UAU..ACU...............AAAAA.A......................................................................................................
Ga0180107_1262812/2765-2832    .CCAGGGAAACACA.C.A..UA.....................GCAA.........................................................................................................
Ga0184597_110025/865-793       .UCAGGGAAACACA.C.A..UA.....................GCAA.........................................................................................................
Ga0247550_100495/487-560       .UCAGGGAAACACA.C.A..UA.....................UGCAA........................................................................................................
Ga0184582_135749/62-135        .UCAGGGAAACACA.C.A..UA.....................UGCAA........................................................................................................
Ga0184603_122688/908-835       .UCAGGGAAACACA.C.A..UA.....................GGCAA........................................................................................................
Ga0247524_126530/125-198       .UCAGGGAAACACA.C.A..UU.....................GGCAA........................................................................................................
Ga0316030_115438/368-435       .CCAGGGAAACACU.G.G..U......................UGAU.........................................................................................................
Ga0307354_155926/276-116       .CCAGGGAAACACA.C.A..AGA..GA................AGUAA.GG.....................................................................................................
Ga0079074_1279626/114-274      .CCAGGGAAACACA.C.A..AGA..GA................AGUAU.GG.....................................................................................................
Ga0184602_132007/314-246       .CCAGGGAAACACA.U.G..AC.....................AAU..........................................................................................................
Ga0307921_1005950/791-877      .CAAGGGAAACACA.G.G..UAU..CA................UAAAA........................................................................................................
Ga0242654_10000487/4544-4475   .GGAGGGAAACACA.U.G..G......................UUUAU........................................................................................................
Ga0157569_1032488/120-205      .CCGGUGAAACACA.G.C..C......................AAAAC.AA.....................................................................................................
Ga0157593_1125711/213-128      .CCGGUGAAACACA.G.C..ACA.....................CAAG........................................................................................................
Ga0157565_1077069/156-67       .CCGGUGAAACACG.U.U..GUG..C.................GCAA.........................................................................................................
Ga0180047_1128081/151-64       .CCGGUGAAACACA.G.C..ACA.....................CAAG........................................................................................................
Ga0265756_135986/152-290       .CCAGGGAAACACA.C.C..ACC....AUUUAGGUUGCCUGCUAAUAU.UUCAA..................................................................................................
Ga0180045_119797/166-305       .CCAAGGAAACACU.C.U..AUG..GA................AGCGG........................................................................................................
Ga0115595_1089740/408-494      .GCAGGGAAACACA.C.U..AC....U.............CUGGUACA........................................................................................................
Ga0179955_1048341/123-51       .CCAGGGAAACACA.C.G.........................UAUUU.GGAAC..................................................................................................
Ga0138280_1140856/341-479      .CCAGGGAAACACG.C.C..GCU..C.................AGCG.........................................................................................................
Ga0180106_1302226/203-273      .CCAGGGAAACACA.G.C..C......................ACUCA........................................................................................................
Ga0310119_169621/253-177       .CCAGGGAAACACA.G.A..C......................AAUU.........................................................................................................
Ga0138277_1023917/182-18       .CCAGGGAAACACA.C.C..GCU..C.AACGAUCC..UAUCUAUAAU.........................................................................................................
Ga0138282_1020546/737-573      .CCAGGGAAACAUG.C.U..GCU..C.AACGAUCC..UAUCUAUAAU.........................................................................................................
Ga0179939_1059820/107-216      .CCAGGGAAACAUG.C.U..ACC..AC................GGAUGCAGAGCUUAGACCGCACGGGAA..................................................................................
Ga0179950_1098211/345-278      .CCAGGGAAACACA.C.C.........................AGGAG........................................................................................................
Ga0115594_1068468/127-56       .CCAGGGAAACACA.C.G.........................CAAAC.A......................................................................................................
Ga0228708_1103570/188-285      .CCAGGGAAACACG.C.G..GAC..UAGG............CUGACAAGA......................................................................................................
Ga0265751_160438/108-215       .CAAGGGAAACACG.C.G..CGC..C.................AGACU........................................................................................................
Ga0242647_1000066/2638-2719    .CCAGGGAAACACC.A.C..A.......................UA..........................................................................................................
Ga0242646_1001227/1243-1308    .CCAGGGAAACACC.A.A..AG.....................CUCA.........................................................................................................
Ga0182091_1104347/98-194       .CAAGGGAAACAUG.C.A..GCG..CGU..............UUGCUA........................................................................................................
Ga0115594_1178636/111-46       .CAAGGGAAACAUG.C.A..CU.....................UCCA.........................................................................................................
Ga0213850_1508869/496-402      .CAAGGGAAACAUG.C.A..CCA..GGU...............UGCU.........................................................................................................
Ga0307482_1152465/114-38       .CCAGGGAAACAUU.C.A..UA...............UGUUUAUUCA.........................................................................................................
Ga0180107_1031754/139-57       .CCAGGGAAACAUG.C.A..CUG..U.................UAUUU........................................................................................................
Ga0316037_121702/176-240       .CCAGGGAAACAUC.C.A..C......................UACA.........................................................................................................
Ga0265759_107962/103-39        .CCAGGGAAACAUC.C.A..C......................UACA.........................................................................................................
Ga0242649_1051170/368-302      .CCAGGGAAACACA.C.U..CU.....................CUUAU........................................................................................................
Ga0307482_1064439/84-153       .CCAGGGAAACACA.C.U..UUG..A.................CUUG.........................................................................................................
Ga0115594_1006122/120-29       .CCAGGGAAACACC.G.U..CAG..CU................GACCA........................................................................................................
Ga0115595_1180708/226-317      .CCAGGGAAACACC.G.U..CAG..CU................GACCA........................................................................................................
Ga0265746_1064626/389-458      .CCAGGGAAACACG.C.U..C......................CAC..........................................................................................................
Ga0265759_108120/277-351       .CCAGGGAAACACC.G.C..U......................UAAUU........................................................................................................
Ga0307482_1000450/2015-2077    .CCAGGGAAACACC.G.U..U......................ACA..........................................................................................................
Ga0247527_115808/280-440       .CCAGGGAAACACC.G.U..AGC.......CUGGC..U.....AUUAC........................................................................................................
Ga0184588_120624/266-426       .CCAGGGAAACACU.G.U..AGC.......CUGGC..U.....AUUAC........................................................................................................
Ga0184579_106194/996-1156      .CCAGGGAAACACU.G.U..AGC.......CUGGC..U.....AUUAC........................................................................................................
Ga0265758_100254/356-516       .CCAGGGAAACACC.G.U..AGC.......CUGGC..U.....AUUAC........................................................................................................
Ga0316035_107544/354-514       .CCAGGGAAACACC.G.U..AGC.......CUGGC..U.....AUUAC........................................................................................................
Ga0184596_109261/979-1139      .CCAGGGAAACACC.G.U..AGC.......CCGGC..U.....AUUAC........................................................................................................
Ga0184600_132066/568-408       .CCAGGGAAACACC.G.U..AGC.......CUGGC..U.....AUUAC........................................................................................................

                               .<<<<.......<<.<.<..<<<..<<<<<<<<<<..<<<<<<.............................................................................................................
                               .2222.......22.2.2..22..................................................................................................................................
                               -●●RGGGAAACAY●-●-●--○○○--------------------●●●○○--------------------------------------------------------------------------------------------------------
```

```
Ga0184597_106861/196-36        .CCAGGGAAACACC.G.U..AGC.......CUGGC..U.....AUUAC......................................................................................................
Ga0316031_108320/216-376       .CCAGGGAAACACC.G.U..AGC.......CUGGC..U.....AUUAC......................................................................................................
Ga0184589_110648/1041-881      .CCAGGGAAACACC.G.U..AGC.......CCGGC..U.....AUCAC......................................................................................................
Ga0184580_124121/239-79        .CCAGGGAAACACC.G.U..AGC.......CCGGC..U.....AUCAC......................................................................................................
Ga0184598_101324/530-690       .CCAGGGAAACACC.G.U..AGC.......CCGGC..U.....AUCAC......................................................................................................
Ga0247547_102464/189-29        .CCAGGGAAACACU.C.C..AUC......AUGGGU..U.....AUUAC......................................................................................................
Ga0247551_100828/546-387       .CCAGGGAAACACU.U.C..AUC.......UGGGC..U.....AUCAC......................................................................................................
Ga0247536_101914/360-201       .CCAGGGAAACACU.U.C..AUC.......AGGGC..U.....AUUAC......................................................................................................
Ga0247538_100480/788-947       .CCAGGGAAACACU.U.C..AUC.......AGGGC..U.....AUUAC......................................................................................................
Ga0247550_100850/456-297       .CCAGGGAAACACU.U.C..AUC.......AGGGC..U.....AUCAC......................................................................................................
Ga0247553_110698/526-367       .CCAGGGAAACACU.U.C..AUC.......AGGGC..U.....AUCAC......................................................................................................
Ga0184589_112135/40-200        .CCAGGGAAACACU.C.U..GUC.....UUGGGUU........AUUAC......................................................................................................
Ga0247526_110221/603-443       .CCAGGGAAACACU.C.C..AUC..AU....GGGU..U.....AUUAC......................................................................................................
Ga0247541_103784/123-283       .CCAGGGAAACACU.C.U..GU......CUUGGGU..U.....AUUAC......................................................................................................
Ga0247545_112960/183-343       .CCAGGGAAACACU.C.U..GU......CUUGGGU..U.....AUUAC......................................................................................................
Ga0184586_133850/368-528       .CCAGGGAAACACU.C.C..AUC......UUGGGC..U.....AUUAC......................................................................................................
Ga0247552_101742/612-452       .CCAGGGAAACACU.C.C..AU......CUUGGGU..U.....AUUAU......................................................................................................
Ga0247545_104341/406-246       .CCAGGGAAACACU.C.C..AU......CUUGGGU..U.....AUUAC......................................................................................................
Ga0184602_114416/11-171        .CCAGGGAAACACU.C.C..AU......CUUGGGU..U.....AUUAC......................................................................................................
Ga0316038_111670/11-171        .CCAGGGAAACACU.C.C..AU......CUUGGGC..U.....AUUAC......................................................................................................
Ga0247529_134913/224-64        .CCAGGGAAACACU.C.C..AU......CUUGGAU..U.....AUUAC......................................................................................................
Ga0184603_143085/253-93        .CCAGGGAAACACU.C.C..AU......CUUGGGC..U.....AUUAC......................................................................................................
Ga0316032_107575/215-55        .CCAGGGAAACACU.C.C..AU......CUUGGGU..U.....AUUAC......................................................................................................
Ga0247519_110701/607-447       .CCAGGGAAACACU.C.C..AU......CUUGGGU..U.....AUUAC......................................................................................................
Ga0247524_111427/50-210        .CCAGGGAAACACU.C.C..AU......CUUGGGC..U.....AUUAC......................................................................................................
Ga0184574_121290/269-109       .CCAGGGAAACACU.C.C..AU......CUUGGGU..U.....AUUAC......................................................................................................
Ga0184595_111222/3-163         .CCAGGGAAACACU.C.C..AU......CUUGGGU..U.....AUUAC......................................................................................................
Ga0184600_123882/188-28        .CCAGGGAAACACU.C.C..AU......CUUGGGU..U.....AUUAC......................................................................................................
Ga0326631_101563/544-705       .CCAGGGAAACACU.C.C..AUC......CUGGGU..U.....AUUAC......................................................................................................
Ga0184573_110190/115-276       .CCAGGGAAACACU.C.C..AU......CUUGGAU..U.....AUUAC......................................................................................................
Ga0184596_111007/525-686       .CCAGGGAAACACU.C.C..AUC......CUGGGU..U.....AUUAU......................................................................................................
Ga0316033_144939/167-328       .CCAGGGAAACACU.C.C..AU......CUUGGGU..U.....AUUAC......................................................................................................
Ga0247526_117235/152-313       .CCAGGGAAACACU.C.U..GU......CUUGGGU..U.....AUUAC......................................................................................................
Ga0247551_100195/490-329       .CCAGGGAAACACU.C.U..GU......CUUGGGU..U.....AUUAC......................................................................................................
Ga0247538_100424/120-281       .CCAGGGAAACACU.C.C..AUC.....UAGGGUU........UUUUA.U....................................................................................................
Ga0247550_114736/167-7         .CCAGGGAAACACU.C.C..AUC.....UAGGGUU........AUUA.......................................................................................................
Ga0247529_103231/270-430       .CCAGGGAAACACU.C.C..AUC..GU....GGGU..A.....UUA........................................................................................................
Ga0184603_111918/241-82        .CCAGGGAAACACU.C.C..AUC......UGGGUU..A.....UUA........................................................................................................
Ga0184603_126764/80-240        .CCAGGGAAACACU.C.C..AUC.....UUGGGUU..A.....UUA........................................................................................................
Ga0247519_107918/217-58        .CCAGGGAAACACU.C.C..AUC......UGGGUU..A.....UUA........................................................................................................
Ga0184580_114961/306-146       .CCAGGGAAACACU.C.C..AUC.....UUGGGUU..A.....UUA........................................................................................................
Ga0213850_1042834/351-428      .UCAGGGAAACAUU.C.U..CG......................CA.G.GAGU.................................................................................................
Ga0265756_103656/97-163        .UCAGGGAAACACC.A.C.........................UCCUC......................................................................................................
Ga0265756_101189/896-833       .UCAGGGAAACACC.C.U..C.......................GU........................................................................................................
Ga0180107_1033011/414-478      .CCAGGGAAACACC.G.U..U......................UAC........................................................................................................
Ga0115594_1157594/155-254      .CCAGGGAAACACC.G.U..CAG..C.................CGACC.A....................................................................................................
Ga0115594_1166236/119-19       .CCAGGGAAACACC.G.U..CAG..CU................GACCA......................................................................................................
Ga0115594_1007070/120-30       .CCAGGGAAACACC.G.U..GAG..CU................GACCA......................................................................................................
Ga0115595_1181384/176-86       .CCAGGGAAACACC.G.U..GAG..CU................GACCA......................................................................................................
Ga0115594_1211214/299-389      .CCAGGGAAACACC.G.U..UGG..CU................GACCA......................................................................................................
Ga0115594_1182529/183-84       .CCAGGGAAACACC.G.U..CAG..CU................GACCA......................................................................................................
Ga0256352_1182139/180-81       .CCAGGGAAACACC.G.U..CAG..CU................GACCA......................................................................................................
Ga0179944_1005311/261-382      .CGAGGGAAACACG.G.C...UA..AGCAUCCUAC..UCC...UUUC.......................................................................................................
Ga0213850_1508120/373-299      .CAUGGGAAACACG.A.U..CU.....................UAACU......................................................................................................
Ga0307484_162891/325-258       .CCAGGGAAACAUG.U.C..UC.....................AUAAU.U....................................................................................................
Ga0184596_111635/86-148        .CAAGGGAAACACG.U.C.........................UUA........................................................................................................
Ga0316051_1005111/447-509      .CAAGGGAAACACG.U.C.........................UUU........................................................................................................
Ga0265753_1379003/85-148       .CAAGGGAAACACG.U.C.........................UUAU.......................................................................................................
Ga0184603_116647/206-269       .CAAGGGAAACACG.U.C.........................UUAC.......................................................................................................
Ga0184587_132427/565-485       .CCAGGGAAACACA.C.C..CCC....................CAGAA......................................................................................................
Ga0184599_110049/275-349       .CCAGGGAAACACA.C.C.........................CUUGC......................................................................................................
Ga0184603_148109/996-920       .CCAGGGAAACACA.C.C..C......................CUUUC......................................................................................................
Ga0310114_133004/56-132        .CCAGGGAAACACA.C.C..C......................CUCUC......................................................................................................
Ga0310119_113172/381-306       .CCAGGGAAACACA.C.C..C......................UCUU.......................................................................................................
Ga0265747_119090/8-84          .CAAGGGAAACAUG.C.C.........................AUAC.......................................................................................................
Ga0247551_122551/29-115        .CCAGGGAAACACU.U.C..A.......................UA........................................................................................................
Ga0316048_107802/239-324       .CCAGGGAAACACU.U.C..A.......................UA........................................................................................................
Ga0242657_1208314/452-382      .CCAGGGAAACACU.C.C..U......................AUU........................................................................................................
Ga0184596_129213/83-168        .ACGGGGAAACACU.A.G..CA...............ACACACACUUA......................................................................................................
Ga0184603_146917/441-510       .ACGGGGAAACACU.A.G..C......................UGU........................................................................................................
Ga0247550_102601/160-89        .ACGGGGAAACACU.A.G..C......................AAAC.......................................................................................................
Ga0184602_110024/263-177       .ACGGGGAAACACU.A.G..GC...............AGCUUACAUAG......................................................................................................
Ga0184597_116115/700-787       .ACGGGGAAACACU.A.G..CG............A..AUUUACAUAC.......................................................................................................
Ga0184582_128596/700-786       .ACGGGGAAACACU.A.G..CU............A..AUUUACAAA........................................................................................................
Ga0184597_102019/330-402       .ACGGGGAAACACU.A.G..C......................UGU........................................................................................................
Ga0242649_1000320/700-631      .CCAGGGAAACACG.G.A.........................UUUUG......................................................................................................
Ga0179943_1102267/215-90       .CCAGGGAAACACG.A.G..UCU..C..AGCAUCC..UACUCCUUUC.......................................................................................................
Ga0179944_1191552/284-411      .CCAGGGAAACACG.C.A..GUC...UCAGCAUCC..UACUCCUUUC.......................................................................................................
Ga0179943_1150468/216-89       .CCAGGGAAACACG.U.A..GUC...UCAGCAUCC..UGCC..UUUC.......................................................................................................
Ga0179944_1204933/216-89       .CCAGGGAAACACG.U.A..GUC...UCAGCAUCC..UGCC..UUUC.......................................................................................................
Ga0265758_104558/436-365       .CCAGGGAAACACA.C.U..A......................AUA........................................................................................................
Ga0180107_1040315/471-547      .CCAGGGAAACACA.A.U..GUU..C.................UUU........................................................................................................
Ga0180106_1332058/4386-4449    .UCAGGGAAACACC.A.U..C......................CAGU.......................................................................................................
Ga0307484_104189/90-171        .GCAGGGAAACACA.C.C..U......................CACA.......................................................................................................
Ga0265752_100023/1212-1290     .CCAGGGAAACACA.U.A..AUA..AC................AGUAU.U....................................................................................................
Ga0157593_1101573/285-478      .CCAGGGAAACACA.U.G..UAU..AAAGGCUCUU........AGCCGCCUGCCCCGUGAGGGGGGGCUUGCCCAACACUCUUUAGUGAGAAAAGCGCGAA.................................................
Ga0307482_1008742/1212-1288    .CCAGGGAAACACU.A.U..C......................AUA........................................................................................................
Ga0247551_104770/284-362       .GCAGGGAAACACC.A.C..UCA..GU................AAAAC.AA...................................................................................................
Ga0184593_112903/290-212       .GCAGGGAAACACC.A.C..UCA..UU................AAAAC.AA...................................................................................................

                               .<<<<.......<<.<.<..<<<..<<<<<<<<<<..<<<<<<...........................................................................................................
                               .2222.......22.2.2..22................................................................................................................................
                               -••RGGGAAACAY•-•-•--∘∘∘--------------------••••∘∘---------------------------------------------------------------------------------------------------------
```

```
Ga0307482_1128390/271-339          .GCAGGGAAACACC.A.C.........................ACCAU.A....................................................................................................
Ga0184580_126177/800-732           .GCAGGGAAACAUC.C.C..A......................UAU........................................................................................................
Ga0242646_1115622/191-109          .GCAGGGAAACAUC.G.C..A......................CCUAU......................................................................................................
JGI12272J11983_1319142/230-159     .GCAGGGAAACAUC.A.U..AC.....................GUU........................................................................................................
Ga0184596_105062/163-229           .GCAGGGAAACACC.U.C.........................UAC........................................................................................................
Ga0307482_1011891/77-10            .GCAGGGAAACACC.U.C.........................AUAC.......................................................................................................
Ga0184588_125064/416-488           .GCAGGGAAACAAC.A.C..U......................ACU........................................................................................................
Ga0184577_107529/254-327           .GCAGGGAAACAAC.A.C..U......................ACU........................................................................................................
Ga0180106_1182034/403-337          .GCAGGGAAACACC.A.C.........................UAUC.......................................................................................................
Ga0180107_1241189/1554-1620        .GCAGGGAAACACC.A.C.........................UAUC.......................................................................................................
Ga0222728_1000110/4754-4821        .GCAGGGAAACACC.A.C.........................UCUC.......................................................................................................
Ga0307484_100154/562-629           .GCAGGGAAACACC.A.C.........................GUGA.......................................................................................................
Ga0180107_1285439/94-155           .ACAGGGAAACAUA.C.C.........................UAAC.......................................................................................................
Ga0184580_116530/249-177           .ACAGGGAAACAUC.G.C..U......................UAUG.......................................................................................................
Ga0247526_117664/434-499           .CGAGGGAAACACA.C.U.........................AUAU.......................................................................................................
Ga0184580_120219/79-4              .CCAGGGAAACACA.U.U..UA.....................UGAUA......................................................................................................
Ga0247539_100482/682-612           .CGAGGGAAACAUG.C.A..UA.....................CGU........................................................................................................
Ga0247516_124175/202-272           .CGAGGGAAACAUG.C.A..UA.....................CGU........................................................................................................
Ga0184580_121247/966-1036          .CGAGGGAAACAUG.C.A..UA.....................CGA........................................................................................................
Ga0265743_100411/1096-1162         .CGAGGGAAACAUG.C...........................GUUA.......................................................................................................
Ga0242657_1070636/108-176          .CCAGGGAAACACG.U.U..C......................AAUGC......................................................................................................
Ga0242657_1122750/95-162           .CCAGGGAAACACA.U.U..C......................GAAA.......................................................................................................
Ga0307482_1405960/253-322          .CCAGGGAAACACA.A.U..C......................AUAU.......................................................................................................
Ga0316049_154968/227-319           .CCAGGGAAACAUA.C.C.........................AUGUA......................................................................................................
Ga0127502_10852756/278-345         .CCAGGGAAACACA.A.U..C......................AUU........................................................................................................
Ga0247534_100003/3657-3582         .CCAGGGAAACACA.C.U..AUG..A.................UUAU.......................................................................................................
Ga0180106_1204848/284-221          .CCAGGGAAACACG.A.A.........................AG.........................................................................................................
Ga0184585_128240/467-390           .CCAGGGAAACACG.A.G..A......................UUUG.......................................................................................................
Ga0180106_1180558/1549-1486        .CCAGGGAAACAUG.A.G.........................AAGU.......................................................................................................
Ga0265755_100134/298-372           .CCAGGGAAACACA.U.C..UC.....................AUUG.......................................................................................................
Ga0247534_100772/309-234           .CCAGGGAAACACA.U.C..U......................AUCU.......................................................................................................
Ga0307482_1202063/402-466          .CCAGGGAAACACG.U.C..C......................AAC........................................................................................................
Ga0247514_114593/280-211           .CCAGGGAAACACA.U.U..C......................UUAAU......................................................................................................
Ga0310101_157764/124-55            .CCAGGGAAACACA.C.U..C......................UUAAC......................................................................................................
Ga0265745_1000136/129-60           .CCAGGGAAACACA.C.U..C......................UUAAC......................................................................................................
Ga0265745_1013560/201-270          .CCAGGGAAACACA.C.U..C......................UUAAC......................................................................................................
Ga0307482_1779657/200-270          .CCAGGGAAACACA.U.C..UA.....................GAAA.......................................................................................................
Ga0307482_1044183/658-591          .CCAGGGAAACACA.U.C..UA.....................GAAA.......................................................................................................
Ga0265758_100017/4376-4309         .UCAGGGAAACACA.G.G.........................UGACA.UAGUC................................................................................................
Ga0242654_10617983/312-237         .CCAGGGAAACACA.C.U..ACU....................UCUU.U.....................................................................................................
Ga0222728_1054736/119-193          .CCAGGGAAACAUG.G.A.........................GAAA.......................................................................................................
Ga0184583_106441/296-227           .CCAGGGAAACACA.U.C..C......................GUAA.......................................................................................................
Ga0247520_104849/891-821           .CCAGGGAAACACA.A.U..C......................UUUAU......................................................................................................
Ga0247518_137824/301-231           .CCAGGGAAACACA.A.U..C......................UUUAU......................................................................................................
Ga0247531_104861/1082-1012         .CCAGGGAAACACA.A.U..C......................UUUAU......................................................................................................
Ga0265746_1040401/190-123          .CCAGGGAAACAUA.G.C..AA.....................UCUU.......................................................................................................
Ga0184603_139760/199-128           .CCAGGGAAACACA.U.G..AU.....................AAACA......................................................................................................
Ga0184603_138085/381-311           .CCAGGGAAACACA.U.G..AU.....................CACG.......................................................................................................
Ga0265747_102820/448-376           .CCAGGGAAACACA.U.G..AU.....................CACG.......................................................................................................
Ga0247554_105388/388-316           .CCAGGGAAACACA.U.G..AU......................UACG......................................................................................................
Ga0316037_123461/289-368           .CCAGGGAAACACA.U.G..AU.....................AACA.......................................................................................................
Ga0184597_121517/262-183           .CCAGGGAAACACA.A.U..AC.....................CUUU.......................................................................................................
Ga0247551_100118/653-723           .CCAGGGAAACACA.A.U..AC.....................CUUU.......................................................................................................
Ga0265750_1365815/134-55           .CCAGGGAAACACA.A.U..AC.....................CUUU.......................................................................................................
Ga0184589_120335/11-90             .CCAGGGAAACACA.A.U..AC.....................CUUU.......................................................................................................
Ga0247521_124040/388-309           .CCAGGGAAACACA.A.U..AC.....................CUUU.......................................................................................................
Ga0247518_108423/758-837           .CCAGGGAAACACA.A.U..AC.....................CUUU.......................................................................................................
Ga0247531_104608/1256-1177         .CCAGGGAAACACA.A.U..AC.....................CUUU.......................................................................................................
Ga0247543_100863/642-721           .CCAGGGAAACACG.A.U..AC.....................CUUU.......................................................................................................
Ga0307482_1026216/884-813          .CCAGGGAAACACA.U.C.........................UUCG.......................................................................................................
Ga0307482_1636410/270-341          .CCAGUGAAACACA.U.C.........................UUCG.......................................................................................................
Ga0310119_128165/432-365           .CCAGGGAAACACA.A.C..A......................CUA........................................................................................................
Ga0307482_1016877/168-97           .CCAGGGAAACACA.U.C.........................GAAA.......................................................................................................
Ga0126318_10746885/3334-3265       .CCAGGGAAACACA.U.C..UU.....................AAAC.......................................................................................................
Ga0206353_11117566/971-1032        .CCAGGGAAACACA.G.C.........................GUAU.......................................................................................................
Ga0180107_1106188/424-495          .CCAGGGAAACACA.U.C.........................GAAA.......................................................................................................
Ga0307482_1000354/2063-1987        .CCAGGGAAACACU.U.C..A......................GUUA.......................................................................................................
Ga0180107_1132096/355-287          .CCAGGGAAACACU.A.U..A......................CUUG.......................................................................................................
Ga0180108_1238667/303-371          .CCAGGGAAACACU.A.U..A......................CUCG.......................................................................................................
Ga0265751_100981/715-788           .CCAGGGAAACACU.G.U..AU.....................CUUUG......................................................................................................
Ga0265756_100898/263-336           .CCAGGGAAACACU.G.U..AU.....................CUUUG......................................................................................................
Ga0307484_102094/153-80            .CCAGGGAAACACG.A.U..CU.....................UAUC.......................................................................................................
Ga0307482_1023270/273-197          .CCAGGGAAACACG.A.U..C......................UCU........................................................................................................
Ga0184596_102741/625-701           .CCAGGGAAACACG.A.U..C......................UCU........................................................................................................
Ga0316033_103637/453-391           .CCAGGGAAACACG.A.U..C......................UCA........................................................................................................
Ga0180107_1069127/170-106          .GCAGGGAAACACA.U.C.........................AUUU.......................................................................................................
Ga0247535_105396/277-348           .GCAGGGAAACACA.G.U.........................UUCU.......................................................................................................
Ga0184576_110410/277-348           .GCAGGGAAACACA.G.U.........................UUCU.......................................................................................................
Ga0307482_1219330/19-95            .CCAGGGAAACACU.G.U.........................UUUAU......................................................................................................
Ga0247534_116177/42-105            .CCAGGGAAACUCA.A.C.........................AUUU.......................................................................................................
Ga0316033_123149/165-228           .CCAGGGAAACACA.A.C.........................AUUU.......................................................................................................
Ga0265751_100560/642-572           .CCAGGGAAACACC.A.U..GA.....................CUUCG......................................................................................................
Ga0307482_1011142/698-628          .CCAGGGAAACAUU.C.C..UU.....................AAAAA......................................................................................................
Ga0307482_1465843/259-190          .CCAGGGAAACAUU.C.C.........................AAUAA.AA...................................................................................................
Ga0184593_115334/333-240           .CCAGGGAAACAUA.C.C..UU.....................UGU........................................................................................................
Ga0265751_100089/3005-3069         .CCAGGGAAACAUC.A.C..AC.....................AUU........................................................................................................
Ga0307482_1000325/380-315          .CCAGGGAAACAUC.A.C..U......................UAU........................................................................................................
Ga0222729_1014021/463-392          .CCAGGGAAACACA.U.U..GUC..A.................UCACU.U....................................................................................................
Ga0307482_1033651/424-494          .GCAGGGAAACACU.G.U..AG.....................AUU........................................................................................................

                                   .<<<<.......<<.<.<..<<<..<<<<<<<<<<..<<<<<<.........................................................................................................
                                   .2222.......22.2.2..22.............................................................................................................................
                                     RGGGAAACAY
```

```
Ga0307482_1015330/1021-1093    .CCAGGGAAACACA.C.C.........................AAA..........................................................................................................
Ga0184570_111551/35-97         .UCAGGGAAACACG.C.A.........................UC...........................................................................................................
Ga0184576_117861/362-299       .UCAGGGAAACACG.C.U.........................AAA..........................................................................................................
Ga0307482_1589279/351-280      .GCAGGGAAACACU.C.A..A......................CAAU.........................................................................................................
Ga0184586_104419/174-104       .GCAGGGAAACACU.C.A..G......................CAAU.........................................................................................................
Ga0222728_1148620/185-250      .GCAGGGAAACACU.C.A.........................UC...........................................................................................................
Ga0316049_103217/889-823       .GCAGGGAAACACU.C.U.........................CCA..........................................................................................................
Ga0316034_104024/504-439       .GCAGGGAAACACU.C.U.........................CCA..........................................................................................................
Ga0184595_117496/296-230       .GCAGGGAAACACU.C.U..........................CUA.........................................................................................................
Ga0184597_109849/897-963       .GCAGGGAAACACU.C.U.........................CCA..........................................................................................................
Ga0184588_134146/1219-1285     .GCAGGGAAACACU.C.U.........................CCA..........................................................................................................
Ga0184584_115247/186-121       .GCAGGGAAACACU.C.U.........................CCA..........................................................................................................
Ga0184596_126546/83-162        .CCAGGGAAACACC.A.C..U......................GUU..........................................................................................................
Ga0184570_116436/278-198       .CCAGGGAAACACC.A.C..U......................GUU..........................................................................................................
Ga0184597_111165/262-182       .CCAGGGAAACACC.A.C..U......................GUU..........................................................................................................
Ga0316037_169124/279-199       .CCAGGGAAACACC.A.C..U......................GUU..........................................................................................................
Ga0247551_103383/322-242       .CCAGGGAAACACC.A.C..U......................GUU..........................................................................................................
Ga0247543_103170/244-164       .UCAGGGAAACACC.A.C..U......................AUU..........................................................................................................
Ga0184589_120451/249-329       .UCAGGGAAACACC.A.C..U......................GUU..........................................................................................................
Ga0184600_118565/177-97        .CCAGGGAAACACC.A.C..U......................GUU..........................................................................................................
Ga0316034_100493/254-174       .CCAGGGAAACACC.A.C..U......................GUU..........................................................................................................
Ga0184576_117703/380-460       .CCAGGGAAACACC.A.C..U......................GUU..........................................................................................................
Ga0184597_129399/366-446       .CCAGGGAAACACC.A.C..U......................GUU..........................................................................................................
Ga0184590_111002/143-223       .CCAGGGAAACAUC.A.C..U......................GUU..........................................................................................................
Ga0184587_105985/71-151        .CCAGGGAAACACC.A.C..U......................GUU..........................................................................................................
Ga0184602_122864/595-515       .CCAGGGAAACACC.A.C..U......................GUU..........................................................................................................
Ga0184593_101086/682-762       .CCAGGGAAACACC.A.C..U......................GUU..........................................................................................................
Ga0247519_108375/286-206       .CCAGGGAAACACC.A.C..U......................GUU..........................................................................................................
Ga0127502_10351452/615-550     .CCAGGGAAACACC.A.C.........................CAU..........................................................................................................
Ga0316038_112263/442-356       .CCAGGGAAACACC.A....UA.....................CAUAU.GCGCG..................................................................................................
Ga0242657_1073895/573-672      .CCAGGGAAACAUC.A.G..UCG..ACACUGCUUG........CAUAC.UUGUA..................................................................................................
Ga0184587_107783/167-95        .CCAGGGAAACACA.U.C..A......................UCA..........................................................................................................
Ga0265758_101918/310-243       .CCAGGGAAACACG.U.C..U......................GAA..........................................................................................................
Ga0307482_1029599/655-579      .CCAGGGAAACACU.U.C..GA.....................UUAAC.A......................................................................................................
Ga0265746_1009702/779-848      .CAAGGGAAACACA.U.C.........................UUCG.........................................................................................................
Ga0184583_113025/102-26        .AUGGGGAAACAUC.A.C..U......................C............................................................................................................
Ga0184580_109961/270-194       .AUGGGGAAACAUC.A.C..U......................C............................................................................................................
Ga0316049_111814/256-181       .AUGGGGAAACAUC.A.C..U......................C............................................................................................................
Ga0247553_120631/200-275       .AUGGGGAAACAUC.A.C..U......................C............................................................................................................
Ga0184593_119849/313-238       .AUGGGGAAACAUC.A.C..U......................C............................................................................................................
Ga0184599_140898/774-849       .AUGGGGAAACAUC.A.C..U......................C............................................................................................................
Ga0242647_1005188/439-360      .CCAGGGAAACACA.A.U..GUG..U.................GCAA.........................................................................................................
Ga0242657_1054283/541-620      .CCAGGGAAACACA.A.U..GUG..U.................GCAA.........................................................................................................
Ga0184597_102100/600-669       .CCAGGGAAACACA.C.U..G......................UACA.........................................................................................................
Ga0213850_1460186/227-149      .ACAGGGAAACACA.G.A..U......................UGAAA.GUAGU..................................................................................................
Ga0242654_10040657/1018-942    .CCAGGGAAACACC.A.U..C......................AAU..........................................................................................................
Ga0307484_100842/1194-1271     .CCAGGGAAACACU.A.U..C......................UAUU.........................................................................................................
Ga0242654_10401151/84-171      .UCAGGGAAACAUU.U.C..AAG..GCAG................UU..AA.....................................................................................................
Ga0310119_105137/743-673       .CUGGGGAAACAUA.C.U..C......................GAAA.........................................................................................................
Ga0247541_104364/333-188       .UAAGGGAAACAUA.C.U..ACG..U.............CACUAAUU.........................................................................................................
Ga0184582_129125/749-675       .CCAGGGAAACACA.C.U..AC.....................AUUU.........................................................................................................
Ga0265753_1022489/457-531      .CCAGGGAAACAUA.C.U..AC.....................AUUU.........................................................................................................
Ga0265751_121301/189-266       .CCAGGGAAACACA.U.G..G......................AAAUA.A......................................................................................................
Ga0247526_105246/689-508       .CCAGGGAAACACG.C.U..UAG..G.................AAUUG.G......................................................................................................
Ga0247515_107373/548-731       .CCAGGGAAACACA.C.U..UAG..G.................AAUUG.G......................................................................................................
Ga0247538_100442/1363-1181     .CCAGGGAAACACG.C.U..UAG..G.................AAUUG.G......................................................................................................
Ga0247538_100551/721-542       .CCAGGGAAACACA.C.U..UAG..G.................AAUUG.G......................................................................................................
                               .<<<<.......<<.<.<..<<<..<<<<<<<<<<..<<<<<<.............................................................................................................
                               .2222.......22.2.2..22..................................................................................................................................
                               -●●RGGGAAACAY●-●-●--○○○--------------------○●●○○--------------------------------------------------------------------------------------------------------
```

alignment positions 601 · · · 658

```
Ga0316035_118720/195-42        .............................CG...AACA.U.AAAGAAAAUUAACUCAA
Ga0265747_125480/96-202        .............................AC...UUAU.A.AAAGUACAUUAACUUAU
Ga0242657_1353977/135-311      ..................................GUAC.U.AUAGCACAUUAUCCUCA
Ga0242649_1051170/34-143       ..............................A...CCUA.U.AAAGAAAAUUAACCUCA
Ga0265758_100138/1527-1633     ...................................CUG.U.AUAGCACAUUAACUGGG
Ga0184593_103988/348-257       ...................................UGU.U.GCGGCACAUUACUGUUC
Ga0316047_106478/13-108        ..................................AUGC.C.CAAGUACAUUAACCUAC
Ga0213850_1056422/14-139       ............AUAGCC..AUCGC.UU.UG...AGCC.G.CGGGCACAUUACUGUCC
Ga0316032_128094/136-241       .....................................U.U.GUGGCACAUUAACCAUG
Ga0307482_1000311/1469-1363    ....................................CA.C.AUGGCAUAUUACCCCUC
Ga0242657_1051800/416-321      ..............................A...C.UC.U.GUAGAAAAUUAACCAUG
Ga0307921_1050750/452-358      ............GUAAC....................A.C.UAAGCACAUUAACCCAC
Ga0265756_117960/316-418       ..............................G...AGUU.G.UCGGCACAUUACCUCCU
Ga0242654_10352202/387-307     ..............................G...GUUC.A.GAAGCACAUUACCUACG
Ga0242652_1159268/284-150      ..................................GCCA.U.UCAGCACAUUACCCAAC
Ga0265744_102237/225-365       ...................................GCA.G.UUGGCACAUUACCCGAU
Ga0316029_101396/632-732       ....................................CC.U.AAGGCACAUUACCCGUC
Ga0265759_100202/139-258       ..................................UGCU.G.GUGGCAUAUUAACCUGG
Ga0179943_1118543/65-150       .............................CU...ACUU.U.GUAGUAAAUUACCCAAG
Ga0182091_1018330/100-226      .................G..UGCAG.AC.AG....CGC.G.AAGGCACAUUACCCAGU
Ga0127503_10119710/546-465     ..................................CUAG.C.GUGGCAAAUUACCACUC
Ga0138277_1022592/399-305      ........................U.UU.GU...UUUU.G.ACAGAACAUUACCCCAC
Ga0138278_1026470/183-287      .................U..GCGA..UC.AU...CGUC.G.AUAGAACAUUACCCCAC

                               ............>>>>>>..>>>>>.>>.>>...>>>>.>.>>>.........>>>>>
                               ....................................22.2.222.........22222
                               ------------------------------------○●-●-●●RGYAYAUUAACY●●Y
```

```
Ga0138277_1089473/408-304      ................U..GCGA..UC.AU...CGUC.G.AUAGAACAUUACCCCAC
Ga0247538_105452/166-82        ...................................CA.C.ACGGUACAUUAAUCGUC
Ga0247554_102783/498-580       ...................................CA.C.ACGGUACAUUAAUCGUC
Ga0138291_1177333/295-213      ............................GU...GCUA.C.AAAGCACAUUAACUGUU
Ga0210324_1290306/191-291      .............................U...GUGC.A.UAGGCACAUUAACUGAC
Ga0242657_1026087/460-322      .............................A...CCGU.G.CUAGCACAUUACCCAGU
Ga0307482_1147486/107-4        .............................G...AGAU.U.ACGGUACCUUACCCGUG
Ga0184573_113483/125-229       ..................................UUU.G.GCGGAAAAUUACCCAUU
Ga0184576_116834/47-151        ..................................UUU.G.GCGGAAAAUUACCCAUU
Ga0184568_108419/492-395       .............................A...C.UC.U.AAGGUACAUUACCCGUU
Ga0307482_1023050/594-496      .............................G...UCCU.A.UGAGUACAUUACCCACC
Ga0179955_1013331/439-311      ......................C......A...UACC.G.CGGGCACAUUACUGUCC
Ga0184596_112078/314-191       ...........AUAGCA..CUCAC.AU.UA.....UU.G.GUGGCACAUUACUUGGA
Ga0184602_108471/49-173        ...........AUAGCA..CUCAC.AU.UA.....UU.G.GUGGCACAUUACUUGGA
Ga0247528_108647/438-562       ...........AUAGCA..CUCAC.AU.UA.....UU.G.GUGGCACAUUACUUGGA
Ga0316039_120091/354-252       ............................CU...UCCG.U.GAGAGAAAUUAACUCAU
Ga0307484_166952/62-163        .................................AGUC.A.GUGGCACAUUACCUCCC
Ga0180107_1244437/408-507      .................................ACUC.A.UCAGCACAUUACCCCUC
Ga0265742_1065119/361-273      .............................A...UUCC.G.GCAGCACAUUACGUAGC
Ga0316035_113756/173-88        .............................G...GUUC.G.ACGGCACAUUACCCACC
Ga0206355_1134705/241-332      .........................UC.AU...UGUU.G.UCGGCAUAUUAACUCAC
Ga0180107_1031754/4443-4551    ............................GA...CGCU.A.UGAGAAAAUUAGCCCAU
Ga0138292_1031932/14-100       .................................CAGA.U.ACGGCACAUUACCGCUU
Ga0316029_100807/1303-1197     .............................A...C.CC.U.AUAGCACCUUACUUGGA
Ga0184568_114257/261-413       ..................................GCU.G.GUAGCACAUUACCCAUC
Ga0242647_1005188/73-152       .................................GAUA.C.UUGGCAAAUUACCCGAC
Ga0153880_1340646/556-457      ...................................GU.G.GUAGCACAUUAACUCAU
Ga0307484_101303/704-866       ................G..GCUCC.AU.GG...GUGC.A.CGGGCACAUUACCCAGG
Ga0242641_1025149/251-89       ................G..GCUCC.GU.GG...GUGC.A.CGGGCACAUUACCCAGG
Ga0242652_1002960/413-574      ................G..GCUCC.GU.GG...GUGC.A.CGGGCACAUUACCCAGG
Ga0265754_1007706/448-552      .............................G...CGUU.A.UAAGCACAUUACCCGAC
Ga0157562_1164332/672-560      ...................................GG.G.CGGGCACCUUACCCGAG
Ga0157569_1058012/474-586      ...................................GG.G.CGGGCACCUUACCCGAG
Ga0307921_1027128/160-73       ............................AU...AGCA.A.GAAGAAAAUUACCCCAC
Ga0138279_1083071/419-326      ......................................A.GGGGUACAUUACCCGAC
Ga0127502_10704012/113-7       ...................................UC.U.GCAGCACAUUAACUGGA
Ga0222728_1000095/4541-4435    ..................................UCC.U.GUAGCACAUUAACUGGA
Ga0265750_1166784/261-415      .................................GCAG.U.GUAGCACAUUACCCGAU
Ga0310115_109341/244-400       .................................GCAG.U.GUAGCACAUUACCCGAU
Ga0265756_158829/72-188        .......................U.UA.GA...UUAA.A.ACGGUACAUUACCUGGA
Ga0184583_104740/342-477       ..................................GCG.A.GUAGCACAUUACCCGAA
Ga0180107_1215312/308-184      .............................A...GACA.G.GUGGCACAUUAACCUAG
Ga0265742_1013613/579-453      .............................U...CCCG.C.GUAGCACAUUACUCGUC
Ga0265748_110851/279-405       .............................U...CCCG.C.GUAGCACAUUACUCGUC
Ga0265752_120463/277-403       .............................U...CCCG.C.GUAGCACAUUACUCGUC
Ga0265759_115288/4-102         .................................ACCC.U.AUGGUAUAUUAACCGGU
Ga0242646_1001122/826-995      .................................UGUG.G.GUAGCACAUUAACCGGA
Ga0157593_1101573/244-147      ............................AC.....AU.G.AGAGAAAAUUACCCACC
Ga0184597_102941/191-111       ......................................G.CUAGUACAUUACCCGAC
Ga0184597_115944/155-80        ....................................A.G.CUAGUAAAUUAACUACC
Ga0265742_1051722/222-295      ....................................G.U.CCGGAAAACUAACUUCC
Ga0180107_1229893/426-318      ...................................CU.U.CGAGCACAUUAACUCUC
Ga0242654_11312609/202-83      ...........AAGGUU..GUGAG.....G...AUGC.A.AUGGCACAUUACCCGUC
Ga0187793_1464320/616-535      ............................CU...C.GU.A.GUAGCACAUUACUUCCG
Ga0187794_1206392/88-6         ............................CU...C.GU.A.GUAGCACAUUACCUCCG
Ga0316034_129624/151-284       .................................AUCG.A.AUGGCACAUUACCCGAA
Ga0184576_119948/531-664       .................................AUCG.A.AUGGCACAUUACCCGAA
Ga0184568_109070/428-286       .................................GGCC.G.GUGGUACAUUAACCUGG
Ga0265756_100717/910-1050      .............................A...GGGC.A.GUAGUACAUUAACCUGG
Ga0179955_1195671/481-563      .............................A...AGUC.U.GAGGCACAUUACUUACC
Ga0184579_120620/502-584       ...................................GA.C.AAGGAAAAUUACCCGGA
Ga0127502_10711632/191-294     .................................AUUA.C.AGAGGAAAUUAACCUUC
Ga0310119_125223/193-319       ...........UCACCU..UCAGA.AA.GU...GAAU.U.UCGGCACAUUACCUCAC
Ga0307482_1064439/425-316      .................................ACCU.A.AAGGAAAAUUAACCAUC
Ga0307484_102002/698-552       ..................................GCG.A.GUAGCACAUUACCCGGU
Ga0265741_107029/232-320       ...................A..GA.CG.GC...UACA.A.AAGGCAAAUUAUCCAAG
Ga0247515_120448/91-178        ...................GGA...CC.CC...UACA.A.AAGGCAAAUUAUCCAAG
Ga0265759_103410/556-638       ................A..GGA...CC.CC...UACA.A.AAGGCAAAUUAUCCAAG
Ga0247534_101509/435-523       ................A..GGA...CC.CC...UACA.A.AAGGCAAAUUAUCCAAG
Ga0310102_119240/132-46        ................A..GGA...CC.CC...UACA.A.AAGGCAAAUUAUCCAAG
Ga0265742_1084621/112-29       ................A..GGACU.CC......UACA.A.AAGGCAAAUUAUCCAAG
Ga0310114_135156/99-185        ................A..GGACU.CC......UACA.A.AAGGCAAAUUAUCCAAG
Ga0265752_102595/694-607       ................A..GGACU.CC......UACA.A.AAGGCAAAUUAUCCAAG
Ga0265756_105276/677-589       ................A..GGACU.CC......UACA.A.AAGGCAAAUUAUCCAAG
Ga0153880_1339022/280-371      .............AGGA........CC.CC...UACA.A.AAGGCAAAUUAUCCAAG
Ga0247518_120275/202-289       ................G..AAAGA.CG.GC...UACA.A.AAGGCAAAUUAUCCAAG
Ga0265752_100023/1618-1523     .............................A...C.CU.A.CGAGCACAUUACCCAUC
Ga0307484_102966/353-250       .................................ACUC.C.AUGGGACAUUAACCAGA
Ga0265752_101001/521-687       .............................A...AGCU.A.GUAGCACAUUAACCUAC
Ga0184593_121910/718-622       .........................GG.GU.....CA.G.AAGGUACCUUACCUCUC
Ga0184602_103319/96-192        .........................GG.GU.....CA.G.AAGGUACCUUACCUCUC
Ga0247528_108722/131-33        .........................GG.GU.....CA.G.AAGGUACCUUACCUCUC
Ga0265757_143567/145-70        .................................GUUC.A.CGAGAAGAUUAACUUGC
Ga0184592_121084/378-271       .................................AUCC.C.AUAGCACAUUAACUGGU
Ga0316037_172473/89-196        .................................UCUC.U.AUAGCACAUUAACUGGU
Ga0307921_1011071/674-791      .........................UG.UG...CUCU.U.GUAGCACAUUAACUCGC
Ga0265744_124024/357-288       ....................................C.A.ACAGUAAAUUAACCAGC
Ga0307482_1000291/592-698      .............................A...C.GU.A.UGAGCACAUUACCCGAC
Ga0184568_108869/934-1072      .............................G...GAGG.A.GUGGCACAUUACCCGAU

                               ...........>>>>>>..>>>>>.>>.>>...>>>>.>.>>>.........>>>>>
                               ...................................22.2.222.........22222
                               -----------------------------------○●-●-●●RGYAYAUUAACY●●Y
```

```
Ga0138276__1121602/329-410        ....................................UG.G.CCAGCAUAUUACCCUGC
Ga0138278__1041650/183-102        ....................................AG.G.CCAGCAUAUUACCCUGC
Ga0138277__1137481/570-489        ....................................AG.G.CCAGCAUAUUACCCUGC
Ga0138278__1041788/293-378        ....................................UG.G.CCAGCAUAUUACCCUGC
Ga0138275__1223952/371-283        ....................................UG.G.CCAGCAUAUUACCCUGC
Ga0138277__1142657/273-361        ....................................UG.G.CCAGCAUAUUACCCUGC
Ga0307482__1202605/45-154         ....................................UC.U.CUAGCACAUUACCUUCC
Ga0180107__1017343/1163-1243      ..................................UGUU.U.GAGGCACAUUACCCGGC
Ga0157560__1170105/128-245        ............GACGUA..UC........C...UAAC.U.AGAGAAAAUUAACUCAC
Ga0316030__137115/325-257         ....................................CG.U.GAAGAACAUUAACACAC
Ga0316046__103474/576-502         ..................................UAAG.C.UUGGCACAUUAUCCAAC
Ga0265754__1141843/290-185        ..............................G...CACU.G.AUAGUAAAUUAACUCCC
Ga0222729__1080556/362-213        .............................GC...UCUU.G.UUGGCACAUUAACUCGU
Ga0265740__1020297/298-150        ....................................AA.A.GCAGCACAUUAACCGUU
Ga0157569__1080558/455-605        ............AU..CA..AACAU.AC.AA...U.AU.A.CCAGCACAUUACCUGCC
Ga0307484__101264/723-941         ...................................GCA.G.GUGGCACAUUACCCAAC
Ga0213850__1513954/279-196        ..............................G...CCCG.U.UCGGUACAUUACCCGAC
Ga0316053__117700/109-179         .....................................A.C.ACAGCAUAUUAACUCAC
Ga0184582__101997/109-1           ....................................GA.A.GAAGAACAUUAACUGGC
Ga0184602__109114/416-317         ............GCUGUA..AUA................A.GUAGAACAUUAACUGAC
Ga0153880__1354682/355-454        ............GCUGUA..AUA................A.GUAGAACAUUAACUGAC
Ga0184602__110257/415-316         ............GCUGUA..AUA................A.GUAGAACAUUAACUGAC
Ga0307484__149222/136-234         ..................................UCCU.U.GUAGUACAUUAACCAGU
Ga0184572__111224/187-319         .................G..AUCUU.CC.GU...GCUU.G.AUAGCACAUUAACUGAC
Ga0138278__1022898/219-127        .............................AC...CUUG.C.GUGGUAAAUUAACCCAC
Ga0184577__108791/182-295         ............CAAGC..........G.CG...CUGC.U.GGGGUACCUUAACUGGU
Ga0265748__107452/280-387         ...........................C.GA...C.CA.U.AGGGCACAUUAACUCCU
Ga0180107__1164033/560-431        .....................................A.G.GCGGCACAUUACCUAGA
Ga0032354__1086862/688-579        ............C................GG...UGCC.A.UUGGCACAUUAACUGGG
Ga0265751__100560/274-382         ..................................AGUG.G.UAUGCACAUUACCCAGA
Ga0180107__1045142/740-541        ....................................UC.G.CUAGCAUAUUAACUGGA
Ga0213850__1296465/305-234        .......................................U.GCAGCACAUUACCCAUA
Ga0265757__111747/356-286         .......................................U.GCAGCACAUUACCCAUA
Ga0247553__104889/634-563         .......................................U.GCAGCACAUUACCCAUA
Ga0265751__102572/231-125         ....................................CC.A.AAAGGAAAUUAUCCCAU
Ga0265758__100017/4028-4134       ....................................CC.A.AAAGGAAAUUAUCCCAU
Ga0180107__1307934/226-315        ..............................U...GCAU.C.GUGGUACAUUAACCGAU
Ga0184572__112077/359-259         ..................................AUUC.A.UGAGUACAUUACCCAGU
Ga0213850__1371453/186-298        ............ACAGG.........AU.CG...CUAA.G.GAGGUACAUUAUCCAGG
Ga0213850__1469439/119-231        ................GA........AU.CG...CUUA.G.GAGGCACAUUACCCAGG
Ga0247535__113123/229-119         ............AGCGAU..GUGAGUGU.GC...GCCG.U.GAGGCACAUUACUCAGG
Ga0316036__112404/482-376         ............GG...C..AGGUC.GU.GC...GCCG.U.GAGGCACAUUACUCAGG
Ga0179943__1118543/263-176        ............AC..........A.GA.UA...GAUU.A.UGGGCACAUUAACUGGA
Ga0180108__1207316/715-601        ............AU....................UGCA.C.CCGGCACAUUAACUGAC
Ga0184578__120646/150-246         .............................AG...AUUU.C.UGUGAAAAUUAACUCAC
Ga0127503__10678853/419-333       ....................................CA.G.CAGGCACAUUAACUCAC
Ga0307919__1065410/76-162         ....................................CA.G.CAGGCACAUUAACUCAC
Ga0180108__1209503/1932-2029      ....................................CA.G.UUGGCACAUUAACCUUC
Ga0180108__1022680/3902-3998      ....................................CA.G.AUGGUACAUUACCCUAC
Ga0316031__107021/319-214         ....................................UC.A.AGAGUAAAUUAACUCCC
Ga0247527__116942/161-88          ...................................AAA.A.CGAGCAUAUUAACUGGC
Ga0307484__100434/1330-1210       ............A....G..AGGAC.CG.GA...UUCG.U.GAGGUACAUUAACCUAC
Ga0307482__1160392/324-400        ....................................CA.G.AUGGUAAAUUAACCCAC
Ga0316033__105623/339-253         ...........................U.GC...CACU.U.GCAGUACAUUACCCGAC
Ga0265754__1037910/322-179        .............................GG...GCGU.U.GUAGCACAUUAACCAGG
Ga0307482__1048753/383-528        ...................................GCG.A.GUAGCACAUUACCCGUC
Ga0265752__102479/223-81          ..............................C...UACU.U.GUUGCAUAUUACCCGAC
Ga0265746__1084801/20-158         ............AUGGUA..UACUU.UA.CC...UACU.U.GUUGCAUAUUACCCGAC
Ga0265756__107066/520-660         ............AUGGUA..UAAUU.UA.CC...UACU.U.GUUGCAUAUUACCCGAC
Ga0265742__1022009/340-200        ....................................CU.U.GUUGCAUAUUACCCGAC
Ga0265748__106902/213-353         ....................................CU.U.GUUGCAUAUUACCCGAC
Ga0180052__116799/419-501         ..................................UAUC.U.AUGGCACAUUACCCGUC
Ga0115594__1106220/441-334        .................A..GCG...........UCUC.A.GCAGCACAUUAGUCGGC
Ga0247534__117029/65-195          ............GAGGAG............A...GGCG.G.GUGGAACAUUACCCGGC
JGI12272J11983__1072695/40-150    ..............................A...C.CC.A.GUAGCACAUUAACUCCC
Ga0247552__100007/4311-4420       ....................................CC.U.CUUGCACAUUACUUGGC
Ga0184596__107732/313-205         ....................................CC.U.CUUGCACAUUACUUGGC
Ga0247542__100007/609-500         ....................................CC.U.CUUGCACAUUACUUGGC
Ga0247534__100047/1899-2008       ....................................CC.U.CUUGCACAUUACUUGGC
Ga0184598__129111/512-403         ....................................CC.U.CUUGCACAUUACUUGGC
Ga0184597__128933/573-464         ....................................CC.U.CUUGCACAUUACUUGGC
Ga0184582__108821/1050-1159       ....................................CC.U.CUUGCACAUUACUUGGC
Ga0247541__100010/3268-3377       ....................................CC.U.CUUGCACAUUACUUGGC
Ga0184580__121439/573-464         ....................................CC.U.CUUGCACAUUACUUGGC
Ga0265756__101275/650-541         ....................................CC.U.CUUGCACAUUACUUGGC
Ga0184575__101094/586-477         ....................................CC.U.CUUGCACAUUACUUGGC
Ga0184594__103311/207-316         ....................................CC.U.CUUGCACAUUACUUGGC
Ga0247554__100006/1592-1485       ....................................CC.U.CUUGCACAUUACUUGGC
Ga0247522__108409/684-554         ...................................GCG.A.GUAGCACAUUACCCGGU
Ga0247534__100003/3147-3245       ....................................CU.A.UGAGCACAUUACCCAUC
Ga0265751__100393/1594-1698       ....................................CU.G.GUAGCAUAUUAACCUCC
Ga0265751__124807/392-283         ..................................ACCG.A.CGUGCACCUUACCCGGA
Ga0310107__144222/327-436         ..................................ACCG.A.CGUGCACCUUACCCGGA
Ga0265743__103007/946-842         ..............................G...CCAU.C.AUGGUAUAUUAACUUCC
Ga0316038__107364/55-195          ....................ACCGG.UA.UG...CUCA.C.UGAGCAUAUUACCUGAC
Ga0184593__123391/157-297         .....................CCGG.UA.UG...CUCA.C.UGAGCAUAUUACCUGAC
Ga0222728__1003482/1262-1107      ...................................GCG.G.UUGGCACAUUACCUCAC
Ga0242654__10701525/53-296        ....................................CC.A.UAGGCACAUUAACCGGA
Ga0242654__11172691/285-48        ....................................CC.A.CAGGCACAUUAACCGGA

                                  ............>>>>>>..>>>>>.>>.>>...>>>>.>.>>>.........>>>>>
                                  ....................................22.2.222.........22222
                                  ------------------------------------○●-●-●●RGYAYAUUAACY●●Y
```

```
Ga0316038_100042/1676-1569       ...................................CA.U.AGGGCACAUUAACUCCU
Ga0307921_1043977/91-164         ......................................C.UCGGCACAUUAACUAUC
Ga0079039_1293848/95-22          ...................................UC.U.GCGGUACAUUACCCAGC
Ga0247534_101560/228-122         ...................................UC.A.CUGGCACAUUACCCGUC
Ga0153880_1379927/66-144         ..................................CCA.G.GCAGCACAUUAAACCCC
Ga0153880_1391790/62-140         ..................................CCA.A.GUAGCACAUUAAACCCC
Ga0307482_1048660/422-625        ..................................GCC.A.GUAGCACAUUAACUGAC
Ga0180108_1022680/4248-4153      .............................A...C.AC.C.AUAGCACAUUAACUGGU
Ga0247516_125012/316-428         ............................CC...CCAG.A.CUGGGAUAUUACCCGGA
Ga0242652_1038498/365-470        .............................U...CCAG.U.CAGGCACAUUAACUCUC
Ga0210323_1050281/92-187         ..................................GCA.C.UUGGUACAUUAACUGGC
Ga0224712_10542545/154-227       ...................................UC.A.CUGGCACAUUAACUCUC
Ga0184602_130775/1275-1431       .............CGGG..AGUCC.AA.GG...UGUA.C.GUAGCACAUUAACCUGG
Ga0206355_1311803/144-334        .......................A.UC.AC...CCAA.U.ACGGUAAAUUAACUGGC
Ga0310119_152175/242-389         ...........GUAGCG..GAGUA.UGAGG...CACA.C.GUAGCACAUUAACUUGC
Ga0255059_10691632/166-76        .............................U...UCCG.A.GUAGCACCUUAACUGGC
Ga0255062_11230321/200-110       .............................U...UCCG.A.GUAGCACCUUAACUGGC
Ga0265743_143797/241-325         ....................................G.U.CGGGCACAUUACCCAGC
Ga0247534_117055/74-212          ...........AGCCCG....CGA....GG...GCUU.G.CUAGCACAUUAACUGGA
Ga0213850_1282564/267-350        ...................................AC.G.GCGGGACAUUACCCGAC
Ga0315826_123252/262-347         ...................................AC.G.GCGGGACAUUACCCGAC
Ga0315817_133437/498-413         ...................................AC.G.GCGGGACAUUACCCGAC
Ga0184577_116723/415-311         ...........AUAGGU..AA...............A.C.ACGGCACAUUAACUGCC
Ga0184576_109717/432-536         ...........AUAGGU..AA...............A.C.ACGGCACAUUAACUGCC
Ga0184572_113323/230-300         ....................................A.C.ACGGCACAUUACCCCUA
Ga0179935_1139075/165-78         ..........................C.UA...CUUA.U.GUAGCACAUUACCUGGC
Ga0179944_1246019/249-162        ..........................C.UA...CUUC.U.GUAGCACAUUACCUUGC
Ga0213850_1307525/101-186        .................................AGAC.G.UUAGCAUAUUACCUCUC
Ga0222728_1062510/166-66         ....................................U.G.AGAGAAUAUUAACUCAC
Ga0265743_101198/448-619         .............................G...UCAU.C.AUGGCAUAUUAACUGGA
Ga0180108_1209503/2438-2343      .............................A...C.AC.U.AUGGUACAUUAACUGGA
Ga0184583_103998/587-740         .................................GGCU.A.GUAGCAUAUUAACCGAC
Ga0184595_100702/414-262         .................................GGCU.A.GUAGCACAUUAACCGAC
Ga0184592_125105/67-219          .................................GGCU.A.GUAGCACAUUAACCGAC
Ga0224712_10002011/1564-1454     .............................A...C.CU.G.GUGGAAUAUUAACUCCU
Ga0316049_114344/181-288         ............................CU...GCUA.C.GCAGCACAUUAACUACC
Ga0184576_110594/6-113           ............................CU...GCUA.C.GCAGCACAUUAACUACC
Ga0316037_112281/325-219         ............................CU...GCUA.C.GCAGCACAUUAACUACC
Ga0316046_116376/278-384         ............................CU...GCUA.C.GCAGCACAUUAACUACC
Ga0247534_102735/124-18          ............................CU...GCUA.C.GCAGCACAUUAACUACC
Ga0247540_102569/210-104         ............................CU...GCUA.C.GCAGCACAUUAACUACC
Ga0247529_116306/255-362         ............................CU...GCUA.C.GCAGCACAUUAACUACC
Ga0184568_104528/922-1000        .................................AAAU.U.CCAGUACAUUAACUCAU
Ga0184578_112031/156-44          ...................UUCUA.GU.UA...CCAU.A.CUAGGAUAUUAACUCAC
Ga0247517_121471/387-499         ...................AUCUA.GU.UA...CCAU.A.CUAGAAUAUUAACUCAC
Ga0184569_108885/200-88          ................C..AACUG.C..UA...CCAU.U.GCAGUAAAUUAACUCAC
Ga0247521_112559/317-205         ................C..AACUG.C..UA...CCAU.U.GCAGUAAAUUAACUCAC
Ga0247539_101233/414-526         ................C..AACUG.C..UA...CCAU.U.GCAGUAAAUUAACUCAC
Ga0265756_100485/1108-1000       .............................G...CAGU.U.CGGGCAAAUUAUCCGUC
Ga0265751_100417/1480-1588       .............................G...CAGU.U.CGGGCAAAUUAUCCGUC
Ga0213850_1295854/263-370        ..............UCG..UUGGG.UG.CC...UUGU.A.AAGGCACAUUAACUCAC
Ga0307919_1029784/253-144        ...........UC...G..UUGGG.UG.CC...UUGU.A.AAGGCACAUUAACUCAC
Ga0307921_1042549/343-452        ...........UC...G..UUGGA.UG.CC...UUGU.A.AAGGCAUAUUAACUCAC
Ga0307484_102966/727-825         ...................................AG.C.AAAGCACAUUAACUCCU
Ga0184588_131258/156-253         ...................................AU.A.GUGGUACAUUACUUAUG
Ga0224712_10010317/1973-2082     ...................................CU.G.AAAGGAAAUUAACUCUC
Ga0224712_10601975/393-502       ..................................ACU.G.AAAGGAAAUUAACUCUC
Ga0222728_1000118/2272-2196      .................................AUCA.G.ACGGCACAUUACCCGAC
Ga0115594_1200134/154-31         ...........GCACAU..GUC......CC...CCUA.C.AGAGCACAUUACCCGAC
Ga0115595_1153837/320-197        ...........GCACAU..GUC......CC...CCUA.C.AGAGCACAUUACCCGAC
Ga0255282_1067513/33-105         ...................................CC.G.GGAGCAUAUUACCCGCC
Ga0242647_1005934/451-617        ...................................CC.G.AUGGCACAUUAACCGGA
Ga0307482_1449537/408-223        ..................................GCU.C.GUAGCACAUUACCUGAC
Ga0115594_1022316/346-239        ............................CG...UCUC.A.GCAGCACAUUAGCCGGC
Ga0115595_1072788/334-441        ............................CG...UCUC.A.GCAGCACAUUAGCCGGC
Ga0184568_108419/50-147          .................................AUAA.C.AGAGCACAUUAACUCUC
Ga0247534_100007/116-225         .................................UCUC.U.AGAGCACAUUAACUCCU
Ga0184569_109084/220-111         .............................G...UCUC.U.AGAGCACAUUAACUCCU
Ga0316033_100036/207-98          .............................G...UCUC.U.AGAGCACAUUAACUCCU
Ga0184578_112042/249-140         .................................UCUC.U.AGAGCACAUUAACUCCU
Ga0184594_123034/256-94          .................................UCGC.A.GCGGCAUAUUAACUGGA
Ga0256798_1305521/221-305        ....................................C.A.GCAGCACAUUAACCACG
Ga0184595_116681/414-266         ..................................GCG.A.GUAGCACAUUACCCGAU
Ga0184568_105547/129-239         ....................................C.C.GCAGCACAUUAACUCGG
Ga0157559_1099797/360-209        ............................CU...CCAU.U.GAAGCACAUUAACUGGU
Ga0157572_1089974/360-209        ............................CU...CCAU.U.GAAGCACAUUAACUGGU
Ga0316037_108094/19-160          ...................................UG.C.GGGGCAUAUUAACUAGC
Ga0316038_107116/33-174          ...................................UG.C.GGGGCAUAUUAACUAGC
Ga0210323_1072445/691-543        .............................G...GCUG.U.CGGGCACAUUACCCGAC
Ga0307482_1000707/704-773        ....................................U.G.AUGGUACAUUAACCGAG
Ga0079039_1671802/197-34         .............................C...UCAC.A.GAGGUACAUGGACUGGU
Ga0247533_103342/137-220         .......................C.UC.AU...UCUA.A.ACAGCACAUUAACCCGC
Ga0157565_1016893/233-334        ...........AUUGAG..AGA.G.AA.UC...GUUG.U.GUGGAAAAUUACCCGAU
Ga0180047_1087048/281-180        ...........AUUGAG..AGAGA.A..UC...GUUG.U.GUGGAAAAUUACCCGAU
Ga0157593_1006201/239-338        ................G..CUGAG.AA.UC...GUUG.U.GUGGAAAAUUACCCGAU
Ga0157560_1010344/357-456        ................G..CUGAG.AA.UC...GUUG.U.GUGGAAAAUUACCCGAU
Ga0316049_154526/155-74          ......................................U.GCAGCACAUUAACCCAC
Ga0316035_177575/224-142         ......................................U.GCAGCACAUUAACCCAC
Ga0316033_101029/151-49          ...................................GC.C.UGAGCACAUUAACUGUC

                                 ...........>>>>>>..>>>>>.>>.>>...>>>>.>.>>>.........>>>>>
                                 ...................................22.2.222.........22222
                                 -----------------------------------○●-●-●●RGYAYAUUAACY●●Y
```

```
Ga0206356__10600373/215-21      ...........................U......AACG.U.GAGGCACAUUAACUGCC
Ga0307482__1009758/562-669      ....................................CU.G.ACAGCAUAUUAACUCCC
Ga0213850__1245299/581-409      ....................................CU.C.GCGGCACAUUAACUGAC
Ga0180107__1134582/382-542      ...................................CCC.A.CAGGCACAUUAACUGCG
Ga0307484__100970/951-1026      ...................................GGA.U.GUGGCAUAUUACCCAGA
Ga0307919__1034281/527-451      ....................................AU.G.AAGGUACAUUAACUCAC
Ga0307921__1020694/406-483      ....................................AU.G.AAGGUACAUUAACUCAC
Ga0316033__105623/3-98          .......................................C.GUGGCAUAUUAACUCAC
Ga0265743__111644/592-512       ..............................G...CCUA.G.GCAGCACAUUAACUGCC
Ga0265755__108708/373-453       ..............................G...CCUA.G.GCAGCACAUUAACUGCC
Ga0316038__121313/184-264       ..............................G...CCUA.G.GCAGCACAUUAACUGCC
Ga0265756__109326/116-196       ..............................G...CCUA.G.GCAGCACAUUAACUGCC
Ga0265759__104327/339-243       ...............ACG..GCUCU.UG.AA...CGAG.C.CUGGUACAUUAACUCAC
Ga0316035__144545/195-301       ...........................A.AU...C.UC.G.UGGGUACAUUACCUCAC
Ga0265743__108998/150-253       ....................................GU.G.AUAGUAAAUUAACUCUC
Ga0153880__1067318/492-566      ....................................UA.U.GUAGUACAUUAACUCGG
Ga0180044__1047678/444-368      ....................................AA.A.GUAGCACAUUACCCAGU
Ga0157564__1065218/271-353      ..................................ACAA.U.GUAGCACAUUACCUAGC
Ga0180044__1069332/269-187      ..................................ACAA.U.GUAGCACAUUACCUAGC
Ga0242647__1014694/423-336      .....................................A.G.AUGGUAAAUUAACUUGC
Ga0180108__1143860/199-116      .....................................G.G.AUGGAAAAUUAACUGCC
Ga0265744__100073/1235-1401     ..............................G...UCAC.A.GGAGCACAUUAACUGGA
Ga0307482__1054608/326-205      .......................................G.UCAGCAUAUUAACUAAC
Ga0242646__1008525/229-119      .......................................G.UCAGCAUAUUAACUAAC
Ga0153880__1260556/441-589      ..............................C...AGUA.U.GUAGCACAUUAACCUGC
Ga0127503__10814644/496-352     .....................AACU.CU.UU...CGAG.U.UUAGCACAUUAACUGAC
Ga0326631__105052/171-77        .....................................G.A.GCAGCACAUUAACUGGA
Ga0307920__126542/349-261       .....................................U.G.GCGGCAUAUUAACUAGA
Ga0247533__100162/1643-1535     ....................................CU.A.CUGGAAAAUUAACUCUC
Ga0316047__100015/734-626       ....................................CU.A.CUGGAAAAUUAACUCUC
Ga0265743__101537/1064-984      ....................................GC.G.UGGGCACAUUAACCAGC
Ga0316030__100208/805-700       ....................................UC.A.UCAGAAUAUUAACUCUC
Ga0316050__103193/566-460       ....................................GU.G.AUAGCAUAUUAACUCUC
Ga0184601__113231/247-149       ....................................CU.C.GUGGUACAUUACCCGGC
Ga0307350__148584/139-224       .........................U.UG.GU...GUGC.G.GAAGCACAUUAACUCAC
Ga0307482__1018929/1182-1081    ..............................G...CACU.G.AUAGUAUAUUAACCGGC
Ga0307482__1000262/4800-4699    ..............................G...CACU.G.AUGGUAUAUUAACCGGC
Ga0316047__100860/1040-888      ..............................U...CCAG.U.GUAGCACAUUAACUGAG
Ga0242654__10068518/113-218     ..................................GCCU.U.GAGGCACAUUAACUUCC
Ga0222729__1010500/11-117       ..............................A...GCCU.U.GAGGCACAUUAACUUCC
Ga0307484__101172/602-495       ..............................A...GCCU.U.GAGGCACAUUAACUUCC
Ga0242647__1002406/915-1021     ..............................A...GCCU.U.GAGGCACAUUAACUUCC
Ga0307483__1000720/1178-1064    ..............................G...C.GU.U.GAGGUACAUUAACUUCC
Ga0307921__1022947/450-369      ............G.................C...GGCU.C.GCAGCACAUUAACUCAC
Ga0153880__1432604/289-395      ..............................U...CA.U.U.CUGGCACAUUAACCCGC
Ga0247533__101442/511-432       ..................................AUAC.A.GCAGCACAUUAACUCAU
Ga0242647__1002473/759-521      ....................................CC.A.ACGGCACAUUAACUGGU
Ga0256352__1087084/23-118       ............AUUGCU..UUAAU.AU.......GGC.G.CGGGCACAUUACCCUUC
Ga0157562__1157806/189-74       .....................UCUG.AU.UU...GAGC.G.CGGGCACAUUACCCUAC
Ga0153880__1457361/236-121      ............UUAGUA..AUGAU.UU.GA.....GC.G.CGGGCACAUUACCCUAC
Ga0157559__1128929/201-87       ............UUAAUU..UUAAU.UU.GA.....AC.G.CGGGCACAUUACCCUAC
Ga0157566__1012736/497-384      ............UUAAUU..UUAAU.UU.GA.....GC.G.CGGGCACAUUACCCUAC
Ga0157579__1155702/323-436      ............UUAAUU..UUAAU.UU.GA.....GC.G.CGGGCACAUUACCCUAC
Ga0316036__116159/257-326       ....................................GA.A.UGGGUAAAUUAACUCAC
Ga0180047__1032966/152-37       ....................................GC.G.CGGGCACAUUACCCUAC
Ga0184587__125934/78-152        ...................................ACC.U.CCAGAAAAUUAACCAGU
Ga0179934__1155244/114-26       .......................CA.UU.CU...AUCG.A.GCAGCACAUUAACUCAC
Ga0307482__1623775/241-347      ....................................CG.C.AUGGCAUAUUAACUCCU
Ga0307484__101582/1018-1124     ....................................CG.C.AUGGCAUAUUAACUCCU
Ga0307484__111921/444-550       ....................................CG.C.AUGGCAUAUUAACUCCU
Ga0184587__113157/56-168        ...........................G.GC...AUGC.C.GUGGCACAUUACCCGGU
Ga0255228__1272153/272-192      ............AACGC..................GCA.C.UUGGAAUAUUAACUAAC
Ga0153880__1325448/483-575      ..........................CC.UU...CGUA.A.GUAGCACAUUACCUAAC
Ga0157565__1130498/91-183       ..........................CC.UU...CGUA.A.GUAGCACAUUACCUAGC
Ga0265758__129208/41-120        ....................................CU.A.CCGGUAAAUUAACUGAC
Ga0265756__146329/172-94        ....................................UU.U.ACGGUAAAUUAACUGAU
Ga0115594__1180137/311-161      ..............................C...CCCC.G.CGAGCACAUUAACUGGC
Ga0265744__100861/398-567       ..............................G...UCAU.C.ACGGCAUAUUAACUGGA
Ga0180107__1285918/13-113       .......................................G.CGGGCACAUUAACUACC
Ga0247519__103291/1381-1460     .....................................G.A.UGGGCACAUUAACCACA
Ga0184603__106561/141-62        .....................................G.A.UGGGCACAUUAACCACA
Ga0247551__100996/477-556       .....................................G.A.UGGGCACAUUAACCACA
Ga0184590__126350/300-380       .....................................G.A.UGGGCACAUUAACCACA
Ga0265756__131580/159-58        ............ACGCUU..GU...............A.C.GUAGCACAUUAACUCGC
Ga0307482__1000325/24-131       ..................................ACCG.A.UAAGUAAAUUACCCAGC
Ga0242646__1001086/737-571      ..................................UACA.U.GUAGCAUAUUAACUGGA
Ga0265747__132694/181-66        .........................A.CA.GA...G.CA.G.UCAGCAUAUUAACUGAC
Ga0157593__1023702/238-314      .....................................C.A.GCGGCACAUUACCCAAC
Ga0316053__103654/814-736       ..................................UUCC.C.CCGGCAAAUUACCCAUC
Ga0247529__117158/253-331       ..................................UUCC.C.CCGGCACAUUACCCAUC
Ga0180107__1129325/244-60       ....................................GC.U.GAGGCACAUUAACUGAC
Ga0307482__1162833/232-148      .........................C.UG.UG...AGCU.U.UCGUCAUAUUAACUGAC
Ga0255267__1268184/91-184       .......................................A.GCGGCACAUUACCCGAC
Ga0307919__1064641/225-73       ...................................UAG.G.GCGGCACAUUAACUCUC
Ga0307921__1075885/188-36       ...................................UAG.G.GCGGCACAUUAACUCUC
Ga0265742__1021317/385-459      .......................................C.ACGGCACAUUAACCCAC
Ga0307483__1002304/791-713      .......................................A.CCGGAAAAUUACCCAGC
Ga0307919__1165008/203-116      .......................UU.AG.GU...UUAA.U.GUGGCACAUUAACUCAC
Ga0210323__1093898/306-192      ..............................G...CUGC.C.GCGGCACAUUAACUGGC

                                ............>>>>>>..>>>>>.>>.>>...>>>>.>.>>>.........>>>>>
                                ......................................22.2.222.........22222
                                ------------------------------------○●-●-●●RGYAYAUUAACY●●Y
```

```
Ga0265743_119695/104-184       ...................................UUC.A.GCAGCACAUUAACGACC
Ga0310118_104706/1022-1118     .................G..AGUCC.....A...AAUG.A.UGGGUACAUUAACUGCU
Ga0307921_1005950/551-460      ....................................UC.A.UCGGUACAUUAACUCGC
Ga0242641_1014724/551-384      .................G..UCCGU.UU.GG...GUAG.C.CGAGCACAUUAACUGGA
Ga0242641_1002366/499-331      .................G..UCCGU.UU.GG...GUAG.C.CGAGCACAUUAACUGGA
Ga0242657_1007384/290-215      ..................................GGCA.U.AGUGCACAUUAACUGGC
Ga0265757_113513/277-362       ............CUGGC....................U.G.GUGGCACAUUAACUCAC
Ga0242646_1001007/790-609      .................G..GCCCU.UU.GG...UGAC.A.GAGGCAUAUUAACUGAG
Ga0316034_107719/314-205       ....................................UU.G.ACAGCAUAUUACCUCCC
Ga0316039_119781/347-457       ....................................UC.A.UCUGCACAUUAACUCCC
Ga0255295_1078742/236-408      ...........................G.GU...CUAG.A.UGUUUAUAUUAACUGGC
Ga0184603_150346/629-518       ....................................CU.A.ACGGUACAUUACCUCUC
Ga0180106_1187148/166-89       ....................................AG.G.GUGGCAUAUUAACCACC
Ga0307482_1417159/374-296      ....................................AG.G.GUGGCAUAUUAACCACC
Ga0222728_1000095/4027-4133    ....................................CU.G.AUAGUAAAUUAACCUCC
Ga0316030_105840/623-504       ....................................UG.U.GUGUUAUAUUAACUUGC
Ga0316033_110175/528-409       ....................................UG.U.GUGUUAUAUUAACUUGC
Ga0326631_124620/105-175       ....................................UG.U.GUGUUAUAUUAACUUGC
Ga0206356_11590518/224-68      .................C..GGCUA.UG.UU...CUAU.G.GAGGUACAUUAACUUGC
Ga0213850_1246156/171-47       ...........................G.GU...GCAU.C.GUAGCACAUUAACUGGC
Ga0184599_140450/516-588       .....................................G.U.CGGGUACAUUAACUGCU
Ga0206356_11002907/329-148     .............................AC...UUUU.C.AUGGCACAUUAACUGGC
Ga0307482_1001469/2898-2828    .......................................G.GUGGUACAUUAACCAAC
Ga0157570_1089798/68-159       ............GGGGG...................GG.G.AGGGCAAAUUAACCUCC
Ga0127503_11028958/51-130      .....................................G.U.ACGGCACAUUAACUCAC
Ga0157560_1126722/83-169       ..................................CAUG.C.UUGGAAAAUUAACUGGC
Ga0138277_1028338/433-516      .....................................U.A.GUAGCACAUUAACUCAC
Ga0265742_1001214/55-127       ....................................UU.U.GUAGCACAUUAACUCAC
Ga0115594_1134035/652-542      ..........................AA.GC...UCUU.U.GUAGCACAUUAACUCAC
Ga0115594_1212468/8-118        ......................GGU.AU.GC...UCUA.U.GUAGUACAUUAACUCAC
Ga0247533_100438/1040-888      ..............................U...CCAG.U.GUAGCACAUUAACUGAG
Ga0326631_117973/181-293       ...........................G.CG...C.CU.U.GCGGCACAUUAACGAGC
Ga0184600_117644/32-108        .....................................G.A.UGAGUAAAUUAACUACC
Ga0184584_133603/317-393       .....................................G.A.UGAGUAAAUUAACUACC
Ga0316052_100527/264-188       .....................................G.A.UGAGUAAAUUAACUACC
Ga0184580_125947/153-229       .....................................G.A.UGAGUAAAUUAACUACC
Ga0247524_105844/561-637       .....................................G.A.UGAGUAAAUUAACUACC
Ga0184580_109821/480-551       ....................................UC.A.UGAGGAAAUUAACCACC
Ga0184598_107135/96-25         .....................................C.A.UGAGGAAAUUAACCACC
Ga0316051_1015080/488-559      .....................................C.A.UGAGGAAAUUAACCACC
Ga0247524_121352/374-303       .....................................C.A.UGAGGAAAUUAACCACC
Ga0179934_1197833/207-115      .......................................A.GAGGUACAUUAACUUCC
Ga0316049_129960/371-262       ............GUCACU..CAACA.AU.GA...C.UC.U.GUAGCACAUUAACUCAC
Ga0265747_125301/405-306       ............GAAG..................UAAU.U.GUAGUACAUUAACUGGA
Ga0157570_1034241/151-229      ..................................GCUG.C.ACGGUACAUUACCCGUC
Ga0157571_1064089/212-134      ..................................GCUG.C.ACGGUACAUUACCCGUC
Ga0242652_1041630/308-377      ....................................CG.G.UAGGCACAUUAACUGCC
Ga0307480_1006257/684-599      ....................................GU.G.CAGGCACAUUACCUAGC
Ga0310119_142698/346-430       .......................CG.GC.GU...U.UU.C.AGGGCACAUUAACUGGA
Ga0307482_1074296/478-354      ............GUGCCU..U...............CA.U.CGGGCACAUUAACUGAC
Ga0307483_1005049/26-150       ............GUGCCU..U...............CA.U.CGGGCACAUUAACUGAC
Ga0265757_127696/205-308       ............GAGUGG.................CCU.C.GCAGCACAUUAACUUGC
Ga0316029_101202/440-551       ....................................CC.A.CGAGCACAUUAACUGGC
Ga0307482_1134687/287-181      ..................................ACUC.G.GCGGCACAUUAGCUUCC
Ga0307484_104189/399-292       ..................................ACUC.G.GCGGCACAUUAGCUUCU
Ga0307921_1050271/251-412      ..................................UUAG.G.GCGGCACAUUAACUCUC
Ga0184580_102899/442-582       ...........................U.GG...CCUU.A.ACGGCACAUUAACUGGC
Ga0316029_136733/88-8          ....................................UG.G.UCAGUAUAUUAACUUGC
Ga0180107_1174136/218-102      .......................................U.GAAGAAAAUUAACUGGC
Ga0179948_1254202/198-296      .................U..AAGC............AC.G.CGGGCAAAUUACCUAGC
Ga0265746_1140777/214-319      ....................GAGUG.UG.......CCC.A.GCAGCACAUUAACUUGC
Ga0265756_108694/382-277       ............AGUGUG..U...............CC.A.GCAGCACAUUAACUUGC
Ga0265758_129717/201-94        ....................GAGAU.UG.C......CC.A.GCAGCACAUUAACUUGC
Ga0213850_1394829/253-105      ..............................U...CACG.A.UGAGCACAUUAACUGGC
Ga0184603_139562/141-280       ............GGGGAU..UAGGU.GU.GA...GCAA.A.UCAGAAAAUUAACCCGC
Ga0157560_1100546/299-385      ..................................CUAA.C.ACGGCACACUAACUAUC
Ga0129331_1494381/292-368      ....................................GA.C.CGAGCACAUUAACUUGC
Ga0265759_100473/818-988       ....................................CC.U.ACGGCACAUUAACUGAC
Ga0265746_1003835/1049-1219    ....................................CC.U.ACGGCACAUUAACUGAC
Ga0265756_100592/1458-1288     ....................................CC.U.ACGGCACAUUAACUGAC
Ga0247536_100773/637-807       ....................................CC.U.ACGGCACAUUAACUGAC
Ga0184578_101068/480-310       ....................................CC.U.ACGGCACAUUAACUGAC
Ga0247530_109035/489-319       ....................................CC.U.ACGGCACAUUAACUGAC
Ga0247513_128543/241-71        ....................................CC.U.ACGGCACAUUAACUGAC
Ga0184573_112336/323-153       ....................................CC.U.ACGGCACAUUAACUGAC
Ga0127502_10575752/515-449     .....................................G.U.AGAGCACAUUACCCGGC
Ga0180107_1033011/786-679      ...................................AUG.U.GGAGAAGAUUACCCGGA
Ga0222728_1380294/134-238      ....................................GA.U.AGAGCACAUUACCCGUC
Ga0307484_107311/306-374       .........................................GGGGCACAUUAACCAAC
Ga0316046_107255/463-562       .....................................G.U.GGAGCACAUUACCCAGU
Ga0184570_114412/209-338       ............UUCCAA..UCCAG.GG.AU...CAUU.C.UAAGUAAAUUAACUUGC
Ga0242646_1000880/695-856      .................G..CCACU.UG.GG...UCAU.A.GAGGCACAUUAACUGGA
Ga0316031_123456/415-346       ....................................GC.U.GCAGCACAUUAGCCAGC
Ga0157630_1203478/203-99       ...............GAC..AAAAA.GU.GA...A.CU.U.GUAGCACAUUAACCCGC
Ga0247541_102707/328-184       .............................GU...GCAC.G.UUAGCACAUUAACUGGC
Ga0115594_1174394/642-729      ..................................AAGU.A.AGGGCACAUUAACUCCC
Ga0115594_1187657/870-960      .............................AA...UUAU.A.AGGGCACAUUAACUCCC
Ga0242646_1047905/92-182       ....................................GG.G.GUAGCACAUUAACUCAC
Ga0247541_101130/558-391       ....................................UC.C.ACGGCACAUUAACUGAC

                               ............>>>>>>..>>>>>.>>.>>...>>>>.>.>>>.........>>>>>
                               ....................................22.2.222.........22222
                               ------------------------------------○●-●-●●RGYAYAUUAACY●●Y
```

```
Ga0307921__1062541/180-338        ...................................UAG.G.GCGGCACAUUAACUCUC
Ga0184572__105168/388-281         ....................................CC.A.AUGGUACAUUAACCAAC
Ga0316036__127178/124-231         ....................................CC.A.AUGGUACAUUAACCAAC
JGI12272J11983__1345183/364-258   ..............................A...C.CU.G.AUGGAAAAUUAACUCCC
Ga0247535__100068/719-828         ..............................G...CCGC.A.GUGGUAUAUUAACUCCC
Ga0222728__1000079/1192-1299      ..............................A...C.UU.G.AUAGCACAUUAACUCCC
Ga0081246__1201717/153-259        ..............................A...C.AC.A.UCGGAAAAUUAACUCCC
Ga0222728__1216887/51-157         ..............................A...C.UC.A.GUGGUACAUUAACUCCU
Ga0157569__1073490/184-258        .....................................G.A.GGAGCACAUUACCUGGC
Ga0180047__1061805/548-622        .....................................G.A.GGAGCACAUUACCUGGC
Ga0206353__11976089/292-380       ..................................GUAA.A.UCGGCACAUUAACUGAC
Ga0247553__101094/709-629         .....................................C.G.UGGGCACAUUAACCAGC
Ga0242641__1004288/32-112         .....................................U.G.GGGGUACAUUAACCAGC
Ga0242641__1014474/336-257        ....................................AG.A.GUAGCACAUUACCCGAC
Ga0126318__10199518/109-204       ..................................GGGC.U.GGGGCACAUUAACUCAC
Ga0307919__1109364/247-158        ...........................C.CG...UUCU.U.CUGGUACAUUAACUAAC
Ga0180107__1307618/257-174        ..............................A...GGAA.G.GCAGCAUAUUAACUCAC
Ga0179937__1126086/216-325        .......................CC.GU.GA...CUGU.A.GUAGCACAUUAACUUCC
Ga0265744__134092/134-214         ..............................A...UGAA.A.GUGGUACAUUAACUUGC
Ga0307482__1015399/786-719        .......................................A.GGAGCAUAUUAACUGGC
Ga0265751__100477/874-675         .............................GG...CAAC.A.GUAGCACAUUAACUGGA
Ga0265756__100433/485-286         .............................GG...CAAC.A.GUAGCACAUUAACUGGA
Ga0265743__100639/33-191          ...........................A.GG...CAAC.A.GUAGCACAUUAACUGGA
Ga0184602__130623/1321-1100       .............................GU...GAUA.A.GGGGUACAUUAACUGGA
Ga0184597__126105/585-362         ..................................GAUA.A.GGGGUACAUUAACUGGA
Ga0187793__1322799/72-151         .....................................C.C.CUGGUACAUUAACCUGC
Ga0316037__128286/253-347         ...........................A.GG...CUCG.C.GUAGCAUAUUAACUGAC
Ga0265758__109576/83-157          .......................................A.GCGGAAAAUUAACUGGG
Ga0265751__100127/176-103         .....................................G.U.GGUGCACAUUAACUGGC
Ga0265758__108618/113-22          .............................GA...C.GC.G.UUAGCAUAUUAACUGCC
Ga0228708__1128185/228-344        ............UAGGU.................UGC..U.AAGGUAAAUUAACUUGC
Ga0256309__1199458/363-444        ...........................G.UC...AGCC.A.AAGGUAAAUUAACUCUU
Ga0256302__1090383/76-157         ...........................G.UC...AGCC.A.AAGGUAAAUUAACUCUU
Ga0255224__1021381/13-113         ............GUGCUG..G.....UG.UC...AGCC.A.AAGGUAAAUUAACUCUU
Ga0255223__1087001/28-107         .............................UC...AGCC.A.AAGGUAAAUUAACUCUU
Ga0127502__11371108/362-252       ..................................ACCU.G.GUAGCACAUUAACUCUC
Ga0222728__1000125/1315-1238      .....................................A.A.CAGGUACAUUAACUGGC
Ga0242654__10000586/1273-1196     .....................................A.A.CAGGCACAUUAACUGGC
Ga0180107__1218358/314-219        ....................................GU.U.GAGGUACAUUAACUGGA
Ga0307482__1020425/440-614        ..............................G...UCAU.C.AUGGCACAUUAACUGGG
Ga0179951__1211246/478-633        ..........................GU.GU...AACA.A.UUAGUAAAUUAACUGCC
Ga0179950__1197742/286-441        ..........................GU.GU...AACA.A.UUAGUAAAUUAACUGCC
Ga0129331__1058147/189-99         ..............................U...GAAC.G.CGGGCACCUUAACUCAC
Ga0265756__102054/286-395         ..................................AAGA.U.GUAGUAAAUUAACUGCU
Ga0222728__1000128/4501-4432      .......................................U.GUGGUAAAUUAACUGGU
Ga0265759__114856/370-262         ............GGUCCA..UAUCA.AU.GA...C.CU.C.UCAGCACAUUAACUGAC
Ga0079039__1551950/237-315        .....................................C.A.GCAGCACAUUAACUUGC
Ga0179943__1170285/153-222        .....................................G.A.GCAGCACAUUAACUGAC
Ga0213850__1119088/23-236         ..............................U...AUGU.A.GUAGCACAUUAACUCAC
Ga0247533__103075/417-305         ........................G.GU.GU...ACCA.U.GUGGUACAUUAACUGGG
Ga0247541__102964/56-168          ........................G.GU.GU...ACCA.U.GUGGUACAUUAACUGGG
Ga0265755__105988/310-386         ....................................AC.U.GUAGUACAUUAACUCGC
Ga0157560__1026167/317-382        .......................................G.CCGGAAGAUUAACUCAC
Ga0115595__1093158/7-137          ............GAAGG.....CGC.UC.UUAAACUUC.U.UGUGCAAAUUAACUGGC
Ga0256352__1101982/155-277        ............GGAGUU..UGAC..UG.CU...GCCA.A.UCGGUACAUUAACUGAC
Ga0256350__1100736/283-405        ....................UGAGC.UG.CC...GCCA.A.UCGGUACAUUAACUGAC
Ga0213850__1360620/298-221        ..................................AUAA.G.CAUGUAAAUUAACUGGC
Ga0081247__1167831/113-35         ..................................GAAU.C.GUAGCACAUUACCCGGC
Ga0127503__10676578/136-67        ....................................GG.C.GCAGCACAUUACCCGGC
Ga0138283__1149501/173-59         ............GCGCG...................GC.C.GCGGCACAUUACCCGGC
Ga0265757__139522/177-70          ....................................CC.A.AUGGCACAUUAACUCCC
Ga0265750__1013321/389-496        ...................................ACC.A.AUGGCACAUUAACUCCC
Ga0316029__116217/368-261         ..................................AUAG.C.GCAGUACAUUAACUCCC
Ga0316047__100016/2681-2574       ..................................AUAG.C.GCAGUACAUUAACUCCC
Ga0184569__112764/943-836         ..................................AUAG.C.GCAGUACAUUAACUCCC
Ga0242652__1131915/134-203        .......................................G.GUGGCACAUUACCCUCC
Ga0307482__1020622/600-404        ..............................G...UCCC.G.GUGGCACAUUAACUGGA
Ga0265756__143657/133-55          ..................................AGGA.A.GUGGUACAUUAACUUGC
Ga0180107__1262812/3136-3066      ....................................GU.A.AUGGCACAUUAACUUCC
Ga0265756__118952/425-318         ...........................U.UA...CACC.A.CGGGAAAAUUACCCGGC
Ga0265757__113703/332-416         ........................C.AU.CU...CGUU.A.ACAGCACAUUAACUUGG
Ga0256332__1243244/48-162         ............CUU.....GACGG.UG.GC...CUAA.G.GGGGUACAUUACCCGAC
Ga0307919__1048575/242-90         ..................................UUAG.G.GCGGCACAUUAACUCUC
Ga0265754__1081367/214-290        ..................................C.CU.G.CUGGUAAAUUAACUUAC
Ga0184597__114231/79-11           .....................................U.A.GAGGAAAAUUAACCAGG
Ga0184582__129853/106-35          ....................................AU.A.GUGGCAUAUUAACCAGA
Ga0265744__136679/344-276         .....................................U.A.GAGGCACAUUAACCAGG
Ga0326631__172366/284-207         .....................................C.G.GCAGCAUAUUAACUGGA
Ga0307921__1021783/160-261        ..............................A...CUCC.C.GCAGCACAUUAACUGGC
Ga0247554__113506/254-161         ..............................A...C.UU.C.AUGGAAAAUUAACCGGA
Ga0179939__1229388/322-392        .....................................U.G.CAGGCACAUUAACUGUC
Ga0184568__100511/100-195         ..................................CACU.GUCGAGCAUAUUAACUGCC
Ga0247525__114510/199-318         ....................................CC.G.GUGGCACAUUAACUGGU
Ga0222728__1008526/24-283         ..................................CAAC.C.UGGGCACAUUAACUGGC
Ga0242654__10278372/461-362       ............AUAGU...................UC.G.UGGGCACCUUAACCUCC
Ga0184570__100371/69-155          ..............................C...UGCC.U.GCGGCACAUUAACUCAC
Ga0184597__120210/233-147         ..............................C...UGCC.U.GCGGCACAUUAACUCAC
Ga0213850__1220553/385-463        .......................................G.GAGGUAAAUUAACUGCC
Ga0265745__1120044/54-129         ....................................GC.G.GCGGCACAUUAACUCCC

                                  ............>>>>>>..>>>>>.>>.>>...>>>>.>.>>>.........>>>>>
                                  ....................................22.2.222.........22222
                                  ------------------------------------○●-●-●●RGYAYAUUAACY●●Y
```

```
Ga0213850_1278242/600-506       ............U..............A.GG...CUGC.C.GCGGUACAUUAACCCAC
Ga0213850_1524759/286-380       ............U..............A.GG...CUGC.C.GCGGUACAUUAACCCAC
Ga0242657_1009512/1576-1466     ....................................CU.G.GUGGUACAUUAACUUCC
Ga0307482_1430195/259-189       ....................................AA.U.GUGGUAAAUUAACCAGC
Ga0184590_129043/450-518        ....................................AA.G.ACGGAAUAUUAACCACC
Ga0184578_132646/9-94           ............GGGGGG..G...............AC.A.GUGGCACAUUAACUGCC
Ga0180107_1040049/1100-1010     ..............................C...UGUC.G.AUGGUACAUUAACUUGC
Ga0307484_103912/620-553        .......................................A.GGAGAACAUUAACUGAC
Ga0307921_1037919/342-457       .................A..UGCUG.UU.CG...AUUU.A.GUGGCACAUUAACCCAC
Ga0307920_113994/654-539        .................A..UGCUG.UU.CG...AUUU.A.GUGGCACAUUAACCCAC
Ga0316040_172648/178-66         .................G..CUCAG.UU.CG...AUUU.A.GUGGCACAUUAACCCAC
Ga0184577_110850/246-358        .................G..CUCAG.UU.CG...AUUU.A.GUGGCACAUUAACCCAC
Ga0307920_113994/130-9          .................U..GCUAG.UU.CG...AUUU.A.GUGGCACAUUAACCCAC
Ga0307919_1081825/191-74        .................G..CUCAG.UU.CG...AUUU.A.GUGGCACAUUAACCCAC
Ga0307921_1095896/178-61        .................G..CUCAG.UU.CG...AUUU.A.GUGGCACAUUAACCCAC
Ga0316031_113250/505-406        ............GUCAUC..UUGCG.AU.GA...C.AU.C.ACGGCACAUUAACUCAC
Ga0265744_118928/137-218        ....................................GG.C.CGGGGAGACUAACUCAC
Ga0265749_106268/131-212        ....................................GG.C.CGGGGAGACUAACUCAC
Ga0157562_1160399/232-315       ..................................AACU.A.CUGGUACAUUAACUUGG
Ga0153880_1231581/116-241       ............GUCCUG..AUUAC.UC.GU...AUUC.A.CGGGUAAAUUAACUCAC
Ga0153880_1191383/455-340       .......................UA.AC.UU...UUUC.A.GUAGUAAAUUAACUUUC
Ga0138282_1242808/325-475       ............GAUG..................GAGC.G.GCGGUACAUUAACUGCC
Ga0242654_10024002/836-915      .....................................A.C.GUGGUACAUUAACUCAC
Ga0242646_1103331/248-318       ....................................AG.A.AUGGCACAUUAACUGGC
Ga0081243_1208438/380-456       .....................................A.A.GUAGCACAUUAACUGGC
Ga0184600_147964/253-339        ..............................G...UACA.U.UUAGUACAUUAACUCAC
Ga0316036_107710/408-322        ..............................G...UACA.U.UUAGUACAUUAACUCAC
Ga0184601_135946/406-321        ..............................G...UACA.U.UUGGUACAUUAACUCAU
Ga0184589_121067/502-587        ..............................G...UACA.U.UUGGUACAUUAACUCAU
Ga0184598_138497/440-355        ..............................G...UACU.U.UUGGUACAUUAACUCAU
Ga0184580_113725/262-335        ..............................G...UACA.U.UUGGUACAUUAACUCAU
Ga0184584_115572/267-354        .....................................A.C.CAAGUACAUUAACUGGA
Ga0247539_100086/1763-1850      .....................................A.C.CAGGUACAUUAACUGGA
Ga0184579_105257/1735-1648      .....................................A.C.CAAGUACAUUAACUGGA
Ga0126318_10229384/609-688      ....................................GU.G.AAGGUACAUUAACUGGC
Ga0307482_1000291/1027-950      ........................G.UA.AA...ACAA.U.GAAGAAAAUUAACUGGC
Ga0316031_119808/254-182        ....................................GU.G.GAGGCACAUUACCCGGU
Ga0307482_1023665/853-778       .....................................C.G.GCAGCACAUUAACUGGU
Ga0180106_1297567/444-515       ..........................................GGGCACAUUAACUGGC
Ga0307484_115660/213-145        .......................................G.GGAGUACAUUAACUGGC
Ga0307484_161007/136-39         .....................................G.G.CGAGAAAAUUAACUGCC
Ga0307484_177982/136-39         .....................................G.G.CGAGAAAAUUAACUGCC
Ga0307482_1033688/516-422       .....................................G.G.CGAGAAAAUUAACUGCC
Ga0265752_111922/418-343        .....................................G.U.GUAGCACAUUAACUGCC
Ga0179939_1199779/67-205        ..............................A...CAUGCU.GAAGCACAUUAACUGGU
Ga0307482_1023050/176-255       ........................A.AG.AU...A.CA.G.GUGGCACAUUAACUGGU
Ga0307482_1202605/470-395       .....................................C.A.CAGGUACAUUAACUGGA
Ga0242657_1476020/160-244       ...........................A.AC...AAGA.A.GUAGCACAUUAACUGGC
Ga0247523_120120/325-403        ....................................GG.U.UGAGCACAUUAACUGGU
Ga0316035_179807/270-184        ..............................G...GAGU.U.GUAGUACAUUAACUGGC
Ga0210323_1050507/418-532       .....................................C.C.GCGGCACAUUAACUGGC
Ga0184571_112742/47-116         .......................................U.GCGGGAAAUUAACUGGC
Ga0247538_100770/928-997        .......................................C.GCGGGAAAUUAACUGGC
Ga0157560_1032840/250-177       .....................................A.A.AGGGUACAUUAACUCCC
Ga0247514_107497/1071-1140      ....................................GA.U.GUGGCAUAUUAACUGGC
Ga0242646_1002014/11-270        ..................................CAAC.G.UGGGCACAUUAACUGGC
Ga0222728_1008391/1281-1023     ..................................CAAC.G.UGGGCACAUUAACUGGC
Ga0242646_1001395/255-509       ...................................CUC.U.CAGGCAUAUUAACUGGC
Ga0307482_1000256/3341-3276     .....................................G.A.UAGGCACAUUAACUGUC
Ga0242647_1006852/526-598       .......................................A.GGUGCACAUUAACUGGC
Ga0184580_111164/678-749        .....................................C.A.UGUGUACAUUAACUGGC
Ga0184600_102725/60-135         .....................................U.G.UGAGCACAUUAACUGGU
Ga0184602_122974/190-265        .....................................U.G.UGAGCACAUUAACUGGU
Ga0256338_1343513/152-228       ....................................CU.G.GUGGUACAUUAACUGGG
Ga0255268_1432700/127-51        ....................................CU.G.GUGGUACAUUAACUGGG
Ga0157560_1147189/148-60        ..................................GACC.G.UGUGAAAAUUAACUGAC
Ga0242654_10672177/244-165      ....................................GU.U.AUGGCACAUUAACUGGC
Ga0242654_10135580/213-336      .......................................G.GUGGUAAAUUAACUAGC
Ga0265742_1000025/2819-2888     .......................................G.CAGGCACAUUAACUGGC
Ga0184599_154888/232-155        ....................................AU.A.GGAGCAAAUUAACUGGU
Ga0228707_1100139/344-194       ............GGG..G..CACAU.AA.GU...CUUG.A.UUGGUACAUUACCCACC
Ga0307919_1084008/308-378       ....................................GG.G.GCAGCACAUUAACUCGC
Ga0307919_1039507/513-440       ....................................GC.G.GCAGCACAUUAACUCGU
Ga0307921_1024041/406-334       ....................................GC.G.GCAGCACAUUAACUCGU
Ga0242657_1015112/947-881       ...................................CAG.A.UCAGGACAUUAACUGGC
Ga0307482_1021658/484-563       ....................................AG.C.AGAGCAUAUUAACUGGC
Ga0307920_133961/233-140        ..................................CUCU.C.GUAGCACAUUAACUUGC
Ga0307921_1019702/90-184        ..................................CUCU.C.GUAGCACAUUAACUUGC
Ga0182092_1031527/199-109       ......................UCU.UC.AG...UCGA.G.GUAGUACAUUAACCCAC
Ga0184575_104772/255-338        ..................................AGGG.U.GUAGUACAUUAACUGGC
Ga0179940_1033563/296-410       ............CAUUC..................CUU.G.GUAGCACAUUAACUUAC
Ga0184596_102602/271-200        .....................................A.U.CGGGCACAUUAACGGCC
Ga0184596_125844/4802-4873      .....................................A.U.CGGGCACAUUAACUGCC
Ga0153880_1058095/344-241       .......................UG.AG.CG...UCGU.U.GUAGCACAUUAUCUGGC
Ga0153880_1167367/224-121       .......................UG.AG.CG...UCGU.U.GUAGCACAUUAACUGGC
Ga0179943_1178349/195-298       ........................G.AG.AG...UCGU.U.GUAGCACAUUAACUGGC
Ga0179944_1313268/129-232       ........................G.AG.CG...UCGU.U.GUAGCACAUUAACUGGC
Ga0213850_1259428/445-342       ........................G.AA.CU...CUGU.U.GUAGCACAUUAACUGGC
Ga0115595_1051440/308-419       .............................AC...ACAU.C.GUAGUACAUUAACUUCC

                                ............>>>>>>..>>>>>.>>.>>...>>>>.>.>>>.........>>>>>
                                ............______.._____.__.__.....22.2.222.........22222
                                ------------------------------------○●-●-●●RGYAYAUUAACY●●Y
```

```
Ga0310106__100998/1608-1690      ..............................G...AAUA.A.CAGGUAAAUUAACUGCU
Ga0307482__1017422/1387-1206     ....................................AU.C.ACGGCACAUUAACUGGG
Ga0307482__1050048/176-95        ....................................GG.A.UGAGCACAUUAACUGGC
Ga0179939__1078463/91-178        ...........................U.GU...GCUG.G.AUAGCACAUUAACUCGA
Ga0247539__107237/153-229        .......................................A.UCGGCAUAUUAACUGGU
Ga0127502__10189886/163-55       ............AGUGCG..AGUUG.AU.CG...GCAC.G.UGGGCACAUUAACUAGC
Ga0265746__1085300/256-329       .....................................C.G.AGGGUACAUUAACUGCC
Ga0242641__1002064/1203-1116     .....................................G.G.AUGGCACAUUAACUGGC
Ga0242657__1090937/459-526       .......................................U.CGGGUACAUUAACUGCC
Ga0180107__1021361/468-373       ....................................CU.U.GGGGCACAUUAACUGGU
Ga0184599__136526/36-116         ..............................A...UGAU.A.UGGGUAAAUUAACUGCA
Ga0316032__127868/210-130        ..............................A...UGAU.A.UGGGCAAAUUAACUGCA
Ga0032354__1062690/2659-2586     .......................................U.GGGGCACAUUAACUGGU
Ga0242657__1027062/255-322       .....................................A.C.GAGGCACAUUAACUGGU
Ga0265747__105373/294-105        .............................GG...CGUA.C.GAGGCACAUUAACUGGU
Ga0265745__1000512/1043-854      .............................GG...CGUA.C.GAGGCACAUUAACUGGU
Ga0265745__1001084/890-701       .............................GG...CGUA.C.GAGGCACAUUAACUGGU
Ga0310117__104105/1058-869       .............................GG...CGUG.C.AAGGCACAUUAACUGGU
Ga0265745__1000697/596-785       ....................................CA.C.GAGGCACAUUAACUGGU
Ga0247551__106718/112-179        .....................................G.U.GCGACAAAUUAACUGUC
Ga0184603__137775/245-174        ....................................AU.C.CAAGAAAAUUAACUGGC
Ga0242641__1105635/265-351       .............................GA...CUCG.G.GUGGCACAUUAACUCAC
Ga0242654__10795375/263-349      .............................GA...CUCG.G.GUGGCACAUUAACUCAC
Ga0157576__1168696/131-64        .....................................G.U.AUGGUACAUUAACUCAC
Ga0255292__1094640/555-357       CAUCCCGCCGGU.........................G.U.AUGGUACAUUAACUCAC
Ga0256349__1073199/279-477       CAUCCCGCCGGU.........................G.U.AUGGUACAUUAACUCAC
Ga0256358__1096062/218-22        CAUCCCGCCGGU.........................G.U.AUGGUACAUUAACUCAC
Ga0256350__1079106/235-433       CAUCCCGCCGGU.........................G.U.AUGGUACAUUAACUCAC
Ga0256352__1080168/251-449       CAUCCCGCCGGU.........................G.U.AUGGUACAUUAACUCAC
Ga0255293__1084149/567-369       CAUCCCGCCGGU.........................G.U.AUGGUACAUUAACUCAC
Ga0256348__1071395/353-551       CAUCCCGCCGGU.........................G.U.AUGGUACAUUAACUCAC
Ga0265758__100178/821-753        ....................................AG.A.UCAGUAUAUUAACUGCC
Ga0157560__1090855/105-186       ...........................G.CG...AUUA.A.GCGGUACAUUAACUCAC
Ga0005851__1006483/274-172       ....................................AA.A.GCAGCACAUUAACUCAC
Ga0184603__107262/477-596        ....................................CC.G.GUGGCACAUUAACUGGU
Ga0184585__151549/495-376        ....................................CC.G.GUGGCAUAUUAACUGGU
Ga0242647__1022053/339-428       ..........................CU.UC...UUCG.C.UGAGUAAAUUAACUGCC
Ga0184599__128731/188-273        ..............................C...UGCU.U.GGGGUACAUUAACCGGA
Ga0310114__149639/68-153         ..............................C...UGCU.U.GGGGUACAUUAACUGGA
Ga0310119__131358/533-447        ............GCUGC...................UU.U.GAGGUACAUUAACUGGA
Ga0079074__1318576/412-571       .............................GC...CCCU.U.AUGGUACAUUAACUGGU
Ga0307484__102285/276-209        .......................................A.CGGGCACAUUAACUGUC
Ga0247534__109145/204-136        .......................................A.CGGGUACAUUAACUGCC
Ga0242657__1013457/154-227       .....................................G.U.CUAGCAUAUUAACUGGC
Ga0242654__10000487/4104-4168    .......................................U.GCGGAAAAUUAACUGGC
Ga0242647__1038796/266-195       .....................................G.G.UAGGAAAAUUAACUGCC
Ga0115594__1156252/268-370       ..............................C...CUCU.G.AGGGCACAUUAACUGGC
Ga0115594__1016490/326-410       ..................................AGCU.G.AGGGCACAUUAACUGGC
Ga0115595__1007417/328-412       ..................................AGCU.G.AGGGCACAUUAACUGGC
Ga0265742__1038020/174-90        .............................AG...UGCA.A.GAAGAAUAUUAACUGGC
Ga0184568__114501/165-82         .............................AG...UGCA.A.GAAGAAUAUUAACUGGC
Ga0265755__113253/366-449        ...........................U.AG...UGCA.A.GAAGAAUAUUAACUGGC
Ga0184600__115185/364-447        .............................AG...UGCA.A.GAAGAAUAUUAACUGGC
Ga0157593__1013623/299-215       ....................................AG.A.GUAGCACAUUAACUCAC
Ga0153880__1374410/101-33        .......................................G.GAGGAAUAUUAACUGAC
Ga0316038__101969/827-896        ...................................UUG.A.AUGGAAAAUUAACUGCC
Ga0316046__130251/204-135        ...................................UCG.A.AUGGAAAAUUAACUGCC
Ga0265758__104499/242-367        .............................GU...GCAC.G.UUAGCACAUUAACUGGC
Ga0307484__108094/186-350        .....................................G.G.AGGGCAUAUUAACUGAC
Ga0307482__1009223/670-508       ..................................GCCA.G.AGGGCAUAUUAACUGAC
Ga0242647__1067563/210-289       .....................................G.G.AUGGCACAUUAACUGGC
Ga0242657__1207391/462-383       ....................................GU.A.AUGGCAUAUUAACUGGC
Ga0265742__1001052/322-80        ....................................CC.G.AUGGCACAUUAACUGGA
Ga0265750__1004028/1262-1504     ....................................CC.G.AUGGCACAUUAACUGGA
Ga0310113__106868/972-730        ....................................CC.G.AUGGCACAUUAACUGGA
Ga0265747__100653/495-253        ....................................CC.G.AUGGCACAUUAACUGGA
Ga0265752__100504/930-688        ....................................CC.G.AUGGCACAUUAACUGGA
Ga0265740__1001378/1084-842      ....................................CC.G.AUGGCACAUUAACUGGA
Ga0265750__1009219/272-30        ....................................CC.G.AUGGCACAUUAACUGGA
Ga0265757__104669/135-376        ....................................CC.G.AUGGCACAUUAACUGGA
Ga0265745__1000618/793-552       ....................................CC.G.AUGGCACAUUAACUGGA
Ga0265742__1088739/51-292        ....................................CC.G.AUGGCACUUUAACUGGA
Ga0265747__100509/1277-1036      ....................................CC.G.AUGGCACAUUAACUGGA
Ga0265752__100314/1466-1225      ....................................CC.G.AUGGCACAUUAACUGGA
Ga0265743__104537/484-553        .......................................G.AGGGCACAUUAACUGGC
Ga0265744__160011/83-18          .......................................U.ACGGUACAUUAACUGGC
Ga0153880__1410217/288-397       ...................................GUG.A.GUAGUACAUUAACUUCC
Ga0115594__1054328/389-276       ........................A.AG.AC...ACAU.C.GUAGUACAUUAACUUCC
Ga0316029__112968/271-337        .......................................G.GUAGUACAUUAACUUGC
Ga0157560__1034839/242-321       ..................................UAAA.A.GUAGUACAUUAACUUGC
Ga0157566__1117351/242-321       ..............................G...UGAA.A.GUAGUACAUUAACUUGC
Ga0157593__1112297/86-12         ....................................AC.A.GUAGUAAAUUAACUUGC
Ga0157565__1020724/148-72        ...................................UAC.A.GUAGUAAAUUAACUUGC
Ga0247519__104852/800-730        .......................................G.UUGGCAUAUUAACUGGC
Ga0307480__1000549/913-1064      ..................................CAUC.G.GUAGCAUAUUAACUGUC
Ga0316031__132099/178-294        .....................................U.G.CGGGUACAUUAACUGGG
Ga0247526__116548/279-354        .....................................A.G.AUGGCAAAUUAACUGUC
Ga0247519__113845/334-409        ....................................CA.G.AUGGCAAAUUAACUGUC
Ga0247529__111070/303-228        ....................................CA.G.AUGGCAAAUUAACUGUC

                                 ............>>>>>>..>>>>>.>>.>>...>>>>.>.>>>.........>>>>>
                                 ....................................22.2.222.........22222
                                 ------------------------------------○●-●-●●RGYAYAUUAACY●●Y
```

```
Ga0184602__101759/1045-970      ....................................CA.G.AUGGCAAAUUAACUGUC
Ga0184600__139292/252-327       ....................................CA.G.AUGGCAAAUUAACUGUC
Ga0184580__108231/296-221       ....................................CA.G.AUGGCAAAUUAACUGUC
Ga0247534__105038/288-211       ....................................CA.G.AUGGCAAAUUAACUGUC
Ga0247552__109074/256-333       ....................................CA.G.AUGGCAAAUUAACUGUC
Ga0247542__100296/1022-1097     .....................................A.G.AUGGCAAAUUAACUGUC
Ga0247545__113720/232-157       .....................................A.G.AUGUCAAAUUAACUGUC
Ga0184597__110829/1090-1011     .....................................G.A.UGGGCACAUUAACUGUC
Ga0184600__105920/81-160        .....................................G.A.UGGGUACAUUAACUGUC
Ga0184578__129447/314-235       .....................................G.A.UGGGCACAUUAACUGUC
Ga0184602__121948/324-245       .....................................G.A.UGGGCACAUUAACUGUC
Ga0184603__125382/9-88          .....................................G.A.UGGGUAUAUUAACUGUC
Ga0247550__100138/208-129       .....................................G.A.UGGGUAUAUUAACUGUC
Ga0316046__122965/221-142       .....................................G.A.UGGGUACAUUAACUGUC
Ga0316051__1003245/138-56       .....................................G.A.UGGGUAUAUUAACUGUC
Ga0316049__122425/209-288       .....................................G.A.UGGGUACAUUAACUGUC
Ga0184588__113246/331-252       .....................................G.A.UGGGUACAUUAACUGUC
Ga0184582__111448/113-237       ....................GGUUC.UU.GC...AACA.U.CGGGUACAUUAACUGGC
Ga0184597__112736/543-466       ....................................CA.U.CGGGUACAUUAACUGGC
Ga0184577__100528/550-473       ....................................CA.U.CGGGUACAUUAACUGGC
Ga0127502__10397722/141-209     .....................................A.U.GGGGCACAUUAACUGCC
Ga0184593__119512/675-608       .......................................U.GGGGCACAUUAACUGUC
Ga0307482__1597388/86-18        .....................................G.U.UGGGUACAUUAACUGUC
Ga0307482__1001973/47-115       .....................................G.U.UGGGUACAUUAACUGUC
Ga0184595__132469/902-822       ...................................AUG.U.AUGGAAAAUUAACUGCC
Ga0079039__1443516/199-38       ..............................U...UCAC.A.GAGGCACAUUAACUGGU
Ga0265756__100130/1310-1239     .......................................A.UUGGCACAUUAACUGGC
Ga0265747__125875/320-395       .....................................G.C.GCAGCACAUUAACUCAC
Ga0180052__189627/240-346       ...................................AGC.A.UCGGUACAUUAACUCGC
Ga0180044__1074338/112-33       .............................CU...AUUA.A.GUGGUACAUUAACUCAC
Ga0157559__1047848/128-41       ....................................AC.A.GUGGUACAUUAACUGGC
Ga0157562__1134247/69-160       ..................................GCGC.A.GUAGCACAUUAACUGGC
Ga0247548__101188/825-888       .......................................A.GCGGUACAUUAACUCAC
Ga0228707__1093045/293-207      ......................CAU.CG.UU...CACC.G.GCAGCACAUUAACUGGA
Ga0184597__119383/488-559       ....................................AG.U.CUAGUAAAUUAACUGGU
Ga0184602__121040/35-106        ....................................AG.U.CUAGUAAAUUAACUGGU
Ga0326631__107633/416-489       ....................................AG.U.CUAGUAAAUUAACUGGU
Ga0316046__149266/91-162        ....................................AG.U.CUAGUAAAUUAACUGGU
Ga0247538__100917/537-466       ....................................AG.U.CUAGUAAAUUAACUGGC
Ga0247519__118271/334-405       ....................................AG.U.CUAGUAAAUUAACUGGU
Ga0265747__100454/1395-1324     .....................................G.G.AUAGAAAAUUAACUGGU
Ga0316046__100502/370-442       .....................................G.G.AUAGUAAAUUAACUGGU
Ga0247551__101484/617-543       .....................................G.G.AUAGUAAAUUAACUGGU
Ga0213850__1280121/544-351      ........................G.AU.CG...CACA.C.UCGGCACAUUAACCAGC
Ga0265759__100017/4211-4288     .....................................G.A.UAGGAAAAUUAACUGGC
Ga0307482__1127544/505-398      ........................A.CC.CA...GCUG.G.AGGGCACAUUAACUGGC
Ga0180107__1057591/210-107      .....................................A.G.UGAGCAAAUUAACUGCC
Ga0157569__1061476/44-119       ...........................G.UG...CUUU.G.ACAGCACAUUAACUACC
Ga0307484__100889/412-594       ....................................AU.C.ACGGCACAUUAACUGGG
Ga0310106__100022/563-489       .............................AG...UGUA.U.GUGGCACAUUAACUGGU
Ga0310115__100154/4483-4559     .............................AG...UGUA.U.GUGGCACAUUAACUGGU
Ga0180107__1262812/2765-2832    ....................................UA.U.GUGGUACAUUAACUGGU
Ga0184597__110025/865-793       ....................................UA.U.GUGGGAAAUUAACUGAG
Ga0247550__100495/487-560       ....................................UA.U.GUGGGAAAUUAACUGAG
Ga0184582__135749/62-135        ....................................UA.U.GUGGGAAAUUAACUGAG
Ga0184603__122688/908-835       ....................................UA.U.GUGGGAAAUUAACUGAG
Ga0247524__126530/125-198       ....................................AA.U.GUGGGAAAUUAACUGAG
Ga0316030__115438/368-435       .....................................A.C.CAGGCACAUUAACUGGC
Ga0307354__155926/276-116       ..............................U...CUCU.U.GUGGCACAUUAACUGGC
Ga0079074__1279626/114-274      ..............................U...CUCU.U.GUGGCACAUUAACUGGC
Ga0184602__132007/314-246       ....................................GU.C.GUGGUACAUUAACUGGC
Ga0307921__1005950/791-877      ..............................U...GACA.C.CUGGCACAUUAACUUGC
Ga0242654__10000487/4544-4475   .....................................C.C.AUGGCACAUUAACUCCC
Ga0157569__1032488/120-205      ....................................AG.G.CUGGUACAUUACCCGGC
Ga0157593__1125711/213-128      ...................................UGU.G.CUGGCACAUUACCCGGC
Ga0157565__1077069/156-67       ..................................GCAC.A.ACGGUACAUUACCCGGC
Ga0180047__1128081/151-64       ...................................UGU.G.CUGGCACAUUACCCGGC
Ga0265756__135986/152-290       ............GACAGG..ACAUA.AA.UA...CAGU.G.GUGGCACAUUAACUGGC
Ga0180045__119797/166-305       ..............................U...CCAU.A.GAGGCACAUUAACUGGC
Ga0115595__1089740/408-494      ..........................CA.GA...CGUC.A.GUGGUACAUUAACUGCG
Ga0179955__1048341/123-51       .......................................C.GUGGUACAUUAACUGGC
Ga0138280__1140856/341-479      ..................................GAGC.G.GCGGCACAUUAACUGGC
Ga0180106__1302226/203-273      .....................................G.G.CUGGUACAUUAACUGGC
Ga0310119__169621/253-177       .....................................G.U.CUGGUACAUUAACUGGC
Ga0138277__1023917/182-18       ............UUGACU..GGCUC.G.......GAGC.G.GUGGUACAUUAACUGGC
Ga0138282__1020546/737-573      ............UUGACU..GGCUC.G.......GAGC.A.GUAGUACAUUAACUGGC
Ga0179939__1059820/107-216      ..............................G...UGGU.A.GUAGCAUAUUAACUGGC
Ga0179950__1098211/345-278      .......................................G.GUGGUACAUUAACUGGC
Ga0115594__1068468/127-56       .......................................C.GUGGUACAUUAACUGGC
Ga0228708__1103570/188-285      ...........................C.CU...AG.A.C.GUGGCACAUUAACUGGC
Ga0265751__160438/108-215       ..................................GGGG.C.GCGGCACAUUAACUUGC
Ga0242647__1000066/2638-2719    .....................................U.G.UGGGCACAUUAACUGGC
Ga0242646__1001227/1243-1308    ....................................CU.U.UGGGUACAUUAACUGGC
Ga0182091__1104347/98-194       ............A................AC...GCGC.U.GUAGCACAUUAACUUGC
Ga0115594__1178636/111-46       ....................................AG.U.GUAGUACAUUAACUUGC
Ga0213850__1508869/496-402      ...........................A.AU...CUGG.U.GUAGCAUAUUAACUUGC
Ga0307482__1152465/114-38       ............UAAAC...................AA.U.GAAGAAAAUUAACUGGC
Ga0180107__1031754/139-57       ..................................UCAG.U.GUAGCAUAUUAACUGGC
Ga0316037__121702/176-240       .....................................G.U.GGAGCAAAUUAACUGGC

                                ............>>>>>>..>>>>>.>>.>>...>>>>.>.>>>.........>>>>>
                                ....................................22.2.222.........22222
                                ------------------------------------○●-●-●●RGYAYAUUAACY●●Y
```

```
Ga0265759_107962/103-39        .....................................G.U.GGAGCACAUUAACUGGC
Ga0242649_1051170/368-302      ....................................AG.A.GUGGCACAUUAACUGGU
Ga0307482_1064439/84-153       ..................................UCAA.U.GUGGCACAUUAACUGGC
Ga0115594_1006122/120-29       ..............................A...GCUG.A.AGGGCACAUUAACUGGC
Ga0115595_1180708/226-317      ..............................G...GCUG.A.AGGGCACAUUAACUGGC
Ga0265746_1064626/389-458      .....................................G.A.GCGGAAUAUUAACUGGC
Ga0265759_108120/277-351       .....................................C.G.CGGGCACAUUAACUGGC
Ga0307482_1000450/2015-2077    .....................................A.A.CGGGCAAAUUAACUGGC
Ga0247527_115808/280-440       .................G..GCCAG.UG.GG...UGCU.A.CGGGCACAUUAACUGGG
Ga0184588_120624/266-426       .................G..GCCAG.UG.GG...UGCU.A.CAGGCACAUUAACUGGG
Ga0184579_106194/996-1156      .................G..GCCAG.UG.GG...UGCU.A.CAGGCACAUUAACUGGG
Ga0265758_100254/356-516       .................G..GCCAG.UG.GG...UGCU.A.CGGGCACAUUAACUGGG
Ga0316035_107544/354-514       .................G..GCCAG.UG.GG...UGCU.A.CGGGCACAUUAACUGGG
Ga0184596_109261/979-1139      .................G..GCCGG.UG.GG...UGCU.A.CGGGCACAUUAACUGGG
Ga0184600_132066/568-408       .................G..GCCAG.UG.GG...UGCU.A.CGGGCACAUUAACUGGG
Ga0184597_106861/196-36        .................G..GCCAG.UG.GG...UGCU.A.CGGGCACAUUAACUGGG
Ga0316031_108320/216-376       .................G..GCCAG.UG.GG...UGCU.A.CGGGCACAUUAACUGGG
Ga0184589_110648/1041-881      .................G..GCCGG.UG.GG...UGCU.A.CGGGCACAUUAACUGGG
Ga0184580_124121/239-79        .................G..GCCGG.UG.GG...UGCU.A.CGGGCACAUUAACUGGG
Ga0184598_101324/530-690       .................G..GCCGG.UG.GG...UGCU.A.CGGGCACAUUAACUGGG
Ga0247547_102464/189-29        .................A..GCCCA.AG.GG...UGAU.G.GAGGCACAUUAACUGGA
Ga0247551_100828/546-387       .................G..GCCCA.UG.GG...UGAU.G.AAGGCACAUUAACUGGA
Ga0247536_101914/360-201       .................G..GCCCA.UG.GG...UGAU.G.AAGGCACAUUAACUGGA
Ga0247538_100480/788-947       .................G..GCCCA.UG.GG...UGAU.G.AAGGCACAUUAACUGGA
Ga0247550_100850/456-297       .................G..GCCCA.UG.GG...UGAU.G.AAGGCACAUUAACUGGA
Ga0247553_110698/526-367       .................G..GCCCA.UG.GG...UGAU.G.AAGGCACAUUAACUGGA
Ga0184589_112135/40-200        ....................AGCCC.AG.GG...UGAC.A.GAGGCACAUUAACUGGC
Ga0247526_110221/603-443       .................A..GCCCA.AG.GG...UGAU.G.GAGGCACAUUAACUGGA
Ga0247541_103784/123-283       .................A..GCCCA.AG.GG...UGAU.G.GAGGCACAUUAACUGGA
Ga0247545_112960/183-343       .................A..GCCCA.AG.GG...UGAC.A.GAGGCACAUUAACUGGA
Ga0184586_133850/368-528       .................A..GCCCA.UG.GG...UGAU.G.GAGGCACAUUAACUGGA
Ga0247552_101742/612-452       .................A..GCCCA.AG.GG...UGAU.G.GAGGCACAUUAACUGGA
Ga0247545_104341/406-246       .................A..GCCCA.AG.GG...UGAU.G.GAGGCACAUUAACUGGA
Ga0184602_114416/11-171        .................A..GCCCA.AG.GG...UGAU.G.GAGGCACAUUAACUGGA
Ga0316038_111670/11-171        .................A..GCCCA.AG.GG...UGAU.G.GAGGCACAUUAACUGGA
Ga0247529_134913/224-64        .................A..GUCCA.AG.GG...UGAU.G.GAGGCACAUUAACUGGA
Ga0184603_143085/253-93        .................G..GCCCA.AG.GG...UGAU.G.GAGGCACAUUAACUGGA
Ga0316032_107575/215-55        .................A..GCCCA.AG.GG...UGAU.G.GAGGCACAUUAACUGGA
Ga0247519_110701/607-447       .................A..GCCCA.AG.GG...UGAU.G.GAGGCACAUUAACUGGA
Ga0247524_111427/50-210        .................A..GCCCA.AG.GG...UGAU.G.GAGGCACAUUAACUGGA
Ga0184574_121290/269-109       .................A..GCCCA.AG.GG...UGAU.G.GAGGCACAUUAACUGGA
Ga0184595_111222/3-163         .................A..GCCCA.AG.GG...UGAU.G.GAGGCACAUUAACUGGA
Ga0184600_123882/188-28        .................A..GCCCA.AG.GG...UGAU.G.GAGGCACAUUAACUGGA
Ga0326631_101563/544-705       .................A..GCCCA.AG.GG...UGAU.G.GAGGCACAUUAACUGGA
Ga0184573_110190/115-276       .................A..GUCCA.AG.GG...UGAU.G.GAGGCACAUUAACUGGA
Ga0184596_111007/525-686       .................A..GCCCA.AG.GG...UGAU.G.GAGGCACAUUAACUGGA
Ga0316033_144939/167-328       .................A..GCCCA.AG.GG...UGAU.G.GAGGCACAUUAACUGGA
Ga0247526_117235/152-313       .................A..GCCCA.AG.GG...UGAC.A.GAGGCACAUUAACUGGA
Ga0247551_100195/490-329       .................A..GCCCA.AG.GG...UGAC.A.GAGGCACAUUAACUGGA
Ga0247538_100424/120-281       ....................AGCCC.AG.GG...UGAU.G.GAGGCACAUUAACUGGA
Ga0247550_114736/167-7         .................U..AGCCC.AG.GG...UGAU.G.GAGGCACAUUAACUGGA
Ga0247529_103231/270-430       .................U..AGCCC.AG.GG...UGAU.G.GAGGCACAUUAACUGGA
Ga0184603_111918/241-82        .................U..AGCCC.AG.GG...UGAU.G.GAGGCACAUUAACUGGA
Ga0184603_126764/80-240        .................U..AGCCC.AG.GG...UGAU.G.GAGGCACAUUAACUGGA
Ga0247519_107918/217-58        .................U..AGCCC.AG.GG...UGAU.G.GAGGCACAUUAACUGGA
Ga0184580_114961/306-146       .................U..AGCCC.AG.GG...UGAU.G.GAGGCACAUUAACUGGA
Ga0213850_1042834/351-428      ....................................GG.A.GAAGCAUAUUAACUGAC
Ga0265756_103656/97-163        .......................................G.UGGGCACAUUAACUGAC
Ga0265756_101189/896-833       .....................................G.A.GGGGCACAUUAACUGAC
Ga0180107_1033011/414-478      .....................................A.G.CGGGCACAUUAACUGGC
Ga0115594_1157594/155-254      ..............................A...GCUG.A.AGGGCACAUUAACUGGC
Ga0115594_1166236/119-19       ..............................A...GCUG.A.AGGGCACAUUAACUGGC
Ga0115594_1007070/120-30       ..............................A...GCUC.A.AGGGCACAUUAACUGGC
Ga0115595_1181384/176-86       ..............................G...GCUC.A.AGGGCACAUUAACUGGC
Ga0115594_1211214/299-389      ..............................A...GCCA.A.AGGGCACAUUAACUGGC
Ga0115594_1182529/183-84       ..............................G...GCUG.A.AGGGCACAUUAACUGGC
Ga0256352_1182139/180-81       ..............................A...GCUG.A.AGGGCACAUUAACUGGC
Ga0179944_1005311/261-382      ...............GG.....GGG.AU.GA...CAGC.G.CCGGUACAUUAACUCGC
Ga0213850_1508120/373-299      ....................................AG.A.UCGGCACAUUAACAUGC
Ga0307484_162891/325-258       ....................................GA.G.AUAGCACAUUAACUGGC
Ga0184596_111635/86-148        .......................................G.ACGGCACAUUAACUUGC
Ga0316051_1005111/447-509      .......................................G.ACGGCACAUUAACUUGC
Ga0265753_1379003/85-148       .......................................G.ACGGCACAUUAACUUGC
Ga0184603_116647/206-269       .......................................G.ACGGCACAUUAACUUGC
Ga0184587_132427/565-485       ...................................GGG.G.GUGGCACAUUAACUGGC
Ga0184599_110049/275-349       .......................................G.GUGGCACAUUAACUGGC
Ga0184603_148109/996-920       .....................................G.G.GUGGCACAUUAACUGGC
Ga0310114_133004/56-132        .....................................G.G.GUGGCACAUUAACUGGC
Ga0310119_113172/381-306       .....................................G.G.GUGGCACAUUAACUGGC
Ga0265747_119090/8-84          .......................................G.GCAGCACAUUAACUUGC
Ga0247551_122551/29-115        .....................................U.G.AAGGCAAAUUAACUGGC
Ga0316048_107802/239-324       .....................................U.G.AAGGCAAAUUAACUGGC
Ga0242657_1208314/452-382      .....................................A.G.GAGGCAAAUUAACUGGC
Ga0184596_129213/83-168        ............GUGUG...................CU.C.UAGGAAAAUUAACCGUC
Ga0184603_146917/441-510       .....................................G.C.UAGGAAAAUUAACCGUC
Ga0247550_102601/160-89        .....................................G.C.UAGGAAAAUUAACCGUC
Ga0184602_110024/263-177       ............UAAGC....................G.C.UAGGAAAAUUAACCGUC
Ga0184597_116115/700-787       ............GUAAA...................UG.C.UAGGAAAAUUAACCGUC
Ga0184582_128596/700-786       ............GUAAG...................UG.C.UAGGAAAAUUAACCGUC

                               ............>>>>>>..>>>>>.>>.>>...>>>>.>.>>>.........>>>>>
                               ............______..._____.__.__.....22.2.222.........22222
                               ------------------------------------○•-•-••RGYAYAUUAACY••Y
```

```
Ga0184597__102019/330-402          ....................................G.C.UAGGAAAAUUAACCGUC
Ga0242649__1000320/700-631         ......................................U.CCGGCACAUUAACUGGC
Ga0179943__1102267/215-90          ...........GG...G..GGAUG.AU.U....GCGA.C.UCGGUAAAUUAACUGGC
Ga0179944__1191552/284-411         ...........GG...G..GGAUG.AU.CG...CGAC.U.ACGGUACAUUAACUGGC
Ga0179943__1150468/216-89          .............GG.G..GGAUG.AU.CG...CGAC.U.GCGGUAAAUUAACUGGC
Ga0179944__1204933/216-89          .............GG.G..GGAUG.AU.CG...CGAC.U.GCGGUAAAUUAACUGGC
Ga0265758__104558/436-365          ....................................U.G.GUGGUAAAUUAACUGGC
Ga0180107__1040315/471-547         .................................GAAU.G.UUGGCACAUUAACUGGC
Ga0180106__1332058/4386-4449       ....................................G.A.UGGGCAUAUUAACUGAC
Ga0307484__104189/90-171           ....................................A.G.GUGGAAAAUUAACUGCC
Ga0265752__100023/1212-1290        .............................G...UUAC.U.AUGGAAAAUUAACUGGU
Ga0157593__1101573/285-478         ...................AGGAA.CC.UU...GAUA.C.AUGGUACAUUAACUGGC
Ga0307482__1008742/1212-1288       ....................................G.A.UAGGCACAUUAACUGGC
Ga0247551__104770/284-362          .............................G...AAGA.G.UGGGCACAUUAACUGCC
Ga0184593__112903/290-212          .............................G...AAGA.G.UGGGCACAUUAACUGCC
Ga0307482__1128390/271-339         ......................................G.UGGGCAAAUUAACUGCC
Ga0184580__126177/800-732          ....................................U.G.GGAGCAAAUUAACUGCC
Ga0242646__1115622/191-109         ....................................U.G.CGAGCAAAUUAACUGCC
JGI12272J11983__1319142/230-159    ...................................GU.A.UGAGCACAUUAACUGCC
Ga0184596__105062/163-229          ......................................G.AGGGUACAUUAACUGCC
Ga0307482__1011891/77-10           ......................................G.AGGGUACAUUAACUGCC
Ga0184588__125064/416-488          ....................................A.G.UGUGCACAUUAACUGCC
Ga0184577__107529/254-327          ....................................A.G.UGUGCACAUUAACUGCC
Ga0180106__1182034/403-337         ......................................G.UGGGCACAUUAACUGCC
Ga0180107__1241189/1554-1620       ......................................G.UGGGCACAUUAACUGCC
Ga0222728__1000110/4754-4821       ......................................G.UGGGCACAUUAACUGCU
Ga0307484__100154/562-629          ......................................G.UGGGCACAUUAACUGCU
Ga0180107__1285439/94-155          ......................................G.GUAGUAAAUUAACUGUC
Ga0184580__116530/249-177          ....................................A.G.CGAGUAUAUUAACUGUC
Ga0247526__117664/434-499          ......................................A.GUGGGAAAUUAACUCGC
Ga0184580__120219/79-4             ...................................UA.A.AUGGAAAAUUAACUGGC
Ga0247539__100482/682-612          ...................................UA.U.GCAGCAUAUUAACUCGC
Ga0247516__124175/202-272          ...................................UA.U.GCAGCAUAUUAACUCGC
Ga0184580__121247/966-1036         ...................................UA.U.GCAGCAUAUUAACUCGC
Ga0265743__100411/1096-1162        ........................................GCAGUAAAUUAACUCGC
Ga0242657__1070636/108-176         ....................................G.A.ACGGAAAAUUAACUGGA
Ga0242657__1122750/95-162          ....................................G.A.AUGGAAAAUUAACUGGA
Ga0307482__1405960/253-322         ....................................G.A.UUGGGACAUUAACUGGU
Ga0316049__154968/227-319          ....................................A.G.GUAGAAAAUUAACUGGC
Ga0127502__10852756/278-345        ....................................G.A.UUGGAAAAUUAACUGGC
Ga0247534__100003/3657-3582        .............................A...UCAU.A.AUGGAAAAUUAACUGGC
Ga0180106__1204848/284-221         ......................................U.UCGGAAAAUUAACUGGC
Ga0184585__128240/467-390          ....................................U.C.UCGGAAAAUUAACUGGC
Ga0180106__1180558/1549-1486       ......................................C.UCAGAAAAUUAACUGGC
Ga0265755__100134/298-372          ...................................UA.G.AUGGAAAAUUAACUGGC
Ga0247534__100772/309-234          ....................................A.G.AUGGCAAAUUAACUGGC
Ga0307482__1202063/402-466         ....................................G.G.GCGGAAUAUUAACUGGC
Ga0247514__114593/280-211          ....................................G.G.AUGGAAAAUUAACUGGC
Ga0310101__157764/124-55           ....................................G.G.GUGGGAAAUUAACUGGC
Ga0265745__1000136/129-60          ....................................G.G.GUGGGAAAUUAACUGGC
Ga0265745__1013560/201-270         ....................................G.G.GUGGGAAAUUAACUGGC
Ga0307482__1779657/200-270         ...................................UA.G.AUGGCACAUUAACUGGA
Ga0307482__1044183/658-591         ...................................UA.G.AUGGCACAUUAACUGGA
Ga0265758__100017/4376-4309        ......................................C.CUGGUACAUUAACUGAC
Ga0242654__10617983/312-237        ..................................UGU.G.GUGGAAAAUUAACUGGC
Ga0222728__1054736/119-193         ......................................U.CCAGAAAAUUAACUGGC
Ga0184583__106441/296-227          ....................................G.G.AUGGGACAUUAACUGGC
Ga0247520__104849/891-821          ....................................G.A.UUGGCACAUUAACUGGC
Ga0247518__137824/301-231          ....................................G.A.UUGGCACAUUAACUGGC
Ga0247531__104861/1082-1012        ....................................G.A.UUGGCACAUUAACUGGC
Ga0265746__1040401/190-123         ...................................UU.G.CUAGUAAAUUAACUGGC
Ga0184603__139760/199-128          ...................................AU.C.AUGGAAAAUUAACUGGU
Ga0184603__138085/381-311          ...................................AU.C.AUGGAAAAUUAACUGGU
Ga0265747__102820/448-376          ...................................AU.C.AUGGAAAAUUAACUGGU
Ga0247554__105388/388-316          ...................................GU.C.AUGGAAAAUUAACUGGU
Ga0316037__123461/289-368          ...................................AU.C.AUGGAAAAUUAACUGGU
Ga0184597__121517/262-183          ...................................GU.A.UUGGGAAAUUAACUGGC
Ga0247551__100118/653-723          ...................................GU.A.UUGGGACAUUAACUGGC
Ga0265750__1365815/134-55          ...................................GU.A.UUGGGAAAUUAACUGGC
Ga0184589__120335/11-90            ...................................GU.A.UUGGGAAAUUAACUGGC
Ga0247521__124040/388-309          ...................................GU.A.UUGGGACAUUAACUGGC
Ga0247518__108423/758-837          ...................................GU.A.UUGGGAAAUUAACUGGC
Ga0247531__104608/1256-1177        ...................................GU.A.UUGGGACAUUAACUGGC
Ga0247543__100863/642-721          ...................................GU.A.UUGGGAAAUUAACUGGC
Ga0307482__1026216/884-813         ......................................G.AUGGAAAAUUAACUGGC
Ga0307482__1636410/270-341         ......................................G.AUGGAAAAUUAACUGGC
Ga0310119__128165/432-365          ....................................U.G.UUGGAAAAUUAACUGGC
Ga0307482__1016877/168-97          ......................................G.AUGGAAAAUUAACUGGC
Ga0126318__10746885/3334-3265      ...................................AA.G.AUGGAAAAUUAACUGGC
Ga0206353__11117566/971-1032       ......................................G.CUGGAAAAUUAACUGGC
Ga0180107__1106188/424-495         ......................................G.AUGGAAAAUUAACUGGC
Ga0307482__1000354/2063-1987       ....................................U.G.AAGGAAAAUUAACUGGC
Ga0180107__1132096/355-287         ....................................U.A.UAGGAAAAUUAACUGGC
Ga0180108__1238667/303-371         ....................................U.A.UAGGAAAAUUAACUGGC
Ga0265751__100981/715-788          ...................................AU.A.UAGGAAAAUUAACUGGC
Ga0265756__100898/263-336          ...................................AU.A.UAGGAAAAUUAACUGGC
Ga0307484__102094/153-80           ...................................GG.G.UCGGCAAAUUAACUGGC
Ga0307482__1023270/273-197         ....................................G.A.UCGGUACAUUAACUGGC
Ga0184596__102741/625-701          ....................................G.A.UCGGUACAUUAACUGGU

                                   ...........>>>>>>..>>>>>.>>.>>...>>>>.>.>>>.........>>>>>
                                   ...................................22.2.222.........22222
                                   ---------------------------------○●-●-●●RGYAYAUUAACY●●Y
```

```
Ga0316033__103637/453-391      .....................................G.A.UCGGAAAAUUAACUGGU
Ga0180107__1069127/170-106     .......................................G.AUGGAAAAUUAACUGCC
Ga0247535__105396/277-348      .......................................A.CUGGAAAAUUAACUGCC
Ga0184576__110410/277-348      .......................................A.CUGGAAAAUUAACUGCC
Ga0307482__1219330/19-95       .......................................A.CAGGCACAUUAACUGGC
Ga0247534__116177/42-105       .......................................G.UUGGAAAAUUAACUGGC
Ga0316033__123149/165-228      .......................................G.UUGGAAAAUUAACUGGC
Ga0265751__100560/642-572      ....................................UC.U.UGGGAAAAUUAACUGGC
Ga0307482__1011142/698-628     ....................................CA.G.GAAGCAUAUUAACUGGC
Ga0307482__1465843/259-190     .....................................A.G.GAAGCAUAUUAACUGGC
Ga0184593__115334/333-240      ....................................AA.G.GUAGAAAAUUAACUGGC
Ga0265751__100089/3005-3069    ....................................GU.G.UGAGAAUAUUAACUGGC
Ga0307482__1000325/380-315     .....................................A.G.UGAGAAUAUUAACUGGC
Ga0222729__1014021/463-392     ..................................UGAU.A.AUGGCAUAUUAACUGGU
Ga0307482__1033651/424-494     ....................................CU.A.CAGGAAAAUUAACUGCC
Ga0307482__1015330/1021-1093   .......................................G.GUGGCACAUUAACUGGC
Ga0184570__111551/35-97        .......................................U.GCGGAAUAUUAACUGAC
Ga0184576__117861/362-299      .......................................A.GCGGAAAAUUAACUGAC
Ga0307482__1589279/351-280     .....................................C.U.GAGGAAAAUUAACUGCC
Ga0184586__104419/174-104      .....................................C.U.GAGGGAAAUUAACUGCC
Ga0222728__1148620/185-250     .......................................U.GAGGUAAAUUAACUGCC
Ga0316049__103217/889-823      .......................................U.GAGGAAAAUUAACUGCC
Ga0316034__104024/504-439      .......................................U.GAGGAAAAUUAACUGCC
Ga0184595__117496/296-230      .......................................U.GAGGAAAAUUAACUGCC
Ga0184597__109849/897-963      .......................................U.GAGGAAAAUUAACUGCC
Ga0184588__134146/1219-1285    .......................................U.GAGGAAAAUUAACUGCC
Ga0184584__115247/186-121      .......................................U.GAGGAAAAUUAACUGCC
Ga0184596__126546/83-162       .....................................A.G.UGGGAAGAUUAACUGGC
Ga0184570__116436/278-198      .....................................A.G.UGGGAAAAUUAACUGGC
Ga0184597__111165/262-182      .....................................A.G.UGGGAAAAUUAACUGGC
Ga0316037__169124/279-199      .....................................A.G.UGGGAAGAUUAACUGGC
Ga0247551__103383/322-242      .....................................A.G.UGGGAAAAUUAACUGGC
Ga0247543__103170/244-164      .....................................A.G.UGGGAAAAUUAACUGGC
Ga0184589__120451/249-329      .....................................A.G.UGGGAAUAUUAACUGGC
Ga0184600__118565/177-97       .....................................A.G.UGGGAAGAUUAACUGGC
Ga0316034__100493/254-174      .....................................A.G.UGGGAAAAUUAACUGGC
Ga0184576__117703/380-460      .....................................A.G.UGGGAAAAUUAACUGGC
Ga0184597__129399/366-446      .....................................A.G.UGGGCAAAUUAACUGGC
Ga0184590__111002/143-223      .....................................A.G.UGAGUAAAUUAACUGGC
Ga0184587__105985/71-151       .....................................A.G.UGGGAAGAUUAACUGGC
Ga0184602__122864/595-515      .....................................A.G.UGGGAAGAUUAACUGGC
Ga0184593__101086/682-762      .....................................A.G.UGGGAAGAUUAACUGGC
Ga0247519__108375/286-206      .....................................A.G.UGGGAAGAUUAACUGGC
Ga0127502__10351452/615-550    .......................................G.UGGGCACAUUAACUGGC
Ga0316038__112263/442-356      ....................................UA.U.UGGGCACAUUAACUGGC
Ga0242657__1073895/573-672     ....................C.AGU.AG.UU...UCGU.C.UGAGUAAAUUAACUGGA
Ga0184587__107783/167-95       .....................................U.G.AUGGCACAUUAACUGGC
Ga0265758__101918/310-243      .....................................A.G.ACGGCAUAUUAACUGGC
Ga0307482__1029599/655-579     ....................................UU.G.AAGGCACAUUAACUGGC
Ga0265746__1009702/779-848     .......................................G.AUGGAAUAUUAACUUGC
Ga0184583__113025/102-26       .....................................A.G.UGAGAACAUUAACCAUC
Ga0184580__109961/270-194      .....................................A.G.UGAGAACAUUAACCAUC
Ga0316049__111814/256-181      .....................................A.G.UGAGAACAUUAACCAUC
Ga0247553__120631/200-275      .....................................A.G.UGAGUAUAUUAACCAUC
Ga0184593__119849/313-238      .....................................A.G.UGAGAACAUUAACCAUC
Ga0184599__140898/774-849      .....................................A.G.UGAGAAUAUUAACCAUC
Ga0242647__1005188/439-360     ..................................ACAU.A.UUGGAAAAUUAACUGGC
Ga0242657__1054283/541-620     ..................................ACAU.A.UUGGAAAAUUAACUGGC
Ga0184597__102100/600-669      ....................................GC.A.GUGGCAAAUUAACUGGC
Ga0213850__1460186/227-149     .....................................A.U.CUGGUAAAUUAACUGUC
Ga0242654__10040657/1018-942   .....................................G.A.UGGGUACAUUAACUGGC
Ga0307484__100842/1194-1271    .....................................G.A.UAGGCACAUUAACUGGC
Ga0242654__10401151/84-171     ...........................U.CG...CUUU.G.AAAGCACAUUAACUGAC
Ga0310119__105137/743-673      .....................................G.A.GUAGUAAAUUAACCAGC
Ga0247541__104364/333-188      ............A................GU...UCGU.A.GUAGCACAUUAACUUAC
Ga0184582__129125/749-675      ....................................GU.A.GUAGCACAUUAACUGGC
Ga0265753__1022489/457-531     ....................................GU.A.GUAGCAUAUUAACUGGC
Ga0265751__121301/189-266      .....................................C.C.AUGGUACAUUAACUGGC
Ga0247526__105246/689-508      ..................................UCUA.A.GUGGCACAUUAACUGGA
Ga0247515__107373/548-731      ..................................CUUA.A.GUGGCACAUUAACUGGA
Ga0247538__100442/1363-1181    ..................................UCUA.A.GUGGCACAUUAACUGGA
Ga0247538__100551/721-542      ..................................UCUA.A.GUGGCACAUUAACUGGA
                               ............>>>>>>..>>>>>.>>.>>...>>>>.>.>>>.........>>>>>
                               ....................................22.2.222.........22222
                               ------------------------------------○●-●-●●RGYAYAUUAACY●●Y
```

6 hairpin-second-candidate

For a description of this alignment, see Supplementary Table 2

6.1 Multiple-sequence alignment

Each ribozyme in this alignment is identified by its genomic location in the form SEQID/START-END. SEQID (the sequence accession) is derived from sources such as IMG/M or GenBank. START is the coordinate of the 5′ nucleotide of the hairpin ribozyme, and END corresponds to the 3′ nucleotide. If START>END, then the ribozyme is on the reverse complement strand.

Nucleotides proposed to basepair as part of the consensus structure are shaded in color when they comprise Watson-Crick or G-U pairs. Otherwise they are shaded gray. Conserved stems are also indicated at the bottom of the alignment by angle brackets, where matching $<$ and $>$ denote base-paired columns. Below these angle brackets, the symbol "2" denotes base pairs exhibiting covariation according to the statistically well-founded R-scape method. "1" denotes base pairs exhibiting covariation according to R2R's simplistic method. "0" denotes base pairs that are not observed to mutate and "?" denotes base pairs that have a significant frequency of non-canonical nucleotides for Watson-Crick or G-U pairs ($>$ 5%). Below these base pair annotation is the consensus sequence: "R" = "A" or "G", "Y" = "C" or "U", red nucleotides: nucleotide identity conserved more than 97% of the time, black nucleotides: 90%, gray nucleotides: 75%, red circle (⊙): nucleotide is present 97% of the time, black circle (⊙): 90%, gray circle (⊙): 75%, white circle (⊙): 50%. All percentages of sequences just described (e.g. 97% conserved) assume that sequences have been weighted by the GSC algorithm implemented by the Infernal software package.

The alignment begins on the next page.

20 duplicate sequences are not shown in the alignment

alignment positions 1 · · · 150

```
Ga0184594_129452/258-513     UAAACGGAU.AUCGUAAUCCGGUGCUGUGUGUGGUA.CCUGGUU.........UGGAUACCAGAGAGGUUAG.GAGACAGCUUCUAAAGA...........UUUAUCUU.GGCGAGGGGAA....ACCCAGGGUAGCUCCCAUCUGAGU.
Ga0247524_115310/154-451     UAAACGGAU.AUCGUAAUCCGGUGCUGUGUGUGGUA.CCUGGUU.........UGGAUACCAGAGAGGUUAG.GAGACAGCUUCUAAAGA...........UUUAUCUU.GGCGAGGGGAA....ACCCAGGGUAGCUCCCAUCUGAGU.
Ga0265756_100207/1084-1358   UAAACGGGUAUUUGGUGCCCGGUGCUGUCCGAUGGAACCAGGCGU........UUGGUACUGGAGGGGUGGA.CAGUCUGCAUCCAUAGGCGACUGG.GGUAAGUCCUAGGGCGAGGGGCGAAAGCUCCAGGGUAGCUCCCAUCUGAGU.
Ga0265756_118005/438-164     UAAACGGGUAUUUGGUGCCCGGUGCUGUCCGAUGGA.ACCAGACGU.......UUGGUACUGGAGGGGUGGA.UAGUCUGCAUCCAUAGGCAACUGG.AGUAAGUCCUAGGGCGAGGAGCGAAAGUUCCAGGGUAGCUCCCAUCUGAGU.
Ga0316037_100890/961-1216    UAAACGGAU.AUCGUAAUCCGGUGCUGUGUGUGGUA.CCUGGUU.........UGGAUACCAGAUAGGUUAG.GAGACAGCUUCUAAAGA...........UUUAUCUU.GGCGAGGGGAA....ACCCAGGGUAGCUCCCAUCUGAGU.
Ga0184590_129607/372-627     UAAACGGAU.AUCGUAAUCCGGUGCUGUGUGUGGUA.CCUGGUU.........UGGAUACCAGAGGGGUUAG.GAGACAGCUUCUAAAGA...........UUUAUCUU.GGCGAGGGGAA....ACCCAGGGUAGCUCCCAUCUGAGU.
Ga0316031_100350/504-249     UAAACGGAU.AUCGUAAUCCGGUGCUGUGUGUGGUA.CCUGGUU.........UGGAUACCAGAGGGGUUAG.GAGACAGCUUCUAAAGA...........UUUAUCUU.GGCGAGGGGAA....ACCCAGGGUAGCUCCCAUCUGAGU.
Ga0184602_129569/1291-1036   UAAACGGAU.AUCGUAAUCCGGUGCUGUGUGUGGUA.CCUGGUU.........UGGAUACCAGAGGGGUUAG.GAGACAGCUUCUAAAGA...........UUUAUCUU.GGCGAGGGGAA....ACCCAGGGUAGCUCCCAUCUGAGU.
Ga0184593_114185/1329-1584   UAAACGGAU.AUCGUAAUCCGGUGCUGUGUGUGGUA.CCUGGUU.........UGGAUACCAGAGGGGUUAG.GAGACAGCUUCUAAAGA...........UUUAUCUU.GGCGAGGGGAA....ACCCAGGGUAGCUCCCAUCUGAGU.
Ga0184597_119352/662-406     UAAACGGAU.AUCGUAAUCCGGUGCUGUGUGUGGUA.CCUGGUU.........UGGAUACCAGAGAGGUUGG.CAAACAGCAGCUAAAGA..........CAUAGUCUU.GACGAGGAGAA....AUCCAGGGUAGCUCCCAUCUGAGU.
Ga0184571_101460/264-521     UAAACGGGUUAUUGUGGCCCGGUGUUGUGUGUGGUA.CCUGGAA.........UUGGCUCCAGAGAGGUUGG.UAAACAGCAGCUAAAGA..........CAUAGUCUU.GACGAGGAGAA....AUCCAGGGUAGCUCCCAUCUGAGU.
Ga0184570_105574/259-2       UAAACGGGUUAUUGUGGCCCGGUGUUGUGUGUGGUA.CCUGGAA.........UUGGCUCCAGAGAGGUUGG.UAAACAGCAGCUAAAGA..........CAUAGUCUU.GACGAGGAGAA....AUCCAGGGUAGCUCCCAUCUGAGU.
Ga0184603_118666/125-382     UAAACGGGUUAUUGUGGCCCGGUGUUGUGUGUGGUA.CCUGGAA.........UUGGCUCCAGAGAGGUUGG.UAAACAGCAGCUAAAGA..........CAUAGUCUU.GACGAGGAGAA....AUCCAGGGUAGCUCCCAUCUGAGU.
Ga0184594_129934/259-516     UAAACGGGUUAUUGUGACCCGGUGUUGUGUGUGGUA.CCUGGAA.........UUGGCUCCAGAGAGGUUGG.CAAACAGCAGCUAAAGA..........CAUAGUCUU.GACGAGGAGAA....AUCCAGGGUAGCUCCCAUCUGAGU.
Ga0184583_107176/526-268     UAAACGGGUUAUUGUGGCCCGGUGCUGUGUGUGGUA.CCUGGGA.........UCGGCUCCAGAGGGGUUGG.CAAACAGCAACUAAAGA..........CUUAGUCUU.GGCGAGGAGAA....AUCCAGGGUAGCUCCCAUCUGAGU.
Ga0265752_102989/468-200     UAAACGAGUUAUUGUUGCUCGGUGUUGUGUGUGAUA.CCUGGAAUUGGGUAG.UGGCAUCCAGAGUGGUUGG.UAGACUGCAUCCAUAGG.........CAUAGACCUA.GGCGAGGGGAA....ACCCAGAGGAGCUCCUAGCUGAGU.
Ga0265755_100703/282-550     UAAAUGGGUUAUUGUUGCCCAGUGUUGUGUGUGAUA.ACUGGAAUUGGGUGA.UGGCAUCCAGAGGGGUUGG.UAGACUGCAUCCAUAGG.........CAUAGACCUA.GGCGAGGGGAA....ACCCAGAGGAACUCCUAUCUGAGU.
Ga0265757_101942/232-501     UAAAUGGGUUAUUGUUGCCCAGUGUUGUGUGUGAUA.CCUGGAAUUGGGUUUUUGGCAUCCAGAGUGGUUGG.UAGACUGCAUCCAUAGG.........UAUAGACCUA.GGCGAGGGGAA....ACCCAGAGGAGCUCCUAUCUGAGU.
Ga0184580_114996/428-172     UAAACAGGGAAUUGUUACCUGGGGCUGUCCGUUCUA.CCGUGAGA........UGAGUGCACGAGGGGUCCC.AAGACUGCUUCAAUCGGCCGAAUUGCGUAAGACCGACGGCAAGGUACA.................UCUUUGAGAGU.
Ga0184597_101813/992-1248    UAAAUGGGUUAUUGUUGCCCAGGGCUGUCCGUGGUA.CCGGGAG.........UAGUGACCCGAGUAGUUCC.AAGACUGCAUCAAUCGGCCGAAUUGCGUAAGACCGAUGGCAGGGUACA.................UCUUUGAGAGU.
Ga0316040_104752/267-523     UAAAUGGGUUAUUGUUGCCCAGGGCUGUCCGUGGUA.CCGGGAG.........UAGUGACCCGAGUAGUUCC.AAGACUGCAUCAAUCGGCCGAAUUGCGUAAGACCGAUGGCAGGGUACA.................UCUUUGAGAGU.
Ga0184596_119904/895-639     UAAAUGGGUUAUUGUUGCCCAGGGCUGUCCGUGGUA.CCGGAGA.........UAGGCAUCCGAGUAGUUCC.AAGACUGCAACAAUCGGCCGAAUUGCGUAAGACCGACGGCAGUGUACA.................UCUUUGAGAGU.
Ga0184603_136155/1082-826    UAAAUGGAUUAUUGUUGUCCAGGGCUGUCCGUCAUA.CCGGGAU.........AUGGUACCCGAGGGGUCCC.AAGACUGCUUCAAUCGGCCGAAAUGCGUAAGACCGACGGCAAGGUACA.................UCUUUGAGAGU.
Ga0184573_104969/1102-845    UAAAUGGGUUAUUGUUGCCCAGGGCUGUCCGUGGUA.CCGGGAGA........UUGGCACCCGAGAGGUCCC.AAGCCUGCAUCAAUCGGCAGAAAUGCGUAAGACCGAUGGCAAGGUACA.................UCUUUGAGAGU.
Ga0184582_134418/584-328     UAAAUGGAUUAUUGUUGUCCAGGGCUGUCCGUCAUA.CCGGGAU.........AUGGUACCCGAGGGGUCCC.AAGACUGCUUCAAUCGGCCGAGAUGCGUAAGACCGACGGCAAGGUACA.................UCUUUGAGAGU.
Ga0184597_108525/2058-1801   UAAAUGGAUUAUUGUUGUCCAGGGCUGUCCGUCAUA.CCGGGAGA........UGGCUACCCGAGGGGUCCC.AAGACUGCUUCAAUCGGCCGAAAUGCGUAAGACCGACGGCAAGGUACA.................UCUUUGAGAGU.
Ga0184588_141412/669-412     UAAAUGGGUUAUUGUUGCCCAGGGCUGUCCGUGGUA.CCGGGAGA........UUGGCACCCGAGAGGUCCC.AAGCCUGCAUCAAUCGGCAGAAAUGCGUAAGACCGAUGGCAAGGUACA.................UCUUUGAGAGU.
Ga0184584_133250/1156-899    UAAAUGGGUUAUUGUUGCCCAGGGCUGUCCGUGGUA.CCGGAAAU........AGGCAAUCCGAGUAGUUCC.AAGACUGCACCAAUCGGCCGAAUUGCGUAAGACCGACGGCGGUGUACA.................UCUUUGAGAGU.
Ga0316034_100408/317-60      UAAAUGGGGUAUUGUUGCCCAGAGCUGUCCGUGGUA.CCGGGAGA........UUGGCACCCGAGAGGUCCC.AAGCCUGCAUCAAUCGGCAGAAAUGCGUAAGACCGAUGGCAAGGUACA.................UCUUUGAGAGU.
Ga0184603_127001/355-612     UAAAUGGGUUAUUGUUGCCCAGGGCUGUCCAUGGUA.CCGGGAGA........UUGGCACCCGAGAGGUCCC.AAGCCUGCAUCAAUCGGCAGAAAUGCGUAAGACCGAUGGCAAGGUACA.................UCUUUGAGAGU.
Ga0316040_100833/417-674     UAAAUGGGUUAUUGUUGCCCAGGGCUGUCCGUGGUA.CCGGGAGA........UUGGCACCCGAGAGGUCCC.AAGCCUGCAUCAAUCGGCAGAAAUGCGUAAGACCGAUGGCAAGGUACA.................UCUUUGAGAGU.
Ga0247538_100125/923-1180    UAAAUGGGUUAUUGUUGCCCAGGGCUGUCCGUGGUA.CCGGGAUA........UGAGUGCCCGAGGGGUCCC.AAGCCUGCAUCAAUCGGCAGAAUUGCGUAAGACCGAUGGCAAGGUACA.................UCUUUGAGAGU.
Ga0184597_123968/1117-860    UAAAUGGGUUAUUGUUGCCCAGGGCUGUCCGUGGUA.CCGGGAGA........UUGGCACCCGAGAGGUCCC.AAGCCUGCAUCAAUCGGCAGAAAUGCGUAAGACCGAUGGCAAGGUACA.................UCUUUGAGAGU.
Ga0247550_100043/1183-926    UAAAUGGGUUGUUGUCGCCCAGGGCUGUCCGUGGUA.CCGGGAUA........UGAGUGCCCGAGGGGUCCC.AAGCCUGCAUCAAUCGGCAGAAUUGCGUAAGACCGAUGGCAAGGUACA.................UCUUUGAGAGU.
Ga0247546_104305/303-560     UAAAUGGGUUAUUGUUGCCCAGGGCUGUCCGUGGUA.CCGGGAGU........UCAGUGCCCGAGGGGUCCC.AAGCCUGCAUCAAUCGGCAGAAAUGCGUAAGACCGAUGGCAAGGUACA.................UCUUUGAGAGU.
Ga0184585_129391/336-593     UAAAUGGGUUAUUGUUGCCCAGGGCUGUCCAUGGUA.CCGGGAGA........UUGGCACCCGAGAGGUCCC.AAGCCUGCAUCAAUCGGCAGAAAUGCGUAAGACCGAUGGCAAGGUACA.................UCUUUGAGAGU.
Ga0184584_143290/747-488     UAAAUAGGGUAUUCUUACCUAGGGCUGUCCGUGGAA.CCAGGAAU........UUGGUGCCUGAGGGGUCCUCAAGUCCGCAUCAAUCGGCCGAAUUGCGUAAGACCGACAGCAAGGUACA.................UCUUUGAGAGU.
Ga0184596_101308/441-181     UAAAUAGGGUAUUCUUACCUAGGGCUGUCCGUGGAA.CCAGGAAAU.......UUGGUGCCUGAGGGGUCCUCAAGUCCGCAUCAAUCGGCCGAAUUGCGUAAGACCGACAGCAAGGUACA.................UCUUUGAGAGU.
Ga0247551_100857/774-518     UAAAUGGGUUAUUCUUGCCCAGGGCUGUCCGUCAUA.CCGUGAGA........UAGGCACACGAGGGGUCCC.AAGACUGCAUCAAUCGGCCGAAUUGCGUAAGACCGACGGCAAGGUACA.................UCUUUGAGAGU.
Ga0184594_123990/327-70      UAAAUAGGGAAUUGUUACCUAGGGUUGUCCGUGGAA.CCGGGAAUU.......UUGGCACCCGAGGGGUCCC.AAGUCUGCAUCAAUCGGCCGAAUUGCGUAAGACCGACGGCAGGGUACA.................UCUUUGAGAGU.
Ga0316051_1068579/277-21     UAAAUAGGUUAUUCUUACCUAGGGCUGUCCGUCAUA.CCGGGAGA........UAGGCACACGAGGGGUCCC.AAGACUGCAUCAAUCGGCCGAAUUGCGUAAGACCGACGGCAAGGUACA.................UCUUUGAGAGU.
Ga0184585_150115/1593-1850   UAAAUAGGGAAUUGUUACCUAGGGUUGUCCGUGGAA.CCGGGAAAU.......UUGGUGCCCGAGGGGUCCC.AAGUCUGCAUCAAUCGGCCGAAUUGCGUAAGACCGACGGCAGGGUACA.................UCUUUGAGAGU.
Ga0184600_102925/2320-2577   UAAAUAGGGAAUUGUUACCUAGGGUUGUCCGUGGAA.CCGGGAAUU.......UUGGCACCCGAGGGGUCCC.AAGUCUGCAUCAAUCGGCCGAAUUGCGUAAGACCGACGGCAGGGUACA.................UCUUUGAGAGU.
Ga0316052_116341/27-283      UAAAUAGGUUAUUCUUACCUAGGGCUGUCCGUCAUA.CCGUGAGA........UAGGCACACGAGGGGUCCC.AAGACUGCAUCAAUCGGCCGAAUUGCGUAAGACCGACGGCAAGGUACA.................UCUUUGAGAGU.
Ga0316046_122820/50-308      UAAAUGGGUUAUUCUUGCCCAGGGCUGUCCGUCAAA.CCGUGAGA........UGAAUACACGAGAGGCUCCCAAGACUGCAUCAAUCGGCCGAAUUGCGUAAGACCGACGGCAAUGUACA.................UCUUUGAGAGU.
Ga0184588_122720/1179-921    UAAAUAGGGAAUUGUUACCUAGGGUUGUCCGUGGAAACCGGGAAAU.......UUGGUGCCCGAGGGGUCCC.AAGUCUGCAUCAAUCGGCCGAAUUGCGUAAGACCGACGGCAGGGUACA.................UCUUUGAGAGU.
Ga0247554_102280/542-286     UAAAUGGGUUAAUGCUACCCAGGGCUGUCCACCAUA.CCGUGAGA........UGGCUACACGAGAGGUCCC.AAGACUGCAUCAAUCGGCCGAGAUGCGUAAGACCGAUGGCAAGUUAAA.................UCUUUGAGAGU.
Ga0247538_102781/455-199     UAAAUGGGUGAAUGCUGCCCAGGGCUGUCCACCAUA.CCGUGAGA........UGGCUACACGAGUGGUCCC.AAGACUGCAUCAAUCGGCCGAGAUGCGUAAGACCGACGGCAAGUUAAA.................UCUUUGAGAGU.
                             ....<<<<<.......>>>>><<<<....<<<<<<<..<<<<.................>>>>....<<<<<..............<<<<...............>>>><<<<<<<.................................>
                             ....2212.........2122.101....11122.0..0121.................1210....00221..............2202...............2022.1.01..................................
                             UAAAYGGGU∘AUUGUURCCCRG•GCUGU•YGU•RUA-CC•GGR•∘--------U•GRYRCY•GAGRGGUY••-•AG•CYGCAUC•AU•GG∘∘∘∘∘∘∘∘∘∘Y•AGACC•A∘GGCRAGG•R•A-----------------YCY•U••GAGU-
```

alignment positions 151 · · · 300

```
Ga0184594_129452/258-513     .ACGUCGCAUC.CUAACAU.GCCAAUCCGGAGAAUGGGGUGAAACUCCUCUGAAAUCCGGUGUUG..CUU.CA...................................UUCCACACGAAGCACAAACAAACAGAAACACCGGGGAAUU..
Ga0247524_115310/154-451     .ACGUCGCAUC.CUAACAU.GCCAAUCCGGAGAAUGGGGUGAAACUCCUCUGUAAUCCGGUCAGGGAAUCUCAUUCCGCUUAUGGAAGAACUUAUCGCCAUAAGGUGACGCGUCGGGAAGCGUGUUGUUGAGGAAACACGGUCUCUCUGC
Ga0265756_100207/1084-1358   .ACGUCGCAAG.UCCACGU.UCCAACCCGGAGAAUGGGGUGAAACUCCUCUGAAAUCCGGUGUCG..AUU.CA...................................UCCAUCGGGAAGCACAAACUUGCAGAAACACACAGAGAUU..
Ga0265756_118005/438-164     .ACGUCGCAAG.UCCACGU.UCCAACCCGGAGAAUGGGGUGAAACUCCUCUGAAAUCCGGUGUAG..AUU.CA...................................UCCAUCGGGAAGCACAAACUAGCAGAAACACACAGAGAGU..
Ga0316037_100890/961-1216    .ACGUCGCAUC.CUAACAU.GCCAAUCCGGAGAAUGGGGUGAAACUCCUCUGAAAUCCGGUGUUG..CUU.CA...................................UUCCACACGAAGCACAAACAAACAGAAACACCGGGGAAUU..
Ga0184590_129607/372-627     .ACGUCGCAUC.CUAACGU.GCCAAUCCGGAGAAUGGGGUGAAACUCCUCUGAAAUCCGGUGUUG..CUU.CA...................................UUCCACACGAAGCACAAACAAACGGAAACACCAGGGAAUU..
Ga0316031_100350/504-249     .ACGUCGCAUC.CUAACAU.GCCAAUCCGGAGAAUGGGGUGAAACUCCUCUGAGAUCCGGUGUUG..CUU.CA...................................UUCCACACGAAGCACAAACAAACGGAAACACCAGGGAAUU..
Ga0184602_129569/1291-1036   .ACGUCGCAUC.CUAACGU.GCCAAUCCGGAGAAUGGGGUGAAACUCCUCUGAGAUCCGGUGUUG..CUU.CA...................................UUCCACACGAAGCACAAACAAACGGAAACACCAGGGAAUU..
Ga0184593_114185/1329-1584   .ACGUUGCAUC.CUAACGU.GCCAAUCCGGAGAAUGGGGUGAAACUCCUCUGAGAUCCGGUGUUG..CUU.CA...................................UUCCACACGAAGCACAAACAAACGGAAACACCAGGGAAUU..
Ga0184597_119352/662-406     .ACGUCGCAAG.CCAACGC.GCCAAUCCGGAGAAUGGGGUGAAACUCCUCUGAAAUCCGGUGUUG..CUU.CA...................................UUCCACACGAAGCACAAACAAACUGAAACACUUCAGAGGU..
Ga0184571_101460/264-521     .ACGUCGCAAA.CCAACGU.GCCAAUCCGGAGAAUGGGGUGAAACUCCUCUGAAAUCCGGUGUUG..CUU.CA...................................UUCCACACGAAGCACAAACAAACUGAAACACUUCAGAGGU..
Ga0184570_105574/259-2       .ACGUCGCAAG.CCAACGU.GCCAAUCCGGAGAAUGGGGUGAAACUCCUCUGAAAUCCGGUGUUG..CUU.CA...................................UUCCACACGAAGCACAAACAAACUGAAACACUUCAGAGGU..
Ga0184603_118666/125-382     .ACGUCGCAAG.CCAACGC.GCCAAUCCGGAGAAUGGGGUGAAACUCCUCUGAAAUCCGGUGUUG..CUU.CA...................................UUCCACACGAAGCACAAACAAACUGAAACACUUCAGAGGU..
Ga0184594_129934/259-516     .ACGUCGCAAG.CCAACGC.GCCAAUCCGGAGAAUGGGGUGAAACUCCUCUGAAAUCCGGUGUUG..CUU.CA...................................UUCCACACGAAGCACAAACAAACUGAAACACUUCAGAGGU..
Ga0184583_107176/526-268     .ACGUCGCAAG.CCAACGC.GCCAAUCCGGAGAAUGGGGUGAAACUCCUCUGAAAUCCGGUGUUG..CUU.CA...................................UUCCACACGAAGCACAAACAAACUGAAACACUUCAGAGGU..
Ga0265752_102989/468-200     .ACGUCGCACA.CCAACGUGGCCAAUCCGGAGAAUGGGGUGAAACUCCUCUGAAAUACGGUGUUG..CCUUCA...................................UAUCACACGAAGCACAAACAAACUGAAACACUUUGGGUGU..
Ga0265755_100703/282-550     .ACGUCGCAAG.CCAACGUGGCCAAUCCGGAGAAUGGGGUGAAACUCCUCUGAAAUACGGUGUUG..CCUUCA...................................UAUCACACGAAGCACAAACAAAUUGAAACACUUUGGGUGU..
Ga0265757_101942/232-501     .ACGUCGCACG.CCAACGUGGCCAAUCCGGAGAAUGGGGUGAAACUCCUCUGAAAUACGGUGUUG..CCUUCA...................................UAUCACACGAAGCACAAACAAAUGGAAACACUUCAGGUGU..
Ga0184580_114996/428-172     CUUGUCGCACA.GGGACGU.GCAAAUCCGGAGAAUGGGGUGGAACUCCUCUGAAAUCCGGUAA.G..CUU.CA...................................UAGAGCGGGAAGCCCAAACAGAAUGAAACACGCUGGCAGUUG
Ga0184597_101813/992-1248    GCUGUCGCAAG.GGAACGC.GCUAAUCCGGAGAAUGGGGUGAGACUCCUCUGAAAUCCGGUGUUG..CUU.CA...................................UUCCACGGGAAGCCCAAACAGAAUGAAACACGCAGAUAGU..
Ga0316040_104752/267-523     GCUGUCGCAAG.GGAACGC.GCUAAUCCGGAGAAUGGGGUGAGACUCCUCUGAAAUCCGGUGUUG..CUU.UA...................................UUCCACGGGAAGCCCAAACAGAAUGAAACACGCAGAUAGU..
Ga0184596_119904/895-639     ACUGUCGCAAG.GGAACGU.GCCAAUCCGGAGAAUGGGGUGAAACUCCUCUGAAAUCCGGUGUUG..CUU.UA...................................UACCACGGGAAGCCCAAACAGAAUGAAACACGCAGAUAGU..
Ga0184603_136155/1082-826    CUUGUCGCACA.GGGACGU.GCCAAUCCGGAGAAUGGGGUGAAACUCCUCUGAGAUCCGGUGUUG..CUU.CA...................................UUUGACGGGAAGCCCAAACAAGUUGAAACACACUGGCAGU..
Ga0184573_104969/1102-845    CUUGUCGCACA.GGGACGC.GCCAAUCCGGAGAAUGGGGUGAAACUCCUCUGAAAUCCGGUGUUG..CUU.CA...................................UUCCACACGAAGCACAAACAAACUGAAACACUUCAGAGGU..
Ga0184582_134418/584-328     CUUGUCGCACA.GGGACGU.GCCAAUCCGGAGAAUGGGGUGAAACUCCUCUGAAAUCUGGUGUUG..CUU.CA...................................UUCCAUGGGAAGCCCAAACAAAGUGAAACACGCAUUUAGU..
Ga0184597_108525/2058-1801   CUUGUCGCACA.GGGACGU.GCCAAUCCGGAGAAUGGGGUGAAACUCCUCUGAGAUCCGGUGUUG..CUU.CA...................................UUUGACGGGAAGCCCAAACAAGUUGAAACACACUGGCAGU..

                             >>>>>>>.....>>>>>.........................................................<<<<....>>>>........<<<<<....>>>>>>>>>>>>....>>>>....<<<<.......<<<<<<<<<<..
                             ..10.1......12200.........................................................0000....0000........00000....000000.22111....101......222.......0212..2.....
                             ∘•YGUCGCA•R-••RACGY-GCYAAUCCGGAGAAUGGGGUGAAACUCCUCUGAAAUCCGGUGYYG--CUU-CA-----------------------------------U•Y•RCR•GAAGC•CAAACARR••GAAACAC•Y•RR•RGU--
```

```
Ga0184588_141412/669-412      CUUGUCGCACA.GGGACGC.GCCAAUCCGGAGAAUGGGGUGAAACUCCUCUGAGAUCCGGUGUUG..CUU.CA...................................UUUGACGGGAAGCCCAAACAAGUUGAAACACACUGGCAGU..
Ga0184584_133250/1156-899     ACUGUCGCAAG.GGAACGC.GCCAAUCCGGAGAAUGGGGUGAAACUCCUCUGAAAUCCGGUGUUG..CUU.UA...................................CACCACGGGAAGCCCAAACAGAAUGAAACACGCAGAUAGU..
Ga0316034_100408/317-60       CUUGUCGCACA.GGGACGU.GCCAAUCCGGAGAAUGGGGUGAAACUCCUCUGAGAUCCGGUGUUG..CUU.CA...................................UUUGACGGGAAGCCCAAACAAGUUGAAACACACUGGCAGU..
Ga0184603_127001/355-612      CUUGUCGCACA.GGGACGC.GCCAAUCCGGAGAAUGGGGUGAAACUCCUCUGAAAUCUGGUGUUG..CUU.CA...................................UUCCAUGGGAAGCCCAAACAAAGUGAAACACGCAUUUAGU..
Ga0316040_100833/417-674      CUUGUCGCACA.GGGACGC.GCCAAUCCGGAGAAUGGGGUGAAACUCCUCUGAAAUCUGGUGUUG..CUU.CA...................................UUCCACGGGAAGCCCAAACAAAGUGAAACACGCAUUUAGU..
Ga0247538_100125/923-1180     CUUGUCGCACA.GGGACGC.GCCAAUCCGGAGAAUGGGGUGAAACUCCUCUGAAAUCCGGUGUUG..CUU.CA...................................UUCCACGGGAAGCCCAAACAAAGUGAAACACGCAUUUGGU..
Ga0184597_123968/1117-860     CUUGUCGCACA.GGGACGC.GCCAAUCCGGAGAAUGGGGUGAAACUCCUCUGAAAUCUGGUGUUG..CUU.CA...................................UUCCAUGGGAAGCCCAAACAAAGUGAAACACGCAUUUAGU..
Ga0247550_100043/1183-926     CUUGUCGCACA.GGGACGC.GCCAAUCCGGAGAAUGGGGUGAAACUCCUCUGAAAUCCGGUGUUG..CUU.CA...................................UUCCACGGGAAGCCCAAACAAAGUGAAACACGCAUUUGGU..
Ga0247546_104305/303-560      CUUGUCGCACA.GGGACGC.GCCAAUCCGGAGAAUGGGGUGAAACUCCUCUGAAAUCCGGUGUUG..CUU.CA...................................UUCCACGGGAAGCCCAAACAAAGUGAAACACGCAUUUGGU..
Ga0184585_129391/336-593      CUUGUCGCACA.GGGACGC.GCCAAUCCGGAGAAUGGGGUGAAACUCCUCUGAAAUCUGGUGUUG..CUU.CA...................................UUCCACGGGAAGCCCAAACAAAGUGAAACACGCAUUUAGU..
Ga0184584_143290/747-488      CUUGUUGCAAGGAGGACAU.GCCAAUCCGGAGAAUGGGGUGAGACUCCUCUGAAAUCCGGUGUCG..CUU.CA...................................UUCCACGGGAAGCCCAAACAAAGUGAAACACGCGAGCAGU..
Ga0184596_101308/441-181      CUUGUUGCAAGGAGGACAU.GCCAAUCCGGAGAAUGGGGUGAGACUCCUCUGAAAUCCGGUGUCG..CUU.CA...................................UUCCACGGGAAGCCCAAACAAAGUGAAACACGCGAGCAGU..
Ga0247551_100857/774-518      GUUGUCGCAUA.GGGACGU.GCAAAUCCGGAGAAUGGGGUGAAACUCCUCUGAAAUCCGGUA.CG..CUU.CA...................................UUUGACGGGAAGCCCAAACAAACUGAAACACGCUGGCAGU..
Ga0184594_123990/327-70       GCUGUCGCAAG.GGGACGU.GCAAAUCCGGAGAAUGGGGUGGAACUCCUCUGAAAUCUGGUG.CG..CUU.CA...................................UUCCACGGGAAACCCAAACAAGAUGAAACACGCAUUCAGU..
Ga0316051_1068579/277-21      GUUGUCGCAUA.GGGACGU.GCAAAUCCGGAGAAUGGGGUGAGACUCCUCUGAAAUCCGGUA.CG..CUU.CA...................................UUUGACGGGAAGCCCAAACAAACUGAAACACGCUGGCAGU..
Ga0184585_150115/1593-1850    GCUGUCGCAAG.GGGACGU.GCAAAUCCGGAGAAUGGGGUGGAACUCCUCUGAAAUCUGGUG.CG..CUU.CA...................................UUCCACGGGAAACCCAAACAAGAUGAAACACGCAUUCAGU..
Ga0184600_102925/2320-2577    GCUGUCGCAAG.GGGACGU.GCAAAUCCGGAGAAUGGGGUGGAACUCCUCUGAAAUCUGGUG.CG..CUU.CA...................................UUCCACGGGAAACCCAAACAAGAUGAAACACGCAUUCAGU..
Ga0316052_116341/27-283       GUUGUCGCAUA.GGGACGU.GCAAAUCCGGAGAAUGGGGUGAGACUCCUCUGAAAUCCGGUA.CG..CUU.CA...................................UUUGACGGGAAGCCCAAACAAACUGAAACACGCUAGCAGU..
Ga0316046_122820/50-308       AUUGUCGCACAGGGAACAA.GCAAAUCCGGAGAAUGGGGUGAAACUCCUCUGAAAUCCGGUC.CG..CUU.CA...................................UUUGACGGGAAGCCCAAACAAACUGAAACACGCUGGCAGU..
Ga0184588_122720/1179-921     GCUGUCGCAAG.GGGACGU.GCAAAUCCGGAGAAUGGGGUGGAACUCCUCUGAAAUCUGGUG.CG..CUU.CA...................................UUCCACGGGAAACCCAAACAAGAUGAAACACGCAUUCAGU..
Ga0247554_102280/542-286      CUUGUCGCAUA.CGGACGU.GCAAACCCGGAGAAUGGGGUGGGACUCCUCUGAAAUCCGGUGC.G..CUU.CA...................................UUUGGUGGGAAGCCCAAACAAGUUGAAACACGCUGACUGU..
Ga0247538_102781/455-199      CUUGUCGCACA.GGGACGU.GCAAACCCGGAGAAUGGGGUGGAACUCCUCUGAAAUCCGGUGC.G..CUU.CA...................................UUUGGUGGGAAGCCCAAACAAGUUGAAACACGCUGAUUGU..
                              >>>>>>>.....>>>>>.........................................................<<<<....>>>>........<<<<<....>>>>>>>>>>>>....>>>>....<<<<.......<<<<<<<<<<..
                              ..10.1......12200.........................................................0000....0000........00000....000000.22111....101.....222.......0212..2.....
                              ○●YGUCGCA●R-●●RACGY-GCYAAUCCGGAGAAUGGGGUGAAACUCCUCUGAAAUCCGGUGYYG--CUU-CA-----------------------------------U●Y●RCR●GAAGC●CAAACARR●●GAAACAC●Y●RR●RGU--
```

alignment positions 301 · · · 331

```
Ga0184594_129452/258-513      UGUC..GAUCCUUGGGUACAUUAA.GUUUGA
Ga0247524_115310/154-451      AGUUCGGGGAGGCCGGCAUAUUAC.UCAUAU
Ga0265756_100207/1084-1358    AGAC..GCCUCUGUGGUAUAUUAUUGCUUGA
Ga0265756_118005/438-164      AGAC..GACUCUGUGGUAUAUUAAUGCUUGA
Ga0316037_100890/961-1216     UGUC..GAUCCUUGGGUACAUUAA.GUUUGA
Ga0184590_129607/372-627      UGUC..GAUCCUUGGGUACAUUAA.GUUUGA
Ga0316031_100350/504-249      UGUC..GAUCCUUGGGUACAUUAA.GUUUGA
Ga0184602_129569/1291-1036    UGUC..GAUCCUUGGGUACAUUAA.GUUUGA
Ga0184593_114185/1329-1584    UGUC..GAUCCUUGGGUACAUUAA.GUUUGA
Ga0184597_119352/662-406      UGUU..GAUCUGGAGGUACAUUAA.GUUUGA
Ga0184571_101460/264-521      UGUU..GACCUGGAGGUACAUUAA.GUUUGA
Ga0184570_105574/259-2        UGUU..GACCUGGAGGUACAUUAA.GUUUGA
Ga0184603_118666/125-382      UGUU..GAUCUGGAGGUACAUUAA.GUUUGA
Ga0184594_129934/259-516      UGUU..GAUCUGGAGGUACAUUAA.GUUUGA
Ga0184583_107176/526-268      UGUU..GAUCUGAAGGCACAUUAA.GUUUGA
Ga0265752_102989/468-200      UGUC..GACUCAGAGGCACAUUAA.GUUUGA
Ga0265755_100703/282-550      UGUC..GACCUGAAGGCACAUUAA.AUUUGA
Ga0265757_101942/232-501      AGUC..GACCUGCAGGCACAUUAA.AUUUGA
Ga0184580_114996/428-172      UC....GAGCUGGUGGUAUAUUAU.UUCUGA
Ga0184597_101813/992-1248     UGUC..GAAUUUGCGGAACAUUAA.UUCUGA
Ga0316040_104752/267-523      UGUC..GAAUUUGCGGAACAUUAA.UUCUGA
Ga0184596_119904/895-639      UGUC..GCAUUUGCGGAACAUUAA.UUCUGA
Ga0184603_136155/1082-826     UGUC..GAGUCAGUGGUAUAUUAU.ACUUGA
Ga0184573_104969/1102-845     UGUU..GACCUGGAGGUACAUUAA.GUUUGA
Ga0184582_134418/584-328      UGGC..GCAUUUGCGGUAUAUUAA.CUUUGA
Ga0184597_108525/2058-1801    UGUC..GAGUCAGUGGUAUAUUAU.ACUUGA
Ga0184588_141412/669-412      UGUC..GAGUCAGUGGUAUAUUAU.ACUUGA
Ga0184584_133250/1156-899     UGUC..GCAGUUGCGGAACAUUAA.UUCUGA
Ga0316034_100408/317-60       UGUC..GAGUCAGUGGUAUAUUAU.ACUUGA
Ga0184603_127001/355-612      UGGC..GCAUUUGCGGUAUAUUAA.CUUUGA
Ga0316040_100833/417-674      UGGC..GCAUUUGCGGGAUAUUAA.CUUUGA
Ga0247538_100125/923-1180     UGGC..GUAUUUGCGGGAUAUUAA.CUUUGA
Ga0184597_123968/1117-860     UGGC..GCAUUUGCGGUAUAUUAA.CUUUGA
Ga0247550_100043/1183-926     UGGC..GUAUUUGCGGGAUAUUAG.CUUUGA
Ga0247546_104305/303-560      UGGC..GUAUUUGCGGGAUAUUAA.CUUUGA
Ga0184585_129391/336-593      UGGC..GCAUUUGCGGGAUAUUAA.CUUUGA
Ga0184584_143290/747-488      CGAC..GCGUUUGCGGUAUAUUAG.CUUUGA
Ga0184596_101308/441-181      UGGC..GCGUUUGCGGUAUAUUAG.CUUUGA
Ga0247551_100857/774-518      UGUC..GCGUUGGCGGUAUAUUAC.GUUUGA
Ga0184594_123990/327-70       UGUC..GCGUUUGCGGUAUAUUAU.UCUUGA
Ga0316051_1068579/277-21      UGUC..GCGUUGGCGGUAUAUUAC.GUUUGA
Ga0184585_150115/1593-1850    UGUC..GCGUUUGCGGUAUAUUAU.UCUUGA
Ga0184600_102925/2320-2577    UGAC..GCGUUUGCGGUAUAUUAU.UCUUGA
Ga0316052_116341/27-283       UGUC..GCGUUGGCGGUAUAUUAC.GUUUGA
Ga0316046_122820/50-308       UGUC..GCGUUGGCGGUAUAUUAC.GUUUGA
Ga0184588_122720/1179-921     UGUC..GCGUUUGCGGUAUAUUAU.UCUUGA
Ga0247554_102280/542-286      UGUC..GCGUUGGCGGUAUAUUAU.ACUUGA
Ga0247538_102781/455-199      UGUC..GUGUUGGCGGUAUAUUAU.ACUUGA
                              .....>>>>>>>>>>..........>>>>..
                              ........2..2120..........222...
                              UG●C--G●●YY●G●GGYAYAUUA●-●YUUGA
```

7 hairpin-TSA

For a description of this alignment, see Supplementary Table 2

7.1 Multiple-sequence alignment

Each ribozyme in this alignment is identified by its genomic location in the form SEQID/START-END. SEQID (the sequence accession) is derived from sources such as IMG/M or GenBank. START is the coordinate of the 5′ nucleotide of the hairpin ribozyme, and END corresponds to the 3′ nucleotide. If START>END, then the ribozyme is on the reverse complement strand.

Nucleotides proposed to basepair as part of the consensus structure are shaded in color when they comprise Watson-Crick or G-U pairs. Otherwise they are shaded gray. Conserved stems are also indicated at the bottom of the alignment by angle brackets, where matching $<$ and $>$ denote base-paired columns. Below these angle brackets, the symbol "2" denotes base pairs exhibiting covariation according to the statistically well-founded R-scape method. "1" denotes base pairs exhibiting covariation according to R2R's simplistic method. "0" denotes base pairs that are not observed to mutate and "?" denotes base pairs that have a significant frequency of non-canonical nucleotides for Watson-Crick or G-U pairs ($>$ 5%). Below these base pair annotation is the consensus sequence: "R" = "A" or "G", "Y" = "C" or "U", red nucleotides: nucleotide identity conserved more than 97% of the time, black nucleotides: 90%, gray nucleotides: 75%, red circle (•): nucleotide is present 97% of the time, black circle (•): 90%, gray circle (•): 75%, white circle (◦): 50%. All percentages of sequences just described (e.g. 97% conserved) assume that sequences have been weighted by the GSC algorithm implemented by the Infernal software package.

The alignment begins on the next page.

alignment positions 1 · · · 150

```
GFLP01288341.1/477-563      CAGC.GGUCGGGGGUG....UU....................................................CAACA.......................................................................
GELB01000625.1/449-345      AAUC.GGUCCCGAAGU....CC.GUGCG..................AAAGC.AAUCC.................CAAGC.......................................................................
GEHE01004364.1/747-855      UGUC.AGUCCCGCCUA...C...............................GAGGUUUA.............AGUC...............CCC........................................................
HAMY010092562.1/352-449     CCAC.GGUCCAAGCAA...G...............................CGCUCU.................AACA........................................................................
GELB01009586.1/9654-9760    GUUC.UGUCCCAGAGU....CCGUGCGA..................AUAGC.AAUCC.................CAAGC.......................................................................
HAMY010944827.1/93-180      GGUC.GGUCCAUCGGA.......................................UG.................CUUCA.......................................................................
GFLV01046660.1/385-314      UCGC.GGUCCAGUAA................................................................GA........AA...........................................................
HAMY011136267.1/17-124      GAUU.UGACCUGAAGUU..CGUG............................CGUUUGA..............CG...UA............GU.........................................................
HAMY010289101.1/84-240      GUGC.UGUCCCAACGAG.AGAAA............................GCUCGA.................AAGCC.......................................................................
GGEZ01023376.1/2323-2425    ACUC.GGUCCCACACUGCCC...A..........................UAGCUCU................AAAGCG.......................................................................
GIFJ01641300.1/289-117      UAGU.GGUCCUCAGGG....UGGGCU.....................CAAC......AGCCCAGCCGCAUCUCC.................AUGACGCCCCCCCCUCCUUCUGUAUCGAAGGGG..........................
GELB01009586.1/4493-4600    UAUU.UGUCCCUGAGU...C.CGUGCU...................GUAAA........................................GCAGU......................................................
HAMY011241849.1/33-148      AUGC.UGUCAUUA......................................UGACUUCGGCC......AUACAC.................AUAAUUGU...................................................
HAMY010478751.1/6-198       UCUC.UGUCCUUGC.....................................AUACGGACUCCCA...AAGUCCA.................UGGGUAUUGGUCCCGGUGUUUAAUAGCCGUGCACUCUGCAAUCCGGU............
GFLP01286833.1/1232-1306    GUGC.GGUCCGGGUCA........................................C.................UUCUU.......................................................................
HAMY010507670.1/339-269     GUGC.AGUCCCGC..................................................................U.........UU...........................................................
GFLP01012994.1/647-848      GUCC.UGUCCCAGCUC...U...GGAACC...............CCAAAGU......UC.............CU.................CGGGUAUGAAUGGUUUGAUUAACUGUGUCAGUGCUUACGUUACGGAUUUCA........
GFLP01441414.1/641-842      GUCC.UGUCCCAGCUC...U...GGAACC...............CCAAAGU......UC.............CU.................CGGGUAUGAAUGGUUUGAUUAACUGUGUCAGUGCUUACGUUACGGAUUUCA........
GFLP01288095.1/76-187       CGUC.GGUCCCAGAGU....CCG............................UGCUUGAGU...........CGA.................GCAAUC.....................................................
GFLP01329911.1/355-466      CGUC.GGUCCCAGAGU....CCG............................UGCUUGAGU...........CGA.................GCAAUC.....................................................
HAMY010552344.1/367-468     UAUC.AGUCCUUUCAU...GGAC............................UACGGC.................UUCAA.......................................................................
HALR01192984.1/243-152      ...C.AGUUGGGGAUU....GAC........................................................CUAAU..CACAU...........................................................
GAOU01012890.1/16-144       CGUC.UGUCUCAAC.....................................ACUGUUCCA..........CUCU.................UUCAG......................................................
GGEZ01023376.1/2884-2783    UGUC.UGAGU.CACAGG.AA..GU..........................UAUCGGC.................UAGCA.......................................................................
GFHZ01110905.1/44-117       GUGC.GGGGCCCCCUA....CA.........................................................GC........CG...........................................................
GFLP01271181.1/44-117       GUGC.GGGGCCCCCUA....CA.........................................................GC........CG...........................................................
GFLP01149597.1/702-629      GUGC.GGGGCCCCCUA....CA.........................................................GC........CG...........................................................
GFLP01283058.1/1206-1133    GUGC.GGGGCCCCCUA....CA.........................................................GC........CG...........................................................
GFLP01851030.1/254-181      GUGC.GGGGCCCCCUA....CA.........................................................GC........CG...........................................................
GFHZ01110905.1/473-392      CUGC.GGUCC.CGGAG....AU.........................................................CCG......CAU...........................................................
GFLP01149597.1/273-354      CUGC.GGUCC.CGGAG....AU.........................................................CCG......CAU...........................................................
GFLP01271181.1/473-392      CUGC.GGUCC.CGGAG....AU.........................................................CCG......CAU...........................................................
GFLP01283058.1/777-858      CUGC.GGUCC.CGGAG....AU.........................................................CCG......CAU...........................................................
GEHE01005692.1/325-248      GCGC.AGUCGGGGAUU...............................................................C..........A...........................................................
GIFI01514496.1/268-111      GAUC.GGUCUUCAUUG....UUUUCACUCUCUUUGUACGAGAAAGAAAGCG........................................GAGCCUGAAGCUUUCACACU.......................................
GIFI01514496.1/991-834      GAUC.GGUCUUCAUUG....UUUUCACUCUCUUUGUACGAGAAAGAAAGCG........................................GAGCCUGAAGCUUUCACACU.......................................
GIFI01514496.1/1714-1557    GAUC.GGUCUUCAUUG....UUUUCACUCUCUUUGUACGAGAAAGAAAGCG........................................GAGCCUGAAGCUUUCACACU.......................................
HAMY010496177.1/29-164      CGUC.UGUCCACAAAGA..U.GA............................AGCUGUAGGCAG.....GAACCA.................CCCUUCCUAACCCG.............................................
HALR01183481.1/47-138       ...C.UGUUGGGAA.....................................UUGAGC.................UAUUC.......................................................................
GELB01009586.1/5137-5031    UAUU.UGAGCUGGGAU......UACAGUG...............GAUUGCACCAAUG.................UCGG........................................................................
GFZV01012863.1/1819-1884    GUGC.AGUCGGGGU.................................................................CC........AC...........................................................
GFZV01012863.1/6433-6498    GUGC.AGUCGGGGU.................................................................CC........AC...........................................................
GFZV01012863.1/11047-11112  GUGC.AGUCGGGGU.................................................................CC........AC...........................................................
HAMY010040351.1/14-335      UCUC.UGUCCCCGUCAC..G.GAUUCCCAAA...........UCCGAGGUA........................................UGGGCCUGUUGAAUGUGUCCACUCGCCGUUCGCGAGCAGUGUUUCGUCUCUGUCGGGUA
GEHG01006370.1/310-247      GAGC.GGUCCGC...................................................................CU.......AGU...........................................................
GFHZ01111161.1/316-388      GUGC.GGUCCA.CCUA...............................................................CUU......CGU...........................................................
GFLP01287108.1/316-388      GUGC.GGUCCA.CCUA...............................................................CUU......CGU...........................................................
GFLP01290863.1/86-27        CAUC.GGUCCCAA.........................................................................................................................................
GFLP01019157.1/196-271      CUGC.GGGGCCUCC.C...............................................................AC........CG...........................................................
GGFA01011333.1/384-316      CGUC.GGUCCCUU.........................................................................................................................................
IACP01004784.1/205-112      GUGU.GGACACUGUUA...............................................................GAUGUGUCCGAA...........................................................
IACP01004784.1/527-434      GUGU.GGACACUGUUA...............................................................GAUGUGUCCGAA...........................................................
IACP01025381.1/120-213      GUGU.GGACACUGUUA...............................................................GAUGUGUCCGAA...........................................................
IACP01025381.1/442-535      GUGU.GGACACUGUUA...............................................................GAUGUGUCCGAA...........................................................
HALR01192983.1/272-180      ....UGGUUGGGAAUU....GA....................................................U....CU........AU...........................................................
                            <<<<.....<<<<<<<...................................<<<<<<.............................................................................................
                            2221.....222222.......................................................................................................................................
                            ●●●C-●GUCY●●●●○○--------------------------------------------------------------------------------------------------------------------------------------
```

alignment positions 151 · · · 300

```
GFLP01288341.1/477-563      ......................................................................................................................................................
GELB01000625.1/449-345      ......................................................................................................................................................
GEHE01004364.1/747-855      ......................................................................................................................................................
HAMY010092562.1/352-449     ......................................................................................................................................................
GELB01009586.1/9654-9760    ......................................................................................................................................................
HAMY010944827.1/93-180      ......................................................................................................................................................
GFLV01046660.1/385-314      ......................................................................................................................................................
HAMY011136267.1/17-124      ......................................................................................................................................................
HAMY010289101.1/84-240      ......................................................................................................................................................
GGEZ01023376.1/2323-2425    ......................................................................................................................................................
GIFJ01641300.1/289-117      ...........................................................................................................................GGGGGCCAUUUGUUGAGUAGCGUCCGA
GELB01009586.1/4493-4600    ......................................................................................................................................................
HAMY011241849.1/33-148      ...................................................................................................................................................GUC
HAMY010478751.1/6-198       .............................................................................................................AACCACCCCGGGUGCACAUGUCACUCCUAUUGAAAUGCCUC
GFLP01286833.1/1232-1306    ......................................................................................................................................................
HAMY010507670.1/339-269     ......................................................................................................................................................
GFLP01012994.1/647-848      ........................................................................................................CUUACGAGGUCCGCGACUUGGGCAUUGCUACCGUGAGUCAUCUCAC
GFLP01441414.1/641-842      ........................................................................................................CUUACGAGGUCCGCGACUUGGGCAUUGCUACCGUGAGUCAUCUCAC
GFLP01288095.1/76-187       .....................................................................................................................................................C
GFLP01329911.1/355-466      .....................................................................................................................................................C
HAMY010552344.1/367-468     ......................................................................................................................................................
HALR01192984.1/243-152      ......................................................................................................................................................
GAOU01012890.1/16-144       ......................................................................................................................................................

                            ......................................................................................................................................................
                            ......................................................................................................................................................
                            ------------------------------------------------------------------------------------------------------------------------------------------------------
```

```
GGEZ01023376.1/2884-2783     ......................................................................................................................................................
GFHZ01110905.1/44-117        ......................................................................................................................................................
GFLP01271181.1/44-117        ......................................................................................................................................................
GFLP01149597.1/702-629       ......................................................................................................................................................
GFLP01283058.1/1206-1133     ......................................................................................................................................................
GFLP01851030.1/254-181       ......................................................................................................................................................
GFHZ01110905.1/473-392       ......................................................................................................................................................
GFLP01149597.1/273-354       ......................................................................................................................................................
GFLP01271181.1/473-392       ......................................................................................................................................................
GFLP01283058.1/777-858       ......................................................................................................................................................
GEHE01005692.1/325-248       ......................................................................................................................................................
GIFI01514496.1/268-111       ........................................................................................................................................GAAAAGUUGUGACG
GIFI01514496.1/991-834       ........................................................................................................................................GAAAAGUUGUGACG
GIFI01514496.1/1714-1557     ........................................................................................................................................GAAAAGUUGUGACG
HAMY010496177.1/29-164       ..............................................................................................................................................GAGGUUGG
HALR01183481.1/47-138        ......................................................................................................................................................
GELB01009586.1/5137-5031     ......................................................................................................................................................
GFZV01012863.1/1819-1884     ......................................................................................................................................................
GFZV01012863.1/6433-6498     ......................................................................................................................................................
GFZV01012863.1/11047-11112   ......................................................................................................................................................
HAMY010040351.1/14-335       ACGCCCCUUGAGAGGGUGUGUACUCAUCGUGAGUCUGGGAAAGCAUGGUGUUGUCACUGGUCACCUGGGAGUAACACCCUGGUGUGACGAUUGUCGCAACAUGUCAACCCUCGCAAACUUUGAGUUCCCACAAGACGUCUCCUGGAUCCU
GEHG01006370.1/310-247       ......................................................................................................................................................
GFHZ01111161.1/316-388       ......................................................................................................................................................
GFLP01287108.1/316-388       ......................................................................................................................................................
GFLP01290863.1/86-27         ......................................................................................................................................................
GFLP01019157.1/196-271       ......................................................................................................................................................
GGFA01011333.1/384-316       ......................................................................................................................................................
IACP01004784.1/205-112       ......................................................................................................................................................
IACP01004784.1/527-434       ......................................................................................................................................................
IACP01025381.1/120-213       ......................................................................................................................................................
IACP01025381.1/442-535       ......................................................................................................................................................
HALR01192983.1/272-180       ......................................................................................................................................................
                             ......................................................................................................................................................
                             ......................................................................................................................................................
                             ------------------------------------------------------------------------------------------------------------------------------------------------------
```

alignment positions 301 · · · 448

```
GFLP01288341.1/477-563       .....................CACCUCU.GGAAGCUG.GCCAGGGAAACACCUAUAUCC....................AUUUG........................................UGGGUAUCGGGCACAUUAACUGGC
GELB01000625.1/449-345       ........GGGUAG.......GAUUCGG.AGAAGAUU.GGCAGGGAAACACACUGAU.........................GUAGC......................................GAAUCAGUGGUACAUUAACUGCC
GEHE01004364.1/747-855       ...UUAGAUCUC........ACGGGUGG.AGAGGACA.ACCAGGGAAACACACAAUGCGCC...................UCA...............................CU.......GGCAUAUUGUGGCACAUUAACUGGU
HAMY010092562.1/352-449      ......AGAGUAC.G.....GUUGC.UG.GGAAGUGG.GUCCAAGAAACAUUUCGCCCA.......................UUAAG......................................UGUGCGAAAGCACAUUACUGGAC
GELB01009586.1/9654-9760     ........GGGUAG.......GAUCUGG.AGAAGAACUGGCAGGGAAACAUAACGGAG.........................UAGC......................................GAACCGUUAGUAUAUUAACUGCC
HAMY010944827.1/93-180       ......CA.............UCCUUUG.GGAAGACC.GCCAGUGAAACAUGCAAACUC.......................ACUCU.......................................GGGUUUGUAGCACAUUGCUGGC
GFLV01046660.1/385-314       ......................UUACUG.GGAAGCGA.GUGAGGGAAACACCUUA............................UUA..........................................CUAAGGGUACAUUAACUCAC
HAMY011136267.1/17-124       ....CCCAAGCGGGU....AGAAUUCAG.GGAAGAUC.GGGAGUGAAACAUGCCAG..C.......................CUAGC......................................GCCCUGGUAGCACAUUACCUCCC
HAMY010289101.1/84-240       ......UUGAGUGAG.....CUCGUUGG.AGAAGAAC.GCCGGUGAAACAUGUAUAGGGAGACCUCCUUGACCGUCGCUUCC.U...CACCCAAGGGGAUUCGGCAUCGGAGUCCA......AAGGCCUAUAUAGCACAUUACCCGGU
GGEZ01023376.1/2323-2425     .....CGGAGCUGG.......CGUGUGG.GGAAGAGG.GCUGGUGAAACAUCUGUGG..........................UGGC......................................GUACACAGAGCACAUUACCCAGC
GIFJ01641300.1/289-117       GUUGUA..GGUUG.......AACCUGAG.GGAAACUA.GUGAGGGAAACACACCCCCUG..................................................................CCGGGGGUGGUACAUUAACUCAC
GELB01009586.1/4493-4600     .CCCAAGCGGGUAG.......GAUCAGGUGGAAGAUA.GGCAGGGAAACACAACAGU.........................GUAGC......................................GAACUGUUGGCACAUUAACUGCC
HAMY011241849.1/33-148       UGCGCCAAGUCAUC...........AAU.AGAAGCAA.GCCAGGGAAACAUCUCGUAUC.......................AUUU............................A........UGAUCUUGAGAGCAAAUUAACUGGC
HAMY010478751.1/6-198        UACCUGCCGUA............GCAAG.AGAAGAUUCUCCAGGGAAACACUCUGGUGU.......................AUG........................................GCACCAGAGGCAUAUUAACUGGA
GFLP01286833.1/1232-1306     ......G..............UGAUCUA.GGAAGCAC.GCCAGGGAAACACGGACU........................................................................UGUCCGGCACAUUAACUGGC
HAMY010507670.1/339-269      ........................GCGG.AGAGGCAC.GCCAGGGAAACACAGUGAC..........................AA........................................UAGUCAUUGGAAAAUUAACUGGC
GFLP01012994.1/647-848       UUCGCC..............AUUGCUGG.AGAAGGAUCGUCAGGGAAACAUCCGUGCAC.......................ACGAG......................................GCACACGGAGCACAUUAACUGAC
GFLP01441414.1/641-842       UUCGCC..............AUUGCUGG.AGAAGGAUCGUCAGGGAAACAUCCGUGCAC.......................ACGAG......................................GCACACGGAGCACAUUAACUGAC
GFLP01288095.1/76-187        CAAUCGC.GGGUAG.......GACCUGG.GGAAGACU.AGGAGGGAAACACUUGAAG..........................GUCGC......................................GACUUCAGGGCACAUUAACUCCU
GFLP01329911.1/355-466       CAAUCGC.GGGUAG.......GACCUGG.GGAAGACU.AGGAGGGAAACACUUGAAG..........................GUCGC......................................GACUUCAGGGCACAUUAACUCCU
HAMY010552344.1/367-468      ......GGCGUGUG.......CUGUAUG.AGAAGGAAGUUCGGUGAAACAUGCUUGCCU.......................GCCG.......................................AGGCAAGUAGCACAUUACCCGAU
HALR01192984.1/243-152       ............AA.......GGUCUCC.CGAAGCUG.GCGAGGGAAACAUGCUGUUUA.......................CAGUG.....................................UUGAACAGUAGCACAUUAACUCGC
GAOU01012890.1/16-144        .GAACCAACAAU........A..GUUGA.GGAAGACG.GUACAGGAAACACCGCGUGGUUUUGAAAC.....CAUAAUAGC.................................AUGACUUUUUCAUGCCGCGGGCACAUUACUGUAC
GGEZ01023376.1/2884-2783     ......GCCGUAG.U.....ACUGUGUA.UGAAGACU.GGGAGGGAAACACAUCAG..C.......................GUAGC......................................GAACUGGUGGCACAUUAACUCCC
GFHZ01110905.1/44-117        .....................UGGGGGG.CGAAGCAC.GACGGUGAAACACCCAGG.........................................................................CUUGGGGCACAUUACCGUC
GFLP01271181.1/44-117        .....................UGGGGGG.CGAAGCAC.GACGGUGAAACACCCAGG.........................................................................CUUGGGGCACAUUACCGUC
GFLP01149597.1/702-629       .....................UGGGGGG.CGAAGCAC.GACGGUGAAACACCCAGG.........................................................................CUUGGGGCACAUUACCGUC
GFLP01283058.1/1206-1133     .....................UGGGGGG.CGAAGCAC.GACGGUGAAACACCCAGG.........................................................................CUUGGGGCACAUUACCGUC
GFLP01851030.1/254-181       .....................UGGGGGG.CGAAGCAC.GACGGUGAAACACCCAGG.........................................................................CUUGGGGCACAUUACCGUC
GFHZ01110905.1/473-392       .....................CUCCGUG.GGAAGCAA.GCCAGGGAAACACCAACUC.C.......................GUGA.........................................GGAUUGGGCACAUUAACUGGC
GFLP01149597.1/273-354       .....................CUCCGUG.GGAAGCAA.GCCAGGGAAACACCAACUC.C.......................GUGA.........................................GGAUUGGGCACAUUAACUGGC
GFLP01271181.1/473-392       .....................CUCCGUG.GGAAGCAA.GCCAGGGAAACACCAACUC.C.......................GUGA.........................................GGAUUGGGCACAUUAACUGGC
GFLP01283058.1/777-858       .....................CUCCGUG.GGAAGCAA.GCCAGGGAAACACCAACUC.C.......................GUGA.........................................GGAUUGGGCACAUUAACUGGC
GEHE01005692.1/325-248       .....................GAUUCCC.AGAGGCGU.AGUGGUGAAACACUAAUCAA.........................UCA.......................................AAUGAUUAGGCACAUUACCCACU
GIFI01514496.1/268-111       AGUUUCCUUGUUCG.......UAGUGAA.GGAAGAUC.GCUGUUGAAACACGCCUCGGA.......................AAGAG......................................UCCGUUGCGGCACAUUAUUCAGC
GIFI01514496.1/991-834       AGUUUCCUUGUUCG.......UAGUGAA.GGAAGAUC.GCUGUUGAAACACGCCUCGGA.......................AAGAG......................................UCCGUUGCGGCACAUUAUUCAGC
GIFI01514496.1/1714-1557     AGUUUCCUUGUUCG.......UAGUGAA.GGAAGAUC.GCUGUUGAAACACGCCUCGGA.......................AAGAG......................................UCCGUUGCGGCACAUUAUUCAGC
HAMY010496177.1/29-164       CUUCAAA.......CAGUGUGCUUUGUG.AGAAGAUG.CUGGAGGAAACCGCACGGUCG.......................GCCG.......................................AAACCGUGUGCAUAUUAACCCAG
HALR01183481.1/47-138        ......ACACAAGC.........UCUUC.CGAAGCAG.GCCAGGGAAACAUGCUGUUGA.......................CAUUG.....................................UUGAACAGUAGCACAUUAACUGGC
GELB01009586.1/5137-5031     ......UGUUG...G....CC.ACCCAG.UGAGGAUA.UCCAGGGAAACACUGUGUC..........................UUGA......................................CCGGCAUAGGUAUAUUAACUGGA
GFZV01012863.1/1819-1884     .......................ACCCC.AGAGGCAC.GACAGGGAAACACCCA.................................U.........................A................CGGGGCACAUUAACUGUC
GFZV01012863.1/6433-6498     .......................ACCCC.AGAGGCAC.GACAGGGAAACACCCA.................................U.........................A................CGGGGCACAUUAACUGUC
GFZV01012863.1/11047-11112   .......................ACCCC.AGAGGCAC.GACAGGGAAACACCCA.................................U.........................A................CGGGGCACAUUAACUGUC
HAMY010040351.1/14-335       UCUCUG.GUCCGC.C.....GUGGCGGG.AGAAGAUUCUCCAGGGAAACACACGUGUG........................AACG.......................................GCACACGUGGCACAUUAACUGGA
GEHG01006370.1/310-247       .........................GCG.GGAAGCUC.GGCAGGGAAACACGGA.................................C........................AA................UCCGGAACAUUAACUGCC
GFHZ01111161.1/316-388       .....................UAGGUUG.GGAAGCAU.GCCAGGGAAACACAACU................................U.........................C...............GGUUGGUAAAUUAACUGGC
GFLP01287108.1/316-388       .....................UAGGUUG.GGAAGCAU.GCCAGGGAAACACAACU................................U.........................C...............GGUUGGUAAAUUAACUGGC
GFLP01290863.1/86-27         .......................UUGGG.GGAAGAUG.GGUAGGGAAACAUC...................................GA.......................UA..................GAGUAAAUUAACUACC

                             ......>>>>>>.........>>>>>>>.....>>>>.<<<<<.......<<<<<<<<<..................................................................>>>>>>>>>.........>>>>>
                             ...........................222222.....1222.22221.......222222.....................................................................222222.........12222
                             ------○-○○○----------○○○●●●R-RGAAG●●●-R●●RGRGAAACAY●●●●○○○○-----------------------○○○○---------------------------------------○○○●●●●●RGCACAUUA●CY●●Y
```

```
GFLP01019157.1/196-271   .....................GGGAUGG.CGAAGCAG.GGCGGUGAAACAAUCCUG...........................GUCGG.........................................UAGGAUGCACAUUACCCGCC
GGFA01011333.1/384-316   ........................AGGG.GGAAGACAGACCAGGGAAACACAUAAUCU..............................C.........................C............GGGUUAUGGCACAUUAACUGGC
IACP01004784.1/205-112   .....................UCAAGGU.UGAAGCAC.GCAAGGGAAACACACUGUCACA.....................CGAUG.............................A........AUGCGAC.GUGGUACAUUAACUUGC
IACP01004784.1/527-434   .....................UCAAGGU.UGAAGCAC.GCAAGGGAAACACACUGUCACA.....................CGAUG.............................A........AUGCGAC.GUGGUACAUUAACUUGC
IACP01025381.1/120-213   .....................UCAAGGU.UGAAGCAC.GCAAGGGAAACACACUGUCACA.....................CGAUG.............................A........AUGCGAC.GUGGUACAUUAACUUGC
IACP01025381.1/442-535   .....................UCAAGGU.UGAAGCAC.GCAAGGGAAACACACUGUCACA.....................CGAUG.............................A........AUGCGAC.GUGGUACAUUAACUUGC
HALR01192983.1/272-180   ......UCACACAA.......GAUCUUC.CGAAGCCA.GCAAGGGAAACAUGCGCUUAAC......................AGCUU......................................GUUGAACGUAGCACAUUAACUUGC
                         ......>>>>>>.........>>>>>>>.....>>>>.<<<<<.......<<<<<<<<<...................................................................>>>>>>>>>.........>>>>>
                         ......................222222.....1222.22221.......222222.......................................................................222222.........12222
                         ------○-○○○----------○○○●●●R-RGAAG●●●-R●●RGRGAAACAY●●●●●●○○----------------------○○○○---------------------------------------○○○●●●●●RGCACAUUA●CY●●Y
```

8 hairpin-previously-published-four

For a description of this alignment, see Supplementary Table 2

8.1 Notes

Superscript numbers are used to annotate any previously-published-hairpin RNA that has special characteristics (described below). These numeric annotations will be used in Section8.2:. The meaning of the numbers are as follows.

[1] From Tobacco ringspot virus satellite RNA. This sequence is from the first published strain, whose hairpin ribozyme was first published in 1986. Beyond some published stems, little attempt has been made to determine the secondary structure of the non-core region.

[2] From Tobacco ringspot virus satellite RNA. This sequence is from a newer strain (isolate 62L), with a comparative analysis to the earlier strain published in 1993. Beyond some published stems, little attempt has been made to determine the secondary structure of the non-core region.

[3] From Arabis mosaic virus small satellite RNA. Little attempt has been made to determine the secondary structure of the non-core region.

[4] From Chicory yellow mottle virus satellite RNA. No attempt has been made to determine the secondary structure of the non-core region.

8.2 Multiple-sequence alignment

Each ribozyme in this alignment is identified by its genomic location in the form SEQID/START-END. SEQID (the sequence accession) is derived from sources such as IMG/M or GenBank. START is the coordinate of the 5′ nucleotide of the hairpin ribozyme, and END corresponds to the 3′ nucleotide. If START>END, then the ribozyme is on the reverse complement strand.

Nucleotides proposed to basepair as part of the consensus structure are shaded in color when they comprise Watson-Crick or G-U pairs. Otherwise they are shaded gray. Conserved stems are also indicated at the bottom of the alignment by angle brackets, where matching $<$ and $>$ denote base-paired columns. Below these angle brackets, the symbol "2" denotes base pairs exhibiting covariation according to the statistically well-founded R-scape method. "1" denotes base pairs exhibiting covariation according to R2R's simplistic method. "0" denotes base pairs that are not observed to mutate and "?" denotes base pairs that have a significant frequency of non-canonical nucleotides for Watson-Crick or G-U pairs ($> 5\%$). Below these base pair annotation is the consensus sequence: "R" = "A" or "G", "Y" = "C" or "U", red nucleotides: nucleotide identity conserved more than 97% of the time, black nucleotides: 90%, gray nucleotides: 75%, red circle (●): nucleotide is present 97% of the time, black circle (●): 90%, gray circle (●): 75%, white circle (○): 50%. All percentages of sequences just described (e.g. 97% conserved) assume that sequences have been weighted by the GSC algorithm implemented by the Infernal software package.

The alignment begins on the next page.

alignment positions 1 · · · 150

```
1 M14879.1/224-40   AAACAGAGAAGUCAACCAGAGAAACACACGUU..GUGGUAUAUUACCUGGU...................................................................................................
2 M17439.1/226-40   AAACAGAGAAGUCAACCAGAGAAACACACGUU..GUGGUAUAUUACCUGGU...................................................................................................
3 M21212.1/157-42   CAACAGCGAAGCGGAACGGCGAAACACACCUUGUGUGGUAUAUUACCCGUU...................................................................................................
4 D00685.1/306-39   CAACAGCGAAGCGCGCCAGGGAAACACACCAUGUGUGGUAUAUUAUCUGGCACCAUACGCGUGUGGUUUGCACCGGUCUGCAUAGAAAUGCGUCGCCGGCUGGACCCCAUAACGGGACCCCCCACCGCUCAUCACGAGGAAUACACUCG
                    <<<<<<....<<<<<<<<<.......<<<.....>>>.........>>>>><<<<<<<<...........................................................................................
                    .00000....011.11010.......000.....000.........0101100000000...........................................................................................
                    •AACAG•GAAGY••R•CRG•GAAACACAC••U∘∘GUGGUAUAUUAYCYG•Y---------------------------------------------------------------------------------------------------
```

alignment positions 151 · · · 300

```
1 M14879.1/224-40   .................ACG.CCUUGACUAGCUAC..ACCG.......CCGCGCCCACACGAAGGCUACAAUCUCCACCUCCAACGAGGUA..GCAGACCCCUACGCUACGCAGCA......CGGUUCA.....CUAGUUUA.CGU.GGU
2 M17439.1/226-40   .................ACG.CCUUGACUAACUAC..ACCG.......CCGCGCCCACACGAAGGCUGCUCCACGAGGUGGCAUCCCACCUCCACGAGGCACAACGCUACGCAGCA......CGGUUCA.....UUAGUUUA.CGU.GGU
3 M21212.1/157-42   .................GGAGUA..UCCAGA.................GGAAUUCACG............................................................................UCUGGU..CUCCCCAU
4 D00685.1/306-39   UGGAGCACGUAUGGUAGGACCG....UCCU.AUACCCUUACCACAGGAUAAA................................................................UCCCAGGUAACAUCAGCA.GGGAA..GGUCAUAA
                    .......>>>>>>>>..<<<<.....<<<<<......<<<<<...<<<....................................................................>>>..>>>>>........>>>>>...>>>>.<<<
                    .......00000000..1222.....20211......11110...000....................................................................000..01111........11202...2221.211
                    -----------------R••∘•∘--∘•C••∘∘∘∘∘--∘∘∘∘-------••R•∘∘∘∘∘∘----------------------------------------------------------------∘∘∘∘∘∘∘-----∘••G••--∘••Y∘•R•
```

alignment positions 301 · · · 340

```
1 M14879.1/224-40   GGC.UUUCG...........GCCACCUGACAGUCCUGUUU
2 M17439.1/226-40   GGC.UUUCG...........GCCACCUGACAGUCCUGUUU
3 M21212.1/157-42   GCC.CAACUC..........GGCAUGACGCAGUACUGUUU
4 D00685.1/306-39   GAGGAUUGAAGUAGUCUCACCUCUUAGCGCAGUACUGUUU
                    <<<<...............>>>>>>>>>>>....>>>>>>
                    0210...............0120112.110....00000.
                    G••-•••••∘---------••C•Y•••RCAGU•CUGUUU
```
